# Systematic Analysis of Adverse Event Reports for Sex Differences in Adverse Drug Events

**DOI:** 10.1038/srep24955

**Published:** 2016-04-22

**Authors:** Yue Yu, Jun Chen, Dingcheng Li, Liwei Wang, Wei Wang, Hongfang Liu

**Affiliations:** 1 Department of Medical Informatics, School of Public Health, Jilin University, Changchun, Jilin 130021, China; 2 Department of Health Sciences Research, Mayo Clinic, Rochester, Minnesota 55901, USA

## Abstract

Increasing evidence has shown that sex differences exist in Adverse Drug Events (ADEs). Identifying those sex differences in ADEs could reduce the experience of ADEs for patients and could be conducive to the development of personalized medicine. In this study, we analyzed a normalized US Food and Drug Administration Adverse Event Reporting System (FAERS). Chi-squared test was conducted to discover which treatment regimens or drugs had sex differences in adverse events. Moreover, reporting odds ratio (ROR) and *P* value were calculated to quantify the signals of sex differences for specific drug-event combinations. Logistic regression was applied to remove the confounding effect from the baseline sex difference of the events. We detected among 668 drugs of the most frequent 20 treatment regimens in the United States, 307 drugs have sex differences in ADEs. In addition, we identified 736 unique drug-event combinations with significant sex differences. After removing the confounding effect from the baseline sex difference of the events, there are 266 combinations remained. Drug labels or previous studies verified some of them while others warrant further investigation.

Adverse drug events (ADEs) are defined as “injury resulting from administration of a medication, including errors in administration”^1^. ADEs significantly increase the length of stay, economic burden, and risk of death for hospitalized patients^2^. An estimated 3% to 6.7% of hospitalized patients in the United States have ADEs, and 5% of those ADEs are fatal^3^. Given that all possible adverse effects of a drug cannot be anticipated on the basis of preapproval studies, which may test the drug with only limited durations in homogeneous or small populations^4^, drug postmarketing surveillance and report is crucial.

Much progress has been made to detect ADEs from diverse sources, including spontaneous adverse event reporting (AER) systems^5^, electronic health records (EHRs)^5^,^6^, and social media data^7^. Meanwhile, sex differences in frequent diseases and the corresponding outcomes and effects of therapies are more widespread than might be assumed^8^. Increasing evidence has shown that sex differences also exist in ADEs, which may be attributed to sex differences in pharmacokinetics and pharmacodynamics^9^,^10^, immunology^11^,^12^, and genetics^13^,^14^. In the era of personalized medicine^15^, detection of sex differences in ADEs can help design personalized treatment guidelines^16^.

A number of studies have analyzed sex differences in ADEs. Zopf *et al.*^6^ conducted a prospective study in 2371 inpatients data of several health departments in Germany and Israel. They found that antibacterials and anti-inflammatory agents may cause ADEs that have sex differences. In another study, Zelinkova *et al.*^17^ reviewed 843 EHR files of patients who took different immune suppressive therapies for inflammatory bowel disease (IBD) and reported that female IBD patients treated with anti–tumor necrosis factor might be more at risk for allergic ADEs. In a nationwide study in the Netherlands, after analyzing 9.2 million hospital admissions data between 2000 and 2005, sex differences in ADE-related hospital admissions were observed, especially with cardiovascular drugs^18^.

To monitor the adverse drug events, the US Food and Drug Administration (FDA) has developed its AER system, FAERS^19^. A spontaneous reporting system, FAERS contains AER data either voluntarily reported by patients and health care professionals or mandatorily reported by various pharmaceutical manufacturers. To date, more than 9 million spontaneous adverse event reports are contained in FAERS, which is updated quarterly^19^. The data compose 7 tables wherein the demographic characteristics table provides an opportunity to investigate sex differences in ADEs.

Multiple ADE detection studies based on FAERS have taken sex differences into consideration. In a retrospective cohort study using FAERS, Okon *et al.*^20^ found that the relative reporting ratio (RRR) of tigecycline-related pancreatitis was greater in women (14.432) than in men (3.125) by using a disproportionality analysis with Bayesian correction methodology. In the FAERS, Salk *et al.*^21^ found nearly twice as many reports of males than of females in whom ischemic colitis developed after interferon-α therapy for hepatitis C virus infection. In contrast, female patients with hepatitis C virus infection treated with interferon-β were more likely to have ischemic colitis. With the analysis of 19,182 immuno-deficiency virus (HIV) infected reports in FAERS, Xiao and colleagues^22^ assessed the sex differences in ADEs on human HIV infection treatment where non-nucleoside reverse transcriptase inhibitors (NNRTIs) induced more adverse events in female patients while protease inhibitors (PIs) and integrase inhibitors (INIs) induced more in male patients.

All previous studies on sex differences in ADEs using FAERS focused on specific diseases and drugs, and a systematic evaluation has not been available. Here, we reported a systematic analysis of sex differences of ADEs using data from FAERS for the top 20 long-term treatment regimens in the US pharmaceutical market in 2013 where drugs were normalized to RxNorm^23^ and aggregated into NDF-RT (National Drug File Reference Terminology)^24^ drug classes and adverse events coded using MedDRA (Medical Dictionary for Regulatory Activities)^25^ were classified into 26 System Organ Classes (SOCs). To our knowledge, our study is the first attempt to assess the extent of sex differences in ADEs across a wide range of treatments, to identify the drugs that show significant sex differences in 20 treatment regimens and 668 specific drugs, and to pinpoint the specific ADEs that account for the observed sex differences in the effects of these drugs.

## Result

### Detect sex difference in drug-event combination frequency distribution for treatment regimens/drugs

We first tested the treatment regimens/drugs that show sex difference in drug-event combination frequency distributions summarized at SOC level. Instead of analyzing one reported drug-event combination at a time, we jointly analyzed all the drug-event combinations passing the inclusion criteria. The results are shown in Table 1. Sex difference was detected in all the 20 treatment regimens. Moreover, of the 668 drugs, 307 were found to have sex differences in drug-event combination frequency distributions after Bonferroni *P* value adjustment. The test results of each treatment regimen or drug are provided in Supplementary Files S1 and S2.

Some of the sex differences summarized in Table 1 have been reported previously, mainly attributed to the sex-based differences in drug activity, such as pharmacokinetics and pharmacodynamics^9^,^10^,^14^,^26^. For example, verapamil has been reported to have greater absorption because of slower gastrointestinal transit times of women^27^. In women, β-blockers, and especially metoprolol, produce a greater reduction in systolic blood pressure and heart rate during exercise^28^. Table 2 shows examples that validate some sex differences in ADEs of drugs.

### Detect sex difference for specific drug-event combinations summarized at SOC level

**Figure 1:**
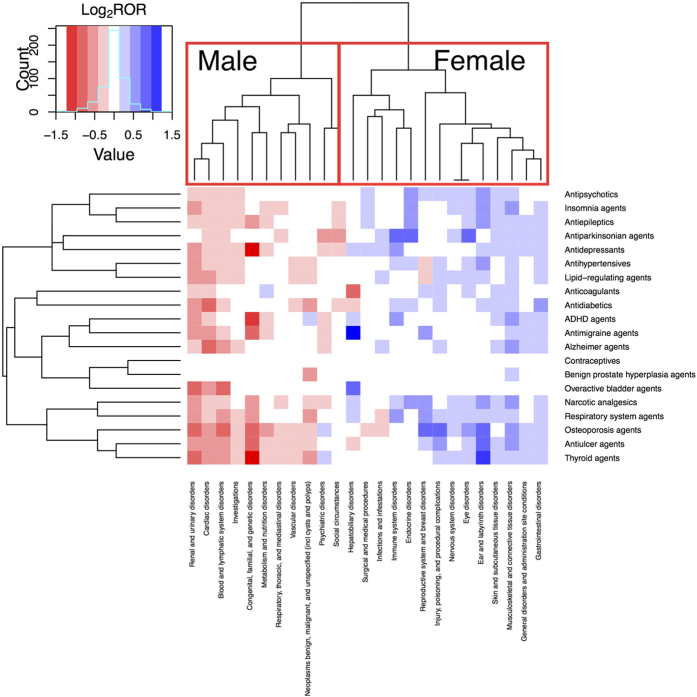
Heat Map of Sex Differences in drug-event combinations of 20 Treatment Regimens at the System Organ Class Category Level. Heat map shows positive signals of sex differences. The color of each cell is based on the logarithmic reporting odds ratio (ROR) for occurrence of drug-event combinations in the sex; blue cell represents the log_2_ ROR > 0, red cell represents the log_2_ ROR < 0; the darker the color, the greater the absolute value of ROR. *P* values were calculated using a proportion test and were adjusted by Bonferroni correction. Only drug-event combinations with sex differences significant at *P* ≤ .05 were selected. ADHD indicates attention-deficit/hypersensitivity disorder; incl, including.

The analysis was based on unique drug-event combinations with at least 50 occurrences in male patients and at least 50 occurrences in female patients. Figure 1 shows a 2-way clustered heat map where the x-axis is the SOC category and the y-axis is the treatment regimen. Only those specific ADE signals with sex differences significant at *P* ≤ 0.05 after Bonferroni correction were colored and the color of each cell is based on the logarithmic Reporting Odds Ratio (ROR) base 2 value. In the heat map, blue cells represent the log_2_ ROR > 0 (ROR > 1), indicating that the female patients are more likely to report these drug-event combinations. On the contrary, red cells represent the log_2_ ROR < 0 (ROR < 1), indicating that male patients are more susceptible to reporting these drug-event combinations. The SOC categories form two clusters by log2 ROR in Fig. 1. In the left cluster, most of cells in those 11 SOC categories are red, which means they have higher RORs in male patients, including renal and urinary disorders; congenital, familial, and genetic disorders; cardiac disorders; and blood and lymphatic system disorders, etc. The right cluster shows SOC categories with higher RORs in female patients, including ear and labyrinth disorders; musculoskeletal and connective tissue disorders; and gastrointestinal disorders. Some of these findings have been reported previously. For example, Zopf *et al.*^6^ found there was a significant difference (*P* = 0.0004) in the incidence of musculoskeletal system adverse events in female patients (12.6%, 44 adverse event occurrences in 349 female patients) vs. male patients (4.6%, 13 adverse event occurrences in 300 male patients). In addition, they also claimed that female patients (n = 133, 32.2%) tended to have more gastrointestinal disorders than male patients (n = 96, 26.6%)^29^. In contrast, Montastruc *et al.*^30^ found cardiovascular ADEs occurred more frequently in male patients (n = 8) than in females (n = 2) (*P* = 0.05).

Supplementary Figure S1 is a heat map showing the positive signals of sex differences in drug-event combinations for specific drugs at the SOC category level. A second round of Bonferroni correction was calculated to reduce false-positive signals. Among the 307 prescription drugs we tested, 178 had positive signals after adjustment. Similar to the heat map of treatment regimens, the detected ADE signals were grouped into 2 clusters where 15 SOC categories had higher RORs in female patients vs 10 categories in male patients. The only category that showed a difference between the clusters in Fig. 1 and Supplementary Figure S1 was social circumstances.

### Detect sex difference for specific drug-event combinations summarized at adverse events level

**Figure 2:**
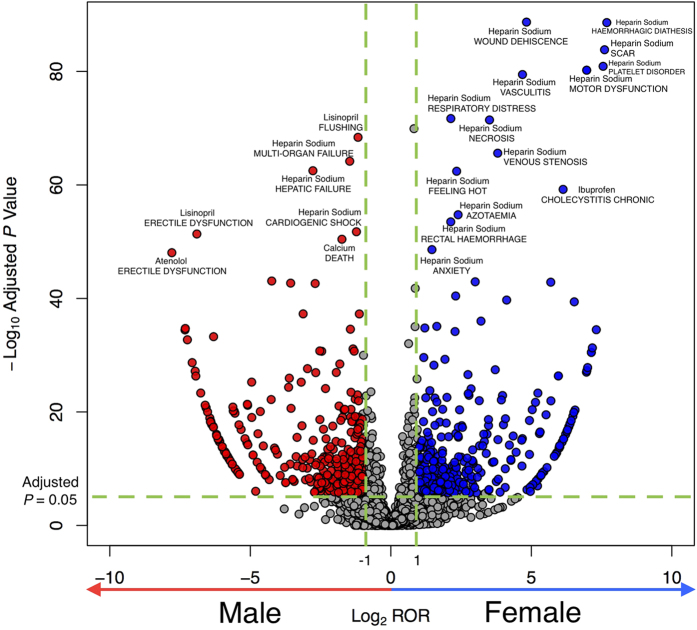
Volcano Plot of Significant Adverse Drug Event (ADE) Signals. In the volcano plot of ADE signals, the signal detection result shows the magnitude (log_2_ reporting odds ratio [ROR], x-axis) and significance (−log_10_ adjusted *P* value, y-axis) for sex- drug-event combinations associations of specific drugs. Each spot represents a specific drug- drug-event combination interaction. The dashed horizontal green line signals statistical significance threshold (*P* ≤ 0.05 after adjustment with Bonferroni correction). Two vertical green lines show the threshold of ROR (log_2_ ROR > 1 or < −1). The blue spots represent the drug-event combinations more frequently associated with female patients; the red spots, drug-event combinations more frequently associated with male patients.

Our analysis is based on unique drug-event combinations with at least 50 overall occurrences altogether in females and males. After *P* value adjustment, 736 positive signals of sex difference were identified in reported drug-event combinations (Supplementary Table S1), associated with 87 medications and 420 adverse events and detected to have sex differences (log_2_ ROR > 1 or log_2_ ROR < −1; adjusted *P* ≤ 0.05). Sex differences in drug-event combinations for specific drugs are summarized in Table 3 and Fig. 2, showing 332 reported drug-event combinations that had preference for female patients and 404 with preference for male patients. The anticoagulant regimen tends to have sex differences in reported drug-event combinations (130 with preference for female patients and 154 with preference for male patients). In contrast, no sex difference in reported drug-event combination is detected for drugs for overactive bladder.

Figure 2 is a volcano plot showing magnitude and significance for sex difference in drug-event combinations. The range of logarithmic ROR is from −7.79 to + 7.68.

We manually validated pairs with high ROR values. Some of them due to sex-specific adverse events (e.g., breast cancer, erectile dysfunction, prostate cancer), and some due to the drugs are sex-specific (e.g., medroxyprogesterone). We also noticed some of them warrant further investigation. For example, among all drugs, heparin and ibuprofen are the top 2 drugs associated with drug-event combinations that have sex differences. Of 621 adverse events associated with heparin, 273 have sex differences. At the SOC category level, we found that heparin-induced blood and lymphatic system disorders greater for female patients (log_2_ ROR, 0.237; *P* = 1.33E-14). According to drug labels on the FDA website, heparin has been reported to have a high risk of bleeding, especially in female patients older than 60 years^31^. Other studies have shown that women taking heparin were more susceptible to bleeding episodes^32^. At the adverse events level, we discovered some clinically significant signals of sex difference in drug-event combinations for heparin. For example, the activated partial thromboplastin time of women is longer than men after taking heparin (log_2_ ROR, 1.12; *P* = 0.04). This ADE has been known and might be caused by pharmacokinetics sex differences^33^. However, some ADE signals (eg, alopecia, amnesia, urticaria) had sex differences detected by our study, but no evidence was found in the literature.

For ibuprofen, 34 of 483 drug-event combinations had sex differences. Ibuprofen is known to be associated with some ADEs that have sex differences^14^,^34^. We detected that female patients taking ibuprofen tend to have hepatobiliary disorders (log_2_ ROR, 1.38; *P* = 1.55E-102). We also detected that they tend to have upper gastrointestinal symptoms, such as upper abdominal pain (log_2_ ROR, 1.01; *P* = 3.61E-8) and dyspepsia (log_2_ ROR, 1.11; *P* = 0.001), which was reported previously^35^. The sex difference may also be due to pharmacokinetics, of which 1 study showed that women had a 2-fold greater volume of ibuprofen distribution, adjusted for body weight^36^.

To filter out drug-event combinations caused by potential sex differences in reporting adverse events, we further analyzed the identified associations using logistic regression, accounting for the potential reporting bias due to sex. After Bonferroni correction, 266 drug-event combinations remained significant. These associations were free of the potential confounding effect of the reporting bias (Supplementary Table S2), thus were more reliable.

## Discussion

We identified a list of drug-event combinations with sex differences; these findings can be used to study the sex difference in pharmacokinetics and pharmacodynamics, as well as provide evidence for tailored medication prescription and instruction.

Despite the obvious physical and physiological sex differences, sex differences in ADEs are rarely considered in clinical treatment^37^. Lack of awareness among physicians may be one of the main reasons. A survey has shown that information regarding sex aspects of medicine was not fully embedded in the existing curriculum of US medical schools^38^. We randomly picked 20 drug-event combinations for diabetes mellitus and 20 drug-event combinations for hypertension and asked 2 primary care physicians to identify the ADEs with sex differences. Both physicians were not aware of any sex difference in these drug-event combinations, even though our data suggested that half of the DRUG-EVENT COMBINATIONs had sex differences.

With millions of reported drug-event combination records, FAERS provides a distinct opportunity for mining ADEs with sex differences. Besides FAERS, other data sources such as EHRs and social media have been used to detect ADEs. Those other data sources provide us a good opportunity to validate our findings. For example, one of the unique drug-event combinations is (“Heparin Sodium”, “Pheumonitis”). We observed males over ten times more to report adverse events of “Phenumonitis” after taking “Heparin Sodium”. While checking Mayo EHR records, we observed an odd ratio of female comparing to male to have “Pheumonitis” is 1.07. Combining these two sources, the observed sex difference is most likely to be true.

## Methods

### FAERS data normalization

More than 20% of FAERS records have been duplicated^39^. Moreover, in contrast to the adverse event terms, which are standardized and coded by the MedDRA^25^ (http://www.meddra.org), the drug names in FAERS are not normalized. Instead, they may be full names, trade names, and abbreviations, and spelling mistakes are not uncommon, which further complicate downstream analysis. Previously, we have standardized the FAERS data into 3 steps^40^. The first step is data de-duplication, where redundant reports were deleted in compliance with the suggested method of the FDA. In the second step for drug name normalization, RxNorm^23^, a standard nomenclature that provides a normalized naming system for clinical drugs, was used. Drug names, together with administration route and dose information, were mapped to concept-unique identifiers in RxNorm through a medication information extraction system named MedEx^41^. The adverse event terms were matched to MedDRA’s preferred term code and classified into MedDRA System Organ Class (SOC). In the third step, drugs were aggregated into classes by NDF-RT^24^, a drug terminology dictionary belonging to RxNorm.

We processed FAERS data files from 2004 through 2011 (Dataset home page: http://informatics.mayo.edu/adepedia/index.php/Download). After standardization, a total of 37,029,228 unique drug-event combinations remained. All drug names in FAERS were normalized into 14,489 RxNorm drug terms, and 10,221 (71%) were aggregated into NDF-RT drug classes. The 14,740 MedDRA adverse event terms were linked with adverse events and were classified into 26 SOCs^40^. In total, 36,198,178 records (97.75%) have sex information, with 14,550,581 (39.29%) from men and 21,647,597 (58.46%) from women. These standardized data were built into 2 tables ultimately: 1) drug classification information based on NDF-RT and 2) drug event co-occurrence records with patient sex information (37,029,228 records). When limited to the most frequent 20 treatment regimens of the United States and excluding 15,038 records in the “pregnancy, puerperium, and perinatal conditions” SOC category, 6,763,148 adverse event records of 668 drugs were available for analysis. Among these records, 3,845,398 were from female patients (56.86%).

### Selection and aggregation criteria

In this study, we selected drugs used in 20 leading US treatment regimens in 2013, according to a report of the IMS Institute for Healthcare Informatics, of IMS Health Inc (original name, Intercontinental Marketing Services) (Supplementary Table S3)^42^. The report also provided the drug classes used for these treatment regimens (Supplementary Table S4), and we used the drug class information to find specific drugs. On the basis of the NDF-RT drug classification system in our ARES data mining set, we identified most drugs belonging to these regimens. However, the NDF-RT was incomplete. Therefore, 2 other drug dictionaries, Micromedex (accessible at http://www.micromedexsolutions.com/micromedex2/librarian) drug class and World Health Organization Anatomical Therapeutic Chemical classification system^43^, were chosen as supplementary to NDF-RT. Supplementary Table S5 shows how we collected information on angiotensin-converting enzyme (ACE) inhibitor drugs for hypertension. Only 10 drug active-ingredient names were cited in the NDF-RT ACE inhibitor drug class, whereas the Micromedex ACE inhibitor drug class had 17 drugs and the World Health Organization Anatomical Therapeutic Chemical had 16. We searched the specific drugs in NDF-RT and identified an additional 3 drugs for the ACE inhibitor class. We retrieved a concept-unique identifier for each drug as the retrieval conditions. We also aggregated 14,740 unique drug-event combinations into 26 classes on the basis of MedDRA.

### Statistical methods

We applied χ2 tests to discover sex difference in overall drug-event combinations for specific treatment regimens or drugs and sex difference in unique drug-event combinations. The following describes the details.

### Detect sex difference in drug-event combination distributions for treatment regimens/drugs

We first detect sex difference for a treatment regimen or a specific drug, considering all drug-event combinations summarized at the SOC level. The objective is to identify treatment regimens or drugs that show overall difference in drug-event combination frequency distributions. Thus the proposed test is an overall test and is designed to identify treatment regimens/drugs that show an overall shift in drug-event combination frequency distribution. It also alleviates the multiple testing burden by avoiding testing individual drug-event combinations separately. Consider the following contingency table of each treatment regimen or drug for all adverse events (1 … n).


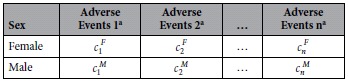


where the value of each cell represents the reported counts of unique drug‐event combinations summarized at System Organ Class (SOC) level. Denote 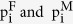 the probability of reporting the *i*th drug‐event combinations for females and males respectively. The null hypothesis of no sex difference for a given treatment regimen/drug can be expressed as:


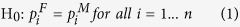


We test the null hypothesis by a χ2 test for the contingency table. To reduce the influence of sex-specific events on the test, we removed data in the category pregnancy, puerperium, and perinatal conditions, and we grouped all other events into 25 SOC categories. To correct for multiple testing and provide a strong control of the family-wise error rate, the P values were adjusted using Bonferroni correction.

### Detect sex difference for specific drug-event combinations

The overall test above points to the treatment regimens or drugs that show an overall shift in drug-event combination frequency distributions but it is unable to tell the specific adverse events accounting for the overall difference. We next proceeded to detect sex differences for specific drug-event combinations. To reduce the number of tests, we filtered out the drug-event combinations with occurrences less than 50. Instead of testing whether the ratio of the occurrences of an adverse event for male and female patients was the same as the baseline ratio (i.e., ratio of drug use), which was unavailable from the database, we tested whether the proportion of a target adverse event, among all reported events, was the same between the males and females. A similar χ^2^ test for a 2*2 contingency table was used to detect the sex-differential drug-event combinations. An adjusted reporting odds ratio (ROR) was used to quantify the difference (i.e., effect size) between the female and male sexes for a specific adverse event.

For a unique drug-event combination, the ROR was defined as the following:


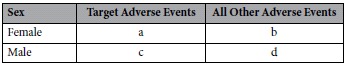


a: the number of female patients with target drug-events combinations. b: the number of female patients with target drugs but not target events. c: the number of male patients with target drug-events combinations. d: the number of male patients with target drugs but not target events.


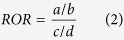


To make the ROR value symmetric, log_2_ ROR was calculated in our study. An ROR greater than 1 (log_2_ ROR > 0) indicates that the female patients are more likely to report a given adverse event than male patients. On the other hand, when the ROR value is less than 1, male patients tend to report the adverse event more frequently. All *P* values of the tests were adjusted through Bonferroni correction to account for multiple testing. Drug-event heat maps were used for visualization. All data analyses and graphical representations were performed with R 3.1.2 software(R Development Core Team).

In the specific tests, we are essentially testing the equality of proportions of specific drug-event combinations instead of the absolute numbers of drug-event combinations to avoid the bias caused by the larger observed number of drug-event combinations in female patients. For instance, we detected verapamil have sex difference in the overall test. The number of female patients is 24084 and male patients is 12681. In addition, we want to identify specific drug-event combinations for verapamil at SOC level. After the Chi-square test, we find there is no difference in “General disorders and administration site conditions” between female patients (n = 1571, 12.39%) and male patients (n = 3001, 12.46%)(*P* = 1). However, we detect that males (n = 1239, 9.77%) have a higher risk in “Cardiac disorders” than females (n = 1794, 7.45%)(log_2_ ROR, −0.43; *P* = 1.31E-9).

Note that there is still possibility that some sex-associated drug-event combinations identified by chi-square tests were, in fact, caused by potential sex bias in reporting a specific ADE. In other words, they are not drug-specific. For example, females tend to report more *alopecia* events. When we test the drug-event combinations related to *alopecia*, the chi-square tests will tend to identify them as sex-associated drug-event combinations. Clearly, these associations are less interesting. To rule out the potential confounding effect by the ADE reporting bias due to sex, we further analyzed the unique drug-event combination identified in the previous step using a logistic model, accounting for the baseline sex difference. Specifically, we use the following model:


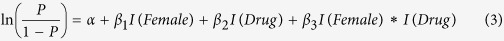


where *I*(Female) and *I*(Drug) are indicator variables, taking on values of 1 if the sex is female or the drug is used, *α* represents the baseline event-reporting probability (Male, No drug use), *β*_*1*_ and *β*_*2*_represent the increase of the odds if the sex is female and if the drug is used, respectively, and *β*_*3*_is the parameter of interest, which quantifies the drug-sex interaction effects, i.e., the drug effects depend on the sex. In the model, the parameter *β*_*1*_is used to account for the sex difference in reporting a specific ADE. We use the Wald test to test for the interaction effects *β*_*3*_. Bonferroni correction is also used to adjust the *P* values.

## Additional Information

**How to cite this article**: Yu, Y. *et al.* Systematic Analysis of Adverse Event Reports for Sex Differences in Adverse Drug Events. *Sci. Rep.* **6**, 24955; doi: 10.1038/srep24955 (2016).

## Supplementary Material

Supplementary document

Supplementary Dataset 1

Supplementary Dataset 2

## Figures and Tables

**Table 1:** Results of sex difference of unique drug-event combinations for 20 treatment regimens.

Treatment Regimens	Unique Drug-Event Combinations			Drug No.	Sex Difference, Drug No. (P ≤ .05)^a^
	Total	Female Patients, No. (%)	Male Patients, No. (%)		
Antihypertensives	1220150	648214 (53.13%)	571936 (46.87%)	86	50
Lipid-regulating agents	324278	156939 (48.40%)	167339 (51.60%)	27	15
Antidepressants	577277	371944 (64.43%)	205333 (35.57%)	58	32
Antiulcer agents	303496	169574 (55.87%)	133922 (44.13%)	10	8
Narcotic analgesics	583271	332976 (57.09%)	250295 (42.91%)	48	26
Antidiabetics	517498	279363 (53.98%)	238135 (46.02%)	36	19
Thyroid agents	154637	119451 (77.25%)	35186 (22.75%)	5	3
Antiepileptics	530893	311807 (58.73%)	219086 (41.27%)	45	23
Contraceptives	41794	40765 (97.54%)	1029 (2.46%)	22	8
Respiratory system agents	423719	234555 (55.36%)	189164 (44.64%)	55	17
Anticoagulants	452923	207137 (45.73%)	245786 (54.27%)	30	10
ADHD agents	171717	104047 (60.59%)	67670 (39.41%)	33	12
Insomnia agents	514712	312534 (60.72%)	202178 (39.28%)	32	18
Benign prostate hyperplasia agents	78622	10942 (13.92%)	67680 (86.08%)	15	6
Antipsychotics	251448	123905 (49.28%)	127543 (50.72%)	56	20
Osteoporosis agents	284008	217217 (76.48%)	66791 (23.52%)	29	13
Overactive bladder agents	27905	18849 (67.55%)	9056 (32.45%)	13	4
Antiparkinsonian agents	70523	35841 (50.82%)	34682 (49.18%)	35	15
Antimigraine agents	176973	112332 (63.47%)	64641 (36.53%)	21	5
Alzheimer agents	57304	37006 (64.58%)	20298 (35.42%)	12	3
Total	6763148	3845398 (56.86%)	2917750 (43.14%)	668	307

Abbreviation: ADHD, attention-deficit/hyperactivity disorder.

^a^ *P* value of the test is adjusted through Bonferroni correction.

**Table 2:** Validations of sex differences.

Drug	Sex Difference in Drug Activity	Implications
Verapamil^27^	Pharmacokinetics/Absorption	Greater gut absorption in women
Diazepam^44^	Pharmacokinetics/Distribution	Larger distribution in women
Midazolam^45^	Pharmacokinetics/Metabolism	Higher clearance in women
Gabapentin^10^	Pharmacokinetics/Excretion	Lower renal clearance in women
Atenolol, Metoprolol… (β-Blockers)^28^	Pharmacodynamics	Greater reduction in blood pressure in women
Fluoxetine, Sertraline… (Selective serotonin reuptake inhibitors)^46^	Pharmacodynamics	Greater effect in women

**Table 3:** Distribution of sex difference drug-event combinations in treatment regimens.

Treatment Regimens	Drug No.	Female-Related drug-event combinations (log_2_ ROR ≥ 1)	Male-Related drug-event combinations (log_2_ ROR ≤ −1)	Total ADEs Signals
Antihypertensives	17	42	47	89
Lipid-regulating agents	5	9	14	23
Antidepressants	8	7	21	28
Antiulcer agents	5	13	15	28
Narcotic analgesics	7	18	26	44
Antidiabetics	7	28	23	51
Thyroid agents	1	4	8	12
Antiepileptics	8	13	24	37
Contraceptives	1	2	0	2
Respiratory system agents	3	20	13	33
Anticoagulants	3	130	154	284
ADHD agents	2	0	2	2
Insomnia agents	1	2	0	2
Benign prostate hyperplasia agents	1	0	1	1
Antipsychotics	5	4	5	9
Osteoporosis agents	4	9	36	45
Overactive bladder agents	0	0	0	0
Antiparkinsonian agents	3	0	4	4
Antimigraine agents	1	25	9	34
Alzheimer agents	1	6	2	8
Total	83	332	404	736

Abbreviations: ADHD, attention-deficit/hyperactivity disorder; ADE, adverse drug events; ROR, reporting odds ratio.
