# Supplementary material for: Systematic Analysis of Adverse Event Reports for Sex Differences in Adverse Drug Events
Source: Sci Rep. 2016 Apr 22;6:24955. doi: 10.1038/srep24955 (PMC4840306; doi:10.1038/srep24955)
Supplement: Supplementary document [file srep24955-s1.pdf]

# Systematic Analysis of Adverse Event Reports for sex Differences in Adverse Drug Events

**Yue Yu[1,2], Jun Chen[2], Dingcheng Li[2], Liwei Wang[1], Wei Wang[1,*], and Hongfang Liu[2,*]**

[1]Department of Medical Informatics, School of Public Health, Jilin University, Changchun, Jilin 130021, China
[2]Department of Health Sciences Research, Mayo Clinic, Rochester, Minnesota 55901, USA
[*]corresponding.author: w_w@jlu.edu.cn
[*]corresponding.author: Liu.Hongfang@mayo.edu

**Page 2 Supplemental Figure S1.** HeatMap of sex differences in drug-event combinations at the SOC Level.

**Page 3 Supplementary Table S3.** The frequency information of the top 20 treatment regimens at the United States in 2013.

**Page 4-5 Supplementary Table S4.** Drug groups based on IMS Institute of Healthcare Informatics Definitions.

**Page 6-7 Supplementary Table S5.** ACE Inhibitor drug class information in three terminologies.

**Page 8-27 Supplementary File S1.** Overall test results of 20 treatment regimens.

**Page 28-334 Supplementary File S2.** Overall test results of 307 specific drugs.

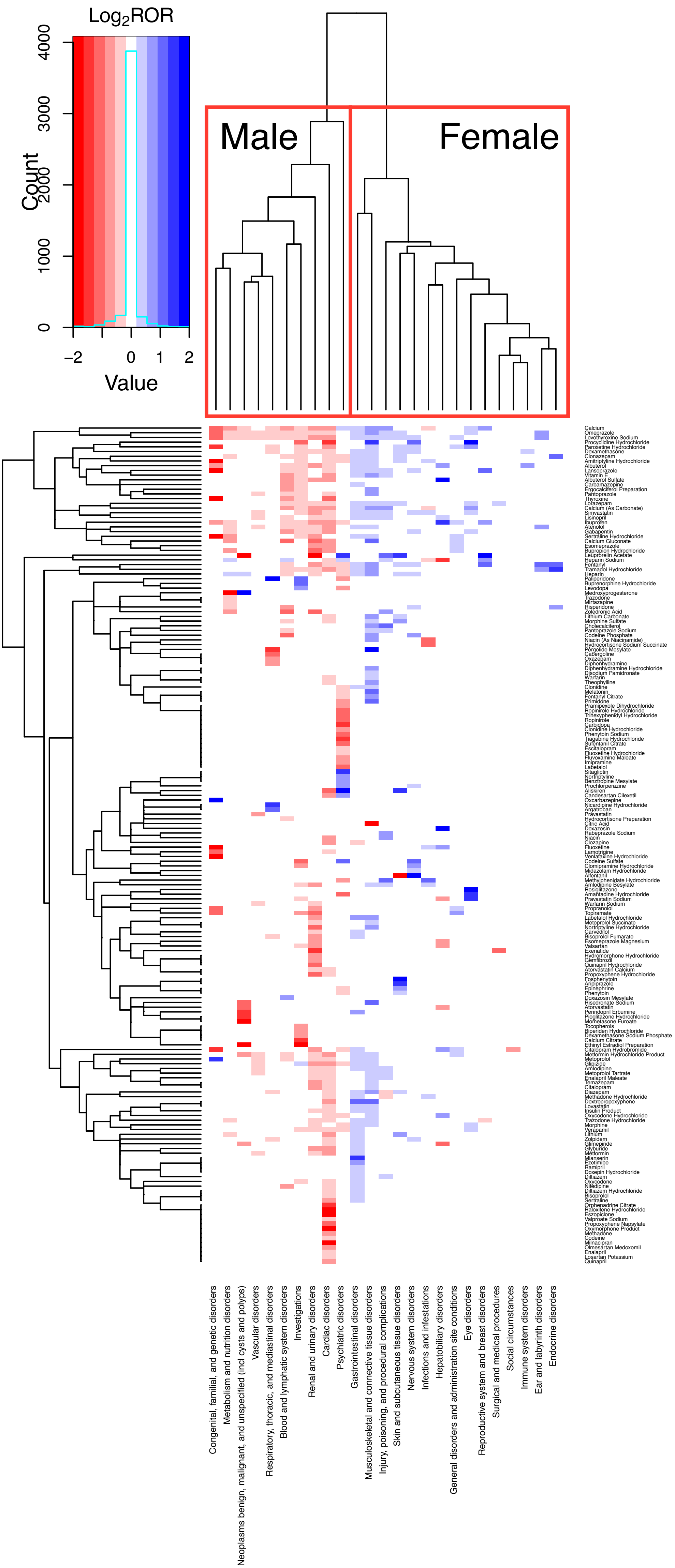

Log2ROR
Count
4000
3000
2000
1000
0
−2
0
1
2
Value
Male
Female
Calcium
Omeprazole
Levothyroxine Sodium
Procyclidine Hydrochloride
Paroxetine Hydrochloride
Dexamethasone
Clonazepam
Amitriptyline Hydrochloride
Albuterol
Lansoprazole
Vitamin E
Albuterol Sulfate
Carbamazepine
Ergocalciferol Preparation
Pantoprazole
Thyroxine
Lorazepam
Calcium (As Carbonate)
Simvastatin
Lisinopril
Ibuprofen
Atenolol
Gabapentin
Sertraline Hydrochloride
Calcium Gluconate
Esomeprazole
Bupropion Hydrochloride
Leuprorelin Acetate
Heparin Sodium
Fentanyl
Tramadol Hydrochloride
Heparin
Paliperidone
Buprenorphine Hydrochloride
Levodopa
Medroxyprogesterone
Trazodone
Mirtazapine
Risperidone
Zoledronic Acid
Lithium Carbonate
Morphine Sulfate
Cholecalciferol
Pantoprazole Sodium
Codeine Phosphate
Niacin (As Niacinamide)
Hydrocortisone Sodium Succinate
Pergolide Mesylate
Cabergoline
Oxazepam
Diphenhydramine
Diphenhydramine Hydrochloride
Disodium Pamidronate
Warfarin
Theophylline
Clonidine
Melatonin
Fentanyl Citrate
Primidone
Pramipexole Dihydrochloride
Ropinirole Hydrochloride
Trihexyphenidyl Hydrochloride
Ropinirole
Carbidopa
Clonidine Hydrochloride
Phenytoin Sodium
Tiagabine Hydrochloride
Sufentanil Citrate
Escitalopram
Fluoxetine Hydrochloride
Fluvoxamine Maleate
Imipramine
Labetalol
Sitagliptin
Nortriptyline
Benztropine Mesylate
Prochlorperazine
Aliskiren
Candesartan Cilexetil
Oxcarbazepine
Nicardipine Hydrochloride
Argatroban
Pravastatin
Hydrocortisone Preparation
Citric Acid
Doxazosin
Rabeprazole Sodium
Niacin
Clozapine
Fluoxetine
Lamotrigine
Venlafaxine Hydrochloride
Codeine Sulfate
Clomipramine Hydrochloride
Midazolam Hydrochloride
Alfentanil
Methylphenidate Hydrochloride
Amlodipine Besylate
Rosiglitazone
Amantadine Hydrochloride
Pravastatin Sodium
Warfarin Sodium
Propranolol
Topiramate
Labetalol Hydrochloride
Metoprolol Succinate
Nortriptyline Hydrochloride
Carvedilol
Bisoprolol Fumarate
Esomeprazole Magnesium
Valsartan
Exenatide
Hydromorphone Hydrochloride
Gemfibrozil
Quinapril Hydrochloride
Atorvastatin Calcium
Propoxyphene Hydrochloride
Fosphenytoin
Aripiprazole
Epinephrine
Phenytoin
Doxazosin Mesylate
Risedronate Sodium
Atorvastatin
Perindopril Erbumine
Pioglitazone Hydrochloride
Mometasone Furoate
Tocopherols
Biperiden Hydrochloride
Dexamethasone Sodium Phosphate
Calcium Citrate
Ethinyl Estradiol Preparation
Citalopram Hydrobromide
Metformin Hydrochloride Product
Metoprolol
Glipizide
Amlodipine
Metoprolol Tartrate
Enalapril Maleate
Temazepam
Citalopram
Diazepam
Methadone Hydrochloride
Dextropropoxyphene
Lovastatin
Insulin Product
Oxycodone Hydrochloride
Trazodone Hydrochloride
Morphine
Verapamil
Lithium
Zolpidem
Glimepiride
Glyburide
Metformin
Mianserin
Ezetimibe
Ramipril
Doxepin Hydrochloride
Diltiazem
Oxycodone
Nifedipine
Diltiazem Hydrochloride
Bisoprolol
Sertraline
Orphenadrine Citrate
Raloxifene Hydrochloride
Eszopiclone
Valproate Sodium
Propoxyphene Napsylate
Oxymorphone Product
Methadone
Codeine
Milnacipran
Olmesartan Medoxomil
Enalapril
Losartan Potassium
Quinapril
Congenital, familial, and genetic disorders
Metabolism and nutrition disorders
Neoplasms benign, malignant, and unspecified (incl cysts and polyps)
Vascular disorders
Respiratory, thoracic, and mediastinal disorders
Blood and lymphatic system disorders
Investigations
Renal and urinary disorders
Cardiac disorders
Psychiatric disorders
Gastrointestinal disorders
Musculoskeletal and connective tissue disorders
Injury, poisoning, and procedural complications
Skin and subcutaneous tissue disorders
Nervous system disorders
Infections and infestations
Hepatobiliary disorders
Eye disorders
General disorders and administration site conditions
Reproductive system and breast disorders
Surgical and medical procedures
Social circumstances
Immune system disorders
Ear and labyrinth disorders
Endocrine disorders

**Supplementary Table S3.** Treated Patient Number in Top 20 Treatment Regimens of the United States in 2013

| **Treatment Regimens** | **Patients Receiving Therapy, No. in Millions** |
|---|---|
| Antihypertensives | 45.7 |
| Lipid-regulating agents | 24.4 |
| Antidepressants | 22.3 |
| Antiulcer agents | 15.3 |
| Narcotic analgesics | 14.9 |
| Antidiabetics | 14.0 |
| Thyroid agents | 13.6 |
| Antiepileptics | 11.4 |
| Contraceptives | 9.1 |
| Respiratory system agents | 8.9 |
| Anticoagulants | 6.0 |
| ADHD agents | 5.7 |
| Insomnia agents | 5.3 |
| Benign prostatic hyperplasia agents | 4.5 |
| Antipsychotics | 3.5 |
| Osteoporosis agents | 2.1 |
| Overactive bladder agents | 1.7 |
| Antiparkinsonian agents | 1.6 |
| Antimigraine agents | 1.3 |
| Alzheimer disease agents | 1.2 |

**Abbreviations: ADHD, attention-deficit/hyperactivity disorder;**

**Supplementary Table S4.** Drug Groups Based on IMS Institute of Healthcare Informatics Definitions

| Treatment Regimens | Drug Group |
|---|---|
| Antihypertensives | ACE inhibitors, angiotensin receptor antagonists, renin inhibitors, adrenergic β-antagonists, calcium channel blockers |
| Lipid-regulating agents | HMG-CoA reductase inhibitors, fibrates, bile acid sequestrants, other hypolipidemic agents |
| Antidepressants | Selective serotonin reuptake inhibitors, serotonin norepinephrine reuptake inhibitors, monoamine oxidase inhibitors, other antidepressants |
| Antiulcer agents | Proton pump inhibitors |
| Narcotic analgesics | Opioid analgesics |
| Antidiabetics | Insulins and analogues, hypoglycemic agents |
| Thyroid agents | Thyroid hormonal preparations |
| Antiepileptics | Barbiturates, benzodiazepines, fatty acids, hydantoins, carboxamides, oxazolidines, succinimides, other antiepileptics |
| Contraceptives | Hormonal contraceptives |
| Respiratory system agents | Antiasthmatic agents, bronchodilator agents |
| Anticoagulants | Anticoagulants, platelet aggregation inhibitors |
| ADHD agents | Sympathomimetics, other psychostimulants |
| Insomnia agents | Nonbarbiturate hypnotics, melatonin receptor agonists, other hypnotics |
| Benign prostatic hyperplasia agents | Adrenergic α-antagonists, 5-α reductase inhibitors |

| | |
|---|---|
| Antipsychotics | Typical antipsychotics, atypical antipsychotics |
| Osteoporosis agents | Diphosphonates, other bone density conservation agents |
| Overactive bladder agents | Urinary antispasmodics |
| Antiparkinsonian agents | Cholinergic antagonists, dopamine agents |
| Antimigraine agents | Ergot alkaloids, serotonin receptor agonists, other antimigraine preparations |
| Alzheimer disease agents | Acetylcholinesterase inhibitors, other drugs |

Abbreviations: ACE, angiotensin-converting enzyme; ADHD, attention-deficit/hyperactivity disorder; HMG-CoA, 3-hydroxy-3-methylglutaryl coenzyme A.

**Supplementary Table S5.** ACE Inhibitor Drug Class in 3 Terminology Dictionaries

| NDF-RT | Micromedex | WHO ATC | Final |
|---|---|---|---|
| Benazepril | Alacepril[a] | Benazepril | Benazepril |
| Captopril | Benazepril | Captopril | Captopril |
| Enalapril | Captopril | Cilazapril[b] | Cilazapril[c] |
| Enalaprilat | Cilazapril[a] | Delapril[b] | Enalapril |
| Fosinopril | Delapril[a] | Enalapril | Enalaprilat |
| Lisinopril | Enalapril | Fosinopril | Fosinopril |
| Moexipril | Enalaprilat | Imidapril[b] | Lisinopril |
| Perindopril | Fosinopril | Lisinopril | Moexipril |
| Quinapril | Imidapril[a] | Moexipril | Perindopril |
| Ramipril | Lisinopril | Perindopril | Quinapril |
| | Moexipril | Quinapril | Ramipril |
| | Perindopril | Ramipril | Spirapril[c] |
| | Quinapril | Spirapril[b] | Trandolapril[c] |
| | Ramipril | Trandolapril[b] | |
| | Spirapril[a] | Temocapril[b] | |
| | Trandolapril[a] | Zofenopril[b] | |
| | Zofenopril[a] | | |

Abbreviations: ACE, angiotensin-converting enzyme; ATC, Anatomic Therapeutic Chemical; NDF-RT, National Drug File–Reference Terminology; WHO, World Health Organization.

[a] Specific drugs in Micromedex ACE inhibitor drug class.

[b] Specific drugs in WHO ATC ACE inhibitor drug class.

[c] Supplementary drugs can be searched in our Adverse Event Reporting System Data Mining set.

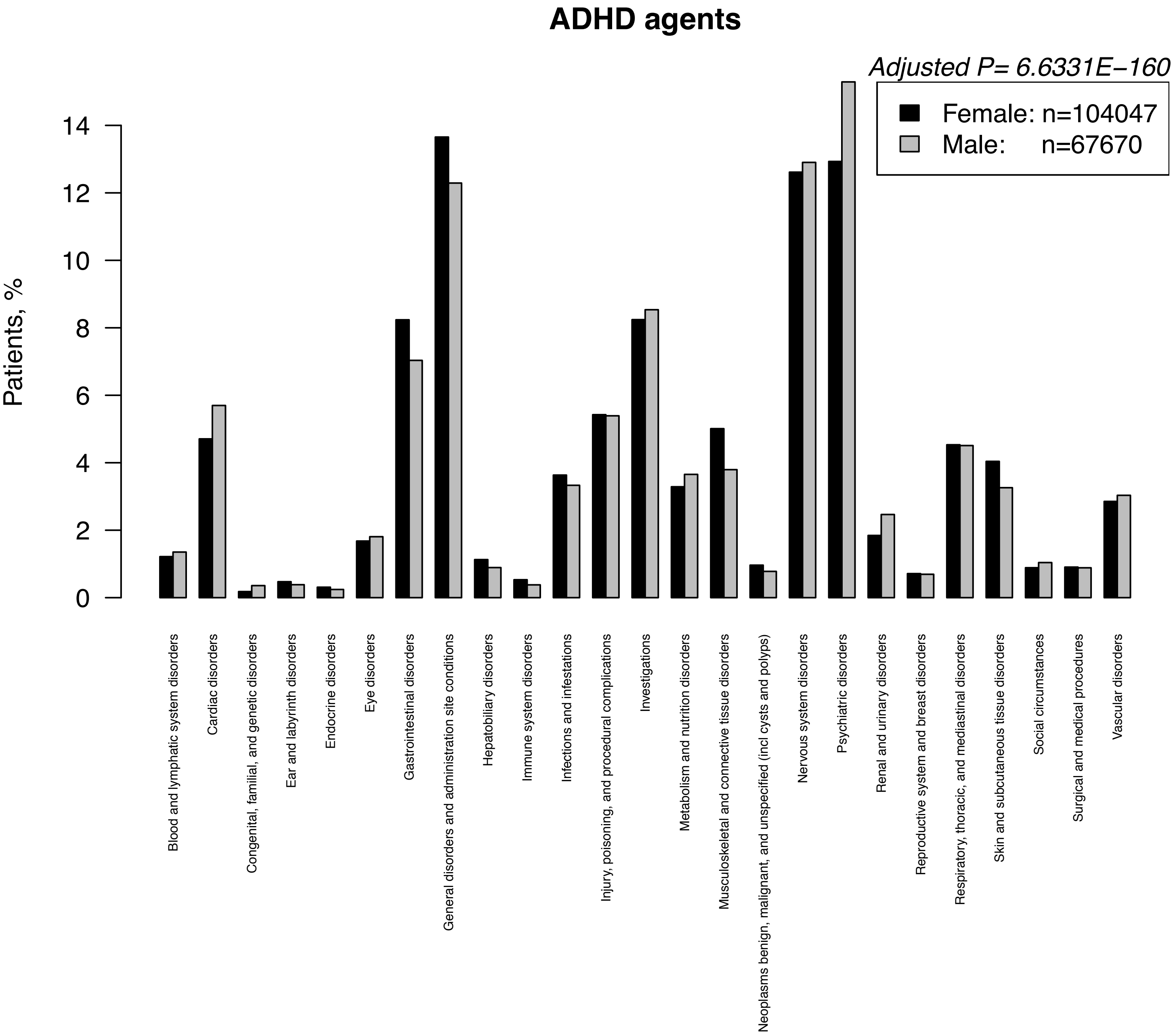
ADHD agents
Adjusted P= 6.6331E−160
Female: n=104047
Male: n=67670
Patients, %
0
2
4
6
8
10
12
14
Blood and lymphatic system disorders
Cardiac disorders
Congenital, familial, and genetic disorders
Ear and labyrinth disorders
Endocrine disorders
Eye disorders
Gastrointestinal disorders
General disorders and administration site conditions
Hepatobiliary disorders
Immune system disorders
Infections and infestations
Injury, poisoning, and procedural complications
Investigations
Metabolism and nutrition disorders
Musculoskeletal and connective tissue disorders
Neoplasms benign, malignant, and unspecified (incl cysts and polyps)
Nervous system disorders
Psychiatric disorders
Renal and urinary disorders
Reproductive system and breast disorders
Respiratory, thoracic, and mediastinal disorders
Skin and subcutaneous tissue disorders
Social circumstances
Surgical and medical procedures
Vascular disorders

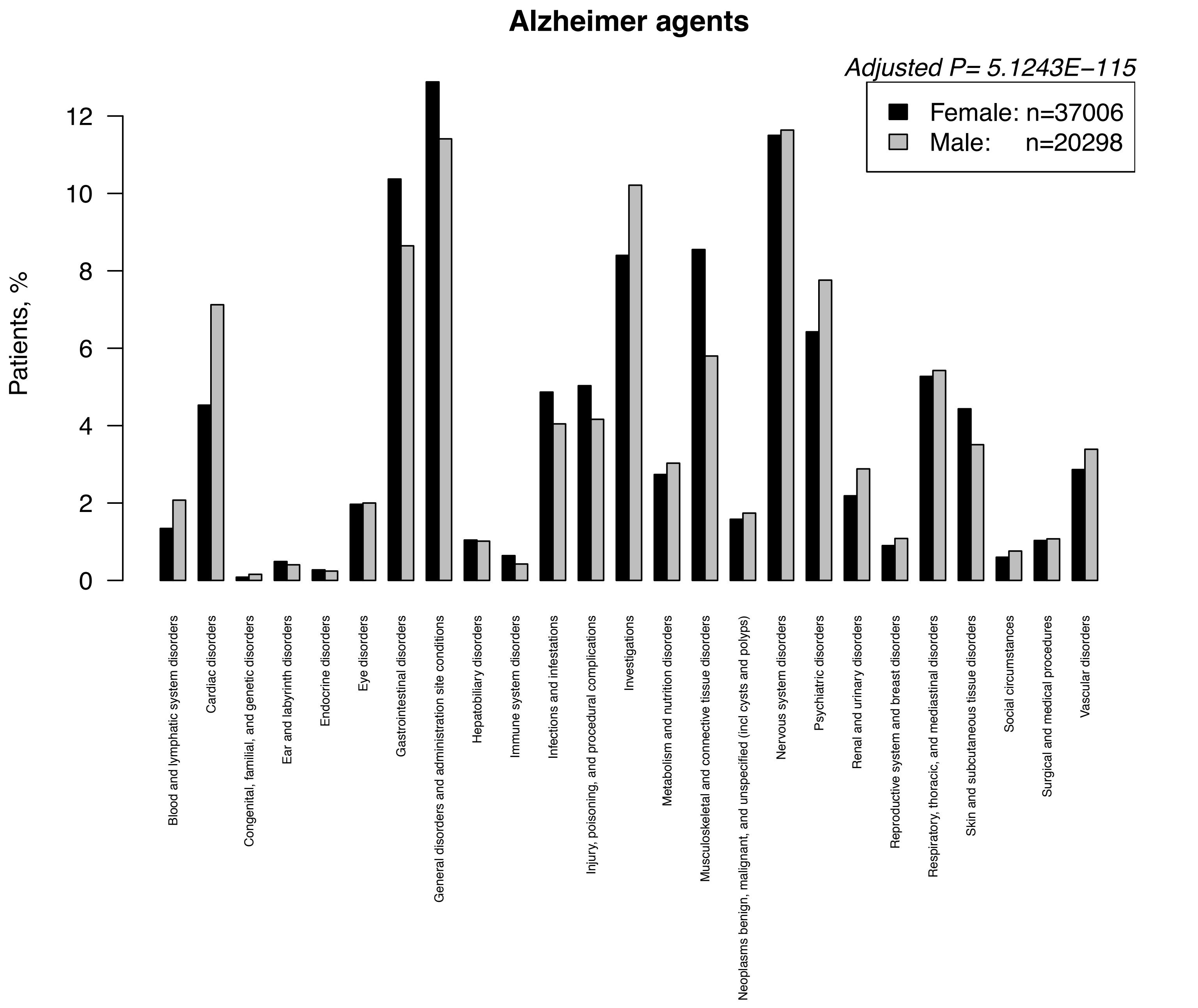

Alzheimer agents
Adjusted P= 5.1243E−115
Female: n=37006
Male: n=20298
Patients, %
0
2
4
6
8
10
12
Blood and lymphatic system disorders
Cardiac disorders
Congenital, familial, and genetic disorders
Ear and labyrinth disorders
Endocrine disorders
Eye disorders
Gastrointestinal disorders
General disorders and administration site conditions
Hepatobiliary disorders
Immune system disorders
Infections and infestations
Injury, poisoning, and procedural complications
Investigations
Metabolism and nutrition disorders
Musculoskeletal and connective tissue disorders
Neoplasms benign, malignant, and unspecified (incl cysts and polyps)
Nervous system disorders
Psychiatric disorders
Renal and urinary disorders
Reproductive system and breast disorders
Respiratory, thoracic, and mediastinal disorders
Skin and subcutaneous tissue disorders
Social circumstances
Surgical and medical procedures
Vascular disorders

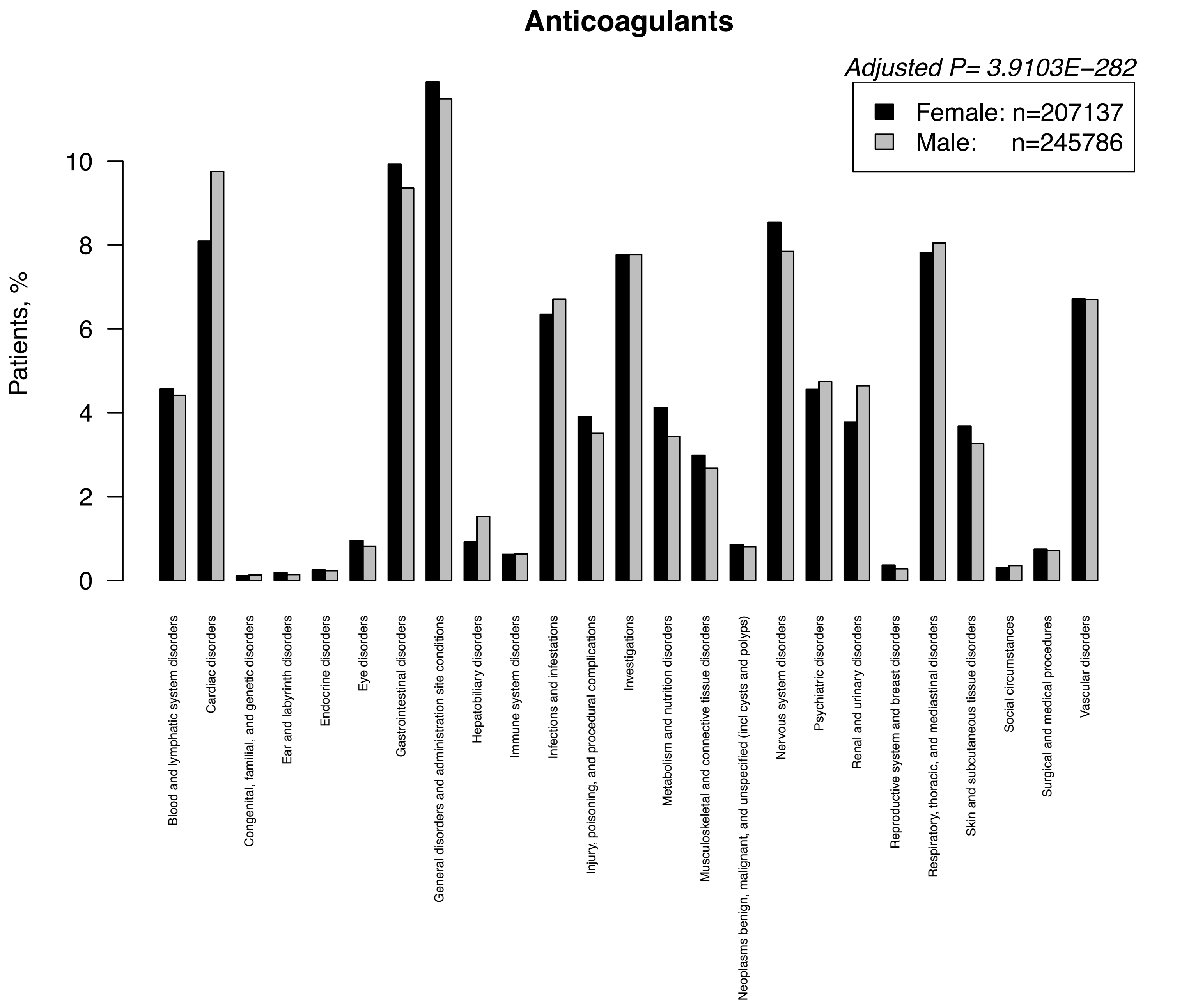
Anticoagulants
Adjusted P= 3.9103E−282
Female: n=207137
Male: n=245786
Patients, %
0
2
4
6
8
10
Blood and lymphatic system disorders
Cardiac disorders
Congenital, familial, and genetic disorders
Ear and labyrinth disorders
Endocrine disorders
Eye disorders
Gastrointestinal disorders
General disorders and administration site conditions
Hepatobiliary disorders
Immune system disorders
Infections and infestations
Injury, poisoning, and procedural complications
Investigations
Metabolism and nutrition disorders
Musculoskeletal and connective tissue disorders
Neoplasms benign, malignant, and unspecified (incl cysts and polyps)
Nervous system disorders
Psychiatric disorders
Renal and urinary disorders
Reproductive system and breast disorders
Respiratory, thoracic, and mediastinal disorders
Skin and subcutaneous tissue disorders
Social circumstances
Surgical and medical procedures
Vascular disorders

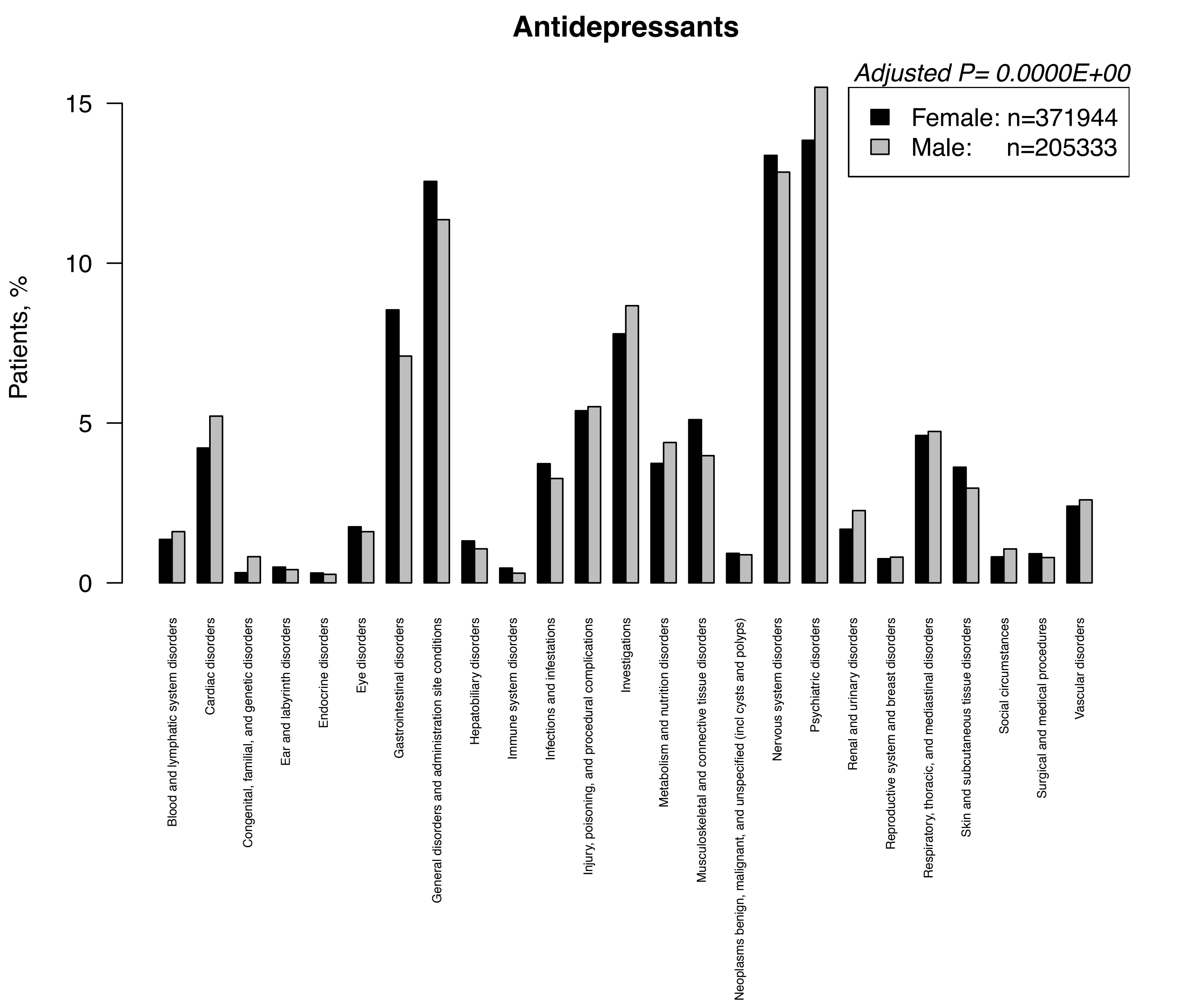
Antidepressants
Adjusted P= 0.0000E+00
Female: n=371944
Male: n=205333
Patients, %
0
5
10
15
Blood and lymphatic system disorders
Cardiac disorders
Congenital, familial, and genetic disorders
Ear and labyrinth disorders
Endocrine disorders
Eye disorders
Gastrointestinal disorders
General disorders and administration site conditions
Hepatobiliary disorders
Immune system disorders
Infections and infestations
Injury, poisoning, and procedural complications
Investigations
Metabolism and nutrition disorders
Musculoskeletal and connective tissue disorders
Neoplasms benign, malignant, and unspecified (incl cysts and polyps)
Nervous system disorders
Psychiatric disorders
Renal and urinary disorders
Reproductive system and breast disorders
Respiratory, thoracic, and mediastinal disorders
Skin and subcutaneous tissue disorders
Social circumstances
Surgical and medical procedures
Vascular disorders

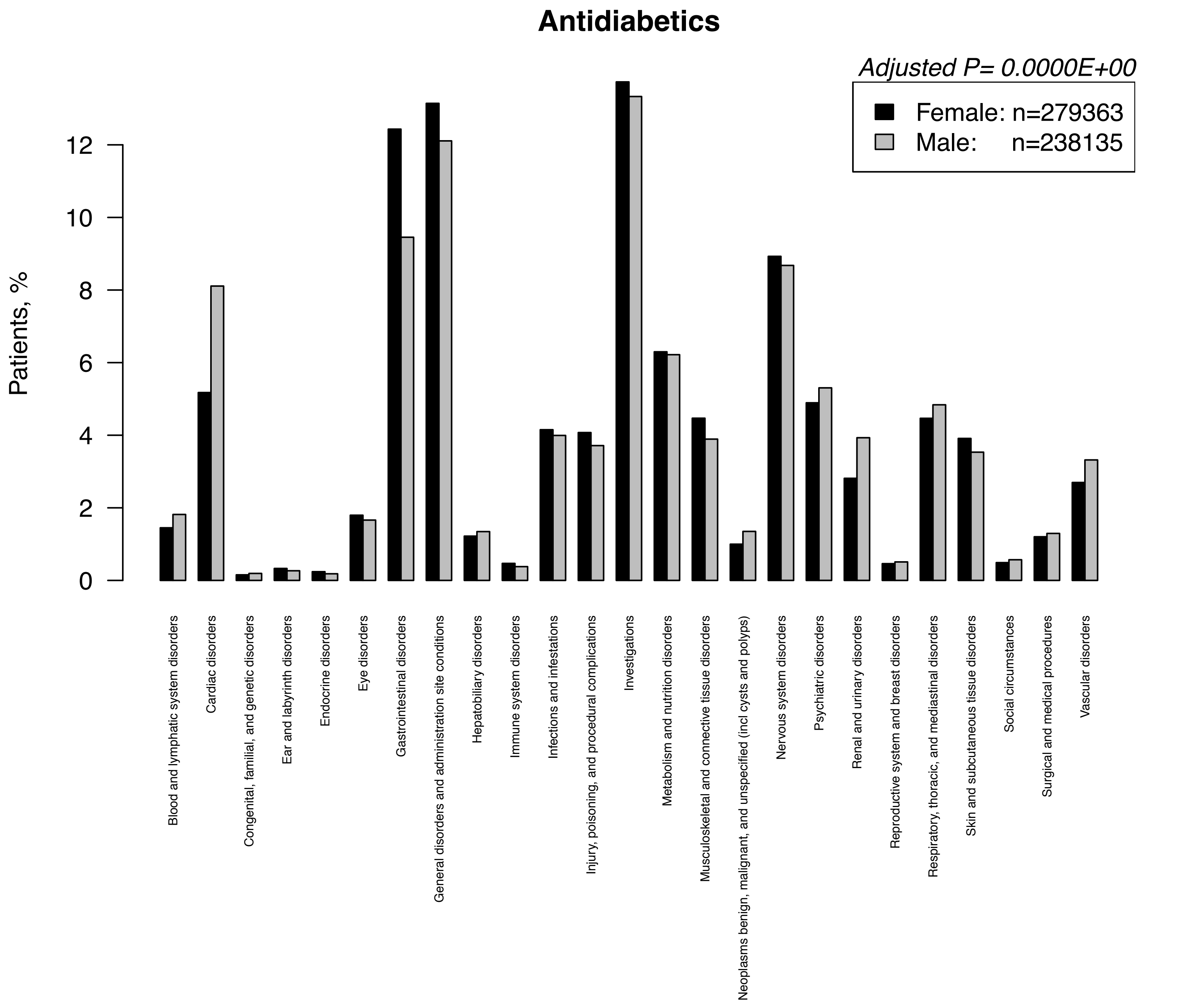

Antidiabetics
Adjusted P= 0.0000E+00
Female: n=279363
Male: n=238135
Patients, %
0
2
4
6
8
10
12
Blood and lymphatic system disorders
Cardiac disorders
Congenital, familial, and genetic disorders
Ear and labyrinth disorders
Endocrine disorders
Eye disorders
Gastrointestinal disorders
General disorders and administration site conditions
Hepatobiliary disorders
Immune system disorders
Infections and infestations
Injury, poisoning, and procedural complications
Investigations
Metabolism and nutrition disorders
Musculoskeletal and connective tissue disorders
Neoplasms benign, malignant, and unspecified (incl cysts and polyps)
Nervous system disorders
Psychiatric disorders
Renal and urinary disorders
Reproductive system and breast disorders
Respiratory, thoracic, and mediastinal disorders
Skin and subcutaneous tissue disorders
Social circumstances
Surgical and medical procedures
Vascular disorders

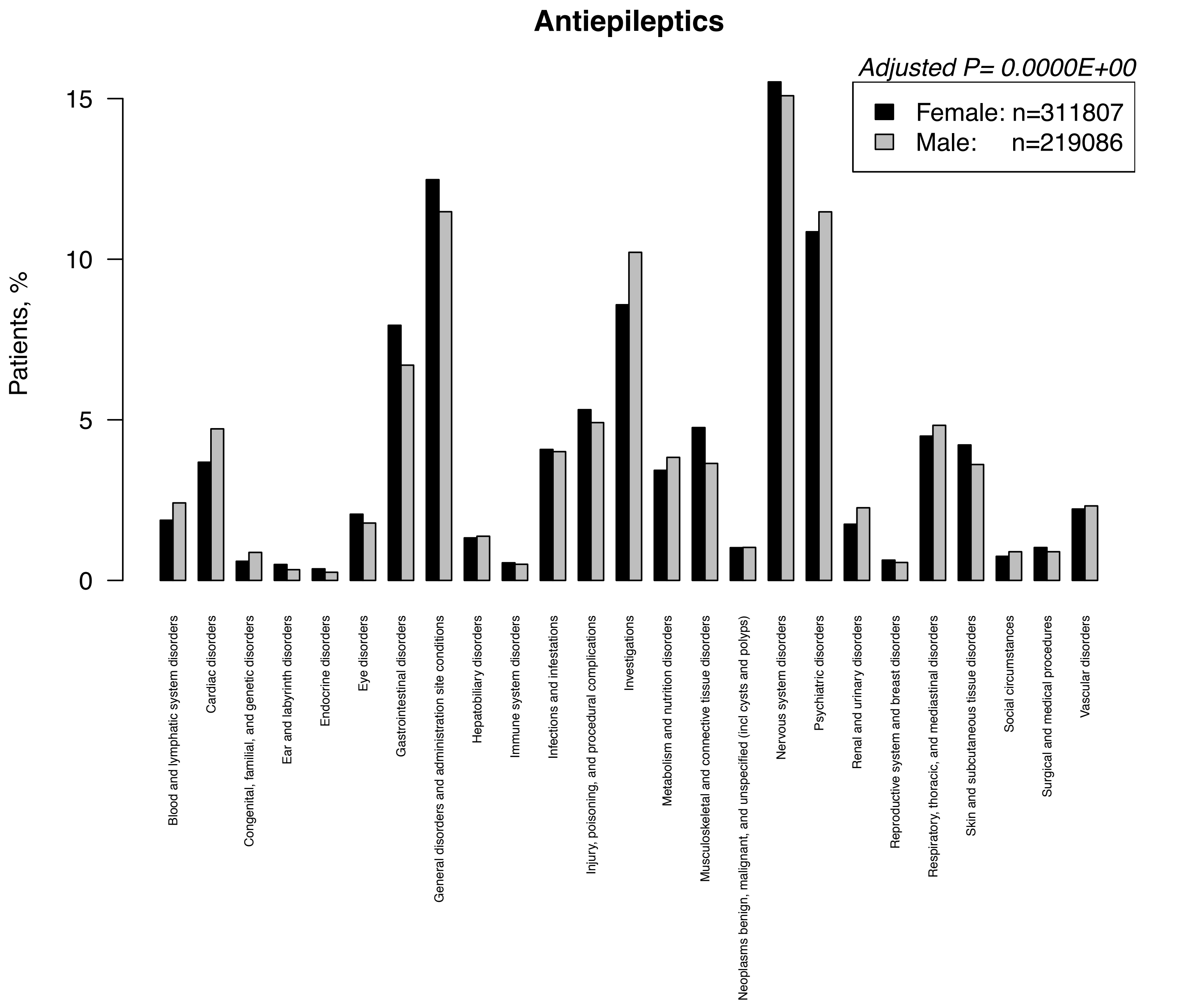

Antiepileptics
Adjusted P= 0.0000E+00
Female: n=311807
Male: n=219086
Patients, %
15
10
5
0
Blood and lymphatic system disorders
Cardiac disorders
Congenital, familial, and genetic disorders
Ear and labyrinth disorders
Endocrine disorders
Eye disorders
Gastrointestinal disorders
General disorders and administration site conditions
Hepatobiliary disorders
Immune system disorders
Infections and infestations
Injury, poisoning, and procedural complications
Investigations
Metabolism and nutrition disorders
Musculoskeletal and connective tissue disorders
Neoplasms benign, malignant, and unspecified (incl cysts and polyps)
Nervous system disorders
Psychiatric disorders
Renal and urinary disorders
Reproductive system and breast disorders
Respiratory, thoracic, and mediastinal disorders
Skin and subcutaneous tissue disorders
Social circumstances
Surgical and medical procedures
Vascular disorders

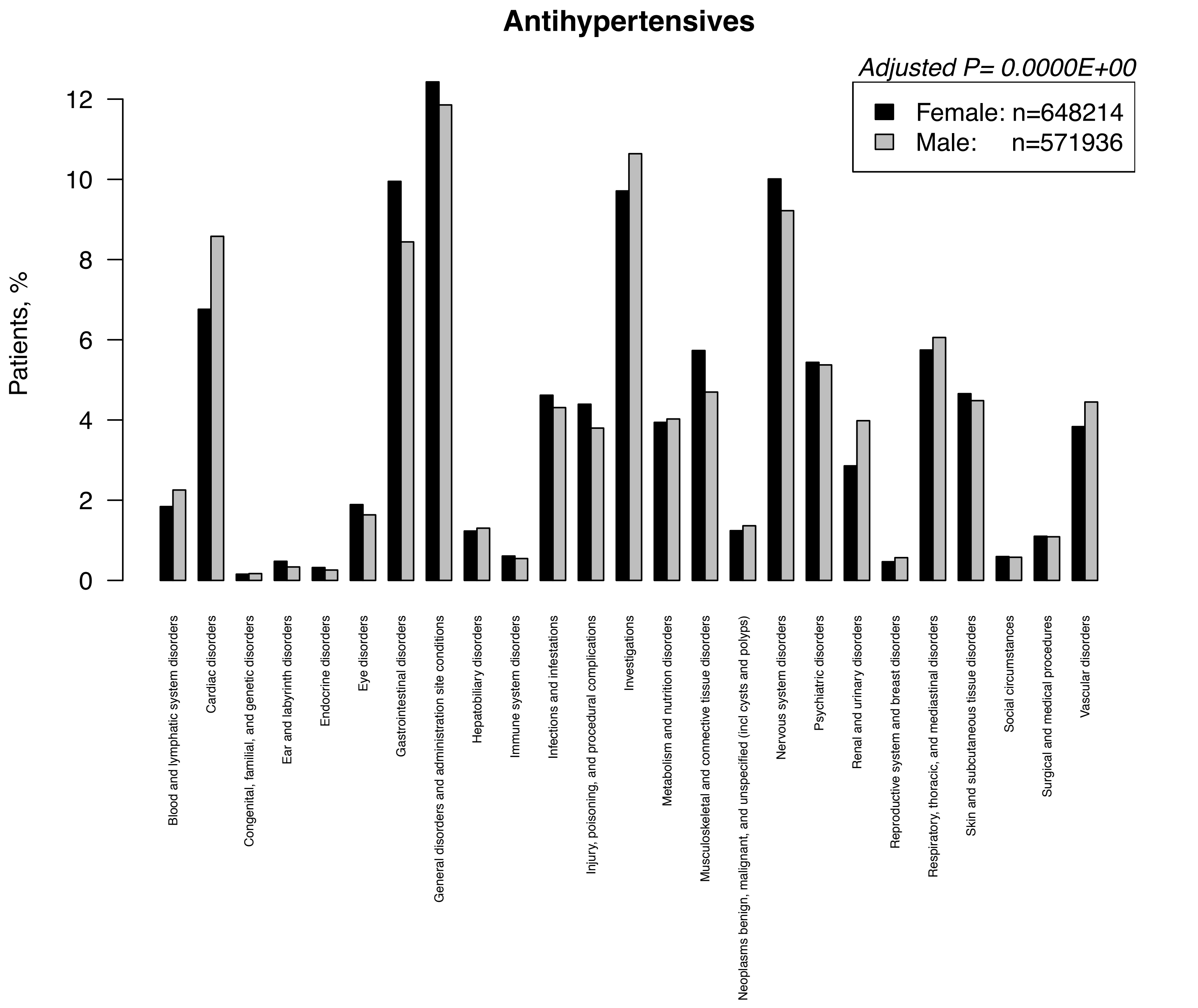

Antihypertensives
Adjusted P= 0.0000E+00
Female: n=648214
Male: n=571936
Patients, %
0
2
4
6
8
10
12
Blood and lymphatic system disorders
Cardiac disorders
Congenital, familial, and genetic disorders
Ear and labyrinth disorders
Endocrine disorders
Eye disorders
Gastrointestinal disorders
General disorders and administration site conditions
Hepatobiliary disorders
Immune system disorders
Infections and infestations
Injury, poisoning, and procedural complications
Investigations
Metabolism and nutrition disorders
Musculoskeletal and connective tissue disorders
Neoplasms benign, malignant, and unspecified (incl cysts and polyps)
Nervous system disorders
Psychiatric disorders
Renal and urinary disorders
Reproductive system and breast disorders
Respiratory, thoracic, and mediastinal disorders
Skin and subcutaneous tissue disorders
Social circumstances
Surgical and medical procedures
Vascular disorders

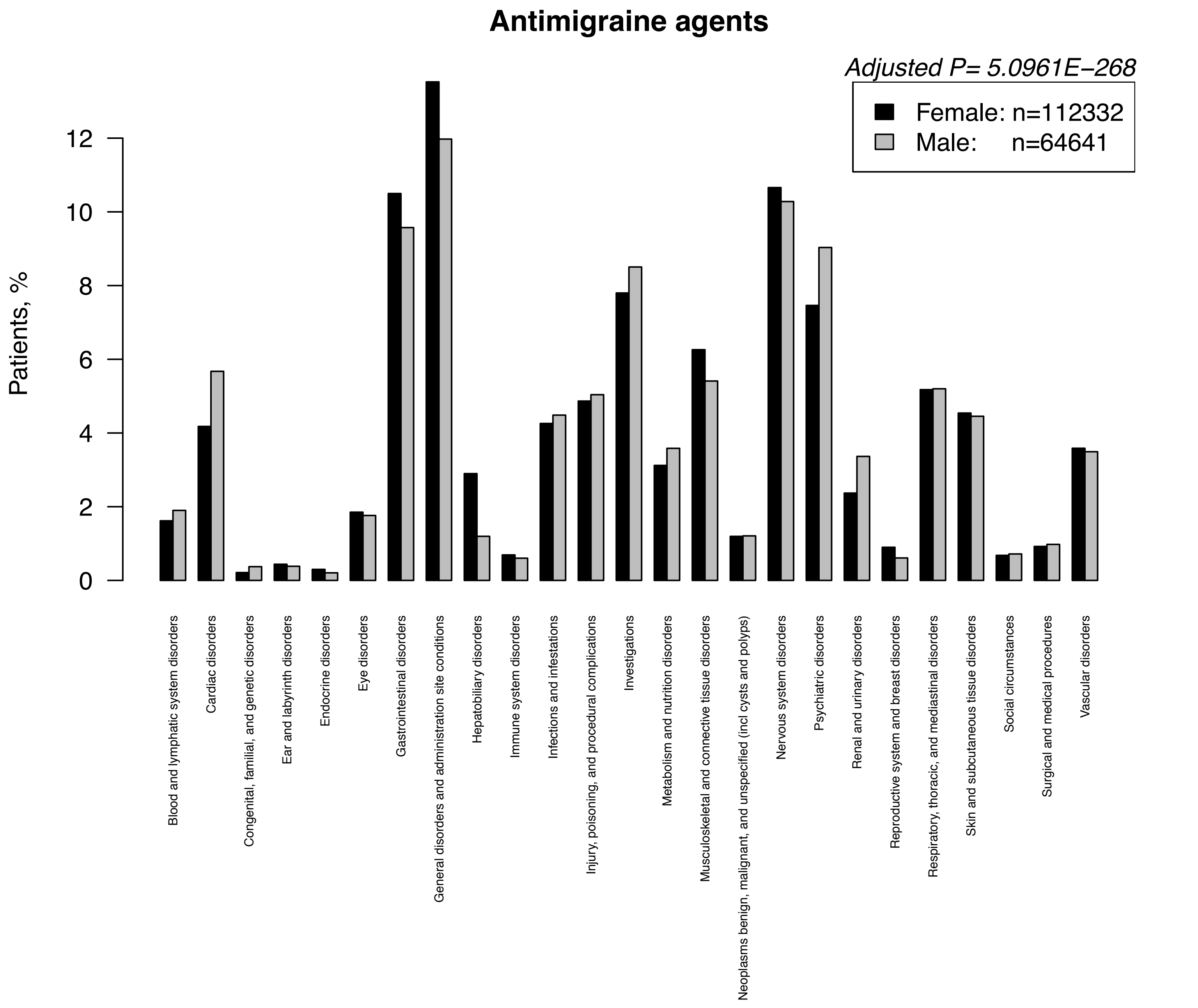

Antimigraine agents
Adjusted P= 5.0961E−268
Female: n=112332
Male: n=64641
Patients, %
0
2
4
6
8
10
12
Blood and lymphatic system disorders
Cardiac disorders
Congenital, familial, and genetic disorders
Ear and labyrinth disorders
Endocrine disorders
Eye disorders
Gastrointestinal disorders
General disorders and administration site conditions
Hepatobiliary disorders
Immune system disorders
Infections and infestations
Injury, poisoning, and procedural complications
Investigations
Metabolism and nutrition disorders
Musculoskeletal and connective tissue disorders
Neoplasms benign, malignant, and unspecified (incl cysts and polyps)
Nervous system disorders
Psychiatric disorders
Renal and urinary disorders
Reproductive system and breast disorders
Respiratory, thoracic, and mediastinal disorders
Skin and subcutaneous tissue disorders
Social circumstances
Surgical and medical procedures
Vascular disorders

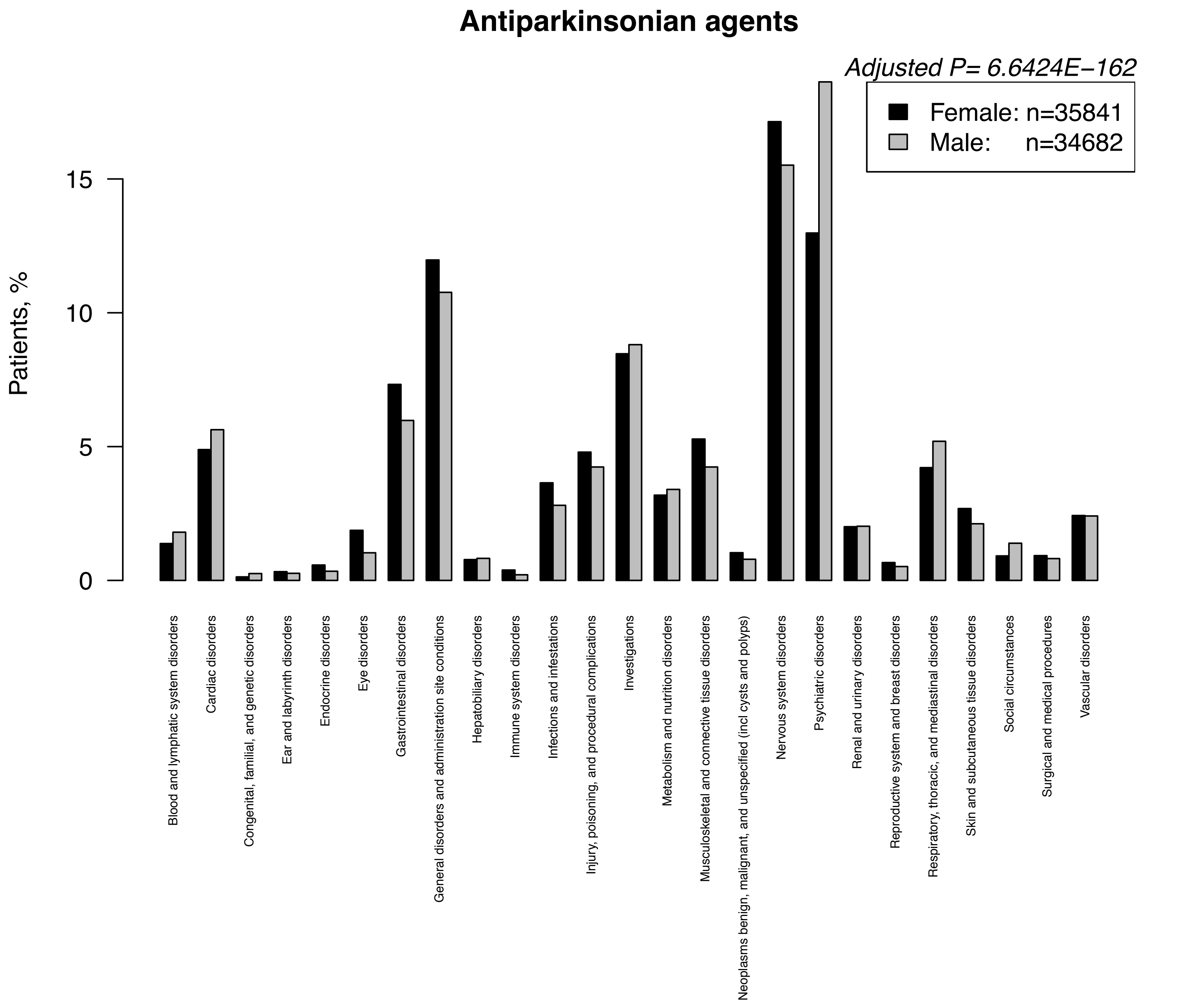
Antiparkinsonian agents
Adjusted P= 6.6424E−162
Female: n=35841
Male: n=34682
Patients, %
0
5
10
15
Blood and lymphatic system disorders
Cardiac disorders
Congenital, familial, and genetic disorders
Ear and labyrinth disorders
Endocrine disorders
Eye disorders
Gastrointestinal disorders
General disorders and administration site conditions
Hepatobiliary disorders
Immune system disorders
Infections and infestations
Injury, poisoning, and procedural complications
Investigations
Metabolism and nutrition disorders
Musculoskeletal and connective tissue disorders
Neoplasms benign, malignant, and unspecified (incl cysts and polyps)
Nervous system disorders
Psychiatric disorders
Renal and urinary disorders
Reproductive system and breast disorders
Respiratory, thoracic, and mediastinal disorders
Skin and subcutaneous tissue disorders
Social circumstances
Surgical and medical procedures
Vascular disorders

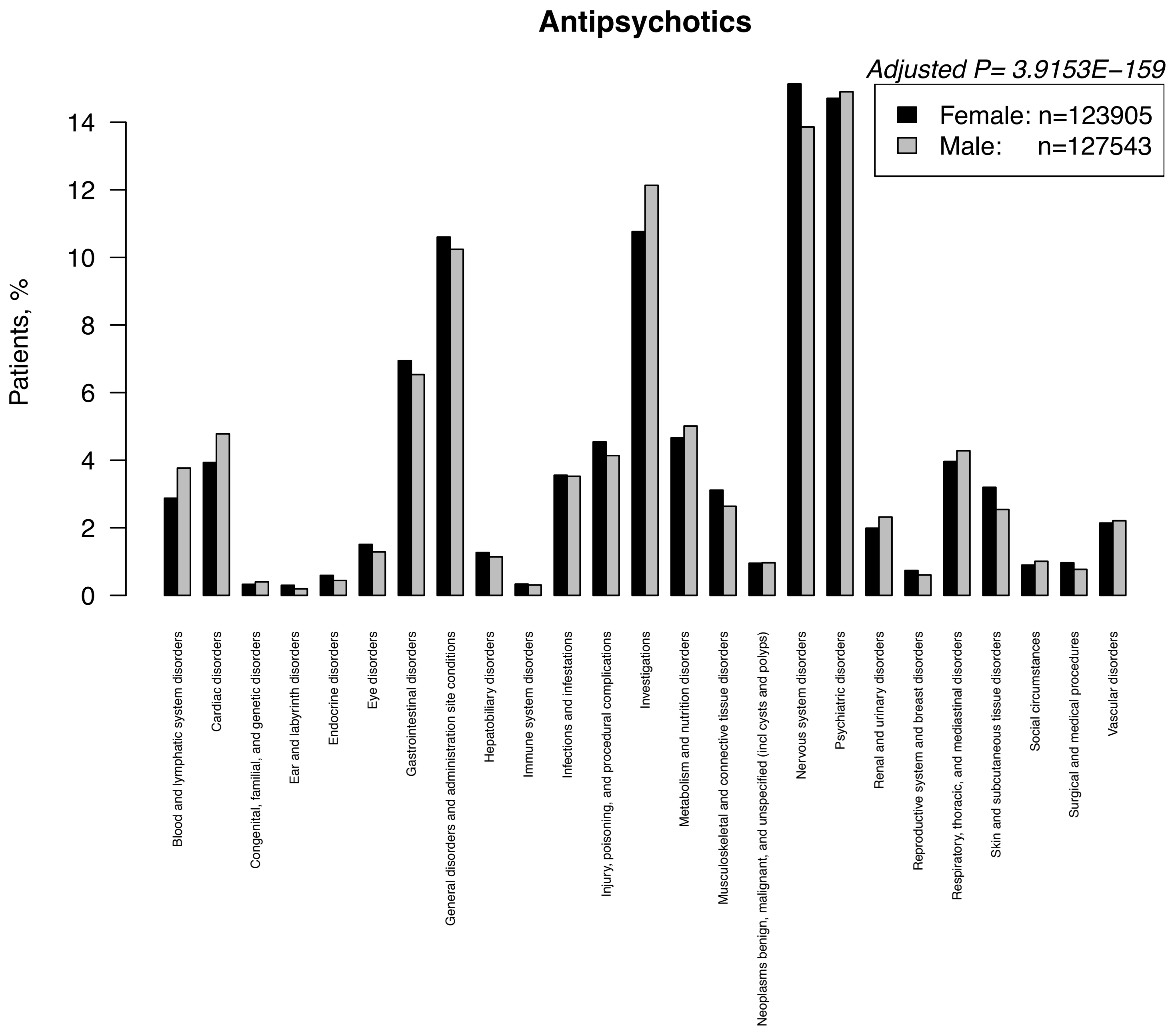
Antipsychotics
Adjusted P= 3.9153E−159
Female: n=123905
Male: n=127543
Patients, %
0
2
4
6
8
10
12
14
Blood and lymphatic system disorders
Cardiac disorders
Congenital, familial, and genetic disorders
Ear and labyrinth disorders
Endocrine disorders
Eye disorders
Gastrointestinal disorders
General disorders and administration site conditions
Hepatobiliary disorders
Immune system disorders
Infections and infestations
Injury, poisoning, and procedural complications
Investigations
Metabolism and nutrition disorders
Musculoskeletal and connective tissue disorders
Neoplasms benign, malignant, and unspecified (incl cysts and polyps)
Nervous system disorders
Psychiatric disorders
Renal and urinary disorders
Reproductive system and breast disorders
Respiratory, thoracic, and mediastinal disorders
Skin and subcutaneous tissue disorders
Social circumstances
Surgical and medical procedures
Vascular disorders

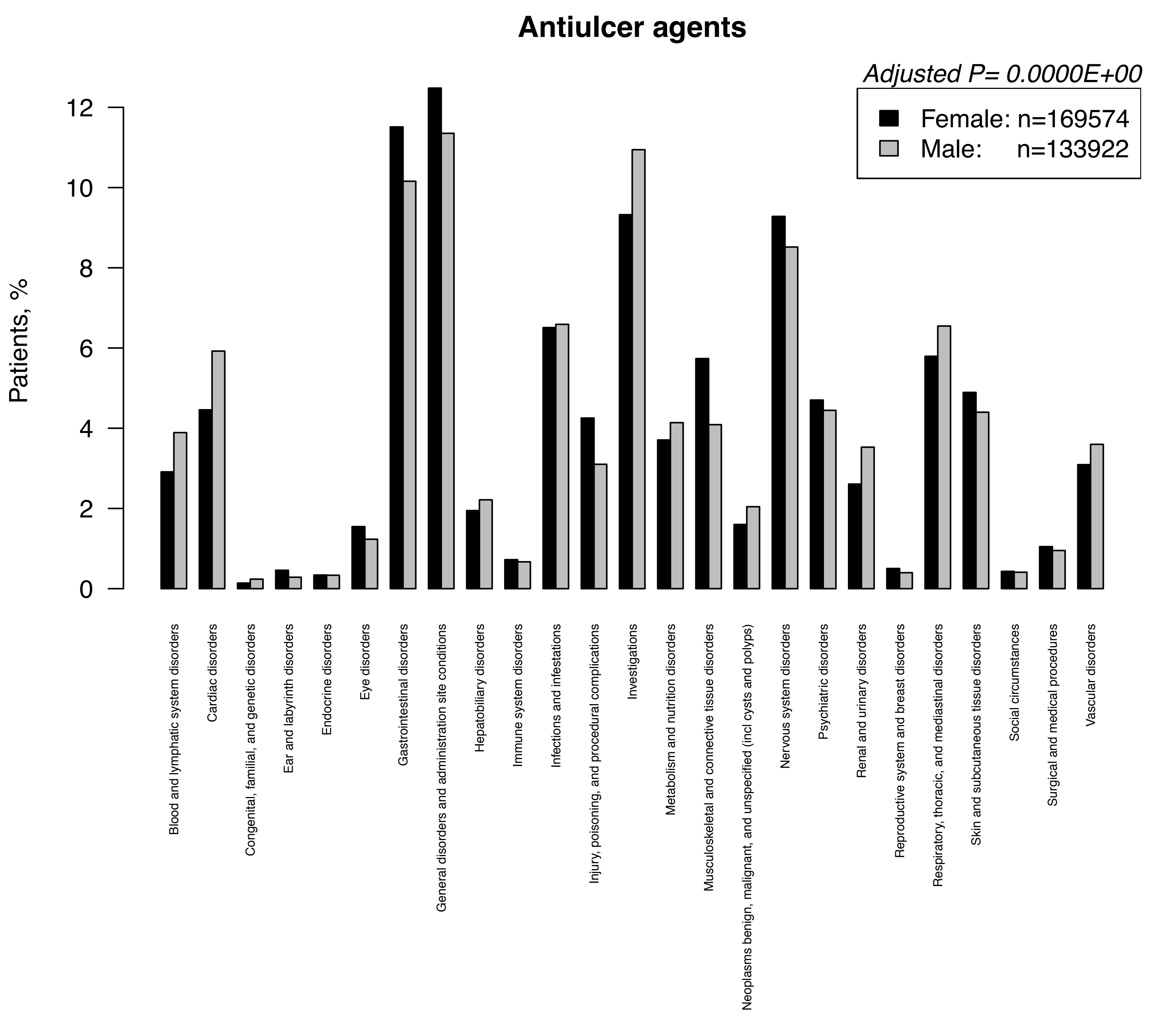
Antiulcer agents
Adjusted P= 0.0000E+00
Female: n=169574
Male: n=133922
Patients, %
0
2
4
6
8
10
12
Blood and lymphatic system disorders
Cardiac disorders
Congenital, familial, and genetic disorders
Ear and labyrinth disorders
Endocrine disorders
Eye disorders
Gastrointestinal disorders
General disorders and administration site conditions
Hepatobiliary disorders
Immune system disorders
Infections and infestations
Injury, poisoning, and procedural complications
Investigations
Metabolism and nutrition disorders
Musculoskeletal and connective tissue disorders
Neoplasms benign, malignant, and unspecified (incl cysts and polyps)
Nervous system disorders
Psychiatric disorders
Renal and urinary disorders
Reproductive system and breast disorders
Respiratory, thoracic, and mediastinal disorders
Skin and subcutaneous tissue disorders
Social circumstances
Surgical and medical procedures
Vascular disorders

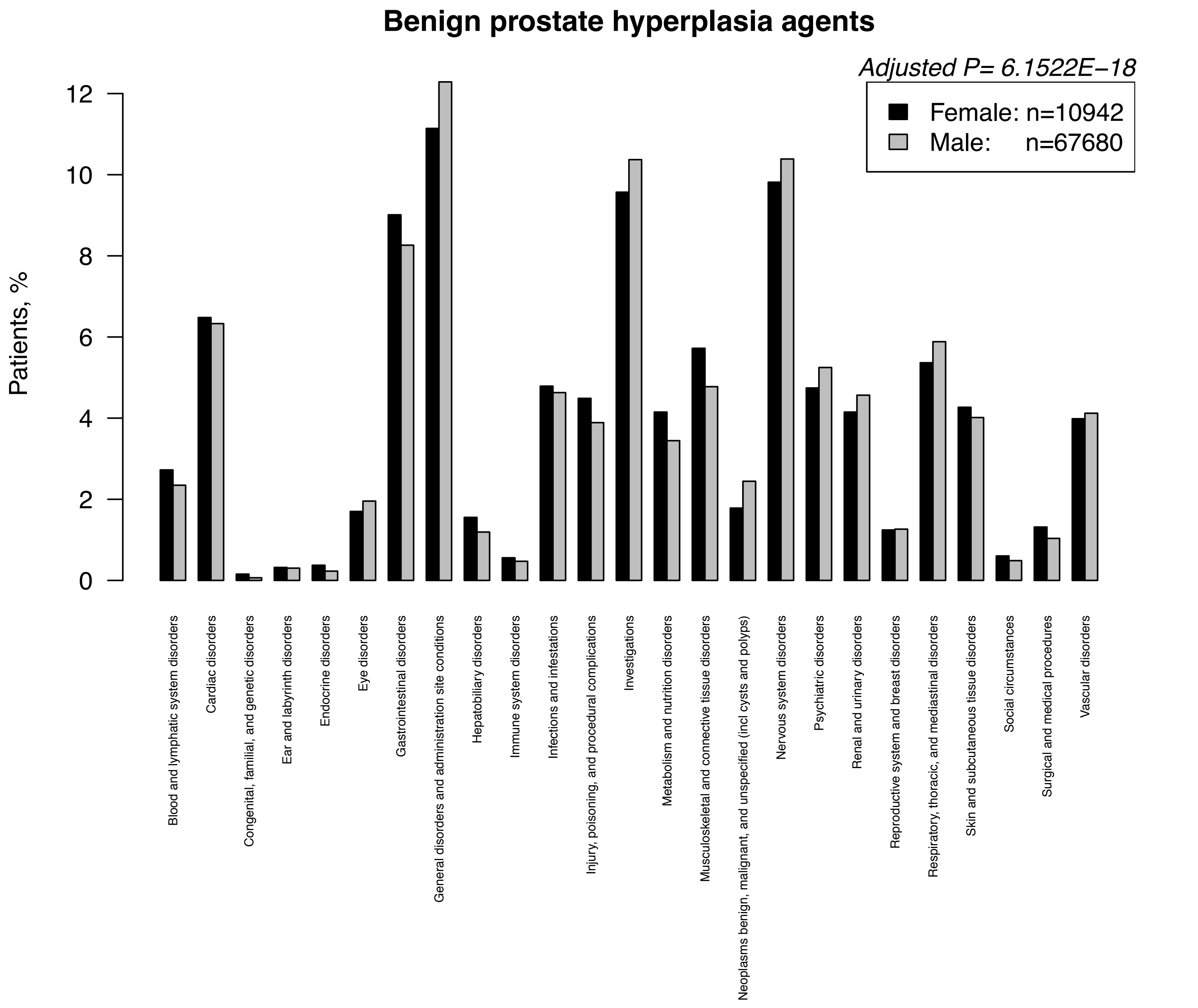
Benign prostate hyperplasia agents
Adjusted P= 6.1522E−18
Female: n=10942
Male: n=67680
Patients, %
0
2
4
6
8
10
12
Blood and lymphatic system disorders
Cardiac disorders
Congenital, familial, and genetic disorders
Ear and labyrinth disorders
Endocrine disorders
Eye disorders
Gastrointestinal disorders
General disorders and administration site conditions
Hepatobiliary disorders
Immune system disorders
Infections and infestations
Injury, poisoning, and procedural complications
Investigations
Metabolism and nutrition disorders
Musculoskeletal and connective tissue disorders
Neoplasms benign, malignant, and unspecified (incl cysts and polyps)
Nervous system disorders
Psychiatric disorders
Renal and urinary disorders
Reproductive system and breast disorders
Respiratory, thoracic, and mediastinal disorders
Skin and subcutaneous tissue disorders
Social circumstances
Surgical and medical procedures
Vascular disorders

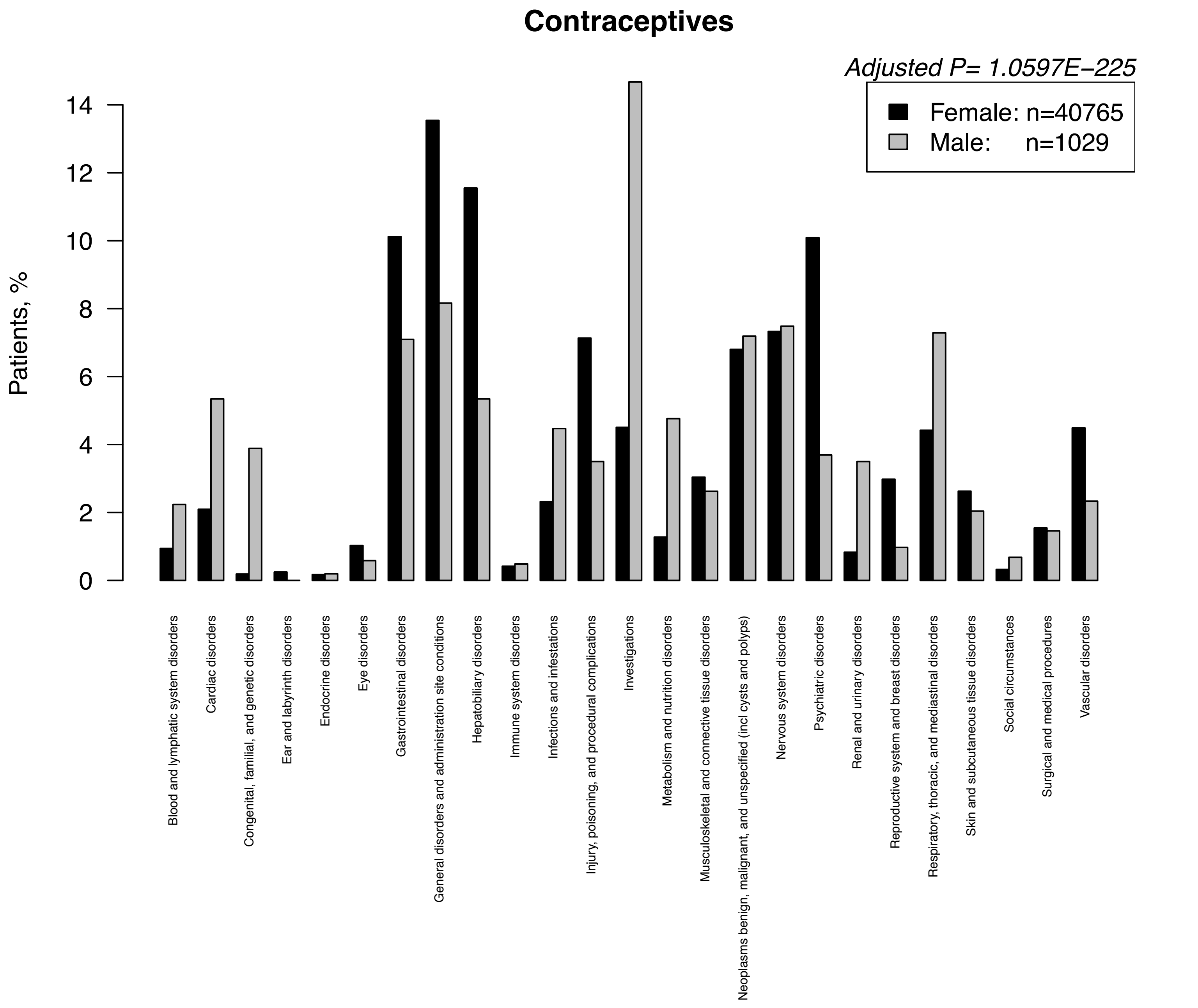
Contraceptives
Adjusted P= 1.0597E−225
Female: n=40765
Male: n=1029
Patients, %
0
2
4
6
8
10
12
14
Blood and lymphatic system disorders
Cardiac disorders
Congenital, familial, and genetic disorders
Ear and labyrinth disorders
Endocrine disorders
Eye disorders
Gastrointestinal disorders
General disorders and administration site conditions
Hepatobiliary disorders
Immune system disorders
Infections and infestations
Injury, poisoning, and procedural complications
Investigations
Metabolism and nutrition disorders
Musculoskeletal and connective tissue disorders
Neoplasms benign, malignant, and unspecified (incl cysts and polyps)
Nervous system disorders
Psychiatric disorders
Renal and urinary disorders
Reproductive system and breast disorders
Respiratory, thoracic, and mediastinal disorders
Skin and subcutaneous tissue disorders
Social circumstances
Surgical and medical procedures
Vascular disorders

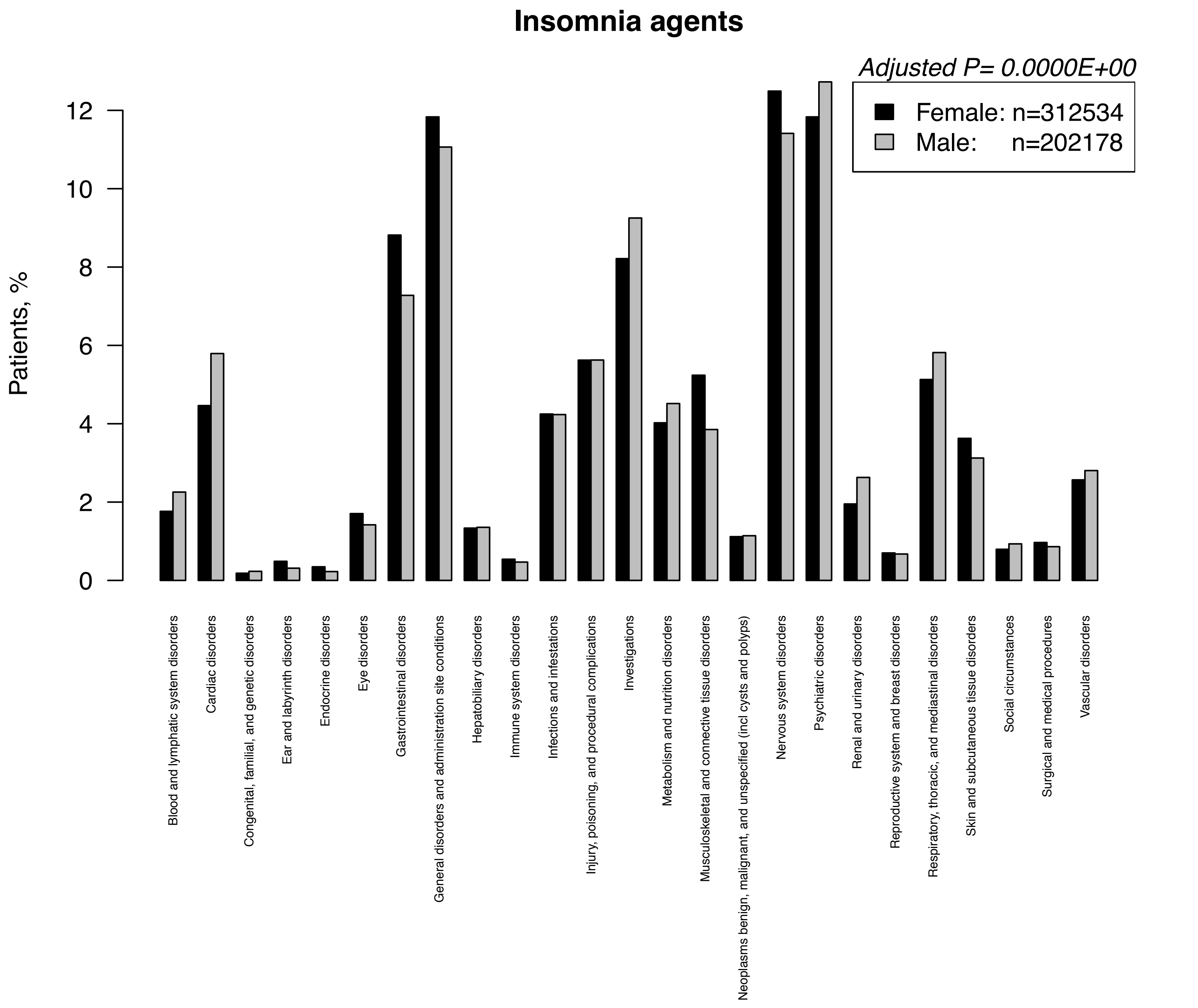
Insomnia agents
Adjusted P= 0.0000E+00
Female: n=312534
Male: n=202178
Patients, %
0
2
4
6
8
10
12
Blood and lymphatic system disorders
Cardiac disorders
Congenital, familial, and genetic disorders
Ear and labyrinth disorders
Endocrine disorders
Eye disorders
Gastrointestinal disorders
General disorders and administration site conditions
Hepatobiliary disorders
Immune system disorders
Infections and infestations
Injury, poisoning, and procedural complications
Investigations
Metabolism and nutrition disorders
Musculoskeletal and connective tissue disorders
Neoplasms benign, malignant, and unspecified (incl cysts and polyps)
Nervous system disorders
Psychiatric disorders
Renal and urinary disorders
Reproductive system and breast disorders
Respiratory, thoracic, and mediastinal disorders
Skin and subcutaneous tissue disorders
Social circumstances
Surgical and medical procedures
Vascular disorders

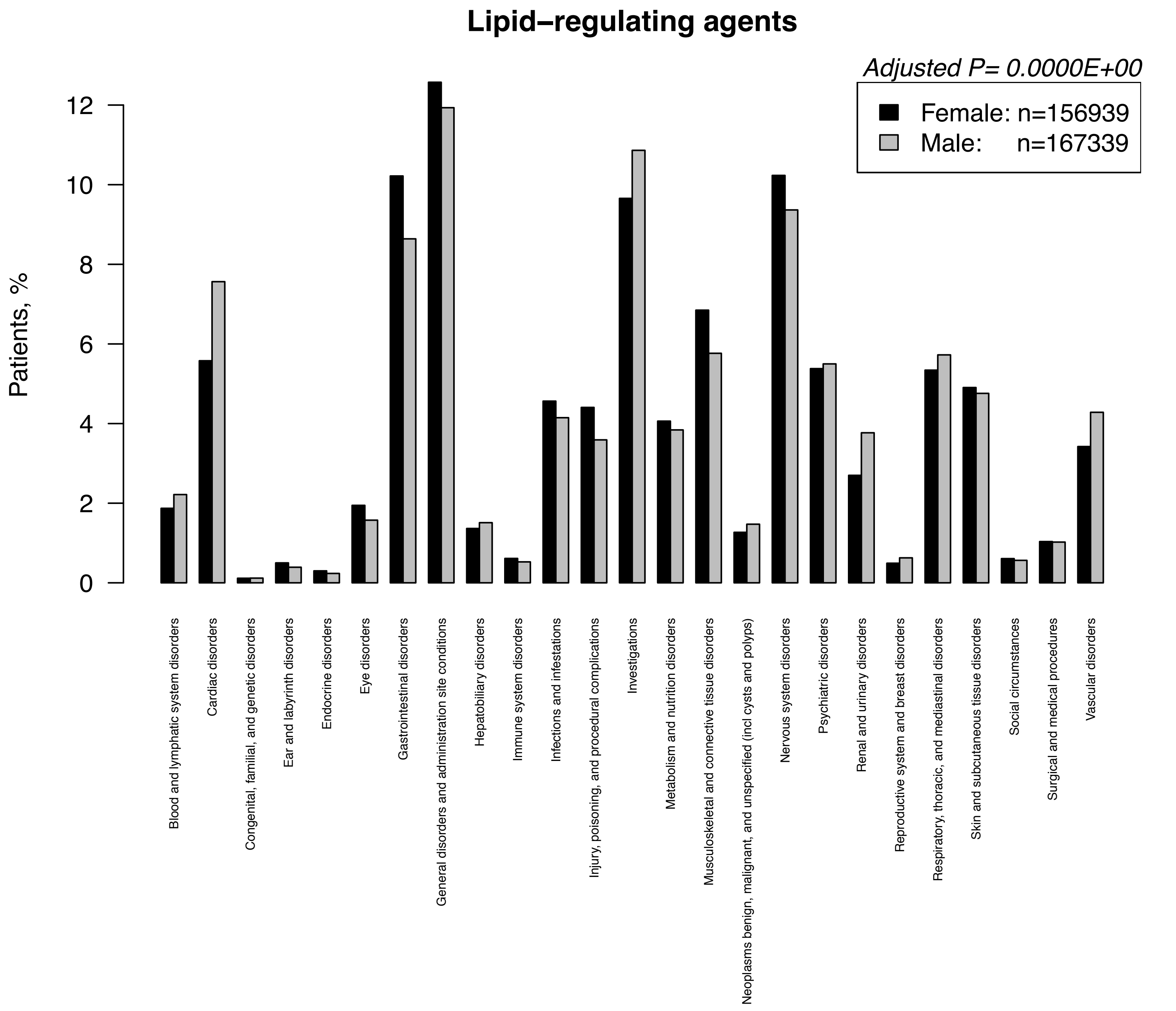
Lipid−regulating agents
Adjusted P= 0.0000E+00
Female: n=156939
Male: n=167339
Patients, %
0
2
4
6
8
10
12
Blood and lymphatic system disorders
Cardiac disorders
Congenital, familial, and genetic disorders
Ear and labyrinth disorders
Endocrine disorders
Eye disorders
Gastrointestinal disorders
General disorders and administration site conditions
Hepatobiliary disorders
Immune system disorders
Infections and infestations
Injury, poisoning, and procedural complications
Investigations
Metabolism and nutrition disorders
Musculoskeletal and connective tissue disorders
Neoplasms benign, malignant, and unspecified (incl cysts and polyps)
Nervous system disorders
Psychiatric disorders
Renal and urinary disorders
Reproductive system and breast disorders
Respiratory, thoracic, and mediastinal disorders
Skin and subcutaneous tissue disorders
Social circumstances
Surgical and medical procedures
Vascular disorders

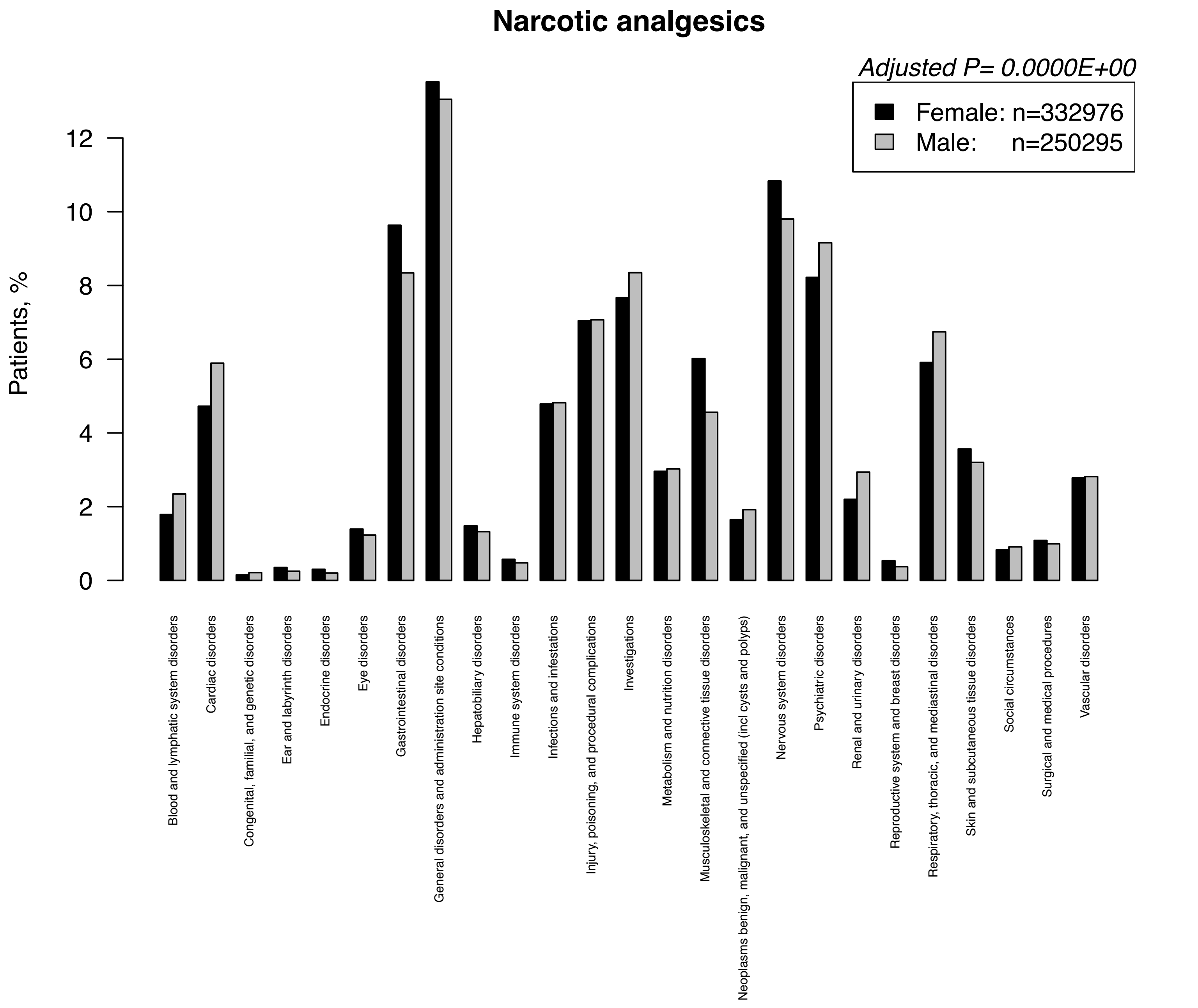
Narcotic analgesics
Adjusted P= 0.0000E+00
Female: n=332976
Male: n=250295
Patients, %
0
2
4
6
8
10
12
Blood and lymphatic system disorders
Cardiac disorders
Congenital, familial, and genetic disorders
Ear and labyrinth disorders
Endocrine disorders
Eye disorders
Gastrointestinal disorders
General disorders and administration site conditions
Hepatobiliary disorders
Immune system disorders
Infections and infestations
Injury, poisoning, and procedural complications
Investigations
Metabolism and nutrition disorders
Musculoskeletal and connective tissue disorders
Neoplasms benign, malignant, and unspecified (incl cysts and polyps)
Nervous system disorders
Psychiatric disorders
Renal and urinary disorders
Reproductive system and breast disorders
Respiratory, thoracic, and mediastinal disorders
Skin and subcutaneous tissue disorders
Social circumstances
Surgical and medical procedures
Vascular disorders

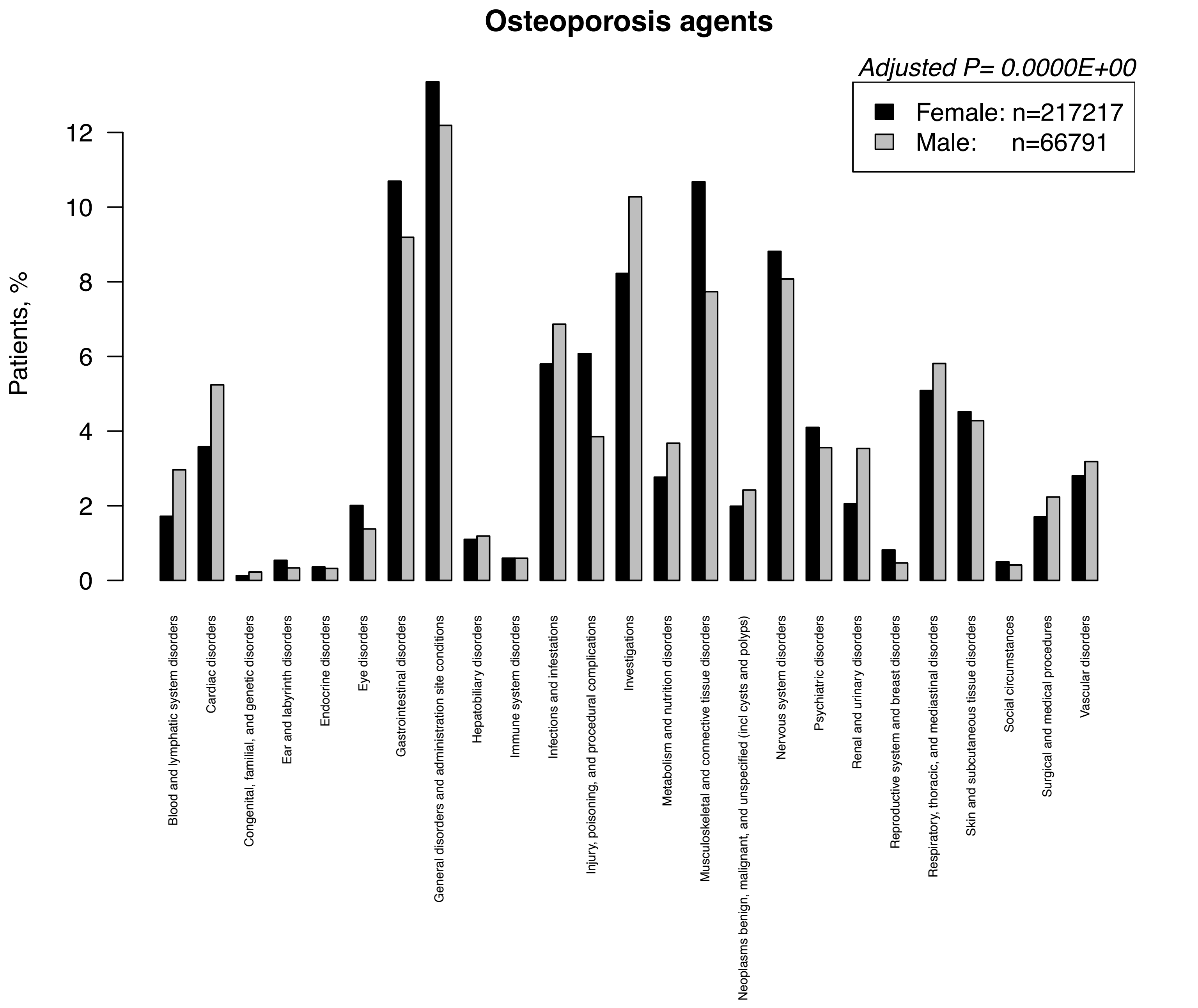
Osteoporosis agents
Adjusted P= 0.0000E+00
Female: n=217217
Male: n=66791
Patients, %
0
2
4
6
8
10
12
Blood and lymphatic system disorders
Cardiac disorders
Congenital, familial, and genetic disorders
Ear and labyrinth disorders
Endocrine disorders
Eye disorders
Gastrointestinal disorders
General disorders and administration site conditions
Hepatobiliary disorders
Immune system disorders
Infections and infestations
Injury, poisoning, and procedural complications
Investigations
Metabolism and nutrition disorders
Musculoskeletal and connective tissue disorders
Neoplasms benign, malignant, and unspecified (incl cysts and polyps)
Nervous system disorders
Psychiatric disorders
Renal and urinary disorders
Reproductive system and breast disorders
Respiratory, thoracic, and mediastinal disorders
Skin and subcutaneous tissue disorders
Social circumstances
Surgical and medical procedures
Vascular disorders

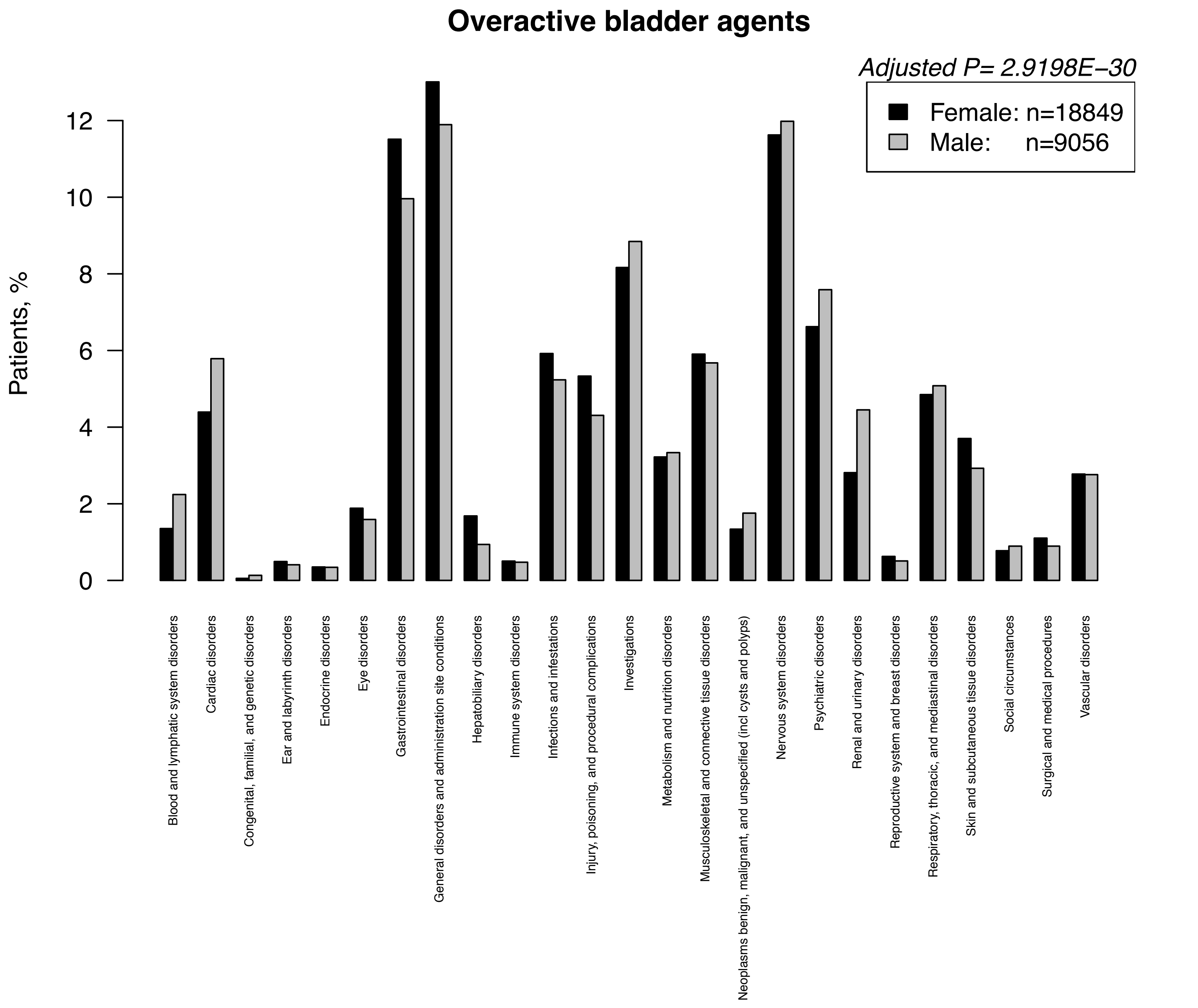
Overactive bladder agents
Adjusted P= 2.9198E−30
Female: n=18849
Male: n=9056
Patients, %
0
2
4
6
8
10
12
Blood and lymphatic system disorders
Cardiac disorders
Congenital, familial, and genetic disorders
Ear and labyrinth disorders
Endocrine disorders
Eye disorders
Gastrointestinal disorders
General disorders and administration site conditions
Hepatobiliary disorders
Immune system disorders
Infections and infestations
Injury, poisoning, and procedural complications
Investigations
Metabolism and nutrition disorders
Musculoskeletal and connective tissue disorders
Neoplasms benign, malignant, and unspecified (incl cysts and polyps)
Nervous system disorders
Psychiatric disorders
Renal and urinary disorders
Reproductive system and breast disorders
Respiratory, thoracic, and mediastinal disorders
Skin and subcutaneous tissue disorders
Social circumstances
Surgical and medical procedures
Vascular disorders

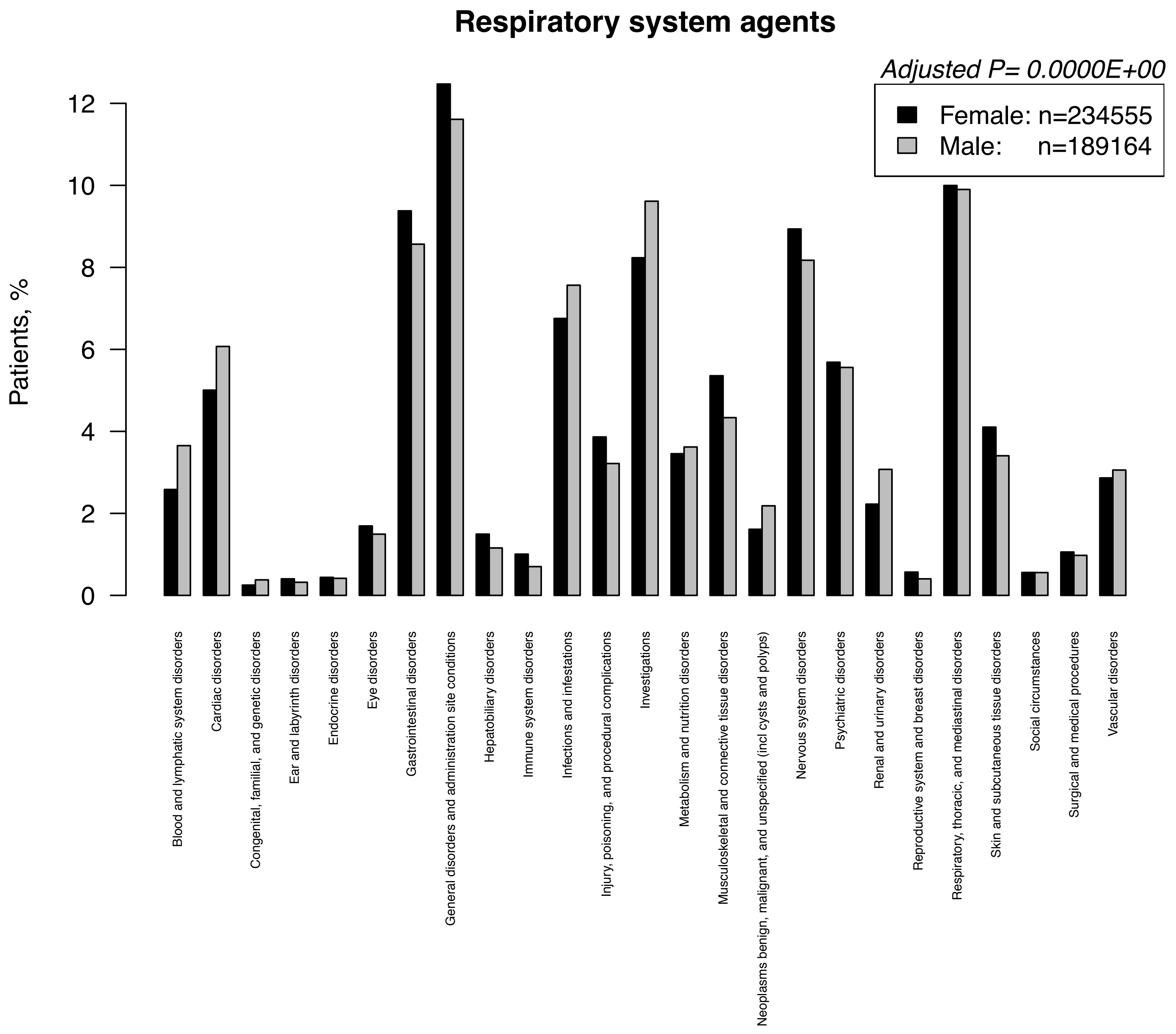

Respiratory system agents
Adjusted P= 0.0000E+00
Female: n=234555
Male: n=189164
Patients, %
0
2
4
6
8
10
12
Blood and lymphatic system disorders
Cardiac disorders
Congenital, familial, and genetic disorders
Ear and labyrinth disorders
Endocrine disorders
Eye disorders
Gastrointestinal disorders
General disorders and administration site conditions
Hepatobiliary disorders
Immune system disorders
Infections and infestations
Injury, poisoning, and procedural complications
Investigations
Metabolism and nutrition disorders
Musculoskeletal and connective tissue disorders
Neoplasms benign, malignant, and unspecified (incl cysts and polyps)
Nervous system disorders
Psychiatric disorders
Renal and urinary disorders
Reproductive system and breast disorders
Respiratory, thoracic, and mediastinal disorders
Skin and subcutaneous tissue disorders
Social circumstances
Surgical and medical procedures
Vascular disorders

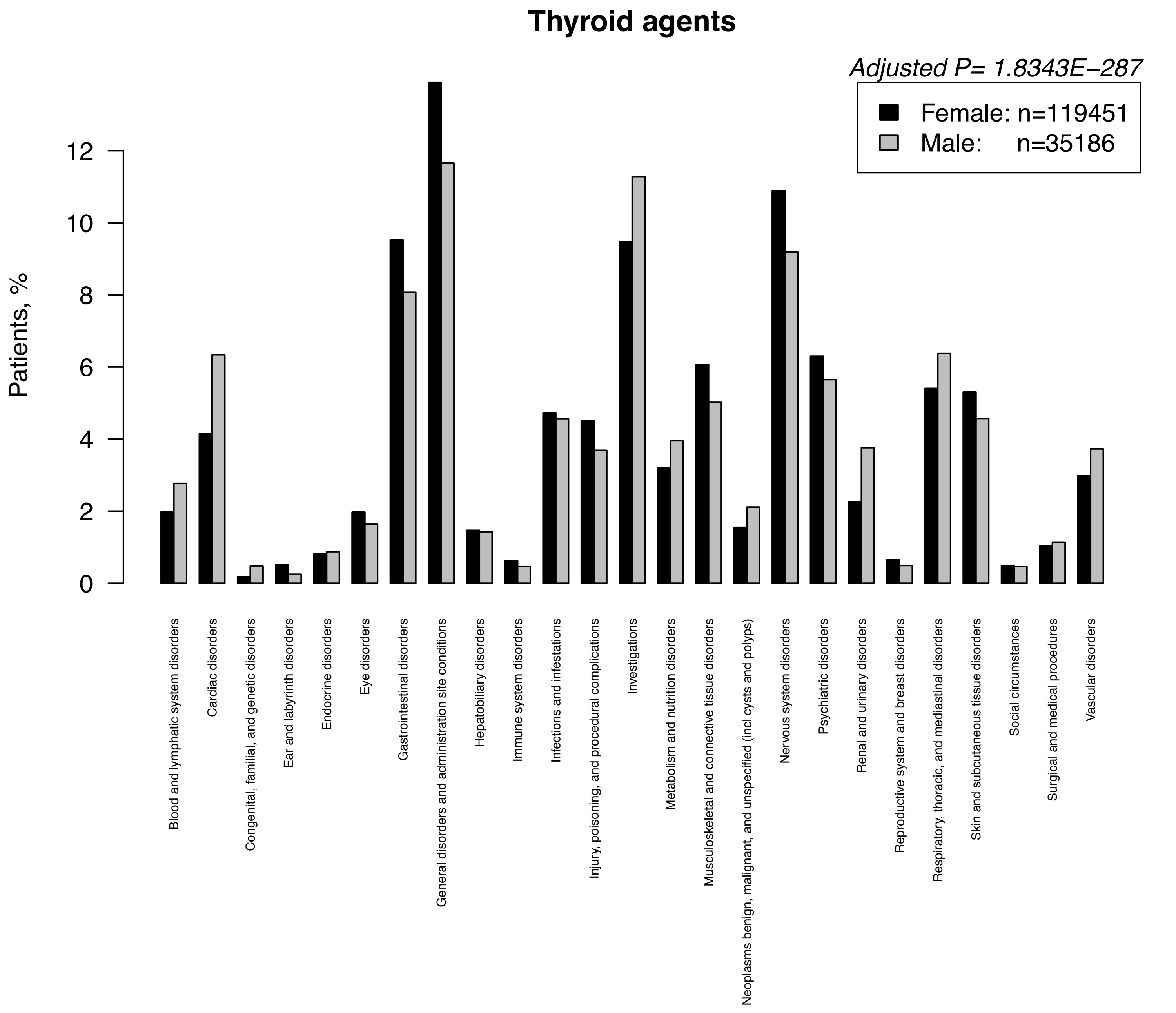
Thyroid agents
Adjusted P= 1.8343E−287
Female: n=119451
Male: n=35186
Patients, %
0
2
4
6
8
10
12
Blood and lymphatic system disorders
Cardiac disorders
Congenital, familial, and genetic disorders
Ear and labyrinth disorders
Endocrine disorders
Eye disorders
Gastrointestinal disorders
General disorders and administration site conditions
Hepatobiliary disorders
Immune system disorders
Infections and infestations
Injury, poisoning, and procedural complications
Investigations
Metabolism and nutrition disorders
Musculoskeletal and connective tissue disorders
Neoplasms benign, malignant, and unspecified (incl cysts and polyps)
Nervous system disorders
Psychiatric disorders
Renal and urinary disorders
Reproductive system and breast disorders
Respiratory, thoracic, and mediastinal disorders
Skin and subcutaneous tissue disorders
Social circumstances
Surgical and medical procedures
Vascular disorders

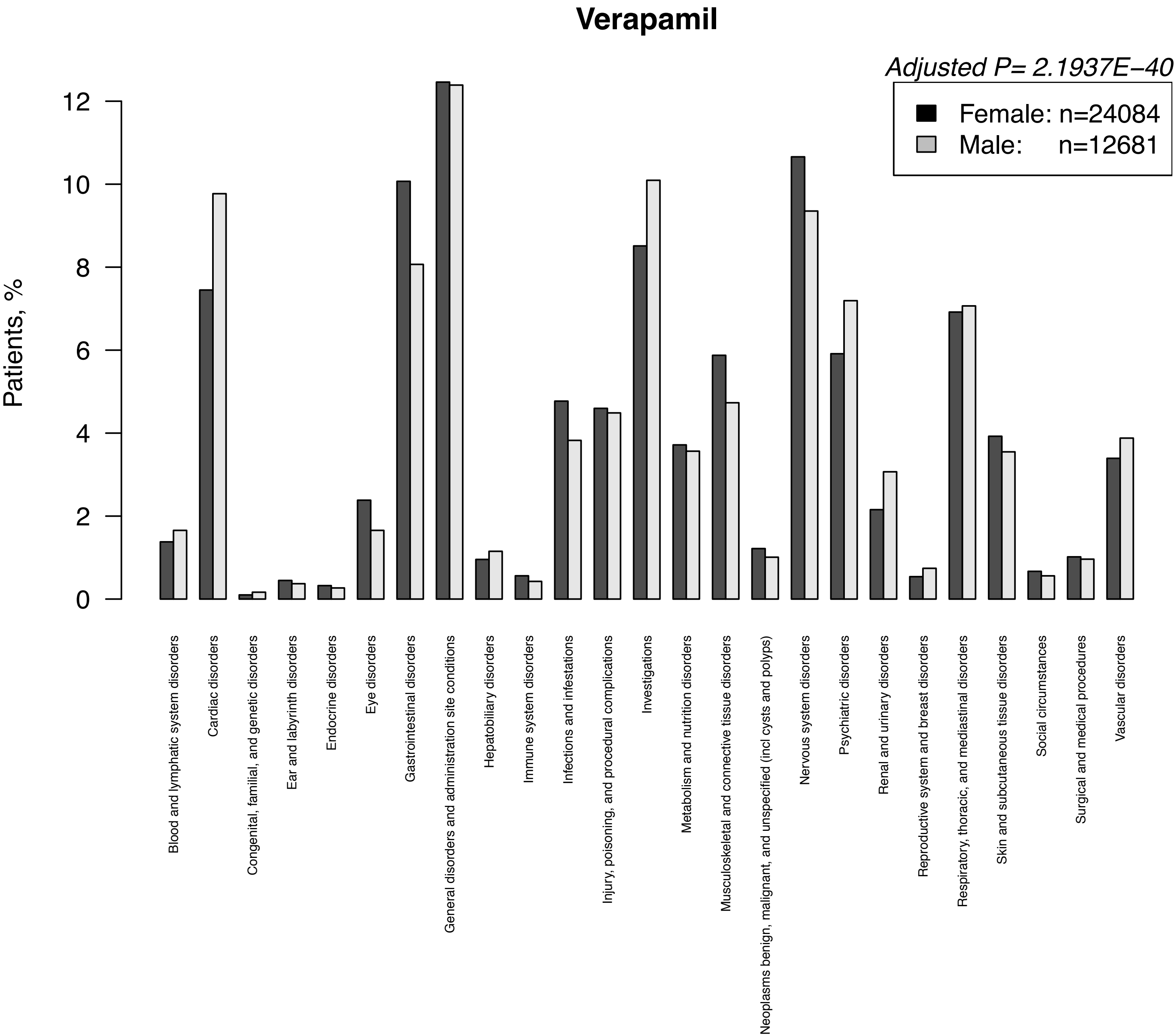

Verapamil
Adjusted P= 2.1937E−40
Female: n=24084
Male: n=12681
Patients, %
0
2
4
6
8
10
12
Blood and lymphatic system disorders
Cardiac disorders
Congenital, familial, and genetic disorders
Ear and labyrinth disorders
Endocrine disorders
Eye disorders
Gastrointestinal disorders
General disorders and administration site conditions
Hepatobiliary disorders
Immune system disorders
Infections and infestations
Injury, poisoning, and procedural complications
Investigations
Metabolism and nutrition disorders
Musculoskeletal and connective tissue disorders
Neoplasms benign, malignant, and unspecified (incl cysts and polyps)
Nervous system disorders
Psychiatric disorders
Renal and urinary disorders
Reproductive system and breast disorders
Respiratory, thoracic, and mediastinal disorders
Skin and subcutaneous tissue disorders
Social circumstances
Surgical and medical procedures
Vascular disorders

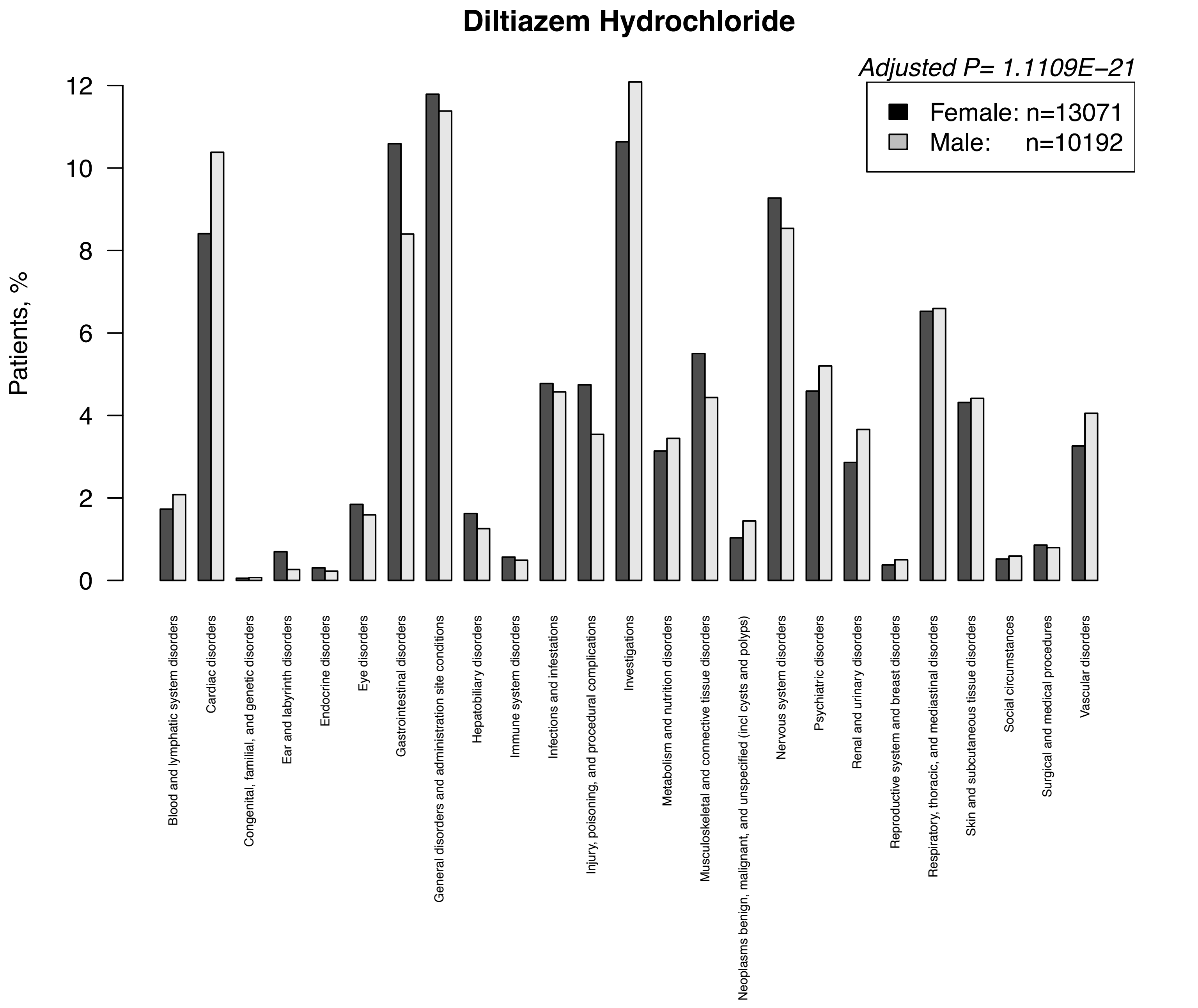
Diltiazem Hydrochloride
Adjusted P= 1.1109E−21
Female: n=13071
Male: n=10192
Patients, %
0
2
4
6
8
10
12
Blood and lymphatic system disorders
Cardiac disorders
Congenital, familial, and genetic disorders
Ear and labyrinth disorders
Endocrine disorders
Eye disorders
Gastrointestinal disorders
General disorders and administration site conditions
Hepatobiliary disorders
Immune system disorders
Infections and infestations
Injury, poisoning, and procedural complications
Investigations
Metabolism and nutrition disorders
Musculoskeletal and connective tissue disorders
Neoplasms benign, malignant, and unspecified (incl cysts and polyps)
Nervous system disorders
Psychiatric disorders
Renal and urinary disorders
Reproductive system and breast disorders
Respiratory, thoracic, and mediastinal disorders
Skin and subcutaneous tissue disorders
Social circumstances
Surgical and medical procedures
Vascular disorders

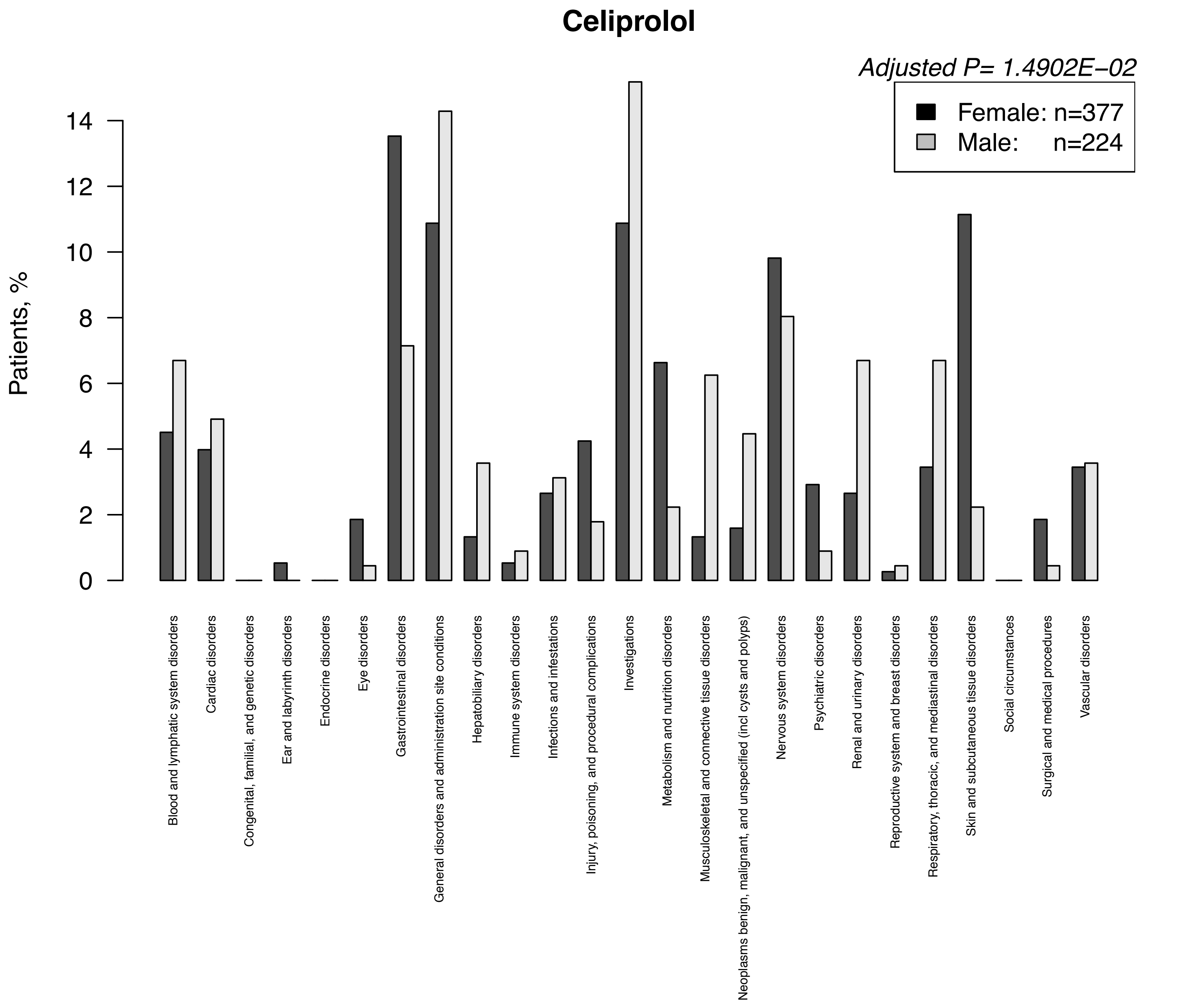

Celiprolol
Adjusted P= 1.4902E−02
Female: n=377
Male: n=224
Patients, %
0
2
4
6
8
10
12
14
Blood and lymphatic system disorders
Cardiac disorders
Congenital, familial, and genetic disorders
Ear and labyrinth disorders
Endocrine disorders
Eye disorders
Gastrointestinal disorders
General disorders and administration site conditions
Hepatobiliary disorders
Immune system disorders
Infections and infestations
Injury, poisoning, and procedural complications
Investigations
Metabolism and nutrition disorders
Musculoskeletal and connective tissue disorders
Neoplasms benign, malignant, and unspecified (incl cysts and polyps)
Nervous system disorders
Psychiatric disorders
Renal and urinary disorders
Reproductive system and breast disorders
Respiratory, thoracic, and mediastinal disorders
Skin and subcutaneous tissue disorders
Social circumstances
Surgical and medical procedures
Vascular disorders

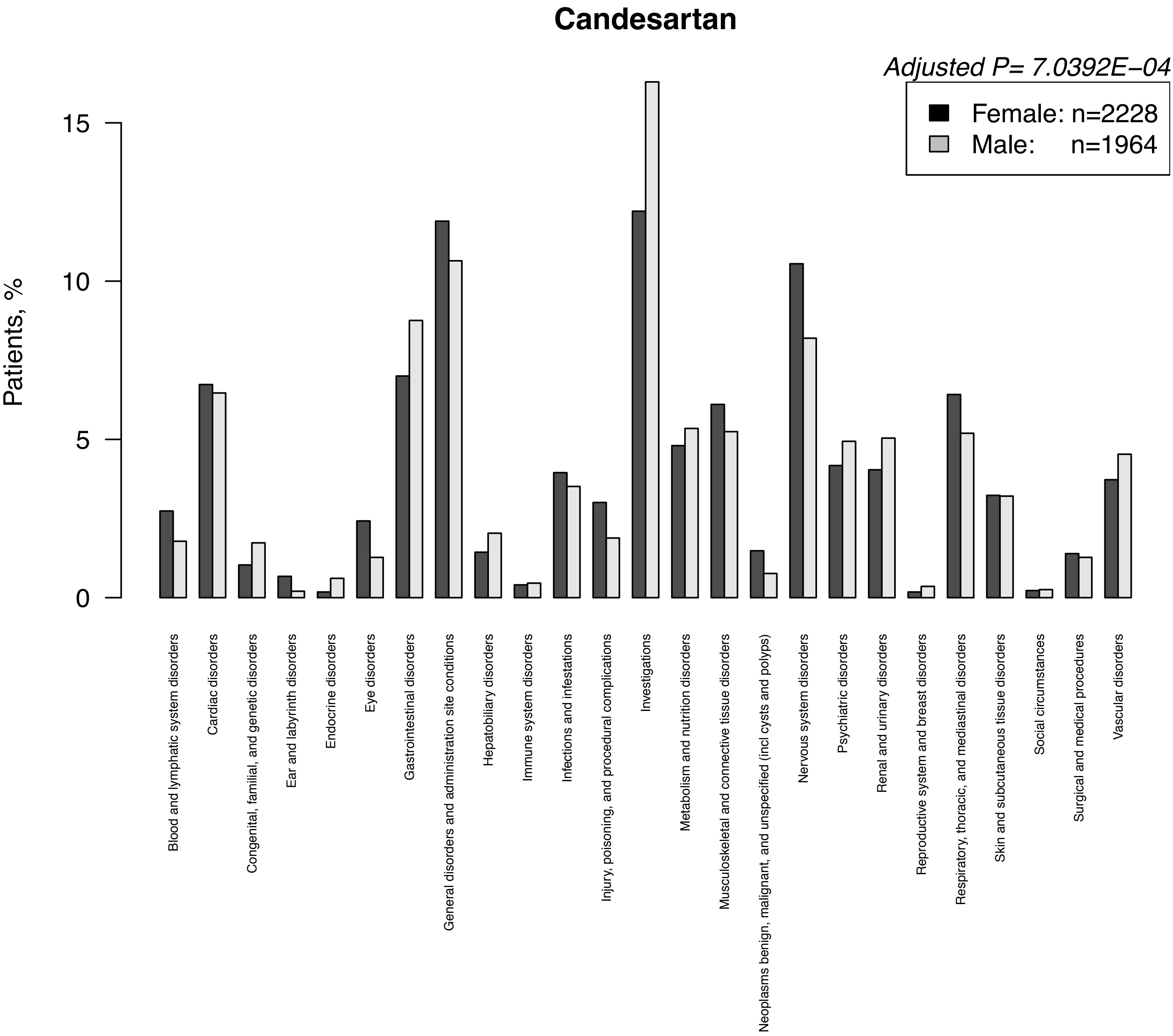
Candesartan
Adjusted P= 7.0392E−04
Female: n=2228
Male: n=1964
Patients, %
15
10
5
0
Blood and lymphatic system disorders
Cardiac disorders
Congenital, familial, and genetic disorders
Ear and labyrinth disorders
Endocrine disorders
Eye disorders
Gastrointestinal disorders
General disorders and administration site conditions
Hepatobiliary disorders
Immune system disorders
Infections and infestations
Injury, poisoning, and procedural complications
Investigations
Metabolism and nutrition disorders
Musculoskeletal and connective tissue disorders
Neoplasms benign, malignant, and unspecified (incl cysts and polyps)
Nervous system disorders
Psychiatric disorders
Renal and urinary disorders
Reproductive system and breast disorders
Respiratory, thoracic, and mediastinal disorders
Skin and subcutaneous tissue disorders
Social circumstances
Surgical and medical procedures
Vascular disorders

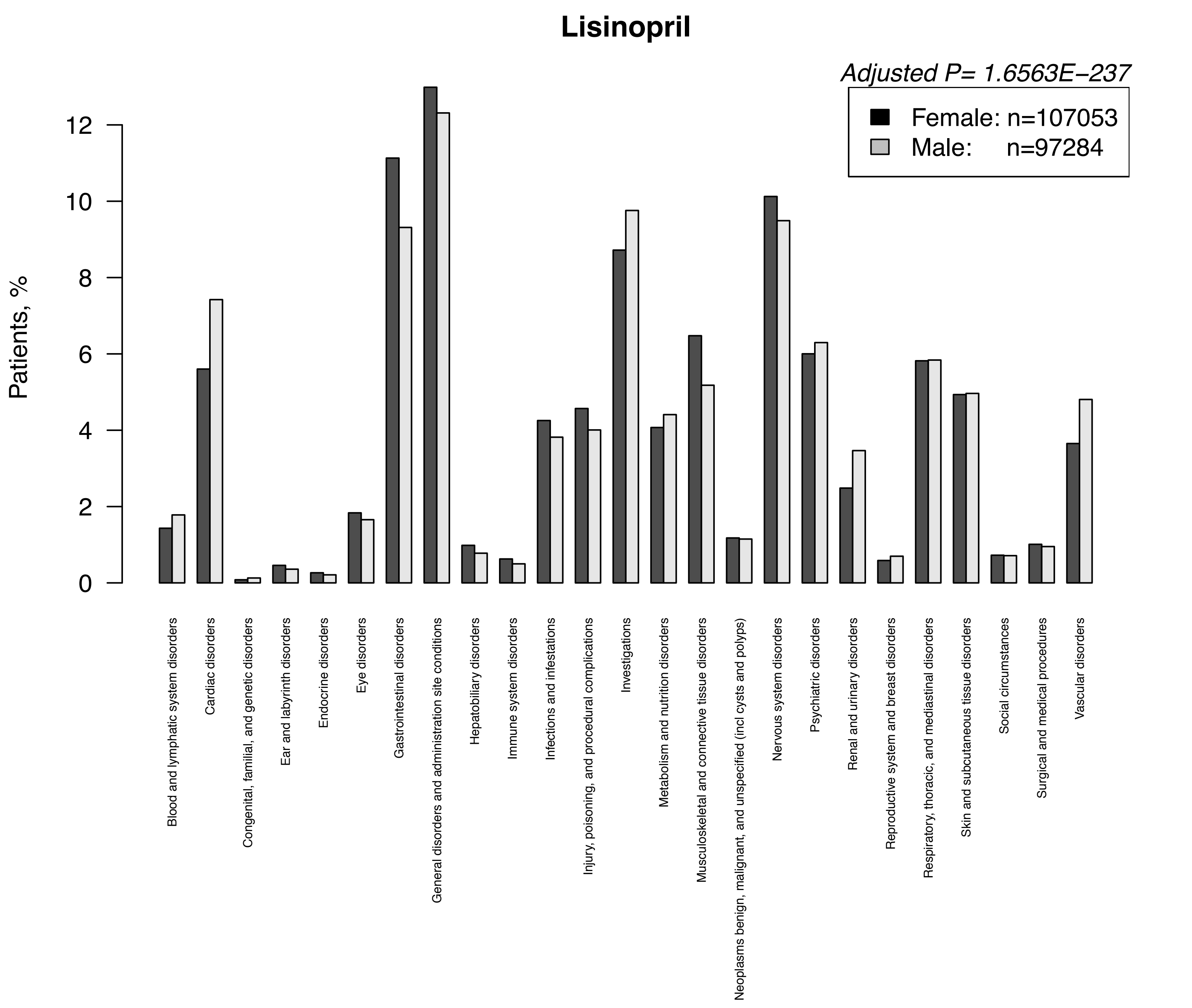

Lisinopril
Adjusted P= 1.6563E−237
Female: n=107053
Male: n=97284
Patients, %
0
2
4
6
8
10
12
Blood and lymphatic system disorders
Cardiac disorders
Congenital, familial, and genetic disorders
Ear and labyrinth disorders
Endocrine disorders
Eye disorders
Gastrointestinal disorders
General disorders and administration site conditions
Hepatobiliary disorders
Immune system disorders
Infections and infestations
Injury, poisoning, and procedural complications
Investigations
Metabolism and nutrition disorders
Musculoskeletal and connective tissue disorders
Neoplasms benign, malignant, and unspecified (incl cysts and polyps)
Nervous system disorders
Psychiatric disorders
Renal and urinary disorders
Reproductive system and breast disorders
Respiratory, thoracic, and mediastinal disorders
Skin and subcutaneous tissue disorders
Social circumstances
Surgical and medical procedures
Vascular disorders

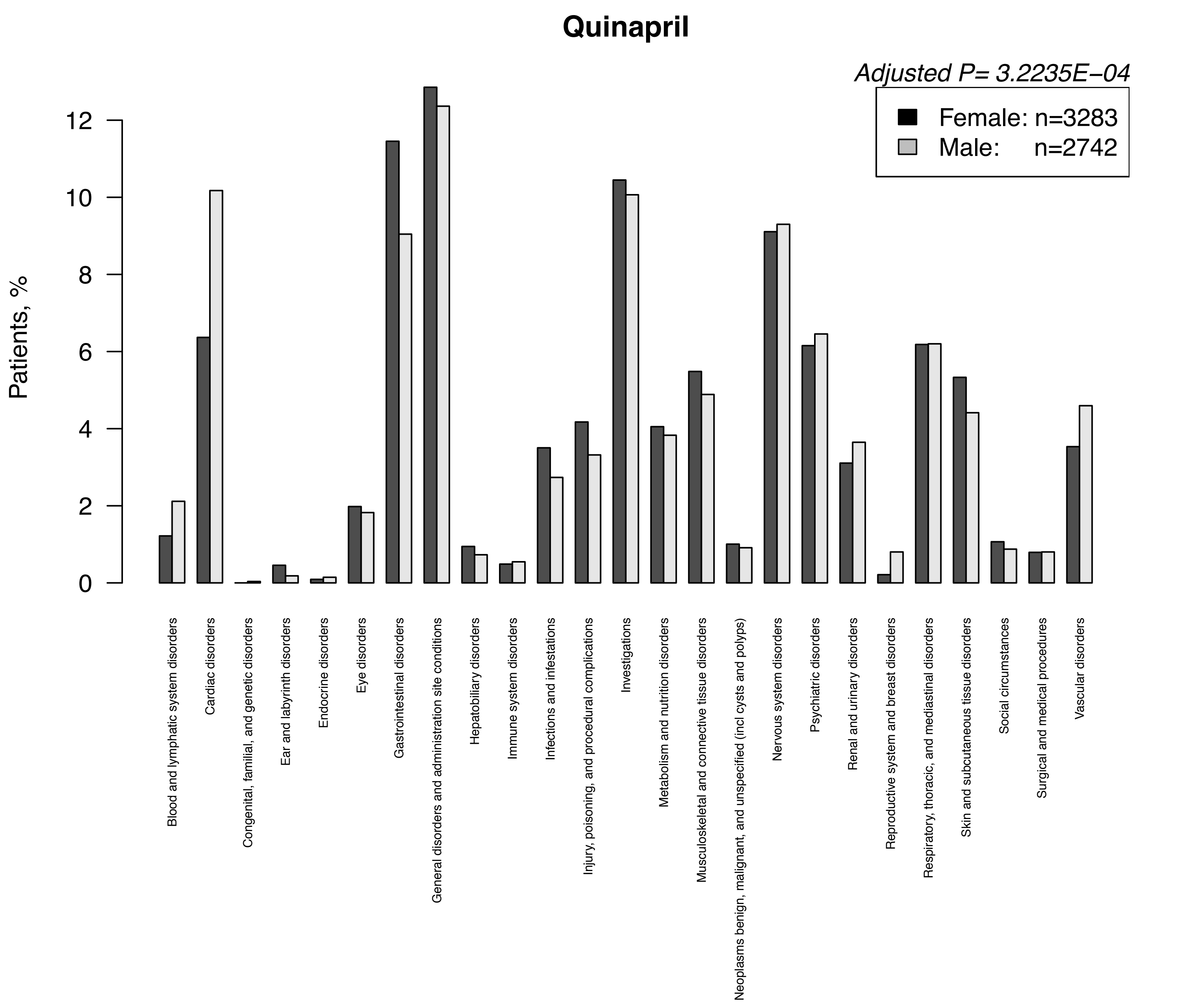
Quinapril
Adjusted P= 3.2235E−04
Female: n=3283
Male: n=2742
Patients, %
0
2
4
6
8
10
12
Blood and lymphatic system disorders
Cardiac disorders
Congenital, familial, and genetic disorders
Ear and labyrinth disorders
Endocrine disorders
Eye disorders
Gastrointestinal disorders
General disorders and administration site conditions
Hepatobiliary disorders
Immune system disorders
Infections and infestations
Injury, poisoning, and procedural complications
Investigations
Metabolism and nutrition disorders
Musculoskeletal and connective tissue disorders
Neoplasms benign, malignant, and unspecified (incl cysts and polyps)
Nervous system disorders
Psychiatric disorders
Renal and urinary disorders
Reproductive system and breast disorders
Respiratory, thoracic, and mediastinal disorders
Skin and subcutaneous tissue disorders
Social circumstances
Surgical and medical procedures
Vascular disorders

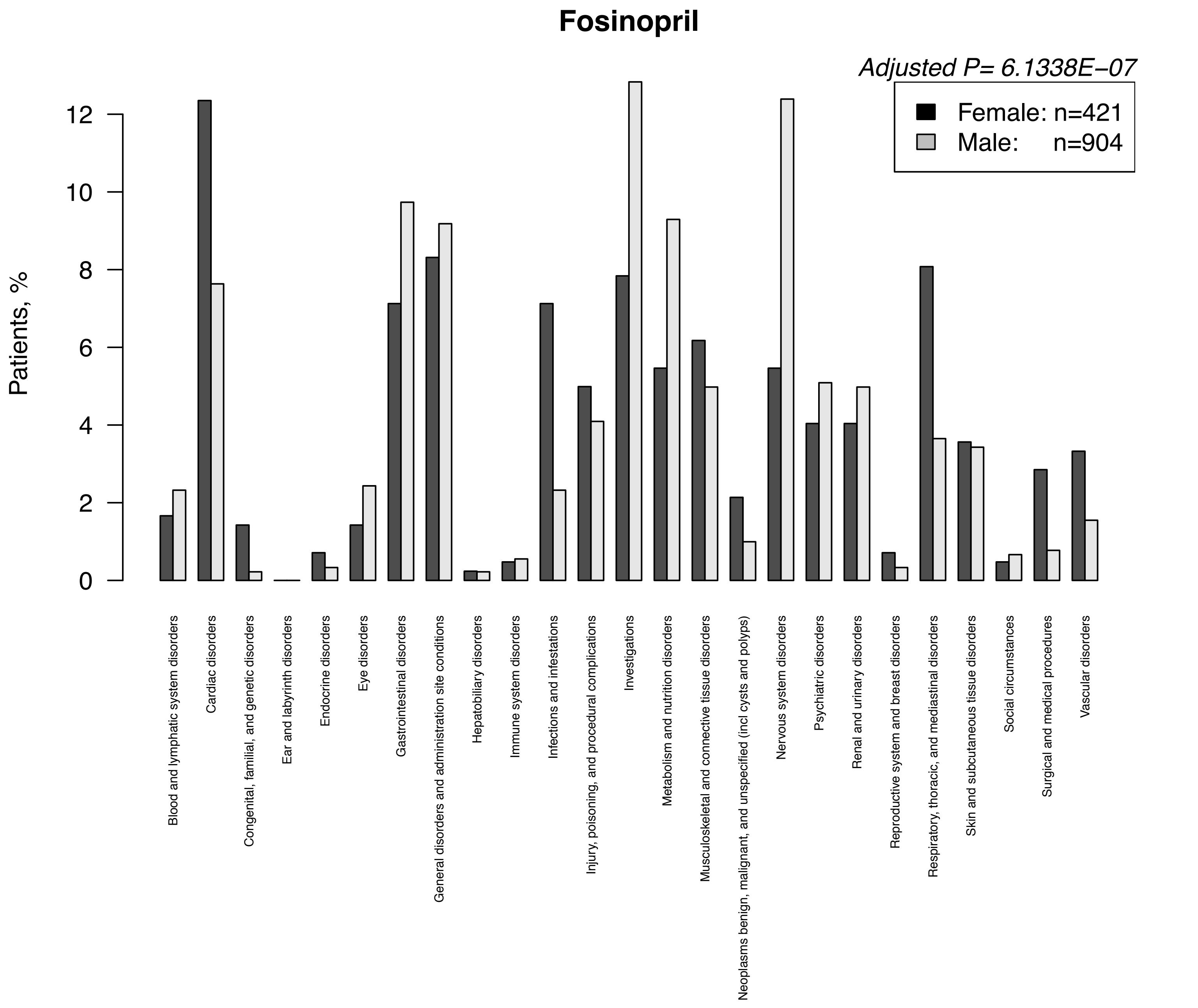

Fosinopril
Adjusted P= 6.1338E−07
Female: n=421
Male: n=904
Patients, %
0
2
4
6
8
10
12
Blood and lymphatic system disorders
Cardiac disorders
Congenital, familial, and genetic disorders
Ear and labyrinth disorders
Endocrine disorders
Eye disorders
Gastrointestinal disorders
General disorders and administration site conditions
Hepatobiliary disorders
Immune system disorders
Infections and infestations
Injury, poisoning, and procedural complications
Investigations
Metabolism and nutrition disorders
Musculoskeletal and connective tissue disorders
Neoplasms benign, malignant, and unspecified (incl cysts and polyps)
Nervous system disorders
Psychiatric disorders
Renal and urinary disorders
Reproductive system and breast disorders
Respiratory, thoracic, and mediastinal disorders
Skin and subcutaneous tissue disorders
Social circumstances
Surgical and medical procedures
Vascular disorders

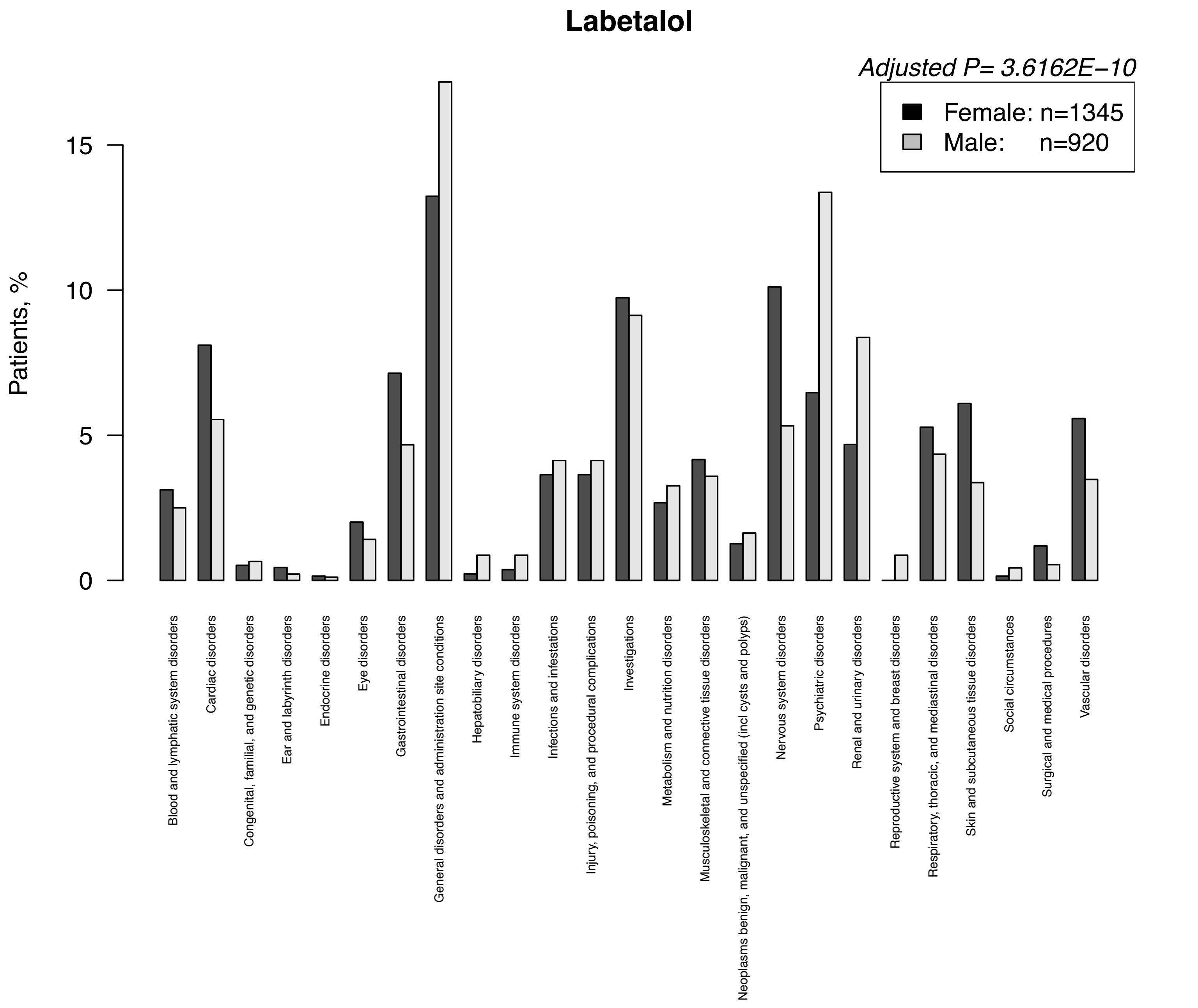
Labetalol
Adjusted P= 3.6162E−10
Female: n=1345
Male: n=920
Patients, %
15
10
5
0
Blood and lymphatic system disorders
Cardiac disorders
Congenital, familial, and genetic disorders
Ear and labyrinth disorders
Endocrine disorders
Eye disorders
Gastrointestinal disorders
General disorders and administration site conditions
Hepatobiliary disorders
Immune system disorders
Infections and infestations
Injury, poisoning, and procedural complications
Investigations
Metabolism and nutrition disorders
Musculoskeletal and connective tissue disorders
Neoplasms benign, malignant, and unspecified (incl cysts and polyps)
Nervous system disorders
Psychiatric disorders
Renal and urinary disorders
Reproductive system and breast disorders
Respiratory, thoracic, and mediastinal disorders
Skin and subcutaneous tissue disorders
Social circumstances
Surgical and medical procedures
Vascular disorders

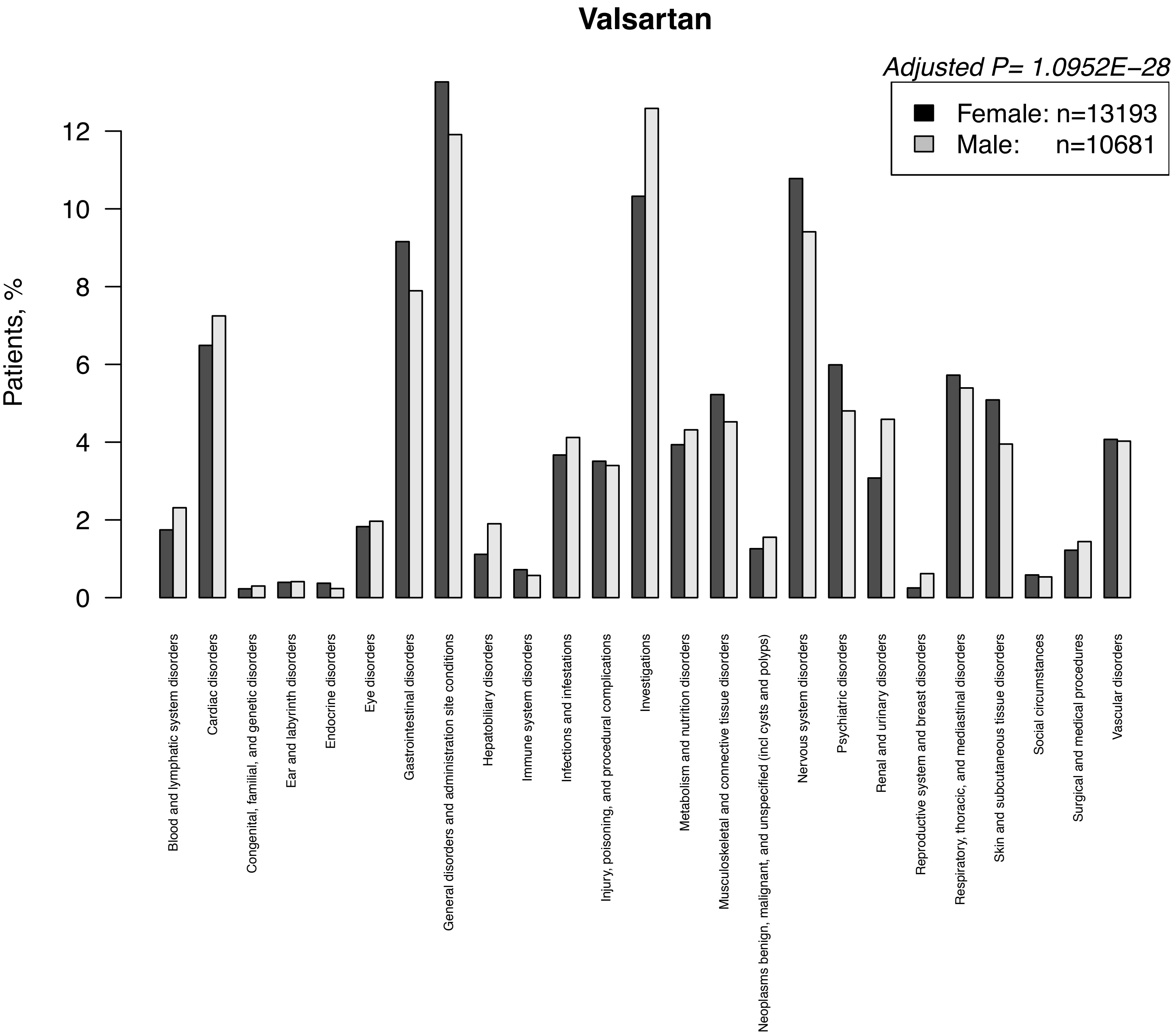
Valsartan
Adjusted P= 1.0952E−28
Female: n=13193
Male: n=10681
Patients, %
0
2
4
6
8
10
12
Blood and lymphatic system disorders
Cardiac disorders
Congenital, familial, and genetic disorders
Ear and labyrinth disorders
Endocrine disorders
Eye disorders
Gastrointestinal disorders
General disorders and administration site conditions
Hepatobiliary disorders
Immune system disorders
Infections and infestations
Injury, poisoning, and procedural complications
Investigations
Metabolism and nutrition disorders
Musculoskeletal and connective tissue disorders
Neoplasms benign, malignant, and unspecified (incl cysts and polyps)
Nervous system disorders
Psychiatric disorders
Renal and urinary disorders
Reproductive system and breast disorders
Respiratory, thoracic, and mediastinal disorders
Skin and subcutaneous tissue disorders
Social circumstances
Surgical and medical procedures
Vascular disorders

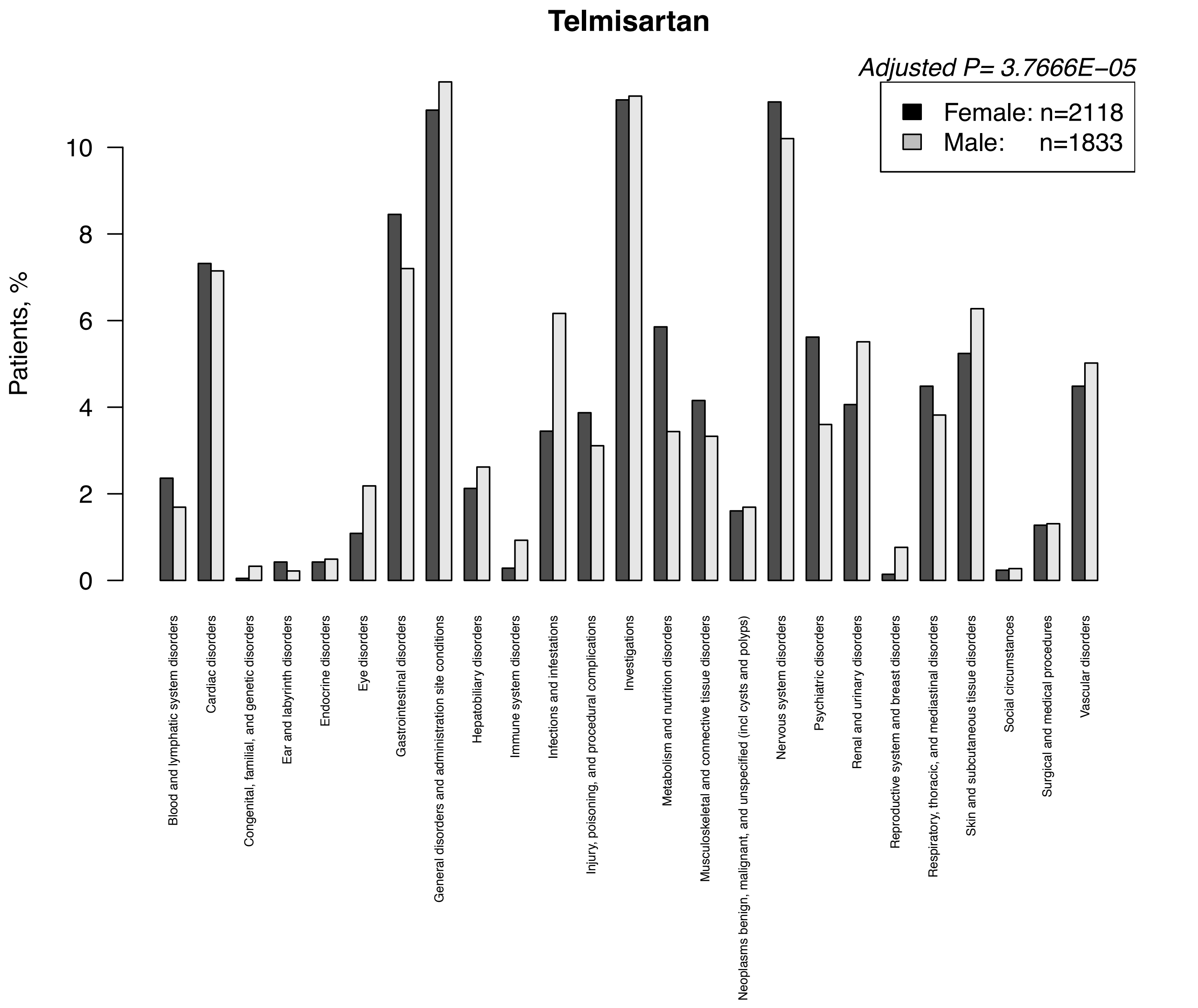

Telmisartan
Adjusted P= 3.7666E−05
Female: n=2118
Male: n=1833
Patients, %
0
2
4
6
8
10
Blood and lymphatic system disorders
Cardiac disorders
Congenital, familial, and genetic disorders
Ear and labyrinth disorders
Endocrine disorders
Eye disorders
Gastrointestinal disorders
General disorders and administration site conditions
Hepatobiliary disorders
Immune system disorders
Infections and infestations
Injury, poisoning, and procedural complications
Investigations
Metabolism and nutrition disorders
Musculoskeletal and connective tissue disorders
Neoplasms benign, malignant, and unspecified (incl cysts and polyps)
Nervous system disorders
Psychiatric disorders
Renal and urinary disorders
Reproductive system and breast disorders
Respiratory, thoracic, and mediastinal disorders
Skin and subcutaneous tissue disorders
Social circumstances
Surgical and medical procedures
Vascular disorders

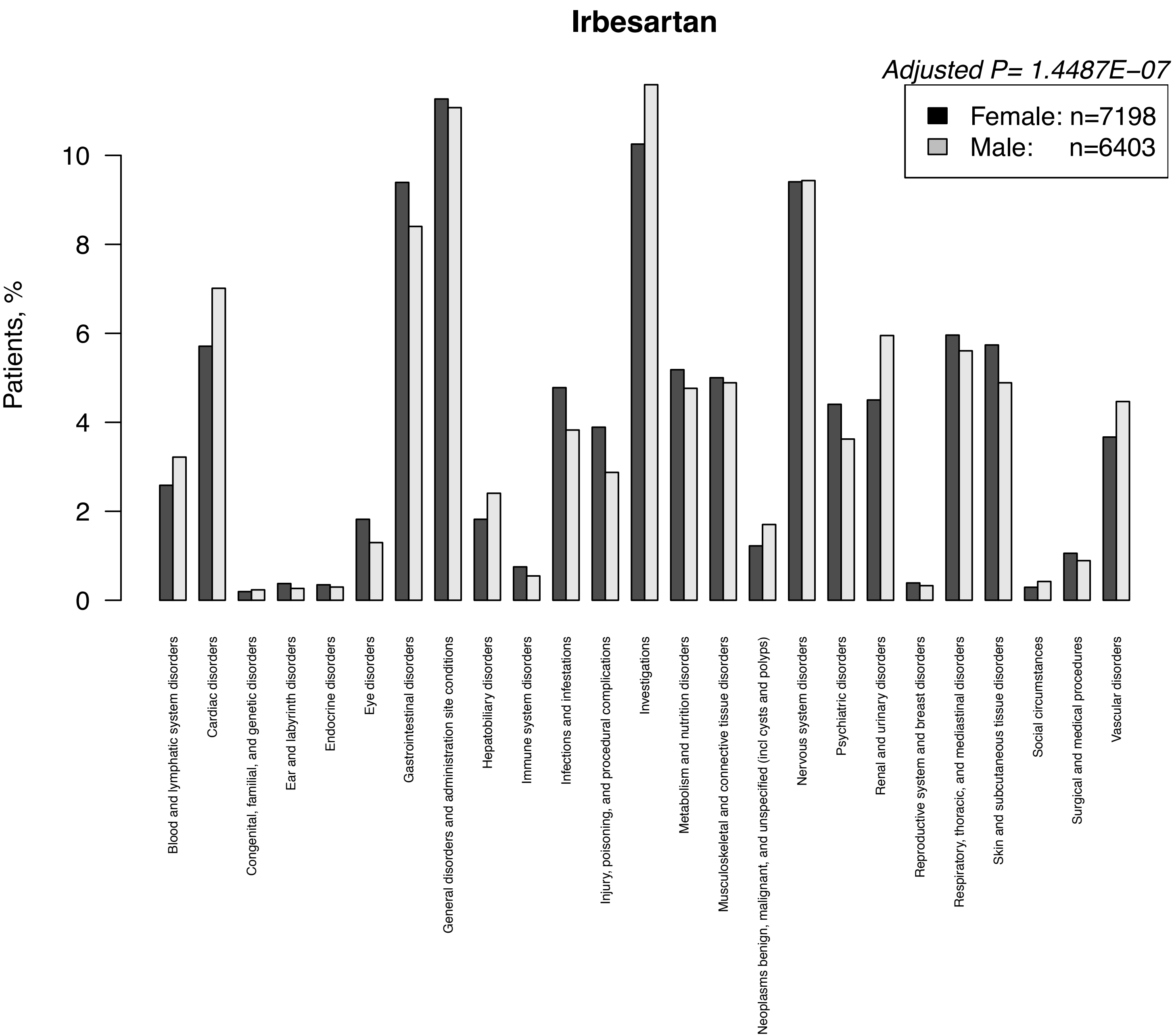
Irbesartan
Adjusted P= 1.4487E−07
Female: n=7198
Male: n=6403
Patients, %
0
2
4
6
8
10
Blood and lymphatic system disorders
Cardiac disorders
Congenital, familial, and genetic disorders
Ear and labyrinth disorders
Endocrine disorders
Eye disorders
Gastrointestinal disorders
General disorders and administration site conditions
Hepatobiliary disorders
Immune system disorders
Infections and infestations
Injury, poisoning, and procedural complications
Investigations
Metabolism and nutrition disorders
Musculoskeletal and connective tissue disorders
Neoplasms benign, malignant, and unspecified (incl cysts and polyps)
Nervous system disorders
Psychiatric disorders
Renal and urinary disorders
Reproductive system and breast disorders
Respiratory, thoracic, and mediastinal disorders
Skin and subcutaneous tissue disorders
Social circumstances
Surgical and medical procedures
Vascular disorders

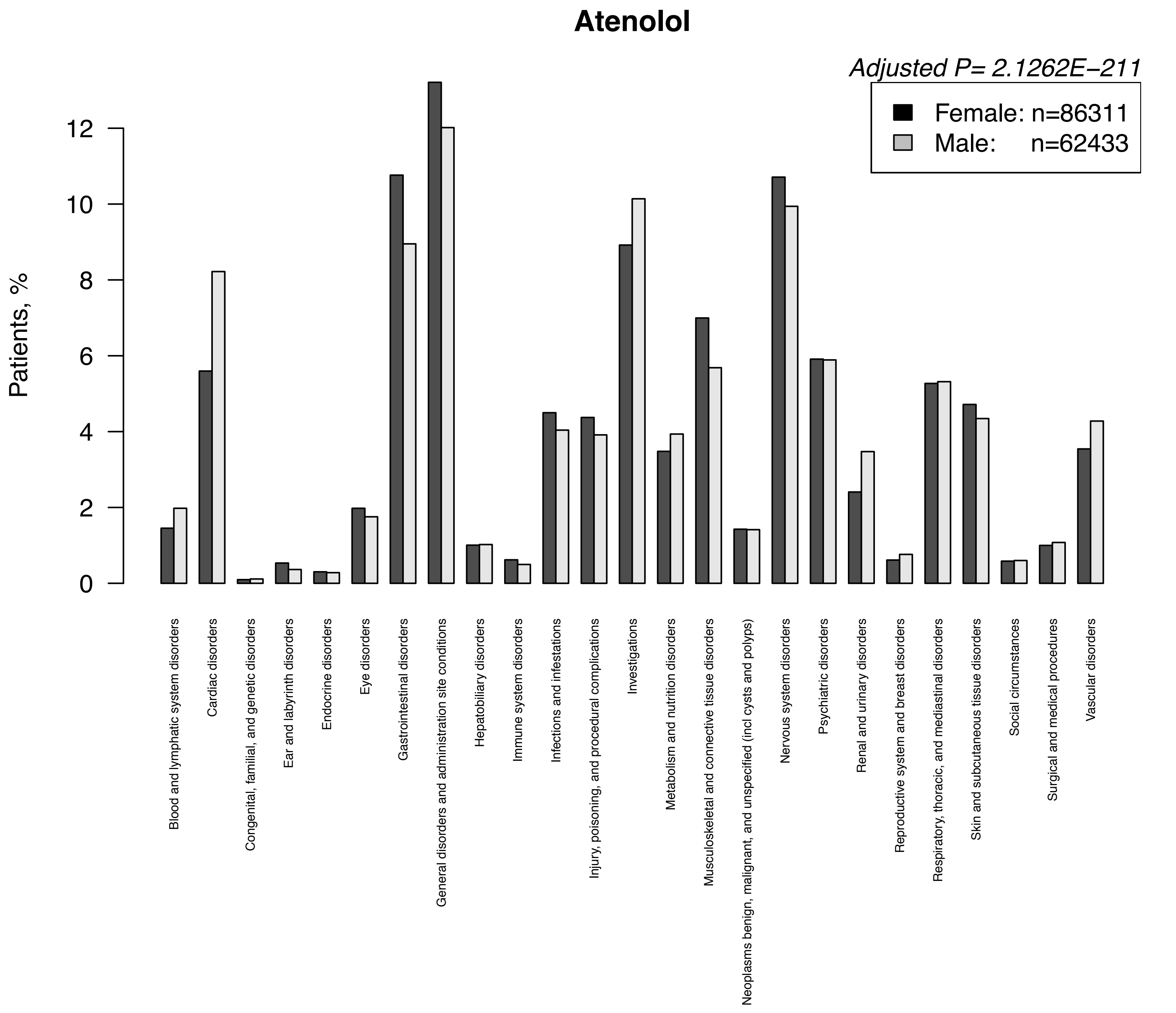
Atenolol
Adjusted P= 2.1262E−211
Female: n=86311
Male: n=62433
Patients, %
0
2
4
6
8
10
12
Blood and lymphatic system disorders
Cardiac disorders
Congenital, familial, and genetic disorders
Ear and labyrinth disorders
Endocrine disorders
Eye disorders
Gastrointestinal disorders
General disorders and administration site conditions
Hepatobiliary disorders
Immune system disorders
Infections and infestations
Injury, poisoning, and procedural complications
Investigations
Metabolism and nutrition disorders
Musculoskeletal and connective tissue disorders
Neoplasms benign, malignant, and unspecified (incl cysts and polyps)
Nervous system disorders
Psychiatric disorders
Renal and urinary disorders
Reproductive system and breast disorders
Respiratory, thoracic, and mediastinal disorders
Skin and subcutaneous tissue disorders
Social circumstances
Surgical and medical procedures
Vascular disorders

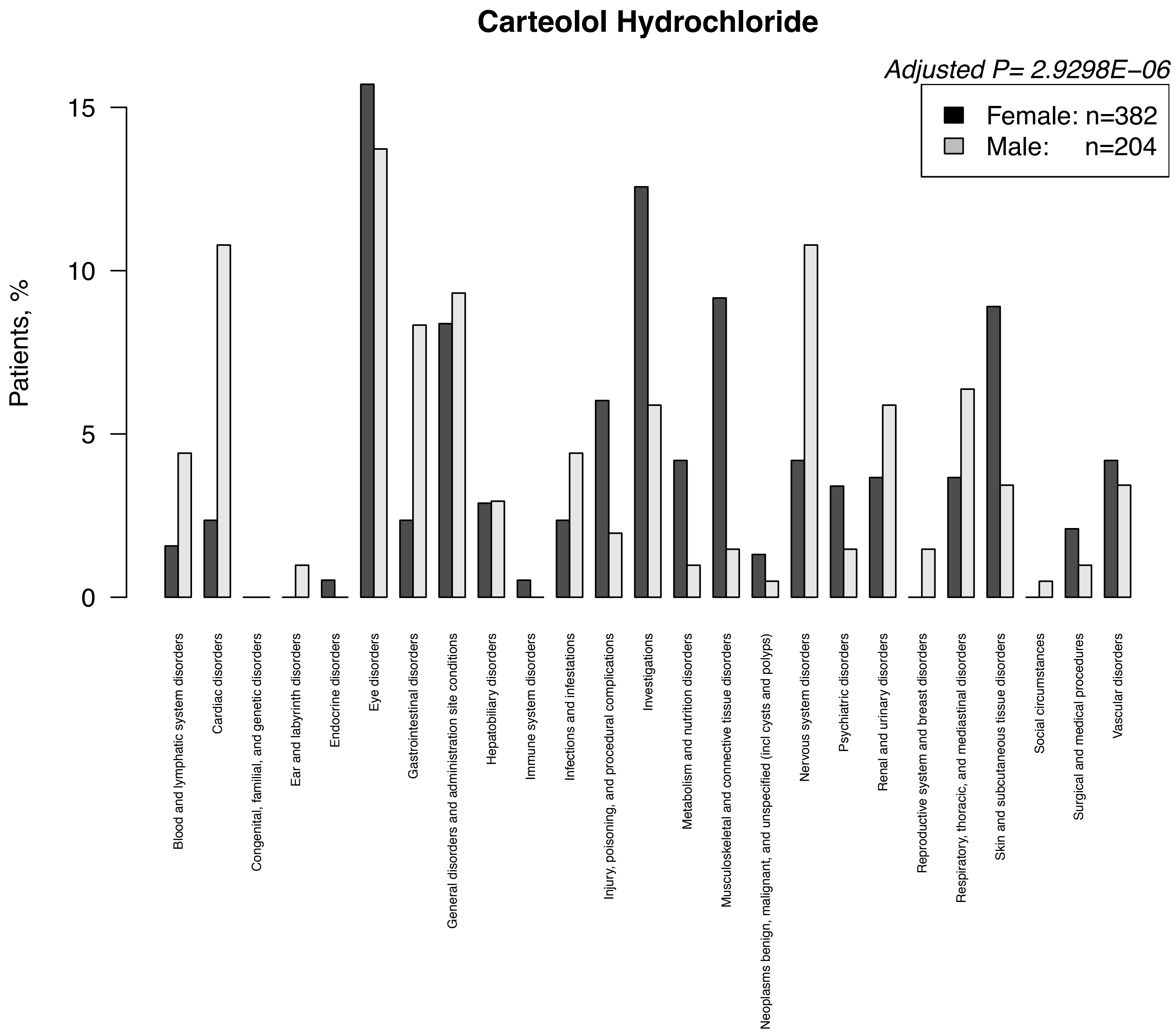

Carteolol Hydrochloride
Adjusted P= 2.9298E−06
Female: n=382
Male: n=204
Patients, %
15
10
5
0
Blood and lymphatic system disorders
Cardiac disorders
Congenital, familial, and genetic disorders
Ear and labyrinth disorders
Endocrine disorders
Eye disorders
Gastrointestinal disorders
General disorders and administration site conditions
Hepatobiliary disorders
Immune system disorders
Infections and infestations
Injury, poisoning, and procedural complications
Investigations
Metabolism and nutrition disorders
Musculoskeletal and connective tissue disorders
Neoplasms benign, malignant, and unspecified (incl cysts and polyps)
Nervous system disorders
Psychiatric disorders
Renal and urinary disorders
Reproductive system and breast disorders
Respiratory, thoracic, and mediastinal disorders
Skin and subcutaneous tissue disorders
Social circumstances
Surgical and medical procedures
Vascular disorders

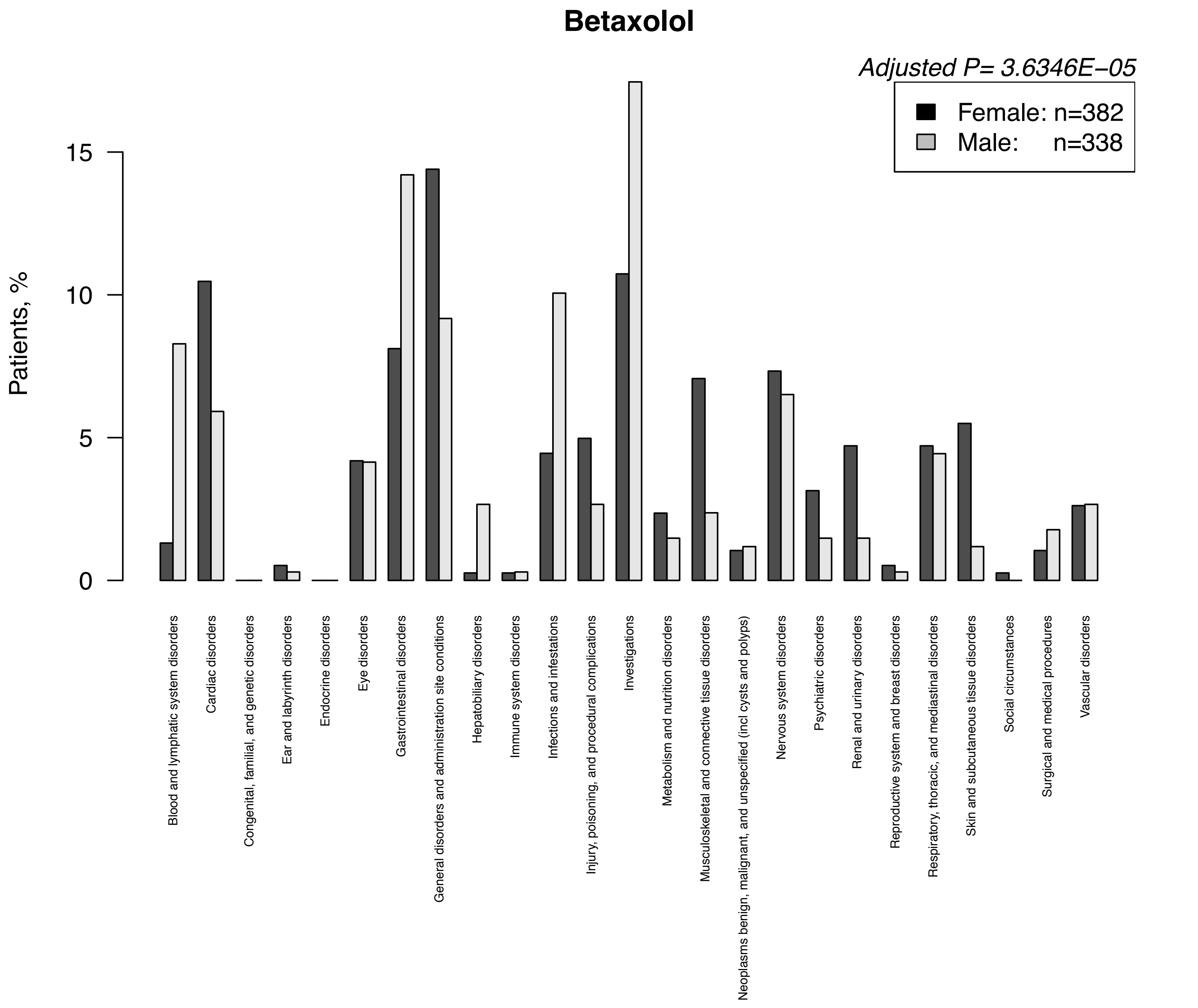

Betaxolol
Adjusted P= 3.6346E−05
Female: n=382
Male: n=338
Patients, %
0
5
10
15
Blood and lymphatic system disorders
Cardiac disorders
Congenital, familial, and genetic disorders
Ear and labyrinth disorders
Endocrine disorders
Eye disorders
Gastrointestinal disorders
General disorders and administration site conditions
Hepatobiliary disorders
Immune system disorders
Infections and infestations
Injury, poisoning, and procedural complications
Investigations
Metabolism and nutrition disorders
Musculoskeletal and connective tissue disorders
Neoplasms benign, malignant, and unspecified (incl cysts and polyps)
Nervous system disorders
Psychiatric disorders
Renal and urinary disorders
Reproductive system and breast disorders
Respiratory, thoracic, and mediastinal disorders
Skin and subcutaneous tissue disorders
Social circumstances
Surgical and medical procedures
Vascular disorders

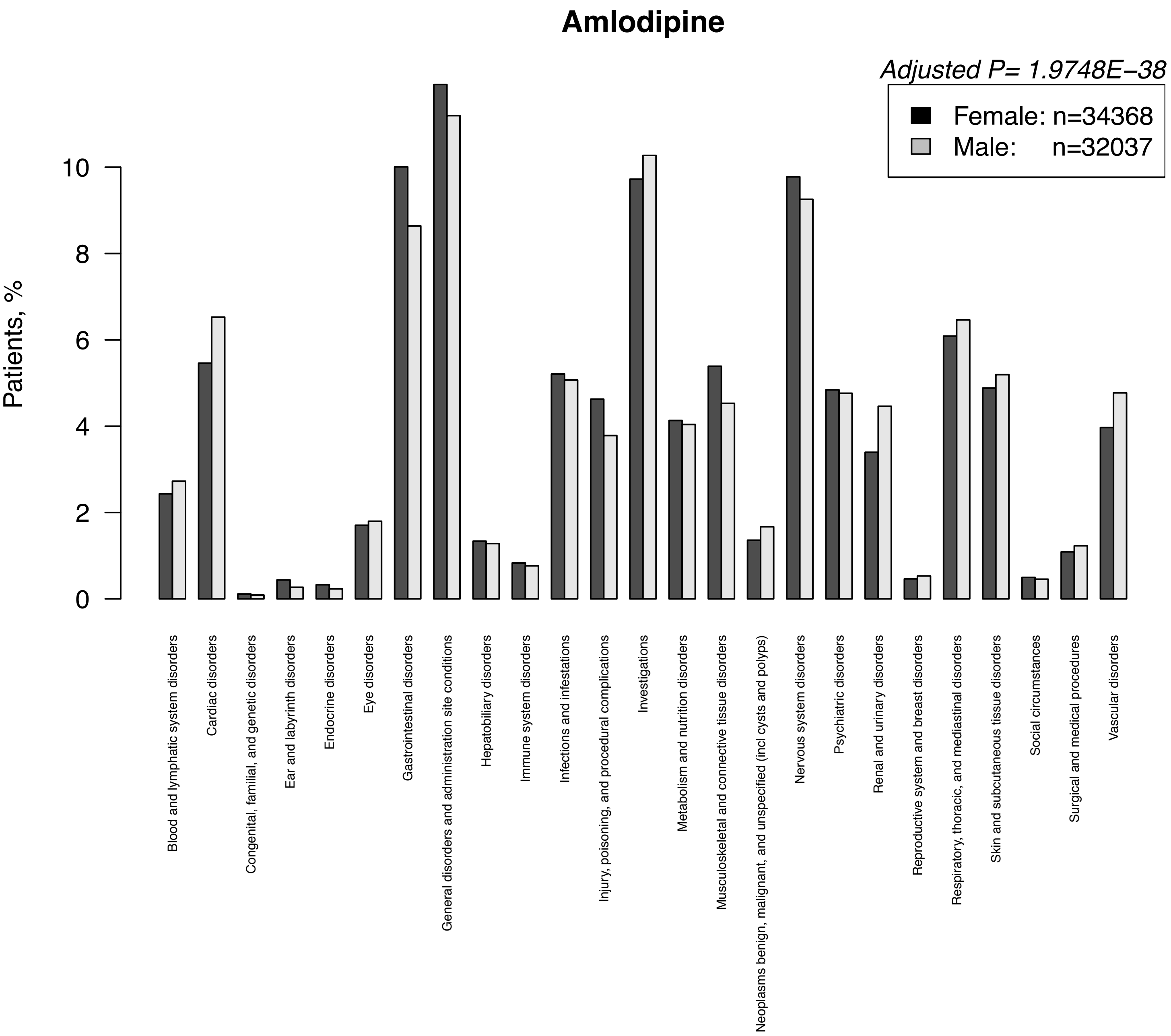

Amlodipine
Adjusted P= 1.9748E−38
Female: n=34368
Male: n=32037
Patients, %
0
2
4
6
8
10
Blood and lymphatic system disorders
Cardiac disorders
Congenital, familial, and genetic disorders
Ear and labyrinth disorders
Endocrine disorders
Eye disorders
Gastrointestinal disorders
General disorders and administration site conditions
Hepatobiliary disorders
Immune system disorders
Infections and infestations
Injury, poisoning, and procedural complications
Investigations
Metabolism and nutrition disorders
Musculoskeletal and connective tissue disorders
Neoplasms benign, malignant, and unspecified (incl cysts and polyps)
Nervous system disorders
Psychiatric disorders
Renal and urinary disorders
Reproductive system and breast disorders
Respiratory, thoracic, and mediastinal disorders
Skin and subcutaneous tissue disorders
Social circumstances
Surgical and medical procedures
Vascular disorders

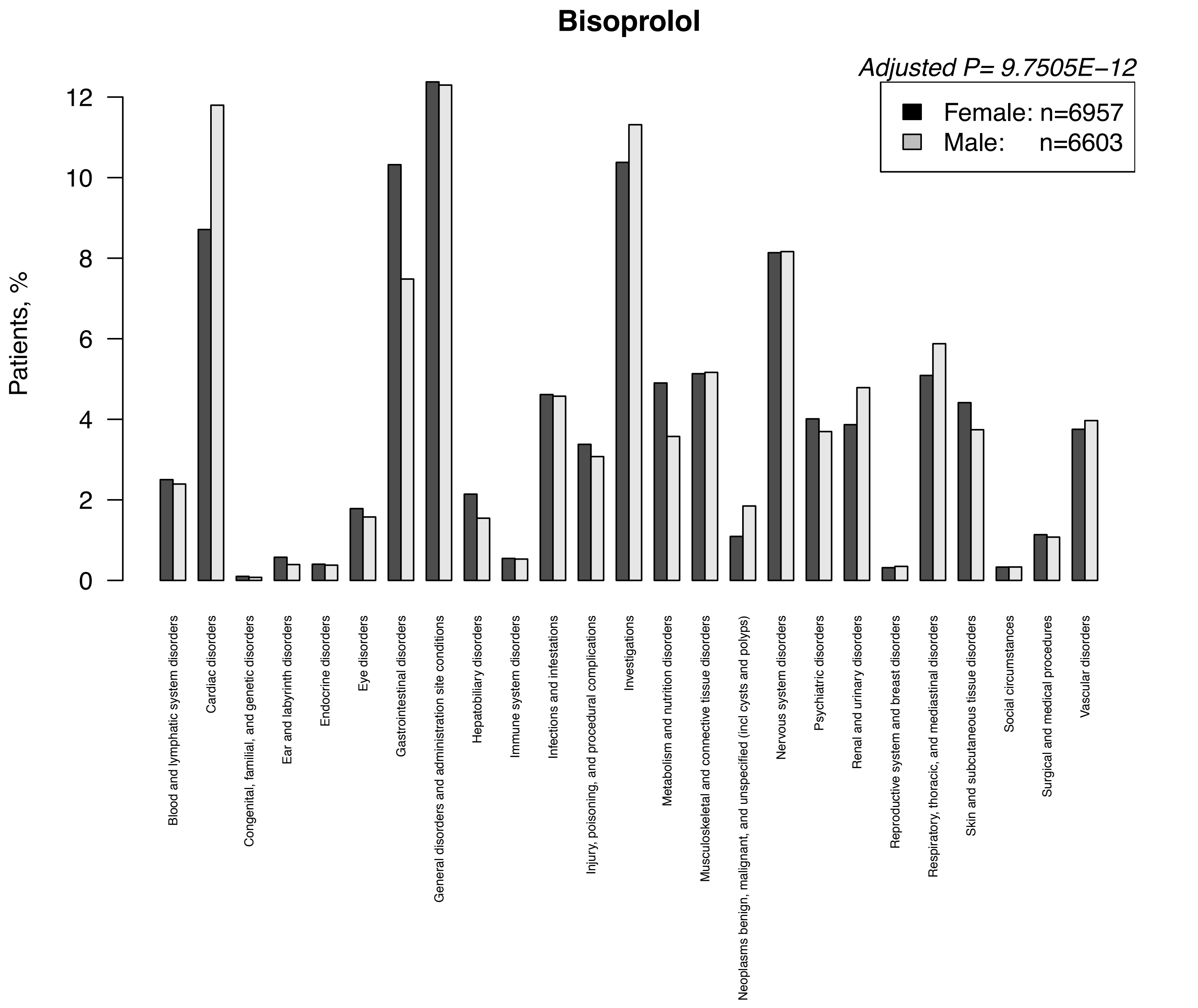

Bisoprolol
Adjusted P= 9.7505E−12
Female: n=6957
Male: n=6603
Patients, %
0
2
4
6
8
10
12
Blood and lymphatic system disorders
Cardiac disorders
Congenital, familial, and genetic disorders
Ear and labyrinth disorders
Endocrine disorders
Eye disorders
Gastrointestinal disorders
General disorders and administration site conditions
Hepatobiliary disorders
Immune system disorders
Infections and infestations
Injury, poisoning, and procedural complications
Investigations
Metabolism and nutrition disorders
Musculoskeletal and connective tissue disorders
Neoplasms benign, malignant, and unspecified (incl cysts and polyps)
Nervous system disorders
Psychiatric disorders
Renal and urinary disorders
Reproductive system and breast disorders
Respiratory, thoracic, and mediastinal disorders
Skin and subcutaneous tissue disorders
Social circumstances
Surgical and medical procedures
Vascular disorders

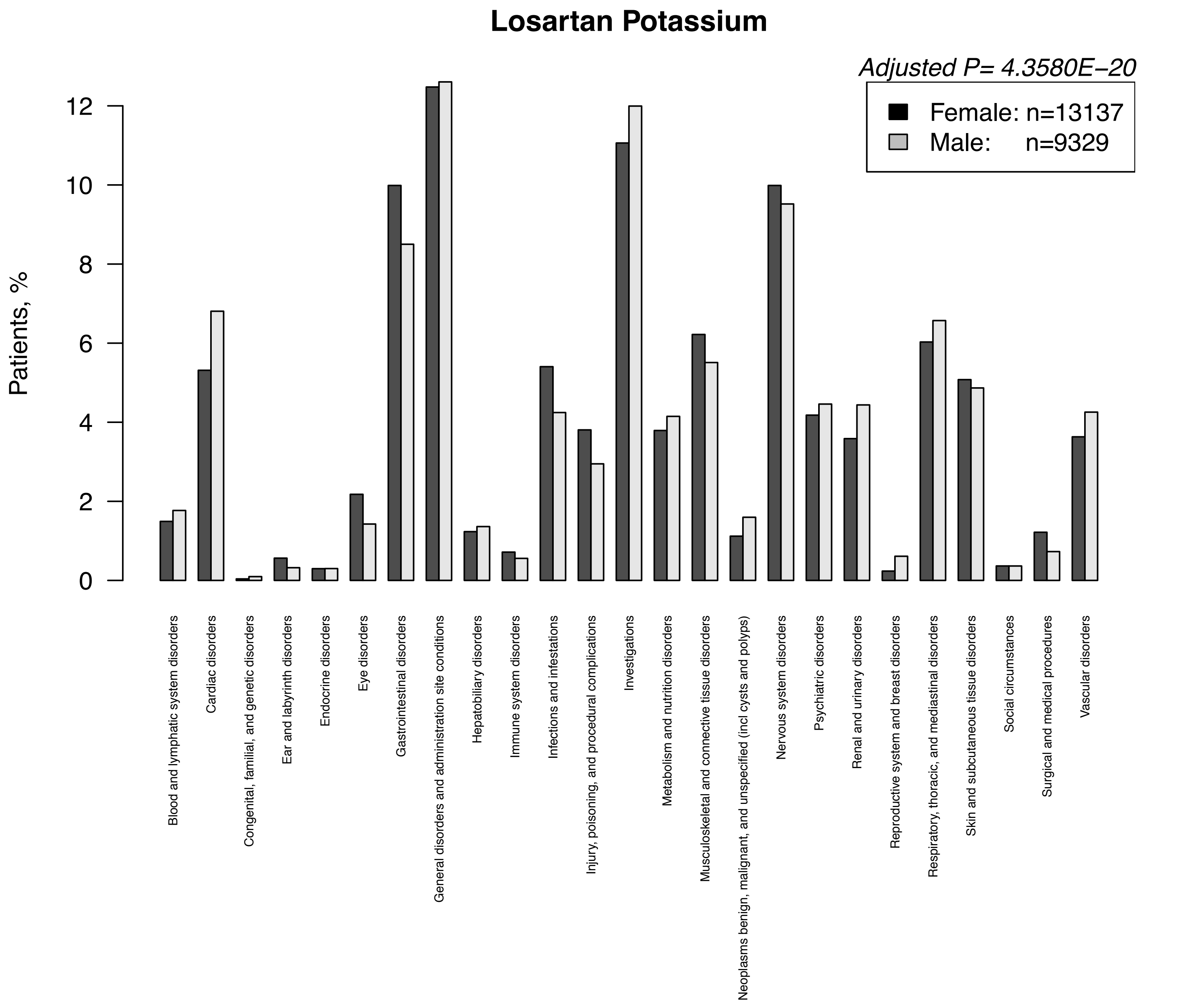
Losartan Potassium
Adjusted P= 4.3580E−20
Female: n=13137
Male: n=9329
Patients, %
0
2
4
6
8
10
12
Blood and lymphatic system disorders
Cardiac disorders
Congenital, familial, and genetic disorders
Ear and labyrinth disorders
Endocrine disorders
Eye disorders
Gastrointestinal disorders
General disorders and administration site conditions
Hepatobiliary disorders
Immune system disorders
Infections and infestations
Injury, poisoning, and procedural complications
Investigations
Metabolism and nutrition disorders
Musculoskeletal and connective tissue disorders
Neoplasms benign, malignant, and unspecified (incl cysts and polyps)
Nervous system disorders
Psychiatric disorders
Renal and urinary disorders
Reproductive system and breast disorders
Respiratory, thoracic, and mediastinal disorders
Skin and subcutaneous tissue disorders
Social circumstances
Surgical and medical procedures
Vascular disorders

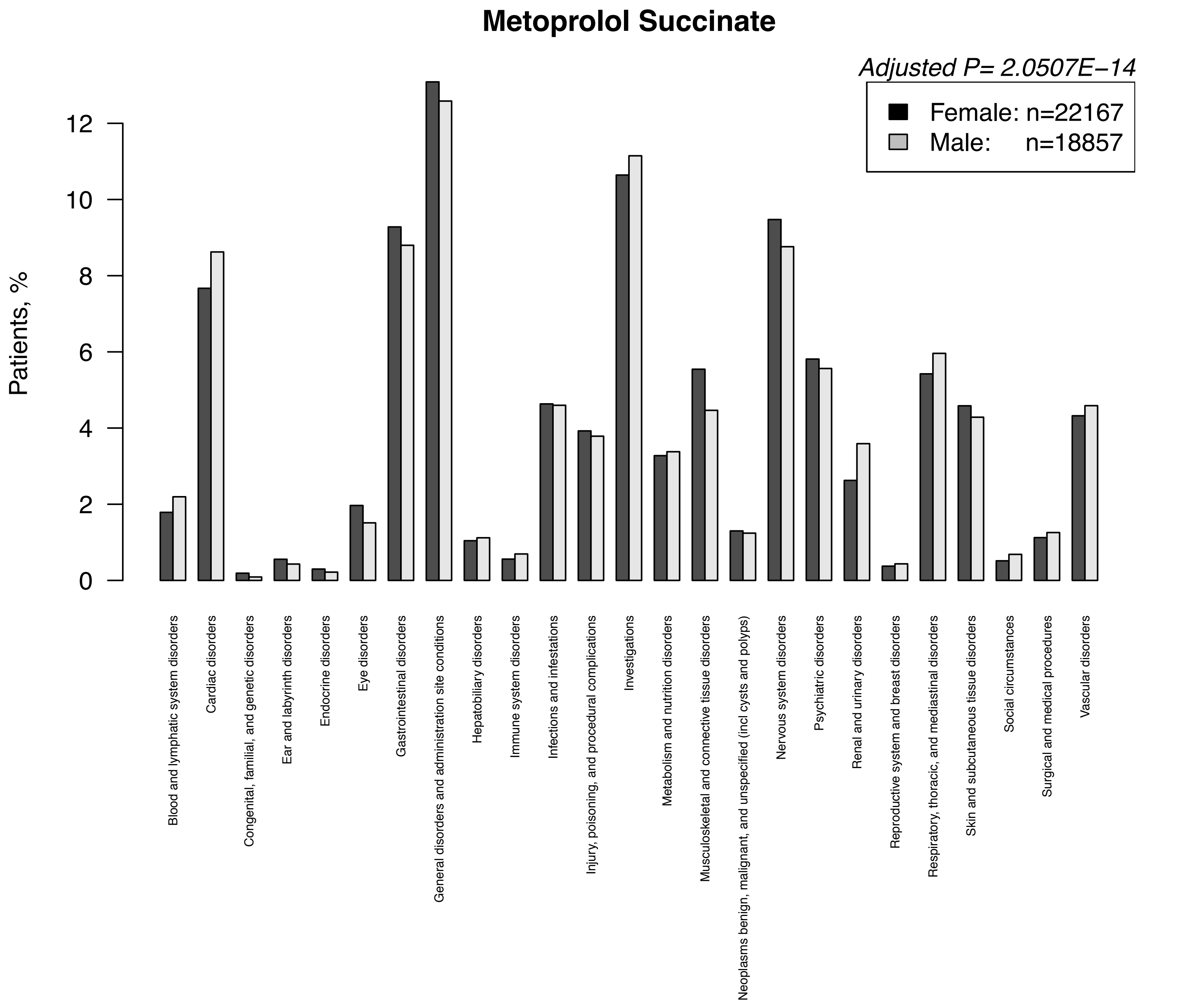
Metoprolol Succinate
Adjusted P= 2.0507E−14
Female: n=22167
Male: n=18857
Patients, %
0
2
4
6
8
10
12
Blood and lymphatic system disorders
Cardiac disorders
Congenital, familial, and genetic disorders
Ear and labyrinth disorders
Endocrine disorders
Eye disorders
Gastrointestinal disorders
General disorders and administration site conditions
Hepatobiliary disorders
Immune system disorders
Infections and infestations
Injury, poisoning, and procedural complications
Investigations
Metabolism and nutrition disorders
Musculoskeletal and connective tissue disorders
Neoplasms benign, malignant, and unspecified (incl cysts and polyps)
Nervous system disorders
Psychiatric disorders
Renal and urinary disorders
Reproductive system and breast disorders
Respiratory, thoracic, and mediastinal disorders
Skin and subcutaneous tissue disorders
Social circumstances
Surgical and medical procedures
Vascular disorders

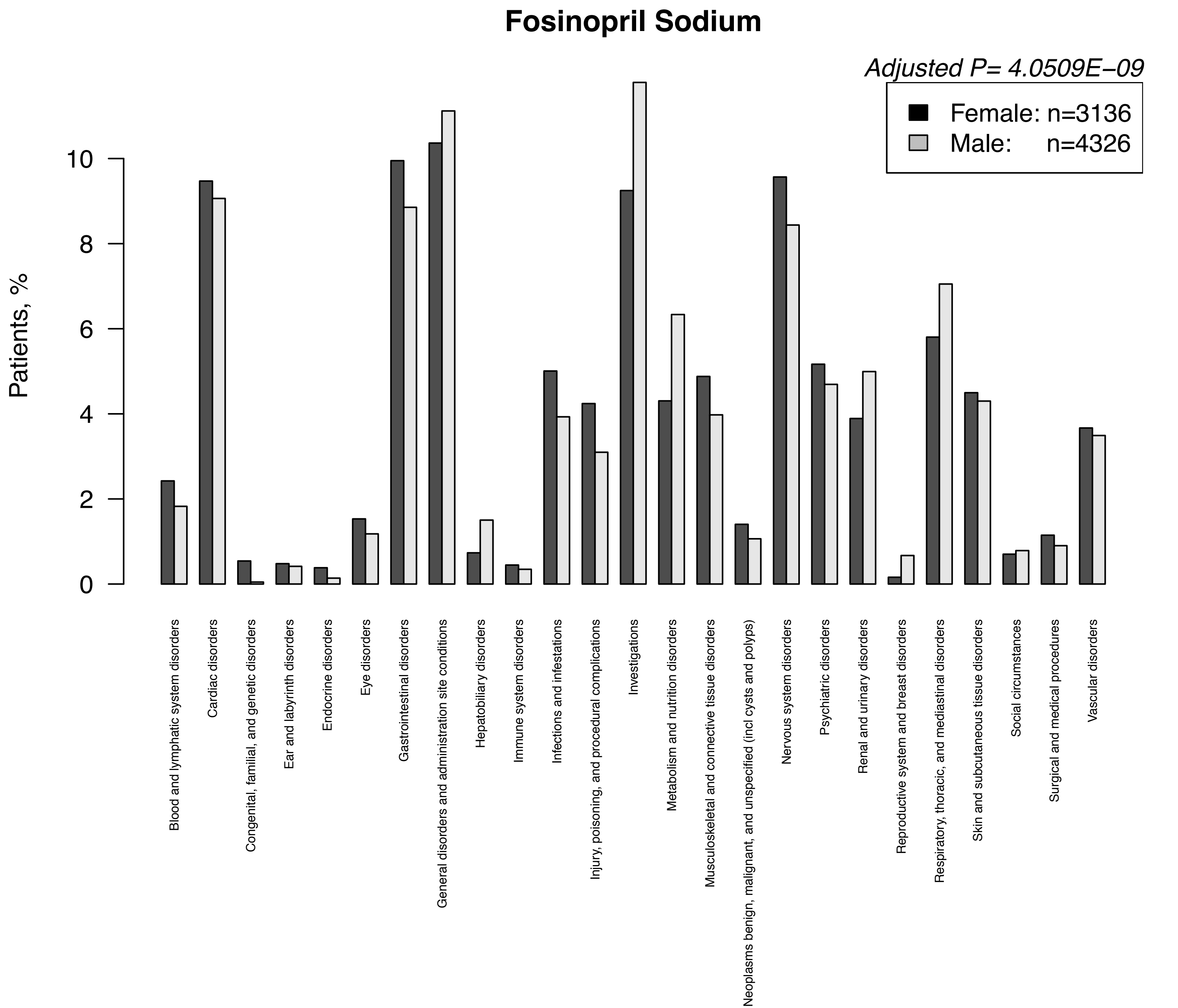

Fosinopril Sodium
Adjusted P= 4.0509E−09
Female: n=3136
Male: n=4326
Patients, %
0
2
4
6
8
10
Blood and lymphatic system disorders
Cardiac disorders
Congenital, familial, and genetic disorders
Ear and labyrinth disorders
Endocrine disorders
Eye disorders
Gastrointestinal disorders
General disorders and administration site conditions
Hepatobiliary disorders
Immune system disorders
Infections and infestations
Injury, poisoning, and procedural complications
Investigations
Metabolism and nutrition disorders
Musculoskeletal and connective tissue disorders
Neoplasms benign, malignant, and unspecified (incl cysts and polyps)
Nervous system disorders
Psychiatric disorders
Renal and urinary disorders
Reproductive system and breast disorders
Respiratory, thoracic, and mediastinal disorders
Skin and subcutaneous tissue disorders
Social circumstances
Surgical and medical procedures
Vascular disorders

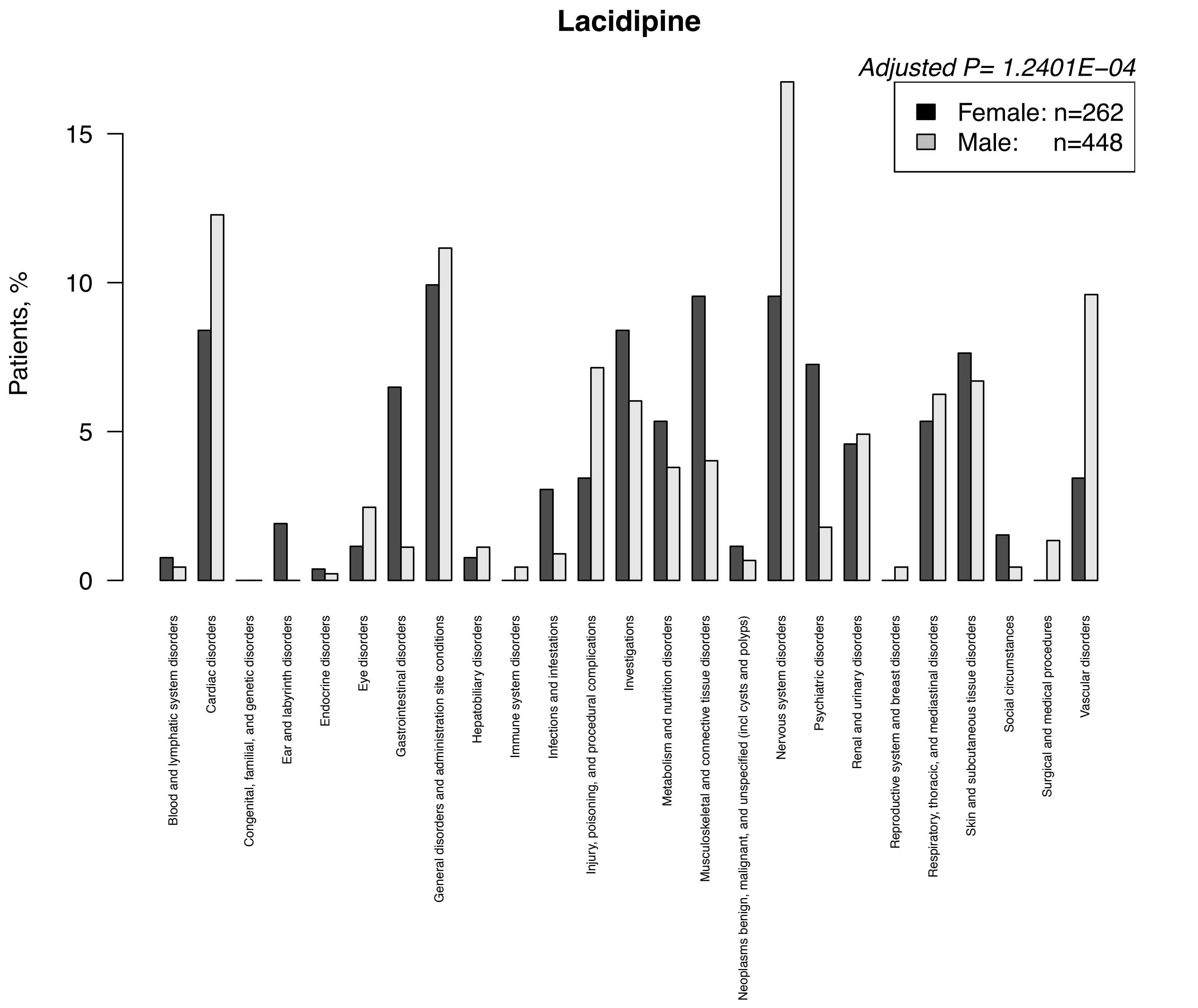
Lacidipine
Adjusted P= 1.2401E−04
Female: n=262
Male: n=448
Patients, %
0
5
10
15
Blood and lymphatic system disorders
Cardiac disorders
Congenital, familial, and genetic disorders
Ear and labyrinth disorders
Endocrine disorders
Eye disorders
Gastrointestinal disorders
General disorders and administration site conditions
Hepatobiliary disorders
Immune system disorders
Infections and infestations
Injury, poisoning, and procedural complications
Investigations
Metabolism and nutrition disorders
Musculoskeletal and connective tissue disorders
Neoplasms benign, malignant, and unspecified (incl cysts and polyps)
Nervous system disorders
Psychiatric disorders
Renal and urinary disorders
Reproductive system and breast disorders
Respiratory, thoracic, and mediastinal disorders
Skin and subcutaneous tissue disorders
Social circumstances
Surgical and medical procedures
Vascular disorders

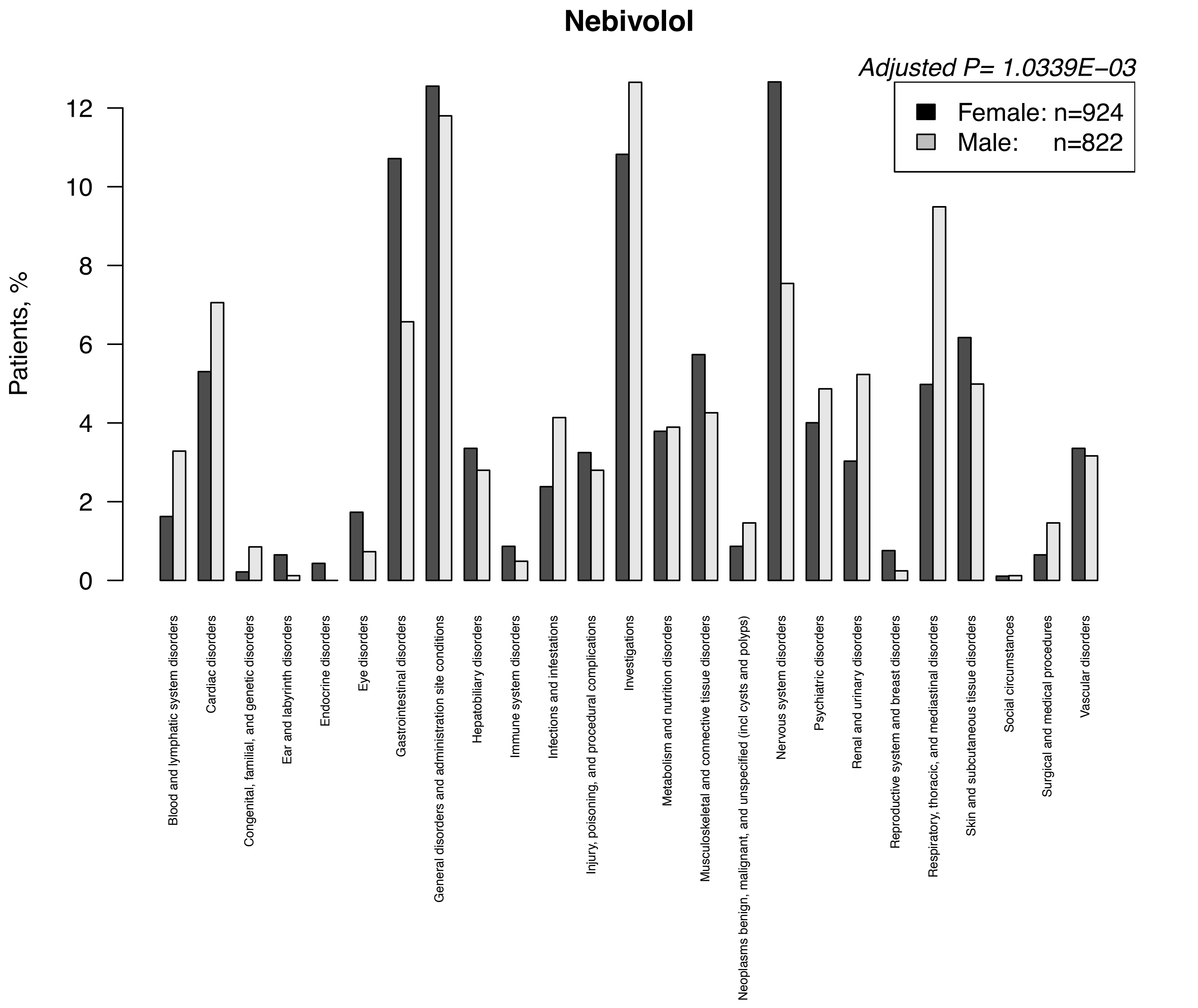
Nebivolol
Adjusted P= 1.0339E−03
Female: n=924
Male: n=822
Patients, %
0
2
4
6
8
10
12
Blood and lymphatic system disorders
Cardiac disorders
Congenital, familial, and genetic disorders
Ear and labyrinth disorders
Endocrine disorders
Eye disorders
Gastrointestinal disorders
General disorders and administration site conditions
Hepatobiliary disorders
Immune system disorders
Infections and infestations
Injury, poisoning, and procedural complications
Investigations
Metabolism and nutrition disorders
Musculoskeletal and connective tissue disorders
Neoplasms benign, malignant, and unspecified (incl cysts and polyps)
Nervous system disorders
Psychiatric disorders
Renal and urinary disorders
Reproductive system and breast disorders
Respiratory, thoracic, and mediastinal disorders
Skin and subcutaneous tissue disorders
Social circumstances
Surgical and medical procedures
Vascular disorders

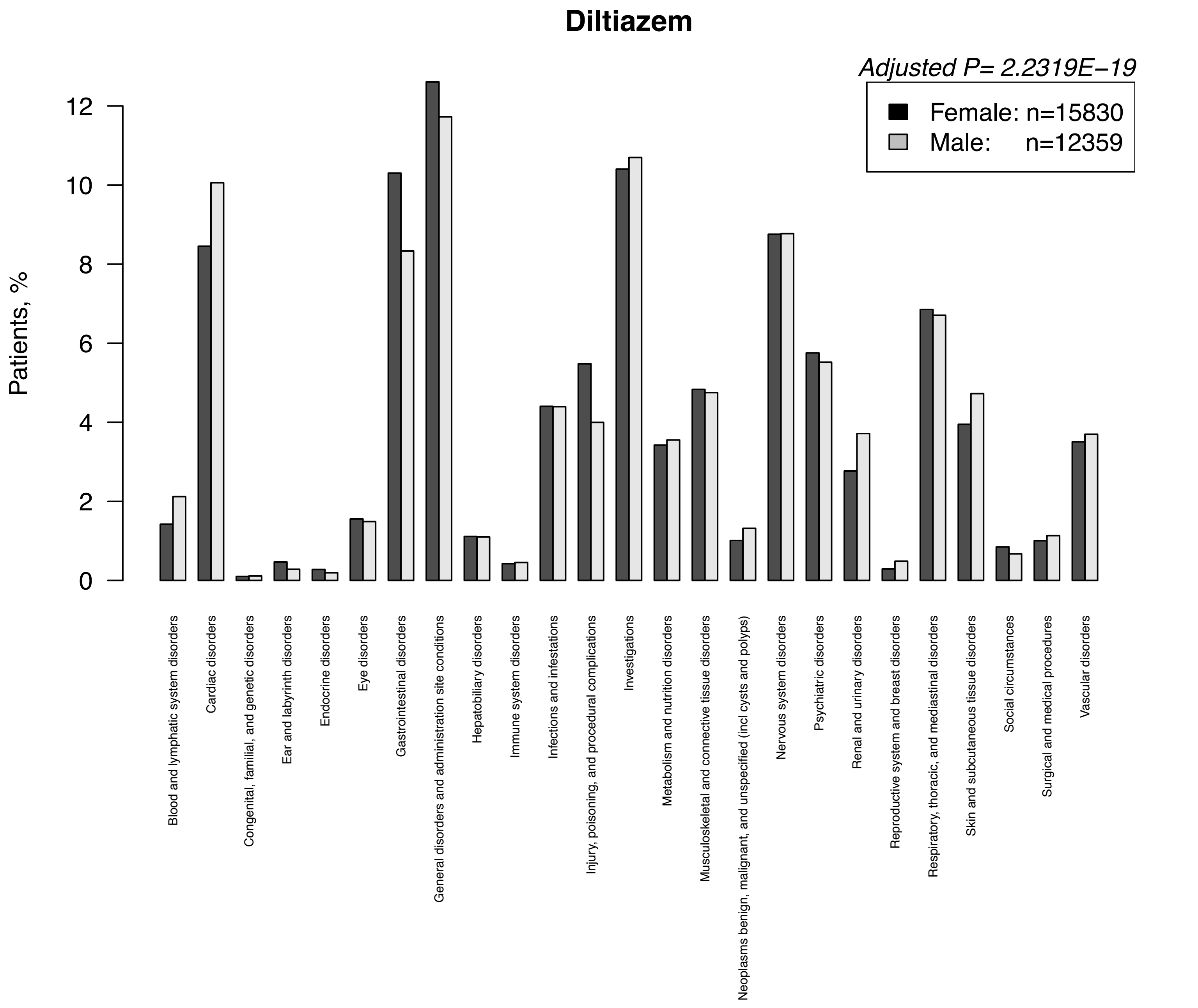

Diltiazem
Adjusted P= 2.2319E−19
Female: n=15830
Male: n=12359
Patients, %
0
2
4
6
8
10
12
Blood and lymphatic system disorders
Cardiac disorders
Congenital, familial, and genetic disorders
Ear and labyrinth disorders
Endocrine disorders
Eye disorders
Gastrointestinal disorders
General disorders and administration site conditions
Hepatobiliary disorders
Immune system disorders
Infections and infestations
Injury, poisoning, and procedural complications
Investigations
Metabolism and nutrition disorders
Musculoskeletal and connective tissue disorders
Neoplasms benign, malignant, and unspecified (incl cysts and polyps)
Nervous system disorders
Psychiatric disorders
Renal and urinary disorders
Reproductive system and breast disorders
Respiratory, thoracic, and mediastinal disorders
Skin and subcutaneous tissue disorders
Social circumstances
Surgical and medical procedures
Vascular disorders

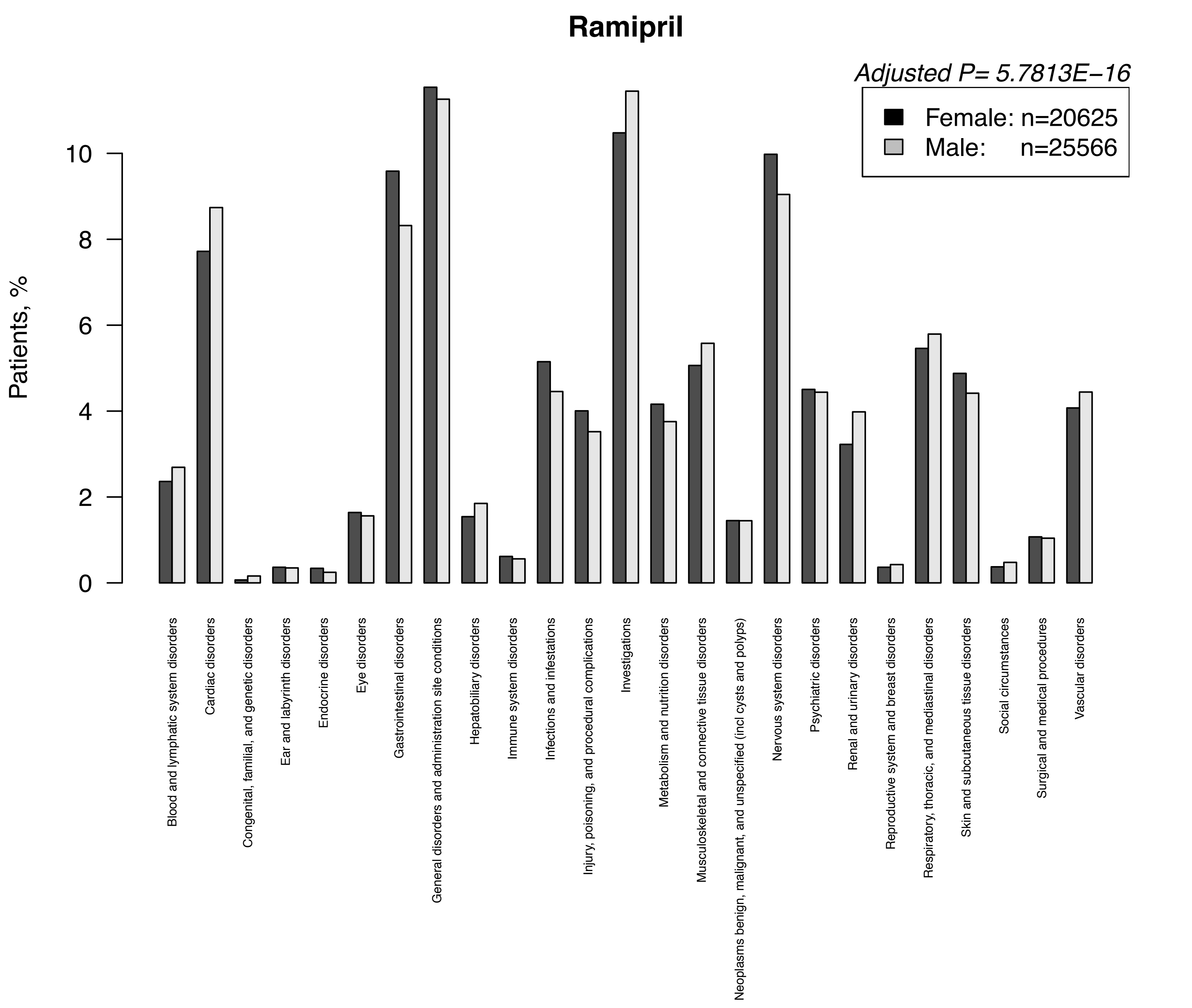
Ramipril
Adjusted P= 5.7813E−16
Female: n=20625
Male: n=25566
Patients, %
0
2
4
6
8
10
Blood and lymphatic system disorders
Cardiac disorders
Congenital, familial, and genetic disorders
Ear and labyrinth disorders
Endocrine disorders
Eye disorders
Gastrointestinal disorders
General disorders and administration site conditions
Hepatobiliary disorders
Immune system disorders
Infections and infestations
Injury, poisoning, and procedural complications
Investigations
Metabolism and nutrition disorders
Musculoskeletal and connective tissue disorders
Neoplasms benign, malignant, and unspecified (incl cysts and polyps)
Nervous system disorders
Psychiatric disorders
Renal and urinary disorders
Reproductive system and breast disorders
Respiratory, thoracic, and mediastinal disorders
Skin and subcutaneous tissue disorders
Social circumstances
Surgical and medical procedures
Vascular disorders

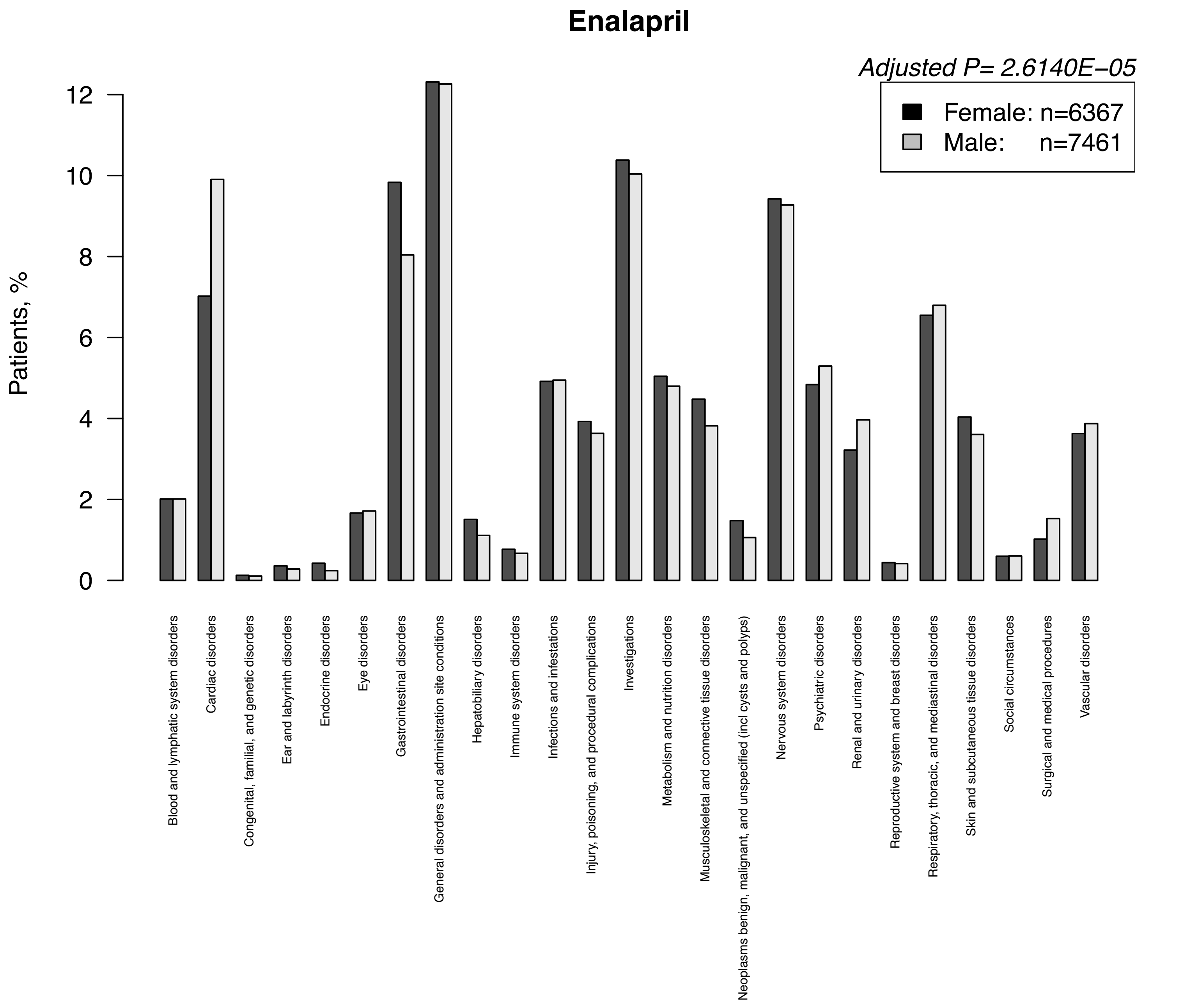

Enalapril
Adjusted P= 2.6140E−05
Female: n=6367
Male: n=7461
Patients, %
0
2
4
6
8
10
12
Blood and lymphatic system disorders
Cardiac disorders
Congenital, familial, and genetic disorders
Ear and labyrinth disorders
Endocrine disorders
Eye disorders
Gastrointestinal disorders
General disorders and administration site conditions
Hepatobiliary disorders
Immune system disorders
Infections and infestations
Injury, poisoning, and procedural complications
Investigations
Metabolism and nutrition disorders
Musculoskeletal and connective tissue disorders
Neoplasms benign, malignant, and unspecified (incl cysts and polyps)
Nervous system disorders
Psychiatric disorders
Renal and urinary disorders
Reproductive system and breast disorders
Respiratory, thoracic, and mediastinal disorders
Skin and subcutaneous tissue disorders
Social circumstances
Surgical and medical procedures
Vascular disorders

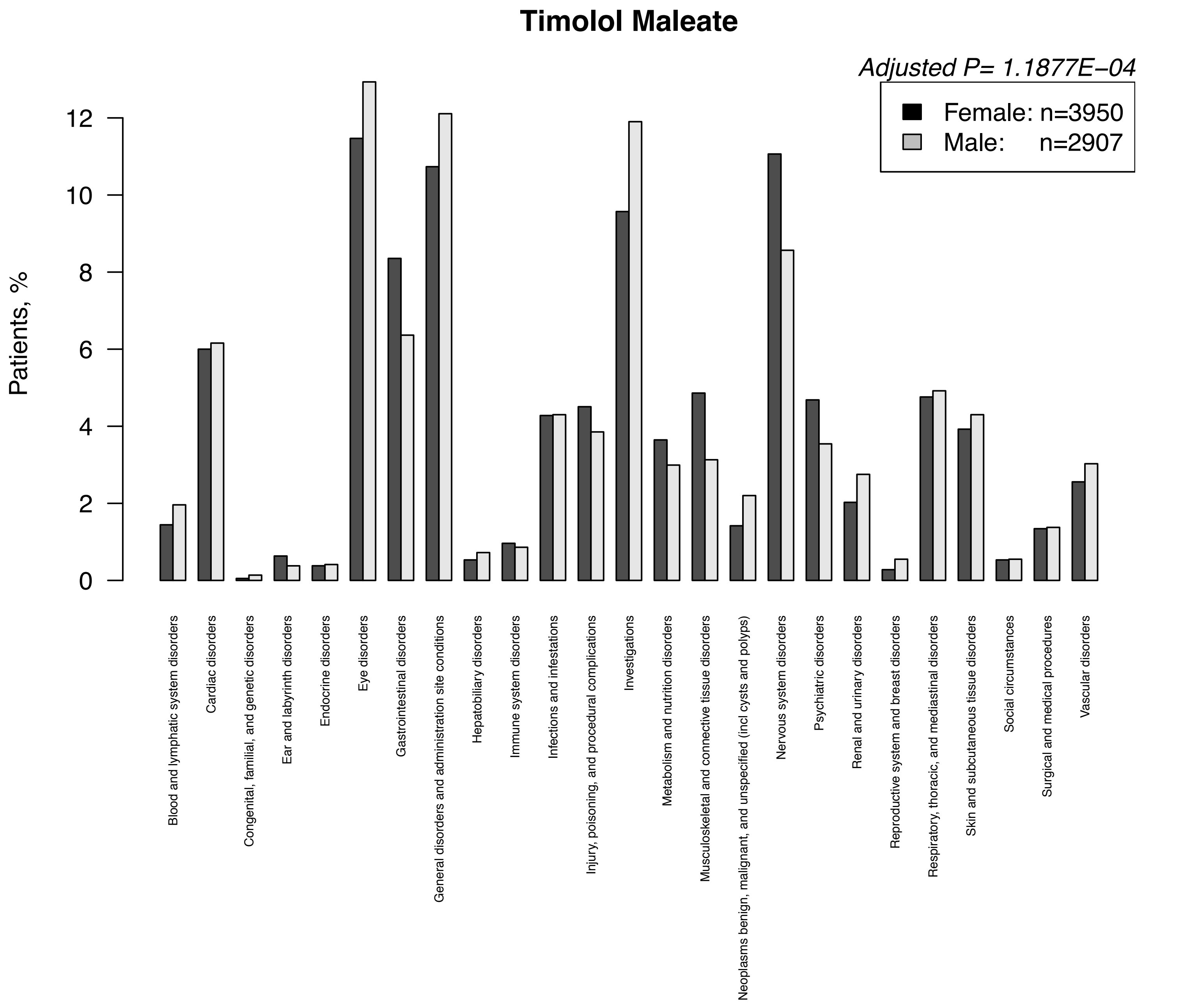
Timolol Maleate
Adjusted P= 1.1877E−04
Female: n=3950
Male: n=2907
Patients, %
0
2
4
6
8
10
12
Blood and lymphatic system disorders
Cardiac disorders
Congenital, familial, and genetic disorders
Ear and labyrinth disorders
Endocrine disorders
Eye disorders
Gastrointestinal disorders
General disorders and administration site conditions
Hepatobiliary disorders
Immune system disorders
Infections and infestations
Injury, poisoning, and procedural complications
Investigations
Metabolism and nutrition disorders
Musculoskeletal and connective tissue disorders
Neoplasms benign, malignant, and unspecified (incl cysts and polyps)
Nervous system disorders
Psychiatric disorders
Renal and urinary disorders
Reproductive system and breast disorders
Respiratory, thoracic, and mediastinal disorders
Skin and subcutaneous tissue disorders
Social circumstances
Surgical and medical procedures
Vascular disorders

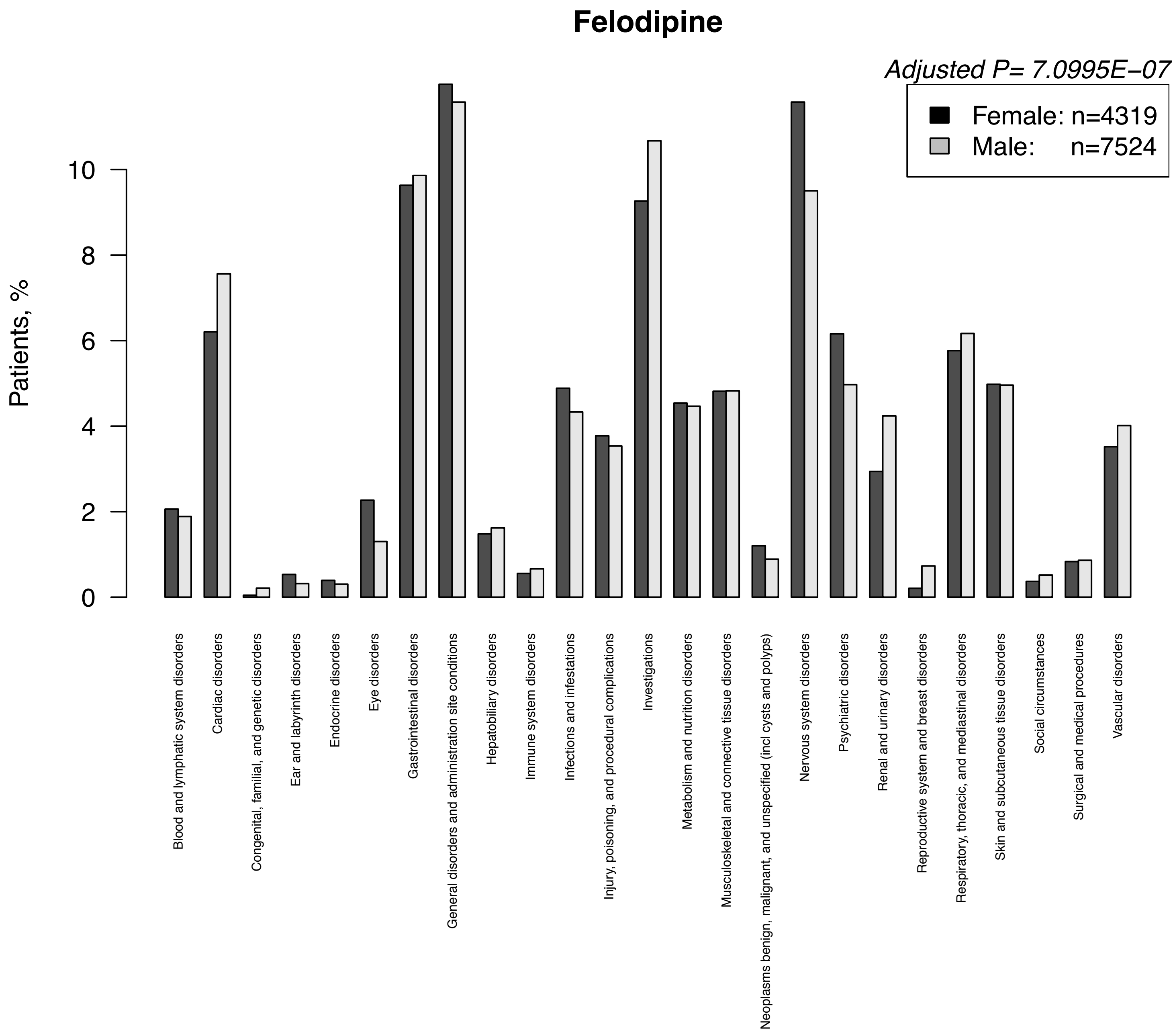

Felodipine
Adjusted P= 7.0995E−07
Female: n=4319
Male: n=7524
Patients, %
0
2
4
6
8
10
Blood and lymphatic system disorders
Cardiac disorders
Congenital, familial, and genetic disorders
Ear and labyrinth disorders
Endocrine disorders
Eye disorders
Gastrointestinal disorders
General disorders and administration site conditions
Hepatobiliary disorders
Immune system disorders
Infections and infestations
Injury, poisoning, and procedural complications
Investigations
Metabolism and nutrition disorders
Musculoskeletal and connective tissue disorders
Neoplasms benign, malignant, and unspecified (incl cysts and polyps)
Nervous system disorders
Psychiatric disorders
Renal and urinary disorders
Reproductive system and breast disorders
Respiratory, thoracic, and mediastinal disorders
Skin and subcutaneous tissue disorders
Social circumstances
Surgical and medical procedures
Vascular disorders

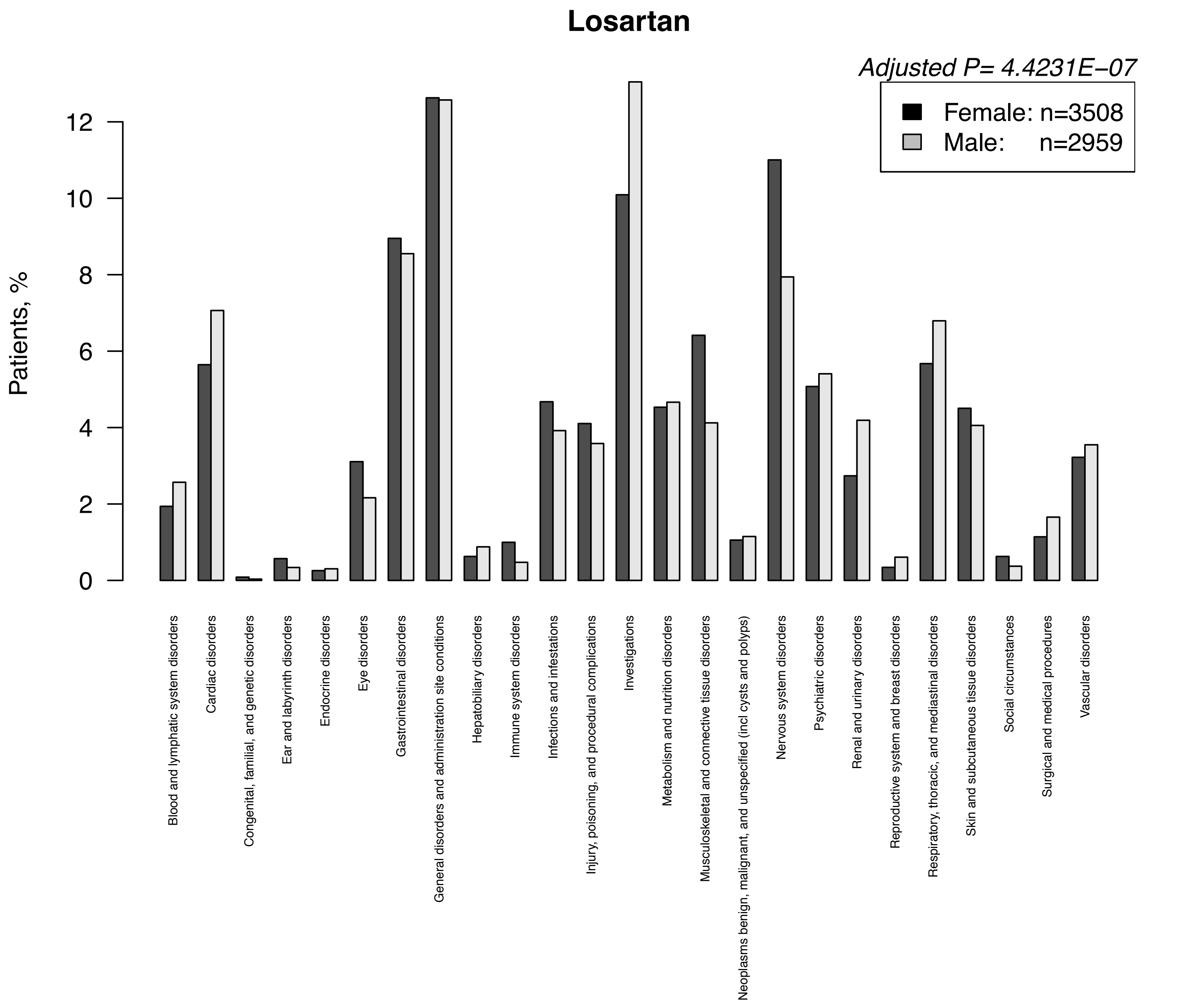
Losartan
Adjusted P= 4.4231E−07
Female: n=3508
Male: n=2959
Patients, %
0
2
4
6
8
10
12
Blood and lymphatic system disorders
Cardiac disorders
Congenital, familial, and genetic disorders
Ear and labyrinth disorders
Endocrine disorders
Eye disorders
Gastrointestinal disorders
General disorders and administration site conditions
Hepatobiliary disorders
Immune system disorders
Infections and infestations
Injury, poisoning, and procedural complications
Investigations
Metabolism and nutrition disorders
Musculoskeletal and connective tissue disorders
Neoplasms benign, malignant, and unspecified (incl cysts and polyps)
Nervous system disorders
Psychiatric disorders
Renal and urinary disorders
Reproductive system and breast disorders
Respiratory, thoracic, and mediastinal disorders
Skin and subcutaneous tissue disorders
Social circumstances
Surgical and medical procedures
Vascular disorders

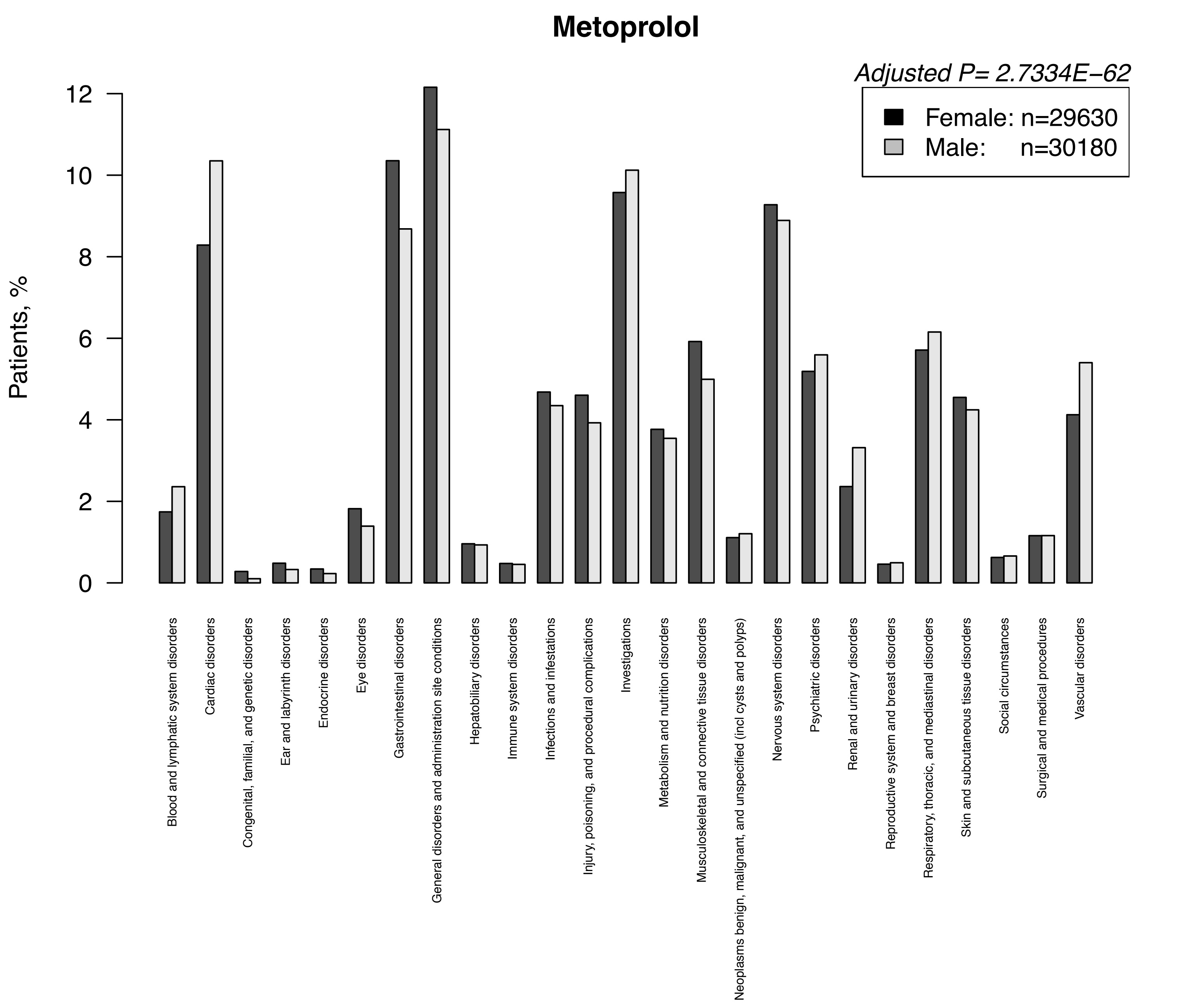

Metoprolol
Adjusted P= 2.7334E−62
Female: n=29630
Male: n=30180
Patients, %
0
2
4
6
8
10
12
Blood and lymphatic system disorders
Cardiac disorders
Congenital, familial, and genetic disorders
Ear and labyrinth disorders
Endocrine disorders
Eye disorders
Gastrointestinal disorders
General disorders and administration site conditions
Hepatobiliary disorders
Immune system disorders
Infections and infestations
Injury, poisoning, and procedural complications
Investigations
Metabolism and nutrition disorders
Musculoskeletal and connective tissue disorders
Neoplasms benign, malignant, and unspecified (incl cysts and polyps)
Nervous system disorders
Psychiatric disorders
Renal and urinary disorders
Reproductive system and breast disorders
Respiratory, thoracic, and mediastinal disorders
Skin and subcutaneous tissue disorders
Social circumstances
Surgical and medical procedures
Vascular disorders

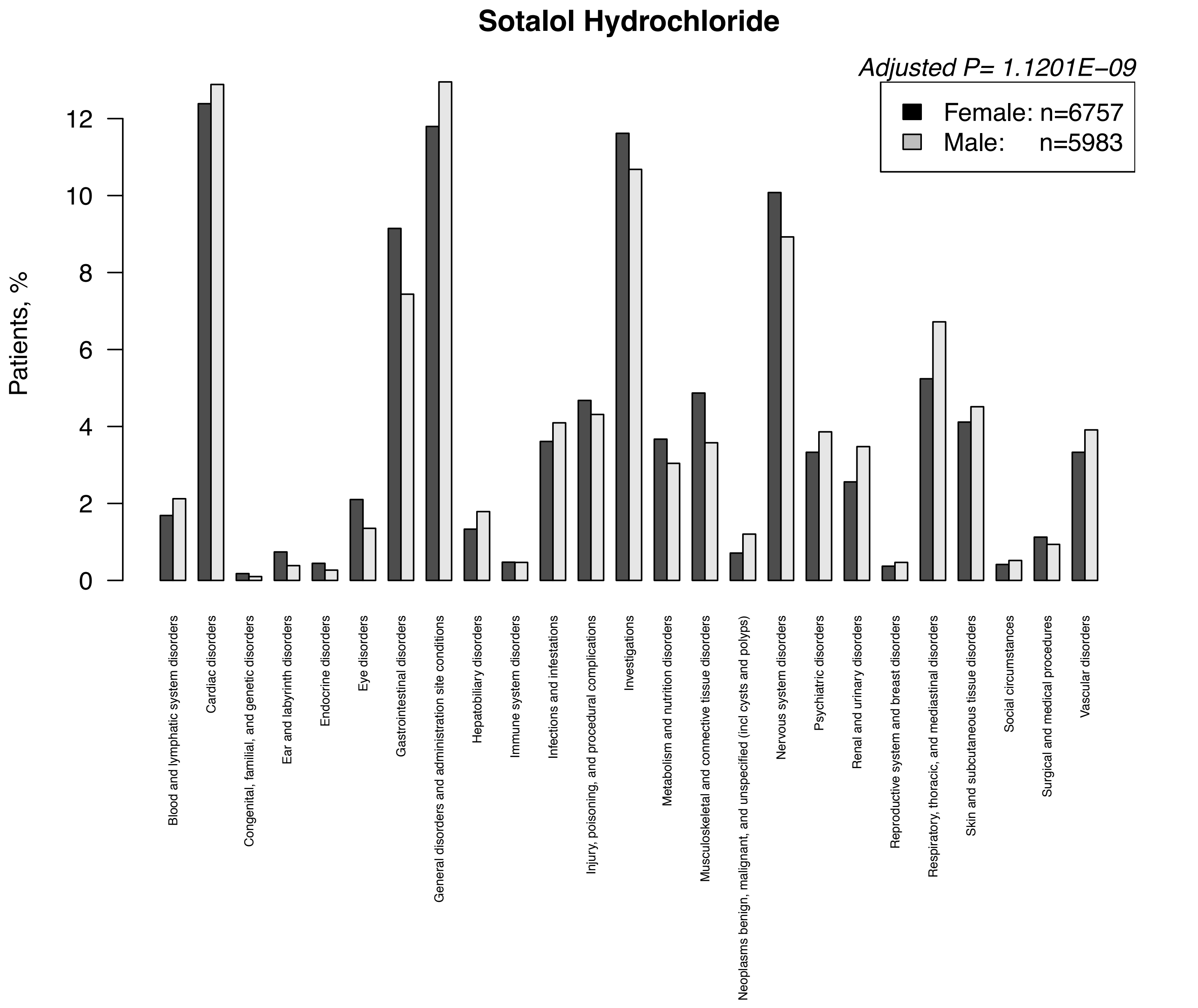

Sotalol Hydrochloride
Adjusted P= 1.1201E−09
Female: n=6757
Male: n=5983
Patients, %
0
2
4
6
8
10
12
Blood and lymphatic system disorders
Cardiac disorders
Congenital, familial, and genetic disorders
Ear and labyrinth disorders
Endocrine disorders
Eye disorders
Gastrointestinal disorders
General disorders and administration site conditions
Hepatobiliary disorders
Immune system disorders
Infections and infestations
Injury, poisoning, and procedural complications
Investigations
Metabolism and nutrition disorders
Musculoskeletal and connective tissue disorders
Neoplasms benign, malignant, and unspecified (incl cysts and polyps)
Nervous system disorders
Psychiatric disorders
Renal and urinary disorders
Reproductive system and breast disorders
Respiratory, thoracic, and mediastinal disorders
Skin and subcutaneous tissue disorders
Social circumstances
Surgical and medical procedures
Vascular disorders

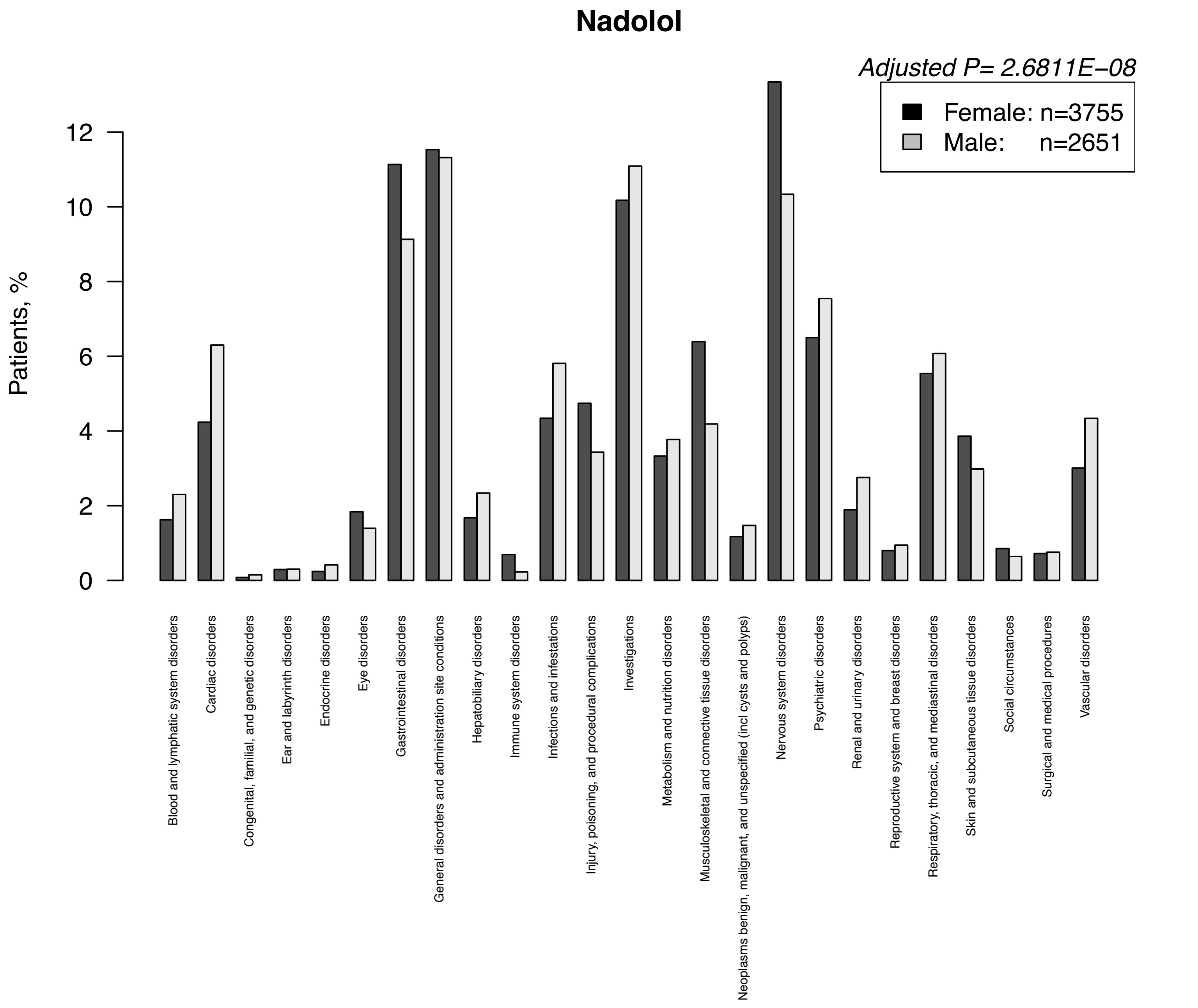

Nadolol
Adjusted P= 2.6811E−08
Female: n=3755
Male: n=2651
Patients, %
0
2
4
6
8
10
12
Blood and lymphatic system disorders
Cardiac disorders
Congenital, familial, and genetic disorders
Ear and labyrinth disorders
Endocrine disorders
Eye disorders
Gastrointestinal disorders
General disorders and administration site conditions
Hepatobiliary disorders
Immune system disorders
Infections and infestations
Injury, poisoning, and procedural complications
Investigations
Metabolism and nutrition disorders
Musculoskeletal and connective tissue disorders
Neoplasms benign, malignant, and unspecified (incl cysts and polyps)
Nervous system disorders
Psychiatric disorders
Renal and urinary disorders
Reproductive system and breast disorders
Respiratory, thoracic, and mediastinal disorders
Skin and subcutaneous tissue disorders
Social circumstances
Surgical and medical procedures
Vascular disorders

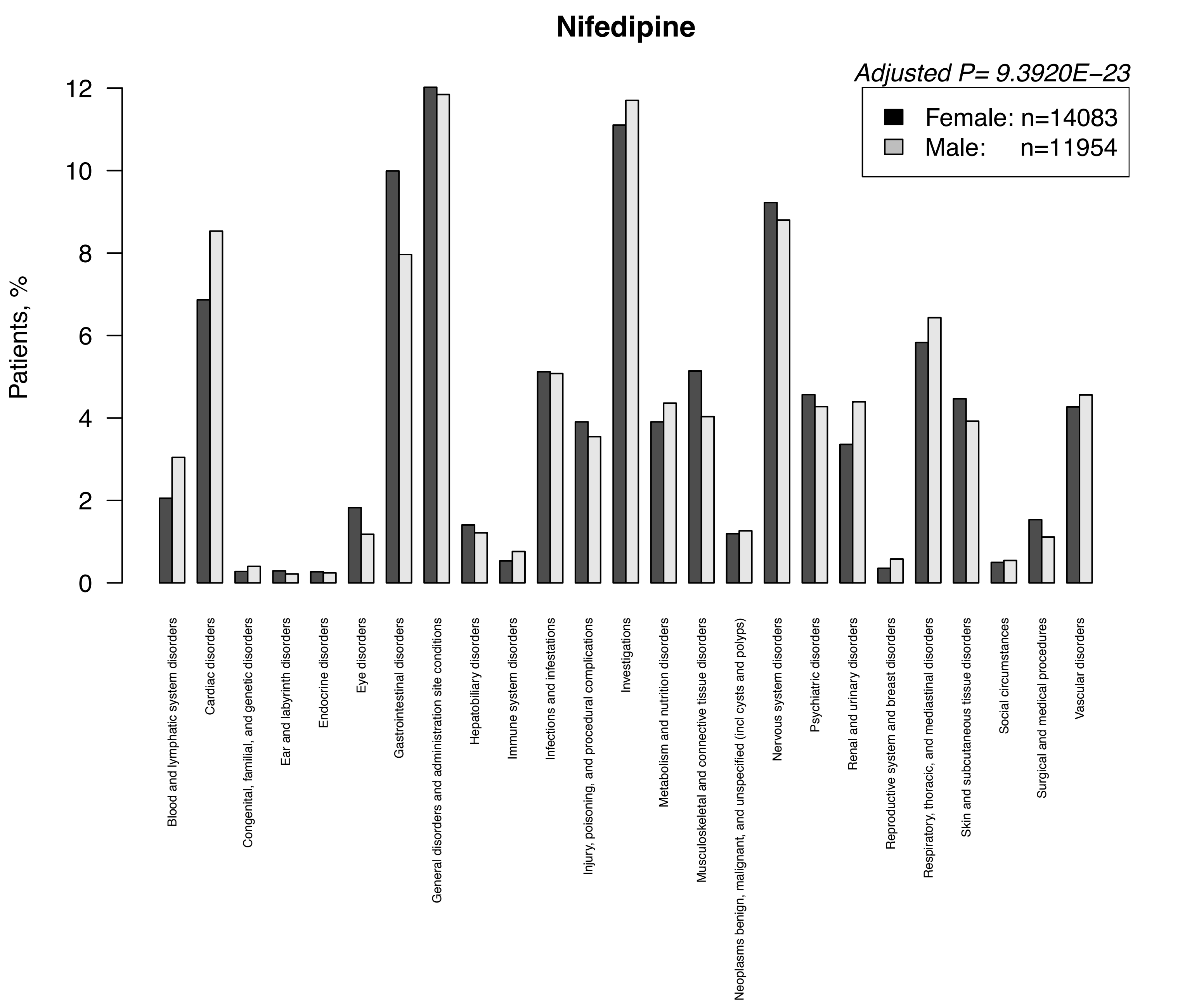

Nifedipine
Adjusted P= 9.3920E−23
Female: n=14083
Male: n=11954
Patients, %
0
2
4
6
8
10
12
Blood and lymphatic system disorders
Cardiac disorders
Congenital, familial, and genetic disorders
Ear and labyrinth disorders
Endocrine disorders
Eye disorders
Gastrointestinal disorders
General disorders and administration site conditions
Hepatobiliary disorders
Immune system disorders
Infections and infestations
Injury, poisoning, and procedural complications
Investigations
Metabolism and nutrition disorders
Musculoskeletal and connective tissue disorders
Neoplasms benign, malignant, and unspecified (incl cysts and polyps)
Nervous system disorders
Psychiatric disorders
Renal and urinary disorders
Reproductive system and breast disorders
Respiratory, thoracic, and mediastinal disorders
Skin and subcutaneous tissue disorders
Social circumstances
Surgical and medical procedures
Vascular disorders

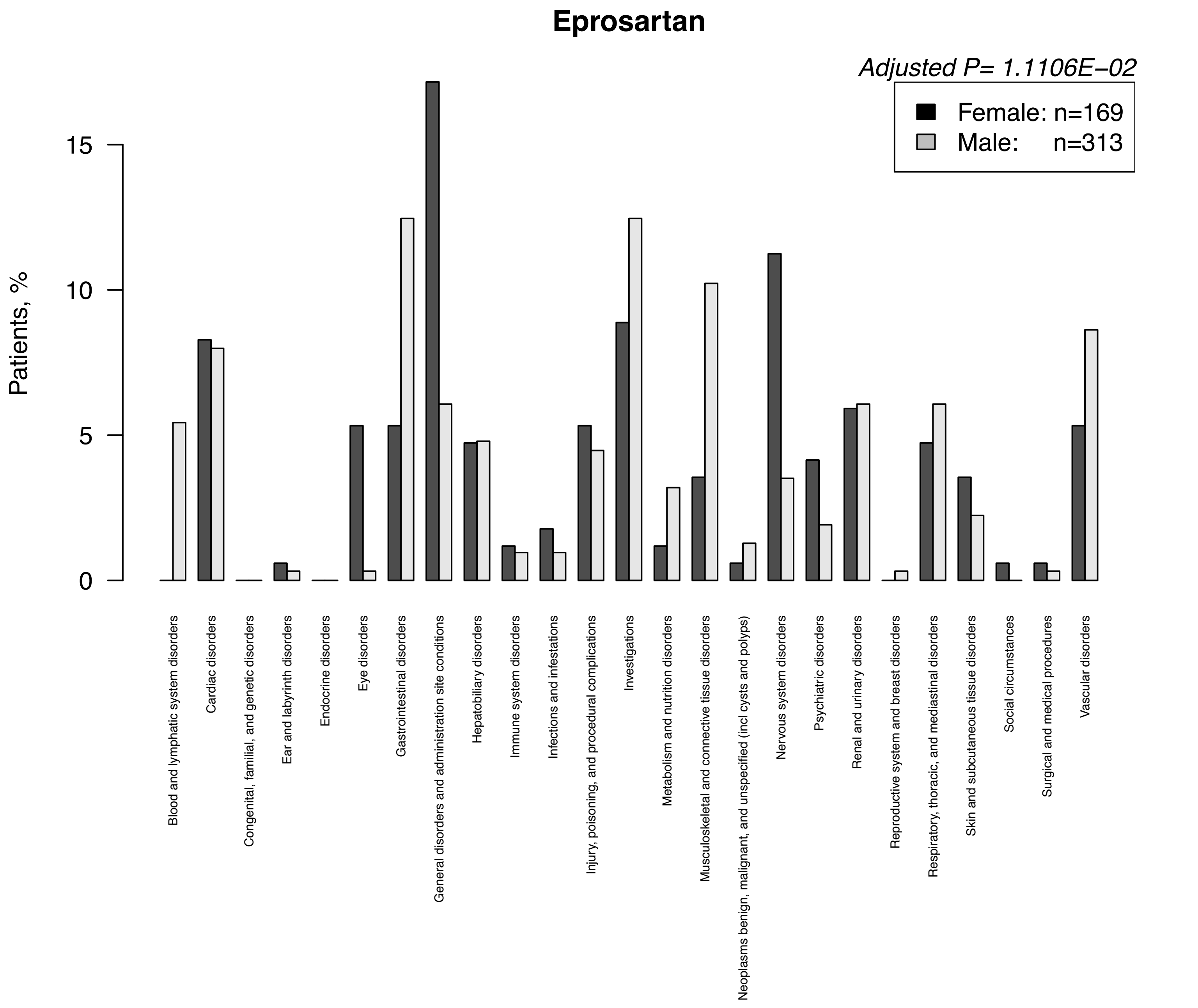

Eprosartan
Adjusted P= 1.1106E−02
Female: n=169
Male: n=313
Patients, %
0
5
10
15
Blood and lymphatic system disorders
Cardiac disorders
Congenital, familial, and genetic disorders
Ear and labyrinth disorders
Endocrine disorders
Eye disorders
Gastrointestinal disorders
General disorders and administration site conditions
Hepatobiliary disorders
Immune system disorders
Infections and infestations
Injury, poisoning, and procedural complications
Investigations
Metabolism and nutrition disorders
Musculoskeletal and connective tissue disorders
Neoplasms benign, malignant, and unspecified (incl cysts and polyps)
Nervous system disorders
Psychiatric disorders
Renal and urinary disorders
Reproductive system and breast disorders
Respiratory, thoracic, and mediastinal disorders
Skin and subcutaneous tissue disorders
Social circumstances
Surgical and medical procedures
Vascular disorders

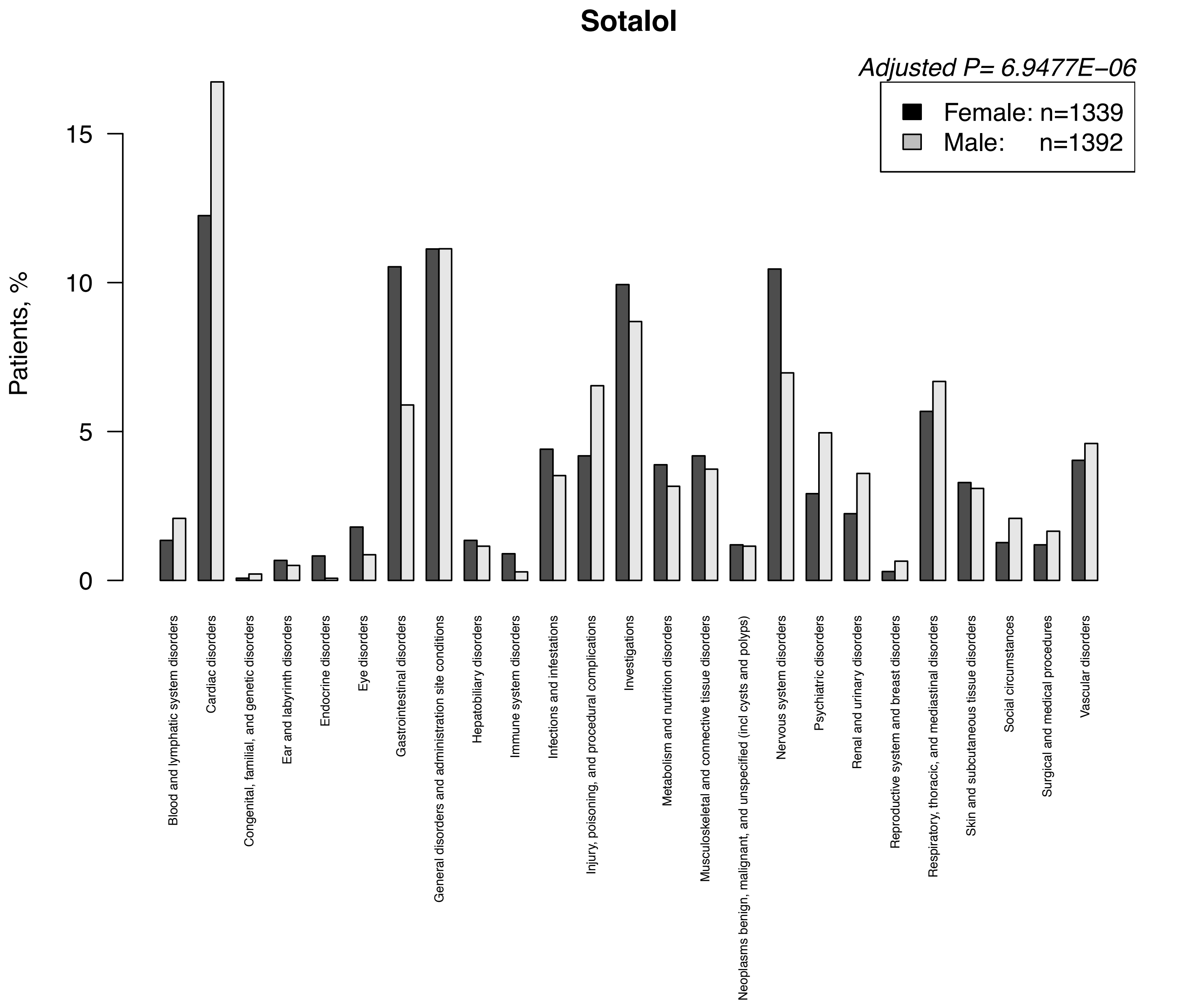
Sotalol
Adjusted P= 6.9477E−06
Female: n=1339
Male: n=1392
Patients, %
0
5
10
15
Blood and lymphatic system disorders
Cardiac disorders
Congenital, familial, and genetic disorders
Ear and labyrinth disorders
Endocrine disorders
Eye disorders
Gastrointestinal disorders
General disorders and administration site conditions
Hepatobiliary disorders
Immune system disorders
Infections and infestations
Injury, poisoning, and procedural complications
Investigations
Metabolism and nutrition disorders
Musculoskeletal and connective tissue disorders
Neoplasms benign, malignant, and unspecified (incl cysts and polyps)
Nervous system disorders
Psychiatric disorders
Renal and urinary disorders
Reproductive system and breast disorders
Respiratory, thoracic, and mediastinal disorders
Skin and subcutaneous tissue disorders
Social circumstances
Surgical and medical procedures
Vascular disorders

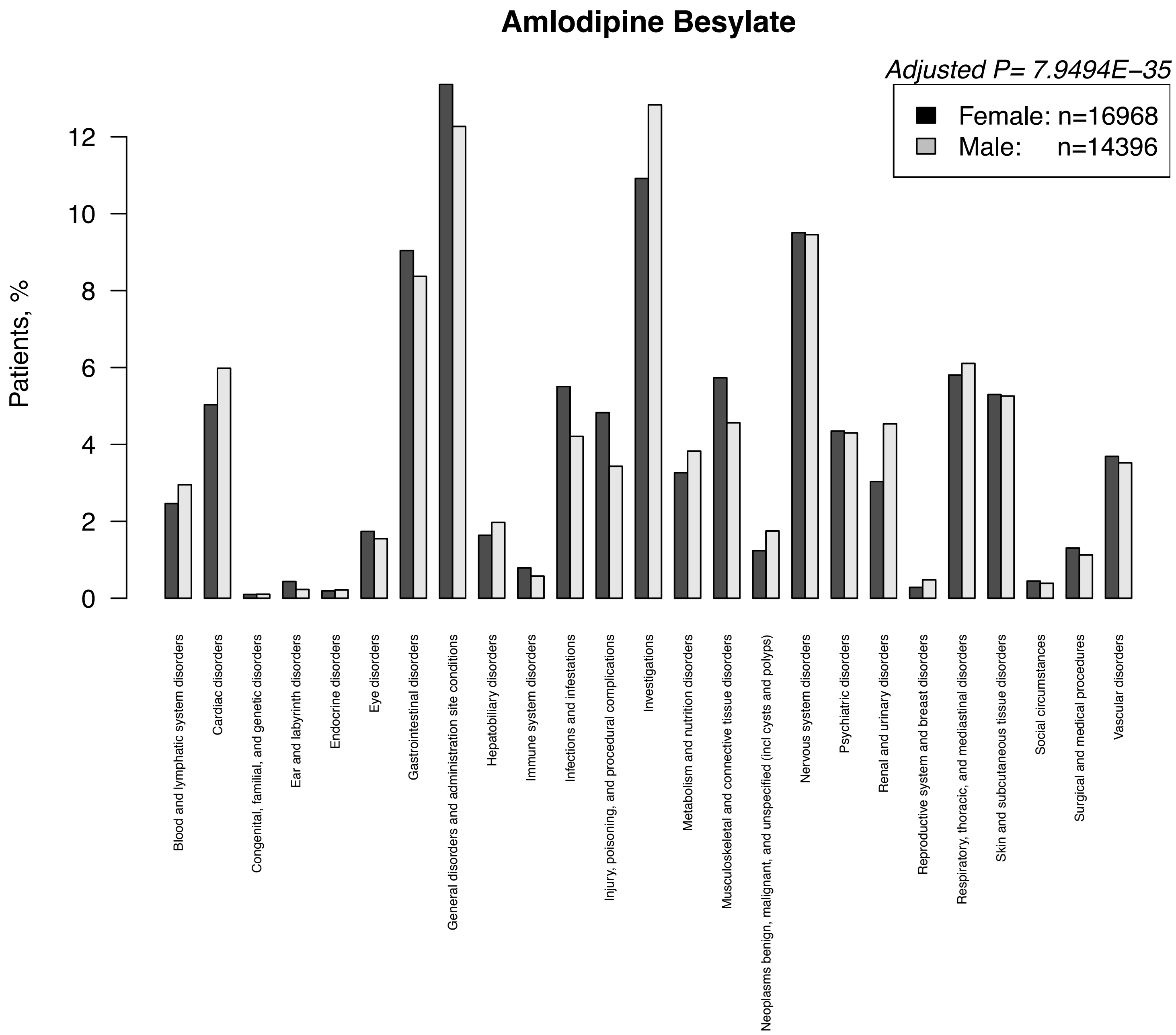

Amlodipine Besylate
Adjusted P= 7.9494E−35
Female: n=16968
Male: n=14396
Patients, %
0
2
4
6
8
10
12
Blood and lymphatic system disorders
Cardiac disorders
Congenital, familial, and genetic disorders
Ear and labyrinth disorders
Endocrine disorders
Eye disorders
Gastrointestinal disorders
General disorders and administration site conditions
Hepatobiliary disorders
Immune system disorders
Infections and infestations
Injury, poisoning, and procedural complications
Investigations
Metabolism and nutrition disorders
Musculoskeletal and connective tissue disorders
Neoplasms benign, malignant, and unspecified (incl cysts and polyps)
Nervous system disorders
Psychiatric disorders
Renal and urinary disorders
Reproductive system and breast disorders
Respiratory, thoracic, and mediastinal disorders
Skin and subcutaneous tissue disorders
Social circumstances
Surgical and medical procedures
Vascular disorders

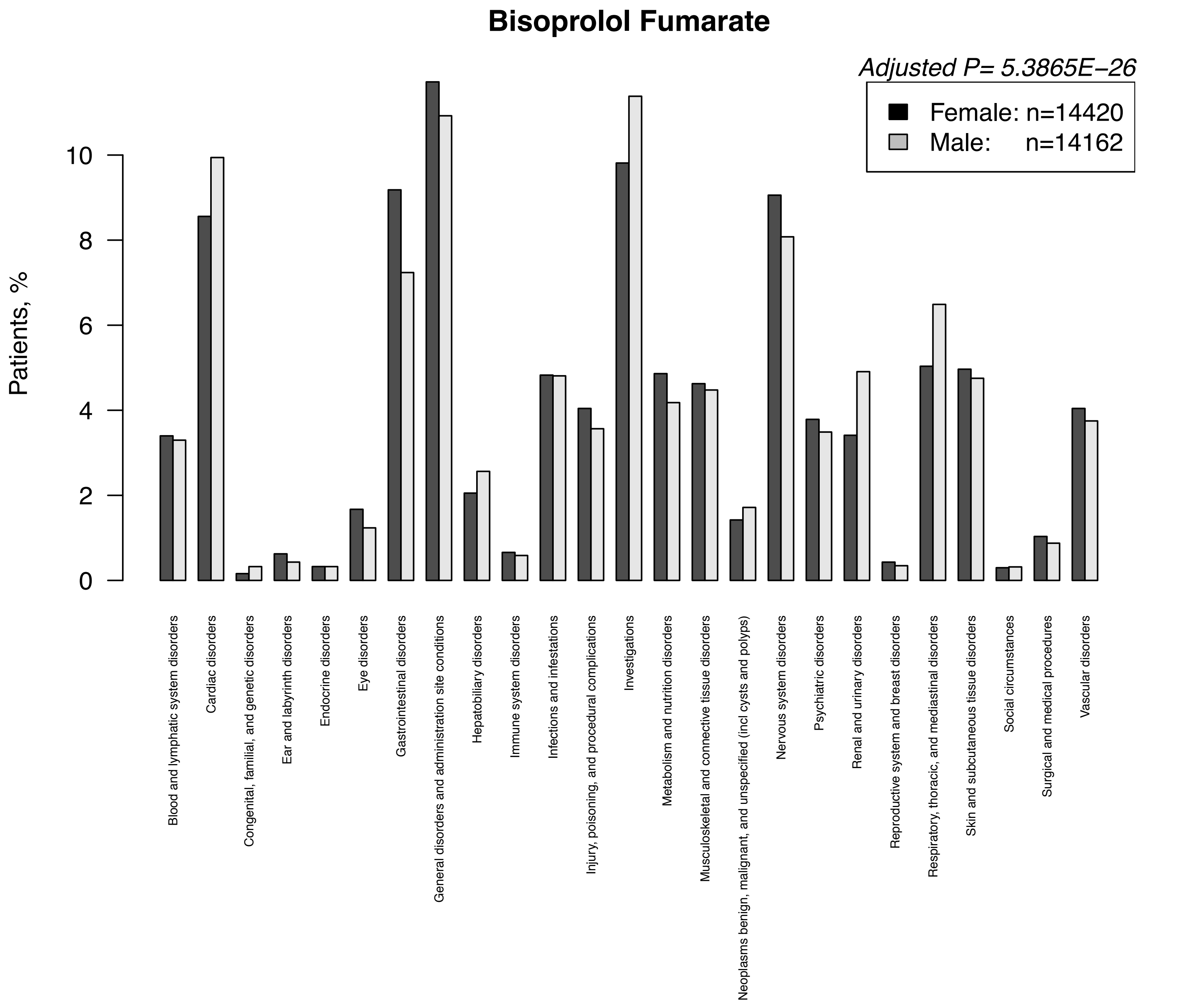

Bisoprolol Fumarate
Adjusted P= 5.3865E−26
Female: n=14420
Male: n=14162
Patients, %
0
2
4
6
8
10
Blood and lymphatic system disorders
Cardiac disorders
Congenital, familial, and genetic disorders
Ear and labyrinth disorders
Endocrine disorders
Eye disorders
Gastrointestinal disorders
General disorders and administration site conditions
Hepatobiliary disorders
Immune system disorders
Infections and infestations
Injury, poisoning, and procedural complications
Investigations
Metabolism and nutrition disorders
Musculoskeletal and connective tissue disorders
Neoplasms benign, malignant, and unspecified (incl cysts and polyps)
Nervous system disorders
Psychiatric disorders
Renal and urinary disorders
Reproductive system and breast disorders
Respiratory, thoracic, and mediastinal disorders
Skin and subcutaneous tissue disorders
Social circumstances
Surgical and medical procedures
Vascular disorders

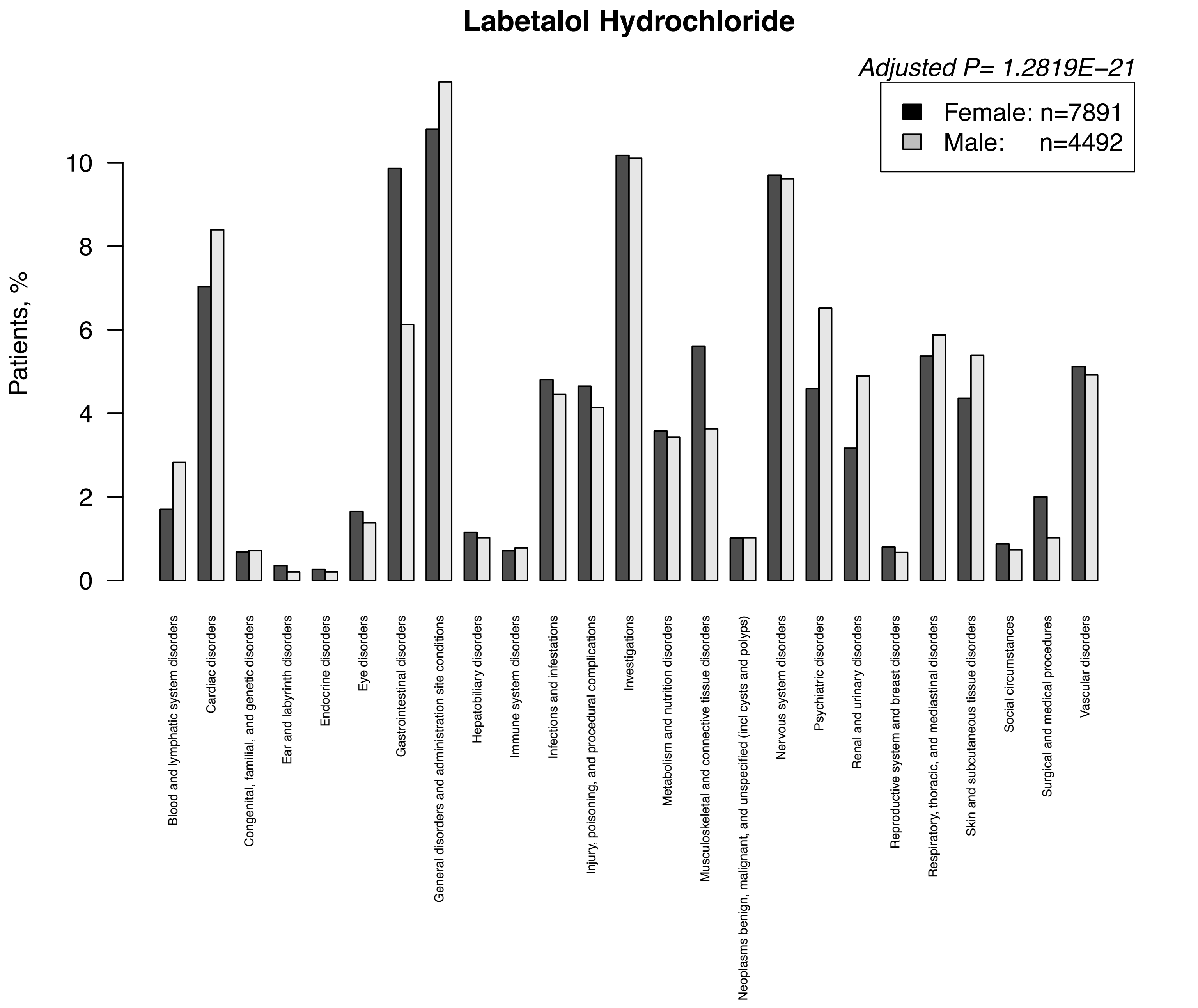
Labetalol Hydrochloride
Adjusted P= 1.2819E−21
Female: n=7891
Male: n=4492
Patients, %
0
2
4
6
8
10
Blood and lymphatic system disorders
Cardiac disorders
Congenital, familial, and genetic disorders
Ear and labyrinth disorders
Endocrine disorders
Eye disorders
Gastrointestinal disorders
General disorders and administration site conditions
Hepatobiliary disorders
Immune system disorders
Infections and infestations
Injury, poisoning, and procedural complications
Investigations
Metabolism and nutrition disorders
Musculoskeletal and connective tissue disorders
Neoplasms benign, malignant, and unspecified (incl cysts and polyps)
Nervous system disorders
Psychiatric disorders
Renal and urinary disorders
Reproductive system and breast disorders
Respiratory, thoracic, and mediastinal disorders
Skin and subcutaneous tissue disorders
Social circumstances
Surgical and medical procedures
Vascular disorders

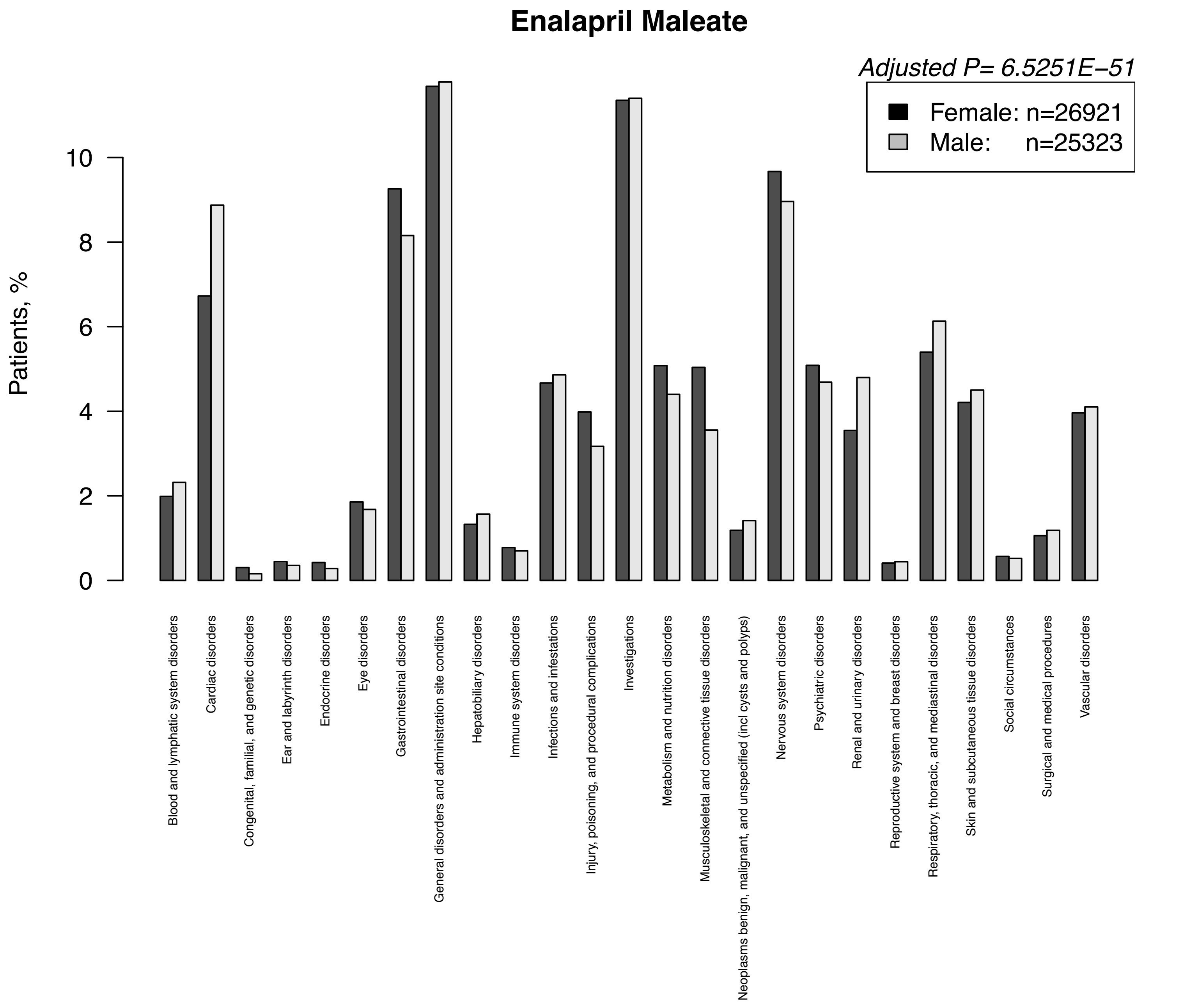
Enalapril Maleate
Adjusted P= 6.5251E−51
Female: n=26921
Male: n=25323
Patients, %
0
2
4
6
8
10
Blood and lymphatic system disorders
Cardiac disorders
Congenital, familial, and genetic disorders
Ear and labyrinth disorders
Endocrine disorders
Eye disorders
Gastrointestinal disorders
General disorders and administration site conditions
Hepatobiliary disorders
Immune system disorders
Infections and infestations
Injury, poisoning, and procedural complications
Investigations
Metabolism and nutrition disorders
Musculoskeletal and connective tissue disorders
Neoplasms benign, malignant, and unspecified (incl cysts and polyps)
Nervous system disorders
Psychiatric disorders
Renal and urinary disorders
Reproductive system and breast disorders
Respiratory, thoracic, and mediastinal disorders
Skin and subcutaneous tissue disorders
Social circumstances
Surgical and medical procedures
Vascular disorders

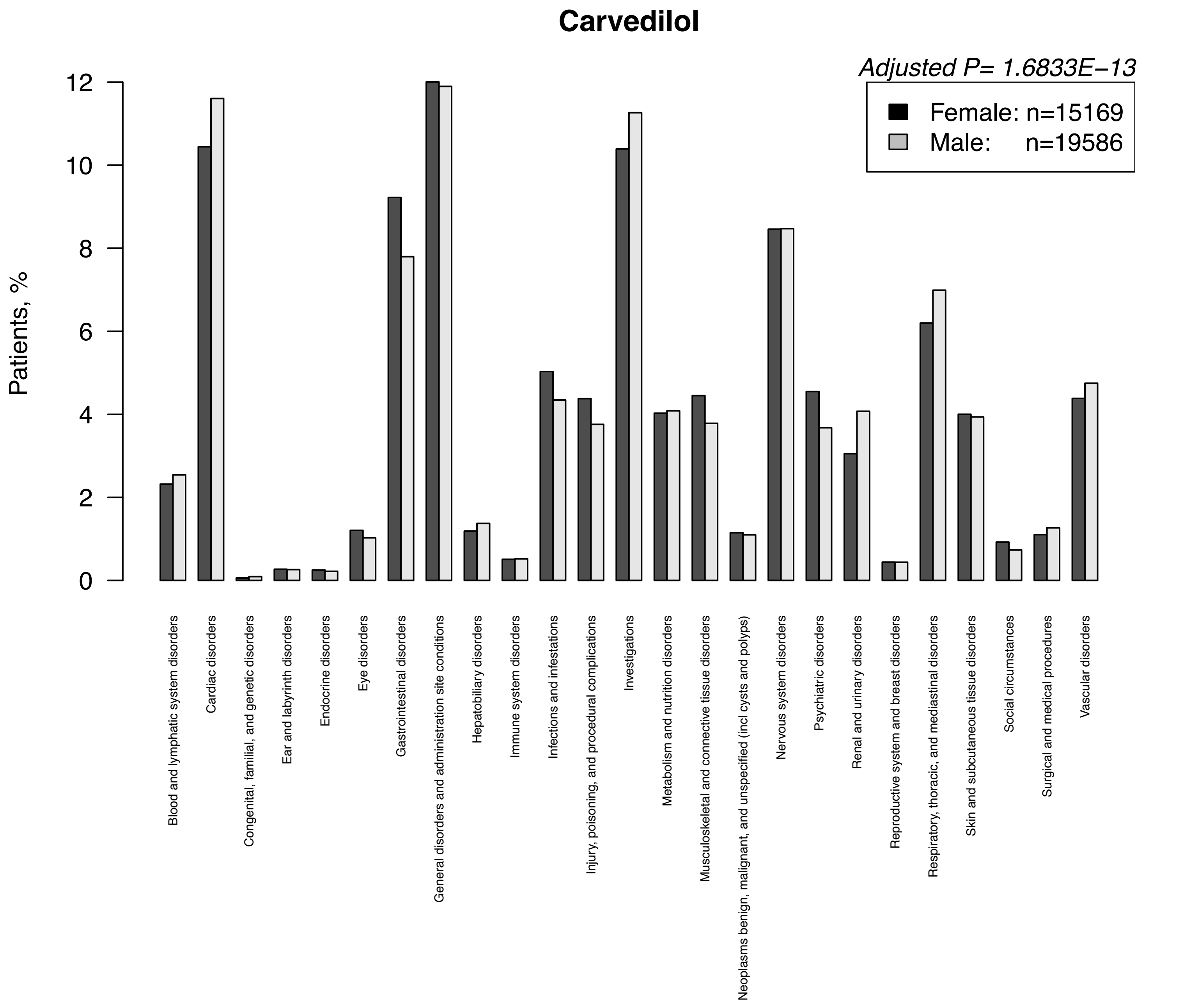
Carvedilol
Adjusted P= 1.6833E−13
Female: n=15169
Male: n=19586
Patients, %
0
2
4
6
8
10
12
Blood and lymphatic system disorders
Cardiac disorders
Congenital, familial, and genetic disorders
Ear and labyrinth disorders
Endocrine disorders
Eye disorders
Gastrointestinal disorders
General disorders and administration site conditions
Hepatobiliary disorders
Immune system disorders
Infections and infestations
Injury, poisoning, and procedural complications
Investigations
Metabolism and nutrition disorders
Musculoskeletal and connective tissue disorders
Neoplasms benign, malignant, and unspecified (incl cysts and polyps)
Nervous system disorders
Psychiatric disorders
Renal and urinary disorders
Reproductive system and breast disorders
Respiratory, thoracic, and mediastinal disorders
Skin and subcutaneous tissue disorders
Social circumstances
Surgical and medical procedures
Vascular disorders

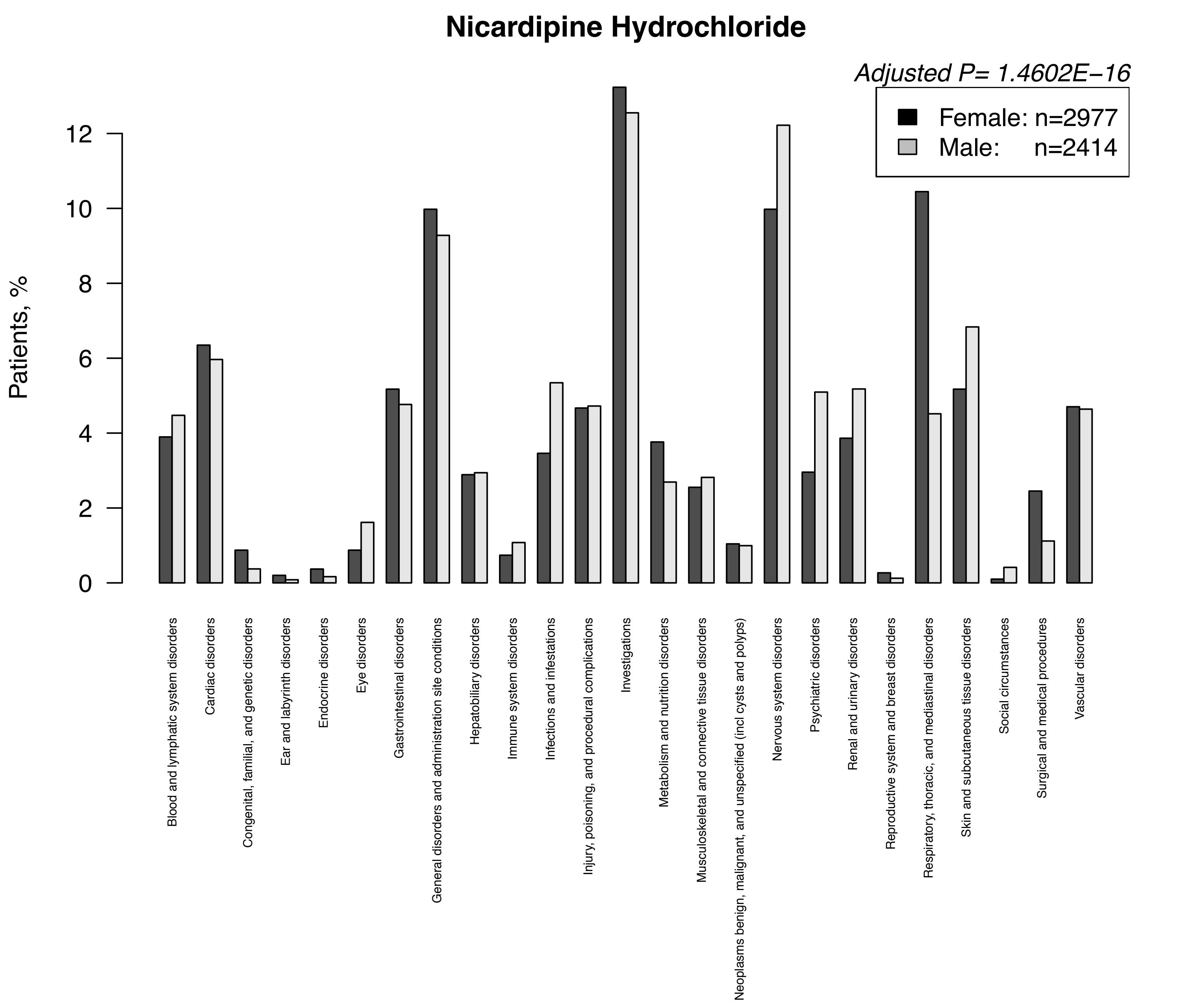
Nicardipine Hydrochloride
Adjusted P= 1.4602E−16
Female: n=2977
Male: n=2414
Patients, %
0
2
4
6
8
10
12
Blood and lymphatic system disorders
Cardiac disorders
Congenital, familial, and genetic disorders
Ear and labyrinth disorders
Endocrine disorders
Eye disorders
Gastrointestinal disorders
General disorders and administration site conditions
Hepatobiliary disorders
Immune system disorders
Infections and infestations
Injury, poisoning, and procedural complications
Investigations
Metabolism and nutrition disorders
Musculoskeletal and connective tissue disorders
Neoplasms benign, malignant, and unspecified (incl cysts and polyps)
Nervous system disorders
Psychiatric disorders
Renal and urinary disorders
Reproductive system and breast disorders
Respiratory, thoracic, and mediastinal disorders
Skin and subcutaneous tissue disorders
Social circumstances
Surgical and medical procedures
Vascular disorders

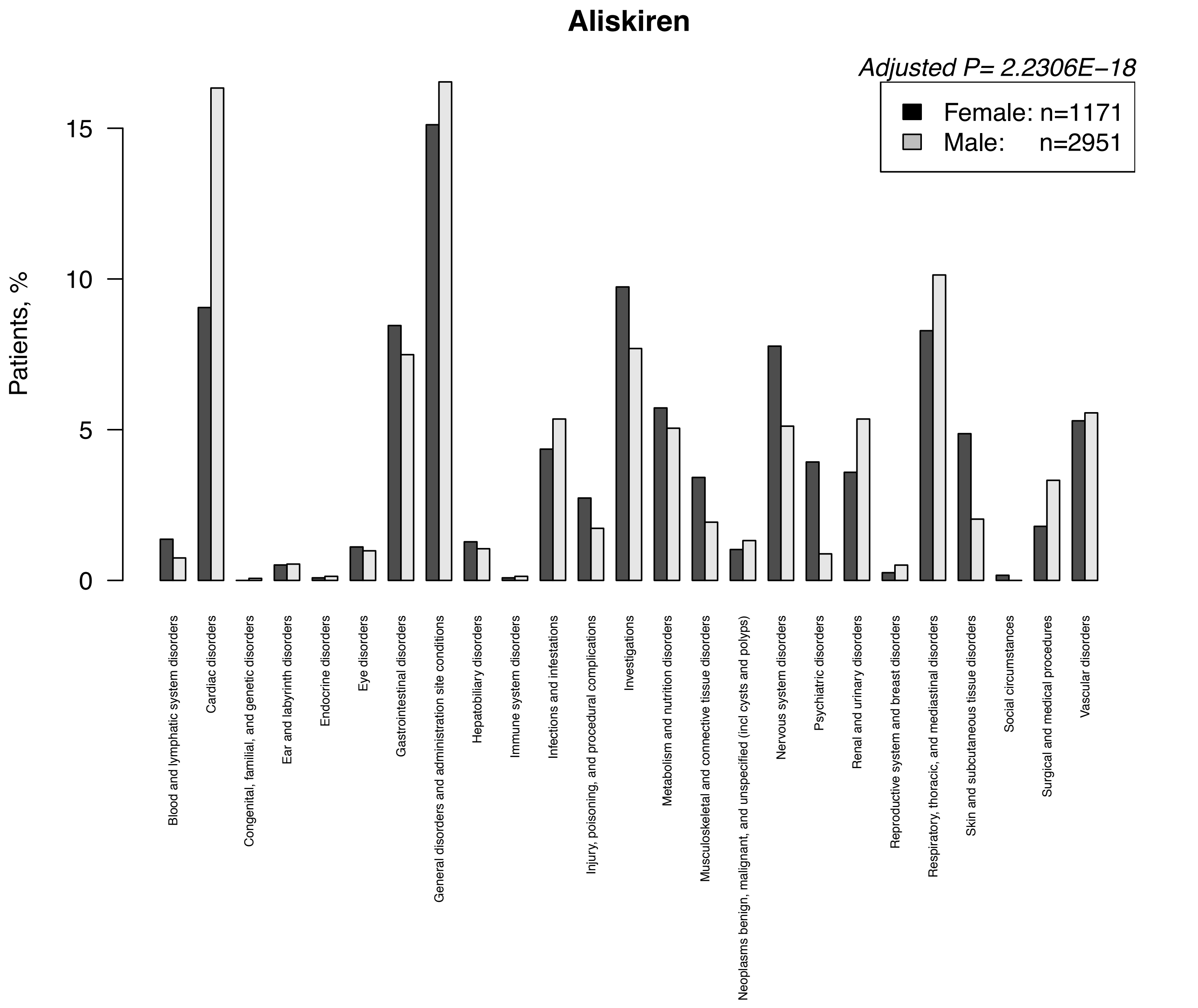
Aliskiren
Adjusted P= 2.2306E−18
Female: n=1171
Male: n=2951
Patients, %
15
10
5
0
Blood and lymphatic system disorders
Cardiac disorders
Congenital, familial, and genetic disorders
Ear and labyrinth disorders
Endocrine disorders
Eye disorders
Gastrointestinal disorders
General disorders and administration site conditions
Hepatobiliary disorders
Immune system disorders
Infections and infestations
Injury, poisoning, and procedural complications
Investigations
Metabolism and nutrition disorders
Musculoskeletal and connective tissue disorders
Neoplasms benign, malignant, and unspecified (incl cysts and polyps)
Nervous system disorders
Psychiatric disorders
Renal and urinary disorders
Reproductive system and breast disorders
Respiratory, thoracic, and mediastinal disorders
Skin and subcutaneous tissue disorders
Social circumstances
Surgical and medical procedures
Vascular disorders

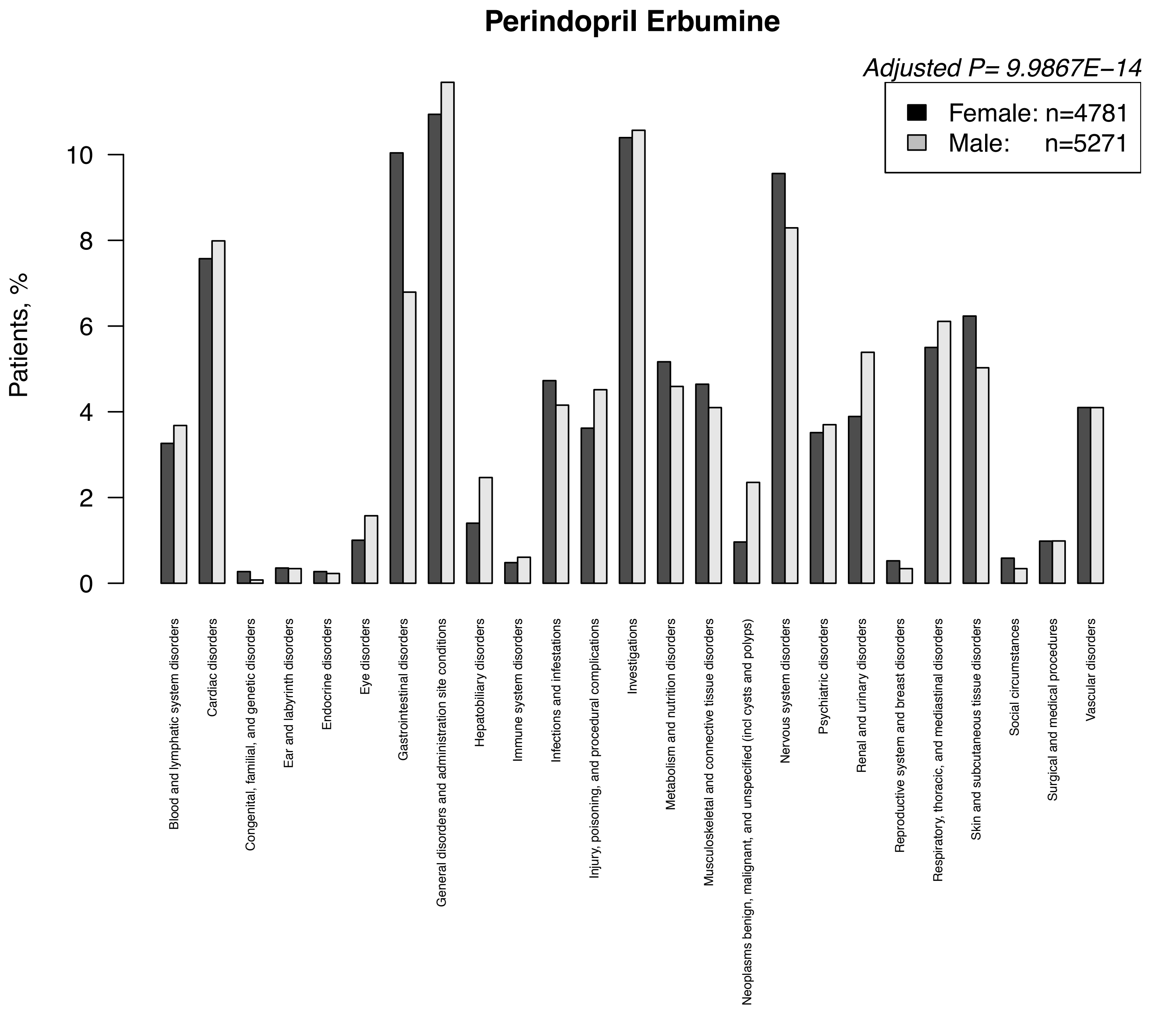
Perindopril Erbumine
Adjusted P= 9.9867E−14
Female: n=4781
Male: n=5271
Patients, %
0
2
4
6
8
10
Blood and lymphatic system disorders
Cardiac disorders
Congenital, familial, and genetic disorders
Ear and labyrinth disorders
Endocrine disorders
Eye disorders
Gastrointestinal disorders
General disorders and administration site conditions
Hepatobiliary disorders
Immune system disorders
Infections and infestations
Injury, poisoning, and procedural complications
Investigations
Metabolism and nutrition disorders
Musculoskeletal and connective tissue disorders
Neoplasms benign, malignant, and unspecified (incl cysts and polyps)
Nervous system disorders
Psychiatric disorders
Renal and urinary disorders
Reproductive system and breast disorders
Respiratory, thoracic, and mediastinal disorders
Skin and subcutaneous tissue disorders
Social circumstances
Surgical and medical procedures
Vascular disorders

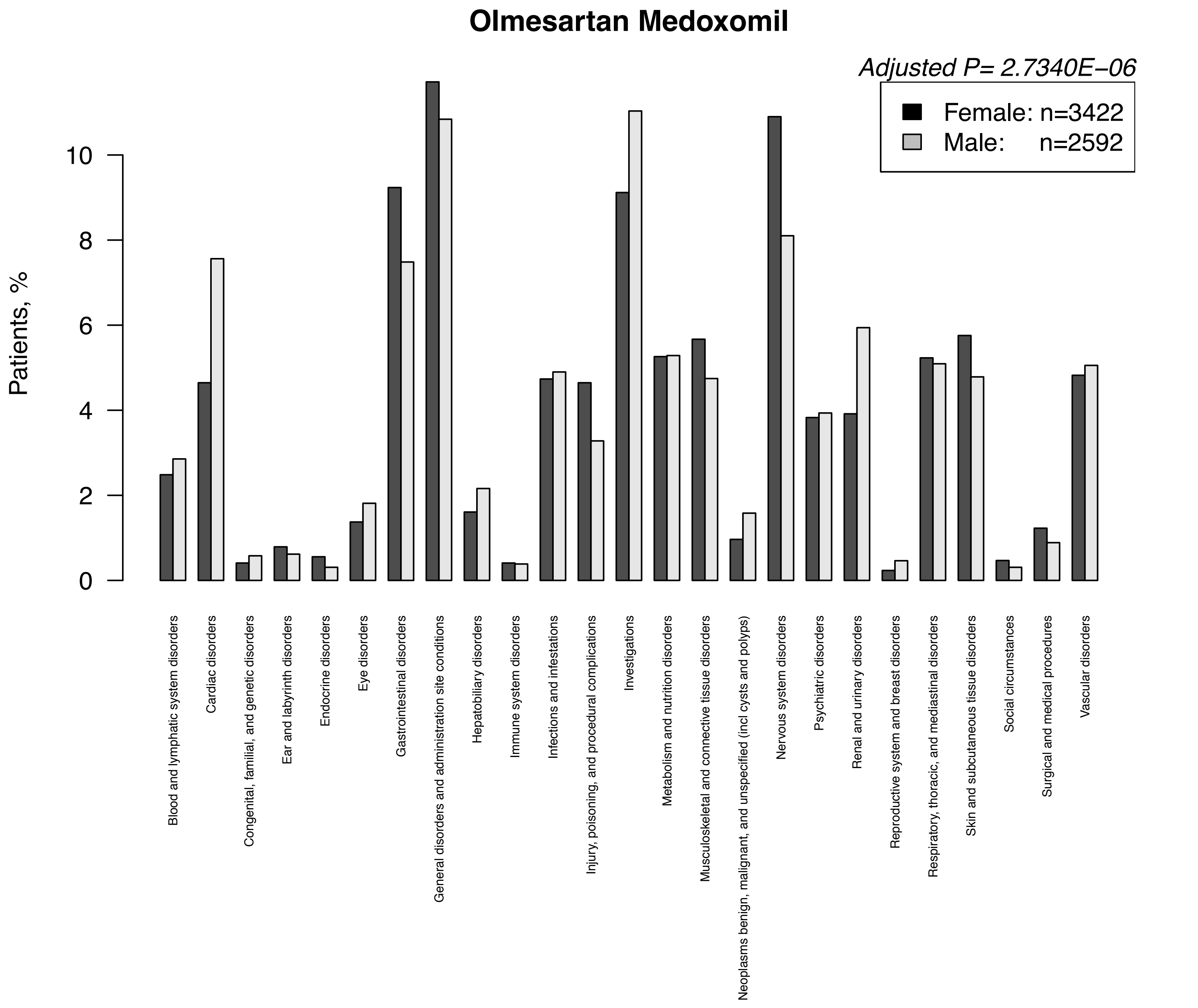

Olmesartan Medoxomil
Adjusted P= 2.7340E−06
Female: n=3422
Male: n=2592
Patients, %
0
2
4
6
8
10
Blood and lymphatic system disorders
Cardiac disorders
Congenital, familial, and genetic disorders
Ear and labyrinth disorders
Endocrine disorders
Eye disorders
Gastrointestinal disorders
General disorders and administration site conditions
Hepatobiliary disorders
Immune system disorders
Infections and infestations
Injury, poisoning, and procedural complications
Investigations
Metabolism and nutrition disorders
Musculoskeletal and connective tissue disorders
Neoplasms benign, malignant, and unspecified (incl cysts and polyps)
Nervous system disorders
Psychiatric disorders
Renal and urinary disorders
Reproductive system and breast disorders
Respiratory, thoracic, and mediastinal disorders
Skin and subcutaneous tissue disorders
Social circumstances
Surgical and medical procedures
Vascular disorders

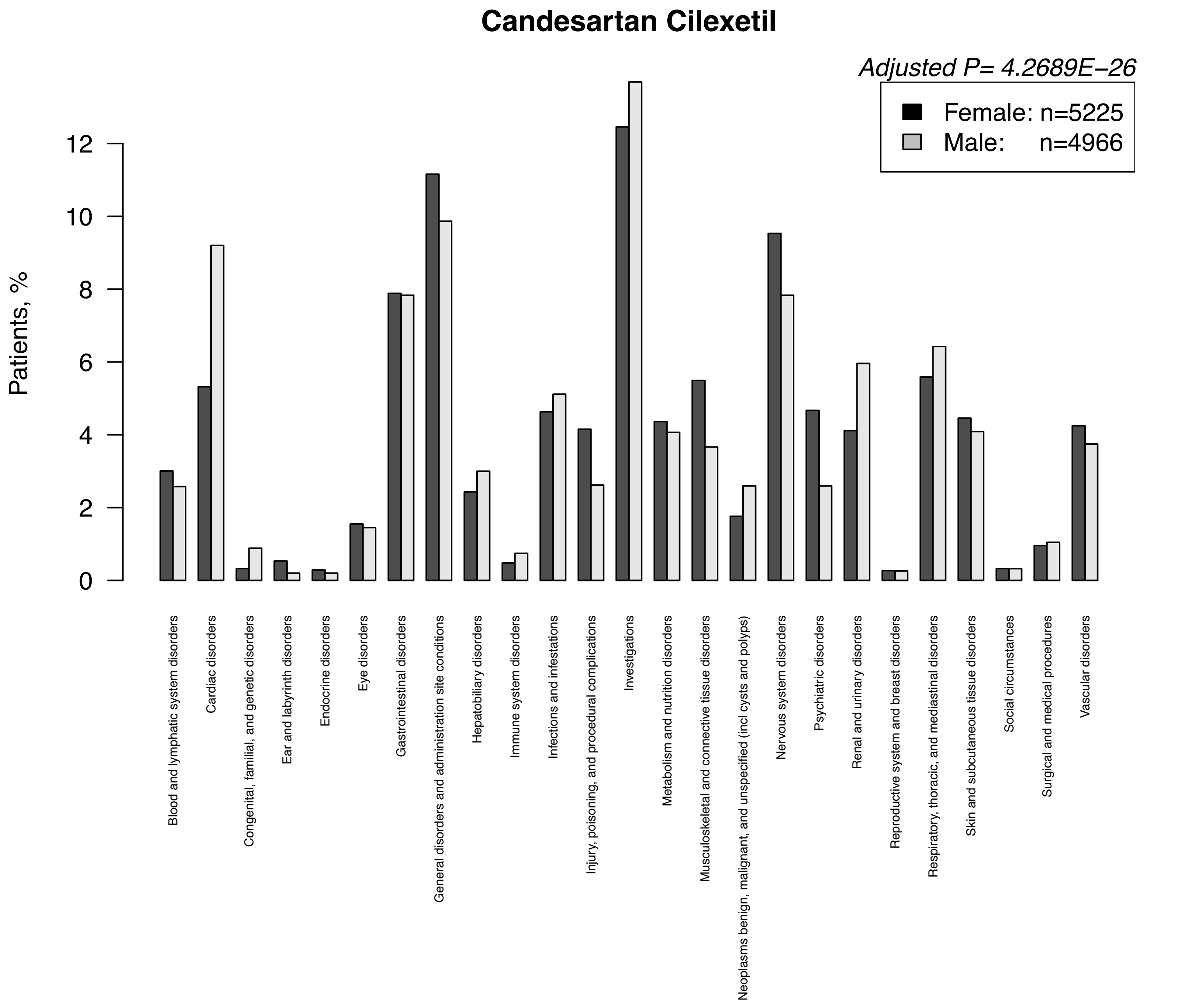

Candesartan Cilexetil
Adjusted P= 4.2689E−26
Female: n=5225
Male: n=4966
Patients, %
0
2
4
6
8
10
12
Blood and lymphatic system disorders
Cardiac disorders
Congenital, familial, and genetic disorders
Ear and labyrinth disorders
Endocrine disorders
Eye disorders
Gastrointestinal disorders
General disorders and administration site conditions
Hepatobiliary disorders
Immune system disorders
Infections and infestations
Injury, poisoning, and procedural complications
Investigations
Metabolism and nutrition disorders
Musculoskeletal and connective tissue disorders
Neoplasms benign, malignant, and unspecified (incl cysts and polyps)
Nervous system disorders
Psychiatric disorders
Renal and urinary disorders
Reproductive system and breast disorders
Respiratory, thoracic, and mediastinal disorders
Skin and subcutaneous tissue disorders
Social circumstances
Surgical and medical procedures
Vascular disorders

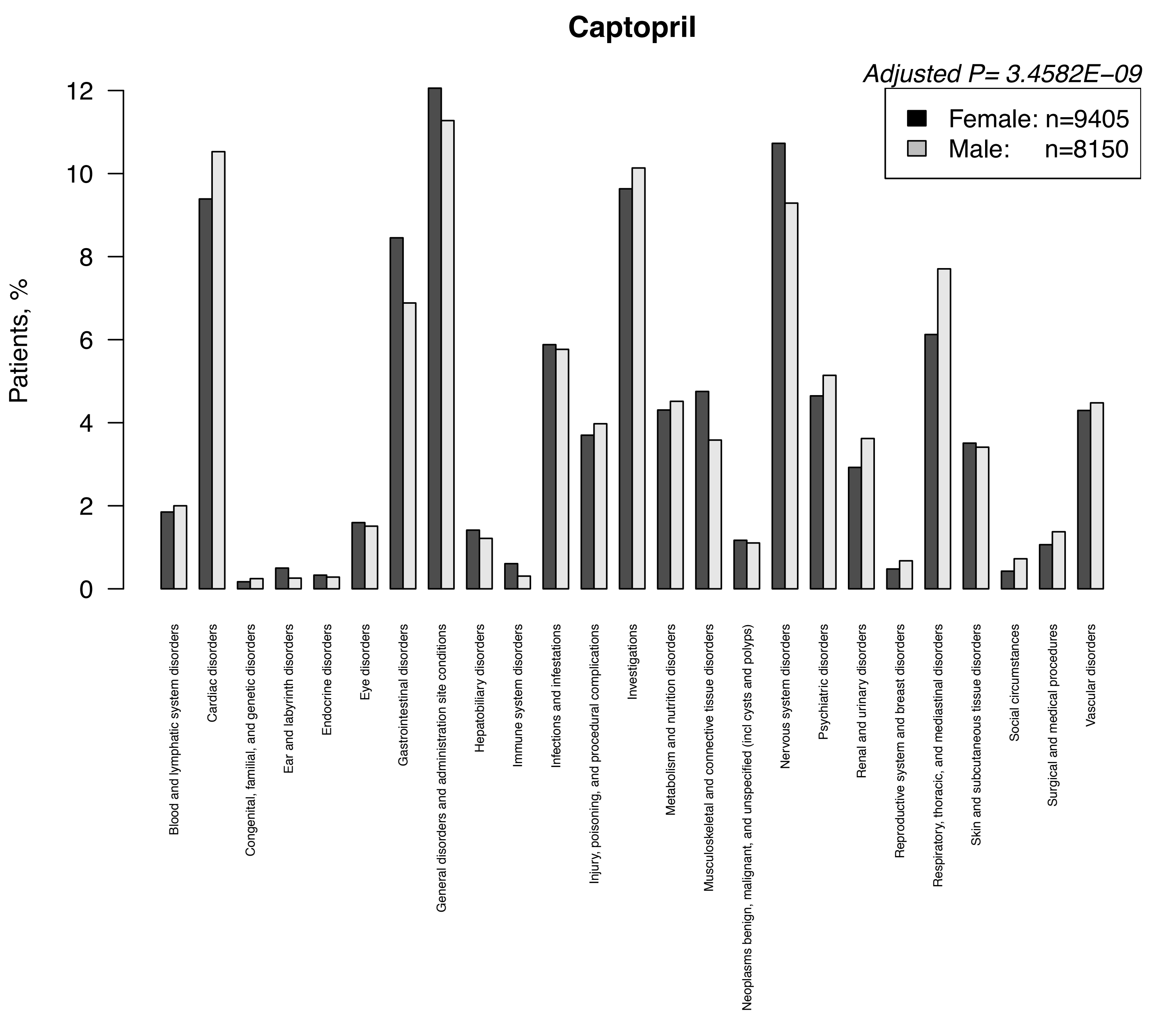

Captopril
Adjusted P= 3.4582E−09
Female: n=9405
Male: n=8150
Patients, %
0
2
4
6
8
10
12
Blood and lymphatic system disorders
Cardiac disorders
Congenital, familial, and genetic disorders
Ear and labyrinth disorders
Endocrine disorders
Eye disorders
Gastrointestinal disorders
General disorders and administration site conditions
Hepatobiliary disorders
Immune system disorders
Infections and infestations
Injury, poisoning, and procedural complications
Investigations
Metabolism and nutrition disorders
Musculoskeletal and connective tissue disorders
Neoplasms benign, malignant, and unspecified (incl cysts and polyps)
Nervous system disorders
Psychiatric disorders
Renal and urinary disorders
Reproductive system and breast disorders
Respiratory, thoracic, and mediastinal disorders
Skin and subcutaneous tissue disorders
Social circumstances
Surgical and medical procedures
Vascular disorders

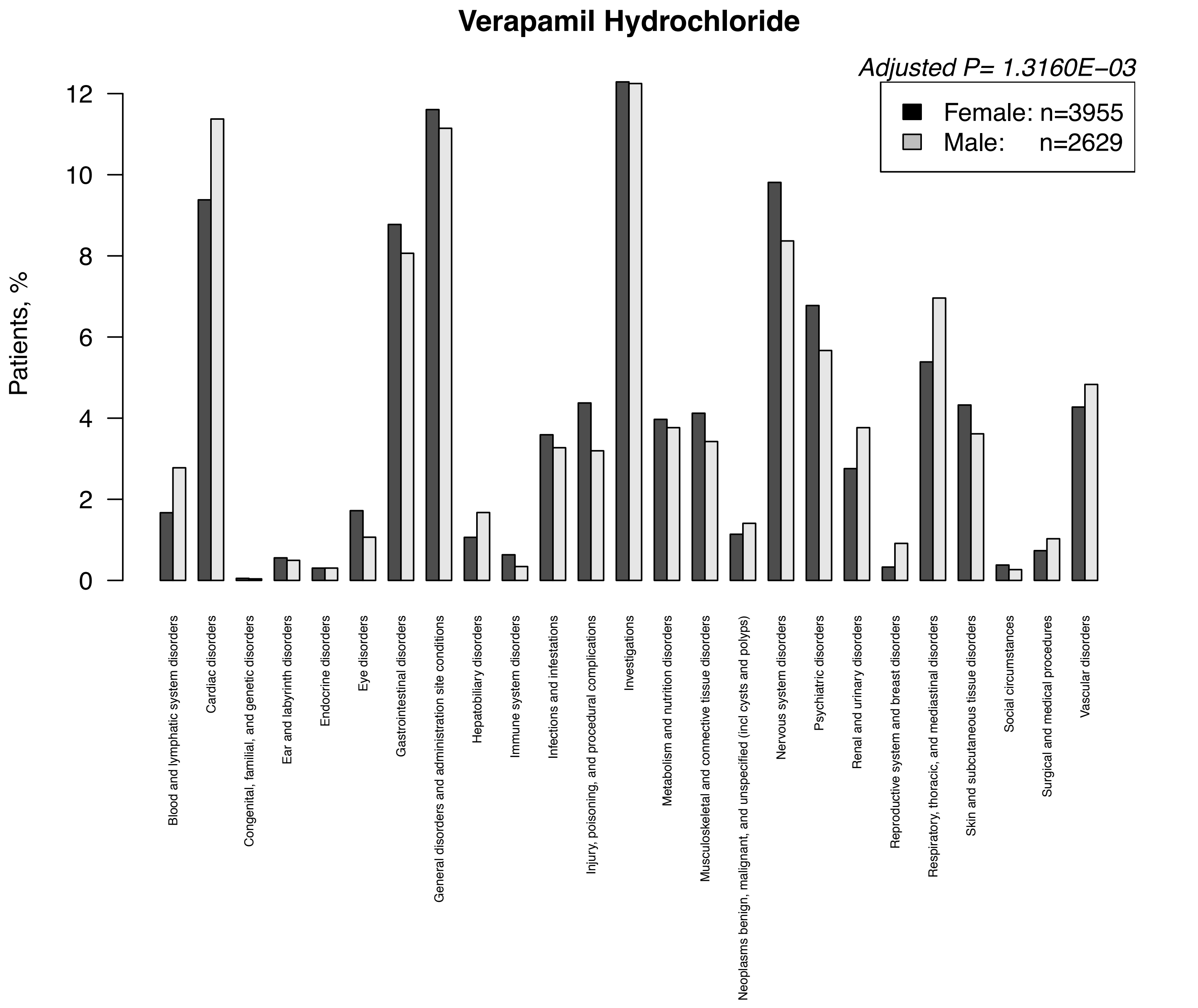

Verapamil Hydrochloride
Adjusted P= 1.3160E−03
Female: n=3955
Male: n=2629
Patients, %
0
2
4
6
8
10
12
Blood and lymphatic system disorders
Cardiac disorders
Congenital, familial, and genetic disorders
Ear and labyrinth disorders
Endocrine disorders
Eye disorders
Gastrointestinal disorders
General disorders and administration site conditions
Hepatobiliary disorders
Immune system disorders
Infections and infestations
Injury, poisoning, and procedural complications
Investigations
Metabolism and nutrition disorders
Musculoskeletal and connective tissue disorders
Neoplasms benign, malignant, and unspecified (incl cysts and polyps)
Nervous system disorders
Psychiatric disorders
Renal and urinary disorders
Reproductive system and breast disorders
Respiratory, thoracic, and mediastinal disorders
Skin and subcutaneous tissue disorders
Social circumstances
Surgical and medical procedures
Vascular disorders

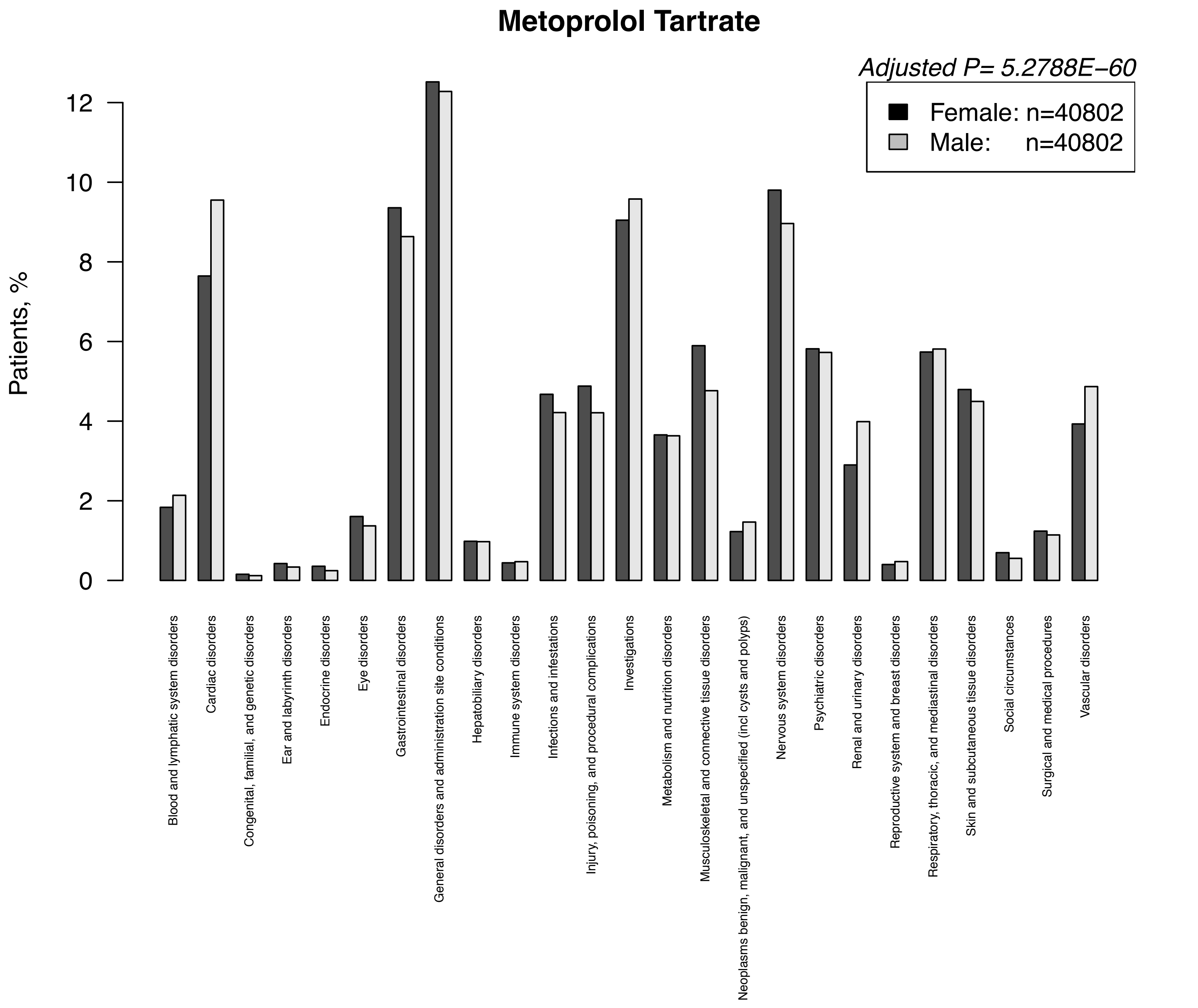

Metoprolol Tartrate
Adjusted P= 5.2788E−60
Female: n=40802
Male: n=40802
Patients, %
0
2
4
6
8
10
12
Blood and lymphatic system disorders
Cardiac disorders
Congenital, familial, and genetic disorders
Ear and labyrinth disorders
Endocrine disorders
Eye disorders
Gastrointestinal disorders
General disorders and administration site conditions
Hepatobiliary disorders
Immune system disorders
Infections and infestations
Injury, poisoning, and procedural complications
Investigations
Metabolism and nutrition disorders
Musculoskeletal and connective tissue disorders
Neoplasms benign, malignant, and unspecified (incl cysts and polyps)
Nervous system disorders
Psychiatric disorders
Renal and urinary disorders
Reproductive system and breast disorders
Respiratory, thoracic, and mediastinal disorders
Skin and subcutaneous tissue disorders
Social circumstances
Surgical and medical procedures
Vascular disorders

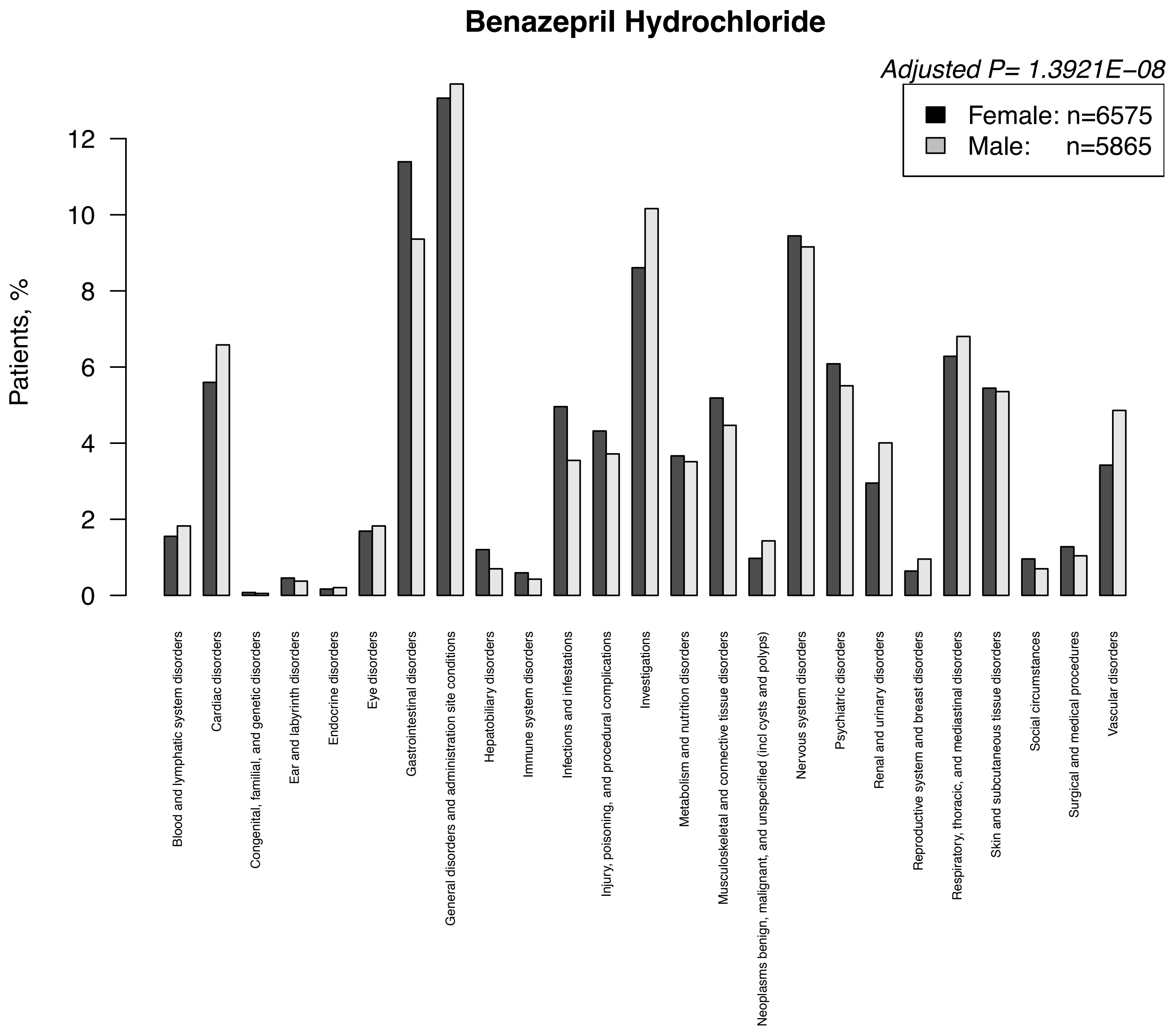
Benazepril Hydrochloride
Adjusted P= 1.3921E−08
Female: n=6575
Male: n=5865
Patients, %
0
2
4
6
8
10
12
Blood and lymphatic system disorders
Cardiac disorders
Congenital, familial, and genetic disorders
Ear and labyrinth disorders
Endocrine disorders
Eye disorders
Gastrointestinal disorders
General disorders and administration site conditions
Hepatobiliary disorders
Immune system disorders
Infections and infestations
Injury, poisoning, and procedural complications
Investigations
Metabolism and nutrition disorders
Musculoskeletal and connective tissue disorders
Neoplasms benign, malignant, and unspecified (incl cysts and polyps)
Nervous system disorders
Psychiatric disorders
Renal and urinary disorders
Reproductive system and breast disorders
Respiratory, thoracic, and mediastinal disorders
Skin and subcutaneous tissue disorders
Social circumstances
Surgical and medical procedures
Vascular disorders

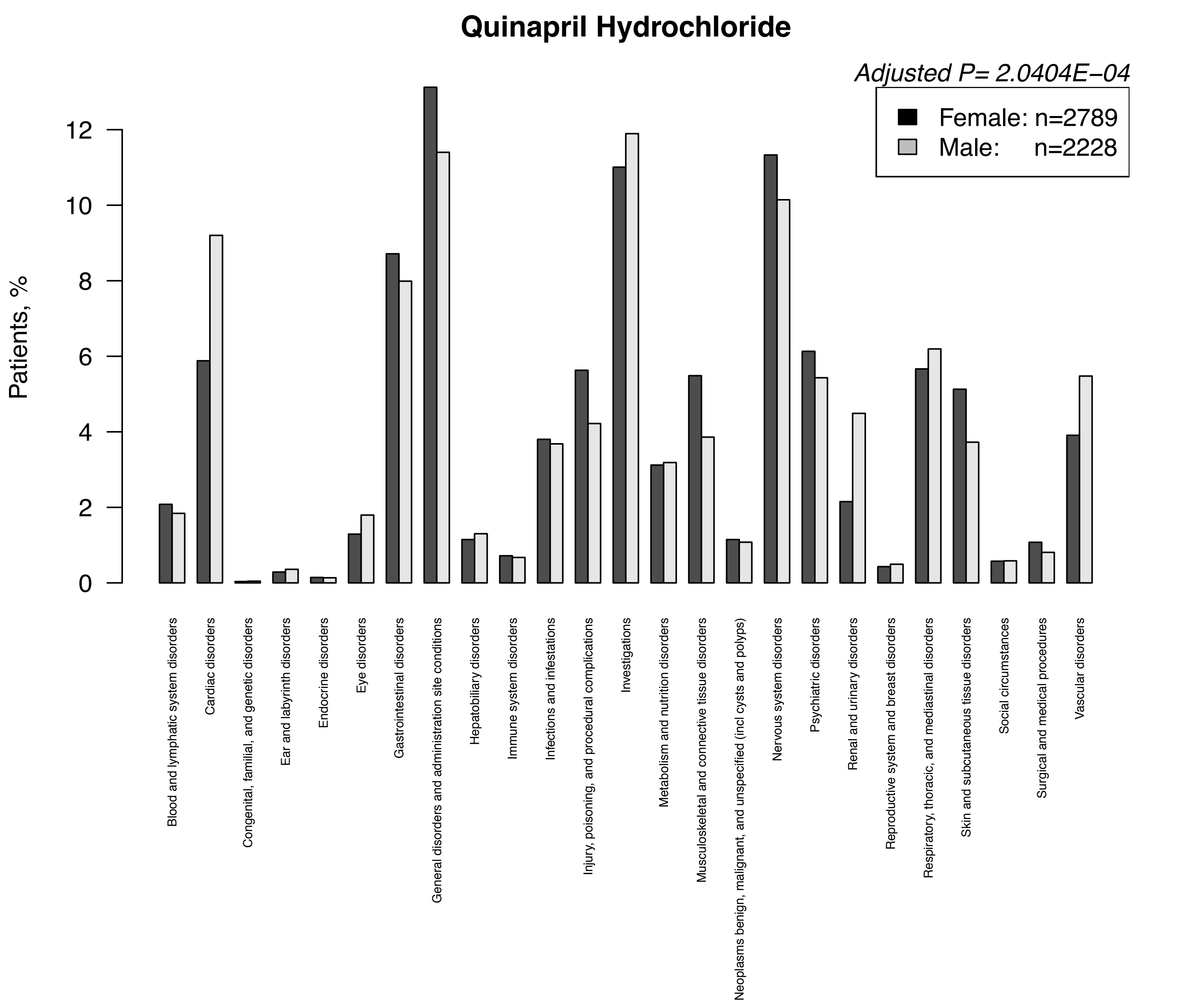
Quinapril Hydrochloride
Adjusted P= 2.0404E−04
Female: n=2789
Male: n=2228
Patients, %
0
2
4
6
8
10
12
Blood and lymphatic system disorders
Cardiac disorders
Congenital, familial, and genetic disorders
Ear and labyrinth disorders
Endocrine disorders
Eye disorders
Gastrointestinal disorders
General disorders and administration site conditions
Hepatobiliary disorders
Immune system disorders
Infections and infestations
Injury, poisoning, and procedural complications
Investigations
Metabolism and nutrition disorders
Musculoskeletal and connective tissue disorders
Neoplasms benign, malignant, and unspecified (incl cysts and polyps)
Nervous system disorders
Psychiatric disorders
Renal and urinary disorders
Reproductive system and breast disorders
Respiratory, thoracic, and mediastinal disorders
Skin and subcutaneous tissue disorders
Social circumstances
Surgical and medical procedures
Vascular disorders

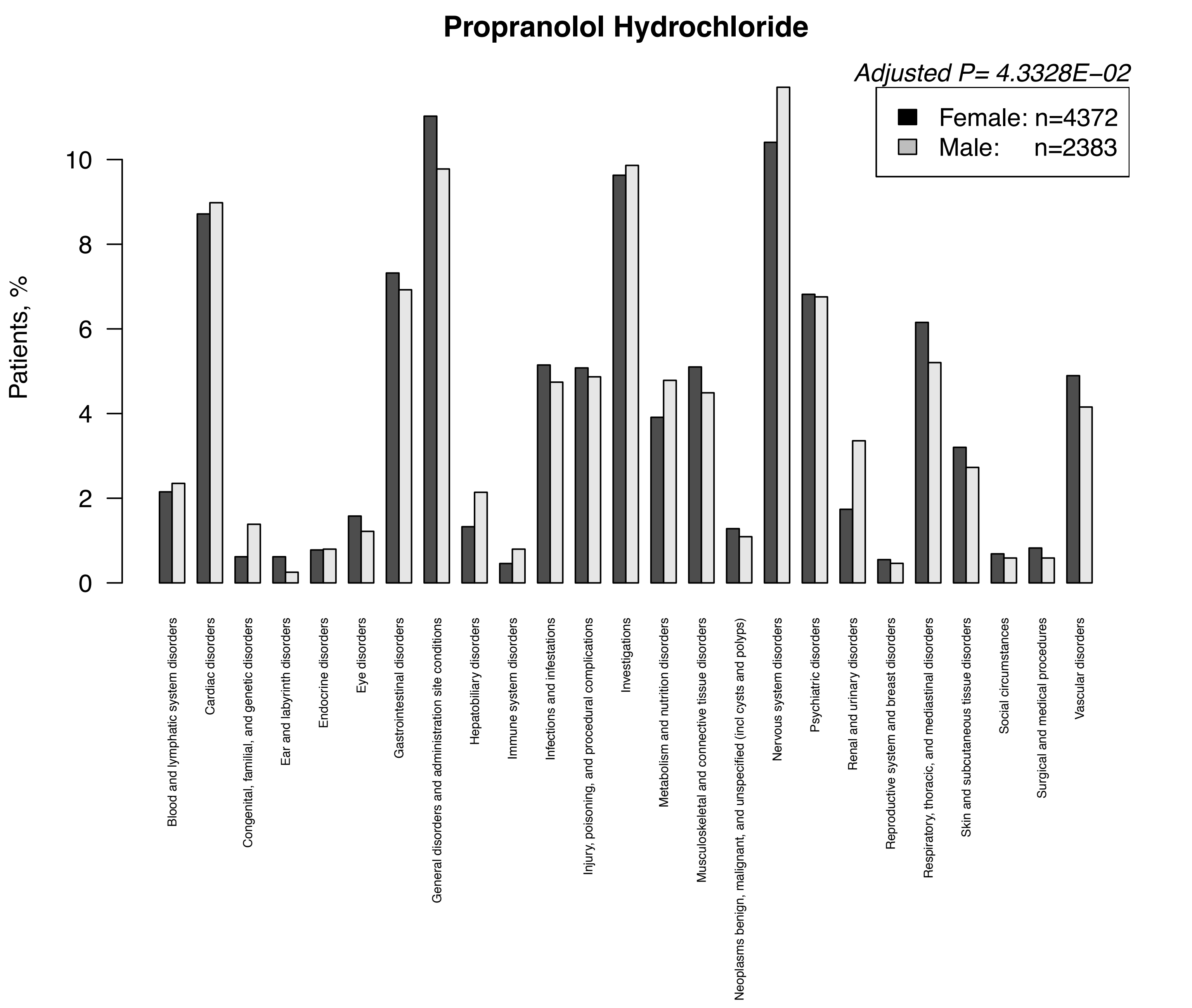
Propranolol Hydrochloride
Adjusted P= 4.3328E−02
Female: n=4372
Male: n=2383
Patients, %
0
2
4
6
8
10
Blood and lymphatic system disorders
Cardiac disorders
Congenital, familial, and genetic disorders
Ear and labyrinth disorders
Endocrine disorders
Eye disorders
Gastrointestinal disorders
General disorders and administration site conditions
Hepatobiliary disorders
Immune system disorders
Infections and infestations
Injury, poisoning, and procedural complications
Investigations
Metabolism and nutrition disorders
Musculoskeletal and connective tissue disorders
Neoplasms benign, malignant, and unspecified (incl cysts and polyps)
Nervous system disorders
Psychiatric disorders
Renal and urinary disorders
Reproductive system and breast disorders
Respiratory, thoracic, and mediastinal disorders
Skin and subcutaneous tissue disorders
Social circumstances
Surgical and medical procedures
Vascular disorders

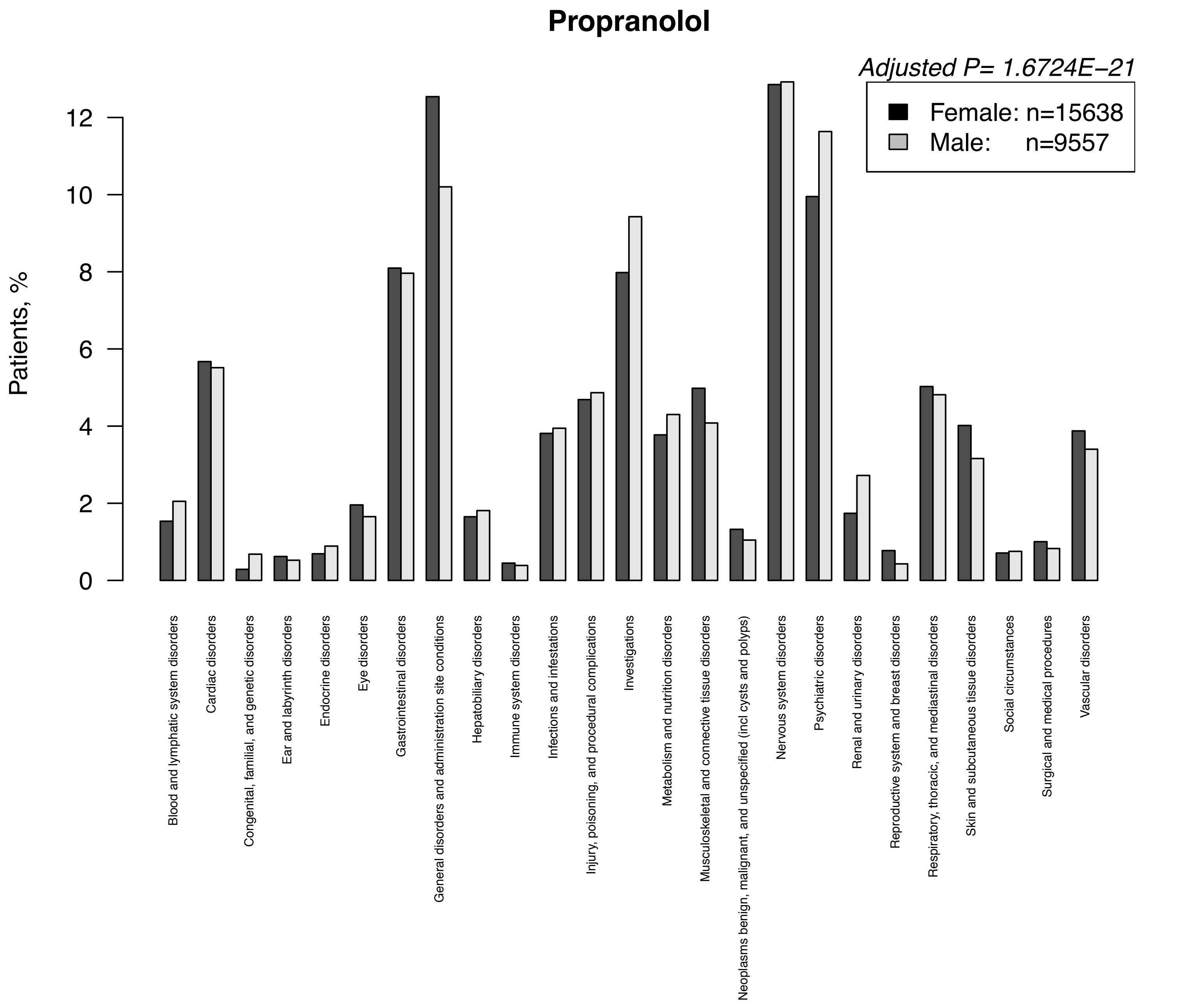
Propranolol
Adjusted P= 1.6724E−21
Female: n=15638
Male: n=9557
Patients, %
0
2
4
6
8
10
12
Blood and lymphatic system disorders
Cardiac disorders
Congenital, familial, and genetic disorders
Ear and labyrinth disorders
Endocrine disorders
Eye disorders
Gastrointestinal disorders
General disorders and administration site conditions
Hepatobiliary disorders
Immune system disorders
Infections and infestations
Injury, poisoning, and procedural complications
Investigations
Metabolism and nutrition disorders
Musculoskeletal and connective tissue disorders
Neoplasms benign, malignant, and unspecified (incl cysts and polyps)
Nervous system disorders
Psychiatric disorders
Renal and urinary disorders
Reproductive system and breast disorders
Respiratory, thoracic, and mediastinal disorders
Skin and subcutaneous tissue disorders
Social circumstances
Surgical and medical procedures
Vascular disorders

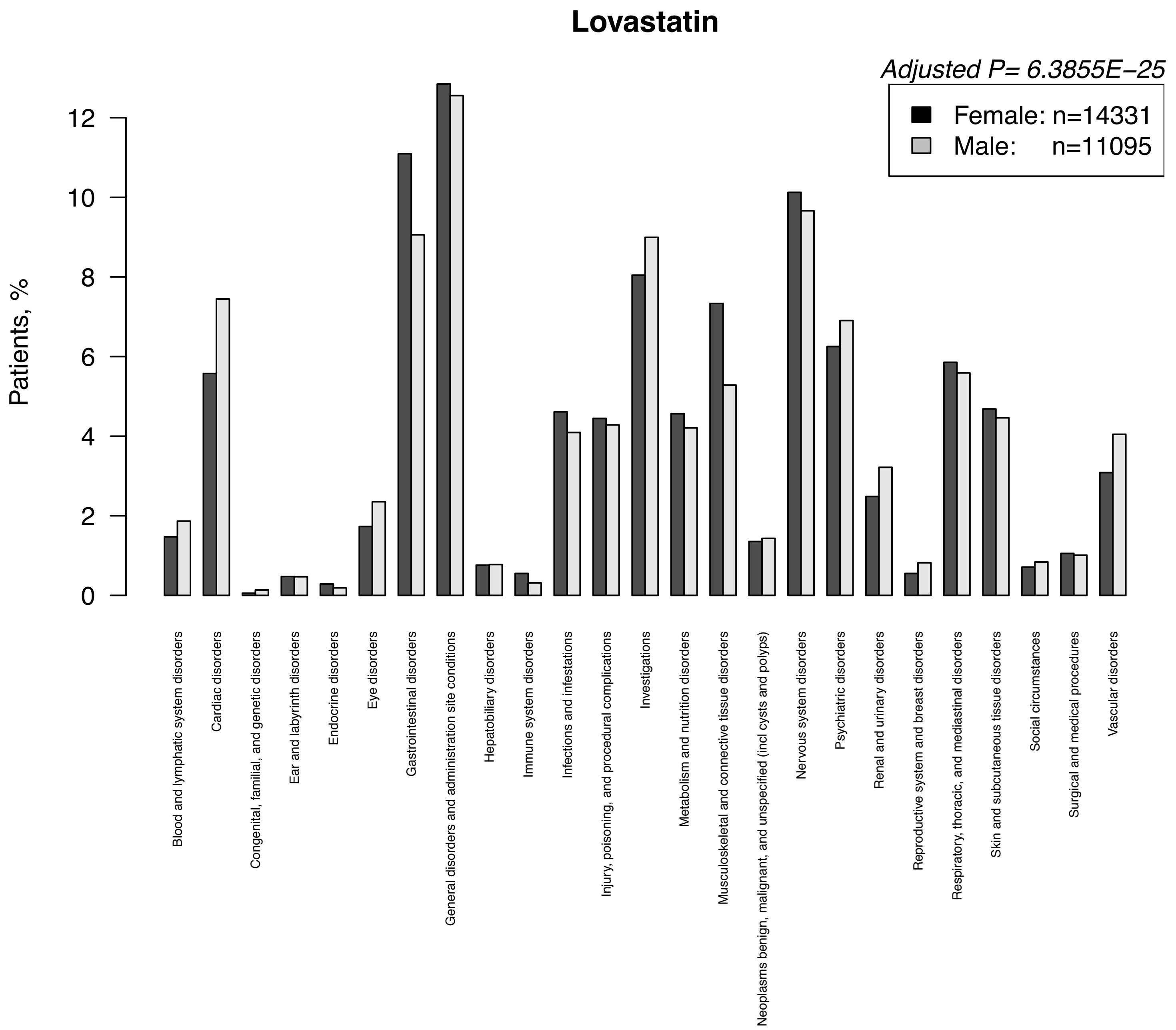
Lovastatin
Adjusted P= 6.3855E−25
Female: n=14331
Male: n=11095
Patients, %
0
2
4
6
8
10
12
Blood and lymphatic system disorders
Cardiac disorders
Congenital, familial, and genetic disorders
Ear and labyrinth disorders
Endocrine disorders
Eye disorders
Gastrointestinal disorders
General disorders and administration site conditions
Hepatobiliary disorders
Immune system disorders
Infections and infestations
Injury, poisoning, and procedural complications
Investigations
Metabolism and nutrition disorders
Musculoskeletal and connective tissue disorders
Neoplasms benign, malignant, and unspecified (incl cysts and polyps)
Nervous system disorders
Psychiatric disorders
Renal and urinary disorders
Reproductive system and breast disorders
Respiratory, thoracic, and mediastinal disorders
Skin and subcutaneous tissue disorders
Social circumstances
Surgical and medical procedures
Vascular disorders

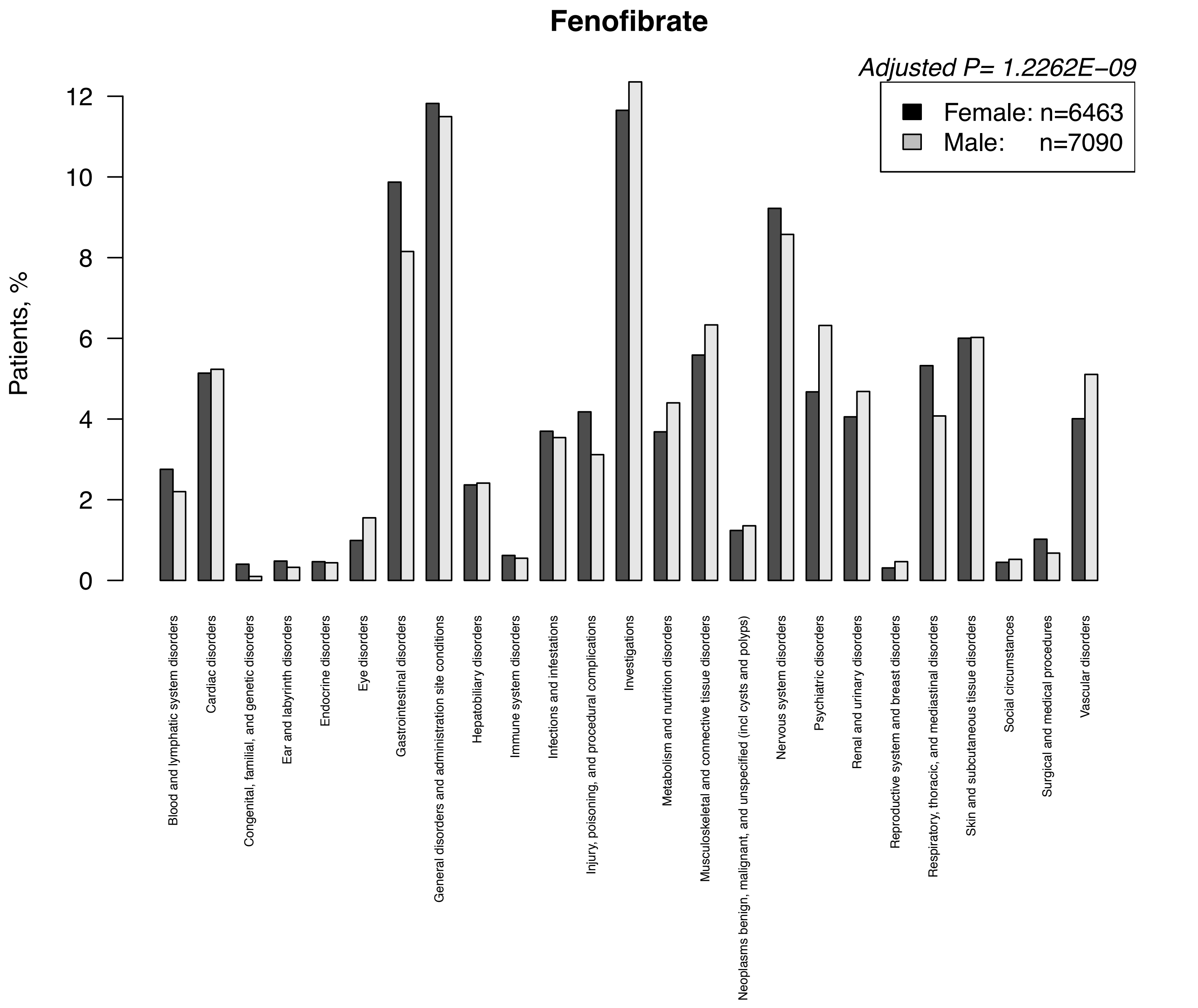

Fenofibrate
Adjusted P= 1.2262E−09
Female: n=6463
Male: n=7090
Patients, %
0
2
4
6
8
10
12
Blood and lymphatic system disorders
Cardiac disorders
Congenital, familial, and genetic disorders
Ear and labyrinth disorders
Endocrine disorders
Eye disorders
Gastrointestinal disorders
General disorders and administration site conditions
Hepatobiliary disorders
Immune system disorders
Infections and infestations
Injury, poisoning, and procedural complications
Investigations
Metabolism and nutrition disorders
Musculoskeletal and connective tissue disorders
Neoplasms benign, malignant, and unspecified (incl cysts and polyps)
Nervous system disorders
Psychiatric disorders
Renal and urinary disorders
Reproductive system and breast disorders
Respiratory, thoracic, and mediastinal disorders
Skin and subcutaneous tissue disorders
Social circumstances
Surgical and medical procedures
Vascular disorders

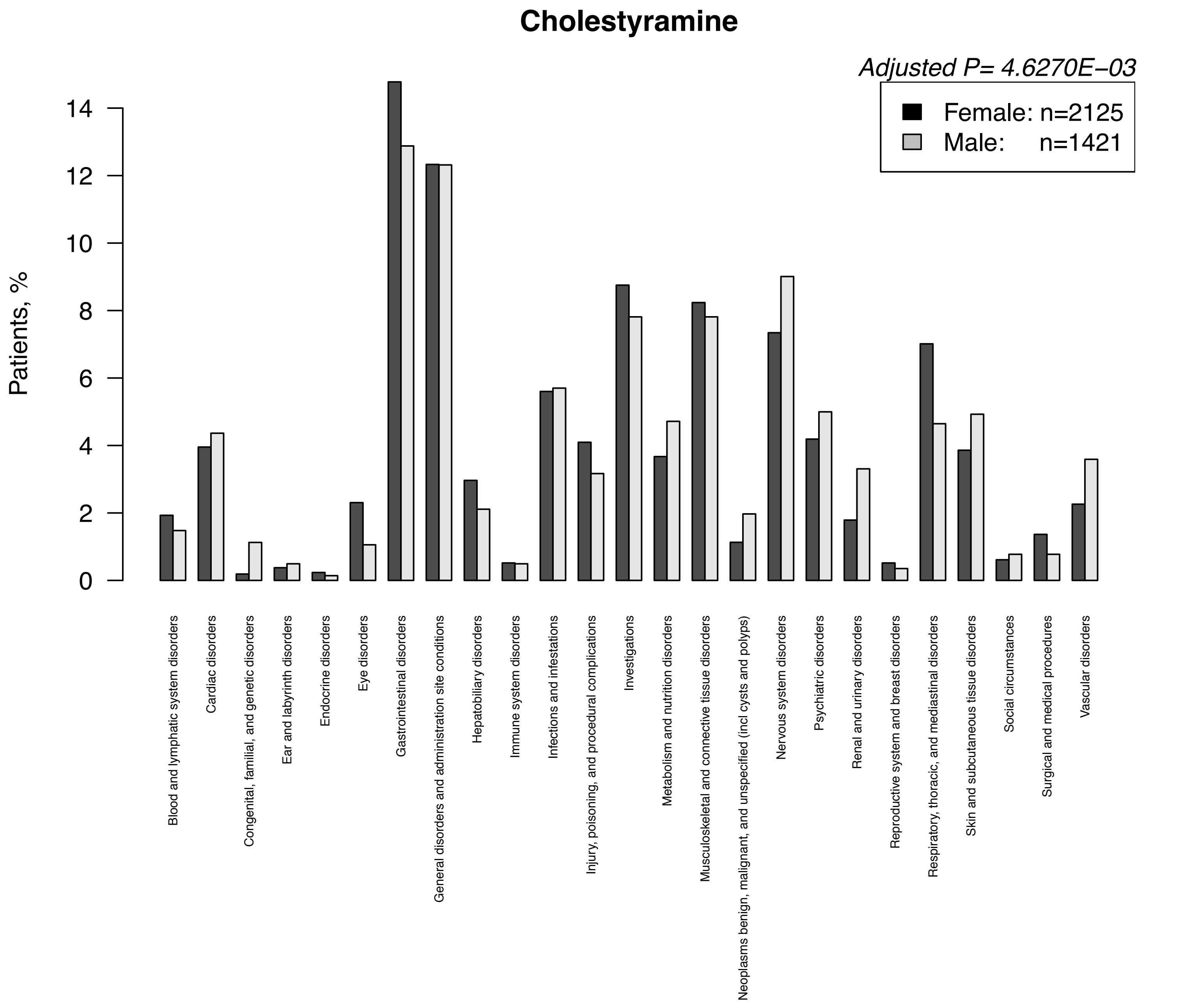

Cholestyramine
Adjusted P= 4.6270E−03
Female: n=2125
Male: n=1421
Patients, %
0
2
4
6
8
10
12
14
Blood and lymphatic system disorders
Cardiac disorders
Congenital, familial, and genetic disorders
Ear and labyrinth disorders
Endocrine disorders
Eye disorders
Gastrointestinal disorders
General disorders and administration site conditions
Hepatobiliary disorders
Immune system disorders
Infections and infestations
Injury, poisoning, and procedural complications
Investigations
Metabolism and nutrition disorders
Musculoskeletal and connective tissue disorders
Neoplasms benign, malignant, and unspecified (incl cysts and polyps)
Nervous system disorders
Psychiatric disorders
Renal and urinary disorders
Reproductive system and breast disorders
Respiratory, thoracic, and mediastinal disorders
Skin and subcutaneous tissue disorders
Social circumstances
Surgical and medical procedures
Vascular disorders

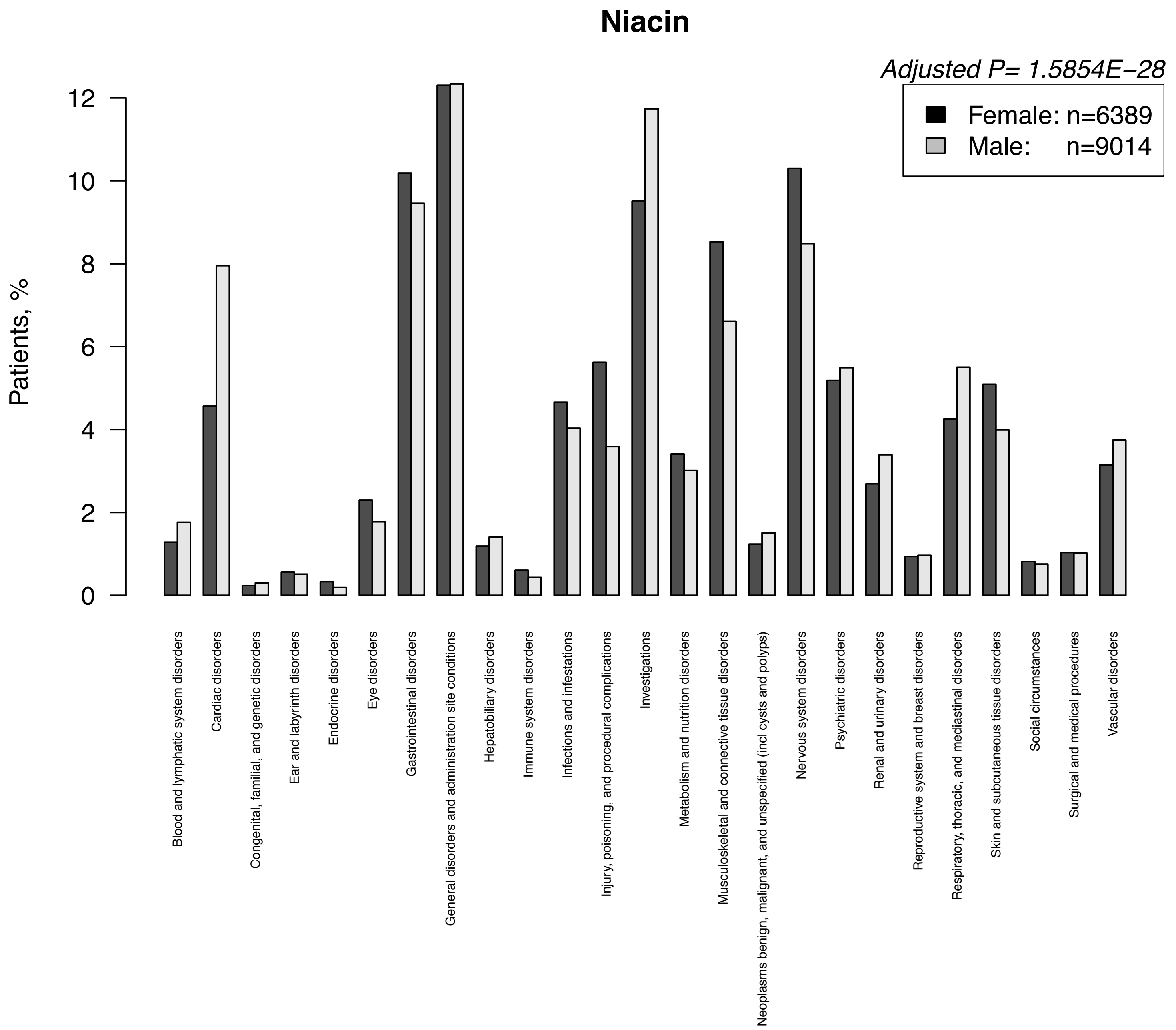

Niacin
Adjusted P= 1.5854E−28
Female: n=6389
Male: n=9014
Patients, %
0
2
4
6
8
10
12
Blood and lymphatic system disorders
Cardiac disorders
Congenital, familial, and genetic disorders
Ear and labyrinth disorders
Endocrine disorders
Eye disorders
Gastrointestinal disorders
General disorders and administration site conditions
Hepatobiliary disorders
Immune system disorders
Infections and infestations
Injury, poisoning, and procedural complications
Investigations
Metabolism and nutrition disorders
Musculoskeletal and connective tissue disorders
Neoplasms benign, malignant, and unspecified (incl cysts and polyps)
Nervous system disorders
Psychiatric disorders
Renal and urinary disorders
Reproductive system and breast disorders
Respiratory, thoracic, and mediastinal disorders
Skin and subcutaneous tissue disorders
Social circumstances
Surgical and medical procedures
Vascular disorders

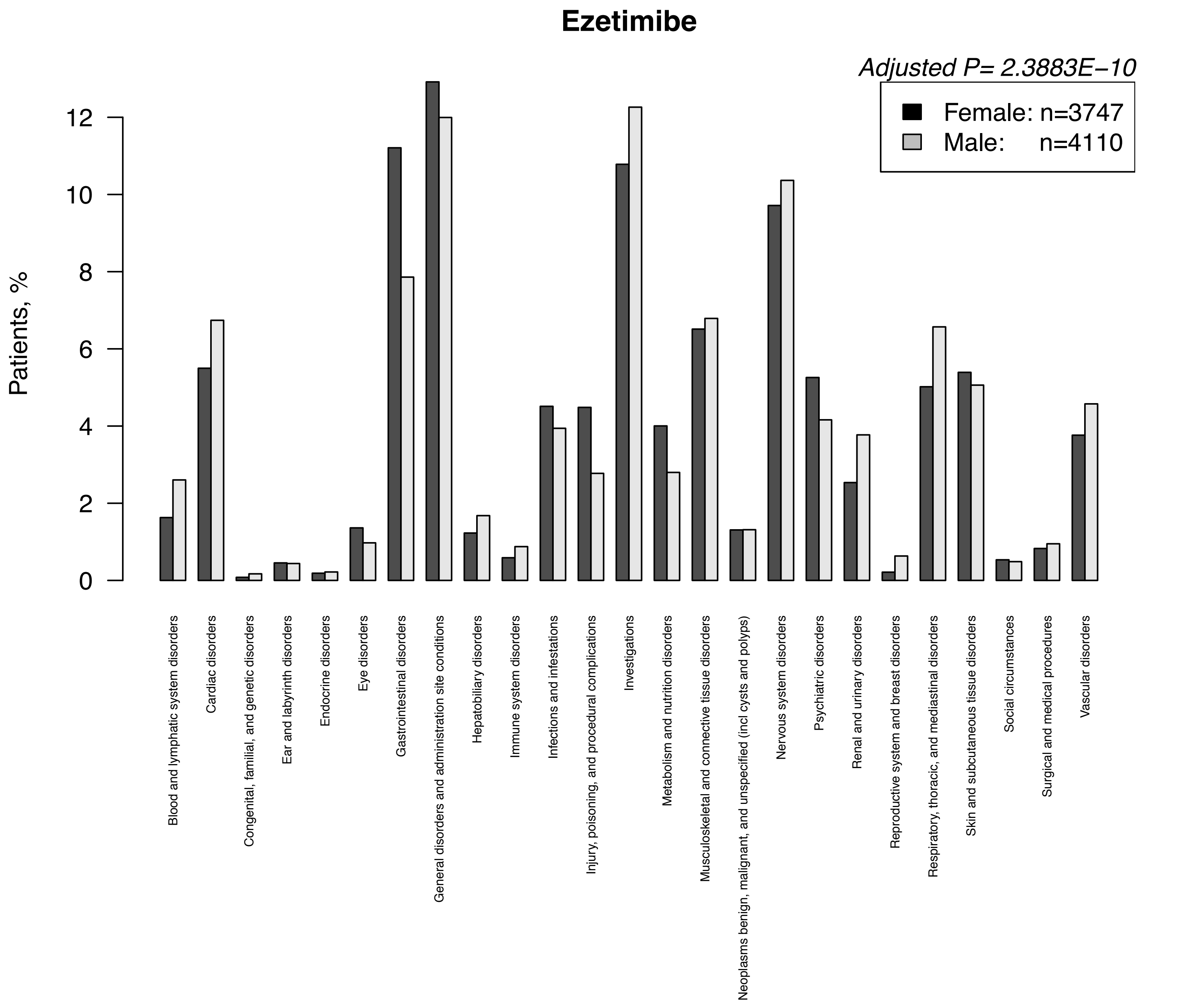

Ezetimibe
Adjusted P= 2.3883E−10
Female: n=3747
Male: n=4110
Patients, %
0
2
4
6
8
10
12
Blood and lymphatic system disorders
Cardiac disorders
Congenital, familial, and genetic disorders
Ear and labyrinth disorders
Endocrine disorders
Eye disorders
Gastrointestinal disorders
General disorders and administration site conditions
Hepatobiliary disorders
Immune system disorders
Infections and infestations
Injury, poisoning, and procedural complications
Investigations
Metabolism and nutrition disorders
Musculoskeletal and connective tissue disorders
Neoplasms benign, malignant, and unspecified (incl cysts and polyps)
Nervous system disorders
Psychiatric disorders
Renal and urinary disorders
Reproductive system and breast disorders
Respiratory, thoracic, and mediastinal disorders
Skin and subcutaneous tissue disorders
Social circumstances
Surgical and medical procedures
Vascular disorders

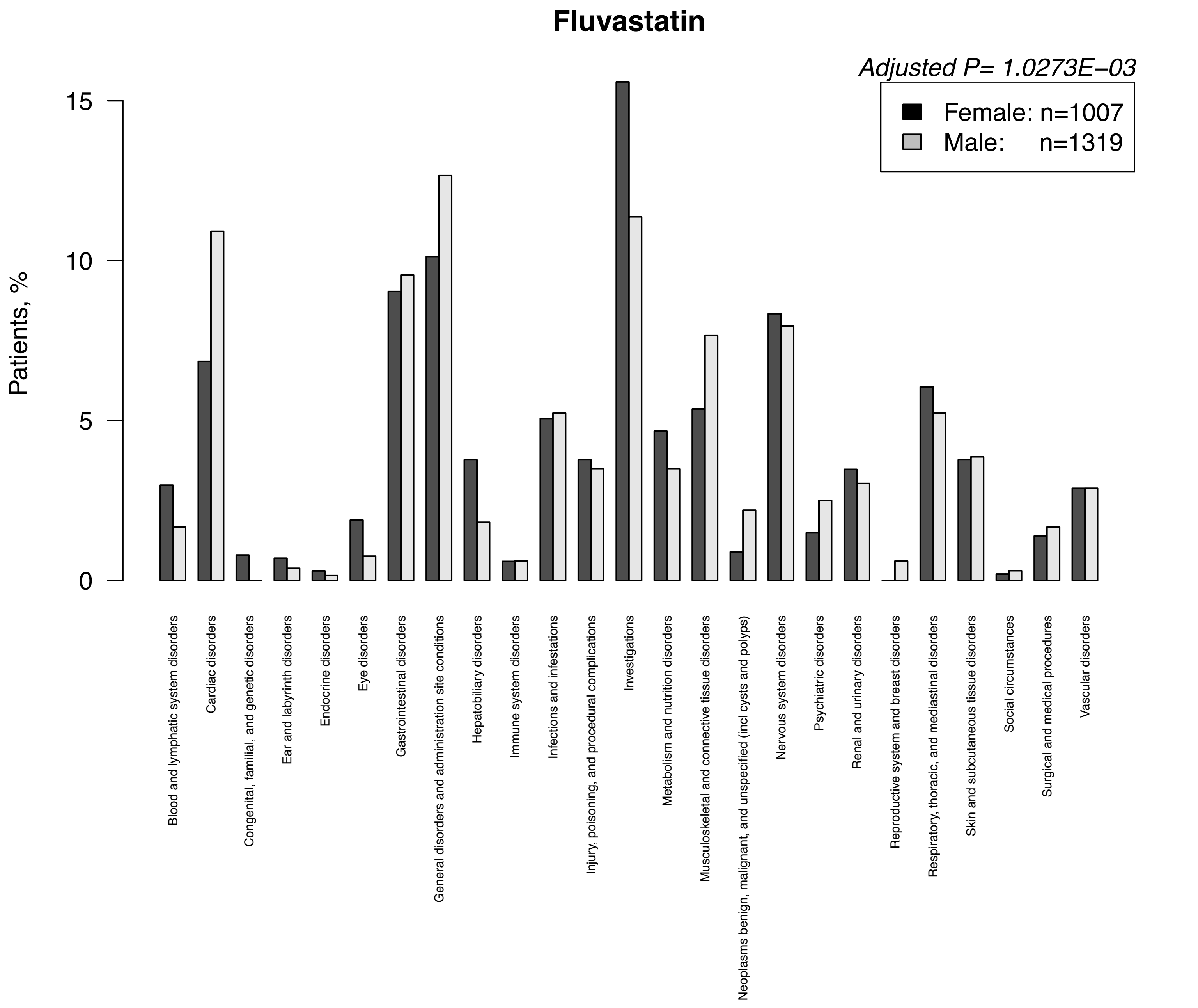

Fluvastatin
Adjusted P= 1.0273E−03
Female: n=1007
Male: n=1319
Patients, %
15
10
5
0
Blood and lymphatic system disorders
Cardiac disorders
Congenital, familial, and genetic disorders
Ear and labyrinth disorders
Endocrine disorders
Eye disorders
Gastrointestinal disorders
General disorders and administration site conditions
Hepatobiliary disorders
Immune system disorders
Infections and infestations
Injury, poisoning, and procedural complications
Investigations
Metabolism and nutrition disorders
Musculoskeletal and connective tissue disorders
Neoplasms benign, malignant, and unspecified (incl cysts and polyps)
Nervous system disorders
Psychiatric disorders
Renal and urinary disorders
Reproductive system and breast disorders
Respiratory, thoracic, and mediastinal disorders
Skin and subcutaneous tissue disorders
Social circumstances
Surgical and medical procedures
Vascular disorders

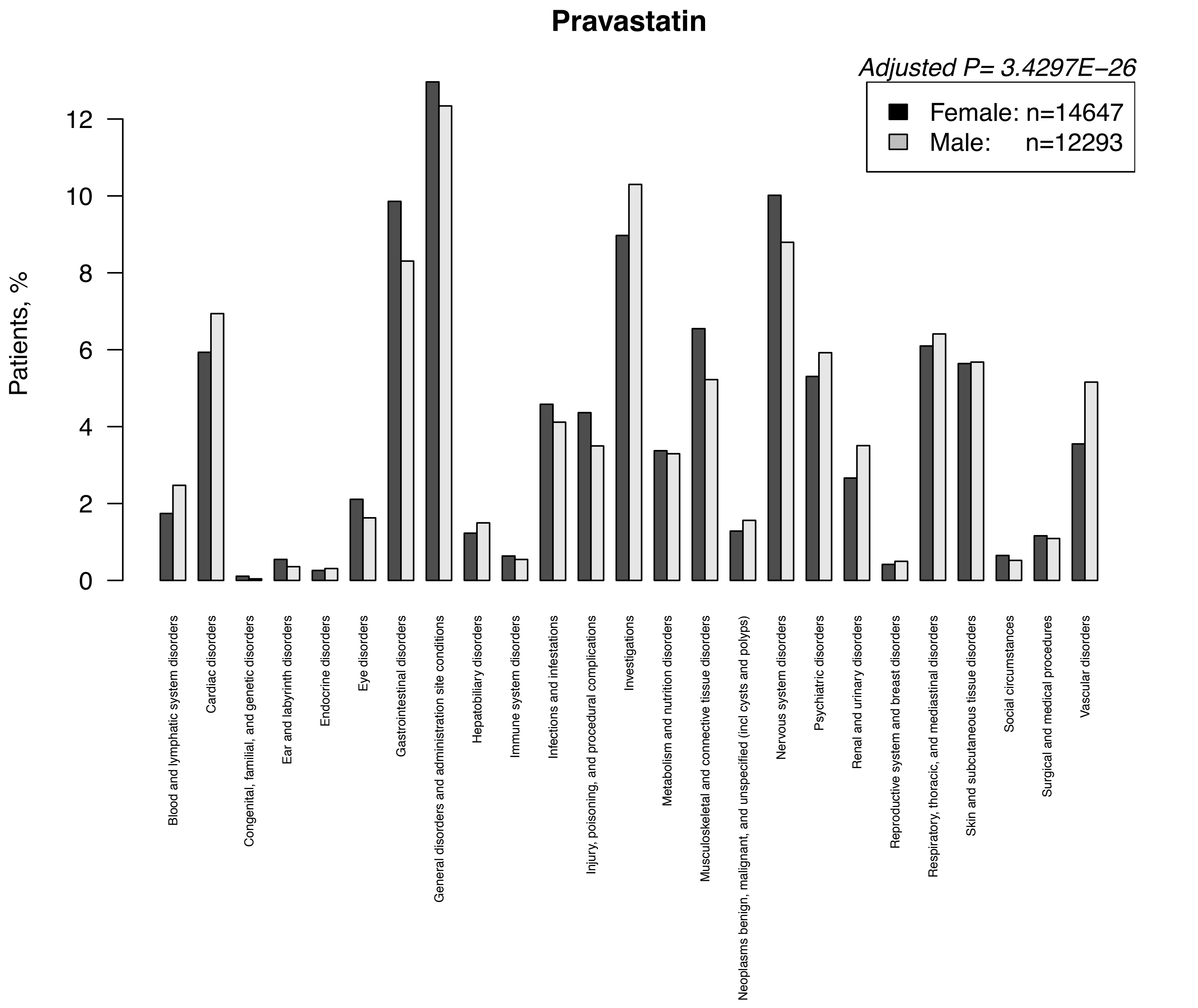
Pravastatin
Adjusted P= 3.4297E−26
Female: n=14647
Male: n=12293
Patients, %
0
2
4
6
8
10
12
Blood and lymphatic system disorders
Cardiac disorders
Congenital, familial, and genetic disorders
Ear and labyrinth disorders
Endocrine disorders
Eye disorders
Gastrointestinal disorders
General disorders and administration site conditions
Hepatobiliary disorders
Immune system disorders
Infections and infestations
Injury, poisoning, and procedural complications
Investigations
Metabolism and nutrition disorders
Musculoskeletal and connective tissue disorders
Neoplasms benign, malignant, and unspecified (incl cysts and polyps)
Nervous system disorders
Psychiatric disorders
Renal and urinary disorders
Reproductive system and breast disorders
Respiratory, thoracic, and mediastinal disorders
Skin and subcutaneous tissue disorders
Social circumstances
Surgical and medical procedures
Vascular disorders

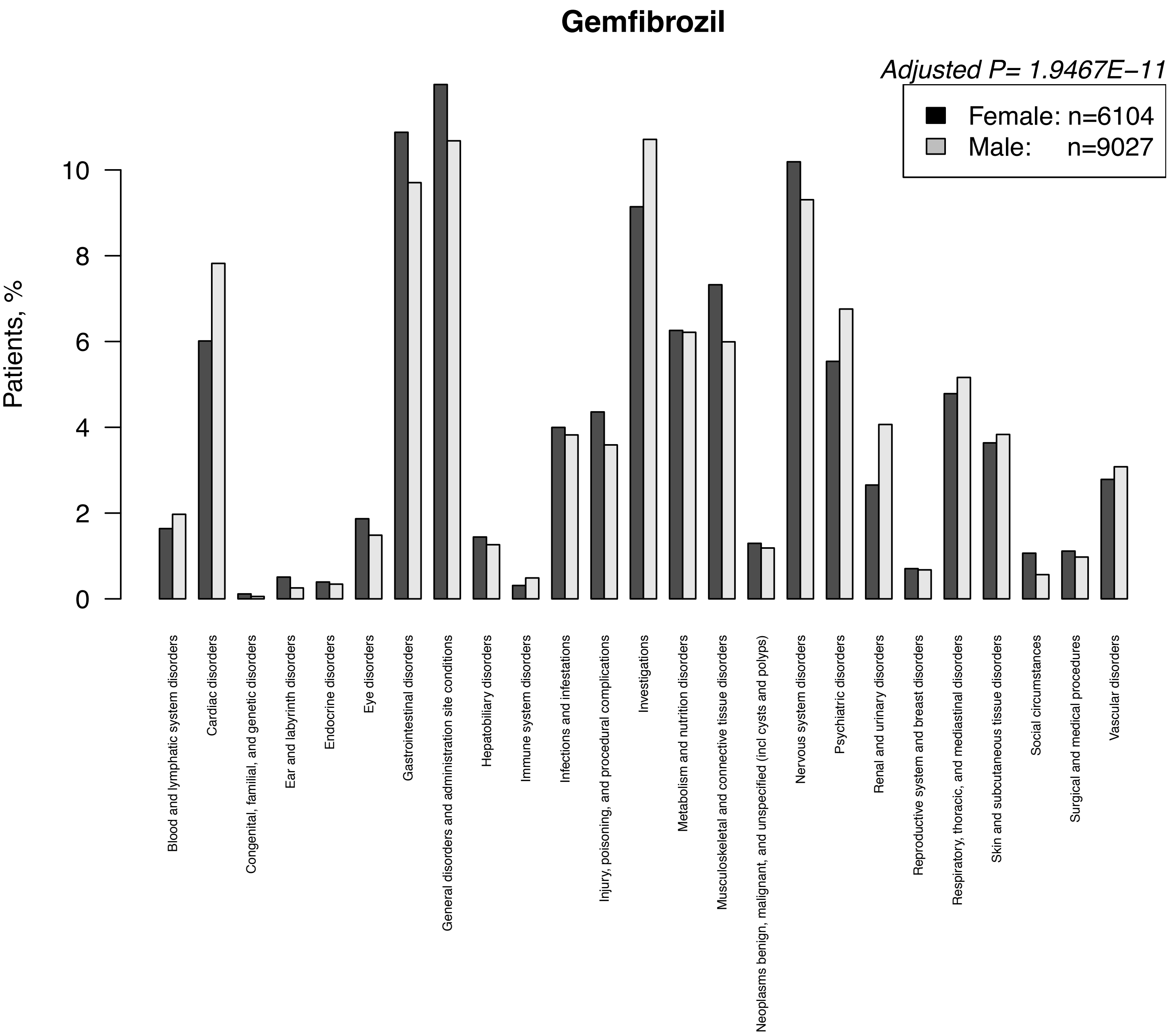

Gemfibrozil
Adjusted P= 1.9467E−11
Female: n=6104
Male: n=9027
Patients, %
0
2
4
6
8
10
Blood and lymphatic system disorders
Cardiac disorders
Congenital, familial, and genetic disorders
Ear and labyrinth disorders
Endocrine disorders
Eye disorders
Gastrointestinal disorders
General disorders and administration site conditions
Hepatobiliary disorders
Immune system disorders
Infections and infestations
Injury, poisoning, and procedural complications
Investigations
Metabolism and nutrition disorders
Musculoskeletal and connective tissue disorders
Neoplasms benign, malignant, and unspecified (incl cysts and polyps)
Nervous system disorders
Psychiatric disorders
Renal and urinary disorders
Reproductive system and breast disorders
Respiratory, thoracic, and mediastinal disorders
Skin and subcutaneous tissue disorders
Social circumstances
Surgical and medical procedures
Vascular disorders

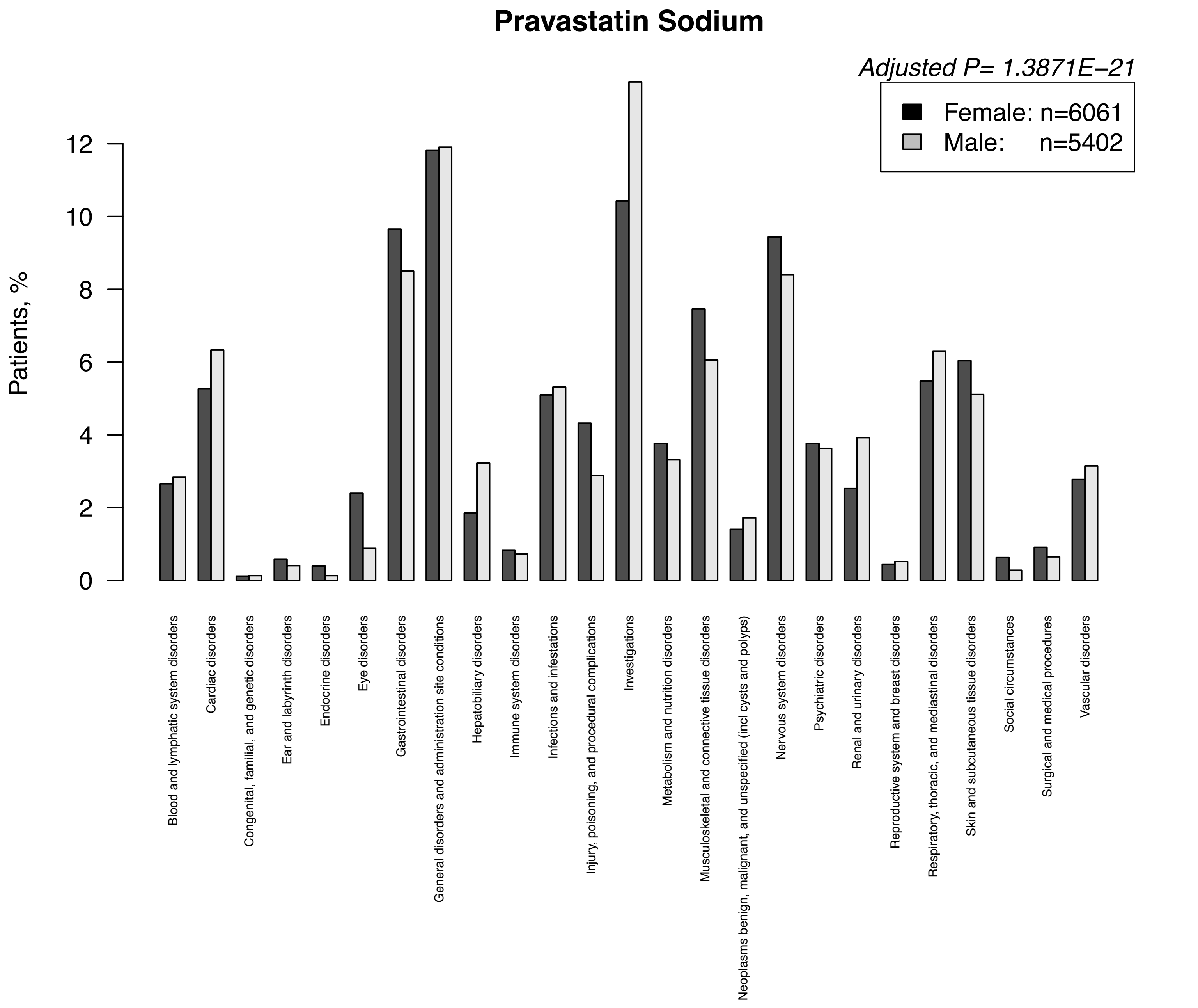

Pravastatin Sodium
Adjusted P= 1.3871E−21
Female: n=6061
Male: n=5402
Patients, %
0
2
4
6
8
10
12
Blood and lymphatic system disorders
Cardiac disorders
Congenital, familial, and genetic disorders
Ear and labyrinth disorders
Endocrine disorders
Eye disorders
Gastrointestinal disorders
General disorders and administration site conditions
Hepatobiliary disorders
Immune system disorders
Infections and infestations
Injury, poisoning, and procedural complications
Investigations
Metabolism and nutrition disorders
Musculoskeletal and connective tissue disorders
Neoplasms benign, malignant, and unspecified (incl cysts and polyps)
Nervous system disorders
Psychiatric disorders
Renal and urinary disorders
Reproductive system and breast disorders
Respiratory, thoracic, and mediastinal disorders
Skin and subcutaneous tissue disorders
Social circumstances
Surgical and medical procedures
Vascular disorders

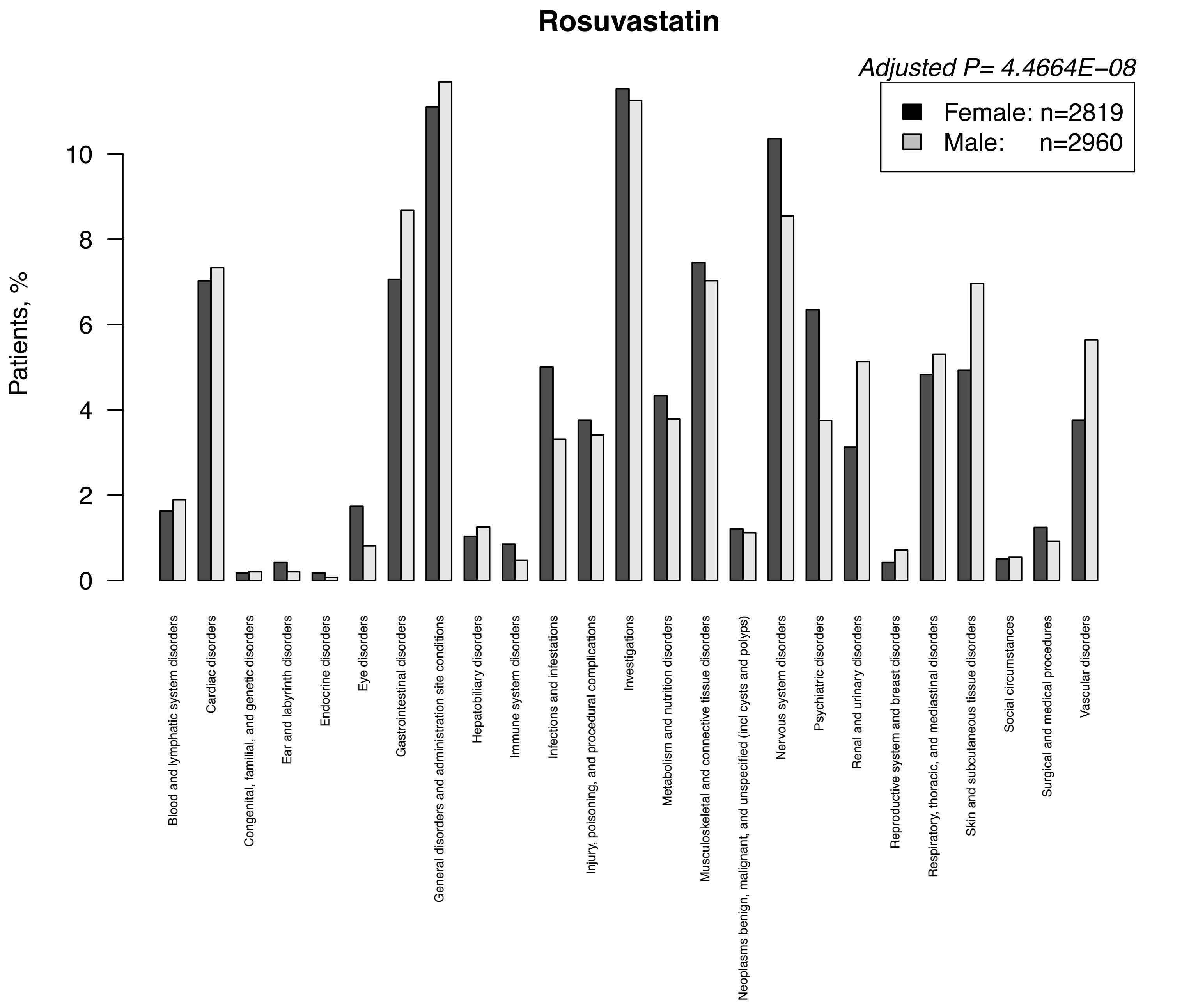

Rosuvastatin
Adjusted P= 4.4664E−08
Female: n=2819
Male: n=2960
Patients, %
0
2
4
6
8
10
Blood and lymphatic system disorders
Cardiac disorders
Congenital, familial, and genetic disorders
Ear and labyrinth disorders
Endocrine disorders
Eye disorders
Gastrointestinal disorders
General disorders and administration site conditions
Hepatobiliary disorders
Immune system disorders
Infections and infestations
Injury, poisoning, and procedural complications
Investigations
Metabolism and nutrition disorders
Musculoskeletal and connective tissue disorders
Neoplasms benign, malignant, and unspecified (incl cysts and polyps)
Nervous system disorders
Psychiatric disorders
Renal and urinary disorders
Reproductive system and breast disorders
Respiratory, thoracic, and mediastinal disorders
Skin and subcutaneous tissue disorders
Social circumstances
Surgical and medical procedures
Vascular disorders

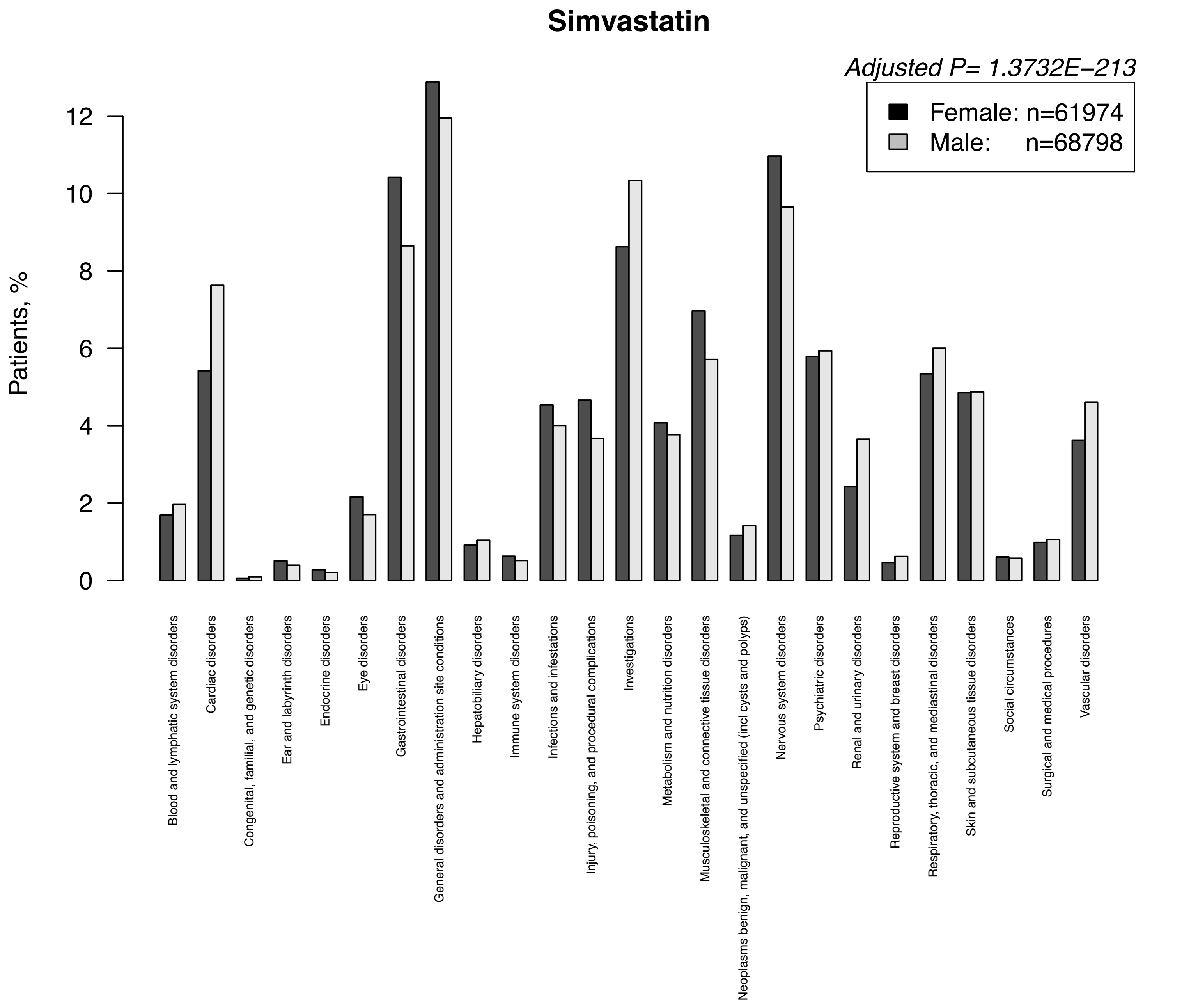

Simvastatin
Adjusted P= 1.3732E−213
Female: n=61974
Male: n=68798
Patients, %
0
2
4
6
8
10
12
Blood and lymphatic system disorders
Cardiac disorders
Congenital, familial, and genetic disorders
Ear and labyrinth disorders
Endocrine disorders
Eye disorders
Gastrointestinal disorders
General disorders and administration site conditions
Hepatobiliary disorders
Immune system disorders
Infections and infestations
Injury, poisoning, and procedural complications
Investigations
Metabolism and nutrition disorders
Musculoskeletal and connective tissue disorders
Neoplasms benign, malignant, and unspecified (incl cysts and polyps)
Nervous system disorders
Psychiatric disorders
Renal and urinary disorders
Reproductive system and breast disorders
Respiratory, thoracic, and mediastinal disorders
Skin and subcutaneous tissue disorders
Social circumstances
Surgical and medical procedures
Vascular disorders

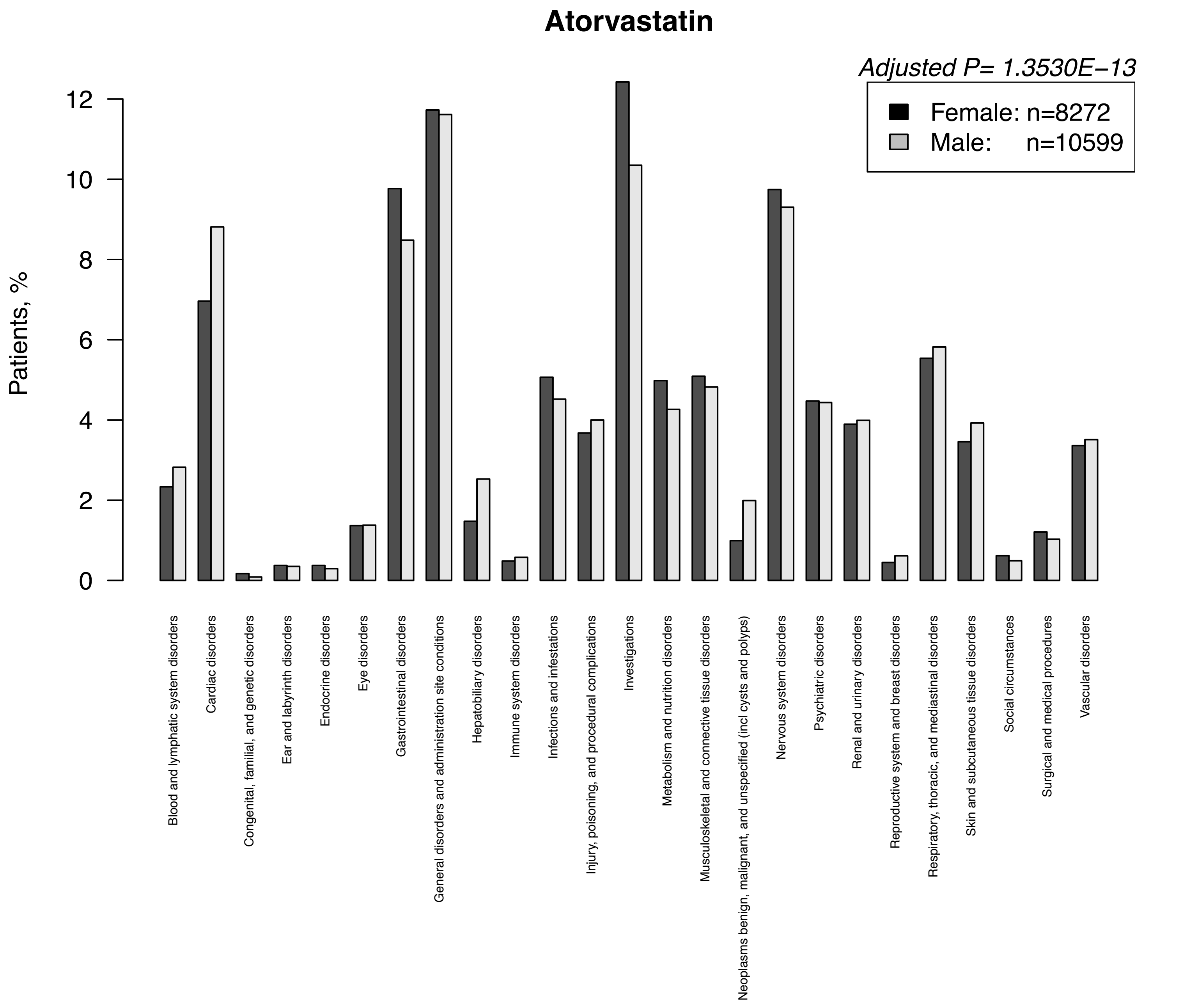

Atorvastatin
Adjusted P= 1.3530E−13
Female: n=8272
Male: n=10599
Patients, %
0
2
4
6
8
10
12
Blood and lymphatic system disorders
Cardiac disorders
Congenital, familial, and genetic disorders
Ear and labyrinth disorders
Endocrine disorders
Eye disorders
Gastrointestinal disorders
General disorders and administration site conditions
Hepatobiliary disorders
Immune system disorders
Infections and infestations
Injury, poisoning, and procedural complications
Investigations
Metabolism and nutrition disorders
Musculoskeletal and connective tissue disorders
Neoplasms benign, malignant, and unspecified (incl cysts and polyps)
Nervous system disorders
Psychiatric disorders
Renal and urinary disorders
Reproductive system and breast disorders
Respiratory, thoracic, and mediastinal disorders
Skin and subcutaneous tissue disorders
Social circumstances
Surgical and medical procedures
Vascular disorders

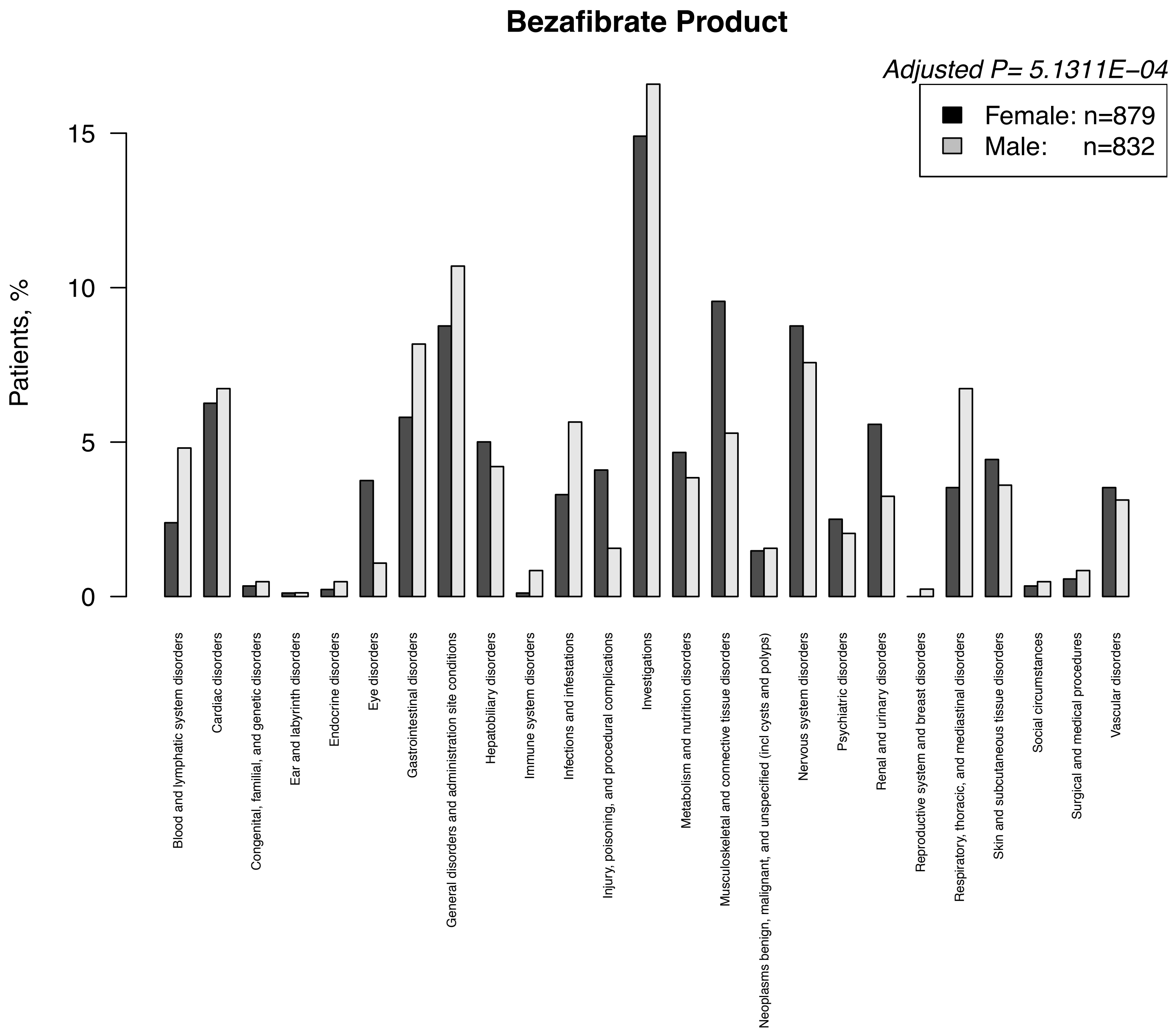

Bezafibrate Product
Adjusted P= 5.1311E−04
Female: n=879
Male: n=832
Patients, %
15
10
5
0
Blood and lymphatic system disorders
Cardiac disorders
Congenital, familial, and genetic disorders
Ear and labyrinth disorders
Endocrine disorders
Eye disorders
Gastrointestinal disorders
General disorders and administration site conditions
Hepatobiliary disorders
Immune system disorders
Infections and infestations
Injury, poisoning, and procedural complications
Investigations
Metabolism and nutrition disorders
Musculoskeletal and connective tissue disorders
Neoplasms benign, malignant, and unspecified (incl cysts and polyps)
Nervous system disorders
Psychiatric disorders
Renal and urinary disorders
Reproductive system and breast disorders
Respiratory, thoracic, and mediastinal disorders
Skin and subcutaneous tissue disorders
Social circumstances
Surgical and medical procedures
Vascular disorders

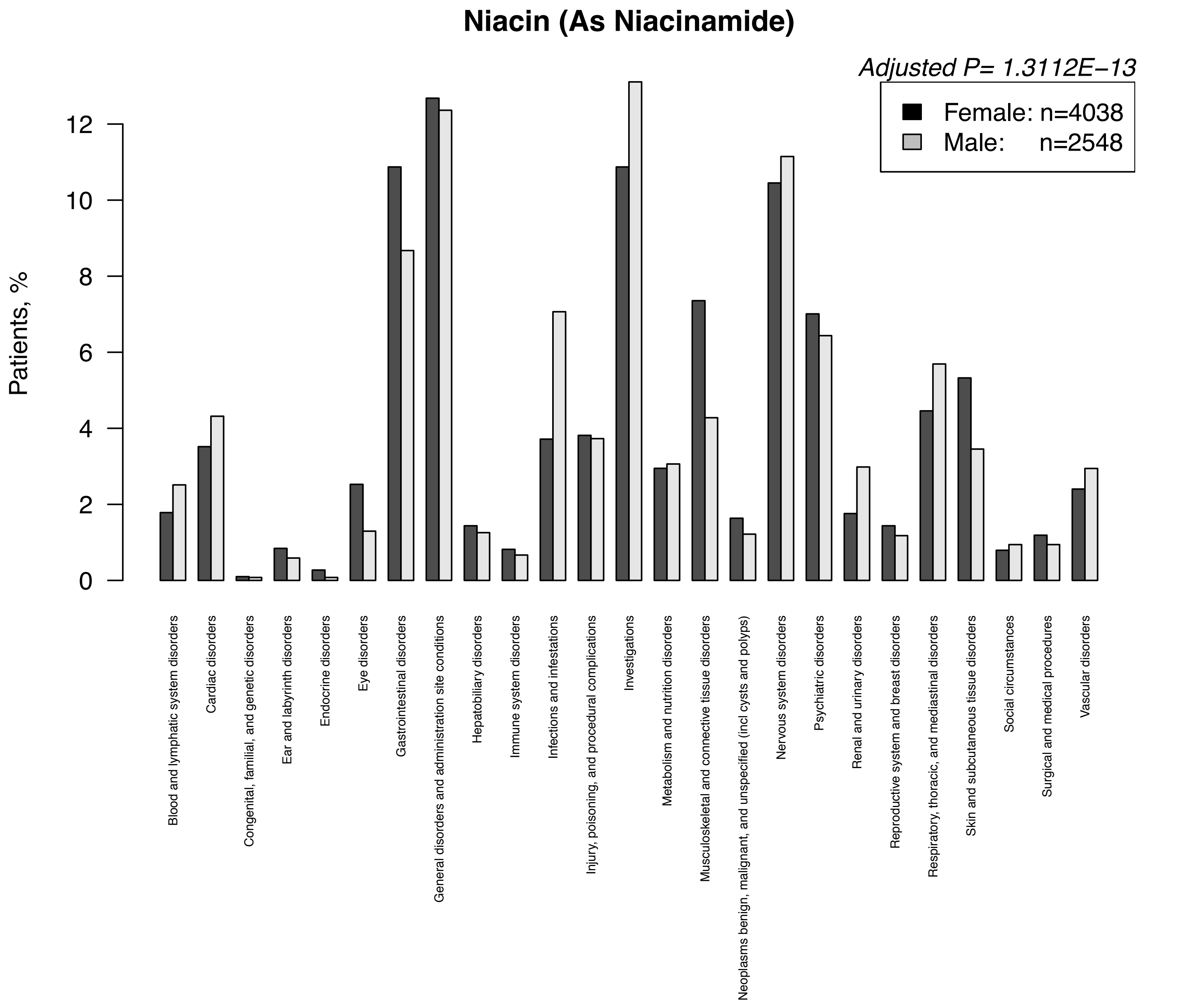

Niacin (As Niacinamide)
Adjusted P= 1.3112E−13
Female: n=4038
Male: n=2548
Patients, %
0
2
4
6
8
10
12
Blood and lymphatic system disorders
Cardiac disorders
Congenital, familial, and genetic disorders
Ear and labyrinth disorders
Endocrine disorders
Eye disorders
Gastrointestinal disorders
General disorders and administration site conditions
Hepatobiliary disorders
Immune system disorders
Infections and infestations
Injury, poisoning, and procedural complications
Investigations
Metabolism and nutrition disorders
Musculoskeletal and connective tissue disorders
Neoplasms benign, malignant, and unspecified (incl cysts and polyps)
Nervous system disorders
Psychiatric disorders
Renal and urinary disorders
Reproductive system and breast disorders
Respiratory, thoracic, and mediastinal disorders
Skin and subcutaneous tissue disorders
Social circumstances
Surgical and medical procedures
Vascular disorders

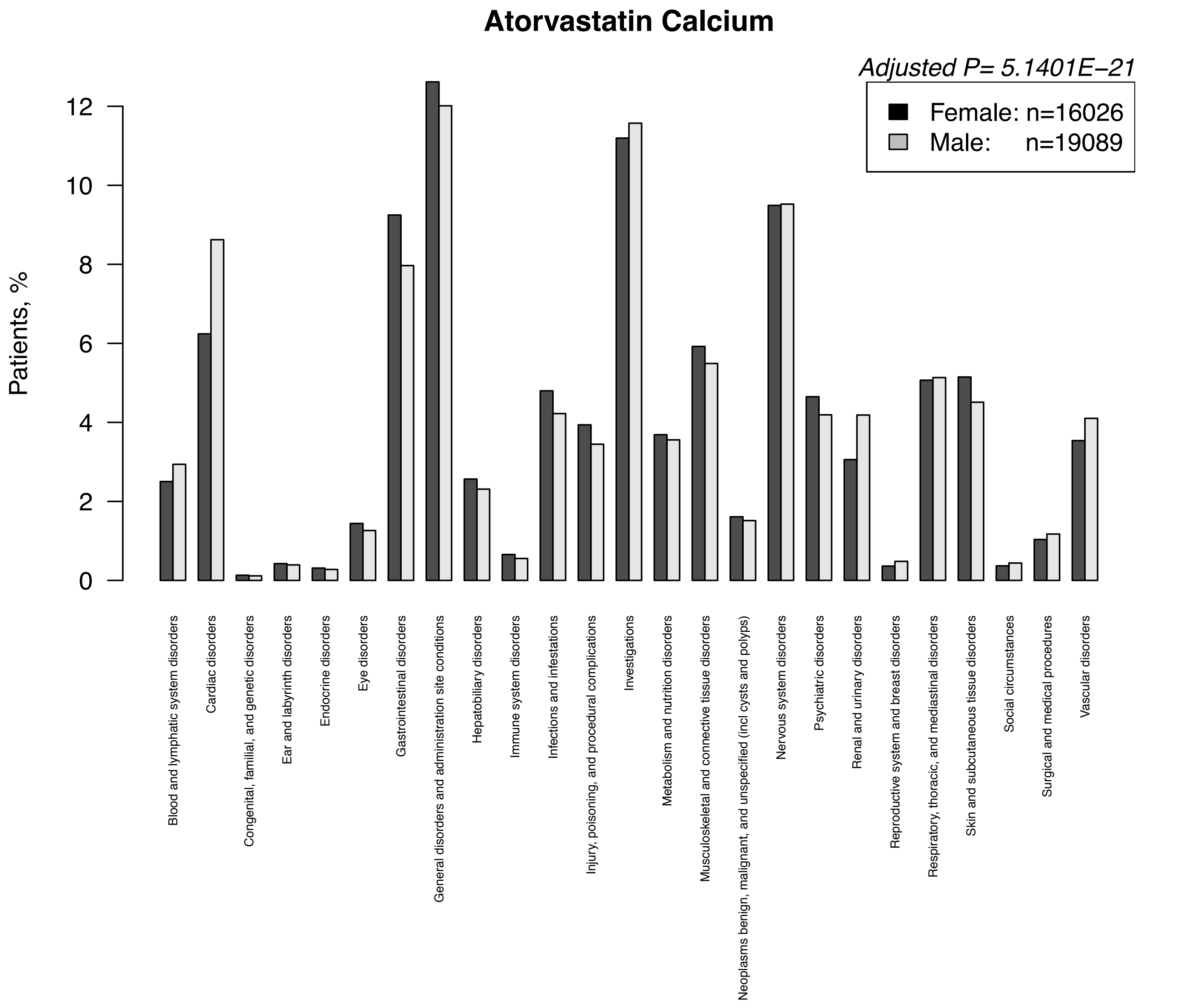

Atorvastatin Calcium
Adjusted P= 5.1401E−21
Female: n=16026
Male: n=19089
Patients, %
0
2
4
6
8
10
12
Blood and lymphatic system disorders
Cardiac disorders
Congenital, familial, and genetic disorders
Ear and labyrinth disorders
Endocrine disorders
Eye disorders
Gastrointestinal disorders
General disorders and administration site conditions
Hepatobiliary disorders
Immune system disorders
Infections and infestations
Injury, poisoning, and procedural complications
Investigations
Metabolism and nutrition disorders
Musculoskeletal and connective tissue disorders
Neoplasms benign, malignant, and unspecified (incl cysts and polyps)
Nervous system disorders
Psychiatric disorders
Renal and urinary disorders
Reproductive system and breast disorders
Respiratory, thoracic, and mediastinal disorders
Skin and subcutaneous tissue disorders
Social circumstances
Surgical and medical procedures
Vascular disorders

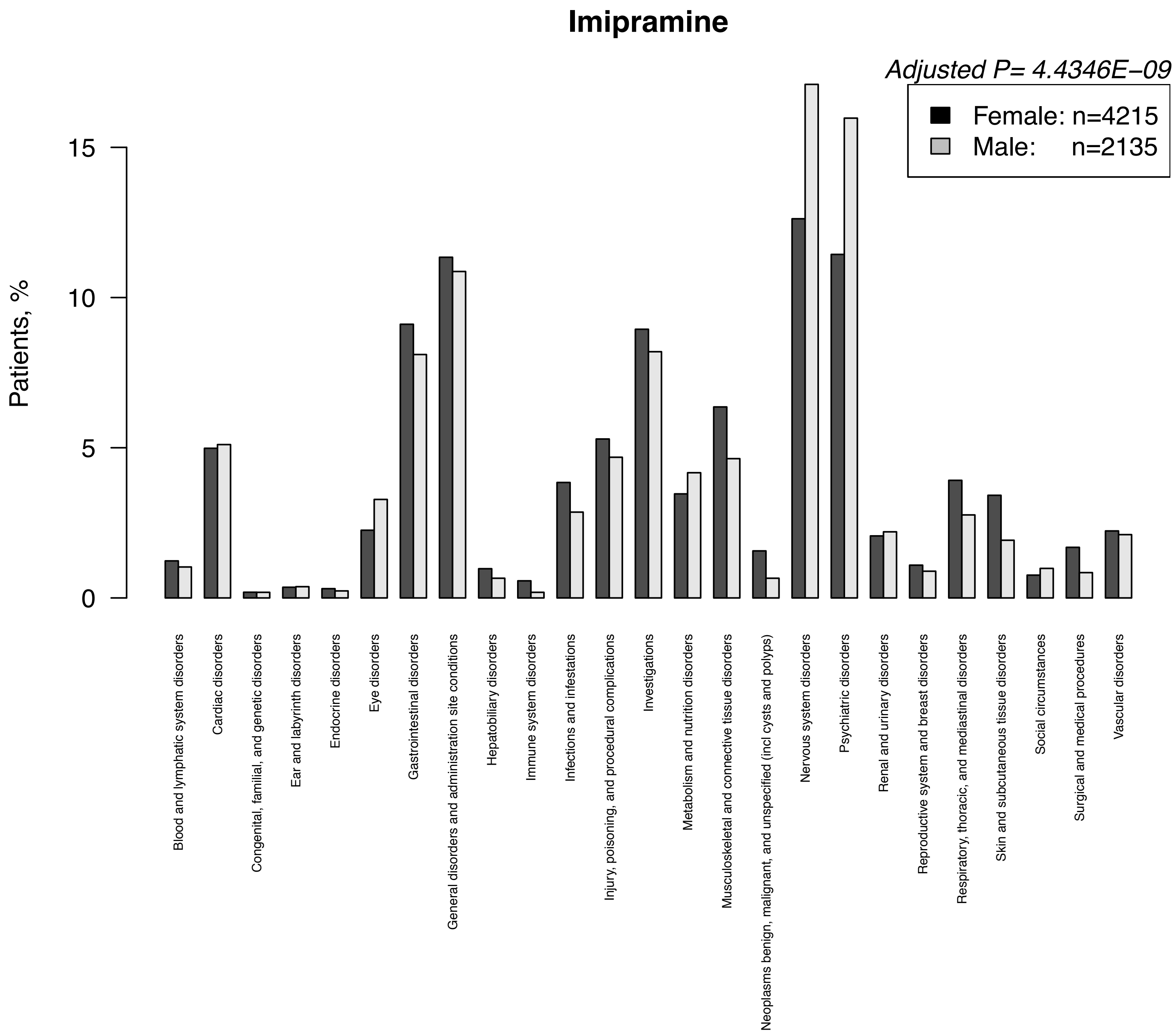

Imipramine
Adjusted P= 4.4346E−09
Female: n=4215
Male: n=2135
Patients, %
0
5
10
15
Blood and lymphatic system disorders
Cardiac disorders
Congenital, familial, and genetic disorders
Ear and labyrinth disorders
Endocrine disorders
Eye disorders
Gastrointestinal disorders
General disorders and administration site conditions
Hepatobiliary disorders
Immune system disorders
Infections and infestations
Injury, poisoning, and procedural complications
Investigations
Metabolism and nutrition disorders
Musculoskeletal and connective tissue disorders
Neoplasms benign, malignant, and unspecified (incl cysts and polyps)
Nervous system disorders
Psychiatric disorders
Renal and urinary disorders
Reproductive system and breast disorders
Respiratory, thoracic, and mediastinal disorders
Skin and subcutaneous tissue disorders
Social circumstances
Surgical and medical procedures
Vascular disorders

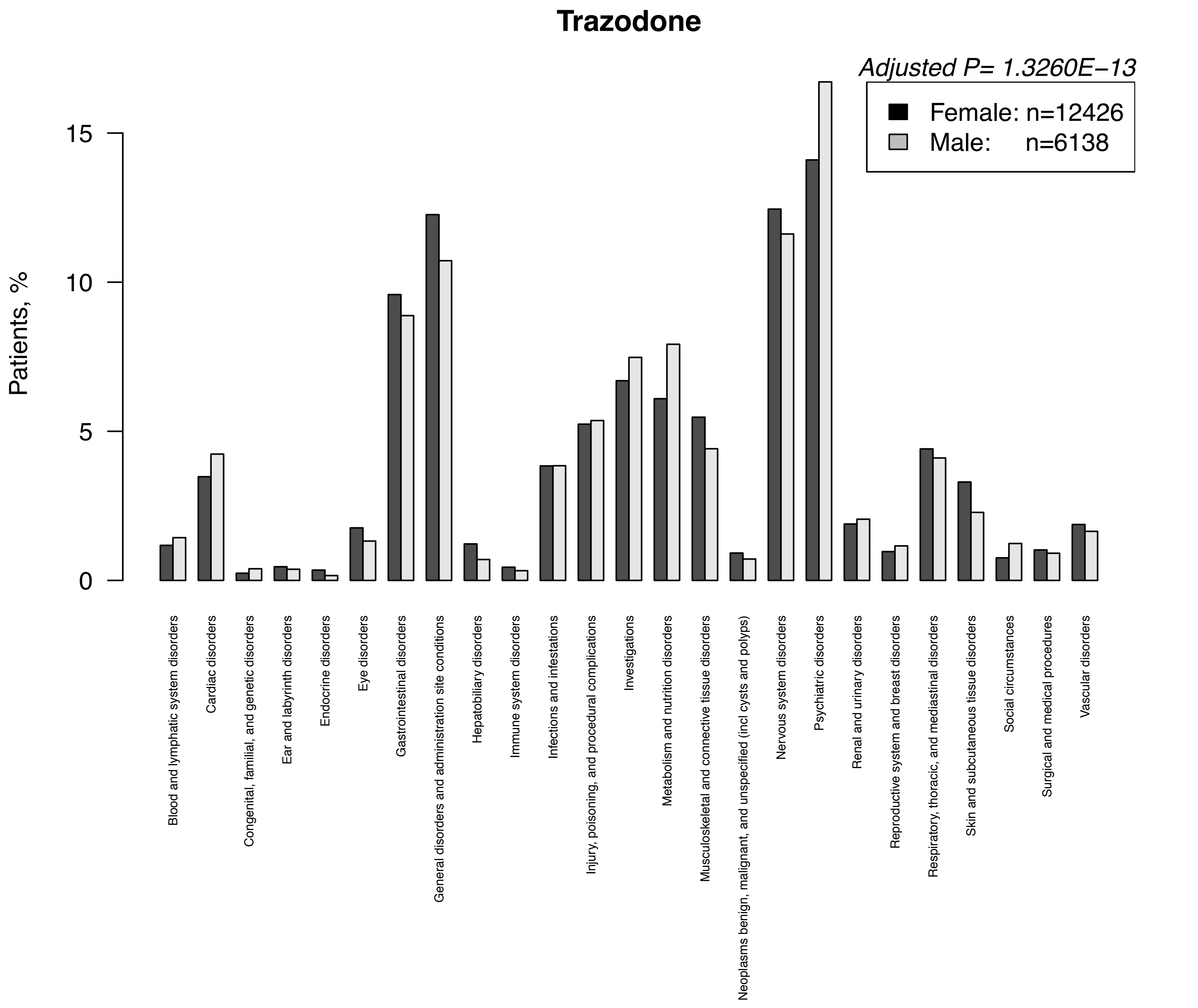
Trazodone
Adjusted P= 1.3260E−13
Female: n=12426
Male: n=6138
Patients, %
0
5
10
15
Blood and lymphatic system disorders
Cardiac disorders
Congenital, familial, and genetic disorders
Ear and labyrinth disorders
Endocrine disorders
Eye disorders
Gastrointestinal disorders
General disorders and administration site conditions
Hepatobiliary disorders
Immune system disorders
Infections and infestations
Injury, poisoning, and procedural complications
Investigations
Metabolism and nutrition disorders
Musculoskeletal and connective tissue disorders
Neoplasms benign, malignant, and unspecified (incl cysts and polyps)
Nervous system disorders
Psychiatric disorders
Renal and urinary disorders
Reproductive system and breast disorders
Respiratory, thoracic, and mediastinal disorders
Skin and subcutaneous tissue disorders
Social circumstances
Surgical and medical procedures
Vascular disorders

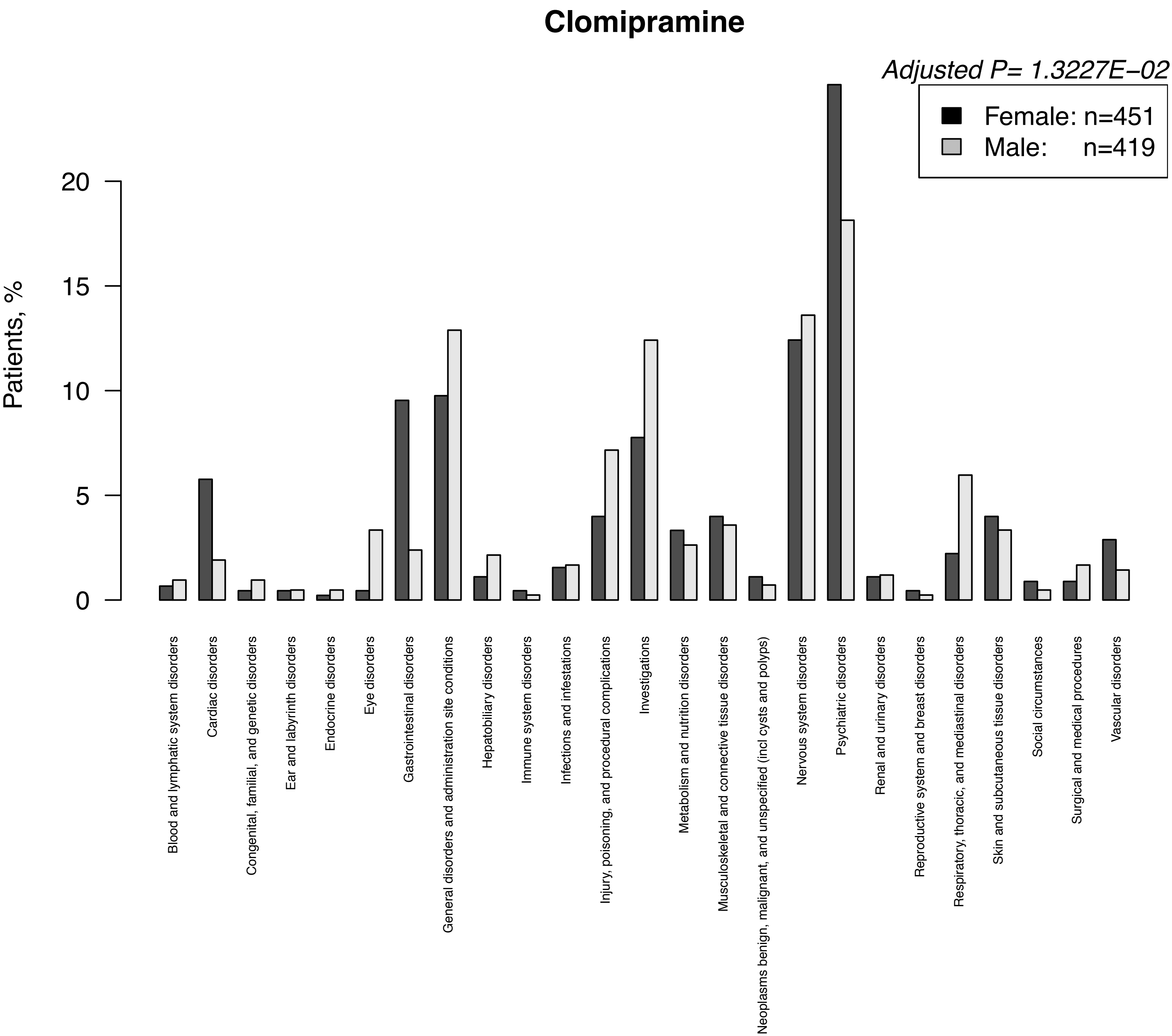

Clomipramine
Adjusted P= 1.3227E−02
Female: n=451
Male: n=419
Patients, %
0
5
10
15
20
Blood and lymphatic system disorders
Cardiac disorders
Congenital, familial, and genetic disorders
Ear and labyrinth disorders
Endocrine disorders
Eye disorders
Gastrointestinal disorders
General disorders and administration site conditions
Hepatobiliary disorders
Immune system disorders
Infections and infestations
Injury, poisoning, and procedural complications
Investigations
Metabolism and nutrition disorders
Musculoskeletal and connective tissue disorders
Neoplasms benign, malignant, and unspecified (incl cysts and polyps)
Nervous system disorders
Psychiatric disorders
Renal and urinary disorders
Reproductive system and breast disorders
Respiratory, thoracic, and mediastinal disorders
Skin and subcutaneous tissue disorders
Social circumstances
Surgical and medical procedures
Vascular disorders

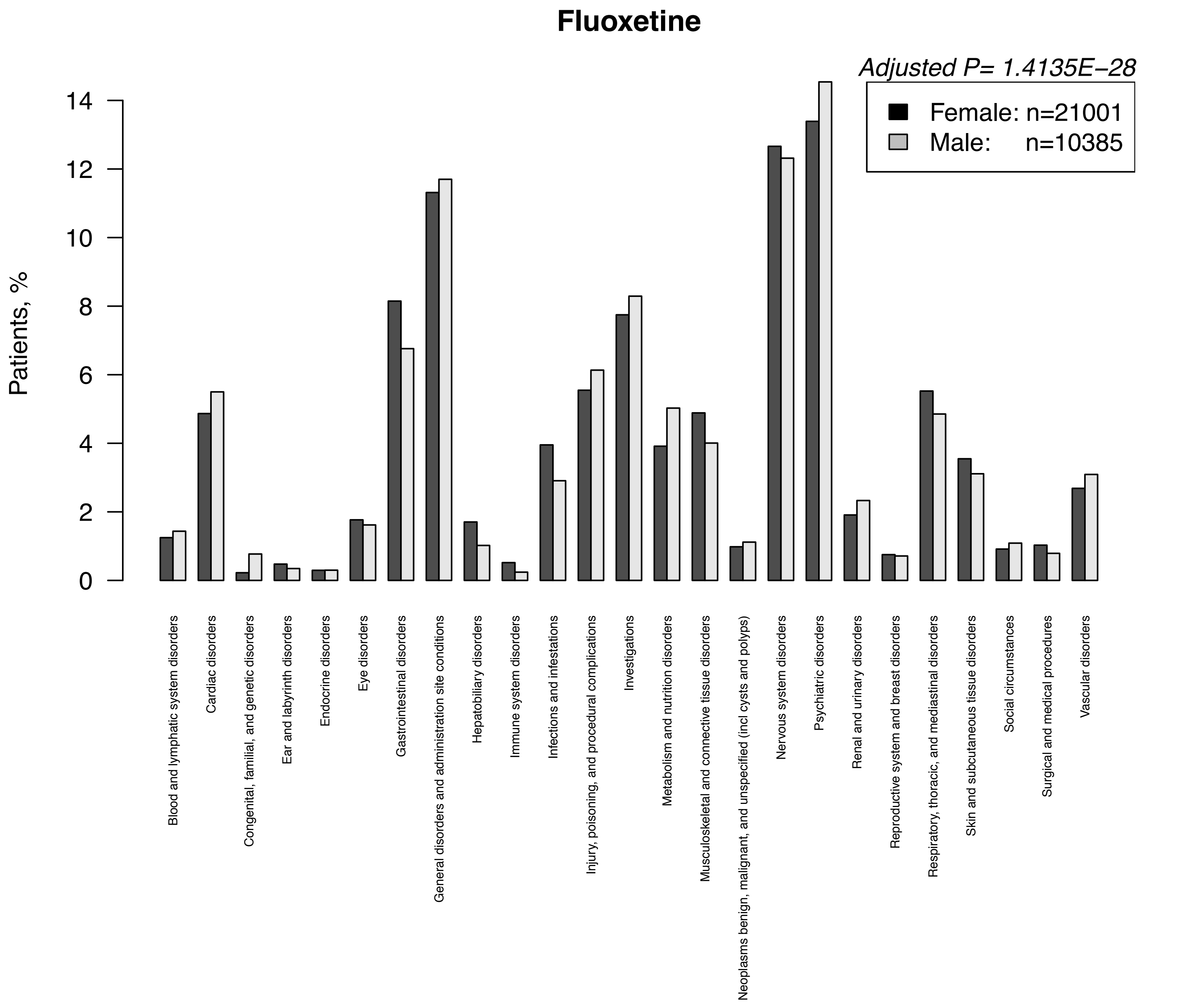
Fluoxetine
Adjusted P= 1.4135E−28
Female: n=21001
Male: n=10385
Patients, %
0
2
4
6
8
10
12
14
Blood and lymphatic system disorders
Cardiac disorders
Congenital, familial, and genetic disorders
Ear and labyrinth disorders
Endocrine disorders
Eye disorders
Gastrointestinal disorders
General disorders and administration site conditions
Hepatobiliary disorders
Immune system disorders
Infections and infestations
Injury, poisoning, and procedural complications
Investigations
Metabolism and nutrition disorders
Musculoskeletal and connective tissue disorders
Neoplasms benign, malignant, and unspecified (incl cysts and polyps)
Nervous system disorders
Psychiatric disorders
Renal and urinary disorders
Reproductive system and breast disorders
Respiratory, thoracic, and mediastinal disorders
Skin and subcutaneous tissue disorders
Social circumstances
Surgical and medical procedures
Vascular disorders

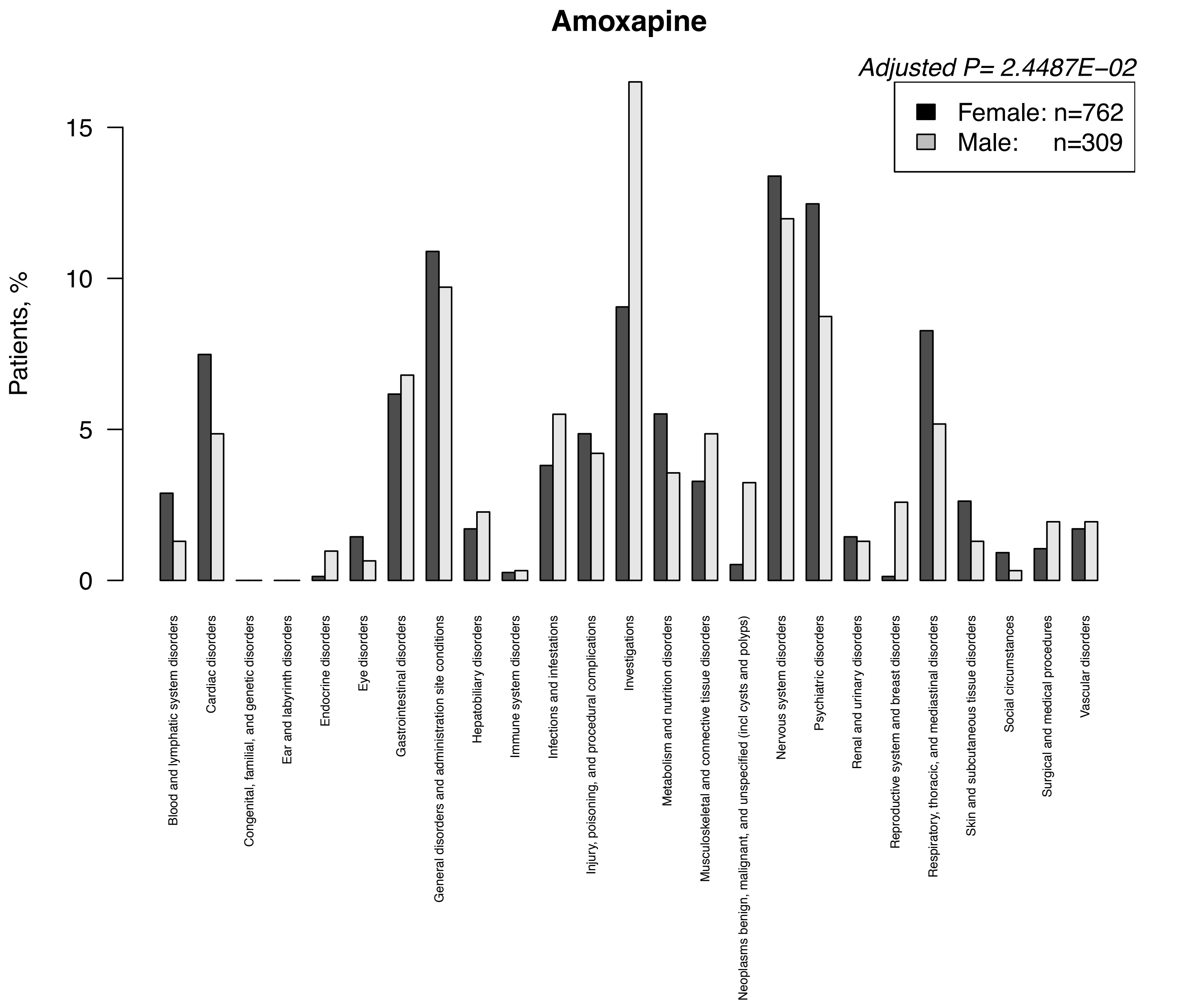
Amoxapine
Adjusted P= 2.4487E−02
Female: n=762
Male: n=309
Patients, %
0
5
10
15
Blood and lymphatic system disorders
Cardiac disorders
Congenital, familial, and genetic disorders
Ear and labyrinth disorders
Endocrine disorders
Eye disorders
Gastrointestinal disorders
General disorders and administration site conditions
Hepatobiliary disorders
Immune system disorders
Infections and infestations
Injury, poisoning, and procedural complications
Investigations
Metabolism and nutrition disorders
Musculoskeletal and connective tissue disorders
Neoplasms benign, malignant, and unspecified (incl cysts and polyps)
Nervous system disorders
Psychiatric disorders
Renal and urinary disorders
Reproductive system and breast disorders
Respiratory, thoracic, and mediastinal disorders
Skin and subcutaneous tissue disorders
Social circumstances
Surgical and medical procedures
Vascular disorders

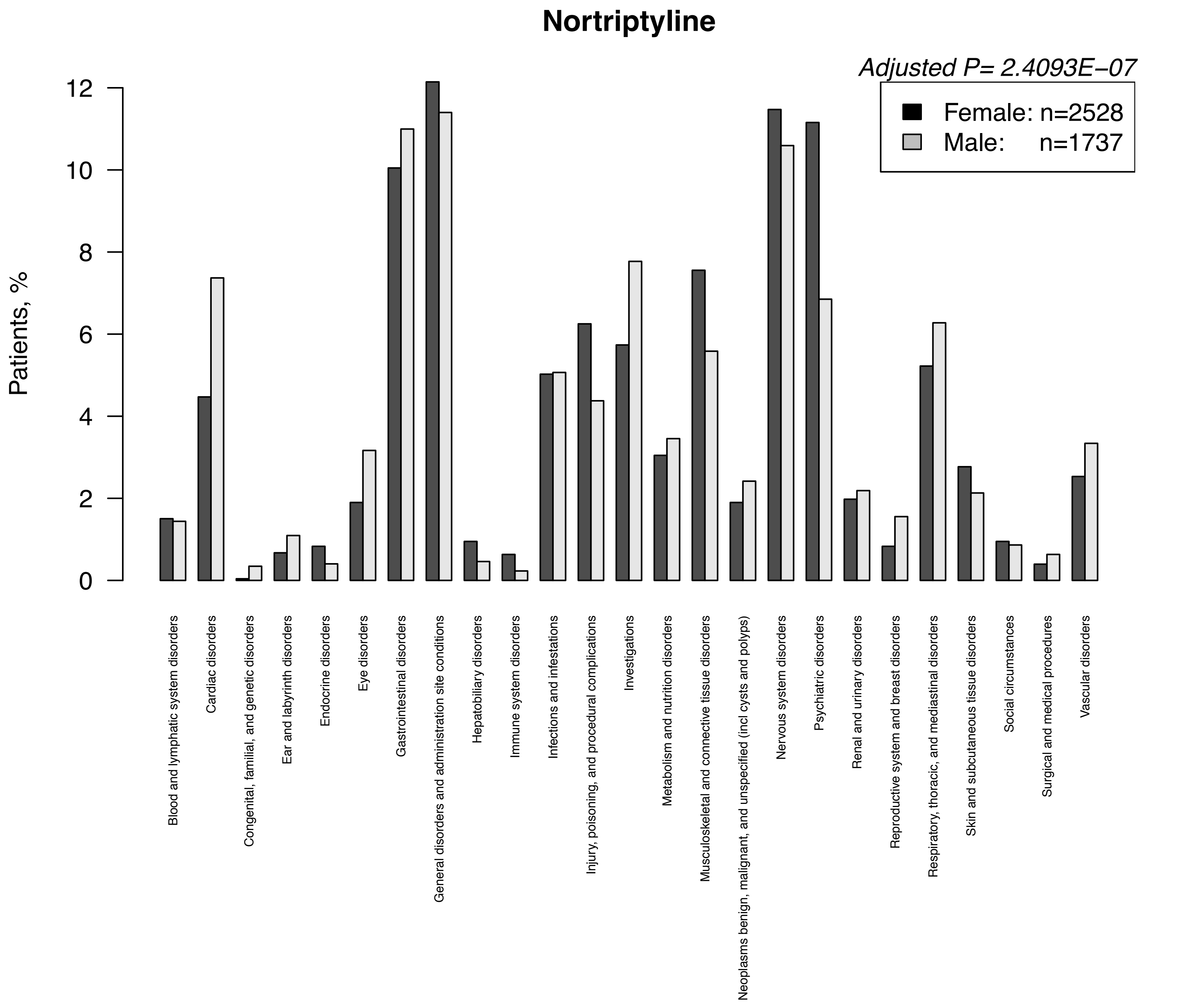
Nortriptyline
Adjusted P= 2.4093E−07
Female: n=2528
Male: n=1737
Patients, %
0
2
4
6
8
10
12
Blood and lymphatic system disorders
Cardiac disorders
Congenital, familial, and genetic disorders
Ear and labyrinth disorders
Endocrine disorders
Eye disorders
Gastrointestinal disorders
General disorders and administration site conditions
Hepatobiliary disorders
Immune system disorders
Infections and infestations
Injury, poisoning, and procedural complications
Investigations
Metabolism and nutrition disorders
Musculoskeletal and connective tissue disorders
Neoplasms benign, malignant, and unspecified (incl cysts and polyps)
Nervous system disorders
Psychiatric disorders
Renal and urinary disorders
Reproductive system and breast disorders
Respiratory, thoracic, and mediastinal disorders
Skin and subcutaneous tissue disorders
Social circumstances
Surgical and medical procedures
Vascular disorders

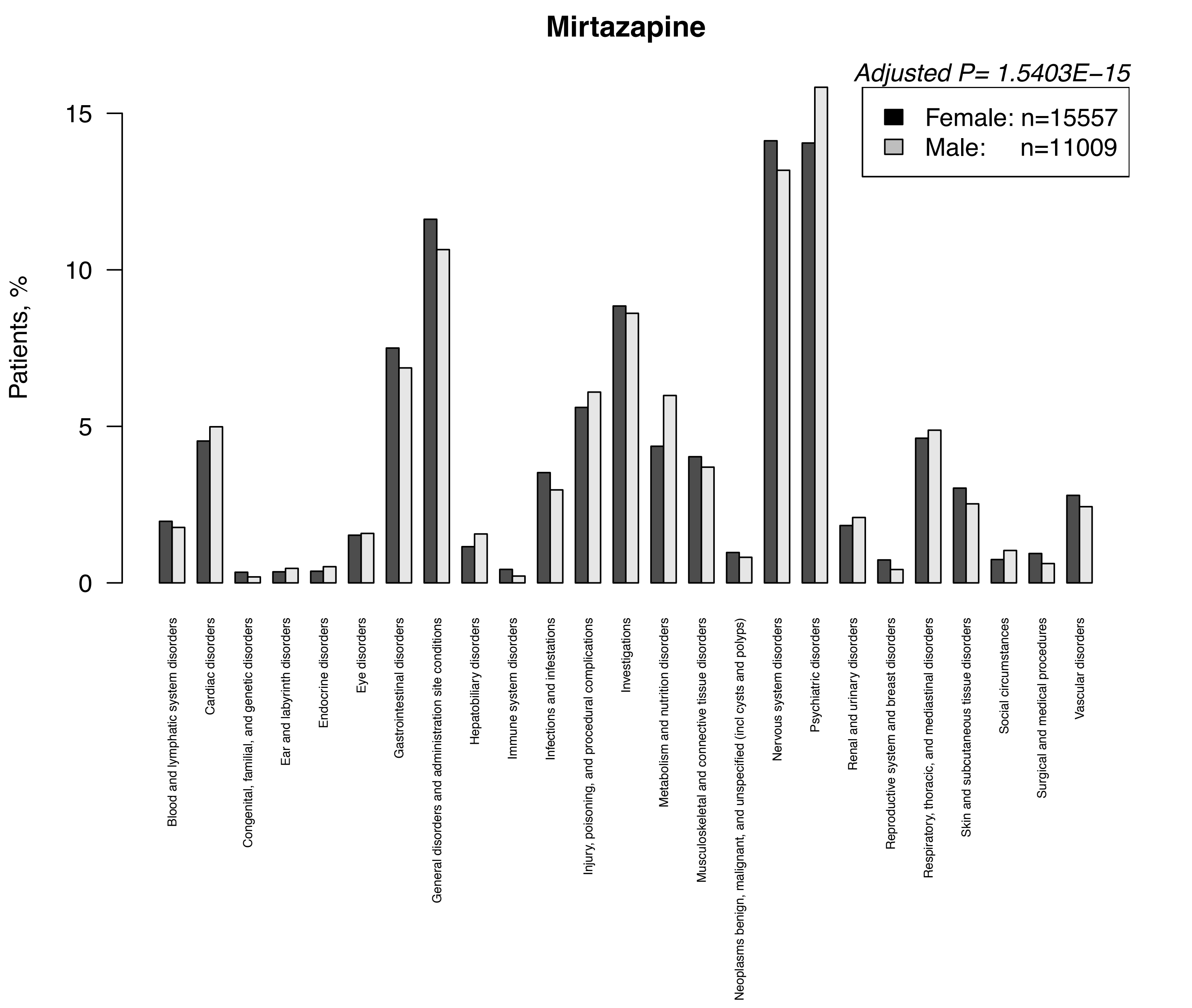

Mirtazapine
Adjusted P= 1.5403E−15
Female: n=15557
Male: n=11009
Patients, %
0
5
10
15
Blood and lymphatic system disorders
Cardiac disorders
Congenital, familial, and genetic disorders
Ear and labyrinth disorders
Endocrine disorders
Eye disorders
Gastrointestinal disorders
General disorders and administration site conditions
Hepatobiliary disorders
Immune system disorders
Infections and infestations
Injury, poisoning, and procedural complications
Investigations
Metabolism and nutrition disorders
Musculoskeletal and connective tissue disorders
Neoplasms benign, malignant, and unspecified (incl cysts and polyps)
Nervous system disorders
Psychiatric disorders
Renal and urinary disorders
Reproductive system and breast disorders
Respiratory, thoracic, and mediastinal disorders
Skin and subcutaneous tissue disorders
Social circumstances
Surgical and medical procedures
Vascular disorders

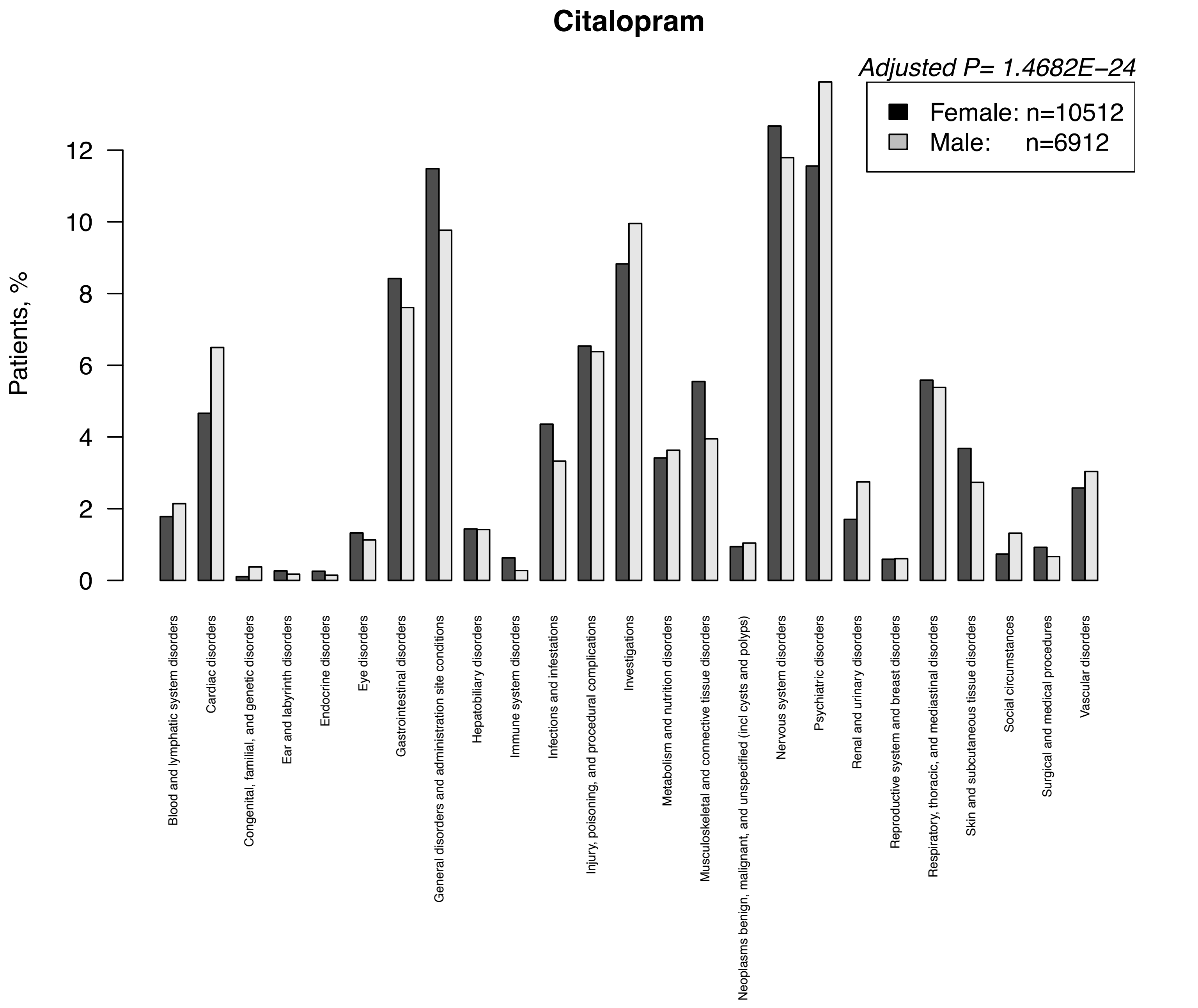
Citalopram
Adjusted P= 1.4682E−24
Female: n=10512
Male: n=6912
Patients, %
0
2
4
6
8
10
12
Blood and lymphatic system disorders
Cardiac disorders
Congenital, familial, and genetic disorders
Ear and labyrinth disorders
Endocrine disorders
Eye disorders
Gastrointestinal disorders
General disorders and administration site conditions
Hepatobiliary disorders
Immune system disorders
Infections and infestations
Injury, poisoning, and procedural complications
Investigations
Metabolism and nutrition disorders
Musculoskeletal and connective tissue disorders
Neoplasms benign, malignant, and unspecified (incl cysts and polyps)
Nervous system disorders
Psychiatric disorders
Renal and urinary disorders
Reproductive system and breast disorders
Respiratory, thoracic, and mediastinal disorders
Skin and subcutaneous tissue disorders
Social circumstances
Surgical and medical procedures
Vascular disorders

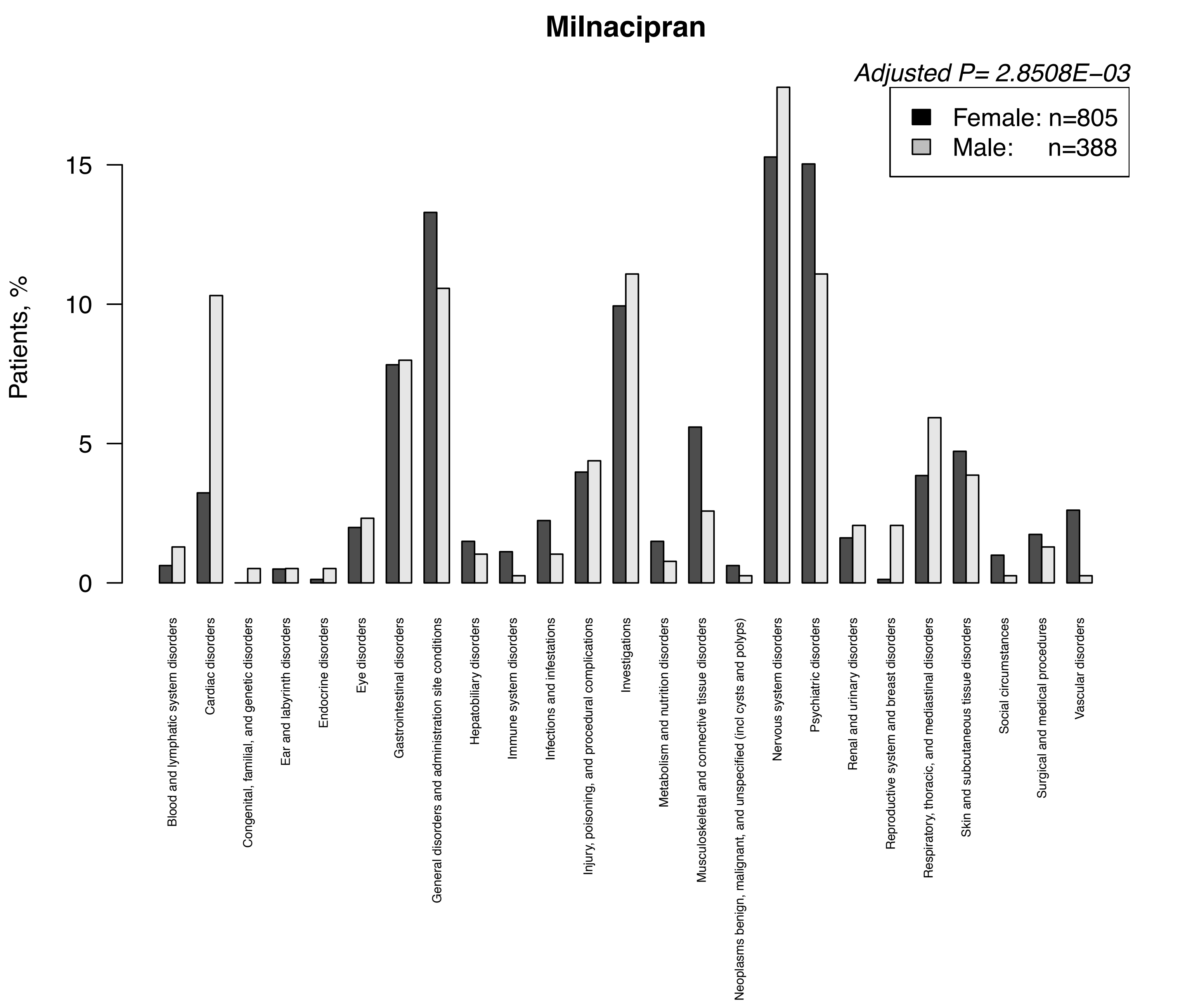

Milnacipran
Adjusted P= 2.8508E−03
Female: n=805
Male: n=388
Patients, %
0
5
10
15
Blood and lymphatic system disorders
Cardiac disorders
Congenital, familial, and genetic disorders
Ear and labyrinth disorders
Endocrine disorders
Eye disorders
Gastrointestinal disorders
General disorders and administration site conditions
Hepatobiliary disorders
Immune system disorders
Infections and infestations
Injury, poisoning, and procedural complications
Investigations
Metabolism and nutrition disorders
Musculoskeletal and connective tissue disorders
Neoplasms benign, malignant, and unspecified (incl cysts and polyps)
Nervous system disorders
Psychiatric disorders
Renal and urinary disorders
Reproductive system and breast disorders
Respiratory, thoracic, and mediastinal disorders
Skin and subcutaneous tissue disorders
Social circumstances
Surgical and medical procedures
Vascular disorders

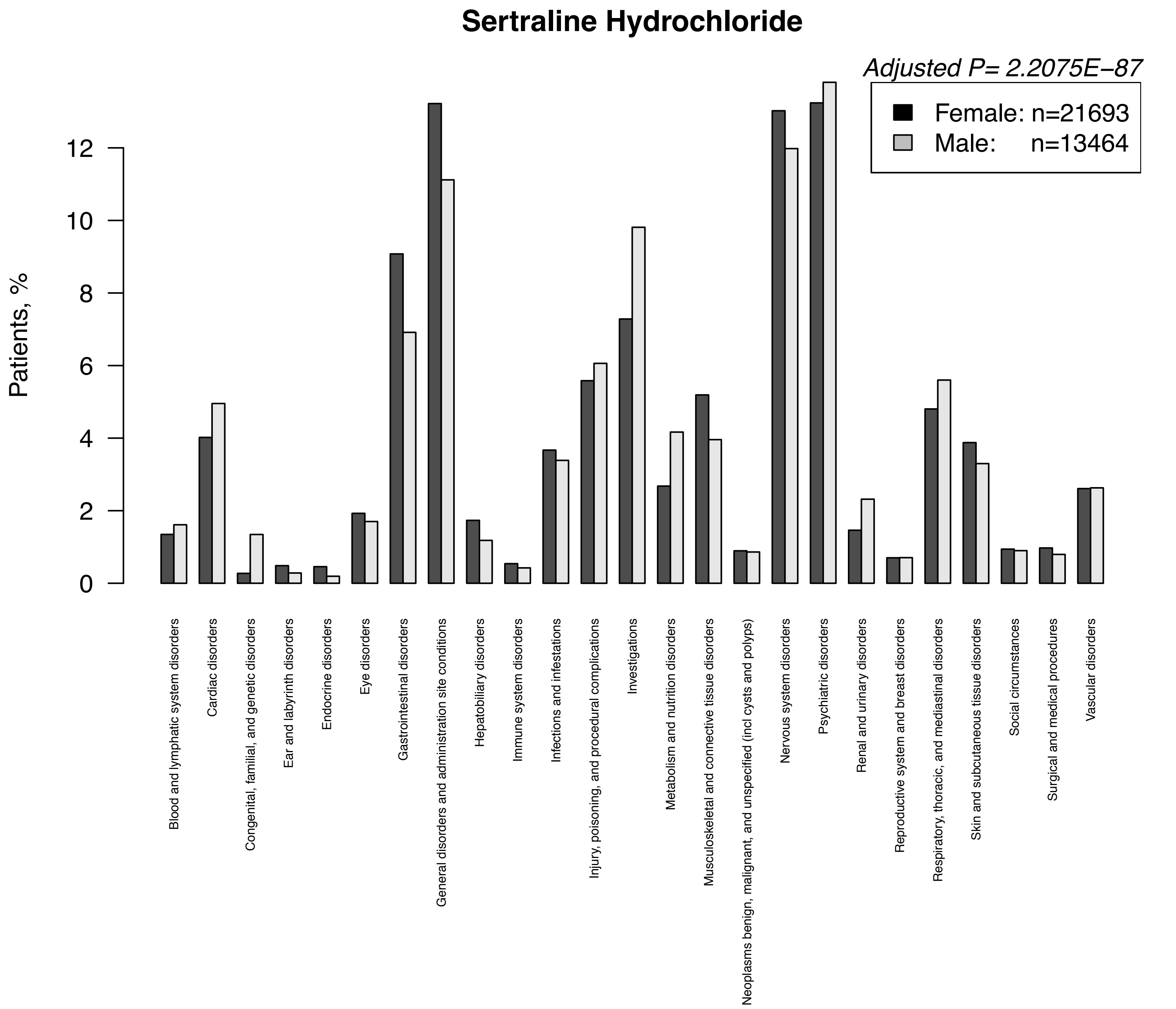
Sertraline Hydrochloride
Adjusted P= 2.2075E−87
Female: n=21693
Male: n=13464
Patients, %
0
2
4
6
8
10
12
Blood and lymphatic system disorders
Cardiac disorders
Congenital, familial, and genetic disorders
Ear and labyrinth disorders
Endocrine disorders
Eye disorders
Gastrointestinal disorders
General disorders and administration site conditions
Hepatobiliary disorders
Immune system disorders
Infections and infestations
Injury, poisoning, and procedural complications
Investigations
Metabolism and nutrition disorders
Musculoskeletal and connective tissue disorders
Neoplasms benign, malignant, and unspecified (incl cysts and polyps)
Nervous system disorders
Psychiatric disorders
Renal and urinary disorders
Reproductive system and breast disorders
Respiratory, thoracic, and mediastinal disorders
Skin and subcutaneous tissue disorders
Social circumstances
Surgical and medical procedures
Vascular disorders

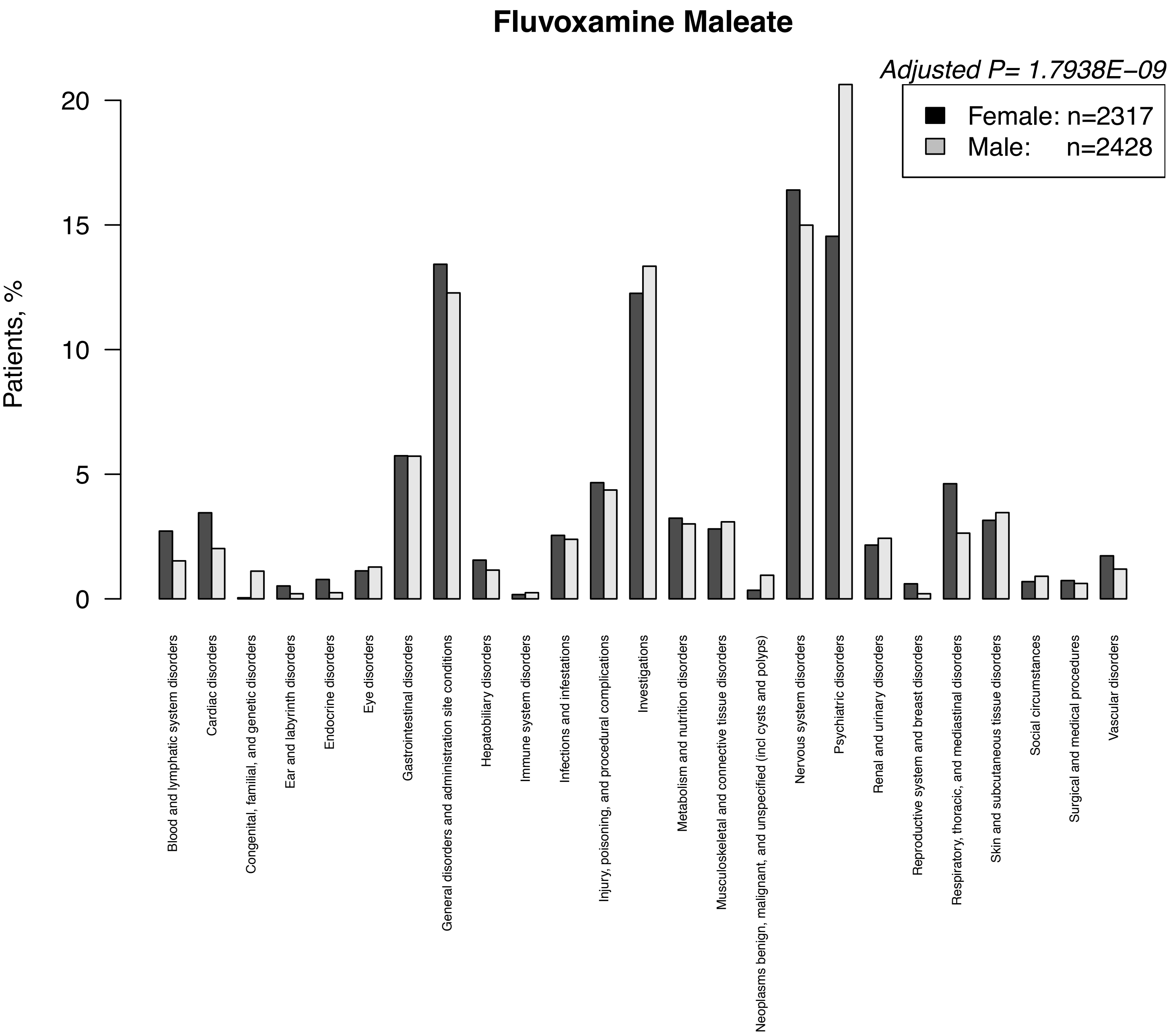

Fluvoxamine Maleate
Adjusted P= 1.7938E−09
Female: n=2317
Male: n=2428
Patients, %
0
5
10
15
20
Blood and lymphatic system disorders
Cardiac disorders
Congenital, familial, and genetic disorders
Ear and labyrinth disorders
Endocrine disorders
Eye disorders
Gastrointestinal disorders
General disorders and administration site conditions
Hepatobiliary disorders
Immune system disorders
Infections and infestations
Injury, poisoning, and procedural complications
Investigations
Metabolism and nutrition disorders
Musculoskeletal and connective tissue disorders
Neoplasms benign, malignant, and unspecified (incl cysts and polyps)
Nervous system disorders
Psychiatric disorders
Renal and urinary disorders
Reproductive system and breast disorders
Respiratory, thoracic, and mediastinal disorders
Skin and subcutaneous tissue disorders
Social circumstances
Surgical and medical procedures
Vascular disorders

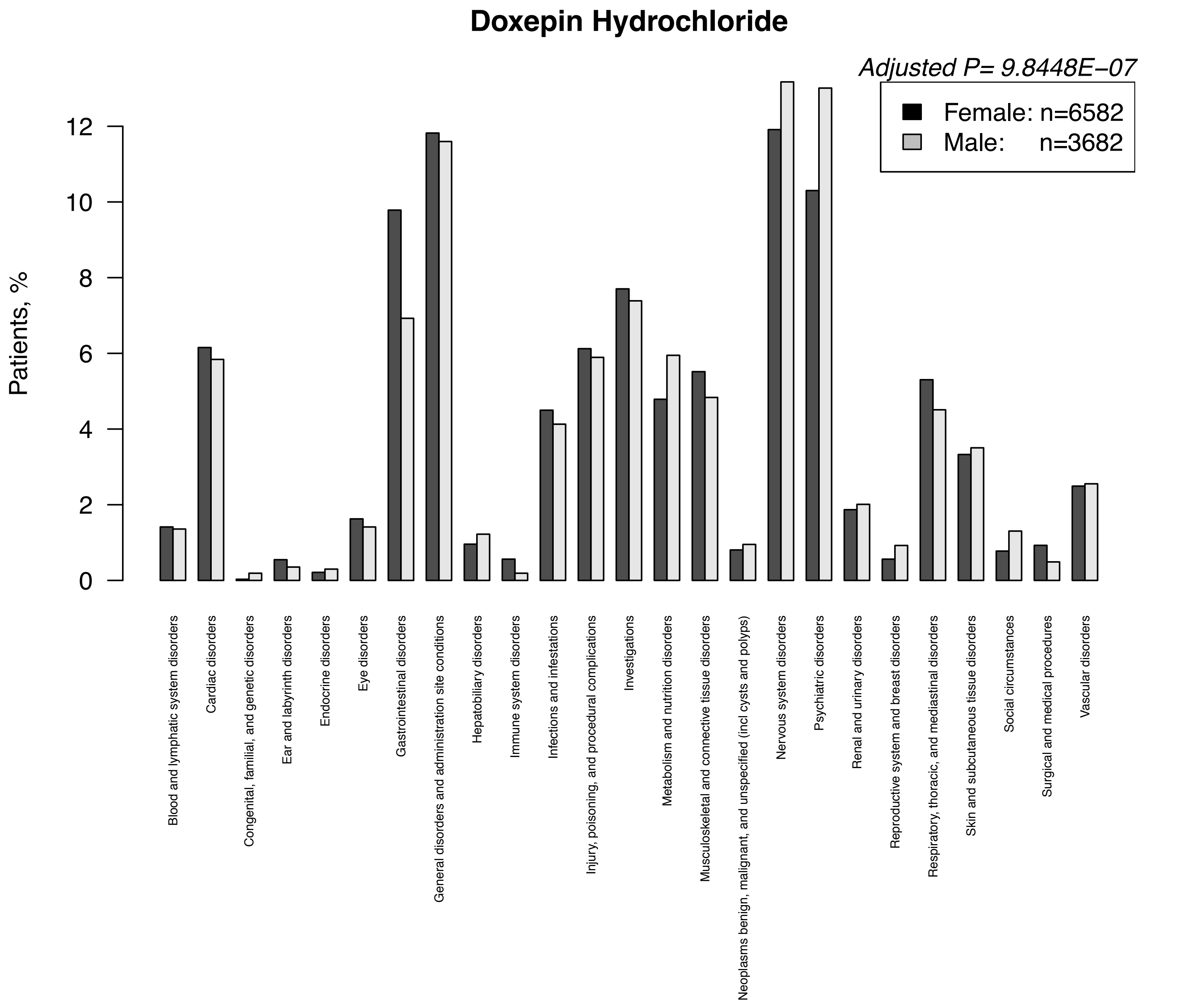
Doxepin Hydrochloride
Adjusted P= 9.8448E−07
Female: n=6582
Male: n=3682
Patients, %
0
2
4
6
8
10
12
Blood and lymphatic system disorders
Cardiac disorders
Congenital, familial, and genetic disorders
Ear and labyrinth disorders
Endocrine disorders
Eye disorders
Gastrointestinal disorders
General disorders and administration site conditions
Hepatobiliary disorders
Immune system disorders
Infections and infestations
Injury, poisoning, and procedural complications
Investigations
Metabolism and nutrition disorders
Musculoskeletal and connective tissue disorders
Neoplasms benign, malignant, and unspecified (incl cysts and polyps)
Nervous system disorders
Psychiatric disorders
Renal and urinary disorders
Reproductive system and breast disorders
Respiratory, thoracic, and mediastinal disorders
Skin and subcutaneous tissue disorders
Social circumstances
Surgical and medical procedures
Vascular disorders

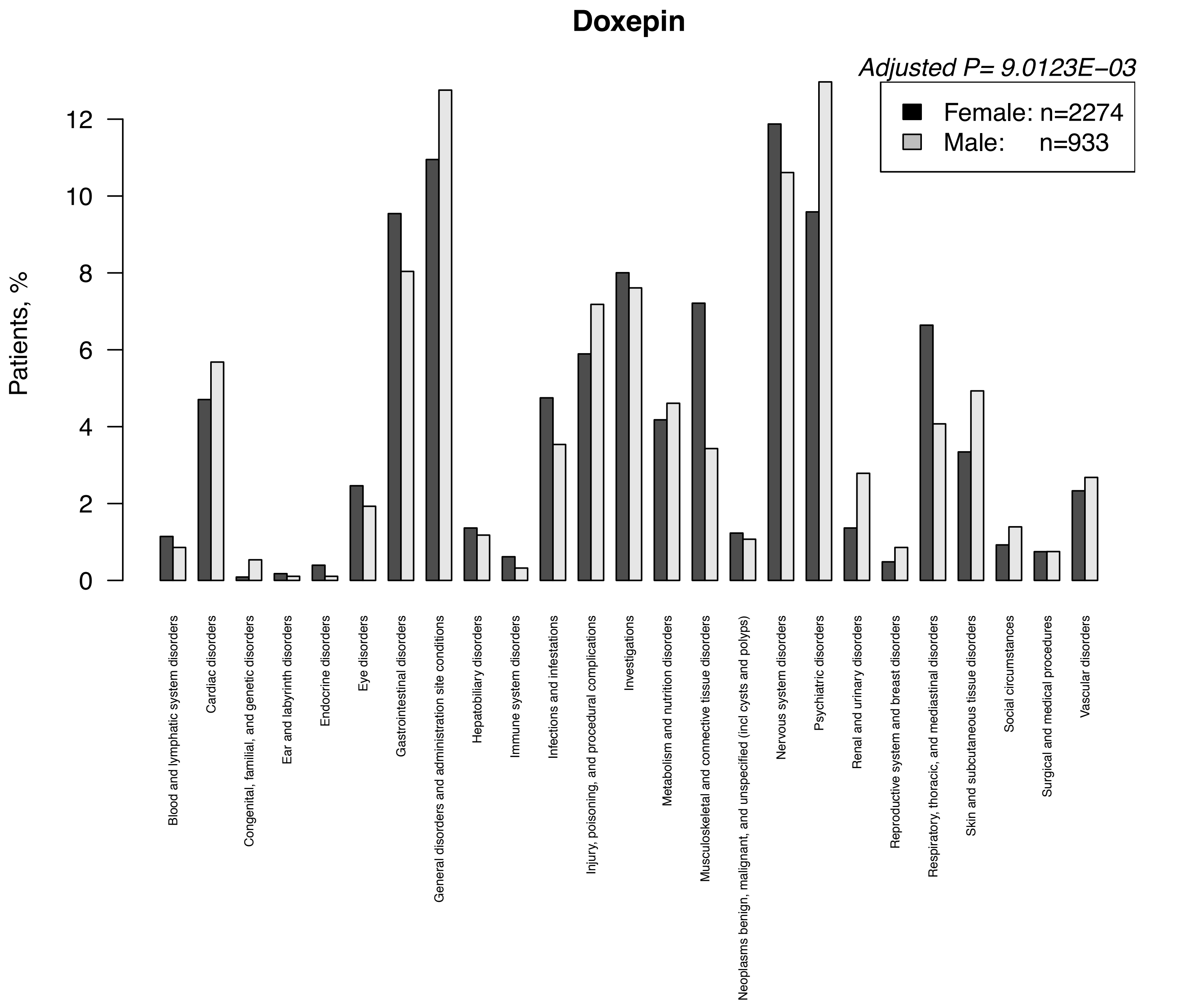
Doxepin
Adjusted P= 9.0123E−03
Female: n=2274
Male: n=933
Patients, %
0
2
4
6
8
10
12
Blood and lymphatic system disorders
Cardiac disorders
Congenital, familial, and genetic disorders
Ear and labyrinth disorders
Endocrine disorders
Eye disorders
Gastrointestinal disorders
General disorders and administration site conditions
Hepatobiliary disorders
Immune system disorders
Infections and infestations
Injury, poisoning, and procedural complications
Investigations
Metabolism and nutrition disorders
Musculoskeletal and connective tissue disorders
Neoplasms benign, malignant, and unspecified (incl cysts and polyps)
Nervous system disorders
Psychiatric disorders
Renal and urinary disorders
Reproductive system and breast disorders
Respiratory, thoracic, and mediastinal disorders
Skin and subcutaneous tissue disorders
Social circumstances
Surgical and medical procedures
Vascular disorders

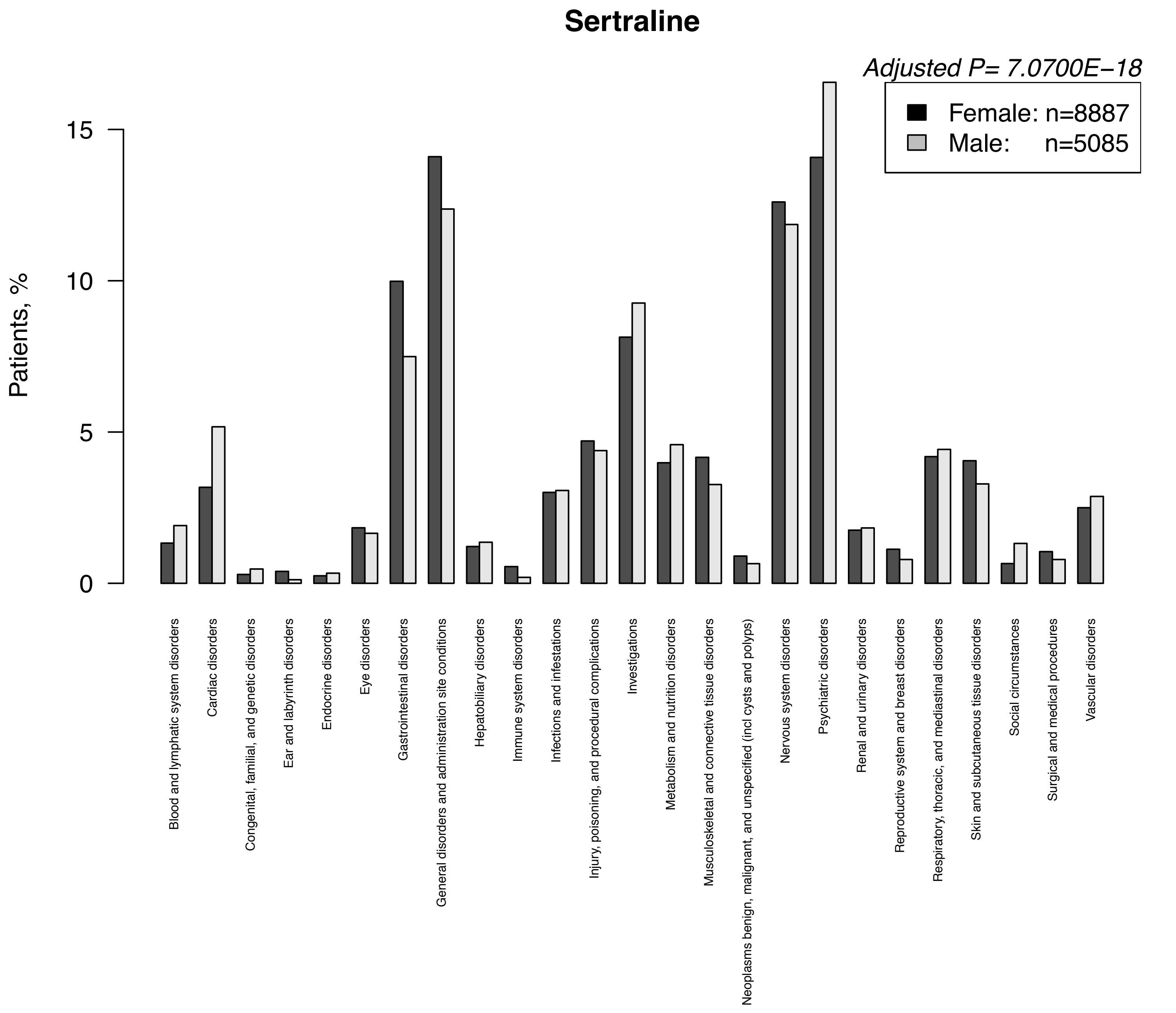

Sertraline
Adjusted P= 7.0700E−18
Female: n=8887
Male: n=5085
Patients, %
0
5
10
15
Blood and lymphatic system disorders
Cardiac disorders
Congenital, familial, and genetic disorders
Ear and labyrinth disorders
Endocrine disorders
Eye disorders
Gastrointestinal disorders
General disorders and administration site conditions
Hepatobiliary disorders
Immune system disorders
Infections and infestations
Injury, poisoning, and procedural complications
Investigations
Metabolism and nutrition disorders
Musculoskeletal and connective tissue disorders
Neoplasms benign, malignant, and unspecified (incl cysts and polyps)
Nervous system disorders
Psychiatric disorders
Renal and urinary disorders
Reproductive system and breast disorders
Respiratory, thoracic, and mediastinal disorders
Skin and subcutaneous tissue disorders
Social circumstances
Surgical and medical procedures
Vascular disorders

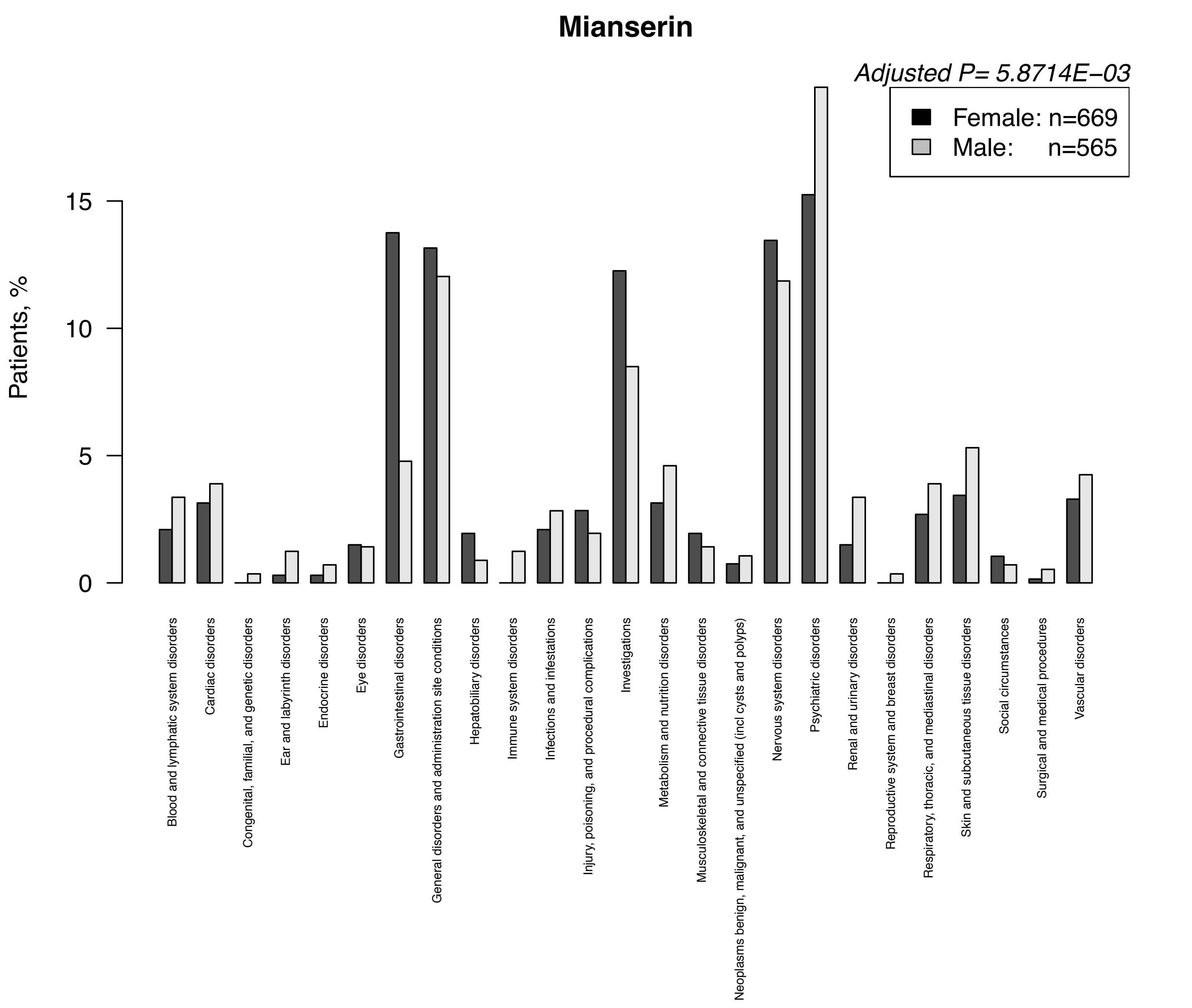
Mianserin
Adjusted P= 5.8714E−03
Female: n=669
Male: n=565
Patients, %
0
5
10
15
Blood and lymphatic system disorders
Cardiac disorders
Congenital, familial, and genetic disorders
Ear and labyrinth disorders
Endocrine disorders
Eye disorders
Gastrointestinal disorders
General disorders and administration site conditions
Hepatobiliary disorders
Immune system disorders
Infections and infestations
Injury, poisoning, and procedural complications
Investigations
Metabolism and nutrition disorders
Musculoskeletal and connective tissue disorders
Neoplasms benign, malignant, and unspecified (incl cysts and polyps)
Nervous system disorders
Psychiatric disorders
Renal and urinary disorders
Reproductive system and breast disorders
Respiratory, thoracic, and mediastinal disorders
Skin and subcutaneous tissue disorders
Social circumstances
Surgical and medical procedures
Vascular disorders

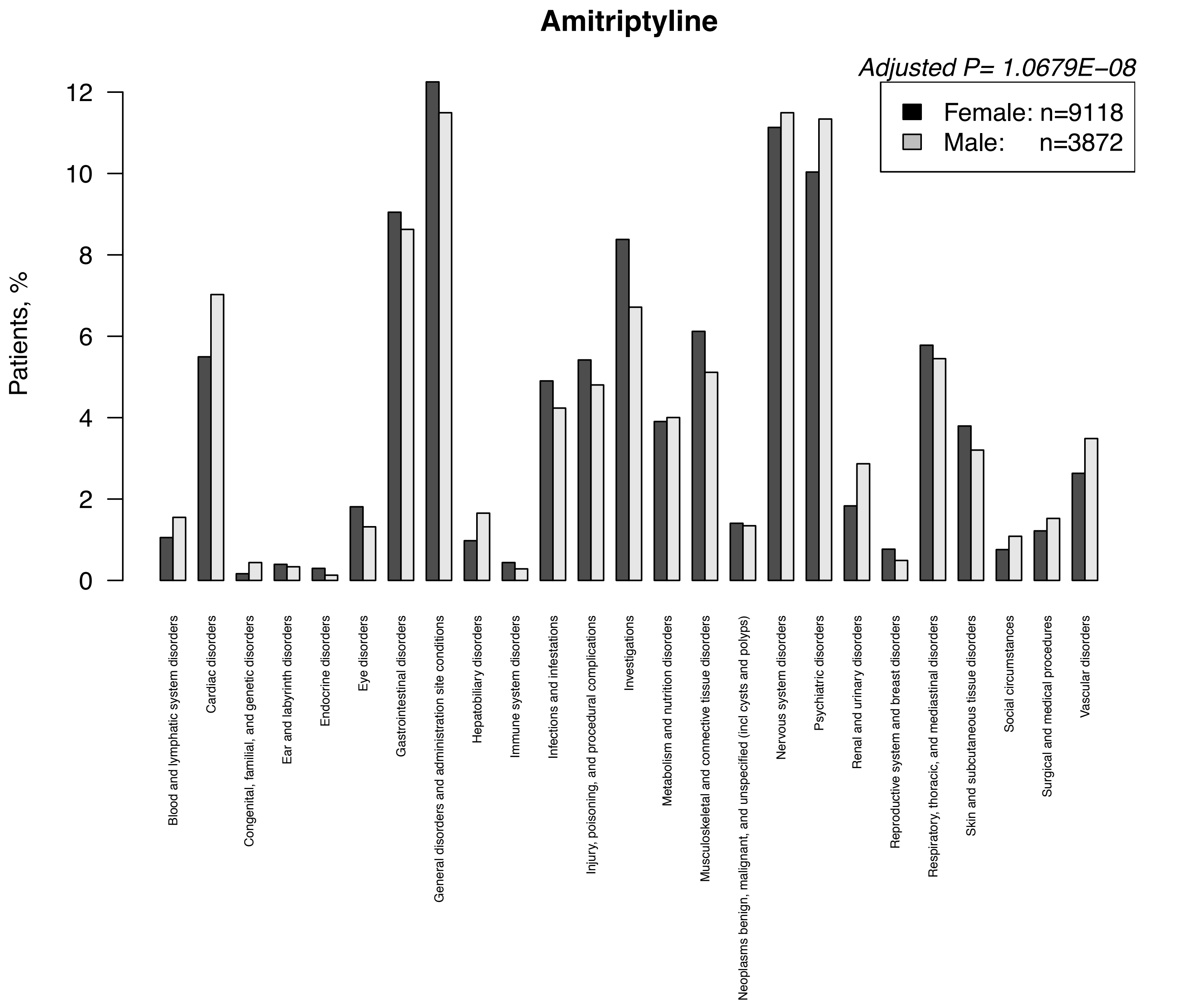
Amitriptyline
Adjusted P= 1.0679E−08
Female: n=9118
Male: n=3872
Patients, %
0
2
4
6
8
10
12
Blood and lymphatic system disorders
Cardiac disorders
Congenital, familial, and genetic disorders
Ear and labyrinth disorders
Endocrine disorders
Eye disorders
Gastrointestinal disorders
General disorders and administration site conditions
Hepatobiliary disorders
Immune system disorders
Infections and infestations
Injury, poisoning, and procedural complications
Investigations
Metabolism and nutrition disorders
Musculoskeletal and connective tissue disorders
Neoplasms benign, malignant, and unspecified (incl cysts and polyps)
Nervous system disorders
Psychiatric disorders
Renal and urinary disorders
Reproductive system and breast disorders
Respiratory, thoracic, and mediastinal disorders
Skin and subcutaneous tissue disorders
Social circumstances
Surgical and medical procedures
Vascular disorders

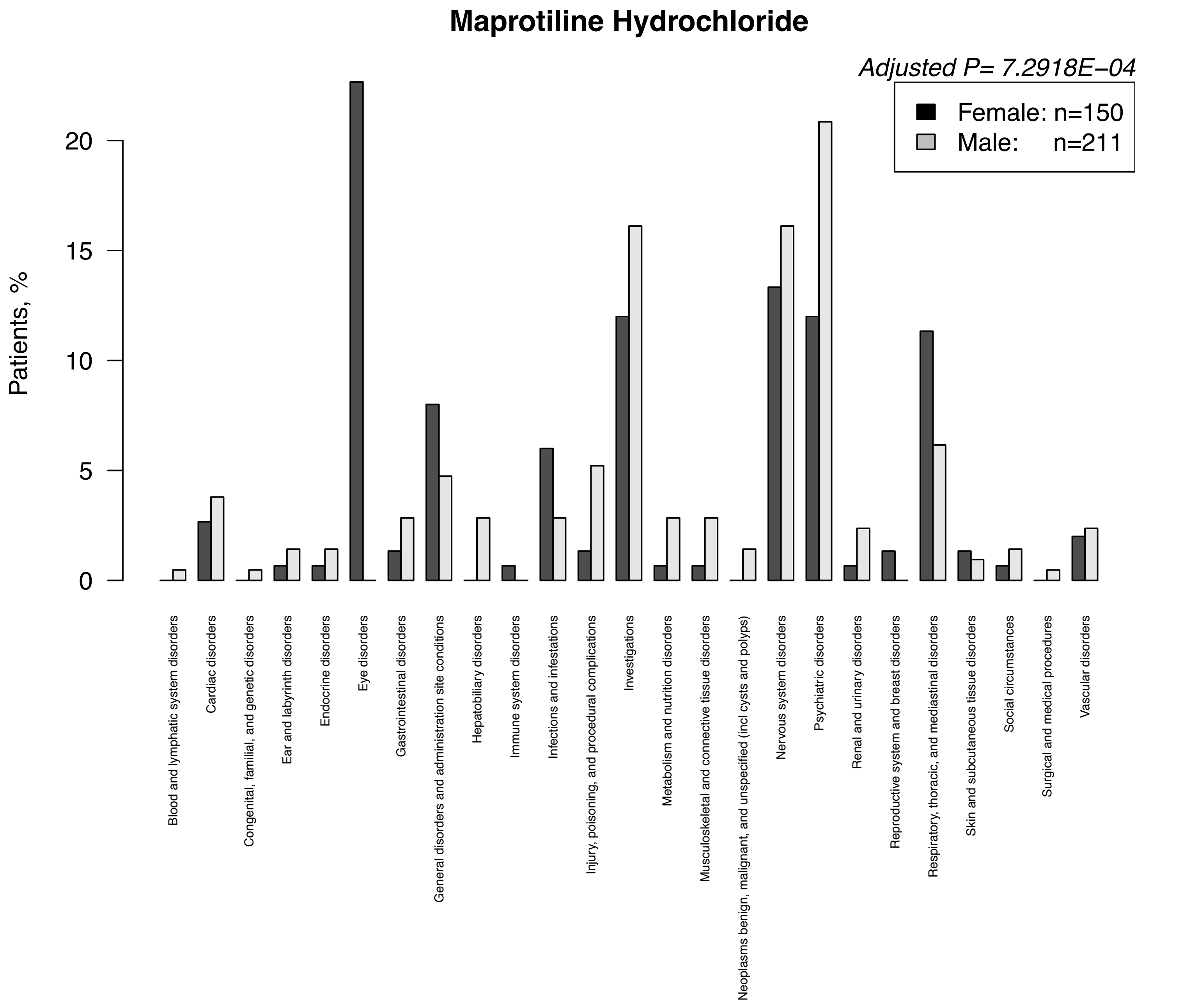

Maprotiline Hydrochloride
Adjusted P= 7.2918E−04
Female: n=150
Male: n=211
Patients, %
0
5
10
15
20
Blood and lymphatic system disorders
Cardiac disorders
Congenital, familial, and genetic disorders
Ear and labyrinth disorders
Endocrine disorders
Eye disorders
Gastrointestinal disorders
General disorders and administration site conditions
Hepatobiliary disorders
Immune system disorders
Infections and infestations
Injury, poisoning, and procedural complications
Investigations
Metabolism and nutrition disorders
Musculoskeletal and connective tissue disorders
Neoplasms benign, malignant, and unspecified (incl cysts and polyps)
Nervous system disorders
Psychiatric disorders
Renal and urinary disorders
Reproductive system and breast disorders
Respiratory, thoracic, and mediastinal disorders
Skin and subcutaneous tissue disorders
Social circumstances
Surgical and medical procedures
Vascular disorders

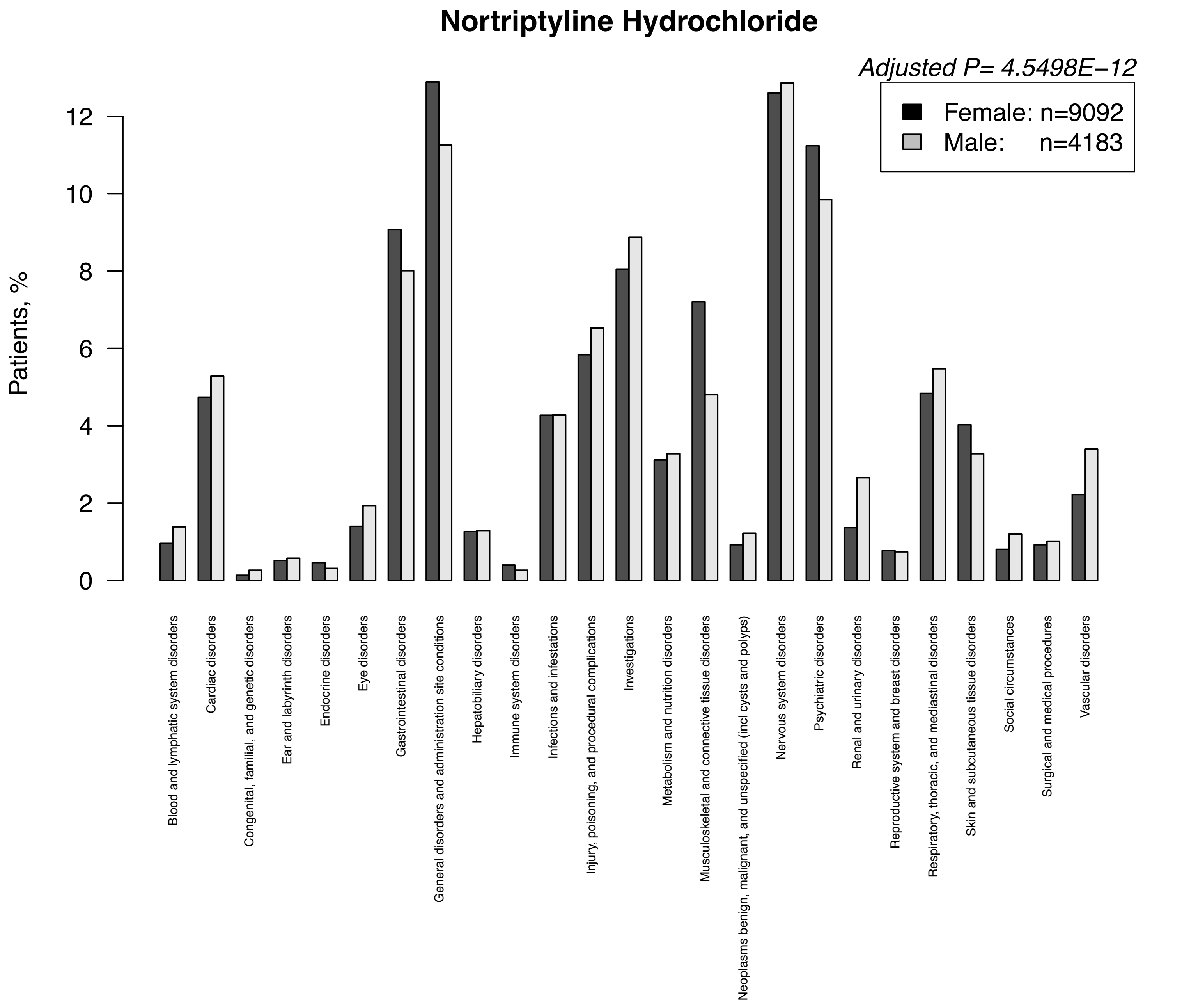
Nortriptyline Hydrochloride
Adjusted P= 4.5498E−12
Female: n=9092
Male: n=4183
Patients, %
0
2
4
6
8
10
12
Blood and lymphatic system disorders
Cardiac disorders
Congenital, familial, and genetic disorders
Ear and labyrinth disorders
Endocrine disorders
Eye disorders
Gastrointestinal disorders
General disorders and administration site conditions
Hepatobiliary disorders
Immune system disorders
Infections and infestations
Injury, poisoning, and procedural complications
Investigations
Metabolism and nutrition disorders
Musculoskeletal and connective tissue disorders
Neoplasms benign, malignant, and unspecified (incl cysts and polyps)
Nervous system disorders
Psychiatric disorders
Renal and urinary disorders
Reproductive system and breast disorders
Respiratory, thoracic, and mediastinal disorders
Skin and subcutaneous tissue disorders
Social circumstances
Surgical and medical procedures
Vascular disorders

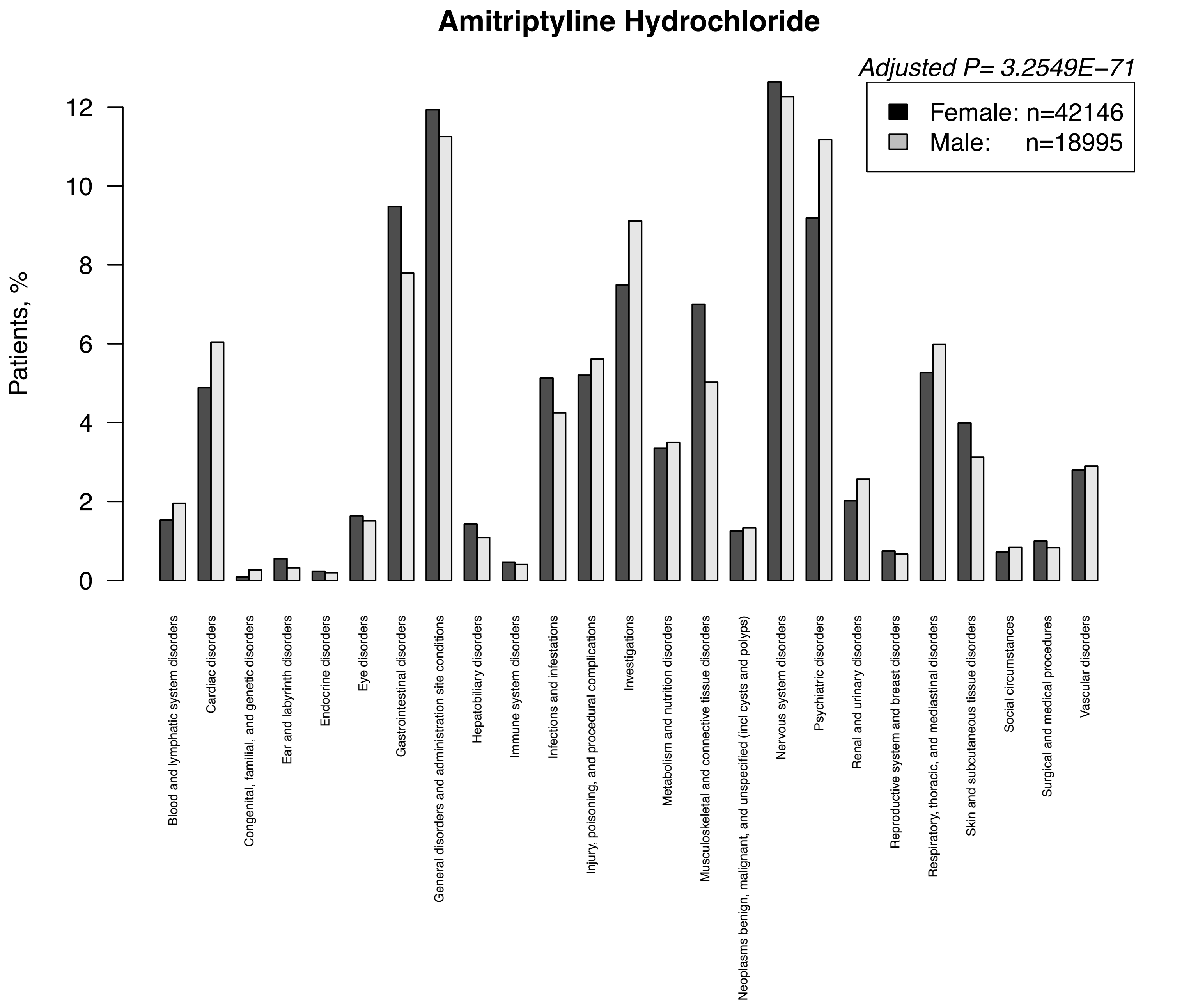
Amitriptyline Hydrochloride
Adjusted P= 3.2549E−71
Female: n=42146
Male: n=18995
Patients, %
0
2
4
6
8
10
12
Blood and lymphatic system disorders
Cardiac disorders
Congenital, familial, and genetic disorders
Ear and labyrinth disorders
Endocrine disorders
Eye disorders
Gastrointestinal disorders
General disorders and administration site conditions
Hepatobiliary disorders
Immune system disorders
Infections and infestations
Injury, poisoning, and procedural complications
Investigations
Metabolism and nutrition disorders
Musculoskeletal and connective tissue disorders
Neoplasms benign, malignant, and unspecified (incl cysts and polyps)
Nervous system disorders
Psychiatric disorders
Renal and urinary disorders
Reproductive system and breast disorders
Respiratory, thoracic, and mediastinal disorders
Skin and subcutaneous tissue disorders
Social circumstances
Surgical and medical procedures
Vascular disorders

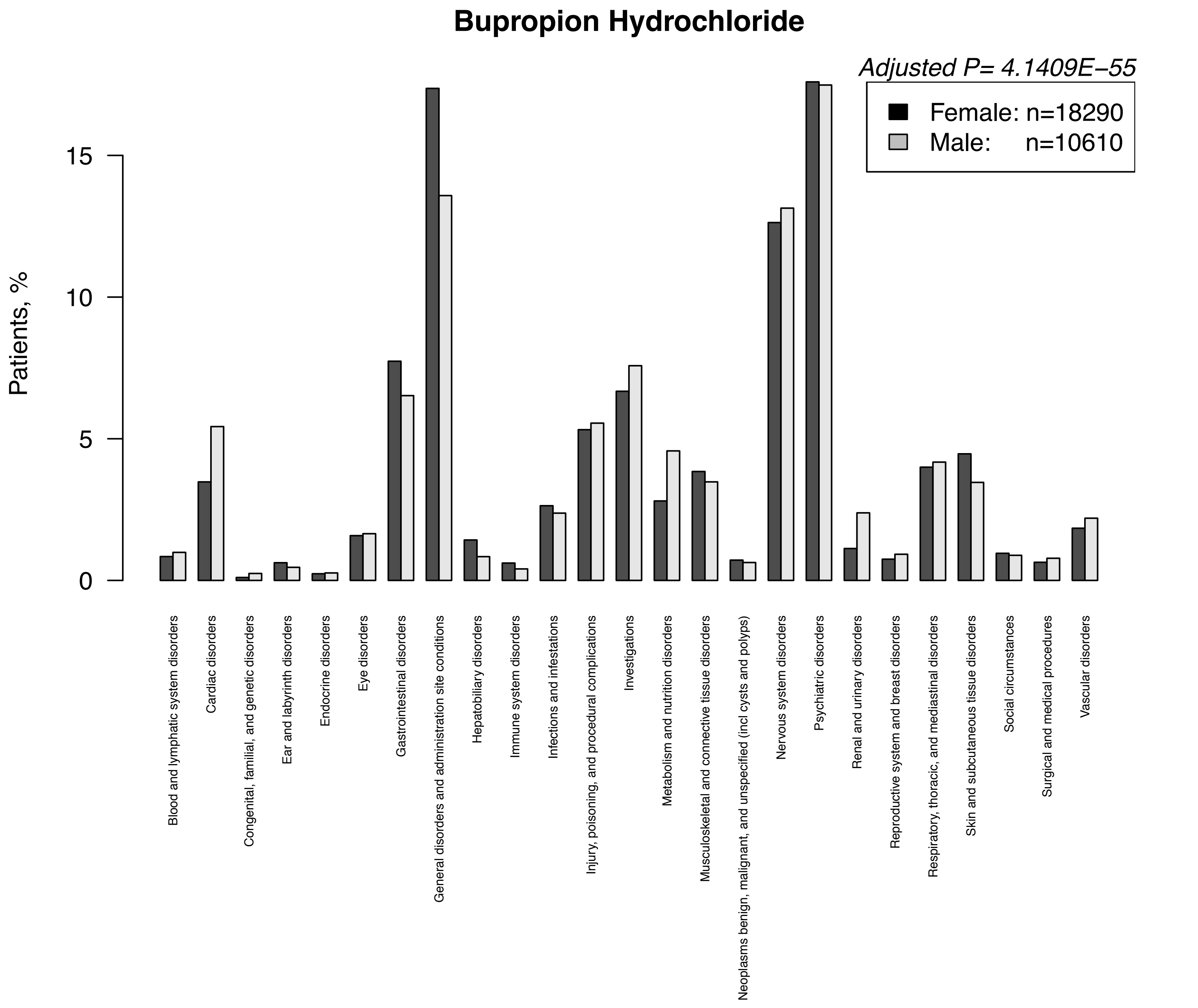
Bupropion Hydrochloride
Adjusted P= 4.1409E−55
Female: n=18290
Male: n=10610
Patients, %
15
10
5
0
Blood and lymphatic system disorders
Cardiac disorders
Congenital, familial, and genetic disorders
Ear and labyrinth disorders
Endocrine disorders
Eye disorders
Gastrointestinal disorders
General disorders and administration site conditions
Hepatobiliary disorders
Immune system disorders
Infections and infestations
Injury, poisoning, and procedural complications
Investigations
Metabolism and nutrition disorders
Musculoskeletal and connective tissue disorders
Neoplasms benign, malignant, and unspecified (incl cysts and polyps)
Nervous system disorders
Psychiatric disorders
Renal and urinary disorders
Reproductive system and breast disorders
Respiratory, thoracic, and mediastinal disorders
Skin and subcutaneous tissue disorders
Social circumstances
Surgical and medical procedures
Vascular disorders

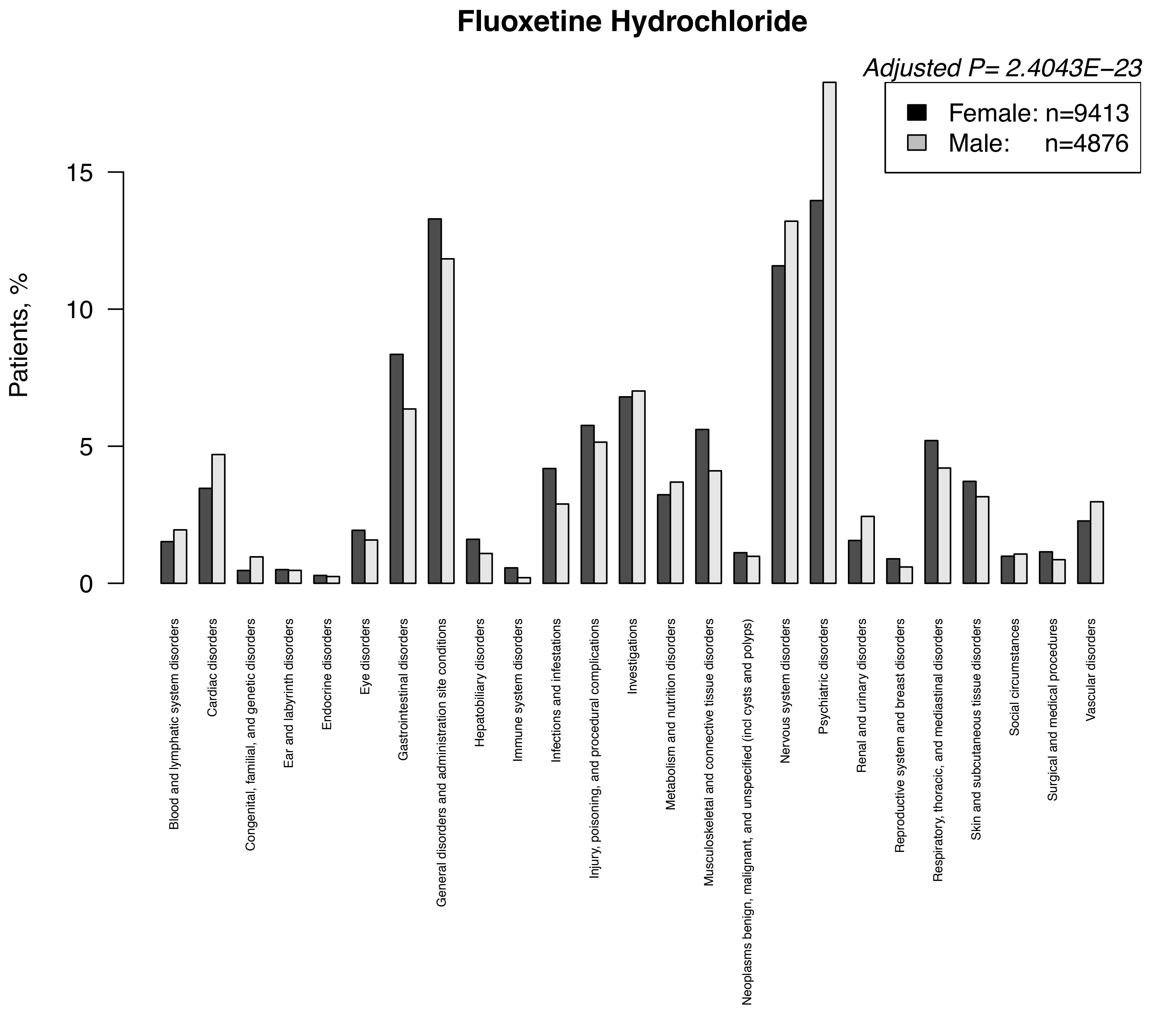
Fluoxetine Hydrochloride
Adjusted P= 2.4043E−23
Female: n=9413
Male: n=4876
Patients, %
0
5
10
15
Blood and lymphatic system disorders
Cardiac disorders
Congenital, familial, and genetic disorders
Ear and labyrinth disorders
Endocrine disorders
Eye disorders
Gastrointestinal disorders
General disorders and administration site conditions
Hepatobiliary disorders
Immune system disorders
Infections and infestations
Injury, poisoning, and procedural complications
Investigations
Metabolism and nutrition disorders
Musculoskeletal and connective tissue disorders
Neoplasms benign, malignant, and unspecified (incl cysts and polyps)
Nervous system disorders
Psychiatric disorders
Renal and urinary disorders
Reproductive system and breast disorders
Respiratory, thoracic, and mediastinal disorders
Skin and subcutaneous tissue disorders
Social circumstances
Surgical and medical procedures
Vascular disorders

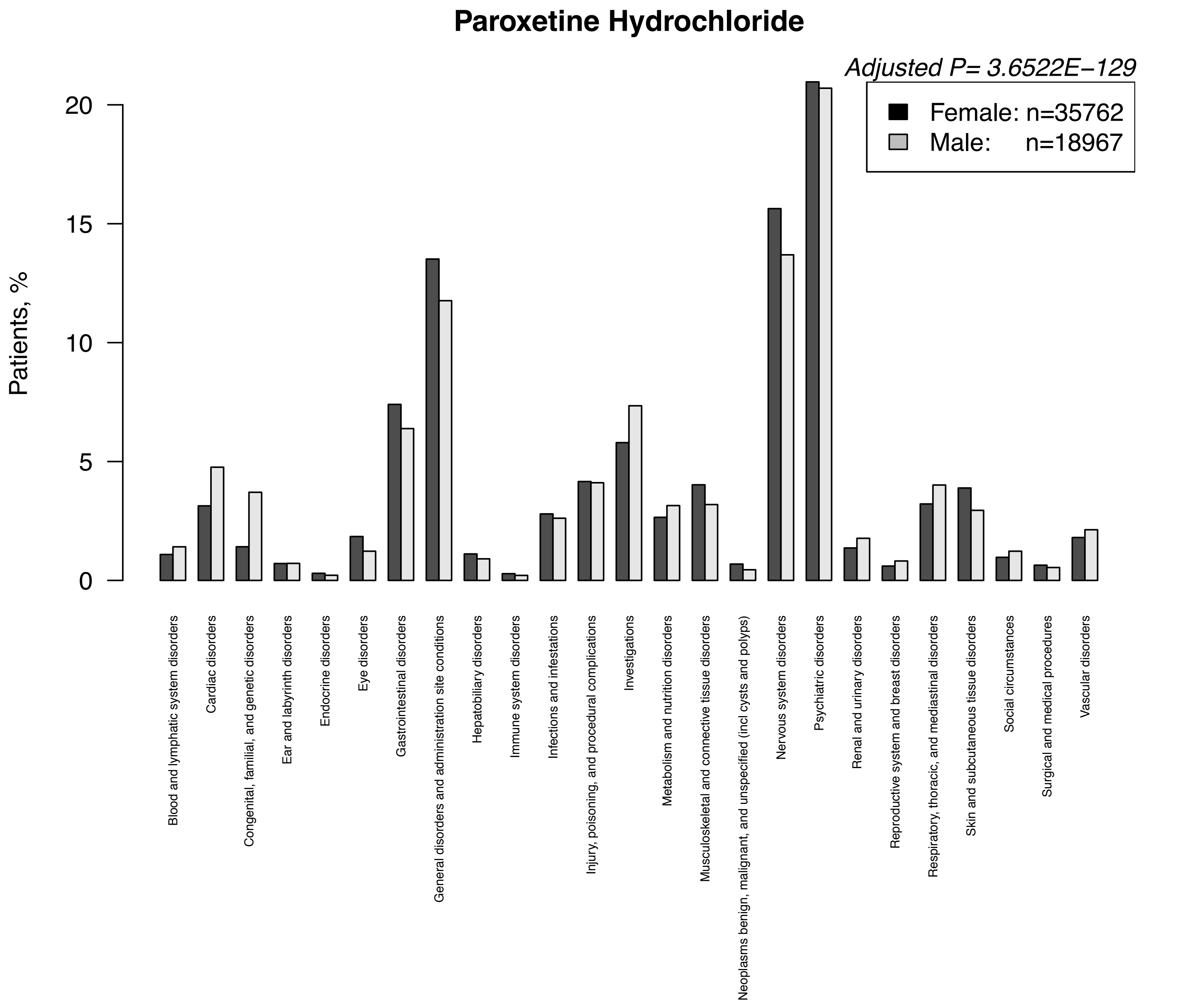
Paroxetine Hydrochloride
Adjusted P= 3.6522E−129
Female: n=35762
Male: n=18967
Patients, %
0
5
10
15
20
Blood and lymphatic system disorders
Cardiac disorders
Congenital, familial, and genetic disorders
Ear and labyrinth disorders
Endocrine disorders
Eye disorders
Gastrointestinal disorders
General disorders and administration site conditions
Hepatobiliary disorders
Immune system disorders
Infections and infestations
Injury, poisoning, and procedural complications
Investigations
Metabolism and nutrition disorders
Musculoskeletal and connective tissue disorders
Neoplasms benign, malignant, and unspecified (incl cysts and polyps)
Nervous system disorders
Psychiatric disorders
Renal and urinary disorders
Reproductive system and breast disorders
Respiratory, thoracic, and mediastinal disorders
Skin and subcutaneous tissue disorders
Social circumstances
Surgical and medical procedures
Vascular disorders

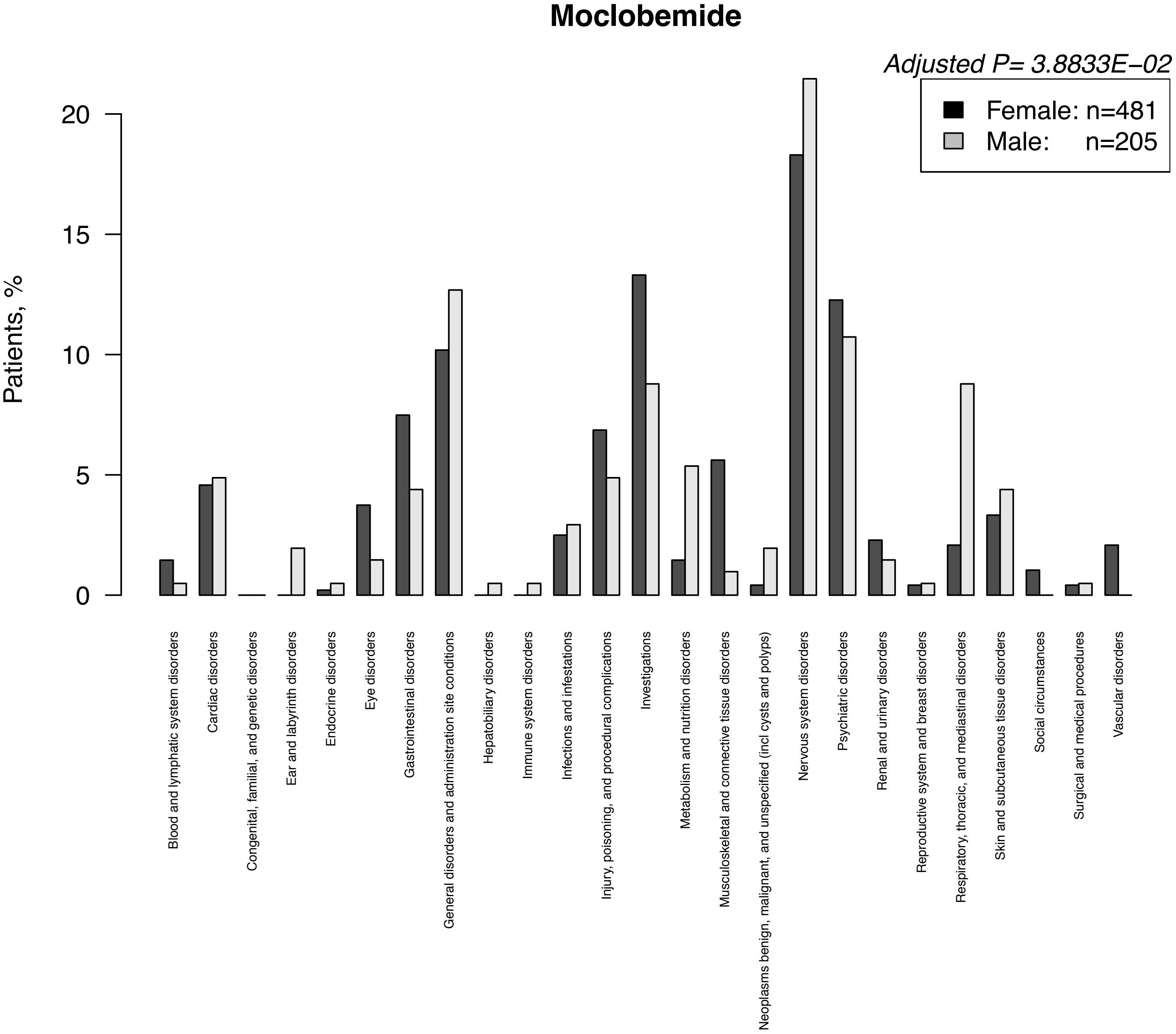
Moclobemide
Adjusted P= 3.8833E−02
Female: n=481
Male: n=205
Patients, %
0
5
10
15
20
Blood and lymphatic system disorders
Cardiac disorders
Congenital, familial, and genetic disorders
Ear and labyrinth disorders
Endocrine disorders
Eye disorders
Gastrointestinal disorders
General disorders and administration site conditions
Hepatobiliary disorders
Immune system disorders
Infections and infestations
Injury, poisoning, and procedural complications
Investigations
Metabolism and nutrition disorders
Musculoskeletal and connective tissue disorders
Neoplasms benign, malignant, and unspecified (incl cysts and polyps)
Nervous system disorders
Psychiatric disorders
Renal and urinary disorders
Reproductive system and breast disorders
Respiratory, thoracic, and mediastinal disorders
Skin and subcutaneous tissue disorders
Social circumstances
Surgical and medical procedures
Vascular disorders

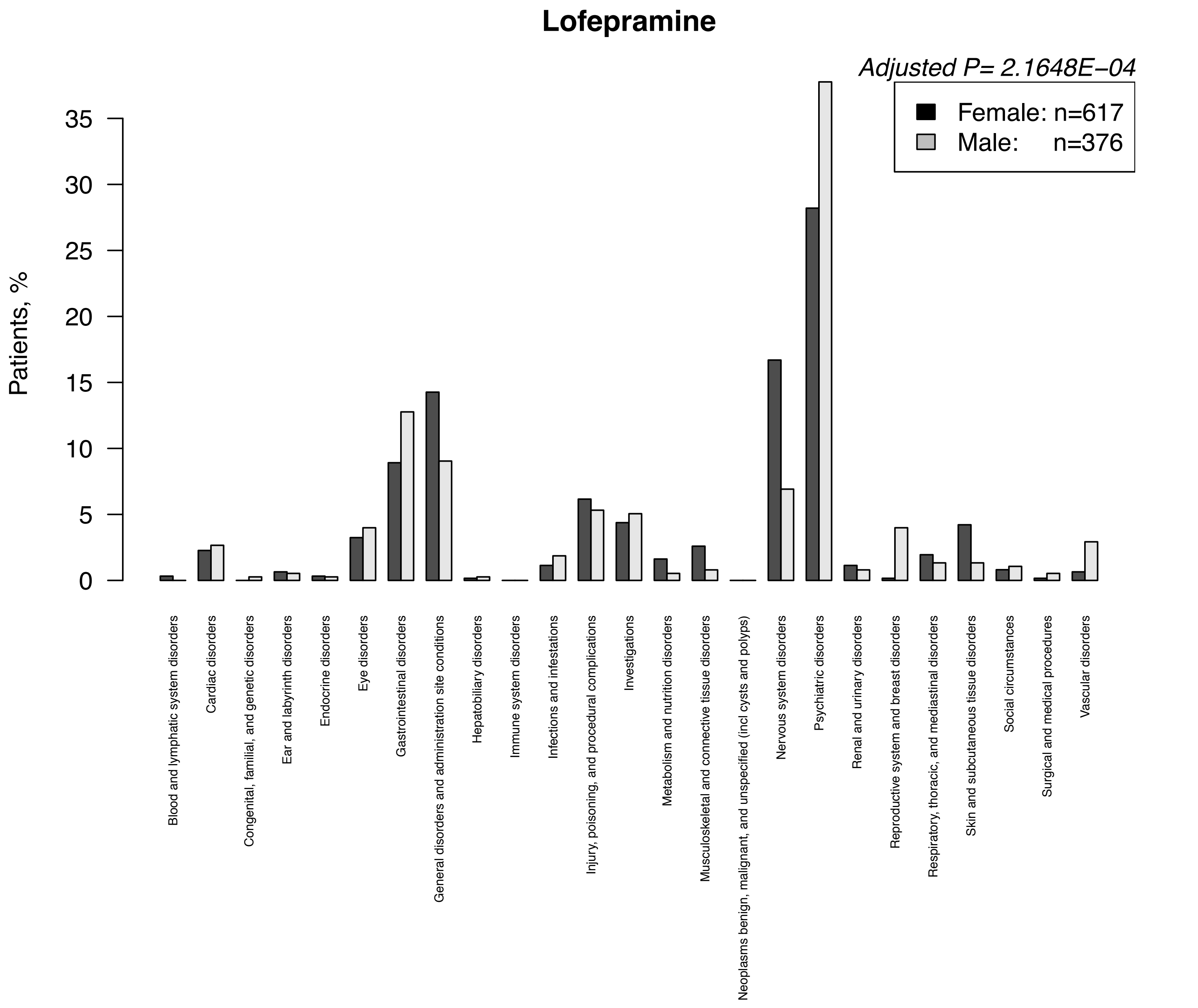
Lofepramine
Adjusted P= 2.1648E−04
Female: n=617
Male: n=376
Patients, %
0
5
10
15
20
25
30
35
Blood and lymphatic system disorders
Cardiac disorders
Congenital, familial, and genetic disorders
Ear and labyrinth disorders
Endocrine disorders
Eye disorders
Gastrointestinal disorders
General disorders and administration site conditions
Hepatobiliary disorders
Immune system disorders
Infections and infestations
Injury, poisoning, and procedural complications
Investigations
Metabolism and nutrition disorders
Musculoskeletal and connective tissue disorders
Neoplasms benign, malignant, and unspecified (incl cysts and polyps)
Nervous system disorders
Psychiatric disorders
Renal and urinary disorders
Reproductive system and breast disorders
Respiratory, thoracic, and mediastinal disorders
Skin and subcutaneous tissue disorders
Social circumstances
Surgical and medical procedures
Vascular disorders

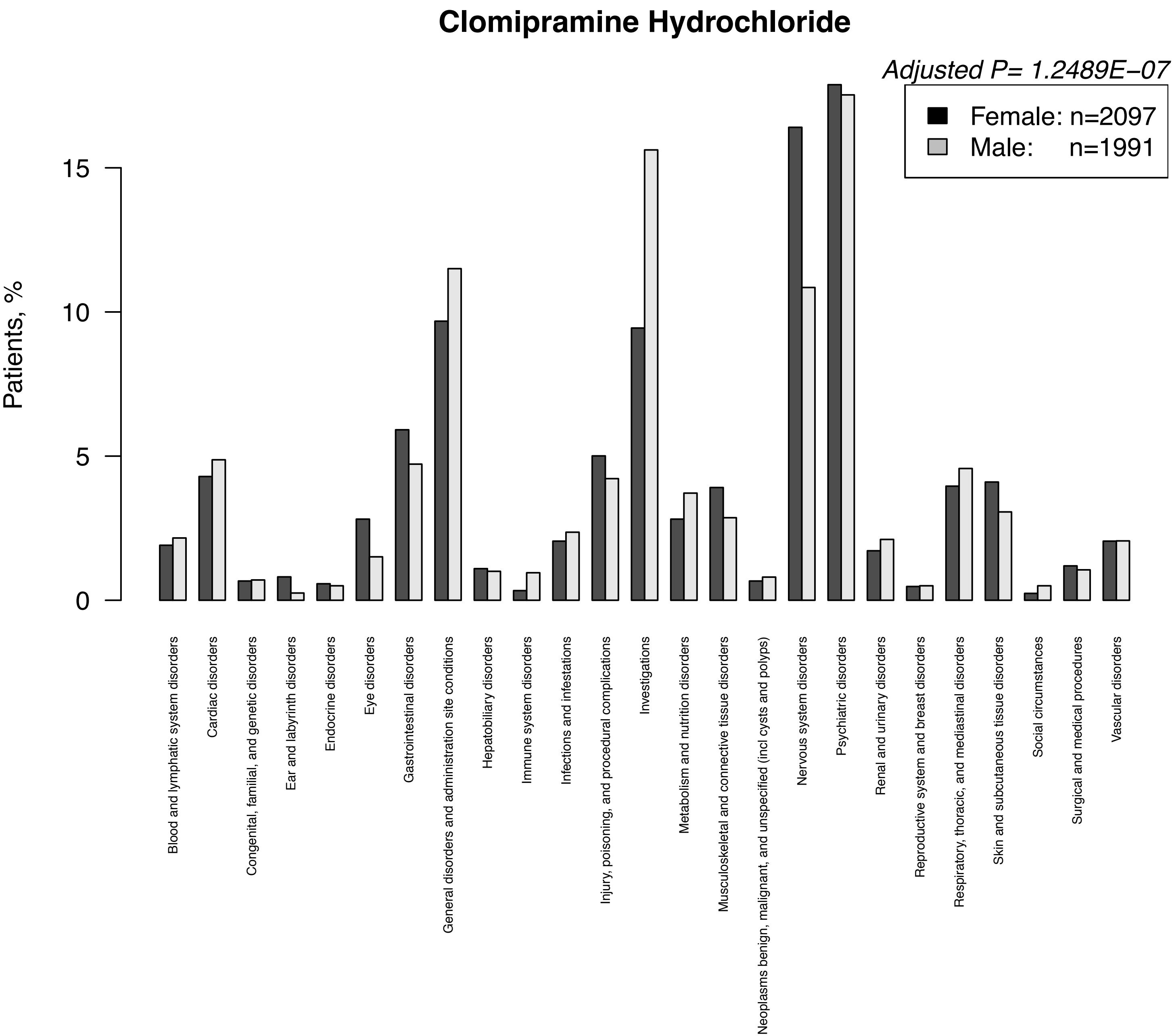

Clomipramine Hydrochloride
Adjusted P= 1.2489E−07
Female: n=2097
Male: n=1991
Patients, %
0
5
10
15
Blood and lymphatic system disorders
Cardiac disorders
Congenital, familial, and genetic disorders
Ear and labyrinth disorders
Endocrine disorders
Eye disorders
Gastrointestinal disorders
General disorders and administration site conditions
Hepatobiliary disorders
Immune system disorders
Infections and infestations
Injury, poisoning, and procedural complications
Investigations
Metabolism and nutrition disorders
Musculoskeletal and connective tissue disorders
Neoplasms benign, malignant, and unspecified (incl cysts and polyps)
Nervous system disorders
Psychiatric disorders
Renal and urinary disorders
Reproductive system and breast disorders
Respiratory, thoracic, and mediastinal disorders
Skin and subcutaneous tissue disorders
Social circumstances
Surgical and medical procedures
Vascular disorders

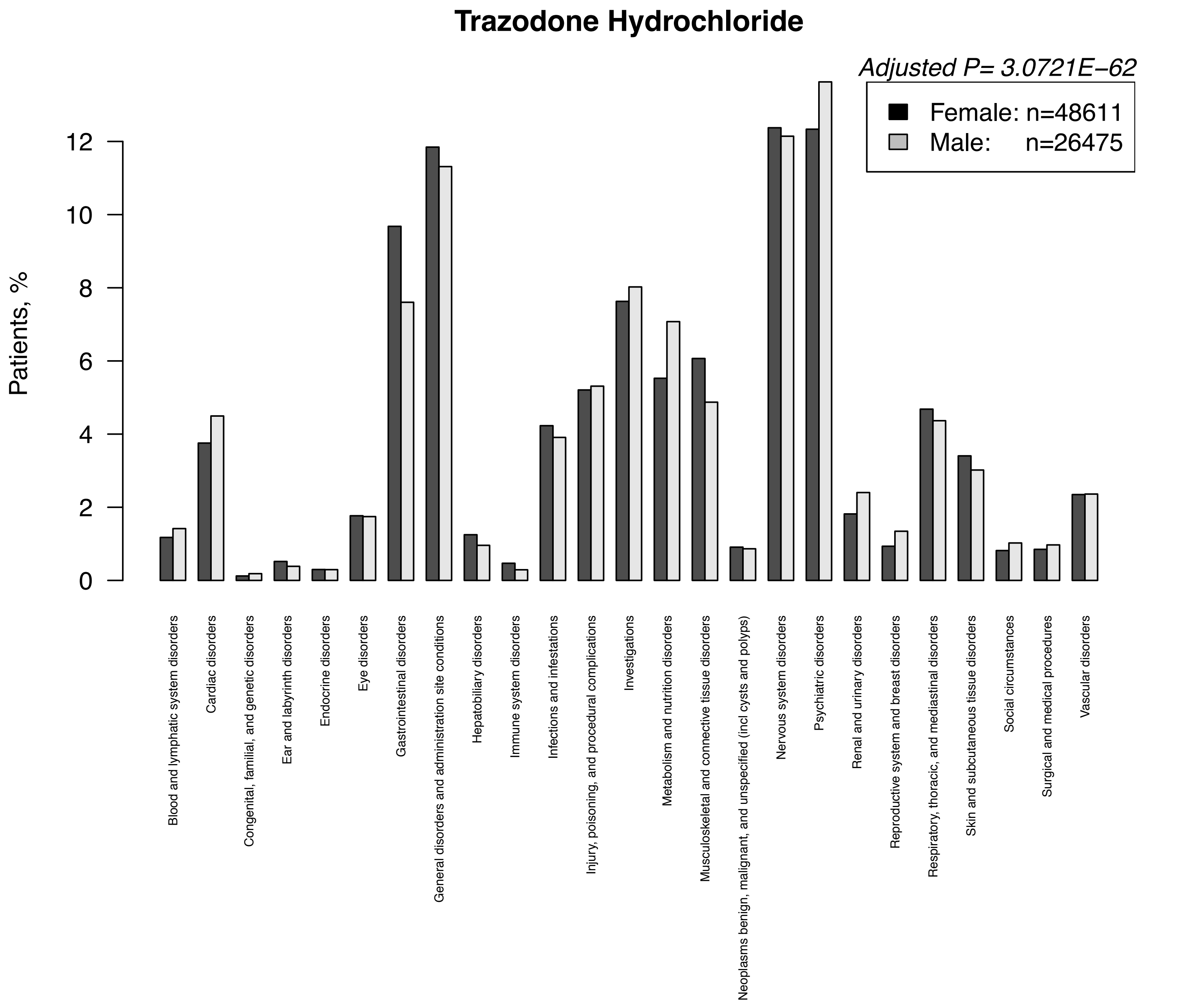
Trazodone Hydrochloride
Adjusted P= 3.0721E−62
Female: n=48611
Male: n=26475
Patients, %
0
2
4
6
8
10
12
Blood and lymphatic system disorders
Cardiac disorders
Congenital, familial, and genetic disorders
Ear and labyrinth disorders
Endocrine disorders
Eye disorders
Gastrointestinal disorders
General disorders and administration site conditions
Hepatobiliary disorders
Immune system disorders
Infections and infestations
Injury, poisoning, and procedural complications
Investigations
Metabolism and nutrition disorders
Musculoskeletal and connective tissue disorders
Neoplasms benign, malignant, and unspecified (incl cysts and polyps)
Nervous system disorders
Psychiatric disorders
Renal and urinary disorders
Reproductive system and breast disorders
Respiratory, thoracic, and mediastinal disorders
Skin and subcutaneous tissue disorders
Social circumstances
Surgical and medical procedures
Vascular disorders

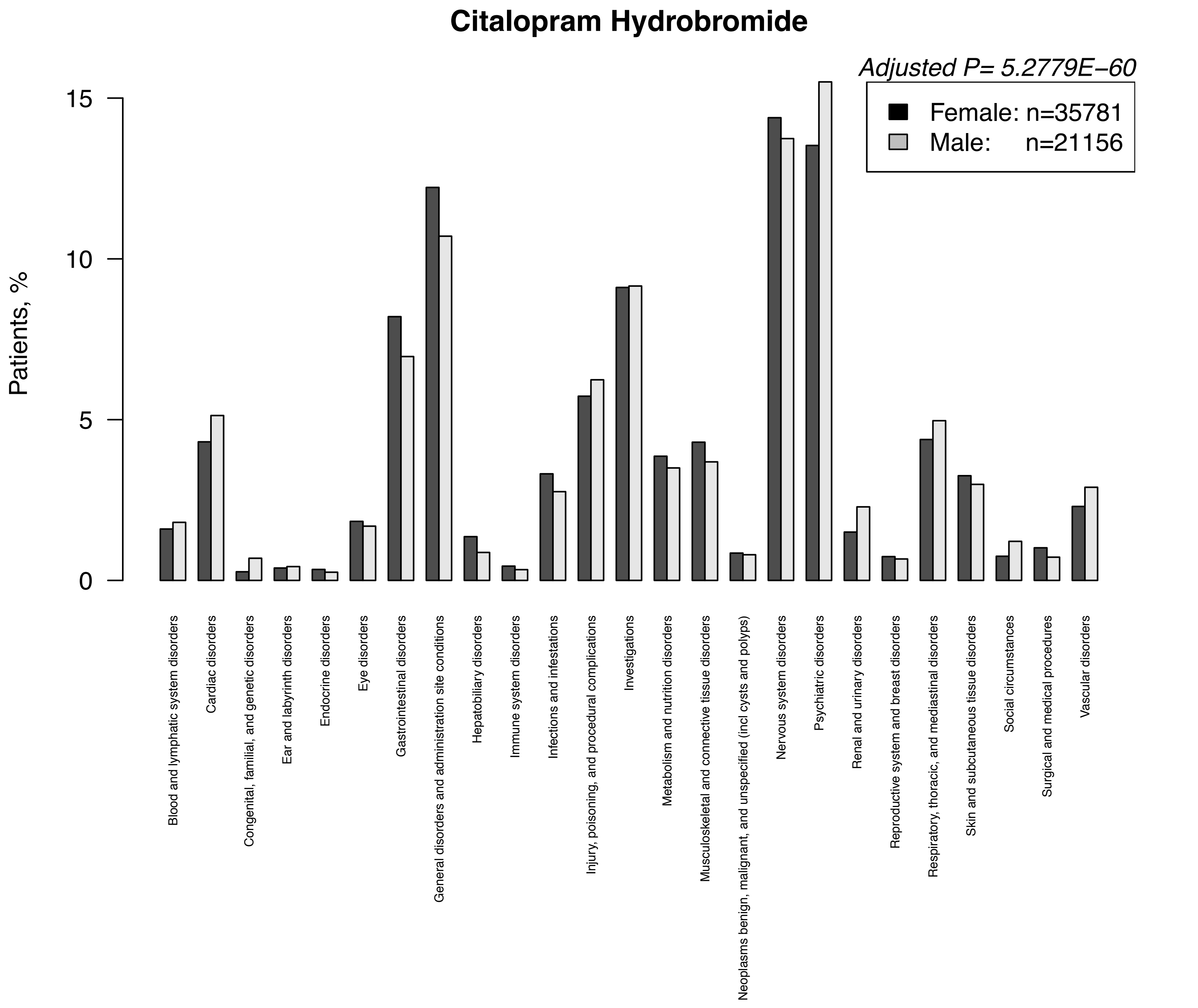

Citalopram Hydrobromide
Adjusted P= 5.2779E−60
Female: n=35781
Male: n=21156
Patients, %
0
5
10
15
Blood and lymphatic system disorders
Cardiac disorders
Congenital, familial, and genetic disorders
Ear and labyrinth disorders
Endocrine disorders
Eye disorders
Gastrointestinal disorders
General disorders and administration site conditions
Hepatobiliary disorders
Immune system disorders
Infections and infestations
Injury, poisoning, and procedural complications
Investigations
Metabolism and nutrition disorders
Musculoskeletal and connective tissue disorders
Neoplasms benign, malignant, and unspecified (incl cysts and polyps)
Nervous system disorders
Psychiatric disorders
Renal and urinary disorders
Reproductive system and breast disorders
Respiratory, thoracic, and mediastinal disorders
Skin and subcutaneous tissue disorders
Social circumstances
Surgical and medical procedures
Vascular disorders

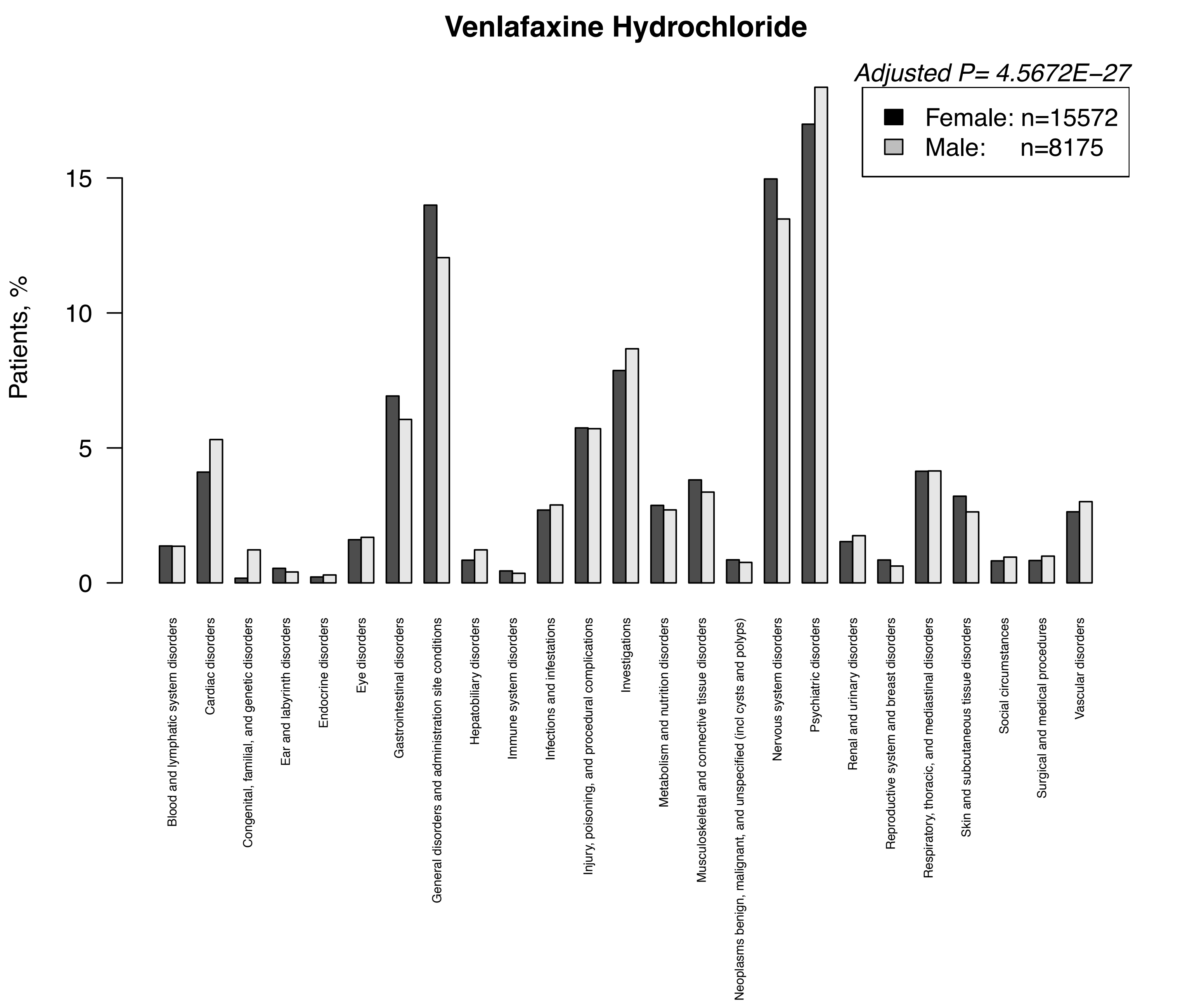
Venlafaxine Hydrochloride
Adjusted P= 4.5672E−27
Female: n=15572
Male: n=8175
Patients, %
0
5
10
15
Blood and lymphatic system disorders
Cardiac disorders
Congenital, familial, and genetic disorders
Ear and labyrinth disorders
Endocrine disorders
Eye disorders
Gastrointestinal disorders
General disorders and administration site conditions
Hepatobiliary disorders
Immune system disorders
Infections and infestations
Injury, poisoning, and procedural complications
Investigations
Metabolism and nutrition disorders
Musculoskeletal and connective tissue disorders
Neoplasms benign, malignant, and unspecified (incl cysts and polyps)
Nervous system disorders
Psychiatric disorders
Renal and urinary disorders
Reproductive system and breast disorders
Respiratory, thoracic, and mediastinal disorders
Skin and subcutaneous tissue disorders
Social circumstances
Surgical and medical procedures
Vascular disorders

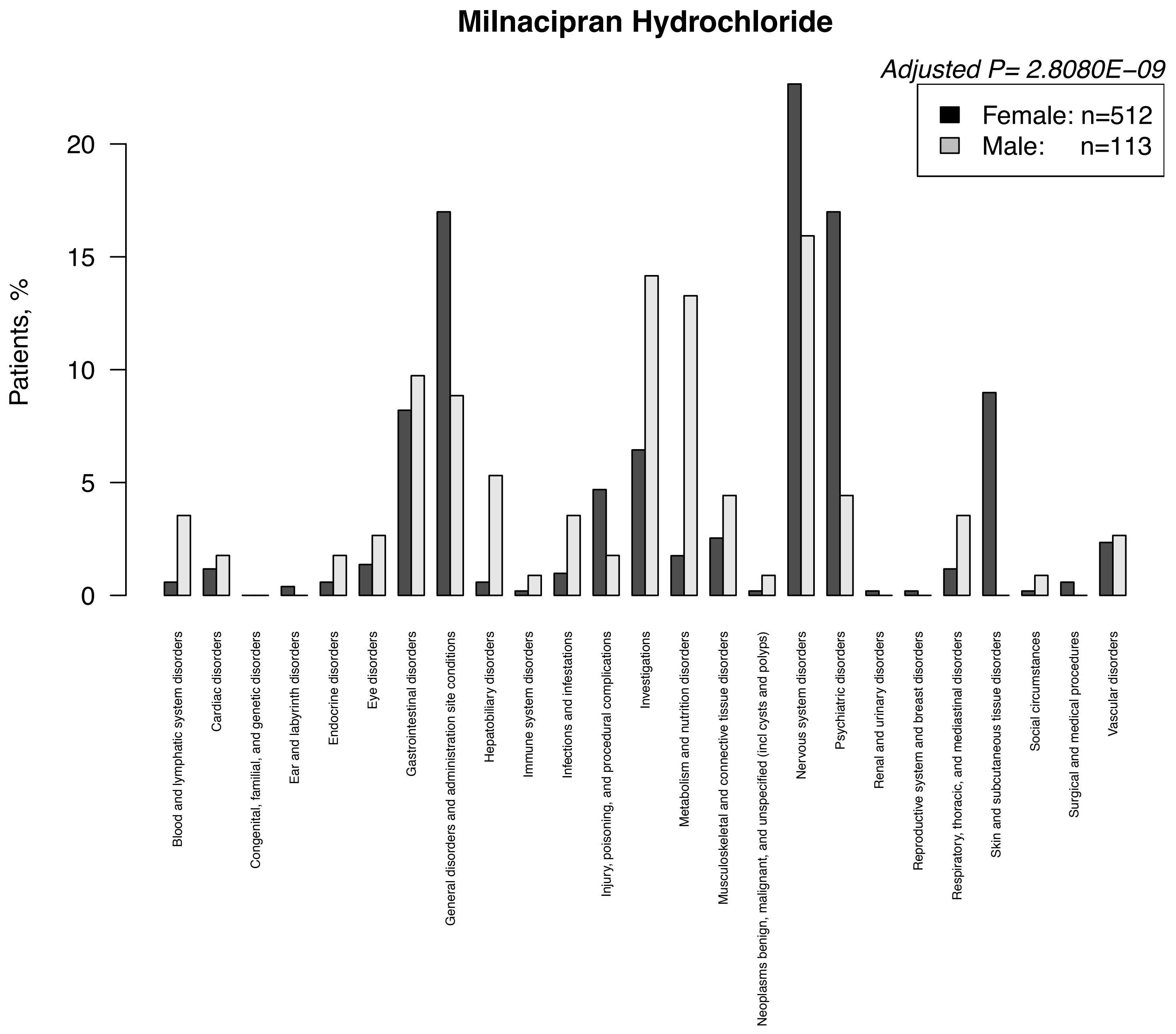

Milnacipran Hydrochloride
Adjusted P= 2.8080E−09
Female: n=512
Male: n=113
Patients, %
0
5
10
15
20
Blood and lymphatic system disorders
Cardiac disorders
Congenital, familial, and genetic disorders
Ear and labyrinth disorders
Endocrine disorders
Eye disorders
Gastrointestinal disorders
General disorders and administration site conditions
Hepatobiliary disorders
Immune system disorders
Infections and infestations
Injury, poisoning, and procedural complications
Investigations
Metabolism and nutrition disorders
Musculoskeletal and connective tissue disorders
Neoplasms benign, malignant, and unspecified (incl cysts and polyps)
Nervous system disorders
Psychiatric disorders
Renal and urinary disorders
Reproductive system and breast disorders
Respiratory, thoracic, and mediastinal disorders
Skin and subcutaneous tissue disorders
Social circumstances
Surgical and medical procedures
Vascular disorders

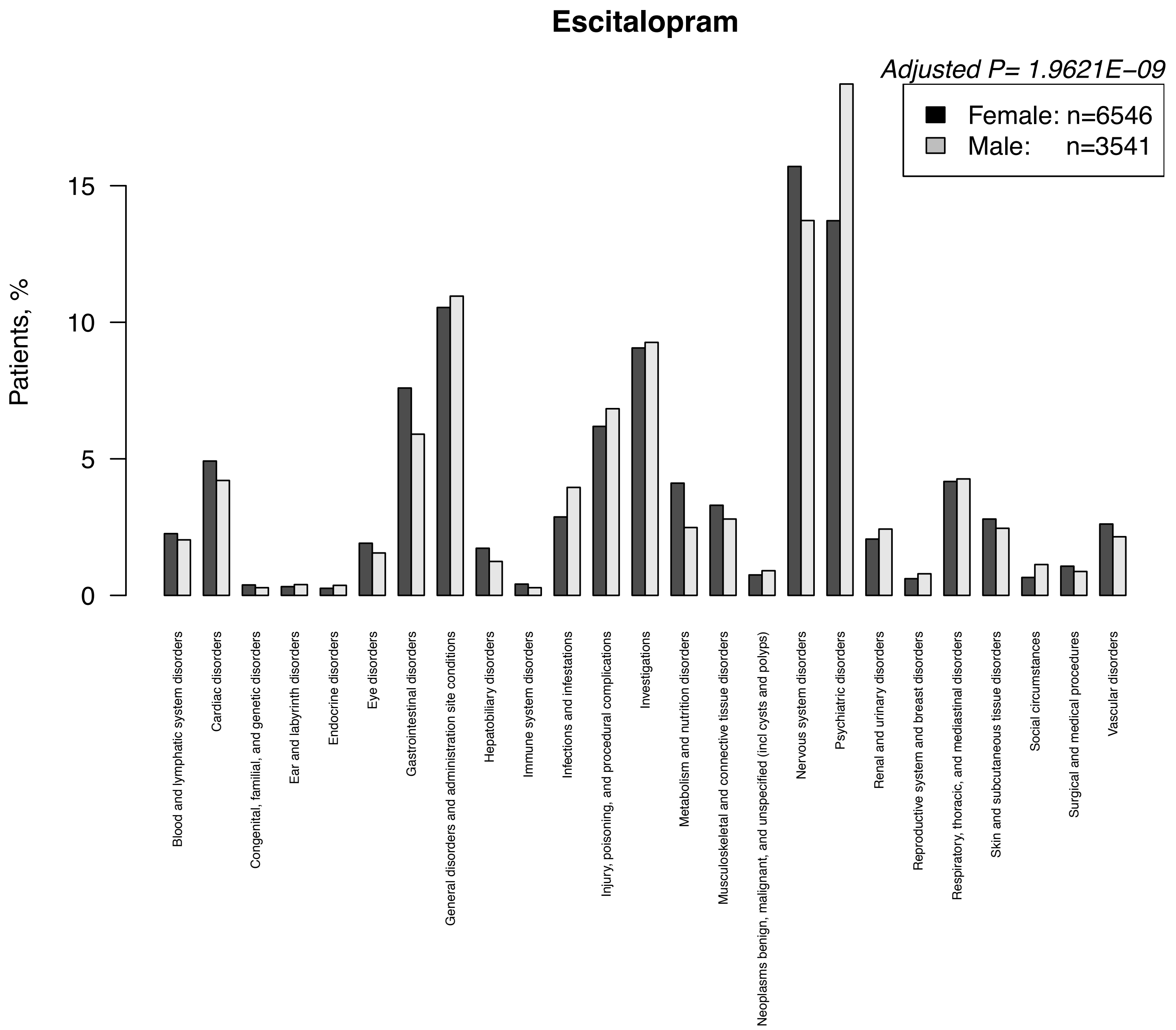
Escitalopram
Adjusted P= 1.9621E−09
Female: n=6546
Male: n=3541
Patients, %
15
10
5
0
Blood and lymphatic system disorders
Cardiac disorders
Congenital, familial, and genetic disorders
Ear and labyrinth disorders
Endocrine disorders
Eye disorders
Gastrointestinal disorders
General disorders and administration site conditions
Hepatobiliary disorders
Immune system disorders
Infections and infestations
Injury, poisoning, and procedural complications
Investigations
Metabolism and nutrition disorders
Musculoskeletal and connective tissue disorders
Neoplasms benign, malignant, and unspecified (incl cysts and polyps)
Nervous system disorders
Psychiatric disorders
Renal and urinary disorders
Reproductive system and breast disorders
Respiratory, thoracic, and mediastinal disorders
Skin and subcutaneous tissue disorders
Social circumstances
Surgical and medical procedures
Vascular disorders

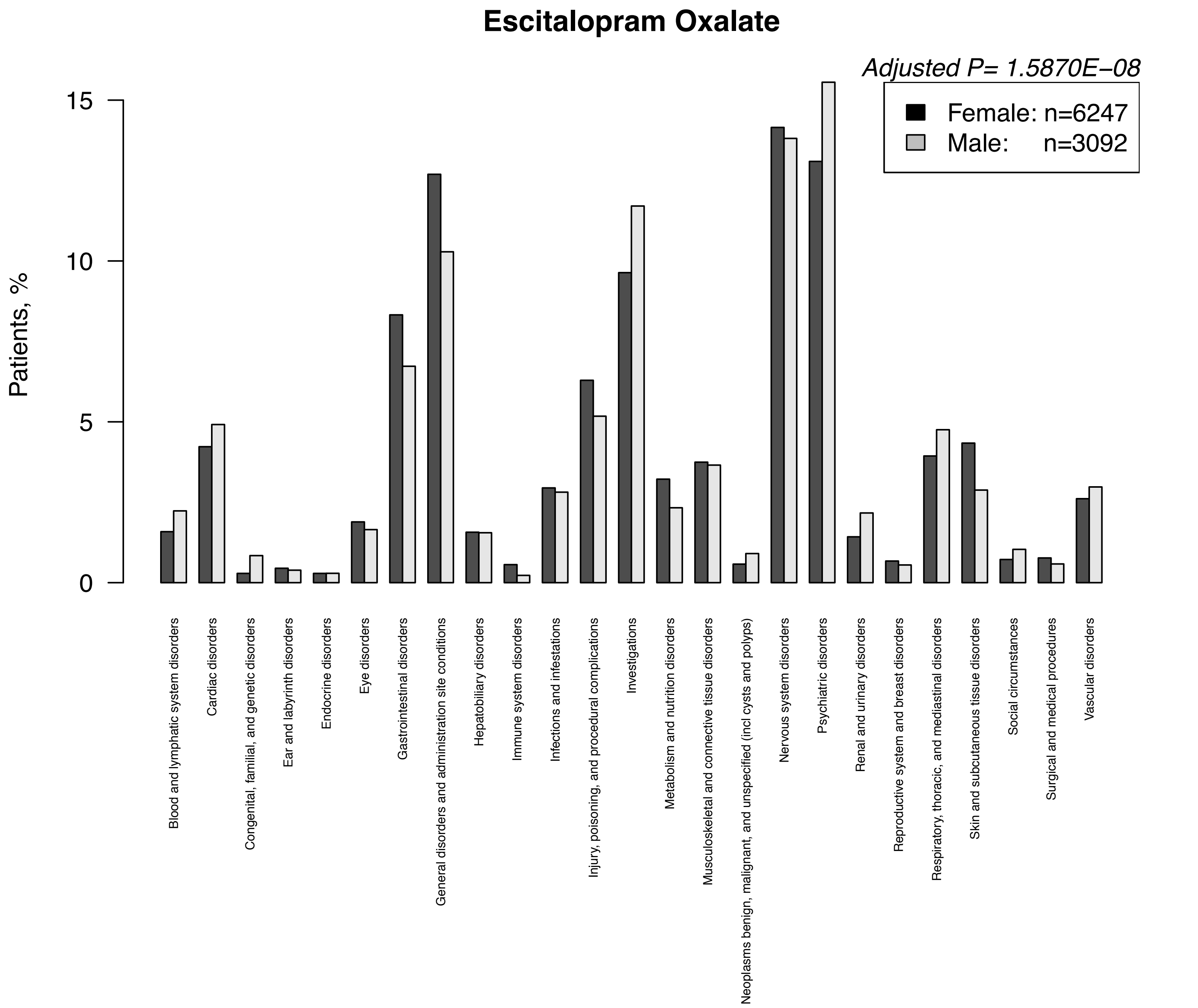
Escitalopram Oxalate
Adjusted P= 1.5870E−08
Female: n=6247
Male: n=3092
Patients, %
0
5
10
15
Blood and lymphatic system disorders
Cardiac disorders
Congenital, familial, and genetic disorders
Ear and labyrinth disorders
Endocrine disorders
Eye disorders
Gastrointestinal disorders
General disorders and administration site conditions
Hepatobiliary disorders
Immune system disorders
Infections and infestations
Injury, poisoning, and procedural complications
Investigations
Metabolism and nutrition disorders
Musculoskeletal and connective tissue disorders
Neoplasms benign, malignant, and unspecified (incl cysts and polyps)
Nervous system disorders
Psychiatric disorders
Renal and urinary disorders
Reproductive system and breast disorders
Respiratory, thoracic, and mediastinal disorders
Skin and subcutaneous tissue disorders
Social circumstances
Surgical and medical procedures
Vascular disorders

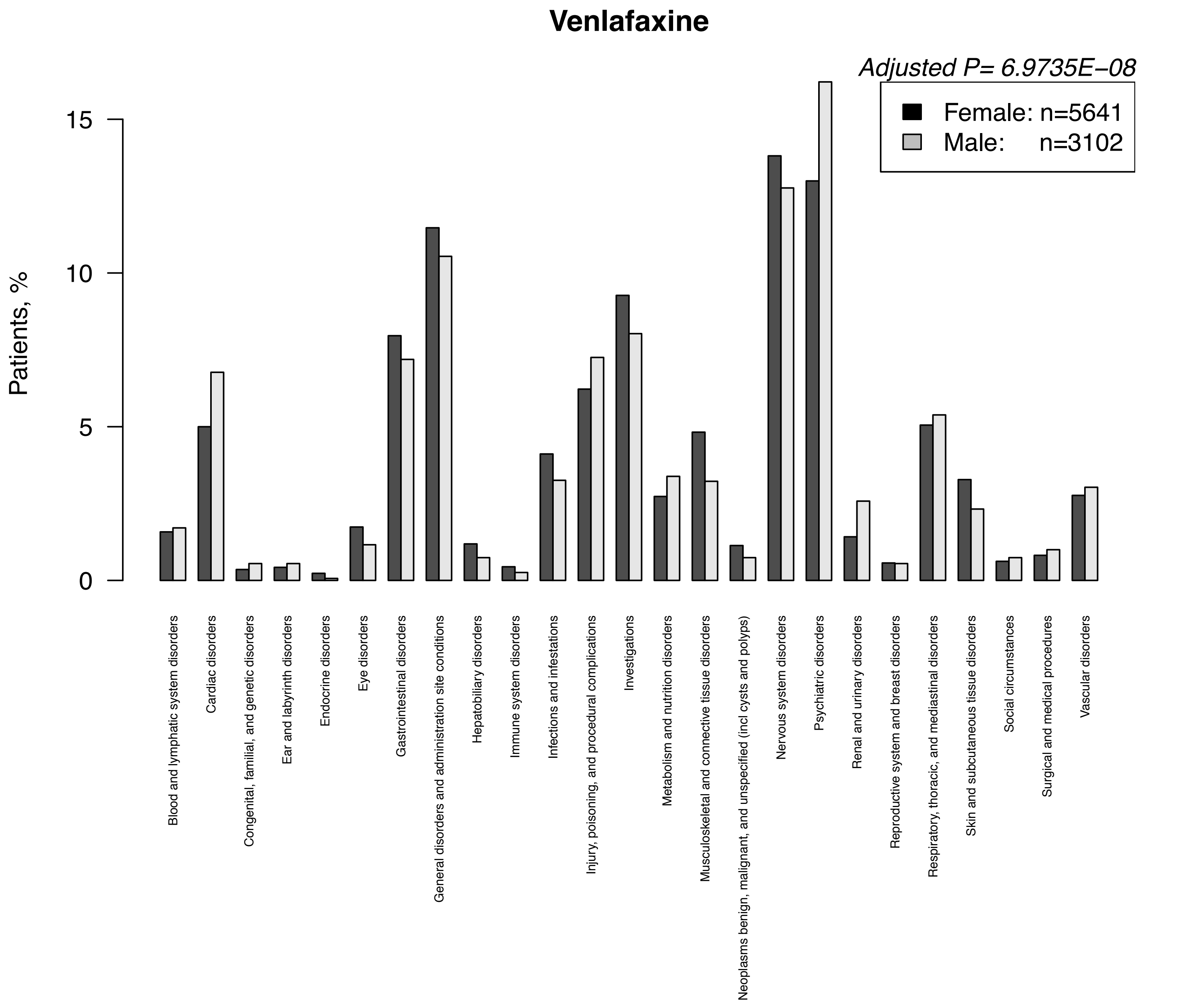

Venlafaxine
Adjusted P= 6.9735E−08
Female: n=5641
Male: n=3102
Patients, %
0
5
10
15
Blood and lymphatic system disorders
Cardiac disorders
Congenital, familial, and genetic disorders
Ear and labyrinth disorders
Endocrine disorders
Eye disorders
Gastrointestinal disorders
General disorders and administration site conditions
Hepatobiliary disorders
Immune system disorders
Infections and infestations
Injury, poisoning, and procedural complications
Investigations
Metabolism and nutrition disorders
Musculoskeletal and connective tissue disorders
Neoplasms benign, malignant, and unspecified (incl cysts and polyps)
Nervous system disorders
Psychiatric disorders
Renal and urinary disorders
Reproductive system and breast disorders
Respiratory, thoracic, and mediastinal disorders
Skin and subcutaneous tissue disorders
Social circumstances
Surgical and medical procedures
Vascular disorders

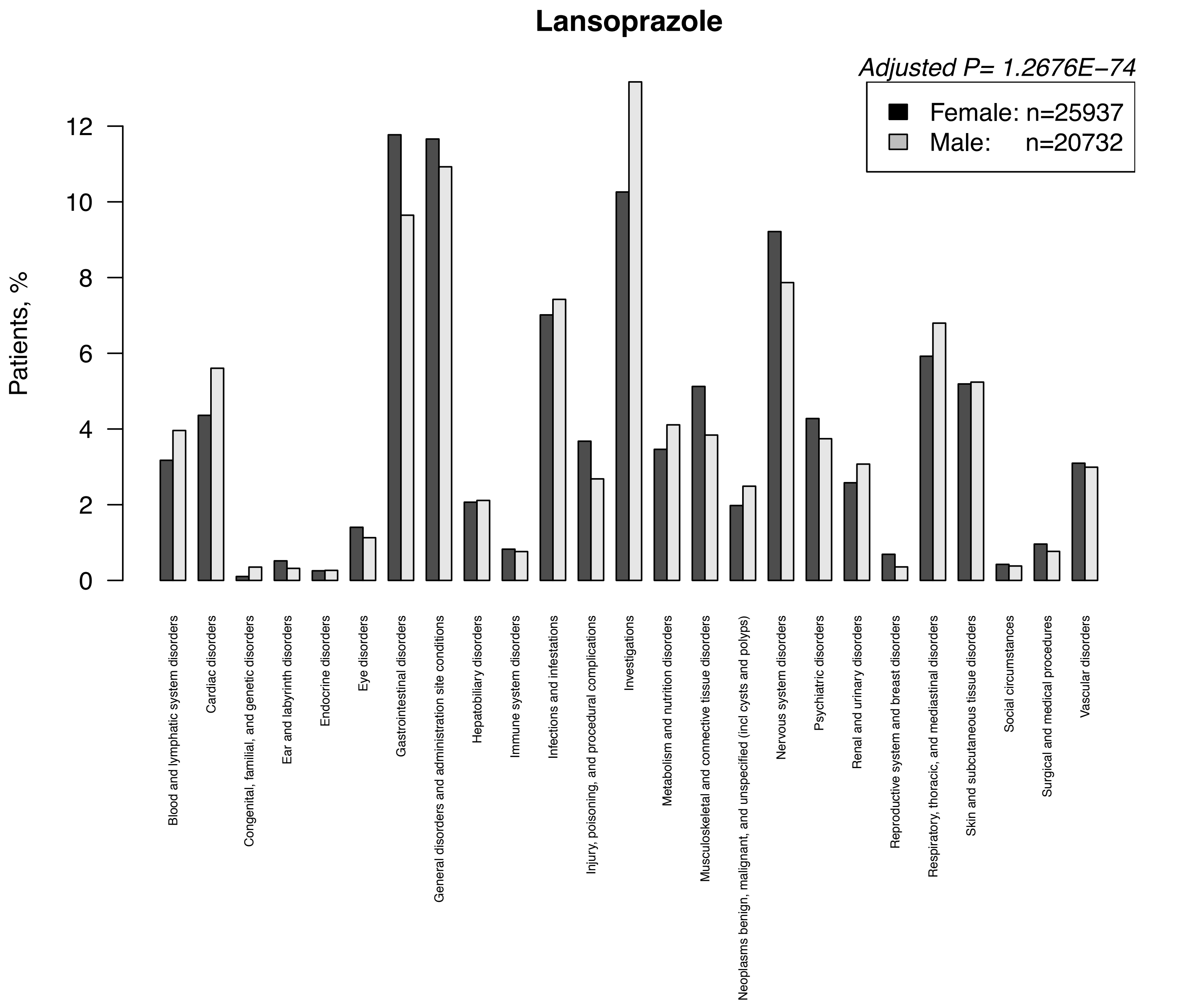
Lansoprazole
Adjusted P= 1.2676E−74
Female: n=25937
Male: n=20732
Patients, %
0
2
4
6
8
10
12
Blood and lymphatic system disorders
Cardiac disorders
Congenital, familial, and genetic disorders
Ear and labyrinth disorders
Endocrine disorders
Eye disorders
Gastrointestinal disorders
General disorders and administration site conditions
Hepatobiliary disorders
Immune system disorders
Infections and infestations
Injury, poisoning, and procedural complications
Investigations
Metabolism and nutrition disorders
Musculoskeletal and connective tissue disorders
Neoplasms benign, malignant, and unspecified (incl cysts and polyps)
Nervous system disorders
Psychiatric disorders
Renal and urinary disorders
Reproductive system and breast disorders
Respiratory, thoracic, and mediastinal disorders
Skin and subcutaneous tissue disorders
Social circumstances
Surgical and medical procedures
Vascular disorders

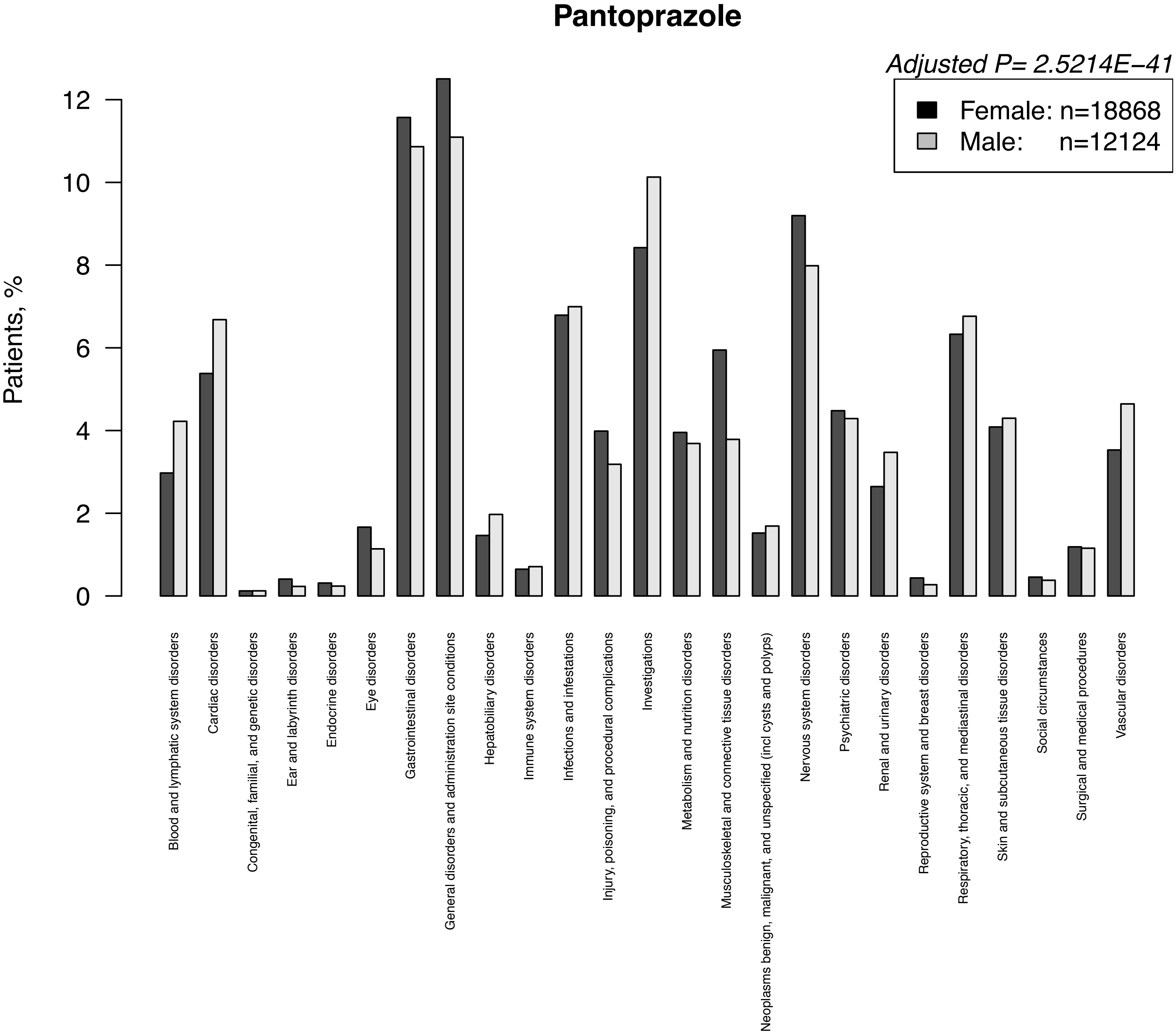

Pantoprazole
Adjusted P= 2.5214E−41
Female: n=18868
Male: n=12124
Patients, %
0
2
4
6
8
10
12
Blood and lymphatic system disorders
Cardiac disorders
Congenital, familial, and genetic disorders
Ear and labyrinth disorders
Endocrine disorders
Eye disorders
Gastrointestinal disorders
General disorders and administration site conditions
Hepatobiliary disorders
Immune system disorders
Infections and infestations
Injury, poisoning, and procedural complications
Investigations
Metabolism and nutrition disorders
Musculoskeletal and connective tissue disorders
Neoplasms benign, malignant, and unspecified (incl cysts and polyps)
Nervous system disorders
Psychiatric disorders
Renal and urinary disorders
Reproductive system and breast disorders
Respiratory, thoracic, and mediastinal disorders
Skin and subcutaneous tissue disorders
Social circumstances
Surgical and medical procedures
Vascular disorders

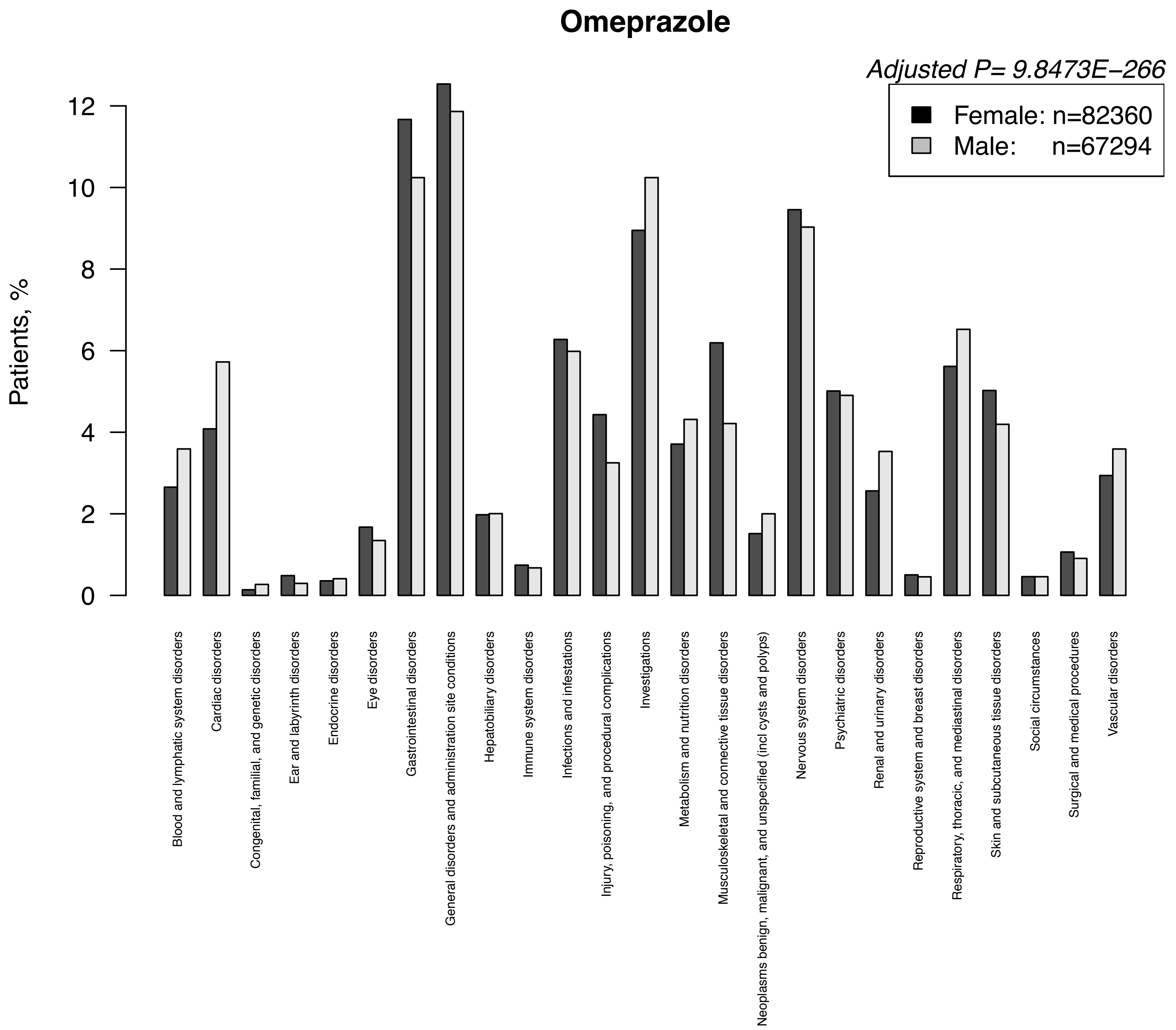
Omeprazole
Adjusted P= 9.8473E−266
Female: n=82360
Male: n=67294
Patients, %
0
2
4
6
8
10
12
Blood and lymphatic system disorders
Cardiac disorders
Congenital, familial, and genetic disorders
Ear and labyrinth disorders
Endocrine disorders
Eye disorders
Gastrointestinal disorders
General disorders and administration site conditions
Hepatobiliary disorders
Immune system disorders
Infections and infestations
Injury, poisoning, and procedural complications
Investigations
Metabolism and nutrition disorders
Musculoskeletal and connective tissue disorders
Neoplasms benign, malignant, and unspecified (incl cysts and polyps)
Nervous system disorders
Psychiatric disorders
Renal and urinary disorders
Reproductive system and breast disorders
Respiratory, thoracic, and mediastinal disorders
Skin and subcutaneous tissue disorders
Social circumstances
Surgical and medical procedures
Vascular disorders

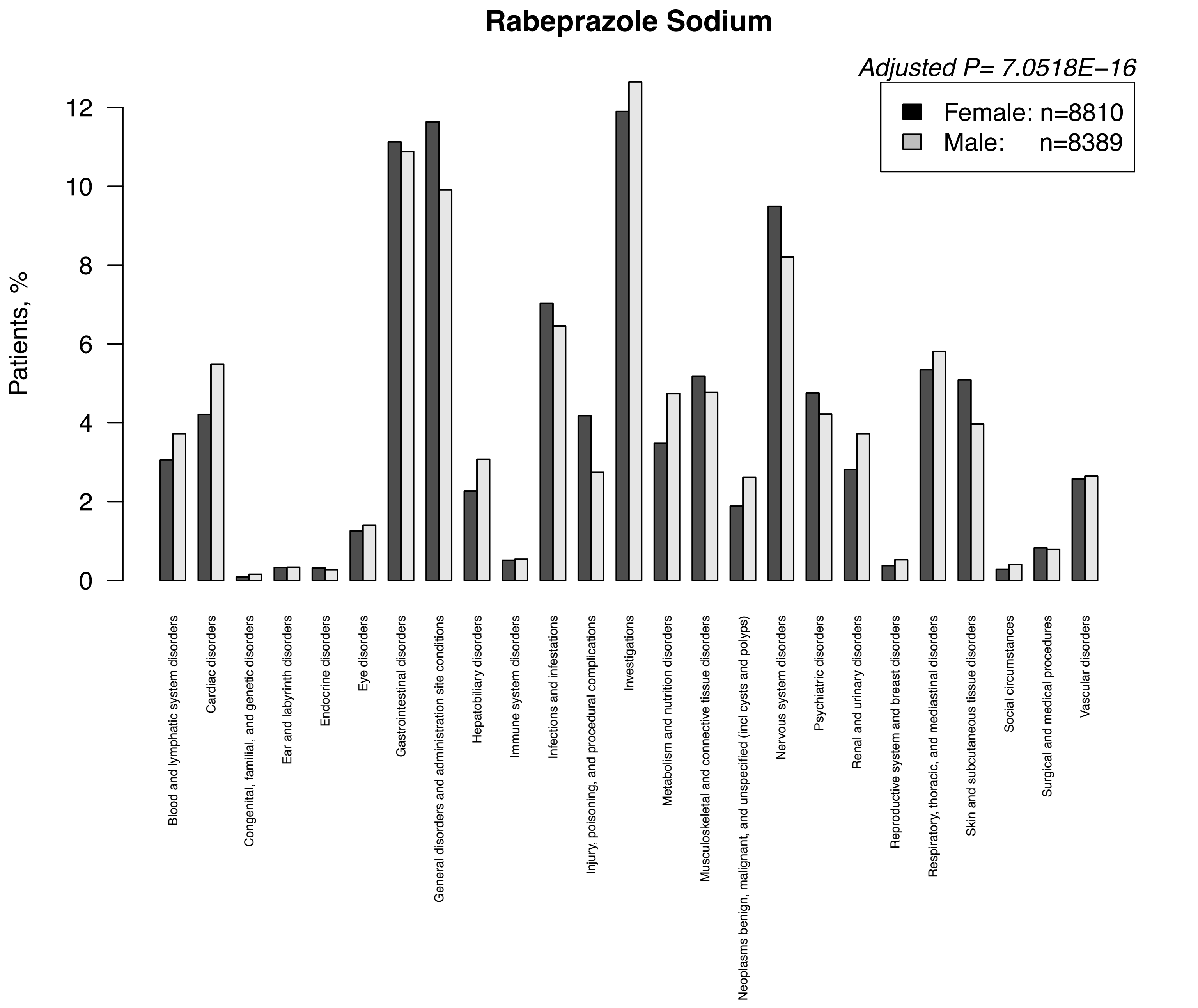
Rabeprazole Sodium
Adjusted P= 7.0518E−16
Female: n=8810
Male: n=8389
Patients, %
0
2
4
6
8
10
12
Blood and lymphatic system disorders
Cardiac disorders
Congenital, familial, and genetic disorders
Ear and labyrinth disorders
Endocrine disorders
Eye disorders
Gastrointestinal disorders
General disorders and administration site conditions
Hepatobiliary disorders
Immune system disorders
Infections and infestations
Injury, poisoning, and procedural complications
Investigations
Metabolism and nutrition disorders
Musculoskeletal and connective tissue disorders
Neoplasms benign, malignant, and unspecified (incl cysts and polyps)
Nervous system disorders
Psychiatric disorders
Renal and urinary disorders
Reproductive system and breast disorders
Respiratory, thoracic, and mediastinal disorders
Skin and subcutaneous tissue disorders
Social circumstances
Surgical and medical procedures
Vascular disorders

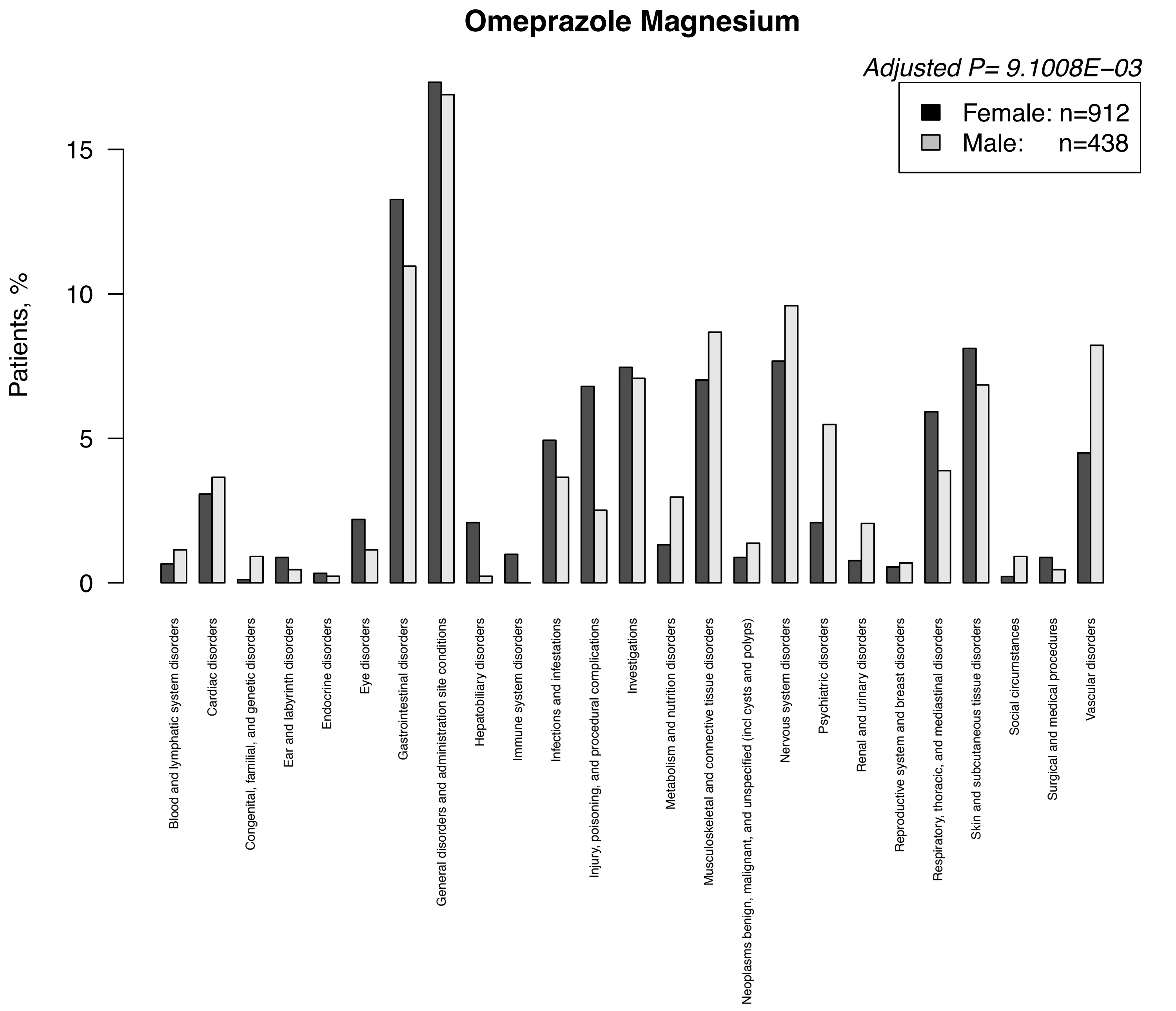

Omeprazole Magnesium
Adjusted P= 9.1008E−03
Female: n=912
Male: n=438
Patients, %
0
5
10
15
Blood and lymphatic system disorders
Cardiac disorders
Congenital, familial, and genetic disorders
Ear and labyrinth disorders
Endocrine disorders
Eye disorders
Gastrointestinal disorders
General disorders and administration site conditions
Hepatobiliary disorders
Immune system disorders
Infections and infestations
Injury, poisoning, and procedural complications
Investigations
Metabolism and nutrition disorders
Musculoskeletal and connective tissue disorders
Neoplasms benign, malignant, and unspecified (incl cysts and polyps)
Nervous system disorders
Psychiatric disorders
Renal and urinary disorders
Reproductive system and breast disorders
Respiratory, thoracic, and mediastinal disorders
Skin and subcutaneous tissue disorders
Social circumstances
Surgical and medical procedures
Vascular disorders

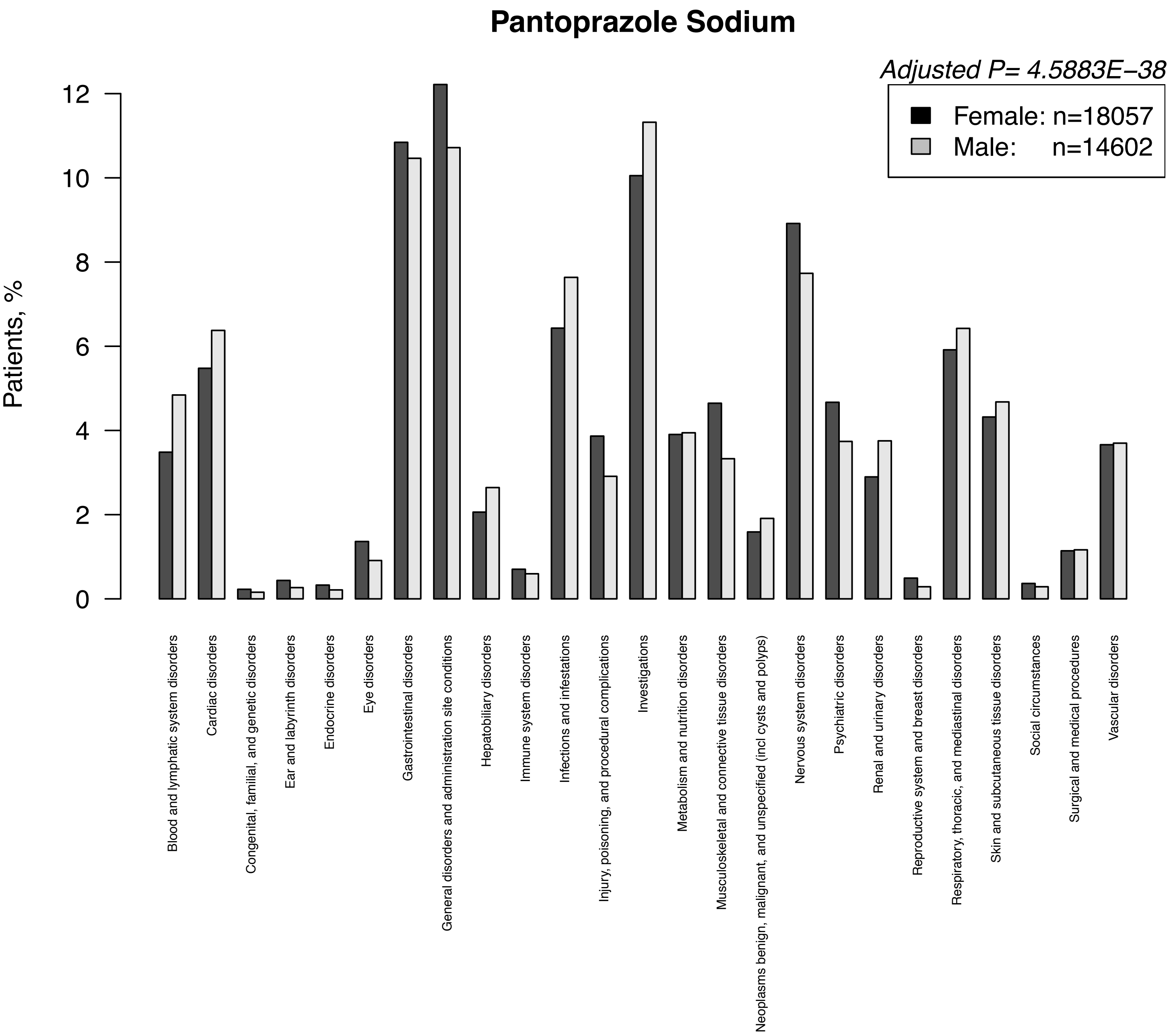
Pantoprazole Sodium
Adjusted P= 4.5883E−38
Female: n=18057
Male: n=14602
Patients, %
0
2
4
6
8
10
12
Blood and lymphatic system disorders
Cardiac disorders
Congenital, familial, and genetic disorders
Ear and labyrinth disorders
Endocrine disorders
Eye disorders
Gastrointestinal disorders
General disorders and administration site conditions
Hepatobiliary disorders
Immune system disorders
Infections and infestations
Injury, poisoning, and procedural complications
Investigations
Metabolism and nutrition disorders
Musculoskeletal and connective tissue disorders
Neoplasms benign, malignant, and unspecified (incl cysts and polyps)
Nervous system disorders
Psychiatric disorders
Renal and urinary disorders
Reproductive system and breast disorders
Respiratory, thoracic, and mediastinal disorders
Skin and subcutaneous tissue disorders
Social circumstances
Surgical and medical procedures
Vascular disorders

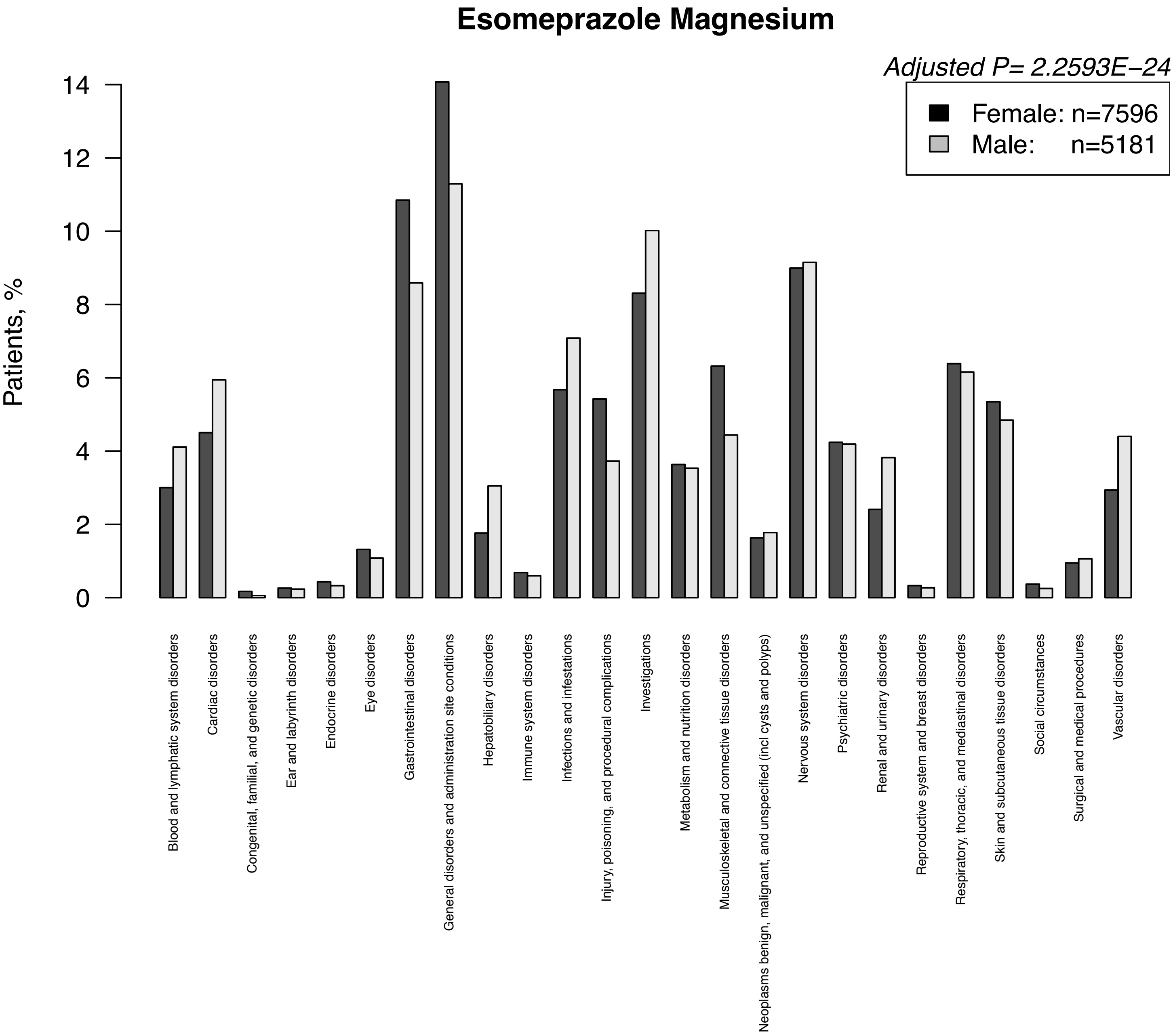

Esomeprazole Magnesium
Adjusted P= 2.2593E−24
Female: n=7596
Male: n=5181
Patients, %
0
2
4
6
8
10
12
14
Blood and lymphatic system disorders
Cardiac disorders
Congenital, familial, and genetic disorders
Ear and labyrinth disorders
Endocrine disorders
Eye disorders
Gastrointestinal disorders
General disorders and administration site conditions
Hepatobiliary disorders
Immune system disorders
Infections and infestations
Injury, poisoning, and procedural complications
Investigations
Metabolism and nutrition disorders
Musculoskeletal and connective tissue disorders
Neoplasms benign, malignant, and unspecified (incl cysts and polyps)
Nervous system disorders
Psychiatric disorders
Renal and urinary disorders
Reproductive system and breast disorders
Respiratory, thoracic, and mediastinal disorders
Skin and subcutaneous tissue disorders
Social circumstances
Surgical and medical procedures
Vascular disorders

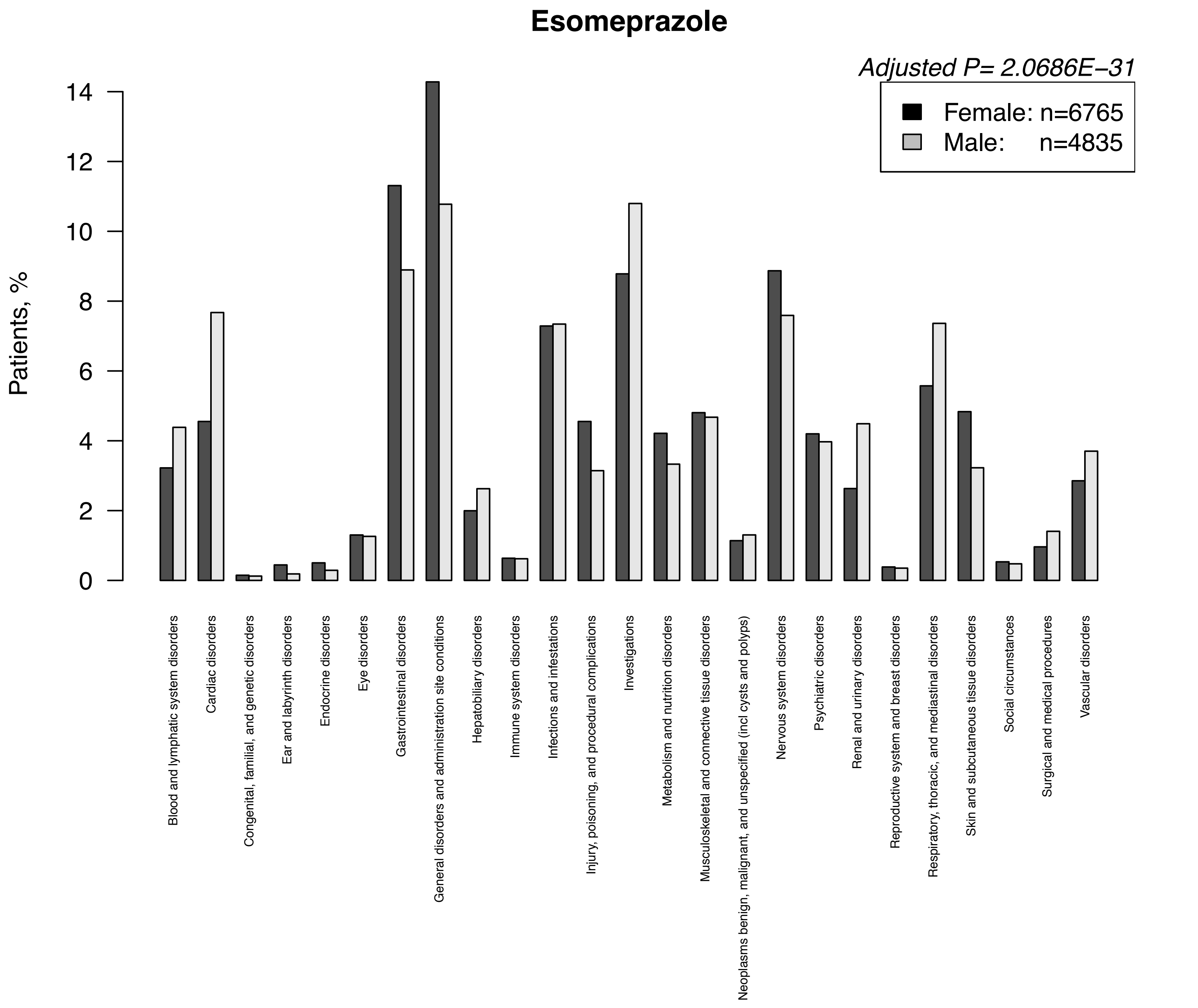
Esomeprazole
Adjusted P= 2.0686E−31
Female: n=6765
Male: n=4835
Patients, %
0
2
4
6
8
10
12
14
Blood and lymphatic system disorders
Cardiac disorders
Congenital, familial, and genetic disorders
Ear and labyrinth disorders
Endocrine disorders
Eye disorders
Gastrointestinal disorders
General disorders and administration site conditions
Hepatobiliary disorders
Immune system disorders
Infections and infestations
Injury, poisoning, and procedural complications
Investigations
Metabolism and nutrition disorders
Musculoskeletal and connective tissue disorders
Neoplasms benign, malignant, and unspecified (incl cysts and polyps)
Nervous system disorders
Psychiatric disorders
Renal and urinary disorders
Reproductive system and breast disorders
Respiratory, thoracic, and mediastinal disorders
Skin and subcutaneous tissue disorders
Social circumstances
Surgical and medical procedures
Vascular disorders

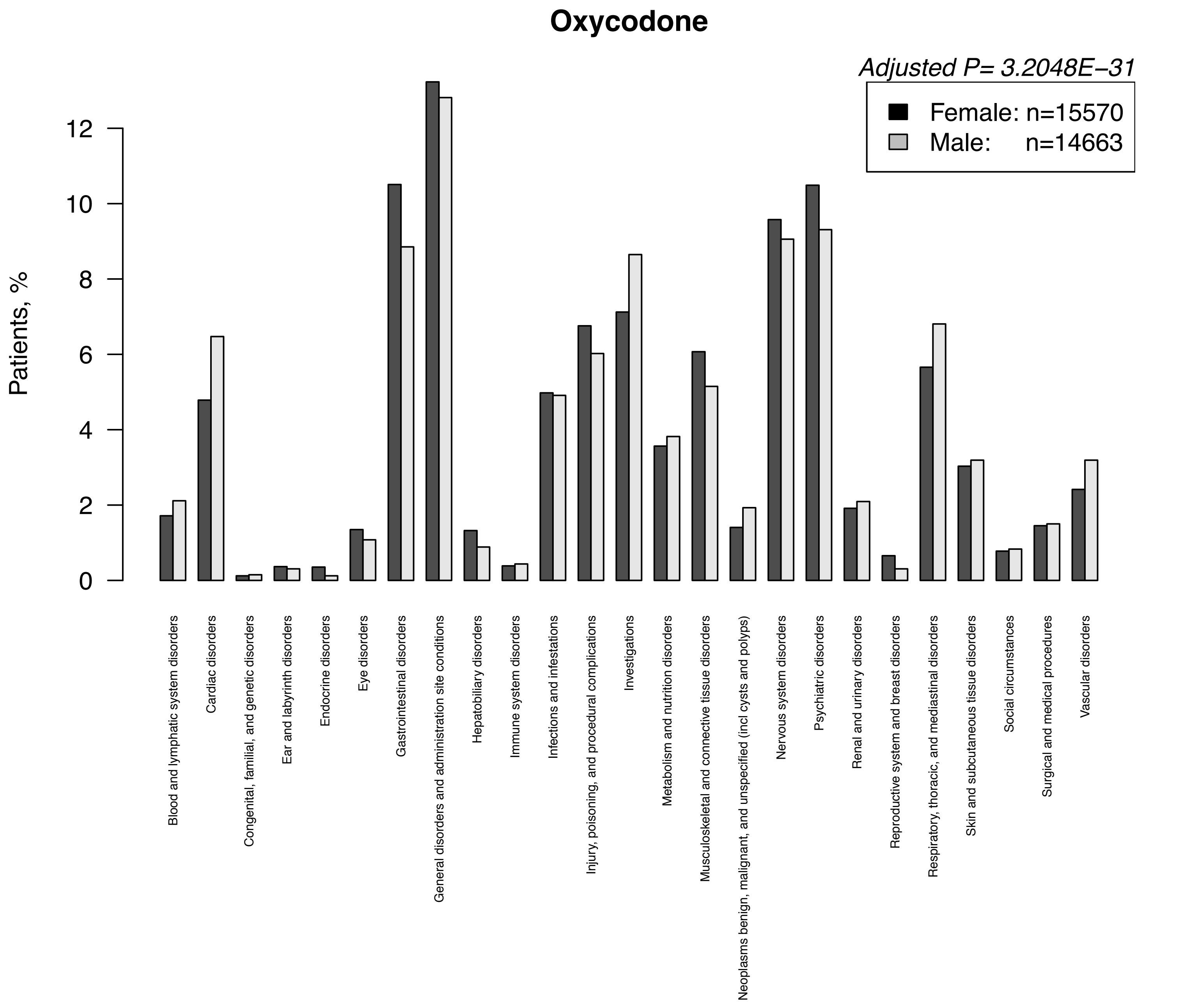

Oxycodone
Adjusted P= 3.2048E−31
Female: n=15570
Male: n=14663
Patients, %
0
2
4
6
8
10
12
Blood and lymphatic system disorders
Cardiac disorders
Congenital, familial, and genetic disorders
Ear and labyrinth disorders
Endocrine disorders
Eye disorders
Gastrointestinal disorders
General disorders and administration site conditions
Hepatobiliary disorders
Immune system disorders
Infections and infestations
Injury, poisoning, and procedural complications
Investigations
Metabolism and nutrition disorders
Musculoskeletal and connective tissue disorders
Neoplasms benign, malignant, and unspecified (incl cysts and polyps)
Nervous system disorders
Psychiatric disorders
Renal and urinary disorders
Reproductive system and breast disorders
Respiratory, thoracic, and mediastinal disorders
Skin and subcutaneous tissue disorders
Social circumstances
Surgical and medical procedures
Vascular disorders

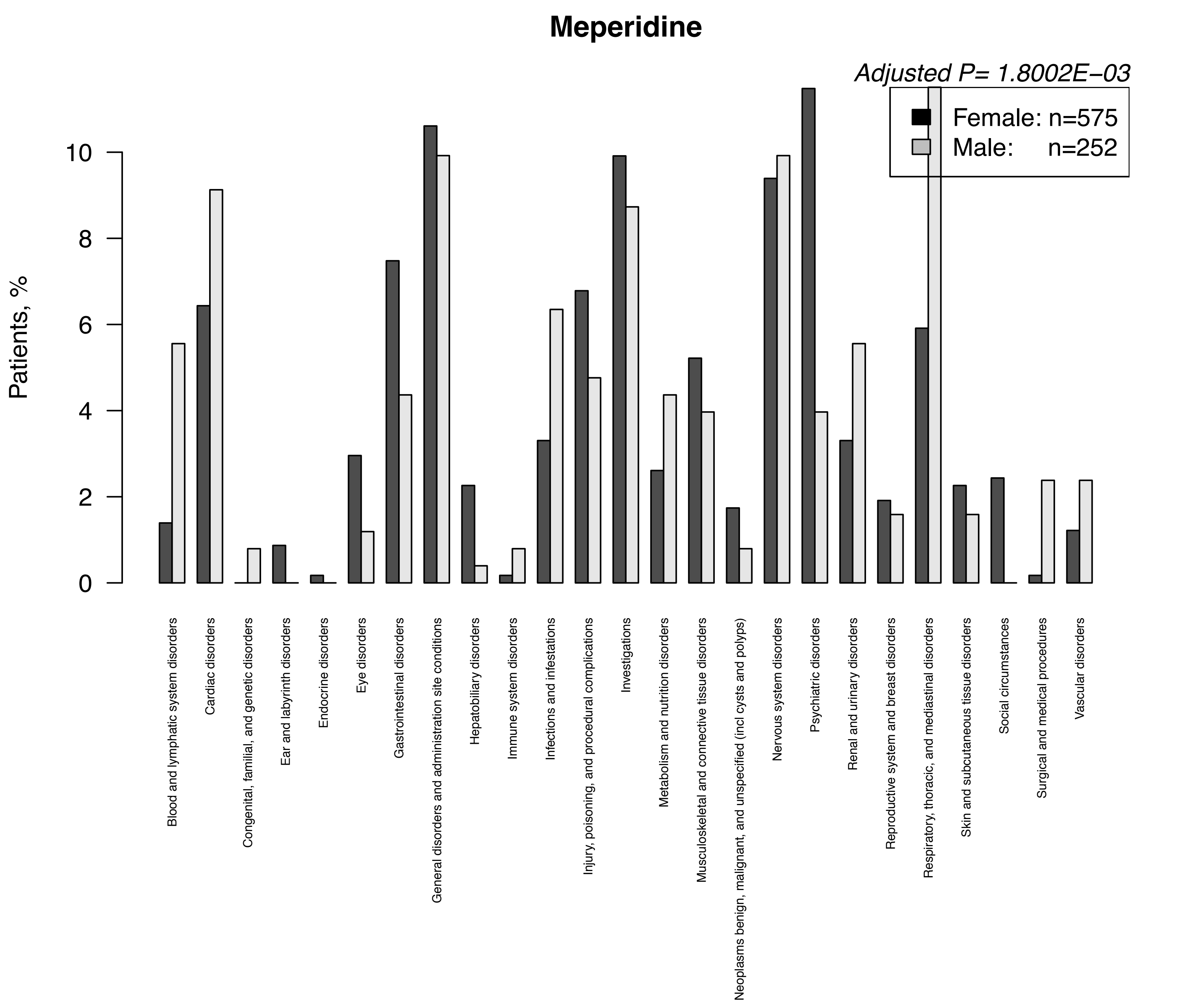
Meperidine
Adjusted P= 1.8002E−03
Female: n=575
Male: n=252
Patients, %
0
2
4
6
8
10
Blood and lymphatic system disorders
Cardiac disorders
Congenital, familial, and genetic disorders
Ear and labyrinth disorders
Endocrine disorders
Eye disorders
Gastrointestinal disorders
General disorders and administration site conditions
Hepatobiliary disorders
Immune system disorders
Infections and infestations
Injury, poisoning, and procedural complications
Investigations
Metabolism and nutrition disorders
Musculoskeletal and connective tissue disorders
Neoplasms benign, malignant, and unspecified (incl cysts and polyps)
Nervous system disorders
Psychiatric disorders
Renal and urinary disorders
Reproductive system and breast disorders
Respiratory, thoracic, and mediastinal disorders
Skin and subcutaneous tissue disorders
Social circumstances
Surgical and medical procedures
Vascular disorders

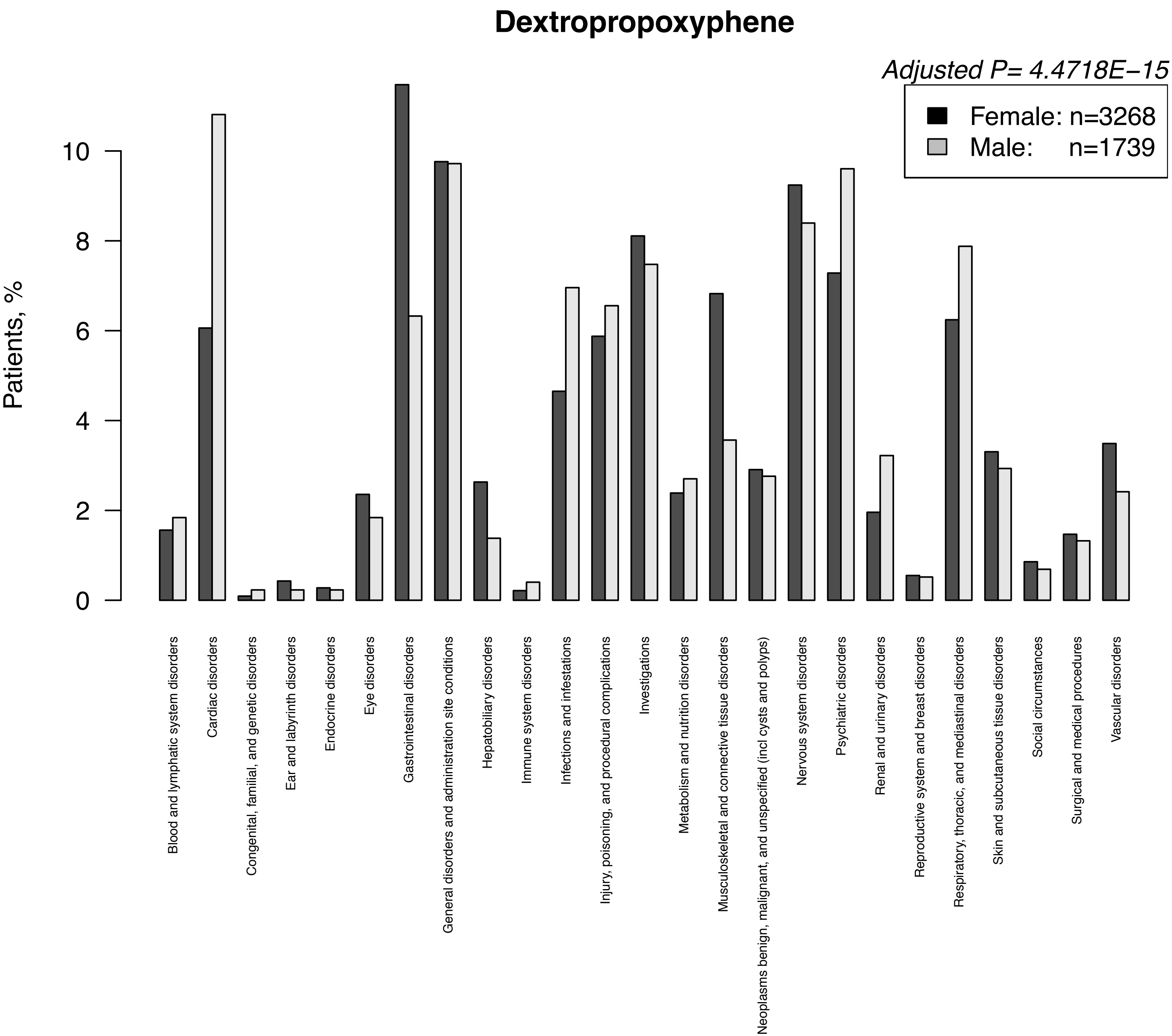

Dextropropoxyphene
Adjusted P= 4.4718E−15
Female: n=3268
Male: n=1739
Patients, %
0
2
4
6
8
10
Blood and lymphatic system disorders
Cardiac disorders
Congenital, familial, and genetic disorders
Ear and labyrinth disorders
Endocrine disorders
Eye disorders
Gastrointestinal disorders
General disorders and administration site conditions
Hepatobiliary disorders
Immune system disorders
Infections and infestations
Injury, poisoning, and procedural complications
Investigations
Metabolism and nutrition disorders
Musculoskeletal and connective tissue disorders
Neoplasms benign, malignant, and unspecified (incl cysts and polyps)
Nervous system disorders
Psychiatric disorders
Renal and urinary disorders
Reproductive system and breast disorders
Respiratory, thoracic, and mediastinal disorders
Skin and subcutaneous tissue disorders
Social circumstances
Surgical and medical procedures
Vascular disorders

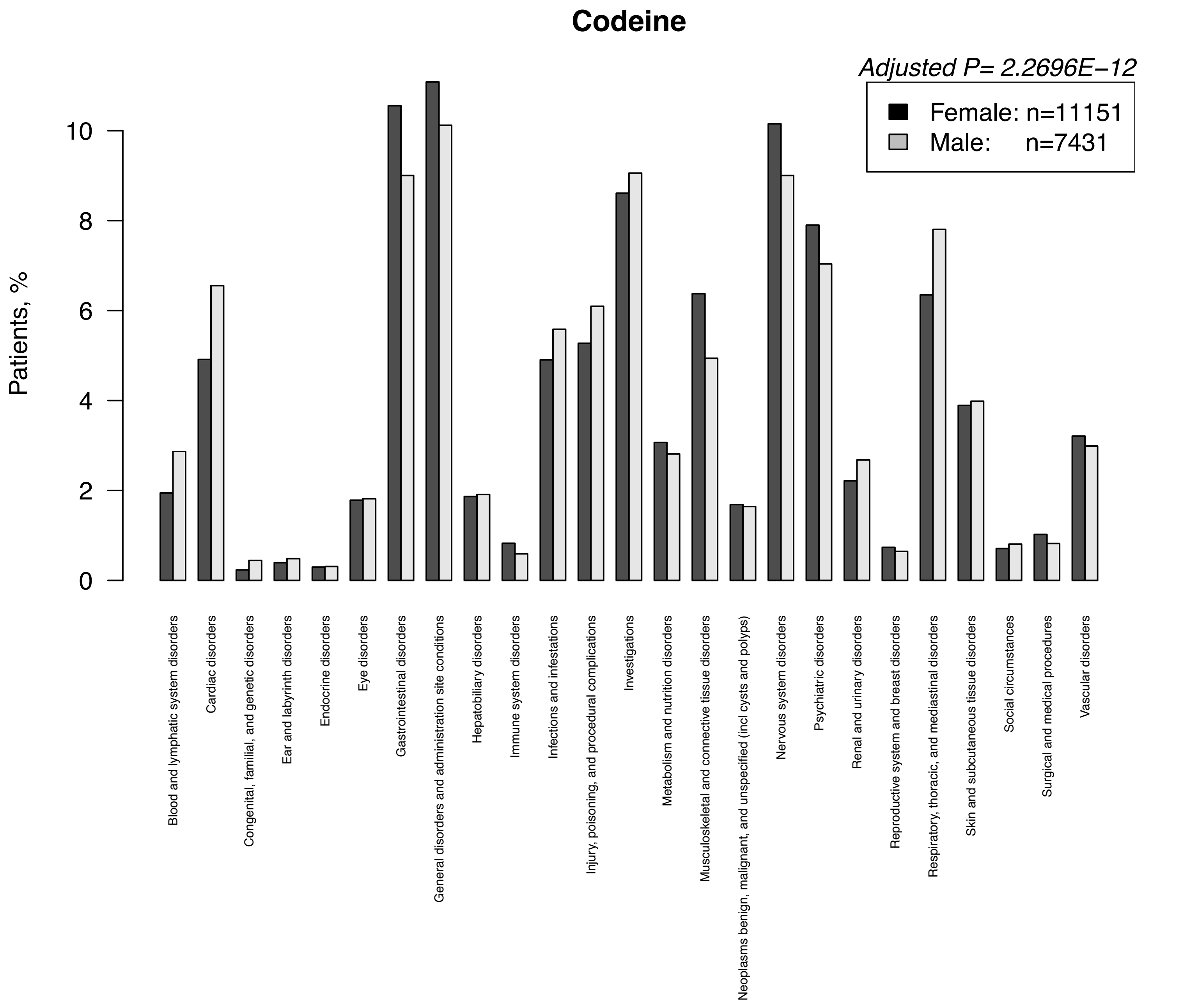
Codeine
Adjusted P= 2.2696E−12
Female: n=11151
Male: n=7431
Patients, %
0
2
4
6
8
10
Blood and lymphatic system disorders
Cardiac disorders
Congenital, familial, and genetic disorders
Ear and labyrinth disorders
Endocrine disorders
Eye disorders
Gastrointestinal disorders
General disorders and administration site conditions
Hepatobiliary disorders
Immune system disorders
Infections and infestations
Injury, poisoning, and procedural complications
Investigations
Metabolism and nutrition disorders
Musculoskeletal and connective tissue disorders
Neoplasms benign, malignant, and unspecified (incl cysts and polyps)
Nervous system disorders
Psychiatric disorders
Renal and urinary disorders
Reproductive system and breast disorders
Respiratory, thoracic, and mediastinal disorders
Skin and subcutaneous tissue disorders
Social circumstances
Surgical and medical procedures
Vascular disorders

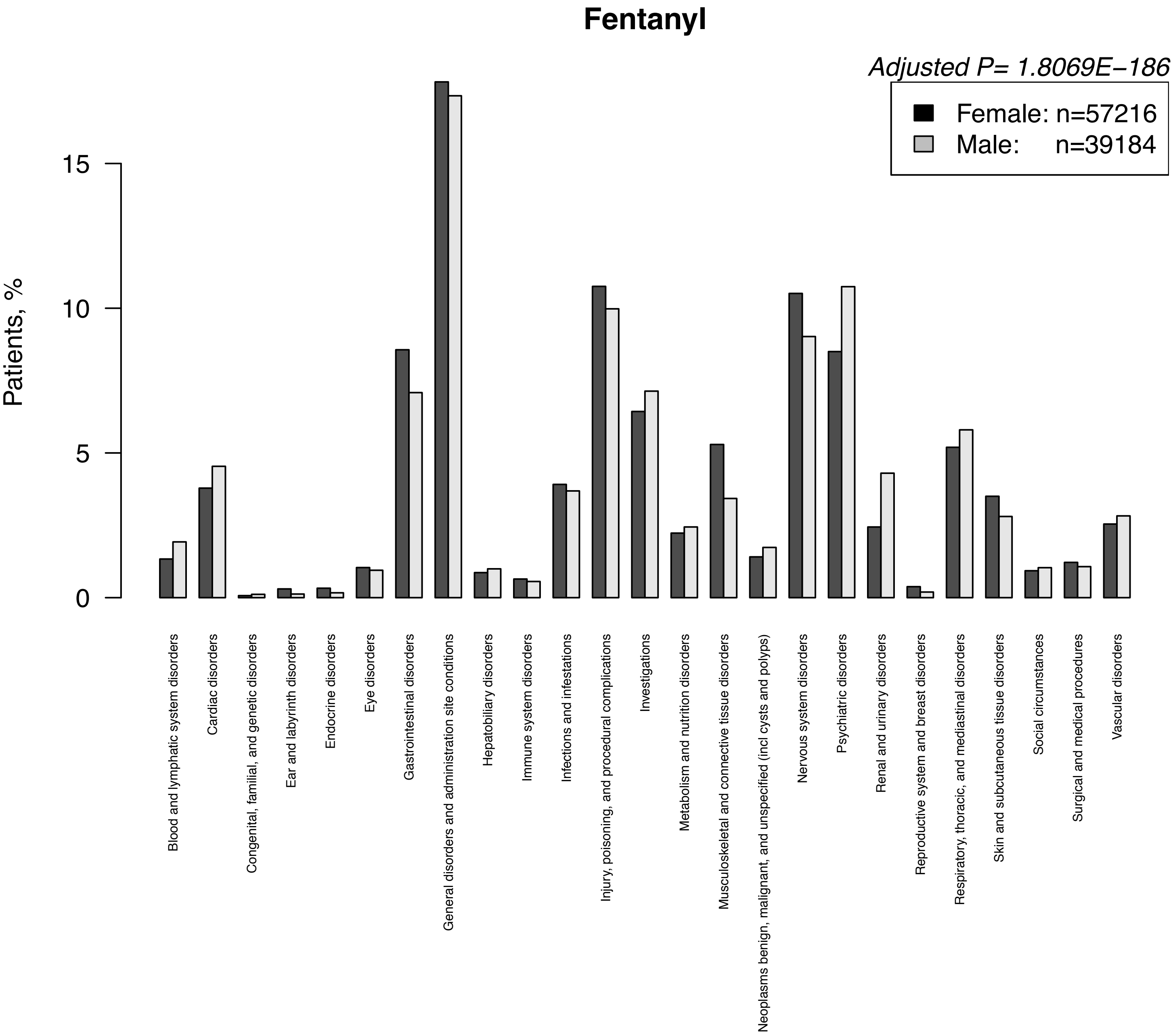
Fentanyl
Adjusted P= 1.8069E−186
Female: n=57216
Male: n=39184
Patients, %
0
5
10
15
Blood and lymphatic system disorders
Cardiac disorders
Congenital, familial, and genetic disorders
Ear and labyrinth disorders
Endocrine disorders
Eye disorders
Gastrointestinal disorders
General disorders and administration site conditions
Hepatobiliary disorders
Immune system disorders
Infections and infestations
Injury, poisoning, and procedural complications
Investigations
Metabolism and nutrition disorders
Musculoskeletal and connective tissue disorders
Neoplasms benign, malignant, and unspecified (incl cysts and polyps)
Nervous system disorders
Psychiatric disorders
Renal and urinary disorders
Reproductive system and breast disorders
Respiratory, thoracic, and mediastinal disorders
Skin and subcutaneous tissue disorders
Social circumstances
Surgical and medical procedures
Vascular disorders

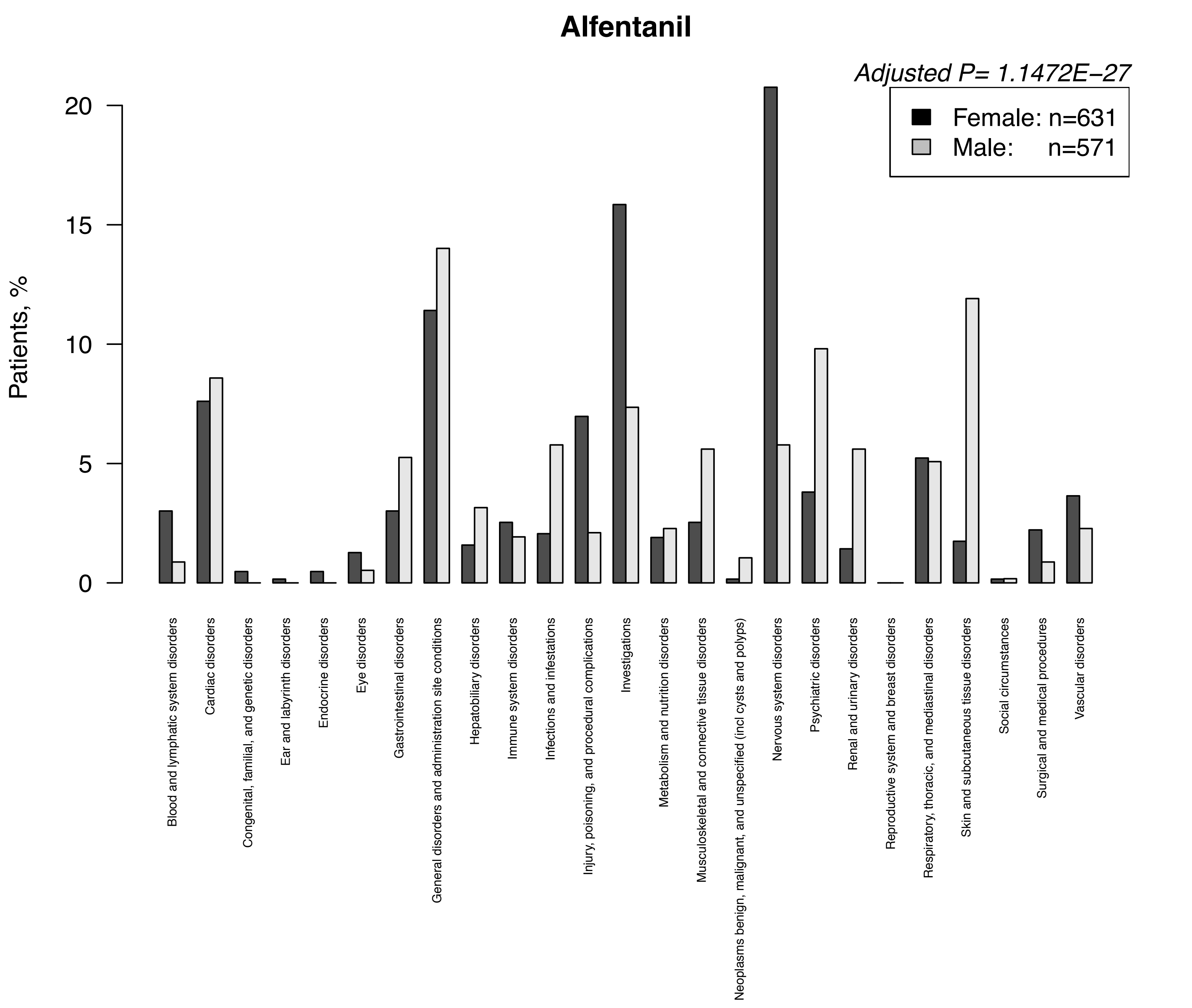
Alfentanil
Adjusted P= 1.1472E−27
Female: n=631
Male: n=571
Patients, %
0
5
10
15
20
Blood and lymphatic system disorders
Cardiac disorders
Congenital, familial, and genetic disorders
Ear and labyrinth disorders
Endocrine disorders
Eye disorders
Gastrointestinal disorders
General disorders and administration site conditions
Hepatobiliary disorders
Immune system disorders
Infections and infestations
Injury, poisoning, and procedural complications
Investigations
Metabolism and nutrition disorders
Musculoskeletal and connective tissue disorders
Neoplasms benign, malignant, and unspecified (incl cysts and polyps)
Nervous system disorders
Psychiatric disorders
Renal and urinary disorders
Reproductive system and breast disorders
Respiratory, thoracic, and mediastinal disorders
Skin and subcutaneous tissue disorders
Social circumstances
Surgical and medical procedures
Vascular disorders

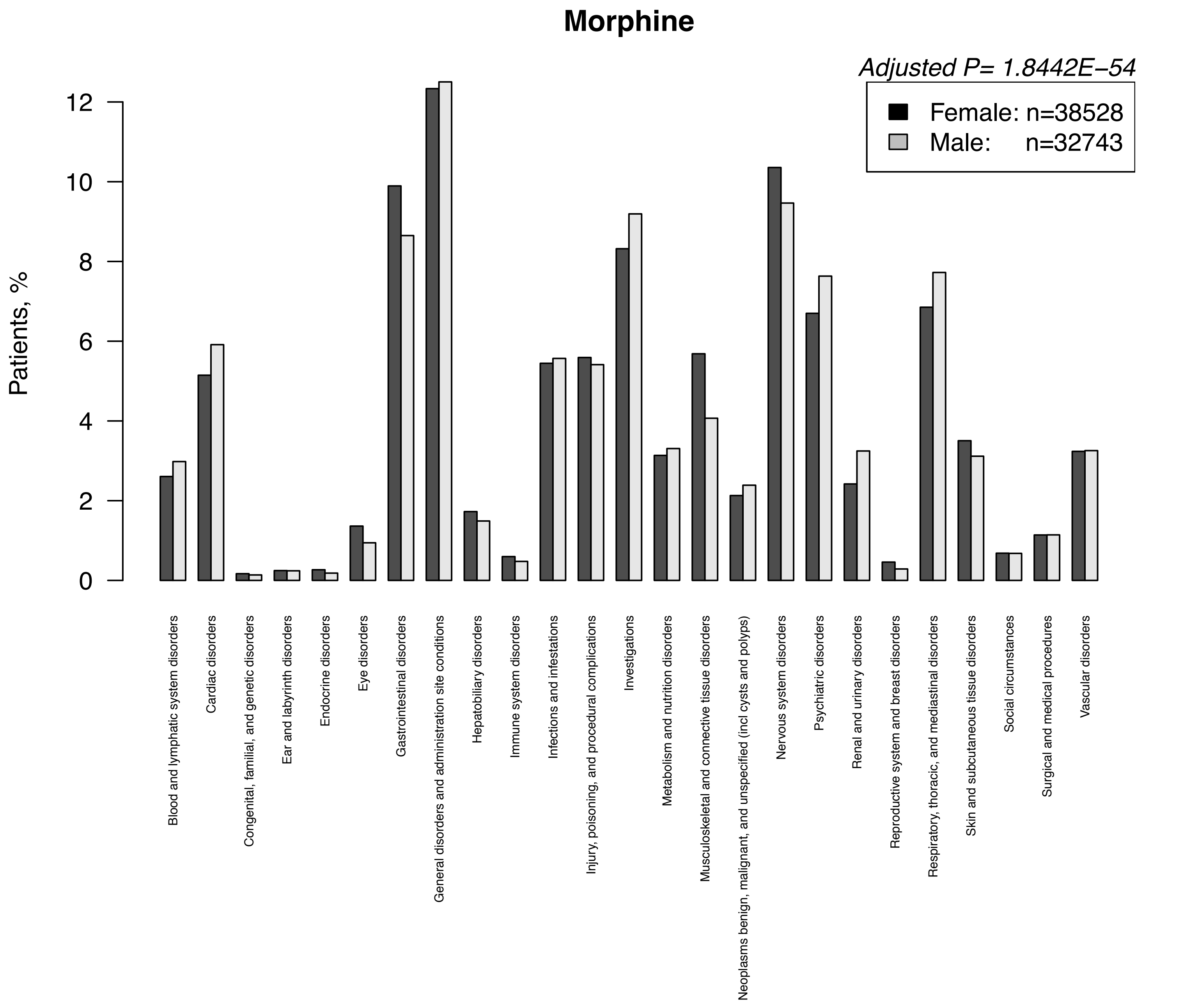

Morphine
Adjusted P= 1.8442E−54
Female: n=38528
Male: n=32743
Patients, %
0
2
4
6
8
10
12
Blood and lymphatic system disorders
Cardiac disorders
Congenital, familial, and genetic disorders
Ear and labyrinth disorders
Endocrine disorders
Eye disorders
Gastrointestinal disorders
General disorders and administration site conditions
Hepatobiliary disorders
Immune system disorders
Infections and infestations
Injury, poisoning, and procedural complications
Investigations
Metabolism and nutrition disorders
Musculoskeletal and connective tissue disorders
Neoplasms benign, malignant, and unspecified (incl cysts and polyps)
Nervous system disorders
Psychiatric disorders
Renal and urinary disorders
Reproductive system and breast disorders
Respiratory, thoracic, and mediastinal disorders
Skin and subcutaneous tissue disorders
Social circumstances
Surgical and medical procedures
Vascular disorders

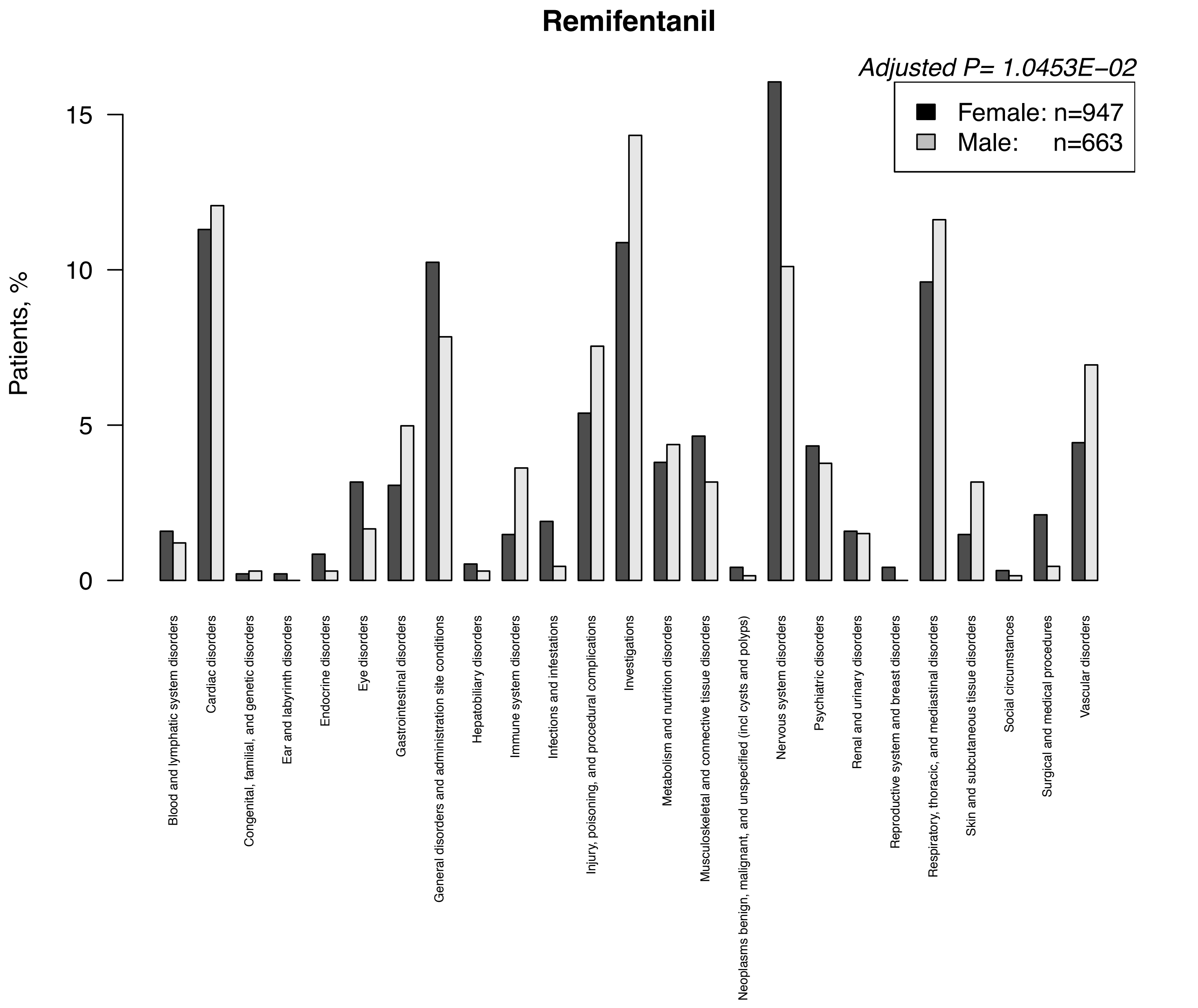
Remifentanil
Adjusted P= 1.0453E−02
Female: n=947
Male: n=663
Patients, %
15
10
5
0
Blood and lymphatic system disorders
Cardiac disorders
Congenital, familial, and genetic disorders
Ear and labyrinth disorders
Endocrine disorders
Eye disorders
Gastrointestinal disorders
General disorders and administration site conditions
Hepatobiliary disorders
Immune system disorders
Infections and infestations
Injury, poisoning, and procedural complications
Investigations
Metabolism and nutrition disorders
Musculoskeletal and connective tissue disorders
Neoplasms benign, malignant, and unspecified (incl cysts and polyps)
Nervous system disorders
Psychiatric disorders
Renal and urinary disorders
Reproductive system and breast disorders
Respiratory, thoracic, and mediastinal disorders
Skin and subcutaneous tissue disorders
Social circumstances
Surgical and medical procedures
Vascular disorders

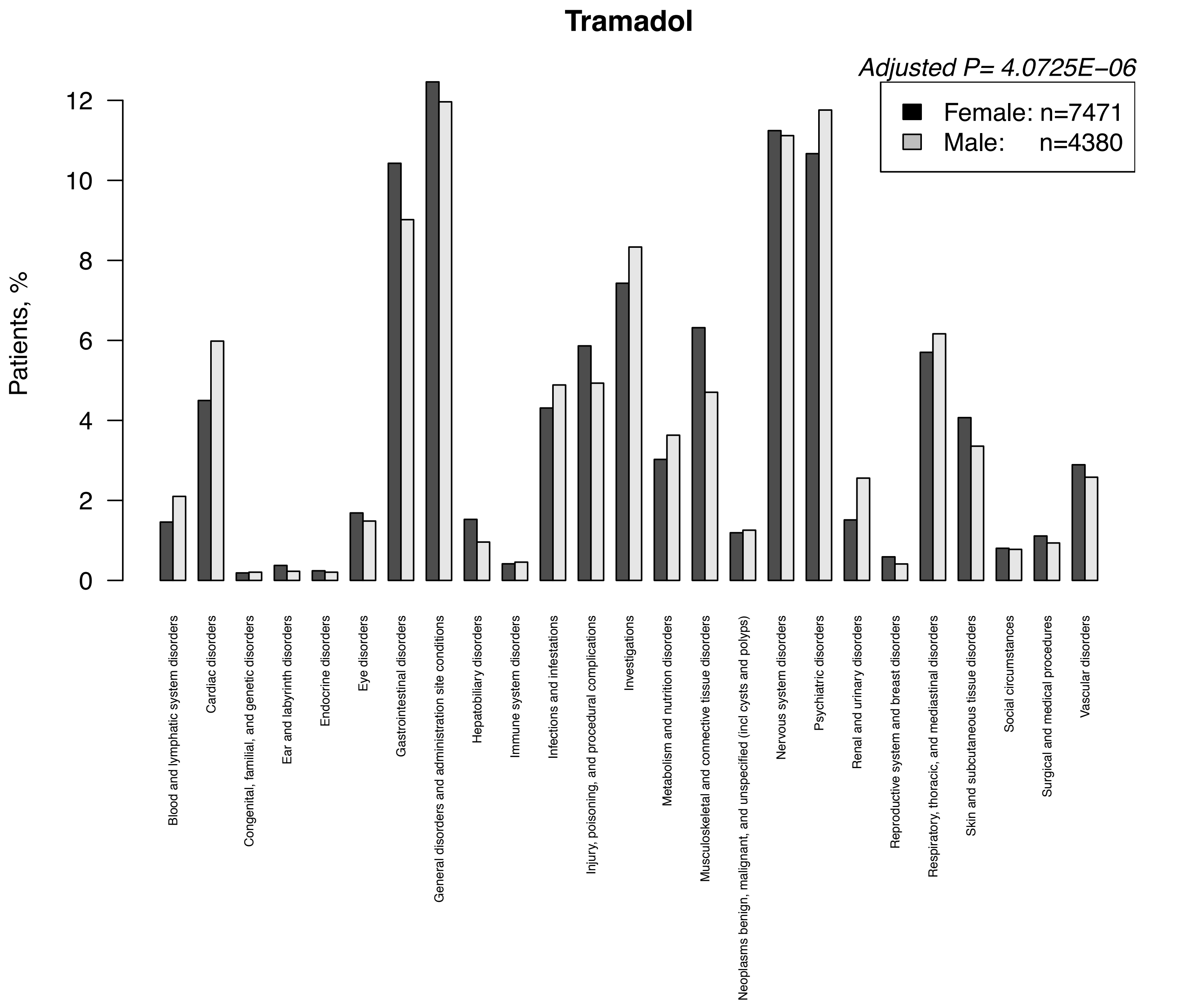

Tramadol
Adjusted P= 4.0725E−06
Female: n=7471
Male: n=4380
Patients, %
0
2
4
6
8
10
12
Blood and lymphatic system disorders
Cardiac disorders
Congenital, familial, and genetic disorders
Ear and labyrinth disorders
Endocrine disorders
Eye disorders
Gastrointestinal disorders
General disorders and administration site conditions
Hepatobiliary disorders
Immune system disorders
Infections and infestations
Injury, poisoning, and procedural complications
Investigations
Metabolism and nutrition disorders
Musculoskeletal and connective tissue disorders
Neoplasms benign, malignant, and unspecified (incl cysts and polyps)
Nervous system disorders
Psychiatric disorders
Renal and urinary disorders
Reproductive system and breast disorders
Respiratory, thoracic, and mediastinal disorders
Skin and subcutaneous tissue disorders
Social circumstances
Surgical and medical procedures
Vascular disorders

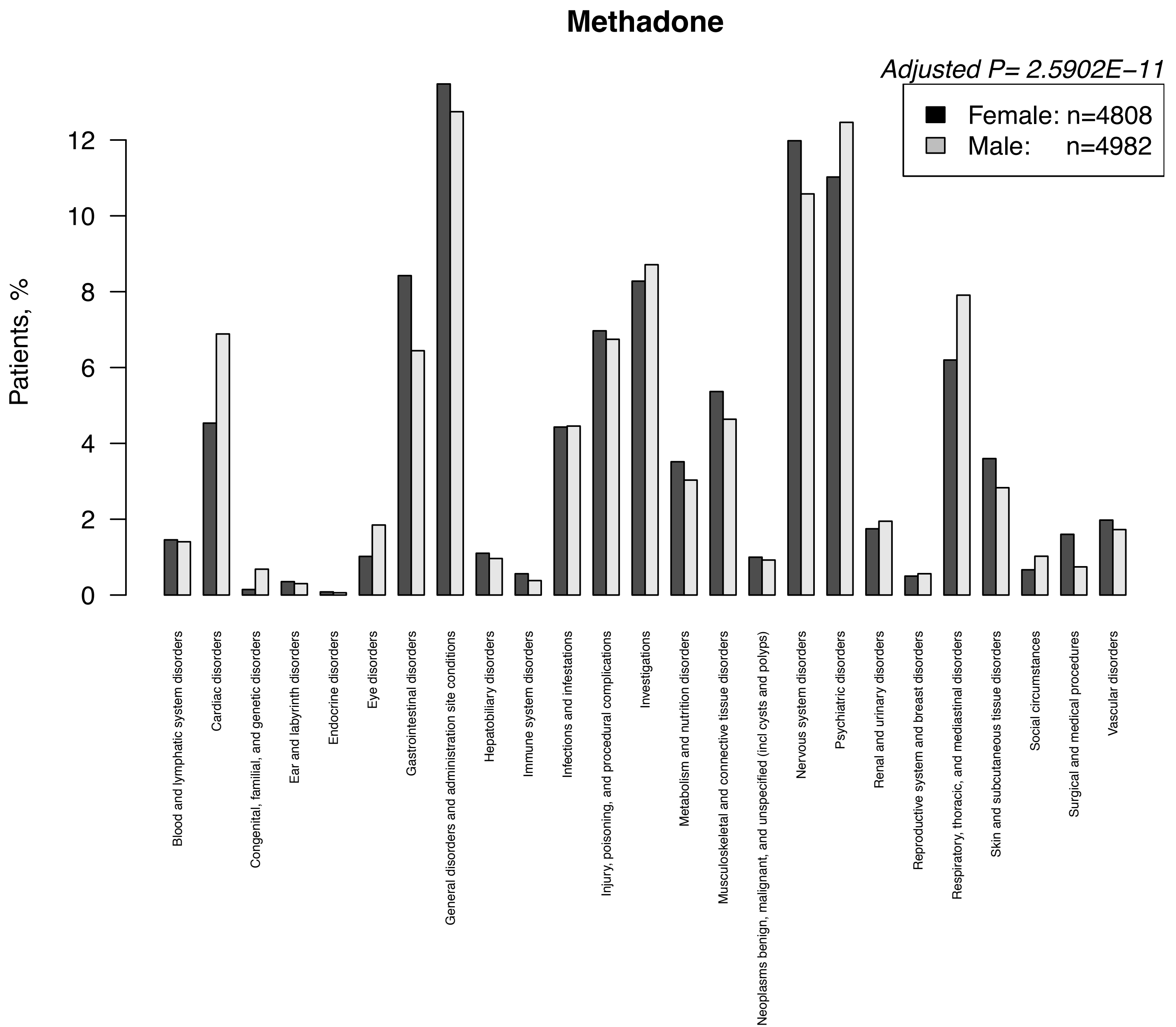
Methadone
Adjusted P= 2.5902E−11
Female: n=4808
Male: n=4982
Patients, %
0
2
4
6
8
10
12
Blood and lymphatic system disorders
Cardiac disorders
Congenital, familial, and genetic disorders
Ear and labyrinth disorders
Endocrine disorders
Eye disorders
Gastrointestinal disorders
General disorders and administration site conditions
Hepatobiliary disorders
Immune system disorders
Infections and infestations
Injury, poisoning, and procedural complications
Investigations
Metabolism and nutrition disorders
Musculoskeletal and connective tissue disorders
Neoplasms benign, malignant, and unspecified (incl cysts and polyps)
Nervous system disorders
Psychiatric disorders
Renal and urinary disorders
Reproductive system and breast disorders
Respiratory, thoracic, and mediastinal disorders
Skin and subcutaneous tissue disorders
Social circumstances
Surgical and medical procedures
Vascular disorders

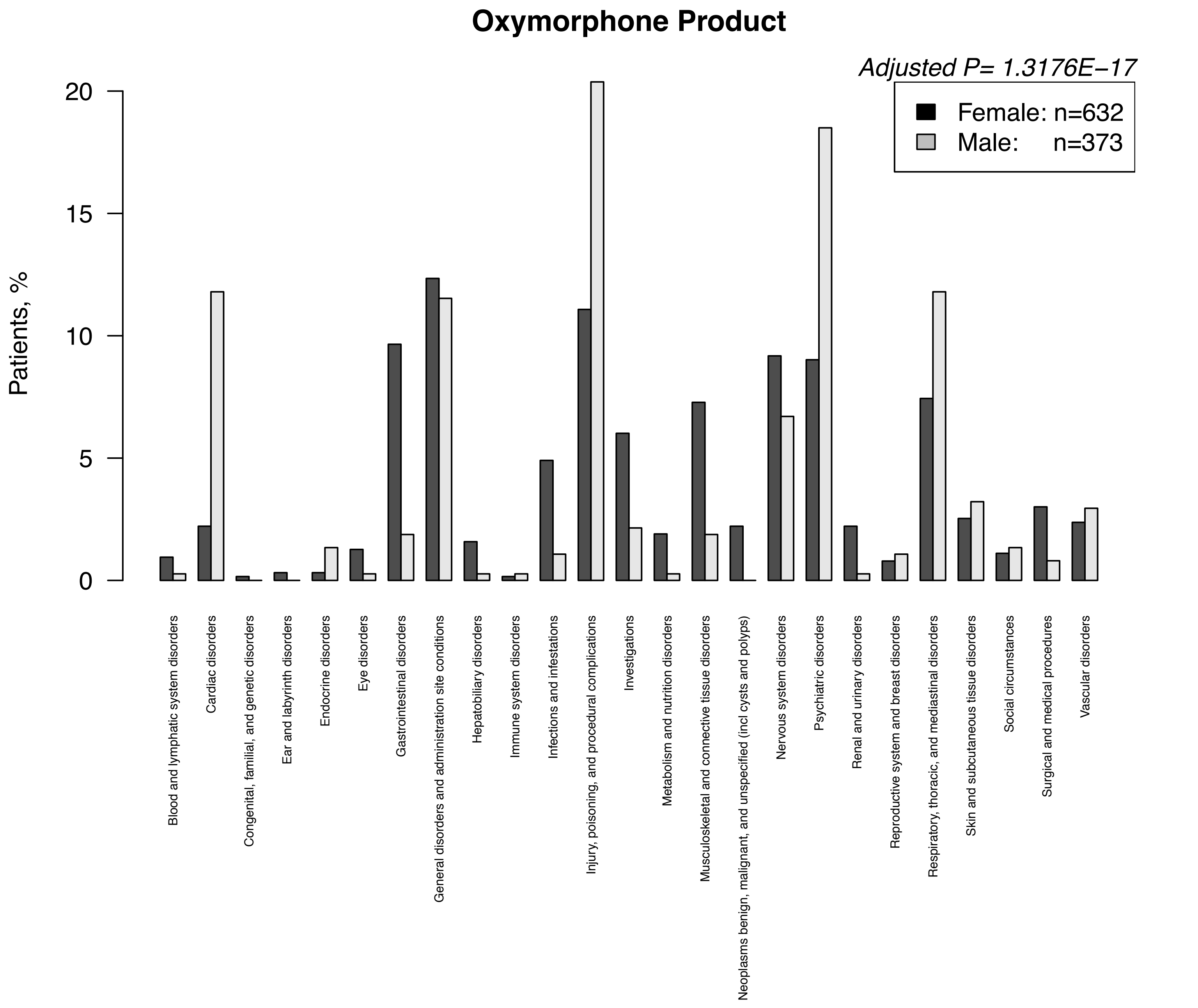
Oxymorphone Product
Adjusted P= 1.3176E−17
Female: n=632
Male: n=373
Patients, %
0
5
10
15
20
Blood and lymphatic system disorders
Cardiac disorders
Congenital, familial, and genetic disorders
Ear and labyrinth disorders
Endocrine disorders
Eye disorders
Gastrointestinal disorders
General disorders and administration site conditions
Hepatobiliary disorders
Immune system disorders
Infections and infestations
Injury, poisoning, and procedural complications
Investigations
Metabolism and nutrition disorders
Musculoskeletal and connective tissue disorders
Neoplasms benign, malignant, and unspecified (incl cysts and polyps)
Nervous system disorders
Psychiatric disorders
Renal and urinary disorders
Reproductive system and breast disorders
Respiratory, thoracic, and mediastinal disorders
Skin and subcutaneous tissue disorders
Social circumstances
Surgical and medical procedures
Vascular disorders

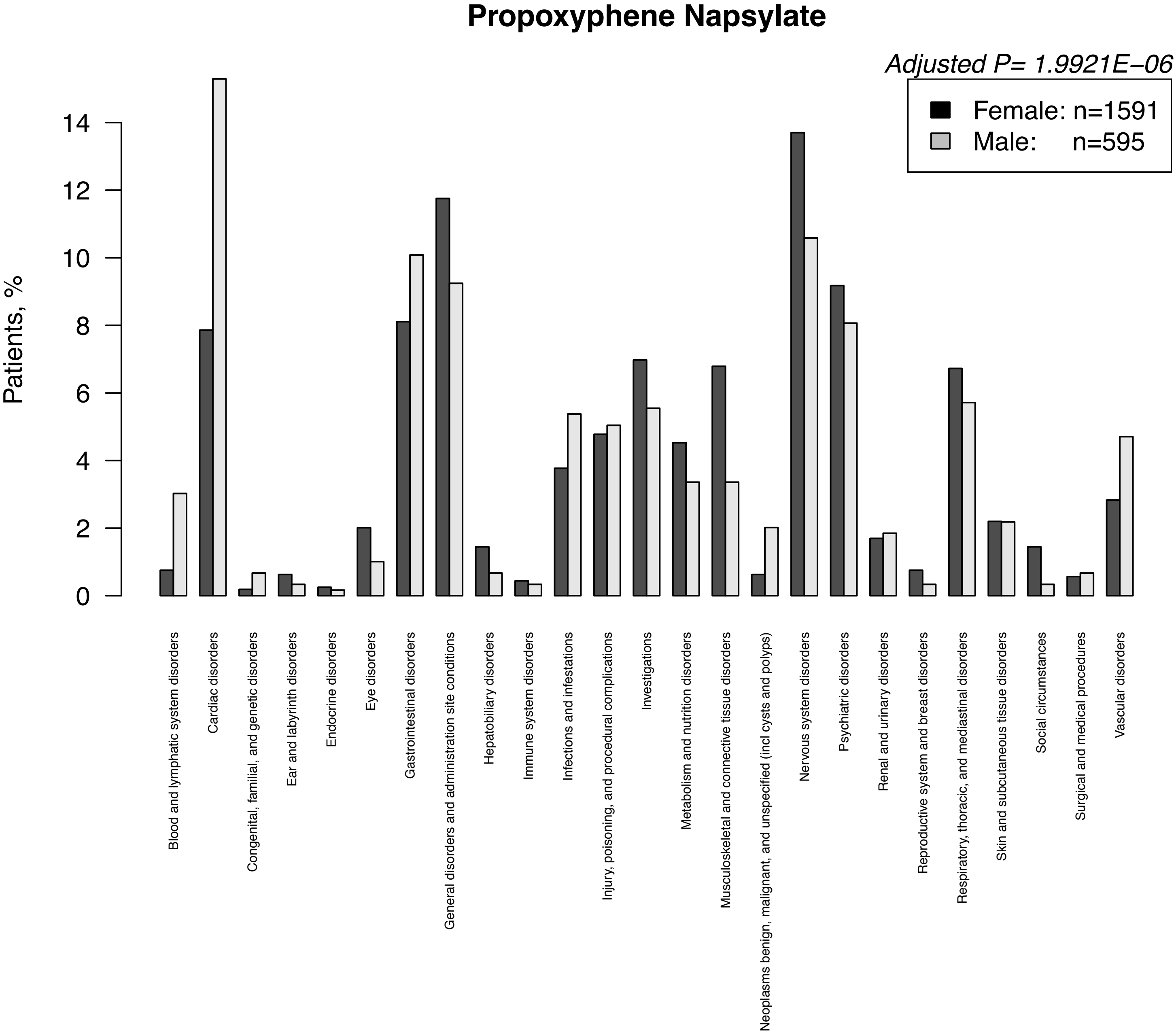

Propoxyphene Napsylate
Adjusted P= 1.9921E−06
Female: n=1591
Male: n=595
Patients, %
0
2
4
6
8
10
12
14
Blood and lymphatic system disorders
Cardiac disorders
Congenital, familial, and genetic disorders
Ear and labyrinth disorders
Endocrine disorders
Eye disorders
Gastrointestinal disorders
General disorders and administration site conditions
Hepatobiliary disorders
Immune system disorders
Infections and infestations
Injury, poisoning, and procedural complications
Investigations
Metabolism and nutrition disorders
Musculoskeletal and connective tissue disorders
Neoplasms benign, malignant, and unspecified (incl cysts and polyps)
Nervous system disorders
Psychiatric disorders
Renal and urinary disorders
Reproductive system and breast disorders
Respiratory, thoracic, and mediastinal disorders
Skin and subcutaneous tissue disorders
Social circumstances
Surgical and medical procedures
Vascular disorders

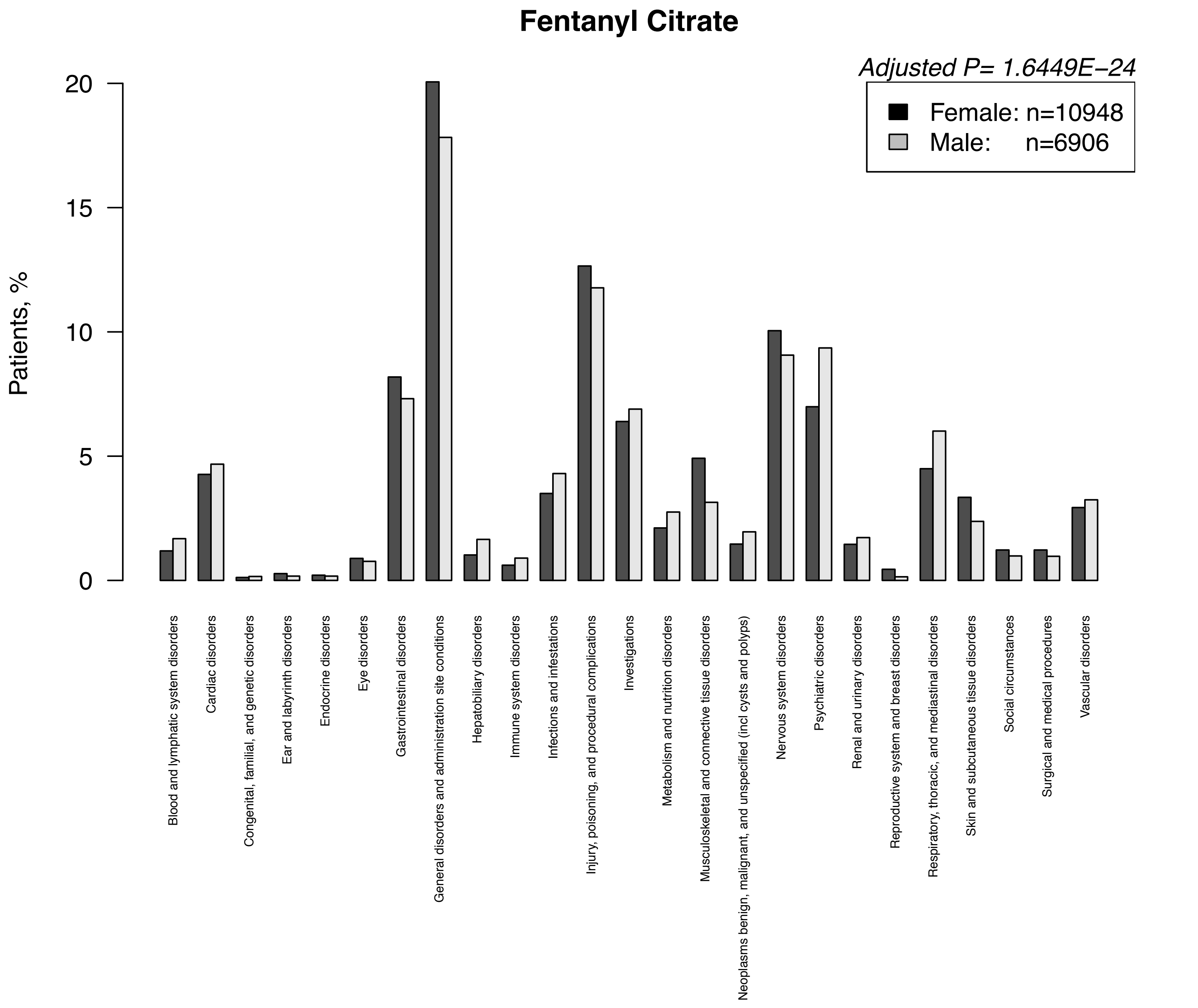

Fentanyl Citrate
Adjusted P= 1.6449E−24
Female: n=10948
Male: n=6906
Patients, %
0
5
10
15
20
Blood and lymphatic system disorders
Cardiac disorders
Congenital, familial, and genetic disorders
Ear and labyrinth disorders
Endocrine disorders
Eye disorders
Gastrointestinal disorders
General disorders and administration site conditions
Hepatobiliary disorders
Immune system disorders
Infections and infestations
Injury, poisoning, and procedural complications
Investigations
Metabolism and nutrition disorders
Musculoskeletal and connective tissue disorders
Neoplasms benign, malignant, and unspecified (incl cysts and polyps)
Nervous system disorders
Psychiatric disorders
Renal and urinary disorders
Reproductive system and breast disorders
Respiratory, thoracic, and mediastinal disorders
Skin and subcutaneous tissue disorders
Social circumstances
Surgical and medical procedures
Vascular disorders

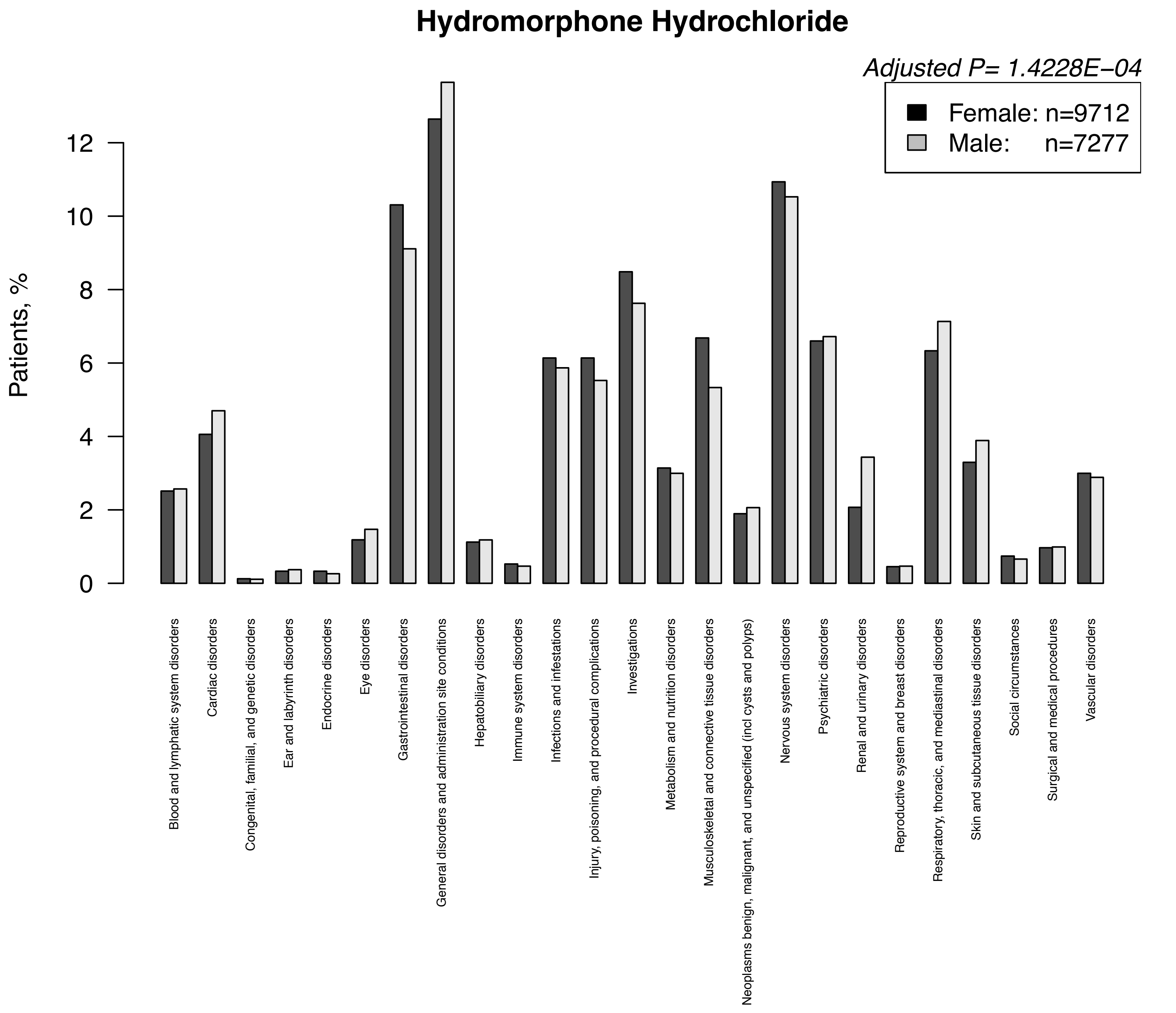

Hydromorphone Hydrochloride
Adjusted P= 1.4228E−04
Female: n=9712
Male: n=7277
Patients, %
0
2
4
6
8
10
12
Blood and lymphatic system disorders
Cardiac disorders
Congenital, familial, and genetic disorders
Ear and labyrinth disorders
Endocrine disorders
Eye disorders
Gastrointestinal disorders
General disorders and administration site conditions
Hepatobiliary disorders
Immune system disorders
Infections and infestations
Injury, poisoning, and procedural complications
Investigations
Metabolism and nutrition disorders
Musculoskeletal and connective tissue disorders
Neoplasms benign, malignant, and unspecified (incl cysts and polyps)
Nervous system disorders
Psychiatric disorders
Renal and urinary disorders
Reproductive system and breast disorders
Respiratory, thoracic, and mediastinal disorders
Skin and subcutaneous tissue disorders
Social circumstances
Surgical and medical procedures
Vascular disorders

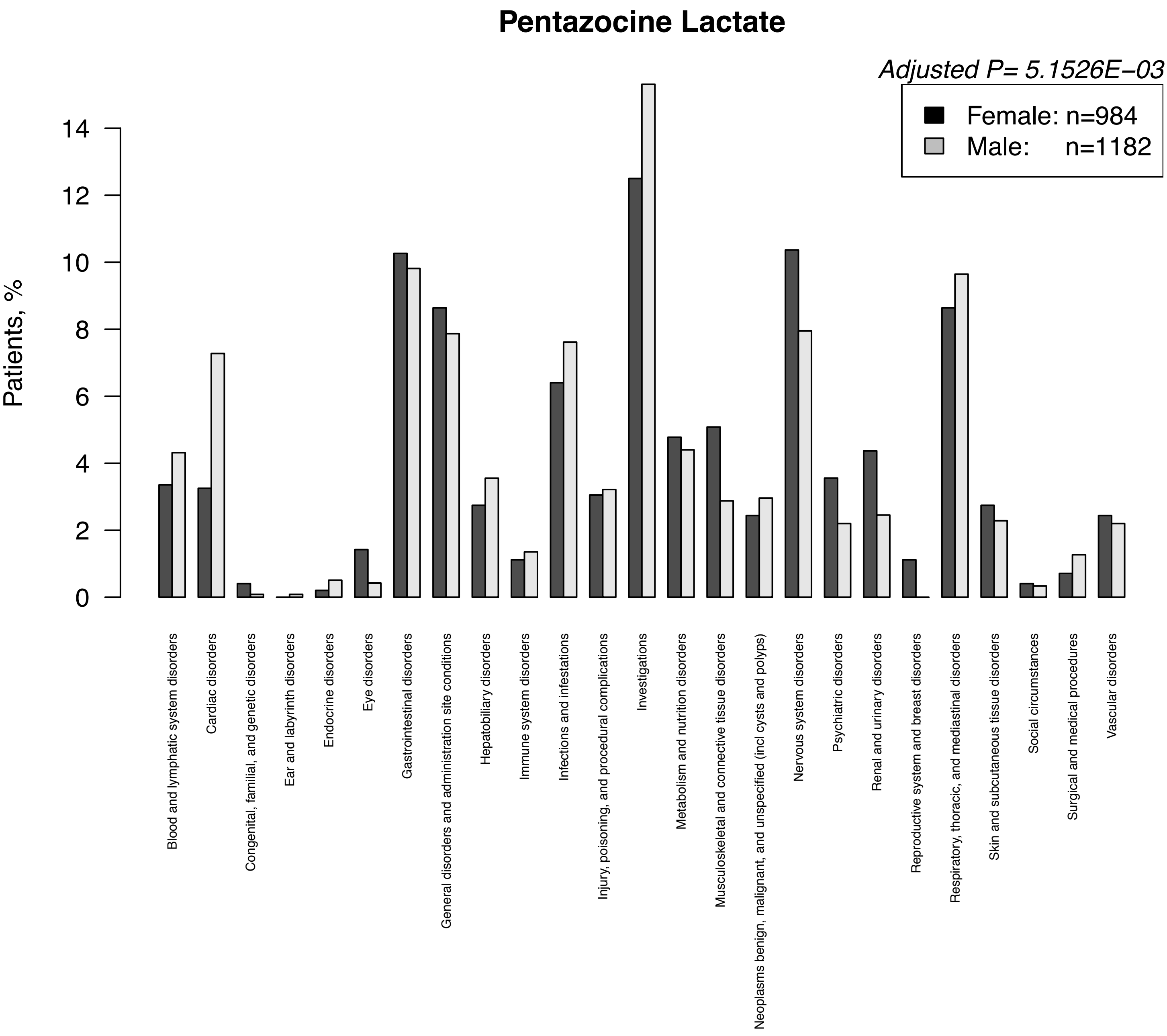
Pentazocine Lactate
Adjusted P= 5.1526E−03
Female: n=984
Male: n=1182
Patients, %
0
2
4
6
8
10
12
14
Blood and lymphatic system disorders
Cardiac disorders
Congenital, familial, and genetic disorders
Ear and labyrinth disorders
Endocrine disorders
Eye disorders
Gastrointestinal disorders
General disorders and administration site conditions
Hepatobiliary disorders
Immune system disorders
Infections and infestations
Injury, poisoning, and procedural complications
Investigations
Metabolism and nutrition disorders
Musculoskeletal and connective tissue disorders
Neoplasms benign, malignant, and unspecified (incl cysts and polyps)
Nervous system disorders
Psychiatric disorders
Renal and urinary disorders
Reproductive system and breast disorders
Respiratory, thoracic, and mediastinal disorders
Skin and subcutaneous tissue disorders
Social circumstances
Surgical and medical procedures
Vascular disorders

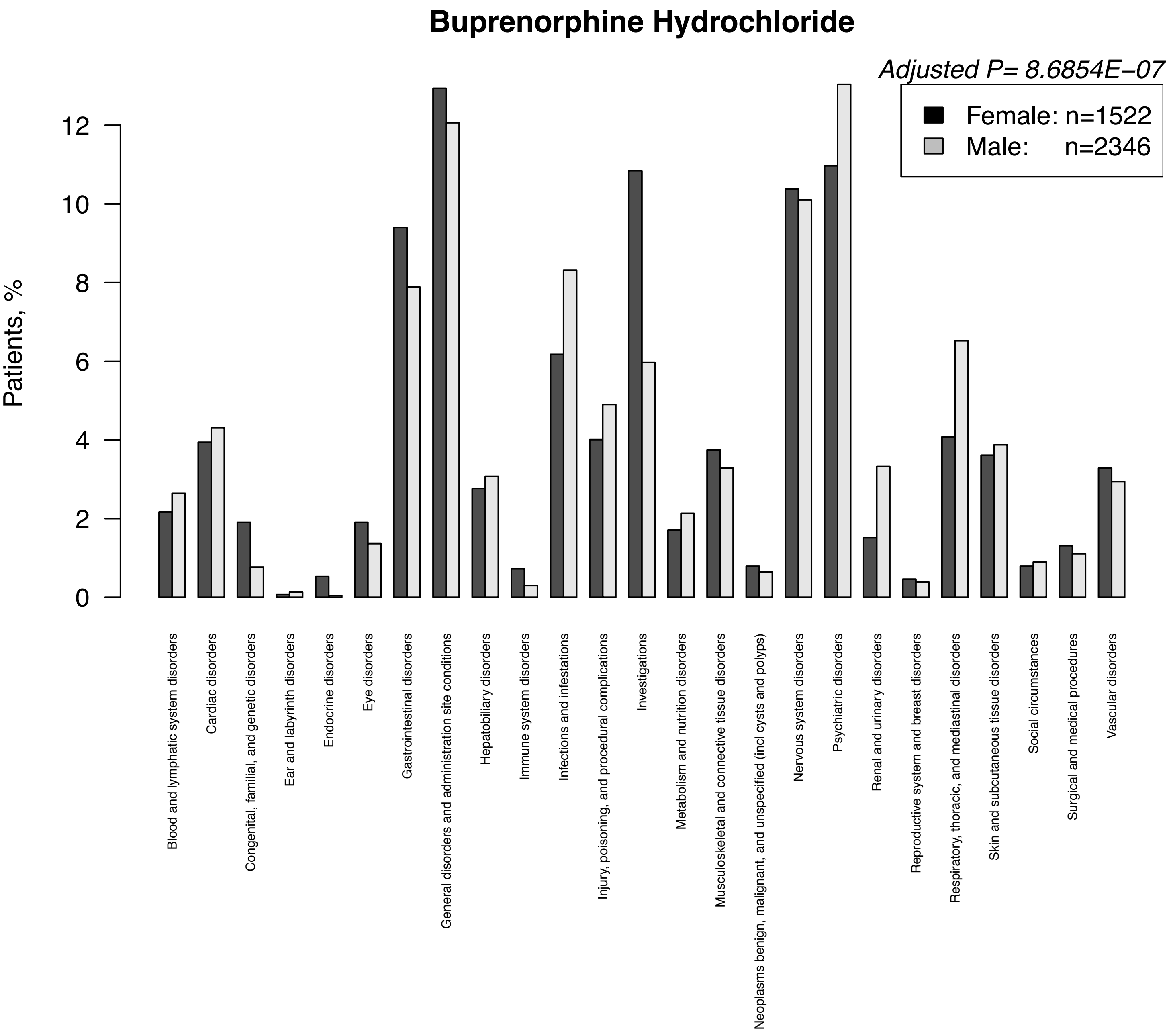

Buprenorphine Hydrochloride
Adjusted P= 8.6854E−07
Female: n=1522
Male: n=2346
Patients, %
0
2
4
6
8
10
12
Blood and lymphatic system disorders
Cardiac disorders
Congenital, familial, and genetic disorders
Ear and labyrinth disorders
Endocrine disorders
Eye disorders
Gastrointestinal disorders
General disorders and administration site conditions
Hepatobiliary disorders
Immune system disorders
Infections and infestations
Injury, poisoning, and procedural complications
Investigations
Metabolism and nutrition disorders
Musculoskeletal and connective tissue disorders
Neoplasms benign, malignant, and unspecified (incl cysts and polyps)
Nervous system disorders
Psychiatric disorders
Renal and urinary disorders
Reproductive system and breast disorders
Respiratory, thoracic, and mediastinal disorders
Skin and subcutaneous tissue disorders
Social circumstances
Surgical and medical procedures
Vascular disorders

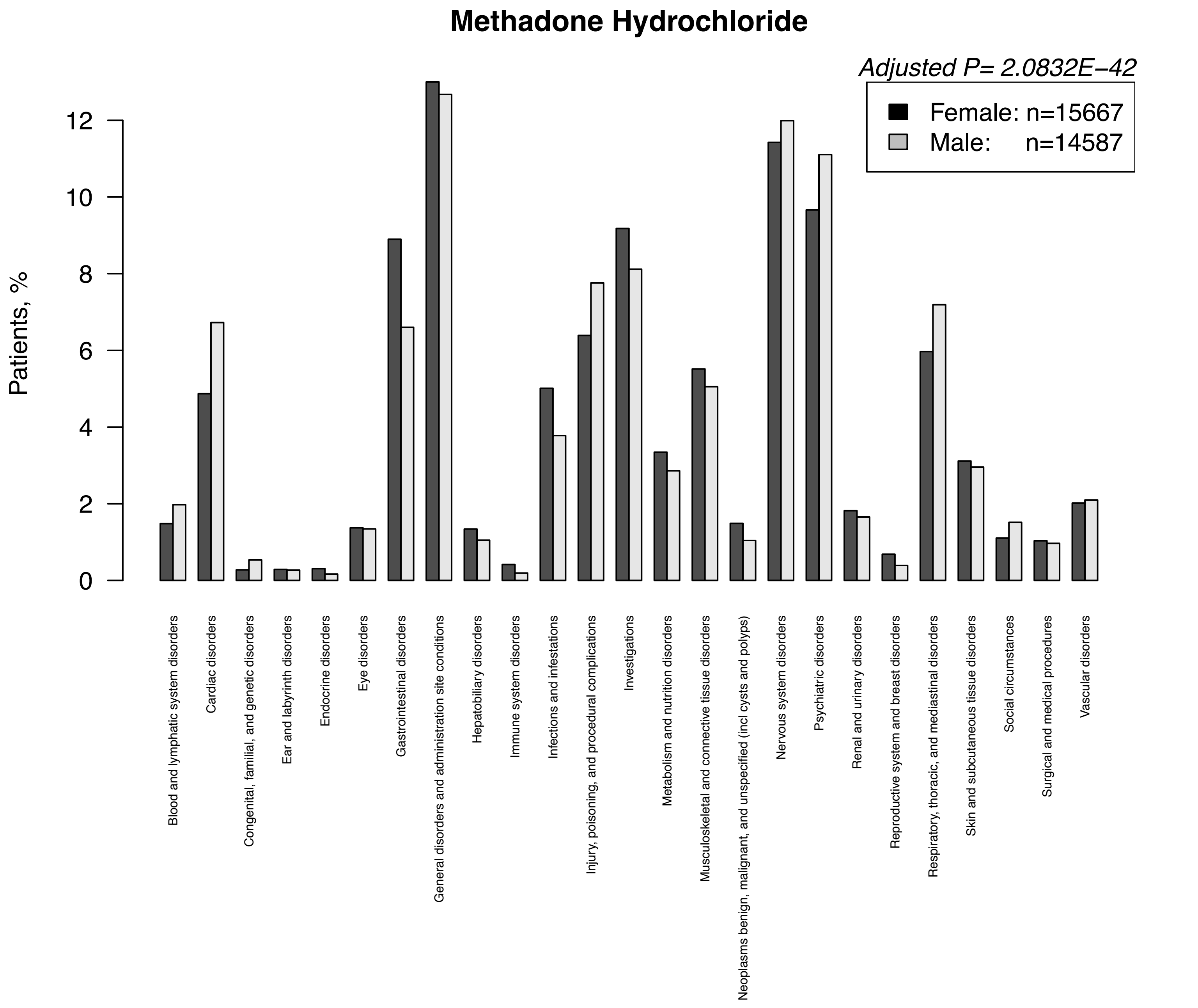
Methadone Hydrochloride
Adjusted P= 2.0832E−42
Female: n=15667
Male: n=14587
Patients, %
0
2
4
6
8
10
12
Blood and lymphatic system disorders
Cardiac disorders
Congenital, familial, and genetic disorders
Ear and labyrinth disorders
Endocrine disorders
Eye disorders
Gastrointestinal disorders
General disorders and administration site conditions
Hepatobiliary disorders
Immune system disorders
Infections and infestations
Injury, poisoning, and procedural complications
Investigations
Metabolism and nutrition disorders
Musculoskeletal and connective tissue disorders
Neoplasms benign, malignant, and unspecified (incl cysts and polyps)
Nervous system disorders
Psychiatric disorders
Renal and urinary disorders
Reproductive system and breast disorders
Respiratory, thoracic, and mediastinal disorders
Skin and subcutaneous tissue disorders
Social circumstances
Surgical and medical procedures
Vascular disorders

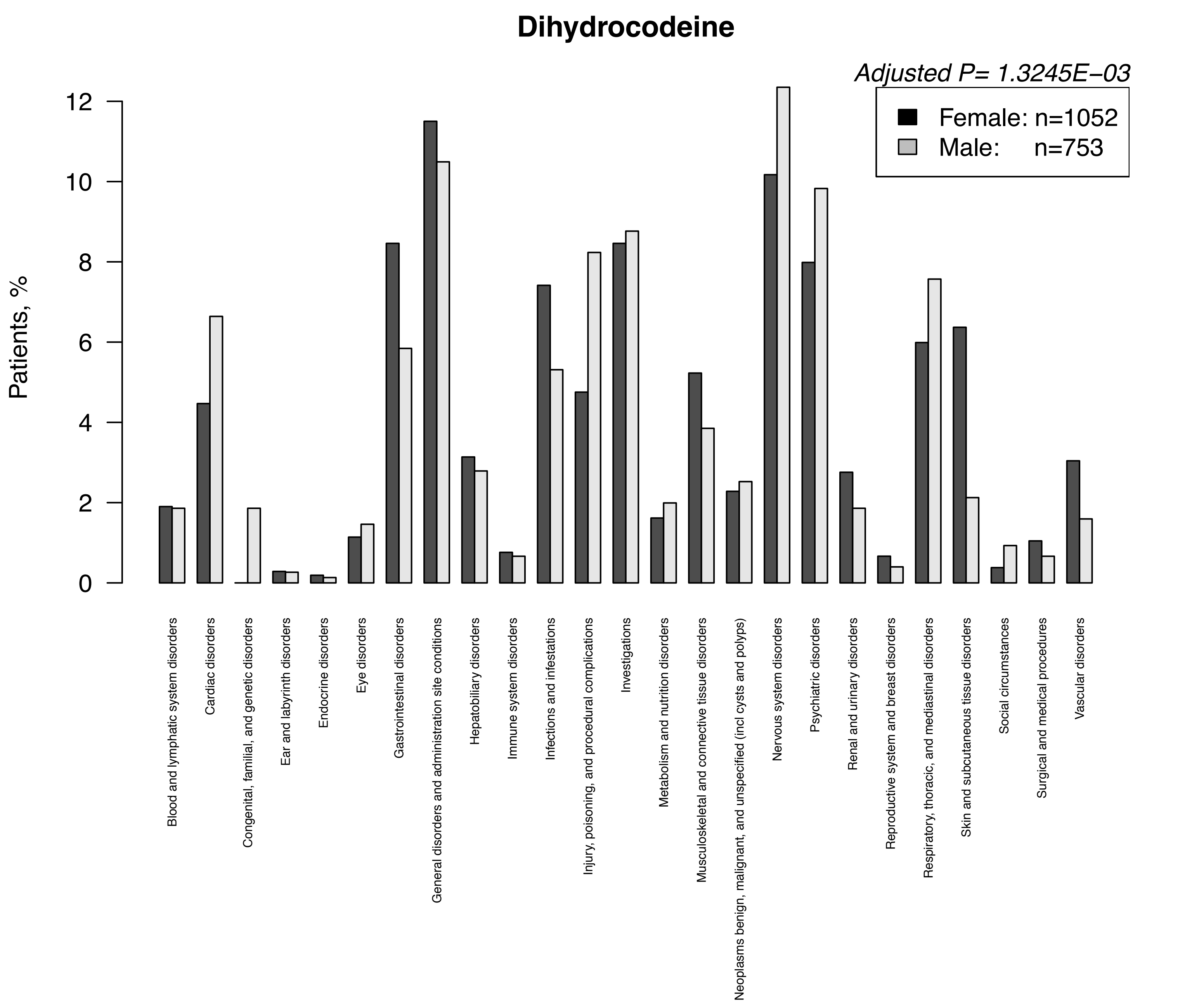
Dihydrocodeine
Adjusted P= 1.3245E−03
Female: n=1052
Male: n=753
Patients, %
0
2
4
6
8
10
12
Blood and lymphatic system disorders
Cardiac disorders
Congenital, familial, and genetic disorders
Ear and labyrinth disorders
Endocrine disorders
Eye disorders
Gastrointestinal disorders
General disorders and administration site conditions
Hepatobiliary disorders
Immune system disorders
Infections and infestations
Injury, poisoning, and procedural complications
Investigations
Metabolism and nutrition disorders
Musculoskeletal and connective tissue disorders
Neoplasms benign, malignant, and unspecified (incl cysts and polyps)
Nervous system disorders
Psychiatric disorders
Renal and urinary disorders
Reproductive system and breast disorders
Respiratory, thoracic, and mediastinal disorders
Skin and subcutaneous tissue disorders
Social circumstances
Surgical and medical procedures
Vascular disorders

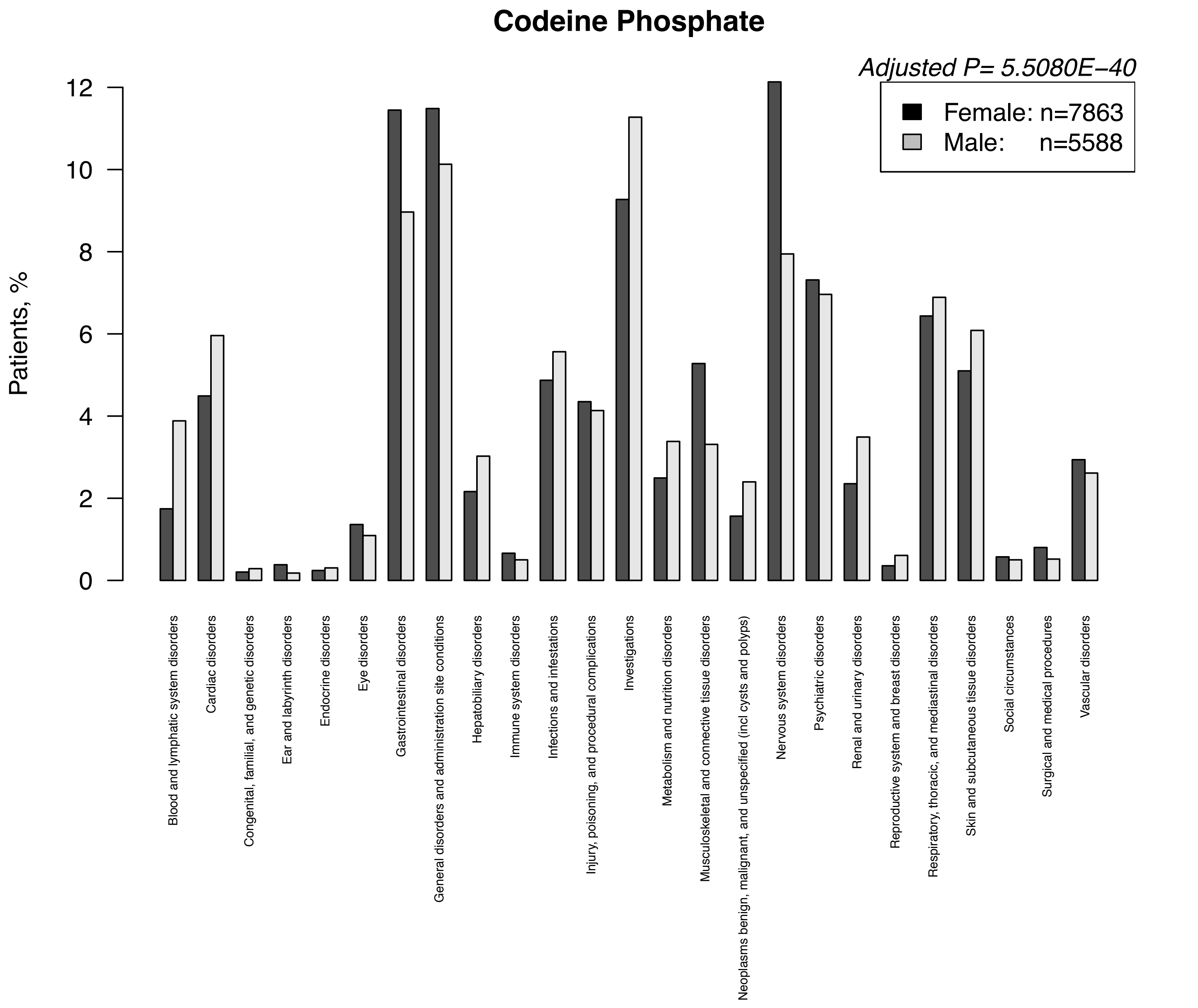
Codeine Phosphate
Adjusted P= 5.5080E−40
Female: n=7863
Male: n=5588
Patients, %
0
2
4
6
8
10
12
Blood and lymphatic system disorders
Cardiac disorders
Congenital, familial, and genetic disorders
Ear and labyrinth disorders
Endocrine disorders
Eye disorders
Gastrointestinal disorders
General disorders and administration site conditions
Hepatobiliary disorders
Immune system disorders
Infections and infestations
Injury, poisoning, and procedural complications
Investigations
Metabolism and nutrition disorders
Musculoskeletal and connective tissue disorders
Neoplasms benign, malignant, and unspecified (incl cysts and polyps)
Nervous system disorders
Psychiatric disorders
Renal and urinary disorders
Reproductive system and breast disorders
Respiratory, thoracic, and mediastinal disorders
Skin and subcutaneous tissue disorders
Social circumstances
Surgical and medical procedures
Vascular disorders

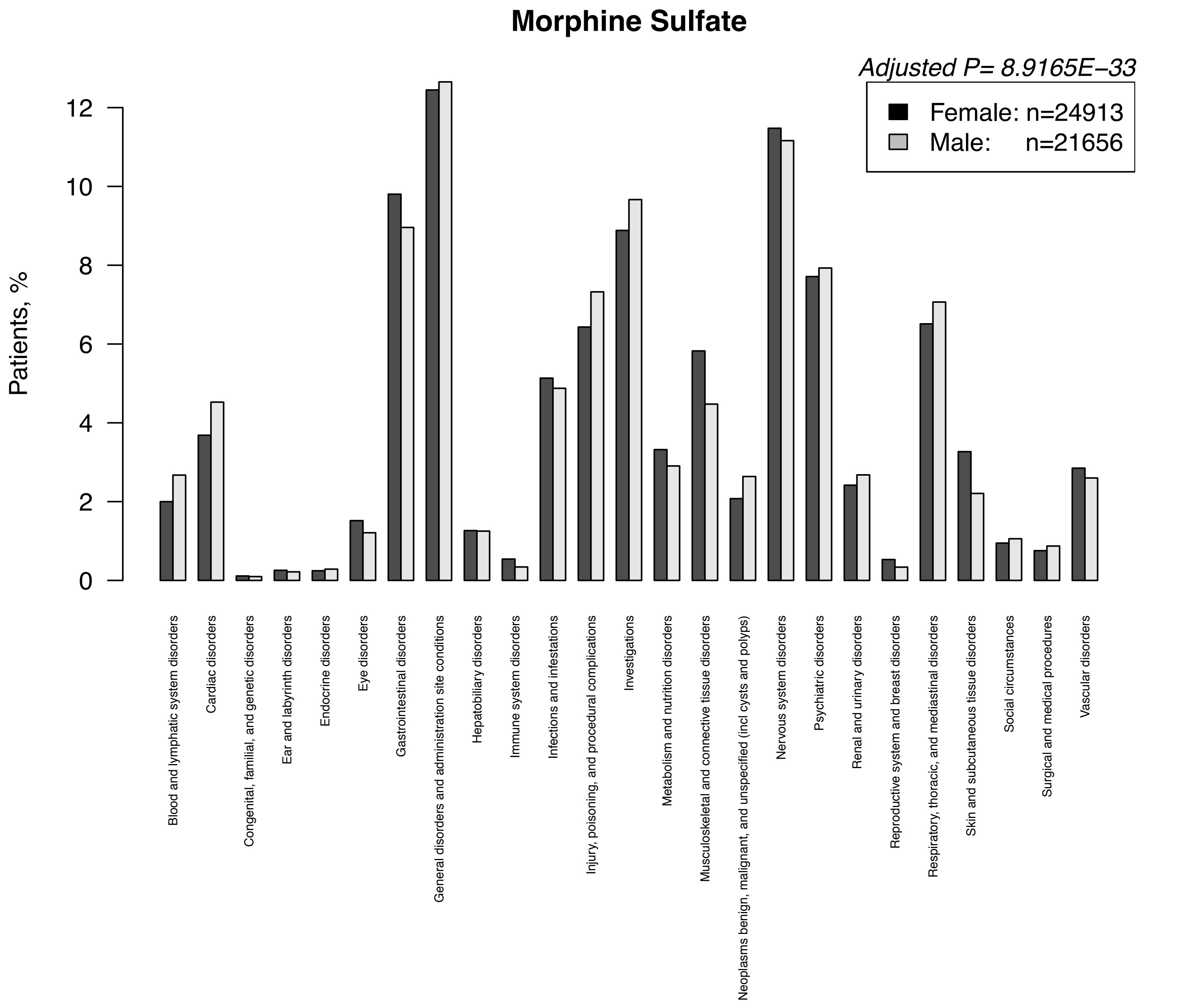
Morphine Sulfate
Adjusted P= 8.9165E−33
Female: n=24913
Male: n=21656
Patients, %
0
2
4
6
8
10
12
Blood and lymphatic system disorders
Cardiac disorders
Congenital, familial, and genetic disorders
Ear and labyrinth disorders
Endocrine disorders
Eye disorders
Gastrointestinal disorders
General disorders and administration site conditions
Hepatobiliary disorders
Immune system disorders
Infections and infestations
Injury, poisoning, and procedural complications
Investigations
Metabolism and nutrition disorders
Musculoskeletal and connective tissue disorders
Neoplasms benign, malignant, and unspecified (incl cysts and polyps)
Nervous system disorders
Psychiatric disorders
Renal and urinary disorders
Reproductive system and breast disorders
Respiratory, thoracic, and mediastinal disorders
Skin and subcutaneous tissue disorders
Social circumstances
Surgical and medical procedures
Vascular disorders

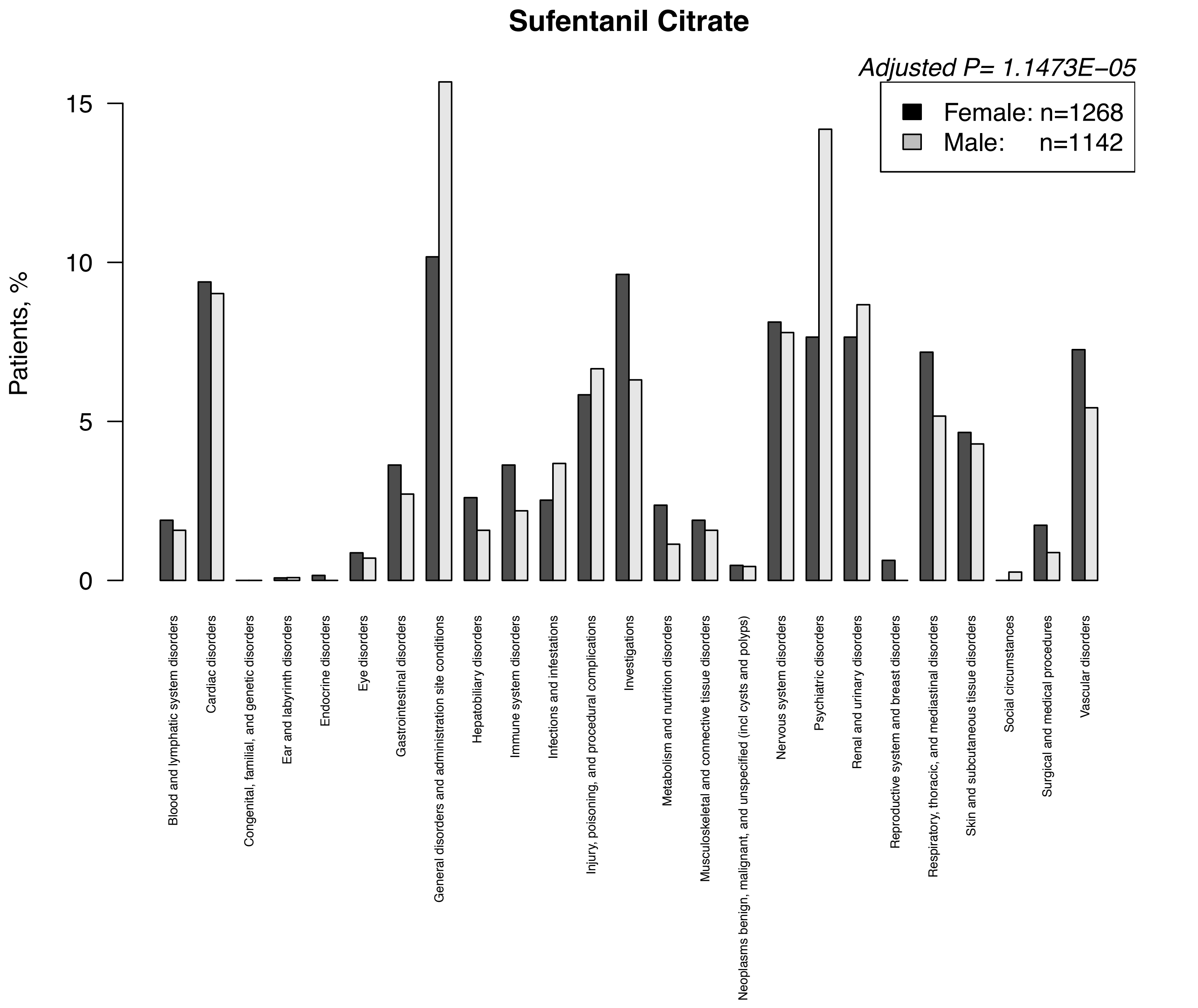

Sufentanil Citrate
Adjusted P= 1.1473E−05
Female: n=1268
Male: n=1142
Patients, %
0
5
10
15
Blood and lymphatic system disorders
Cardiac disorders
Congenital, familial, and genetic disorders
Ear and labyrinth disorders
Endocrine disorders
Eye disorders
Gastrointestinal disorders
General disorders and administration site conditions
Hepatobiliary disorders
Immune system disorders
Infections and infestations
Injury, poisoning, and procedural complications
Investigations
Metabolism and nutrition disorders
Musculoskeletal and connective tissue disorders
Neoplasms benign, malignant, and unspecified (incl cysts and polyps)
Nervous system disorders
Psychiatric disorders
Renal and urinary disorders
Reproductive system and breast disorders
Respiratory, thoracic, and mediastinal disorders
Skin and subcutaneous tissue disorders
Social circumstances
Surgical and medical procedures
Vascular disorders

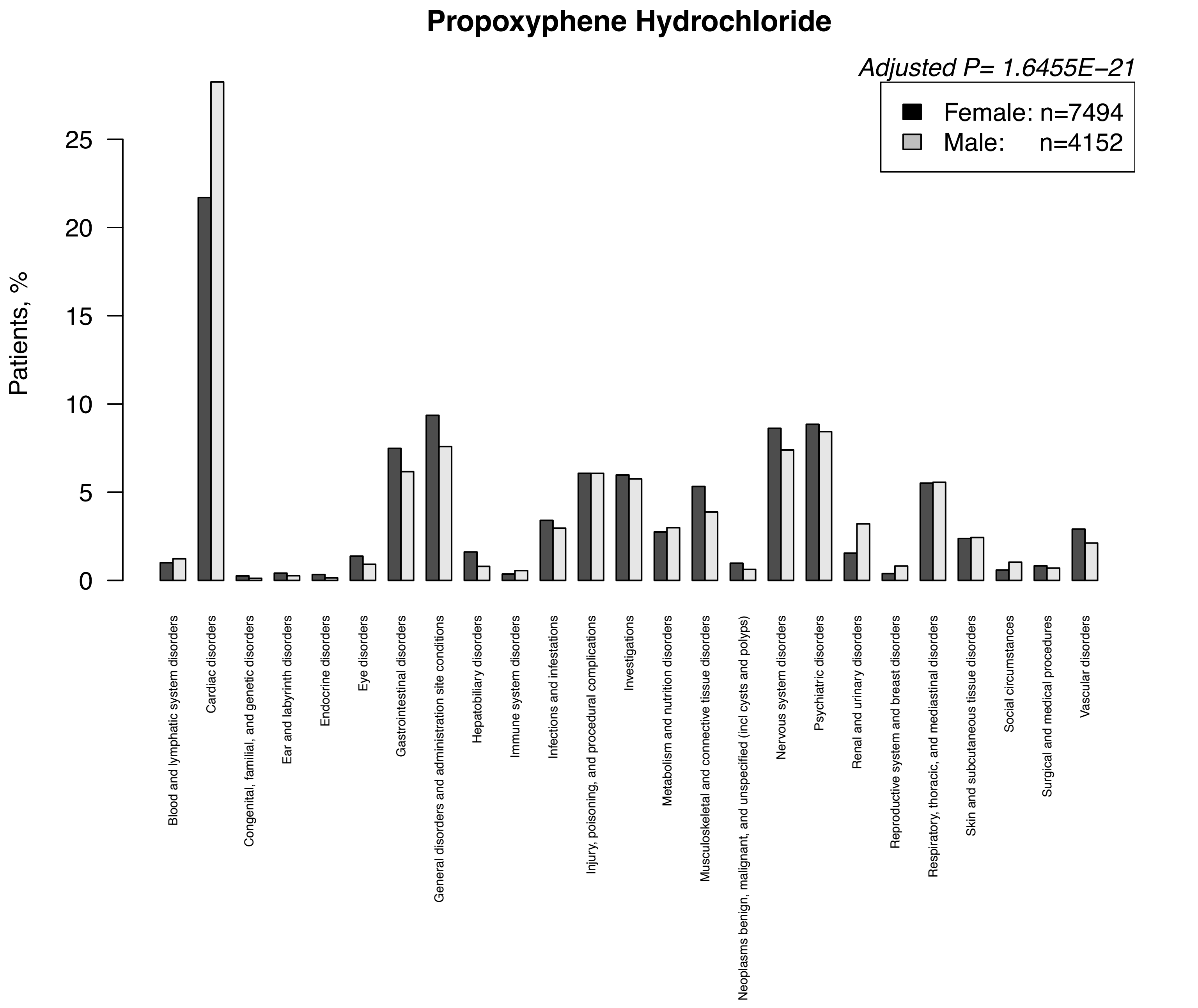
Propoxyphene Hydrochloride
Adjusted P= 1.6455E−21
Female: n=7494
Male: n=4152
Patients, %
0
5
10
15
20
25
Blood and lymphatic system disorders
Cardiac disorders
Congenital, familial, and genetic disorders
Ear and labyrinth disorders
Endocrine disorders
Eye disorders
Gastrointestinal disorders
General disorders and administration site conditions
Hepatobiliary disorders
Immune system disorders
Infections and infestations
Injury, poisoning, and procedural complications
Investigations
Metabolism and nutrition disorders
Musculoskeletal and connective tissue disorders
Neoplasms benign, malignant, and unspecified (incl cysts and polyps)
Nervous system disorders
Psychiatric disorders
Renal and urinary disorders
Reproductive system and breast disorders
Respiratory, thoracic, and mediastinal disorders
Skin and subcutaneous tissue disorders
Social circumstances
Surgical and medical procedures
Vascular disorders

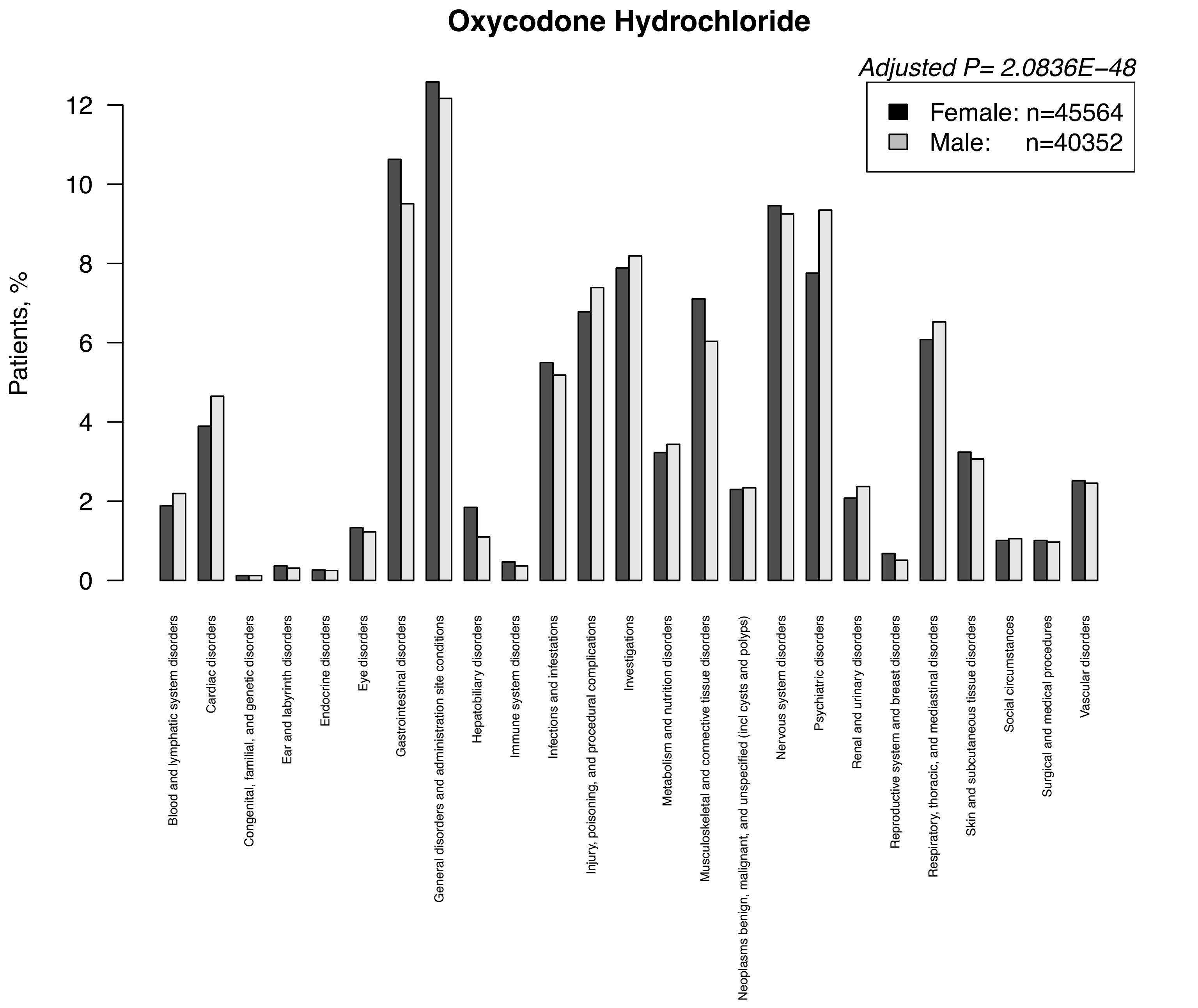

Oxycodone Hydrochloride
Adjusted P= 2.0836E−48
Female: n=45564
Male: n=40352
Patients, %
0
2
4
6
8
10
12
Blood and lymphatic system disorders
Cardiac disorders
Congenital, familial, and genetic disorders
Ear and labyrinth disorders
Endocrine disorders
Eye disorders
Gastrointestinal disorders
General disorders and administration site conditions
Hepatobiliary disorders
Immune system disorders
Infections and infestations
Injury, poisoning, and procedural complications
Investigations
Metabolism and nutrition disorders
Musculoskeletal and connective tissue disorders
Neoplasms benign, malignant, and unspecified (incl cysts and polyps)
Nervous system disorders
Psychiatric disorders
Renal and urinary disorders
Reproductive system and breast disorders
Respiratory, thoracic, and mediastinal disorders
Skin and subcutaneous tissue disorders
Social circumstances
Surgical and medical procedures
Vascular disorders

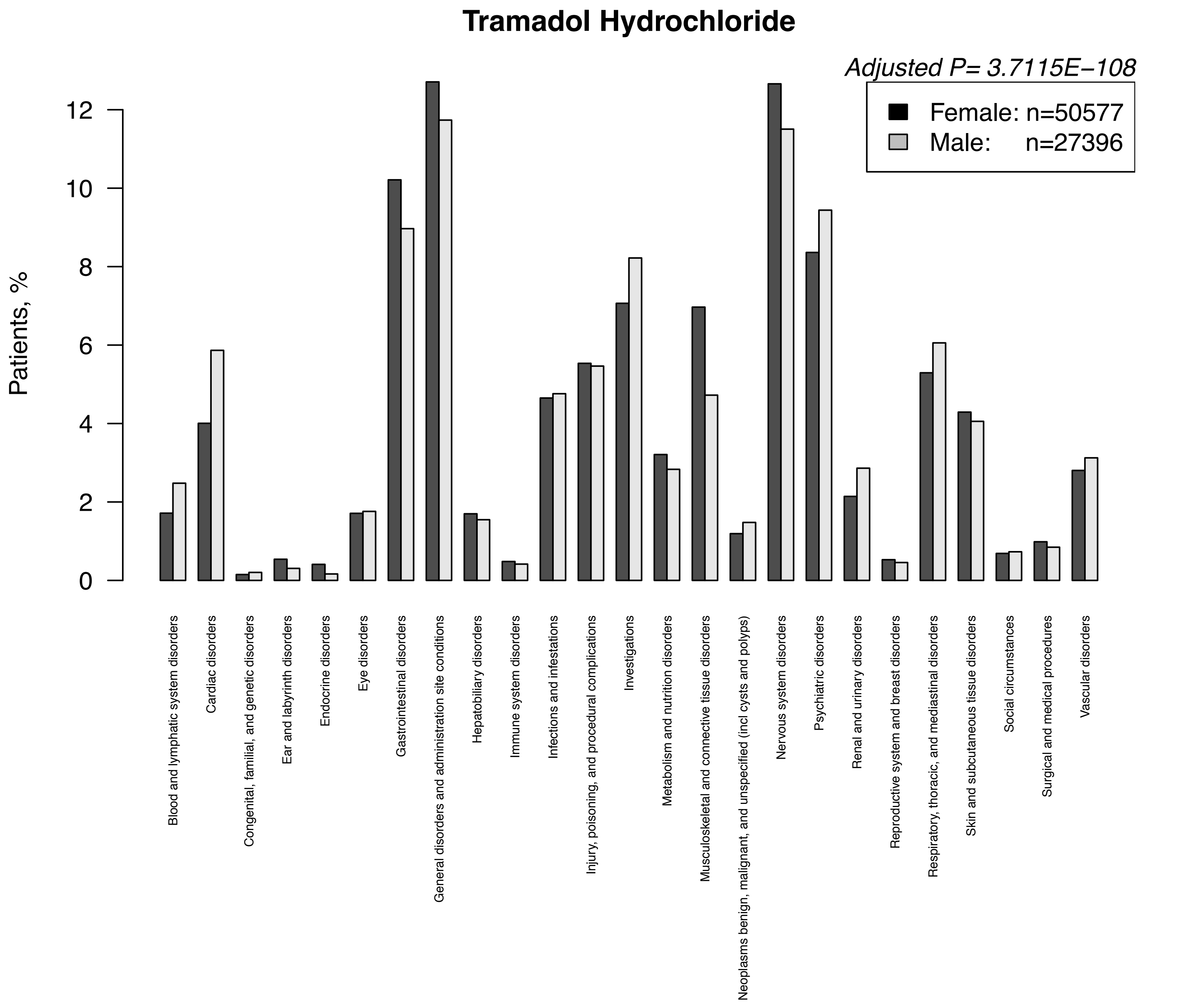
Tramadol Hydrochloride
Adjusted P= 3.7115E−108
Female: n=50577
Male: n=27396
Patients, %
0
2
4
6
8
10
12
Blood and lymphatic system disorders
Cardiac disorders
Congenital, familial, and genetic disorders
Ear and labyrinth disorders
Endocrine disorders
Eye disorders
Gastrointestinal disorders
General disorders and administration site conditions
Hepatobiliary disorders
Immune system disorders
Infections and infestations
Injury, poisoning, and procedural complications
Investigations
Metabolism and nutrition disorders
Musculoskeletal and connective tissue disorders
Neoplasms benign, malignant, and unspecified (incl cysts and polyps)
Nervous system disorders
Psychiatric disorders
Renal and urinary disorders
Reproductive system and breast disorders
Respiratory, thoracic, and mediastinal disorders
Skin and subcutaneous tissue disorders
Social circumstances
Surgical and medical procedures
Vascular disorders

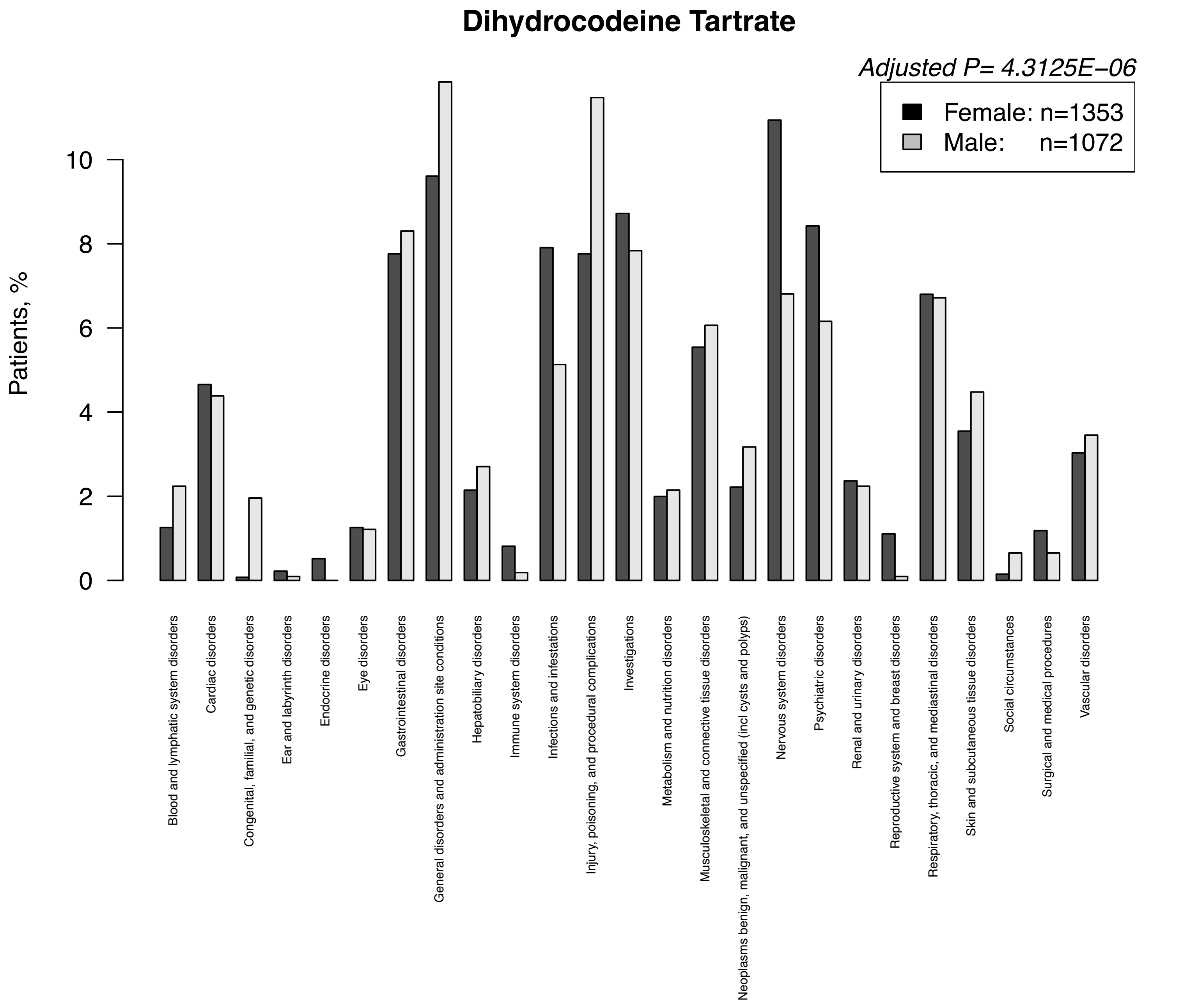

Dihydrocodeine Tartrate
Adjusted P= 4.3125E−06
Female: n=1353
Male: n=1072
Patients, %
0
2
4
6
8
10
Blood and lymphatic system disorders
Cardiac disorders
Congenital, familial, and genetic disorders
Ear and labyrinth disorders
Endocrine disorders
Eye disorders
Gastrointestinal disorders
General disorders and administration site conditions
Hepatobiliary disorders
Immune system disorders
Infections and infestations
Injury, poisoning, and procedural complications
Investigations
Metabolism and nutrition disorders
Musculoskeletal and connective tissue disorders
Neoplasms benign, malignant, and unspecified (incl cysts and polyps)
Nervous system disorders
Psychiatric disorders
Renal and urinary disorders
Reproductive system and breast disorders
Respiratory, thoracic, and mediastinal disorders
Skin and subcutaneous tissue disorders
Social circumstances
Surgical and medical procedures
Vascular disorders

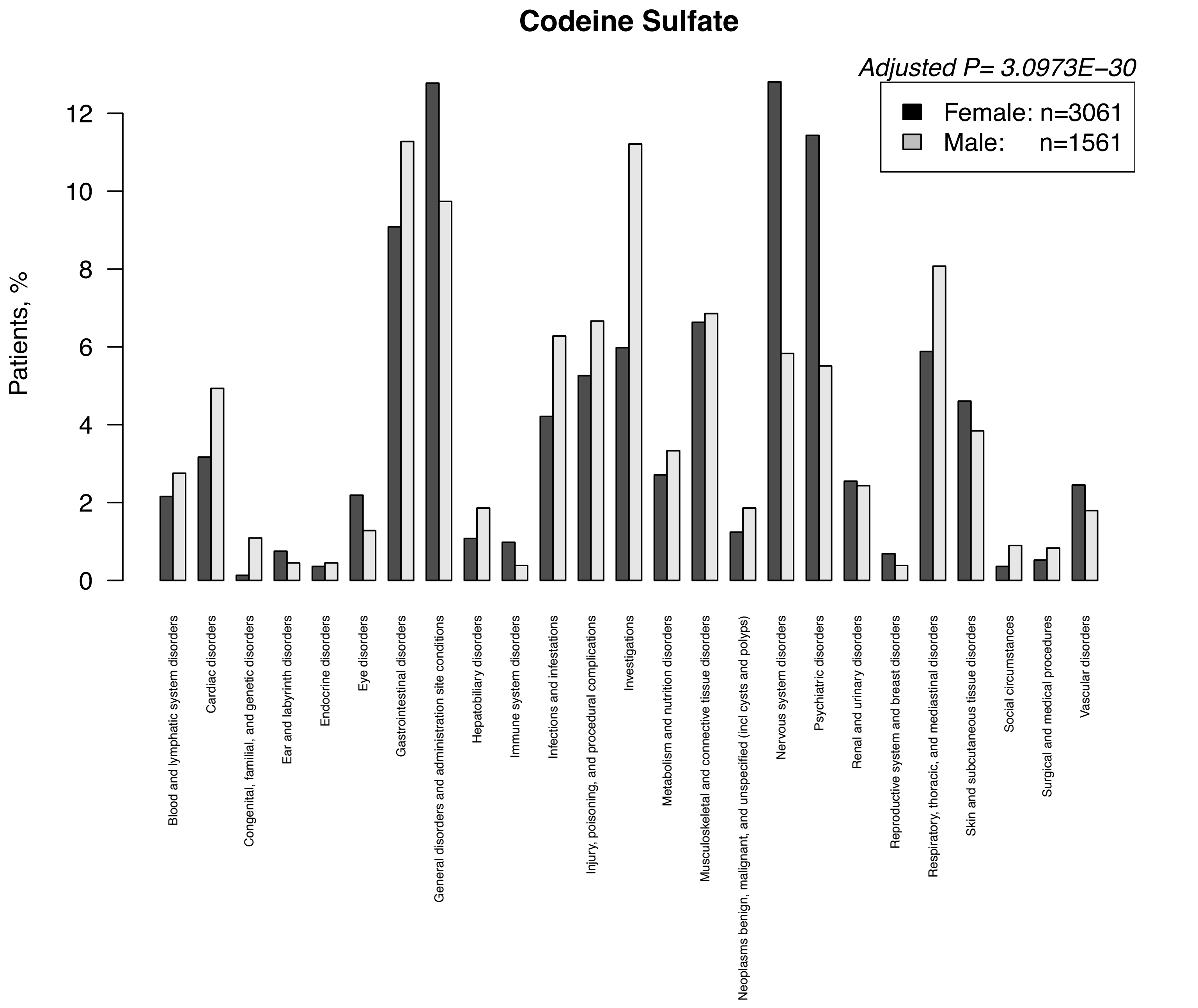
Codeine Sulfate
Adjusted P= 3.0973E−30
Female: n=3061
Male: n=1561
Patients, %
0
2
4
6
8
10
12
Blood and lymphatic system disorders
Cardiac disorders
Congenital, familial, and genetic disorders
Ear and labyrinth disorders
Endocrine disorders
Eye disorders
Gastrointestinal disorders
General disorders and administration site conditions
Hepatobiliary disorders
Immune system disorders
Infections and infestations
Injury, poisoning, and procedural complications
Investigations
Metabolism and nutrition disorders
Musculoskeletal and connective tissue disorders
Neoplasms benign, malignant, and unspecified (incl cysts and polyps)
Nervous system disorders
Psychiatric disorders
Renal and urinary disorders
Reproductive system and breast disorders
Respiratory, thoracic, and mediastinal disorders
Skin and subcutaneous tissue disorders
Social circumstances
Surgical and medical procedures
Vascular disorders

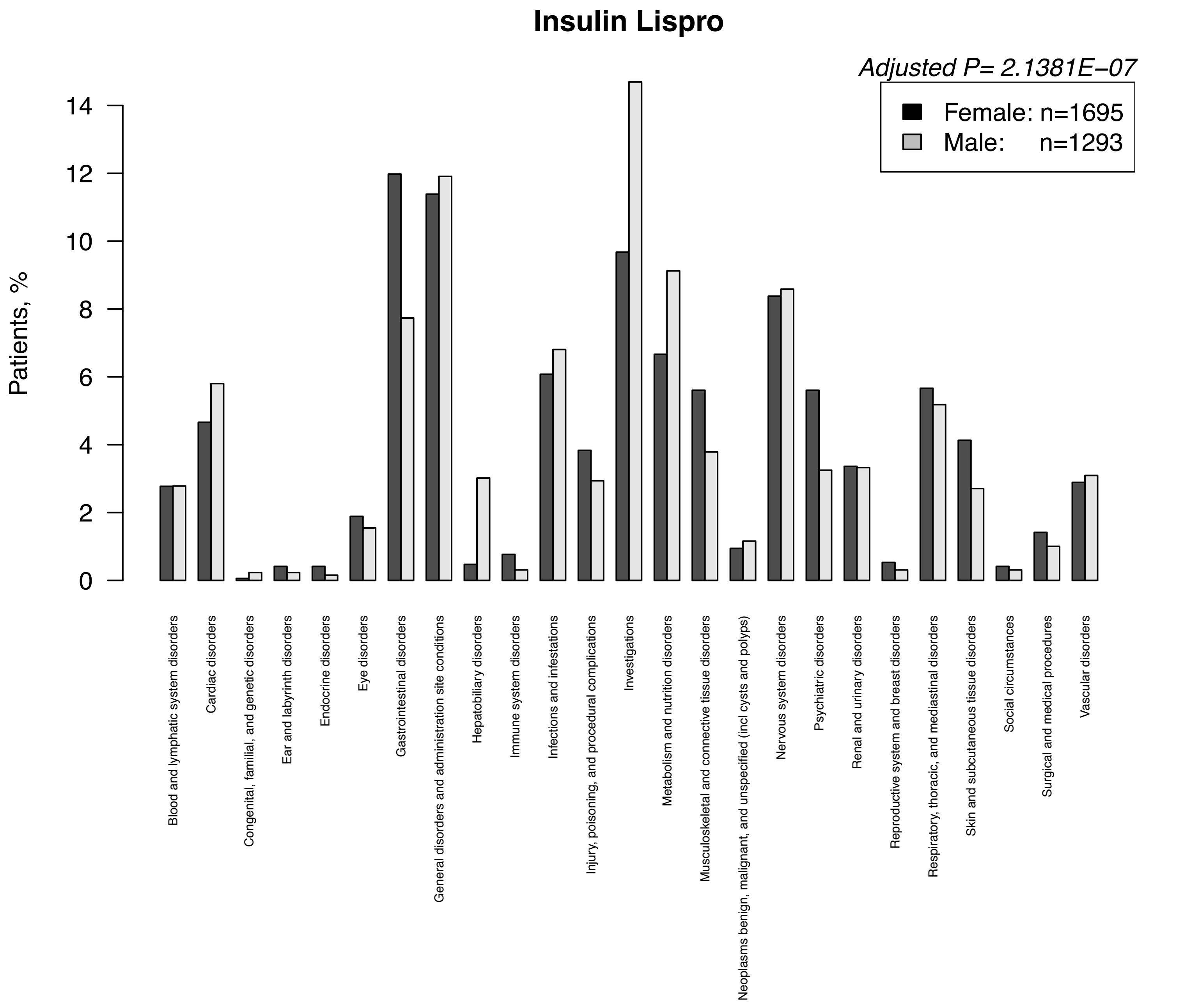

Insulin Lispro
Adjusted P= 2.1381E−07
Female: n=1695
Male: n=1293
Patients, %
0
2
4
6
8
10
12
14
Blood and lymphatic system disorders
Cardiac disorders
Congenital, familial, and genetic disorders
Ear and labyrinth disorders
Endocrine disorders
Eye disorders
Gastrointestinal disorders
General disorders and administration site conditions
Hepatobiliary disorders
Immune system disorders
Infections and infestations
Injury, poisoning, and procedural complications
Investigations
Metabolism and nutrition disorders
Musculoskeletal and connective tissue disorders
Neoplasms benign, malignant, and unspecified (incl cysts and polyps)
Nervous system disorders
Psychiatric disorders
Renal and urinary disorders
Reproductive system and breast disorders
Respiratory, thoracic, and mediastinal disorders
Skin and subcutaneous tissue disorders
Social circumstances
Surgical and medical procedures
Vascular disorders

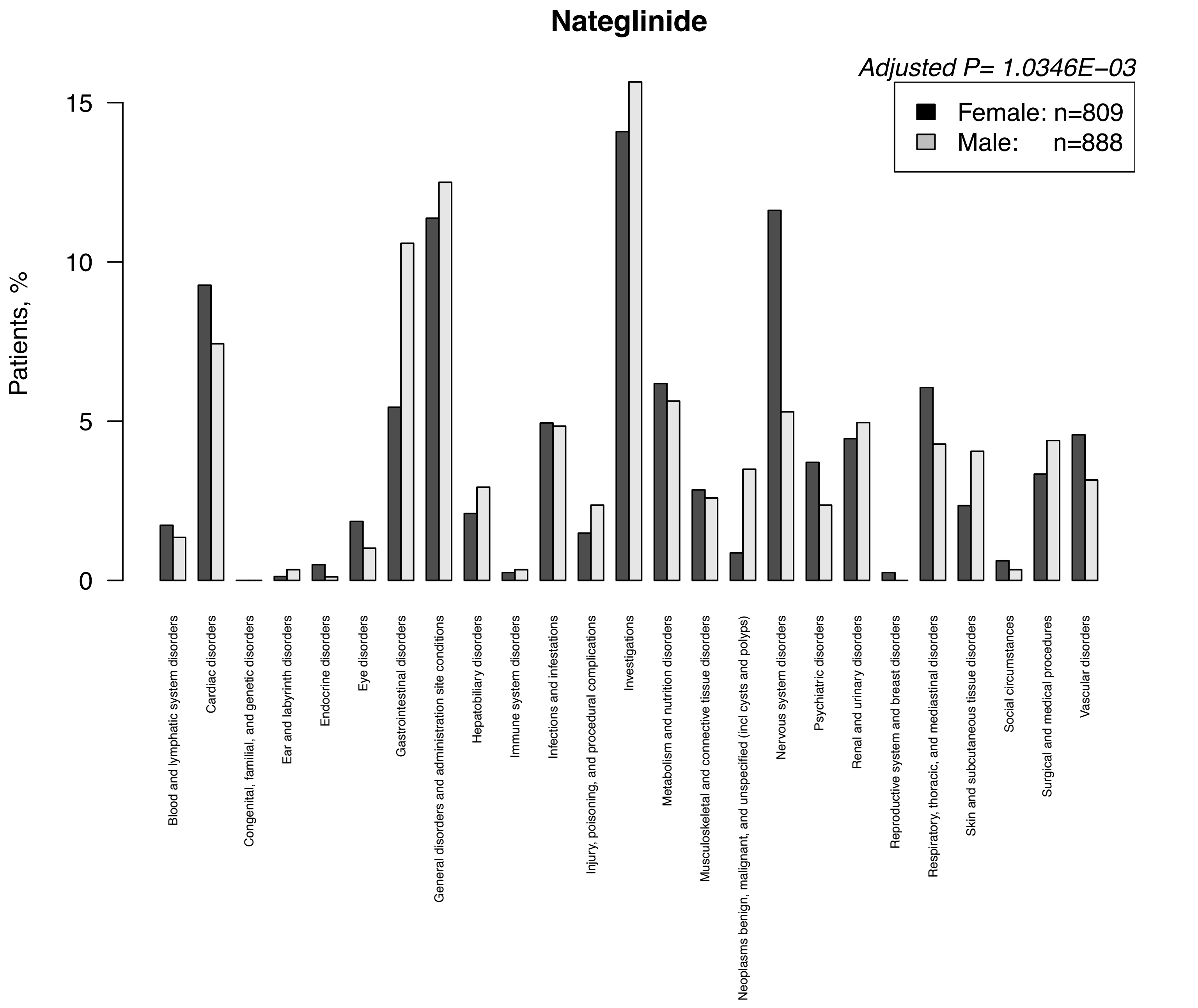
Nateglinide
Adjusted P= 1.0346E−03
Female: n=809
Male: n=888
Patients, %
0
5
10
15
Blood and lymphatic system disorders
Cardiac disorders
Congenital, familial, and genetic disorders
Ear and labyrinth disorders
Endocrine disorders
Eye disorders
Gastrointestinal disorders
General disorders and administration site conditions
Hepatobiliary disorders
Immune system disorders
Infections and infestations
Injury, poisoning, and procedural complications
Investigations
Metabolism and nutrition disorders
Musculoskeletal and connective tissue disorders
Neoplasms benign, malignant, and unspecified (incl cysts and polyps)
Nervous system disorders
Psychiatric disorders
Renal and urinary disorders
Reproductive system and breast disorders
Respiratory, thoracic, and mediastinal disorders
Skin and subcutaneous tissue disorders
Social circumstances
Surgical and medical procedures
Vascular disorders

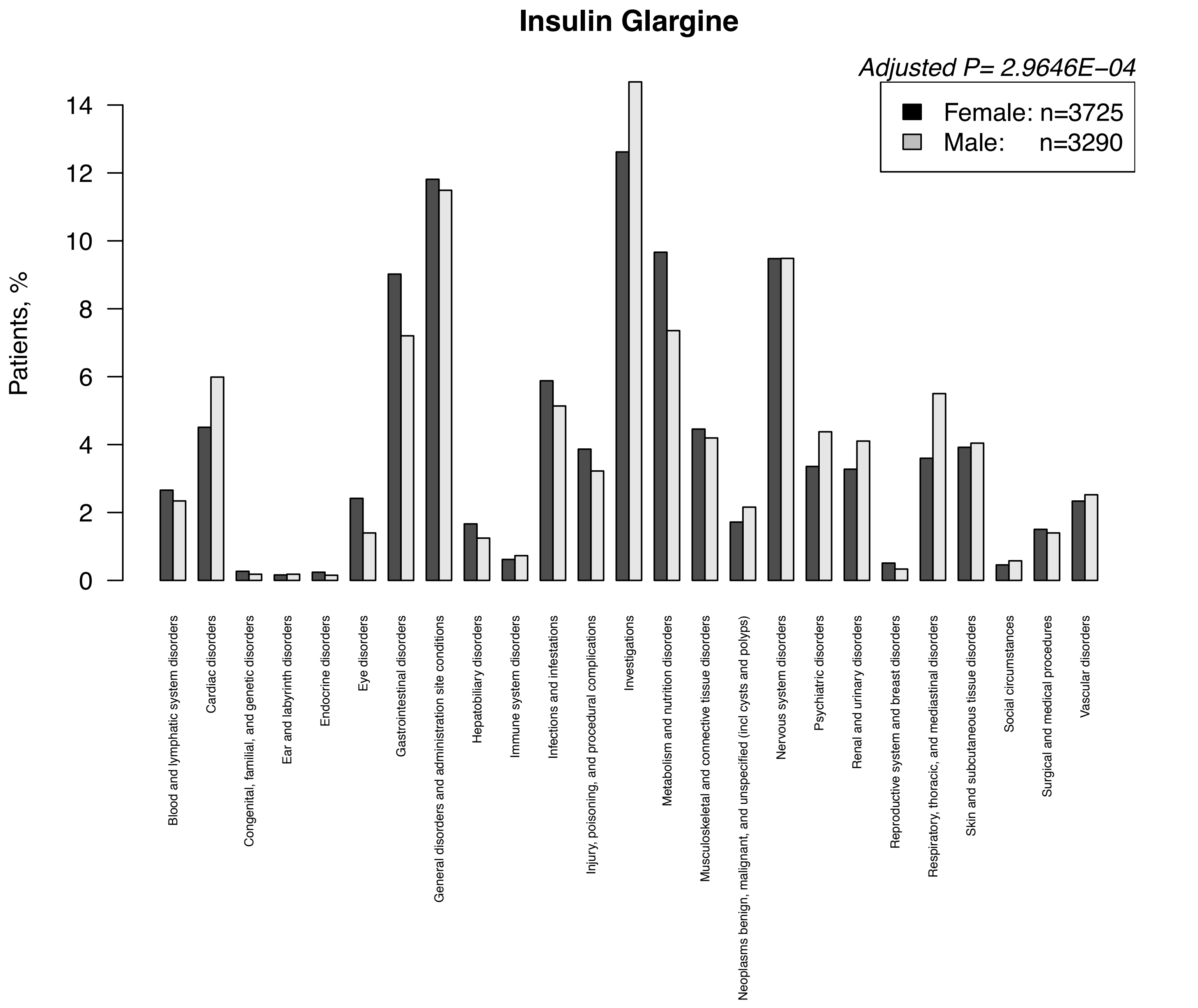

Insulin Glargine
Adjusted P= 2.9646E−04
Female: n=3725
Male: n=3290
Patients, %
0
2
4
6
8
10
12
14
Blood and lymphatic system disorders
Cardiac disorders
Congenital, familial, and genetic disorders
Ear and labyrinth disorders
Endocrine disorders
Eye disorders
Gastrointestinal disorders
General disorders and administration site conditions
Hepatobiliary disorders
Immune system disorders
Infections and infestations
Injury, poisoning, and procedural complications
Investigations
Metabolism and nutrition disorders
Musculoskeletal and connective tissue disorders
Neoplasms benign, malignant, and unspecified (incl cysts and polyps)
Nervous system disorders
Psychiatric disorders
Renal and urinary disorders
Reproductive system and breast disorders
Respiratory, thoracic, and mediastinal disorders
Skin and subcutaneous tissue disorders
Social circumstances
Surgical and medical procedures
Vascular disorders

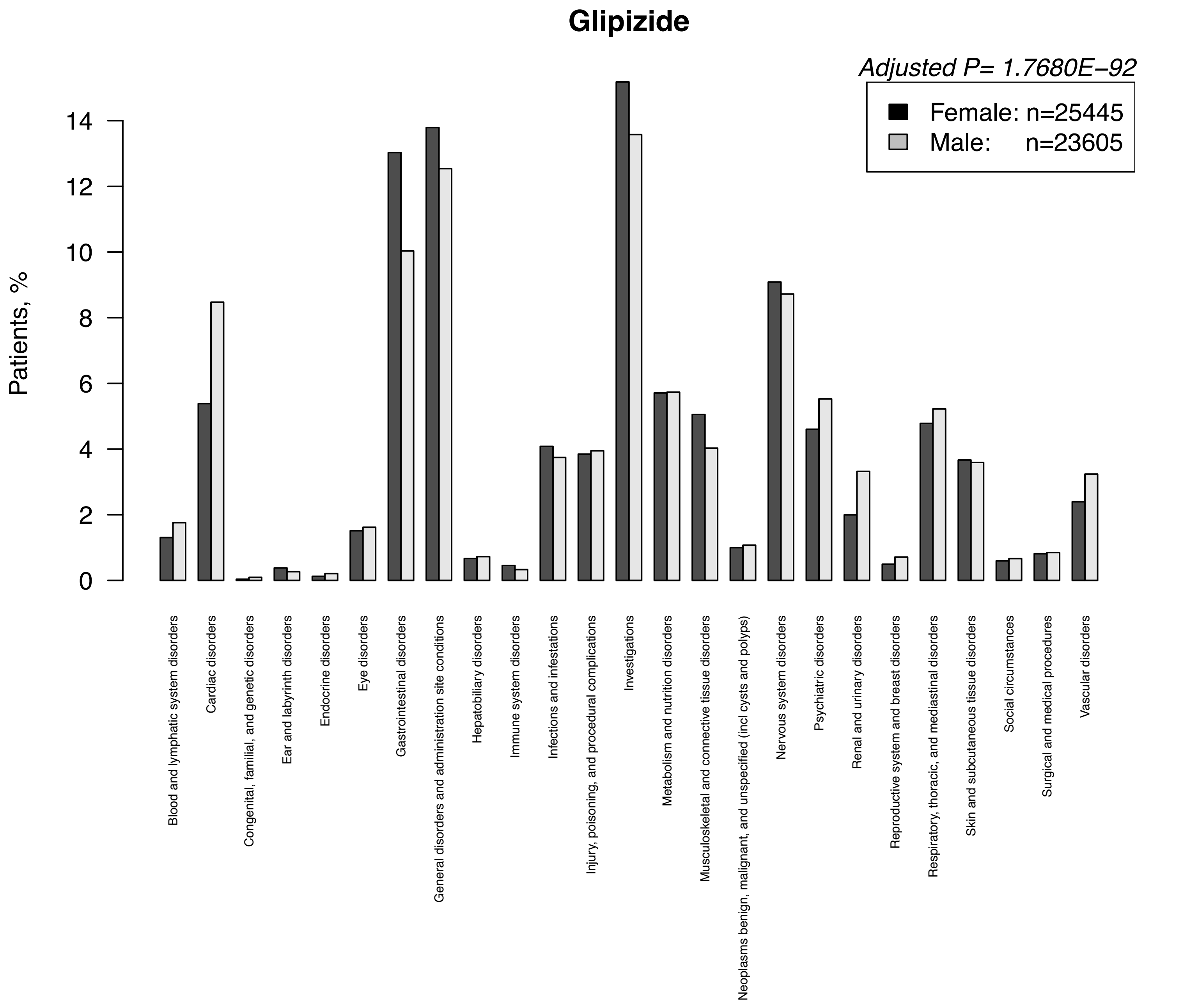
Glipizide
Adjusted P= 1.7680E−92
Female: n=25445
Male: n=23605
Patients, %
0
2
4
6
8
10
12
14
Blood and lymphatic system disorders
Cardiac disorders
Congenital, familial, and genetic disorders
Ear and labyrinth disorders
Endocrine disorders
Eye disorders
Gastrointestinal disorders
General disorders and administration site conditions
Hepatobiliary disorders
Immune system disorders
Infections and infestations
Injury, poisoning, and procedural complications
Investigations
Metabolism and nutrition disorders
Musculoskeletal and connective tissue disorders
Neoplasms benign, malignant, and unspecified (incl cysts and polyps)
Nervous system disorders
Psychiatric disorders
Renal and urinary disorders
Reproductive system and breast disorders
Respiratory, thoracic, and mediastinal disorders
Skin and subcutaneous tissue disorders
Social circumstances
Surgical and medical procedures
Vascular disorders

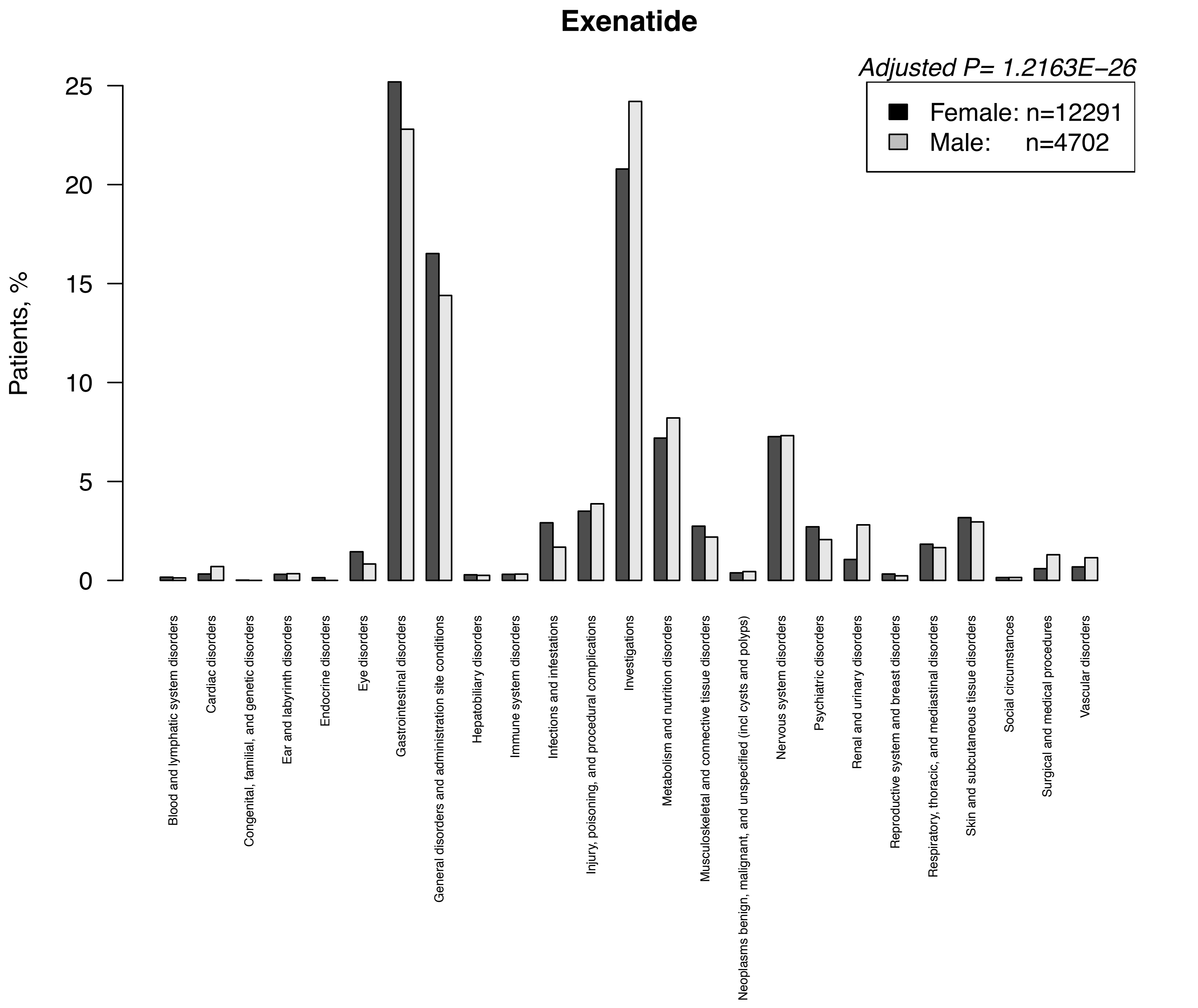
Exenatide
Adjusted P= 1.2163E−26
Female: n=12291
Male: n=4702
Patients, %
0
5
10
15
20
25
Blood and lymphatic system disorders
Cardiac disorders
Congenital, familial, and genetic disorders
Ear and labyrinth disorders
Endocrine disorders
Eye disorders
Gastrointestinal disorders
General disorders and administration site conditions
Hepatobiliary disorders
Immune system disorders
Infections and infestations
Injury, poisoning, and procedural complications
Investigations
Metabolism and nutrition disorders
Musculoskeletal and connective tissue disorders
Neoplasms benign, malignant, and unspecified (incl cysts and polyps)
Nervous system disorders
Psychiatric disorders
Renal and urinary disorders
Reproductive system and breast disorders
Respiratory, thoracic, and mediastinal disorders
Skin and subcutaneous tissue disorders
Social circumstances
Surgical and medical procedures
Vascular disorders

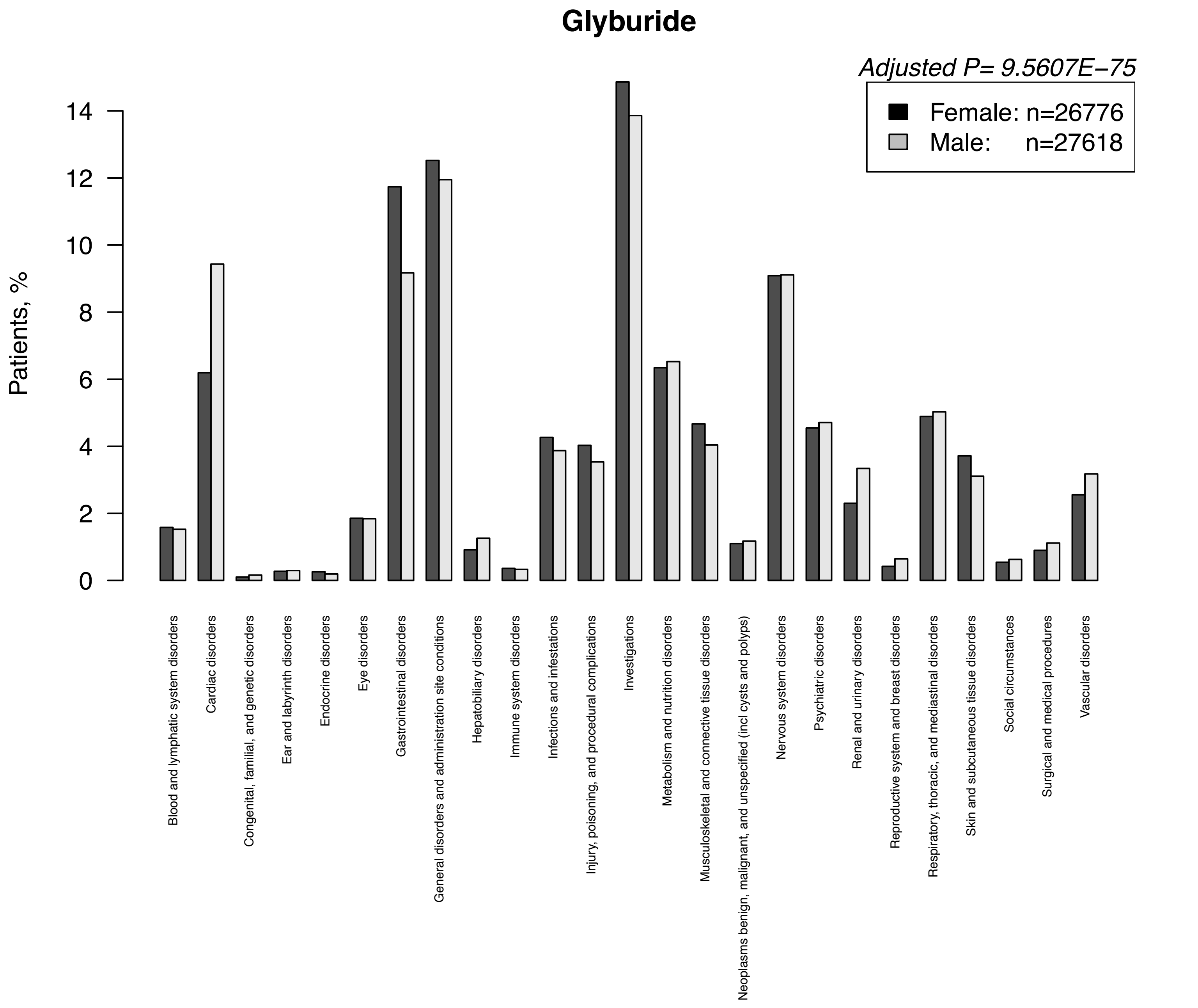
Glyburide
Adjusted P= 9.5607E−75
Female: n=26776
Male: n=27618
Patients, %
0
2
4
6
8
10
12
14
Blood and lymphatic system disorders
Cardiac disorders
Congenital, familial, and genetic disorders
Ear and labyrinth disorders
Endocrine disorders
Eye disorders
Gastrointestinal disorders
General disorders and administration site conditions
Hepatobiliary disorders
Immune system disorders
Infections and infestations
Injury, poisoning, and procedural complications
Investigations
Metabolism and nutrition disorders
Musculoskeletal and connective tissue disorders
Neoplasms benign, malignant, and unspecified (incl cysts and polyps)
Nervous system disorders
Psychiatric disorders
Renal and urinary disorders
Reproductive system and breast disorders
Respiratory, thoracic, and mediastinal disorders
Skin and subcutaneous tissue disorders
Social circumstances
Surgical and medical procedures
Vascular disorders

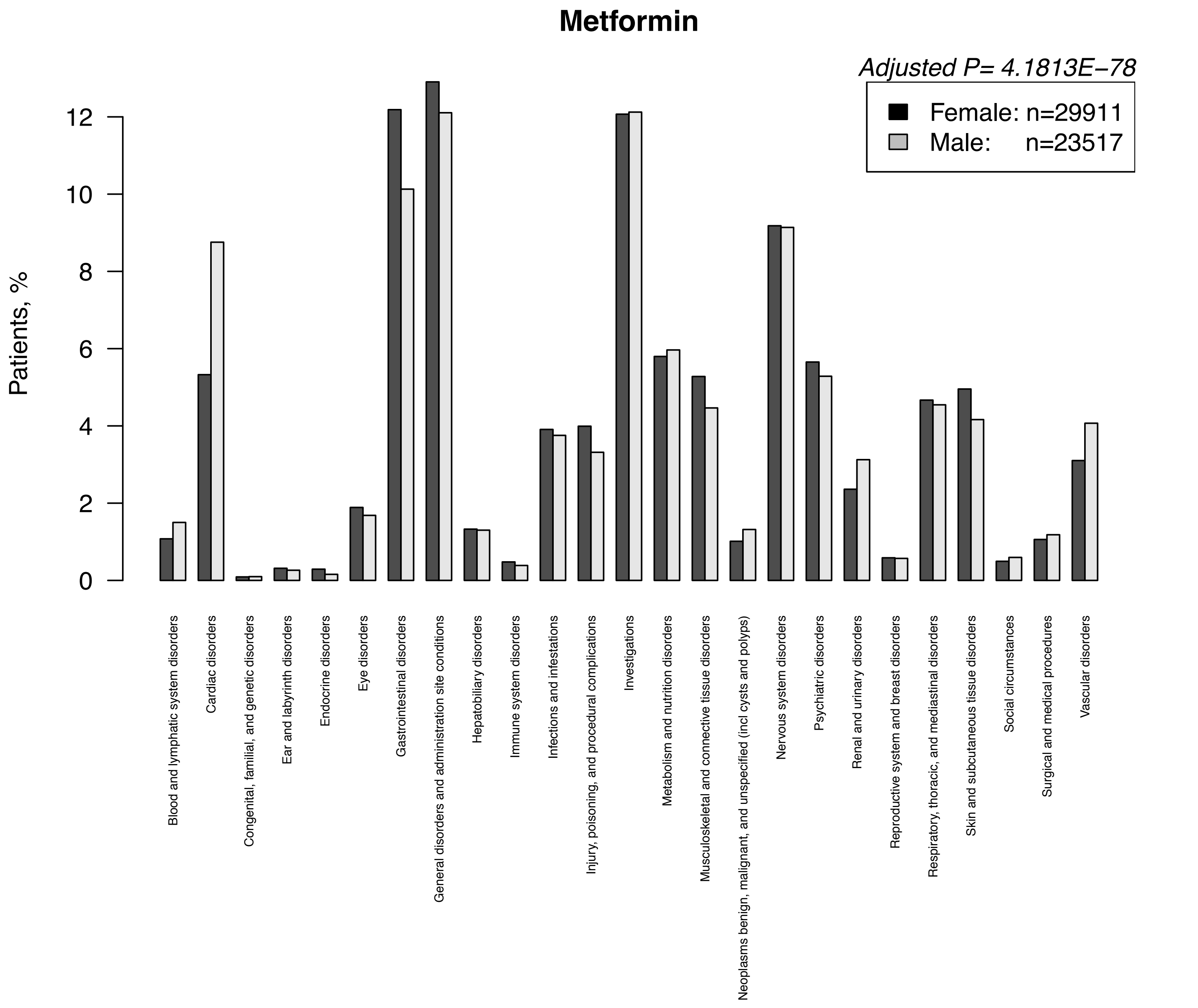

Metformin
Adjusted P= 4.1813E−78
Female: n=29911
Male: n=23517
Patients, %
0
2
4
6
8
10
12
Blood and lymphatic system disorders
Cardiac disorders
Congenital, familial, and genetic disorders
Ear and labyrinth disorders
Endocrine disorders
Eye disorders
Gastrointestinal disorders
General disorders and administration site conditions
Hepatobiliary disorders
Immune system disorders
Infections and infestations
Injury, poisoning, and procedural complications
Investigations
Metabolism and nutrition disorders
Musculoskeletal and connective tissue disorders
Neoplasms benign, malignant, and unspecified (incl cysts and polyps)
Nervous system disorders
Psychiatric disorders
Renal and urinary disorders
Reproductive system and breast disorders
Respiratory, thoracic, and mediastinal disorders
Skin and subcutaneous tissue disorders
Social circumstances
Surgical and medical procedures
Vascular disorders

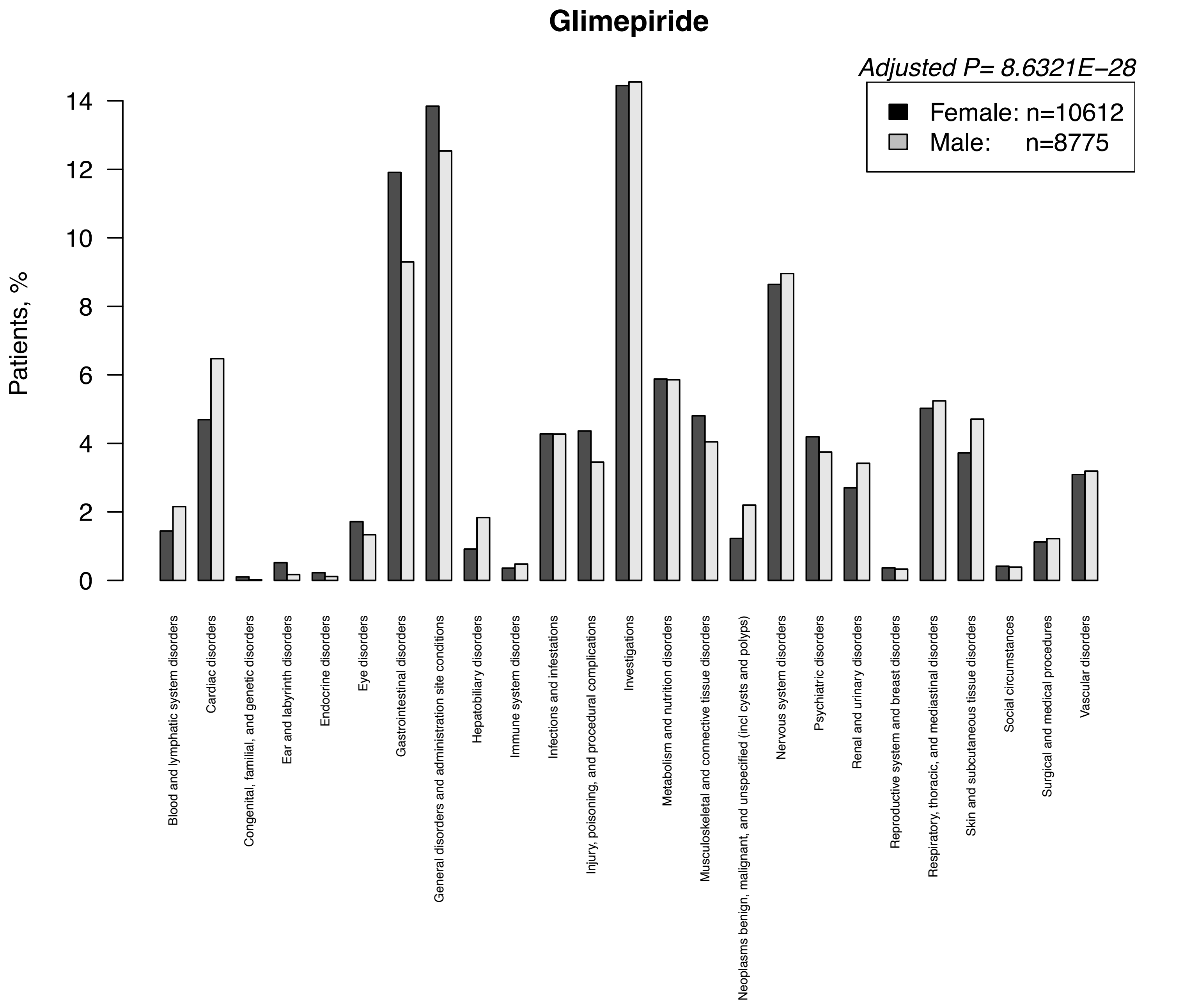
Glimepiride
Adjusted P= 8.6321E−28
Female: n=10612
Male: n=8775
Patients, %
0
2
4
6
8
10
12
14
Blood and lymphatic system disorders
Cardiac disorders
Congenital, familial, and genetic disorders
Ear and labyrinth disorders
Endocrine disorders
Eye disorders
Gastrointestinal disorders
General disorders and administration site conditions
Hepatobiliary disorders
Immune system disorders
Infections and infestations
Injury, poisoning, and procedural complications
Investigations
Metabolism and nutrition disorders
Musculoskeletal and connective tissue disorders
Neoplasms benign, malignant, and unspecified (incl cysts and polyps)
Nervous system disorders
Psychiatric disorders
Renal and urinary disorders
Reproductive system and breast disorders
Respiratory, thoracic, and mediastinal disorders
Skin and subcutaneous tissue disorders
Social circumstances
Surgical and medical procedures
Vascular disorders

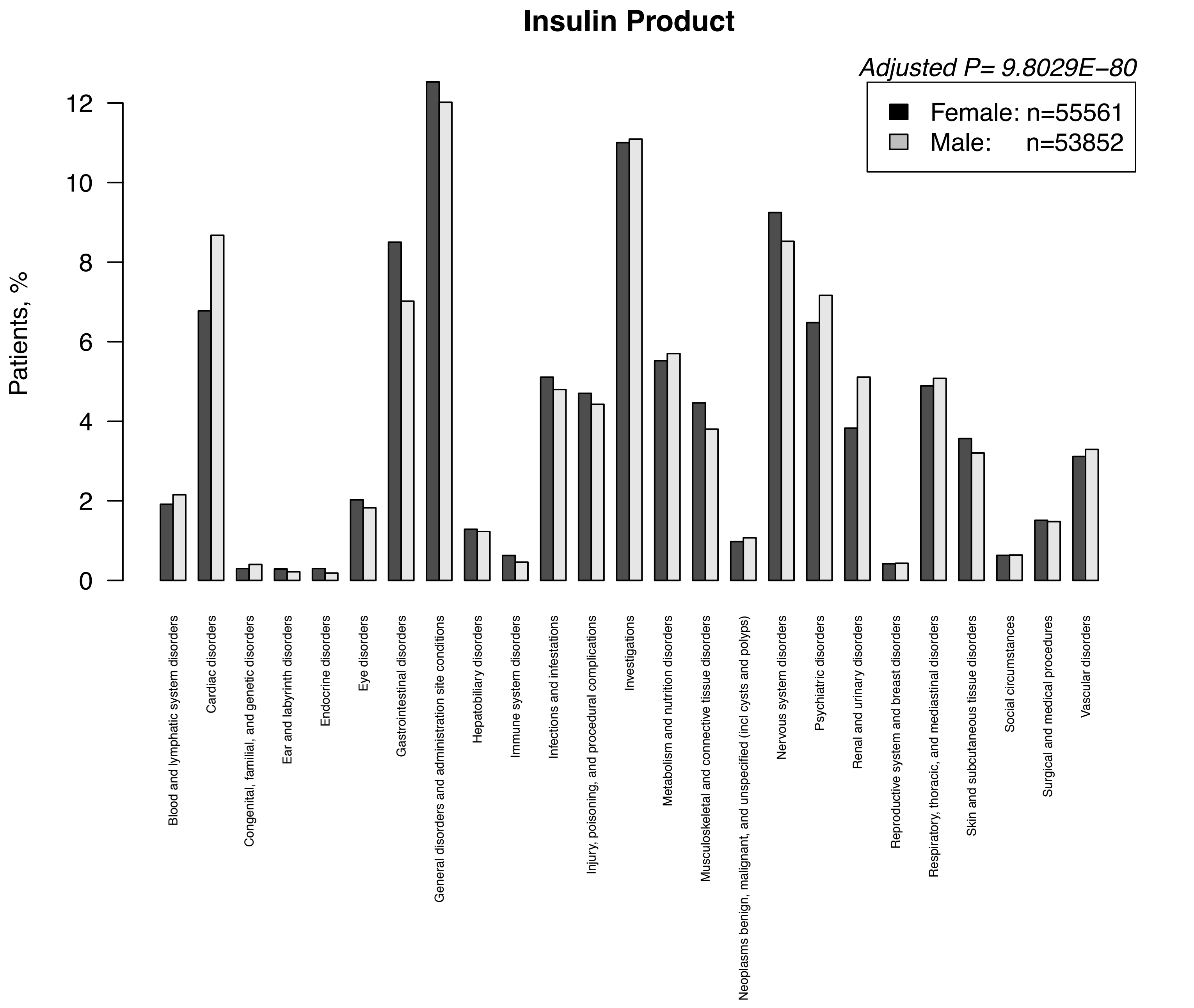

Insulin Product
Adjusted P= 9.8029E−80
Female: n=55561
Male: n=53852
Patients, %
0
2
4
6
8
10
12
Blood and lymphatic system disorders
Cardiac disorders
Congenital, familial, and genetic disorders
Ear and labyrinth disorders
Endocrine disorders
Eye disorders
Gastrointestinal disorders
General disorders and administration site conditions
Hepatobiliary disorders
Immune system disorders
Infections and infestations
Injury, poisoning, and procedural complications
Investigations
Metabolism and nutrition disorders
Musculoskeletal and connective tissue disorders
Neoplasms benign, malignant, and unspecified (incl cysts and polyps)
Nervous system disorders
Psychiatric disorders
Renal and urinary disorders
Reproductive system and breast disorders
Respiratory, thoracic, and mediastinal disorders
Skin and subcutaneous tissue disorders
Social circumstances
Surgical and medical procedures
Vascular disorders

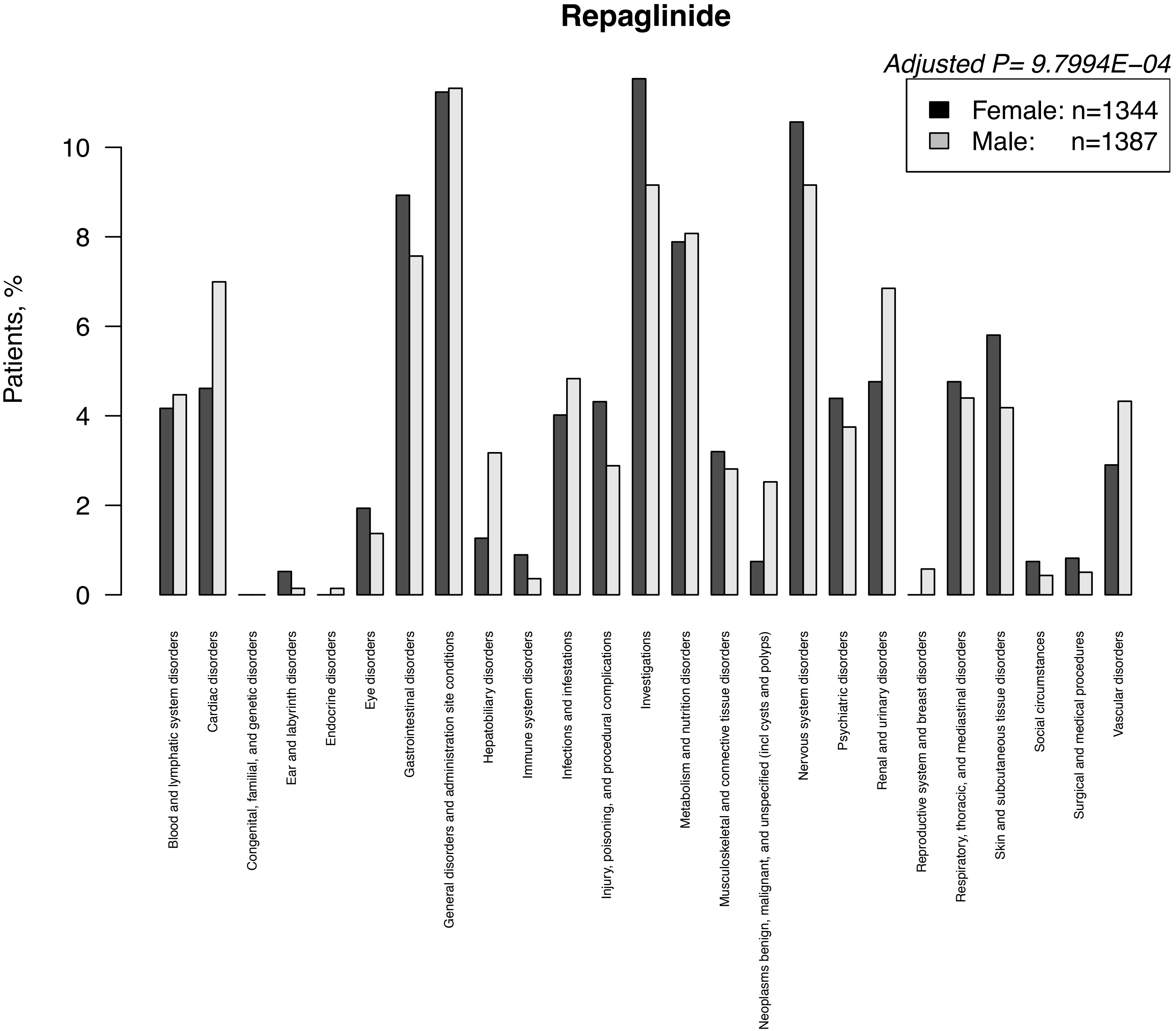
Repaglinide
Adjusted P= 9.7994E−04
Female: n=1344
Male: n=1387
Patients, %
0
2
4
6
8
10
Blood and lymphatic system disorders
Cardiac disorders
Congenital, familial, and genetic disorders
Ear and labyrinth disorders
Endocrine disorders
Eye disorders
Gastrointestinal disorders
General disorders and administration site conditions
Hepatobiliary disorders
Immune system disorders
Infections and infestations
Injury, poisoning, and procedural complications
Investigations
Metabolism and nutrition disorders
Musculoskeletal and connective tissue disorders
Neoplasms benign, malignant, and unspecified (incl cysts and polyps)
Nervous system disorders
Psychiatric disorders
Renal and urinary disorders
Reproductive system and breast disorders
Respiratory, thoracic, and mediastinal disorders
Skin and subcutaneous tissue disorders
Social circumstances
Surgical and medical procedures
Vascular disorders

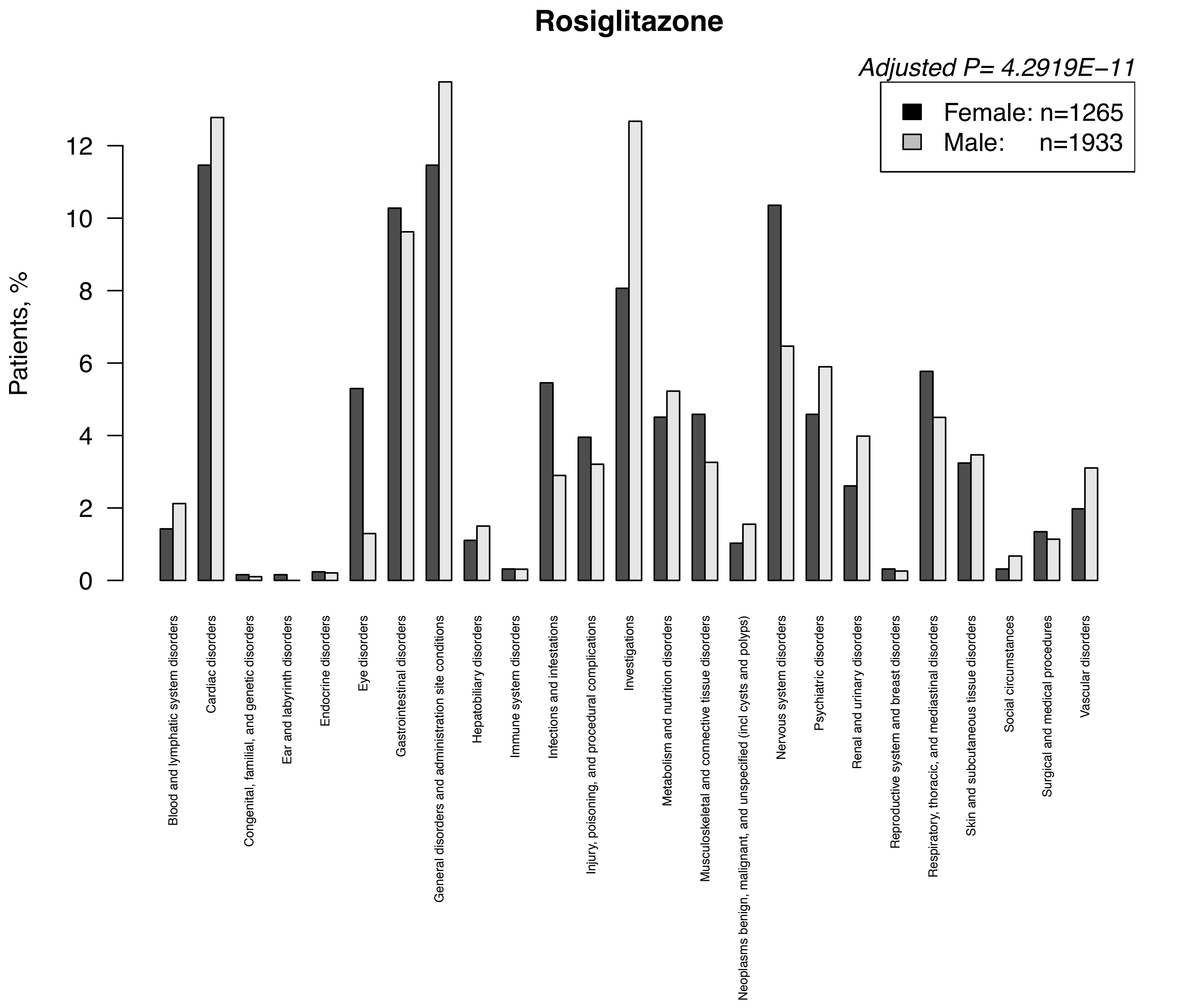

Rosiglitazone
Adjusted P= 4.2919E−11
Female: n=1265
Male: n=1933
Patients, %
0
2
4
6
8
10
12
Blood and lymphatic system disorders
Cardiac disorders
Congenital, familial, and genetic disorders
Ear and labyrinth disorders
Endocrine disorders
Eye disorders
Gastrointestinal disorders
General disorders and administration site conditions
Hepatobiliary disorders
Immune system disorders
Infections and infestations
Injury, poisoning, and procedural complications
Investigations
Metabolism and nutrition disorders
Musculoskeletal and connective tissue disorders
Neoplasms benign, malignant, and unspecified (incl cysts and polyps)
Nervous system disorders
Psychiatric disorders
Renal and urinary disorders
Reproductive system and breast disorders
Respiratory, thoracic, and mediastinal disorders
Skin and subcutaneous tissue disorders
Social circumstances
Surgical and medical procedures
Vascular disorders

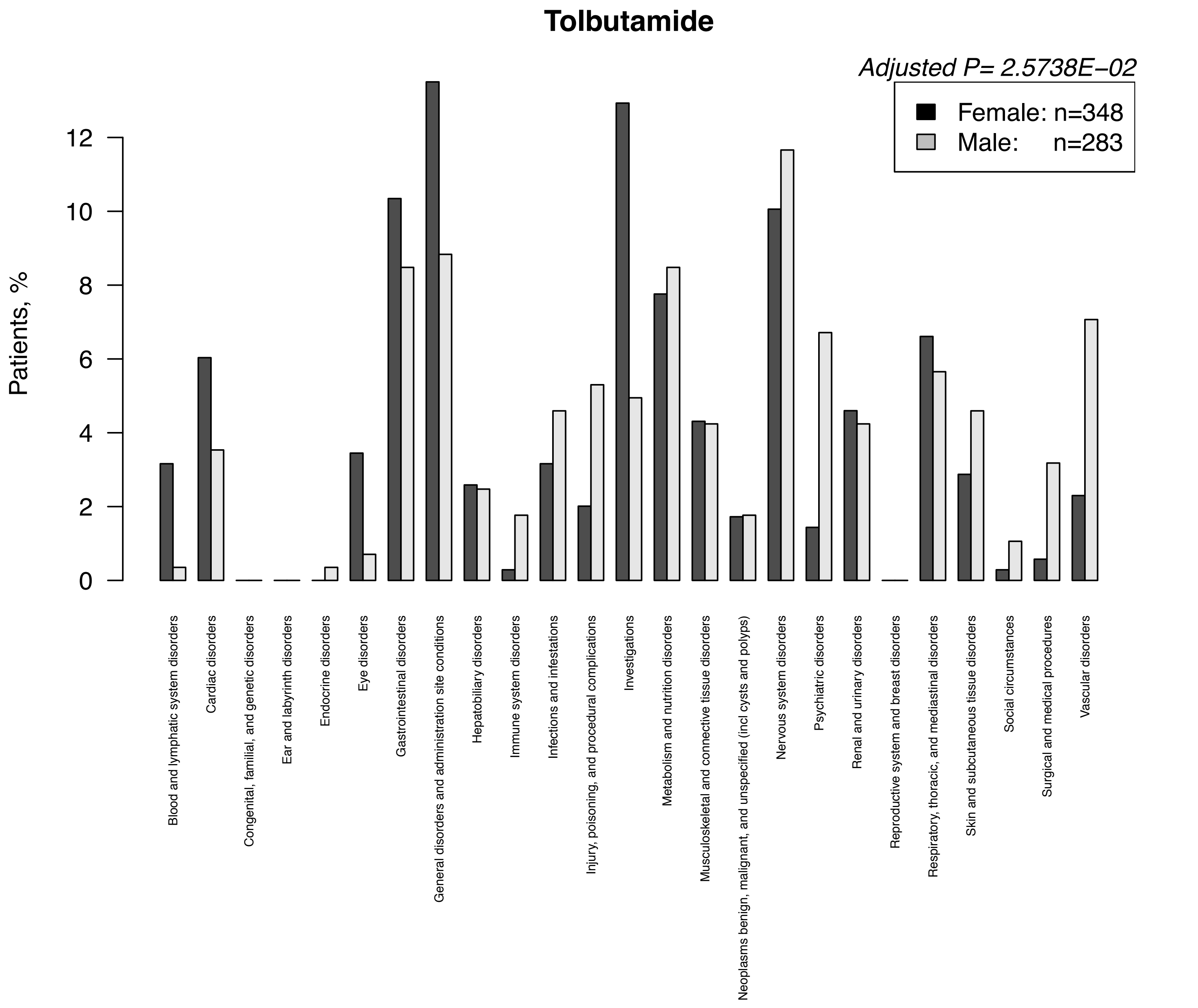
Tolbutamide
Adjusted P= 2.5738E−02
Female: n=348
Male: n=283
Patients, %
0
2
4
6
8
10
12
Blood and lymphatic system disorders
Cardiac disorders
Congenital, familial, and genetic disorders
Ear and labyrinth disorders
Endocrine disorders
Eye disorders
Gastrointestinal disorders
General disorders and administration site conditions
Hepatobiliary disorders
Immune system disorders
Infections and infestations
Injury, poisoning, and procedural complications
Investigations
Metabolism and nutrition disorders
Musculoskeletal and connective tissue disorders
Neoplasms benign, malignant, and unspecified (incl cysts and polyps)
Nervous system disorders
Psychiatric disorders
Renal and urinary disorders
Reproductive system and breast disorders
Respiratory, thoracic, and mediastinal disorders
Skin and subcutaneous tissue disorders
Social circumstances
Surgical and medical procedures
Vascular disorders

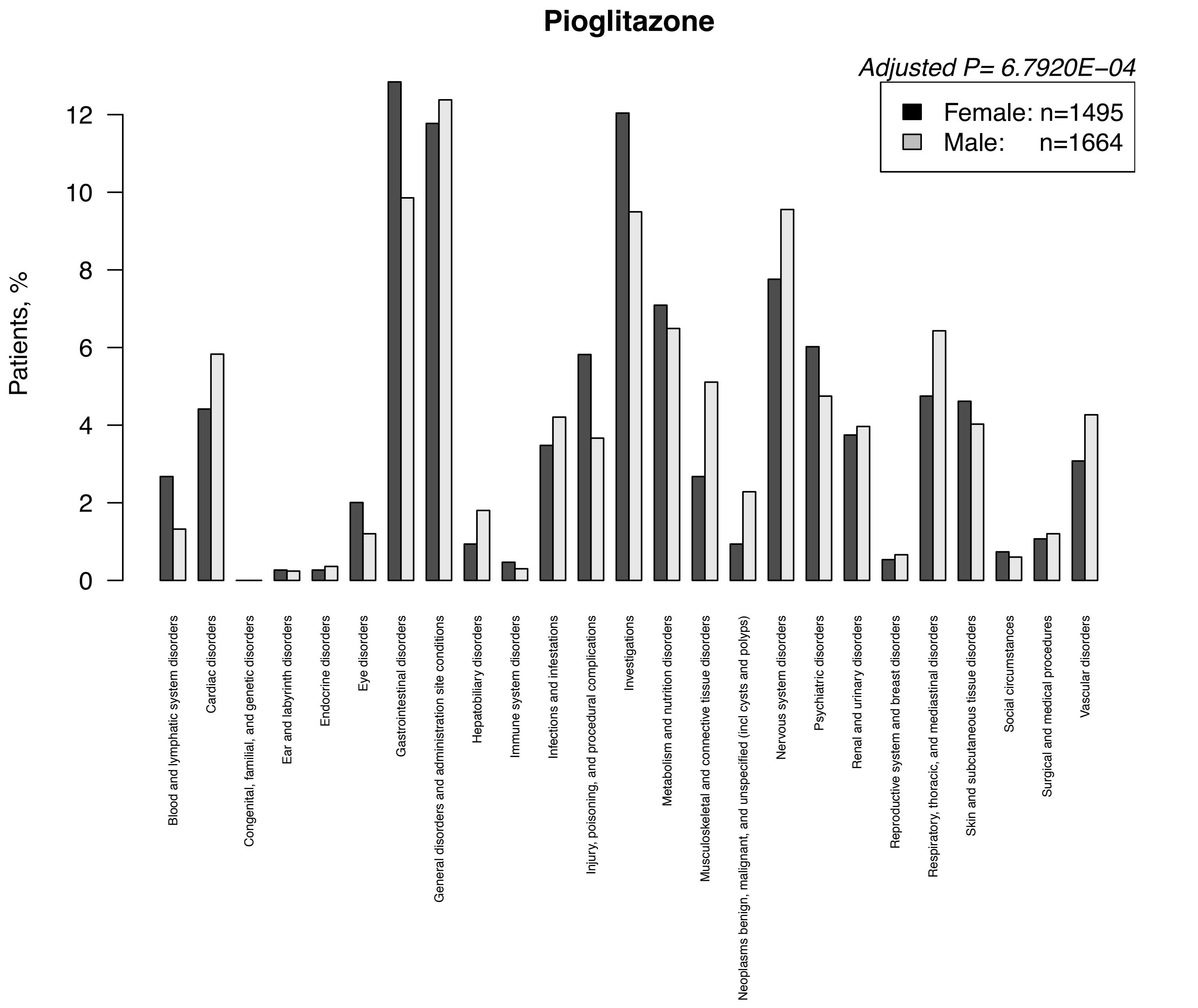

Pioglitazone
Adjusted P= 6.7920E−04
Female: n=1495
Male: n=1664
Patients, %
0
2
4
6
8
10
12
Blood and lymphatic system disorders
Cardiac disorders
Congenital, familial, and genetic disorders
Ear and labyrinth disorders
Endocrine disorders
Eye disorders
Gastrointestinal disorders
General disorders and administration site conditions
Hepatobiliary disorders
Immune system disorders
Infections and infestations
Injury, poisoning, and procedural complications
Investigations
Metabolism and nutrition disorders
Musculoskeletal and connective tissue disorders
Neoplasms benign, malignant, and unspecified (incl cysts and polyps)
Nervous system disorders
Psychiatric disorders
Renal and urinary disorders
Reproductive system and breast disorders
Respiratory, thoracic, and mediastinal disorders
Skin and subcutaneous tissue disorders
Social circumstances
Surgical and medical procedures
Vascular disorders

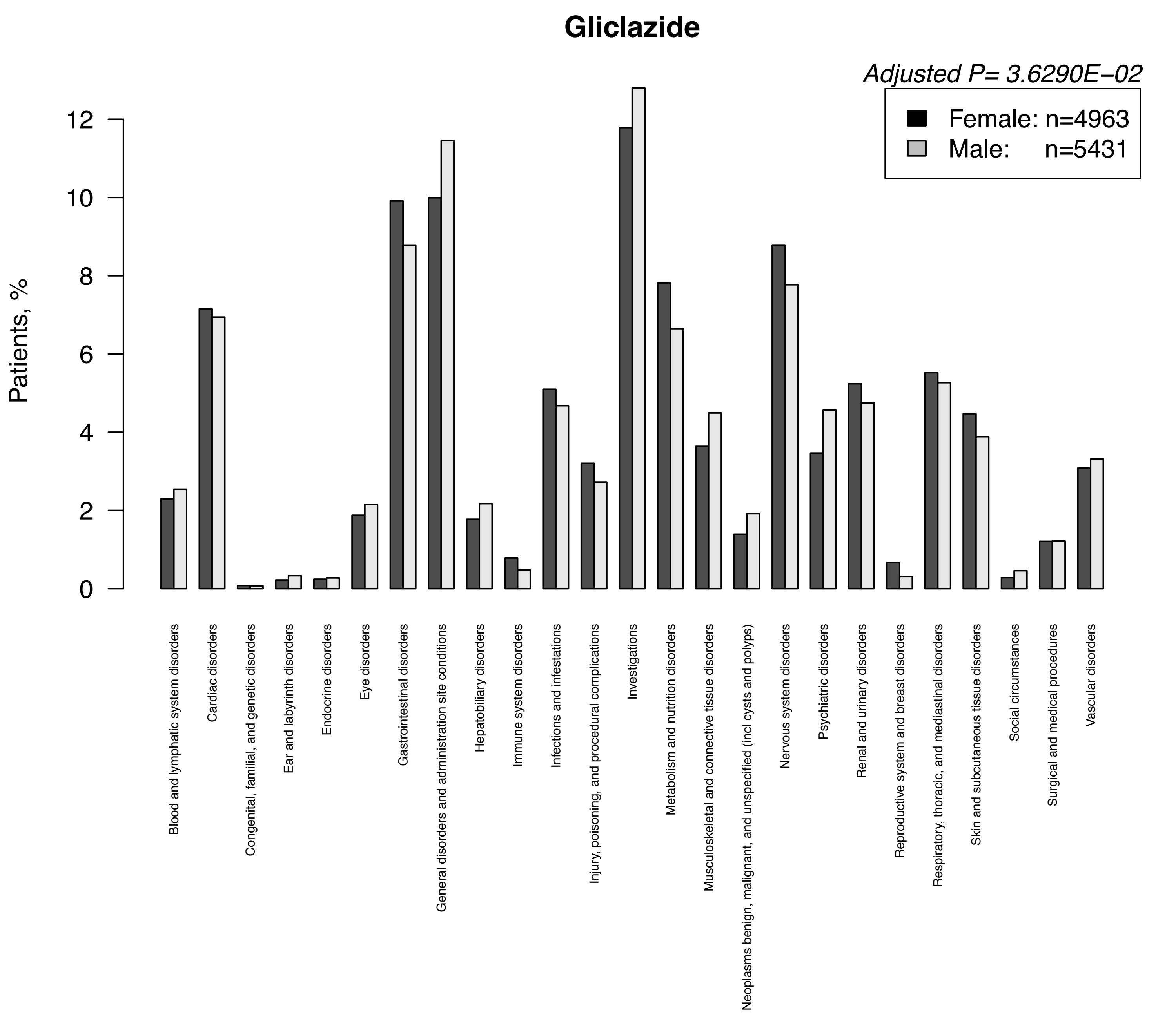

Gliclazide
Adjusted P= 3.6290E−02
Female: n=4963
Male: n=5431
Patients, %
0
2
4
6
8
10
12
Blood and lymphatic system disorders
Cardiac disorders
Congenital, familial, and genetic disorders
Ear and labyrinth disorders
Endocrine disorders
Eye disorders
Gastrointestinal disorders
General disorders and administration site conditions
Hepatobiliary disorders
Immune system disorders
Infections and infestations
Injury, poisoning, and procedural complications
Investigations
Metabolism and nutrition disorders
Musculoskeletal and connective tissue disorders
Neoplasms benign, malignant, and unspecified (incl cysts and polyps)
Nervous system disorders
Psychiatric disorders
Renal and urinary disorders
Reproductive system and breast disorders
Respiratory, thoracic, and mediastinal disorders
Skin and subcutaneous tissue disorders
Social circumstances
Surgical and medical procedures
Vascular disorders

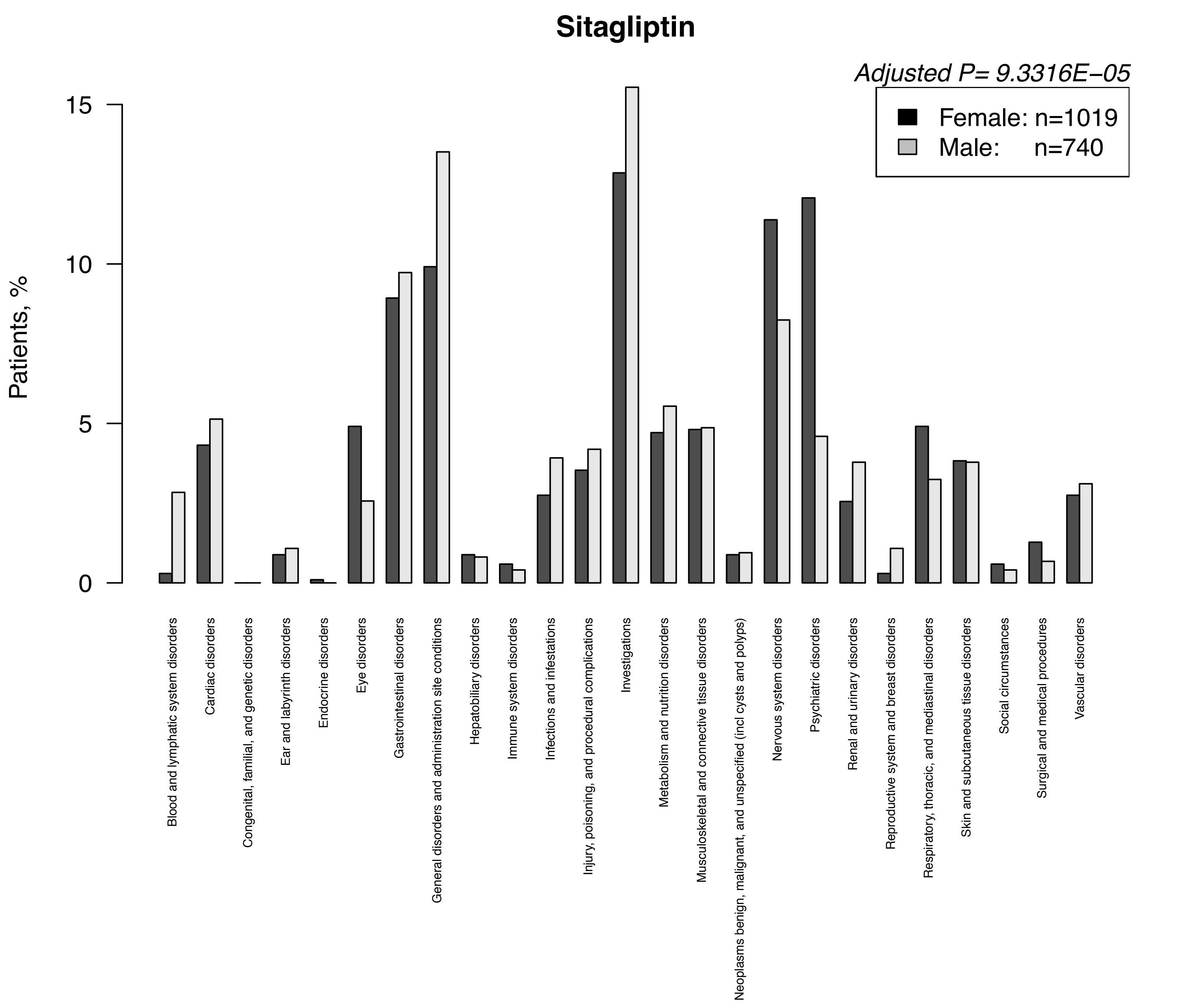

Sitagliptin
Adjusted P= 9.3316E−05
Female: n=1019
Male: n=740
Patients, %
15
10
5
0
Blood and lymphatic system disorders
Cardiac disorders
Congenital, familial, and genetic disorders
Ear and labyrinth disorders
Endocrine disorders
Eye disorders
Gastrointestinal disorders
General disorders and administration site conditions
Hepatobiliary disorders
Immune system disorders
Infections and infestations
Injury, poisoning, and procedural complications
Investigations
Metabolism and nutrition disorders
Musculoskeletal and connective tissue disorders
Neoplasms benign, malignant, and unspecified (incl cysts and polyps)
Nervous system disorders
Psychiatric disorders
Renal and urinary disorders
Reproductive system and breast disorders
Respiratory, thoracic, and mediastinal disorders
Skin and subcutaneous tissue disorders
Social circumstances
Surgical and medical procedures
Vascular disorders

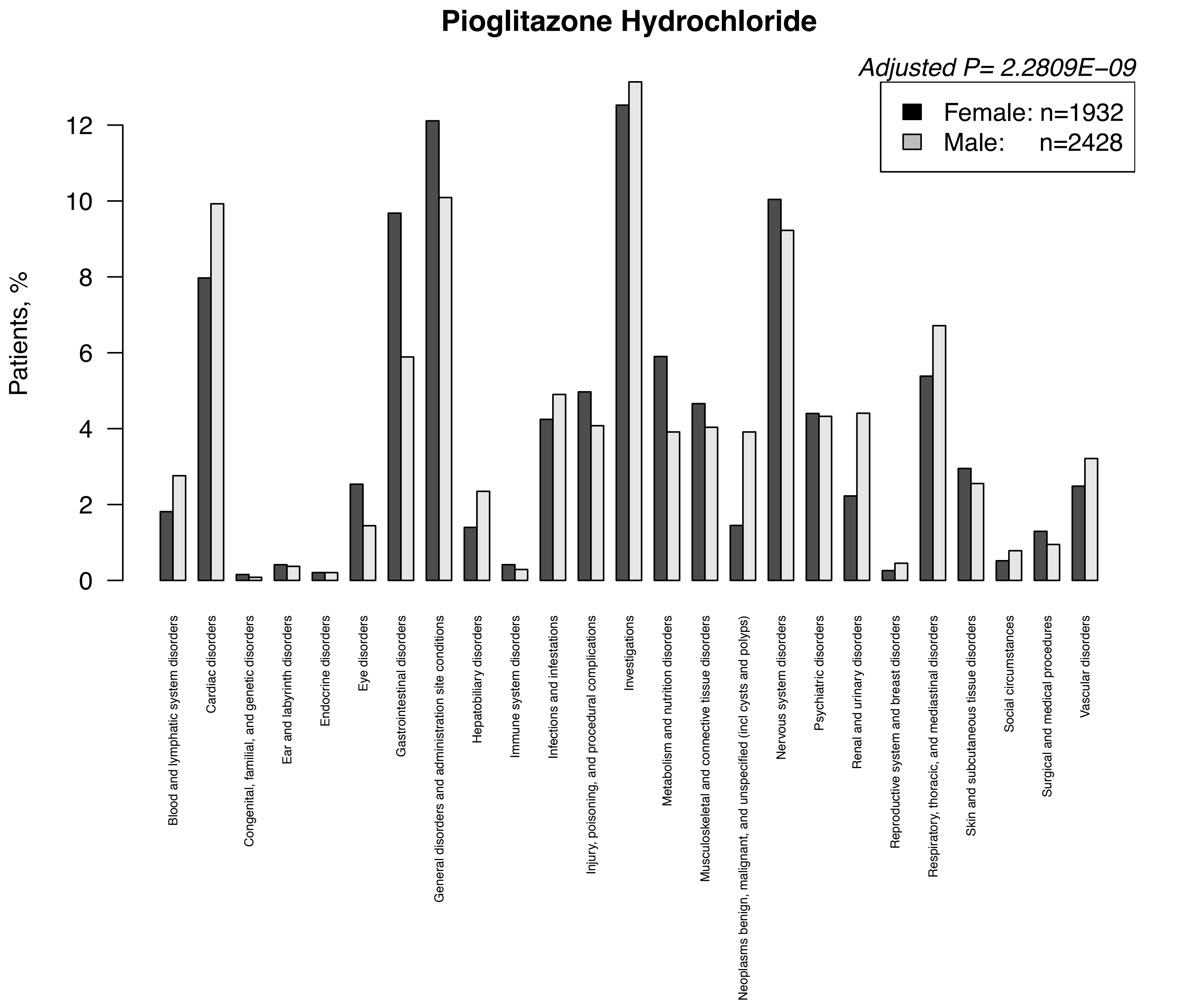

Pioglitazone Hydrochloride
Adjusted P= 2.2809E−09
Female: n=1932
Male: n=2428
Patients, %
0
2
4
6
8
10
12
Blood and lymphatic system disorders
Cardiac disorders
Congenital, familial, and genetic disorders
Ear and labyrinth disorders
Endocrine disorders
Eye disorders
Gastrointestinal disorders
General disorders and administration site conditions
Hepatobiliary disorders
Immune system disorders
Infections and infestations
Injury, poisoning, and procedural complications
Investigations
Metabolism and nutrition disorders
Musculoskeletal and connective tissue disorders
Neoplasms benign, malignant, and unspecified (incl cysts and polyps)
Nervous system disorders
Psychiatric disorders
Renal and urinary disorders
Reproductive system and breast disorders
Respiratory, thoracic, and mediastinal disorders
Skin and subcutaneous tissue disorders
Social circumstances
Surgical and medical procedures
Vascular disorders

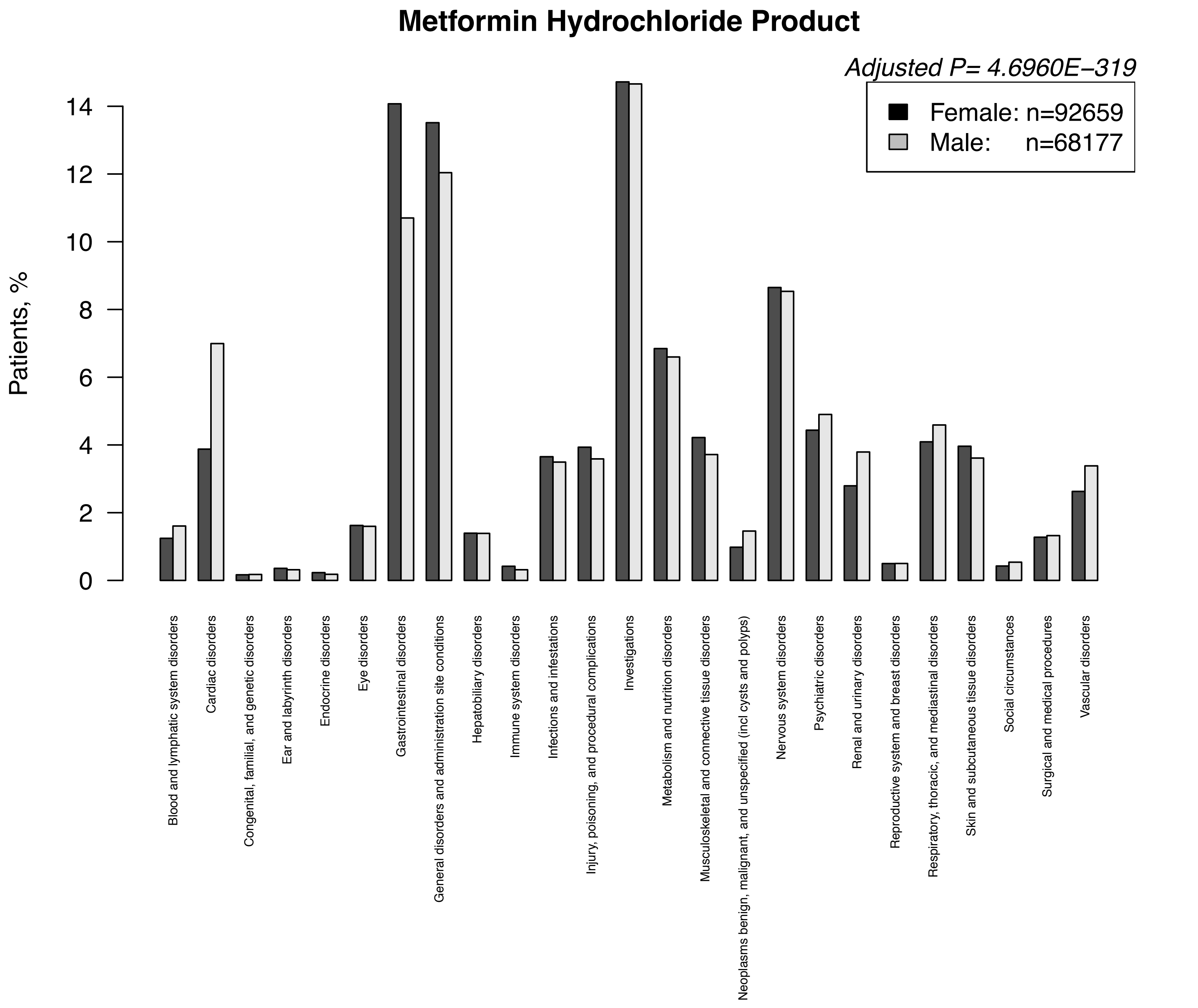
Metformin Hydrochloride Product
Adjusted P= 4.6960E−319
Female: n=92659
Male: n=68177
Patients, %
0
2
4
6
8
10
12
14
Blood and lymphatic system disorders
Cardiac disorders
Congenital, familial, and genetic disorders
Ear and labyrinth disorders
Endocrine disorders
Eye disorders
Gastrointestinal disorders
General disorders and administration site conditions
Hepatobiliary disorders
Immune system disorders
Infections and infestations
Injury, poisoning, and procedural complications
Investigations
Metabolism and nutrition disorders
Musculoskeletal and connective tissue disorders
Neoplasms benign, malignant, and unspecified (incl cysts and polyps)
Nervous system disorders
Psychiatric disorders
Renal and urinary disorders
Reproductive system and breast disorders
Respiratory, thoracic, and mediastinal disorders
Skin and subcutaneous tissue disorders
Social circumstances
Surgical and medical procedures
Vascular disorders

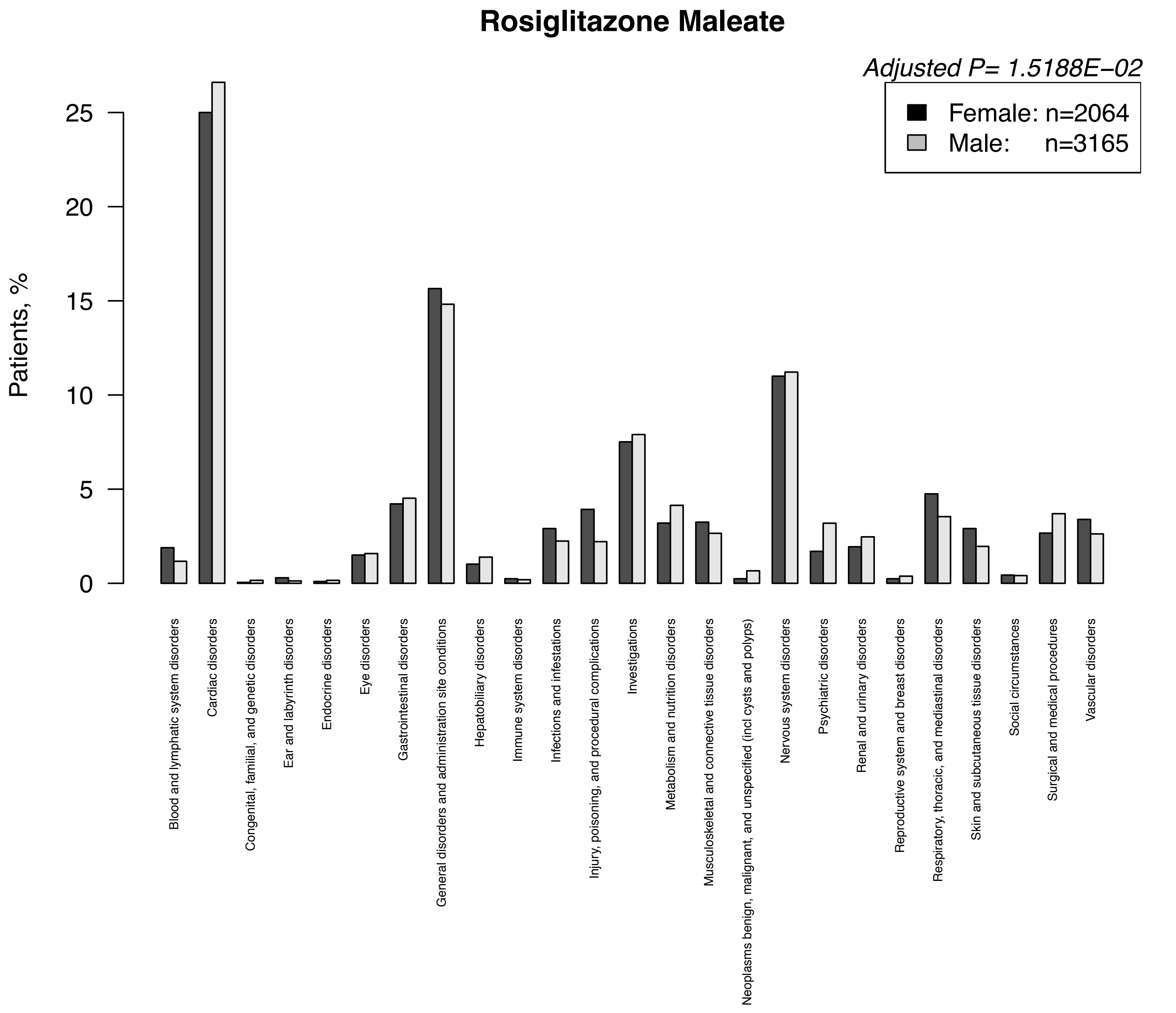
Rosiglitazone Maleate
Adjusted P= 1.5188E−02
Female: n=2064
Male: n=3165
Patients, %
0
5
10
15
20
25
Blood and lymphatic system disorders
Cardiac disorders
Congenital, familial, and genetic disorders
Ear and labyrinth disorders
Endocrine disorders
Eye disorders
Gastrointestinal disorders
General disorders and administration site conditions
Hepatobiliary disorders
Immune system disorders
Infections and infestations
Injury, poisoning, and procedural complications
Investigations
Metabolism and nutrition disorders
Musculoskeletal and connective tissue disorders
Neoplasms benign, malignant, and unspecified (incl cysts and polyps)
Nervous system disorders
Psychiatric disorders
Renal and urinary disorders
Reproductive system and breast disorders
Respiratory, thoracic, and mediastinal disorders
Skin and subcutaneous tissue disorders
Social circumstances
Surgical and medical procedures
Vascular disorders

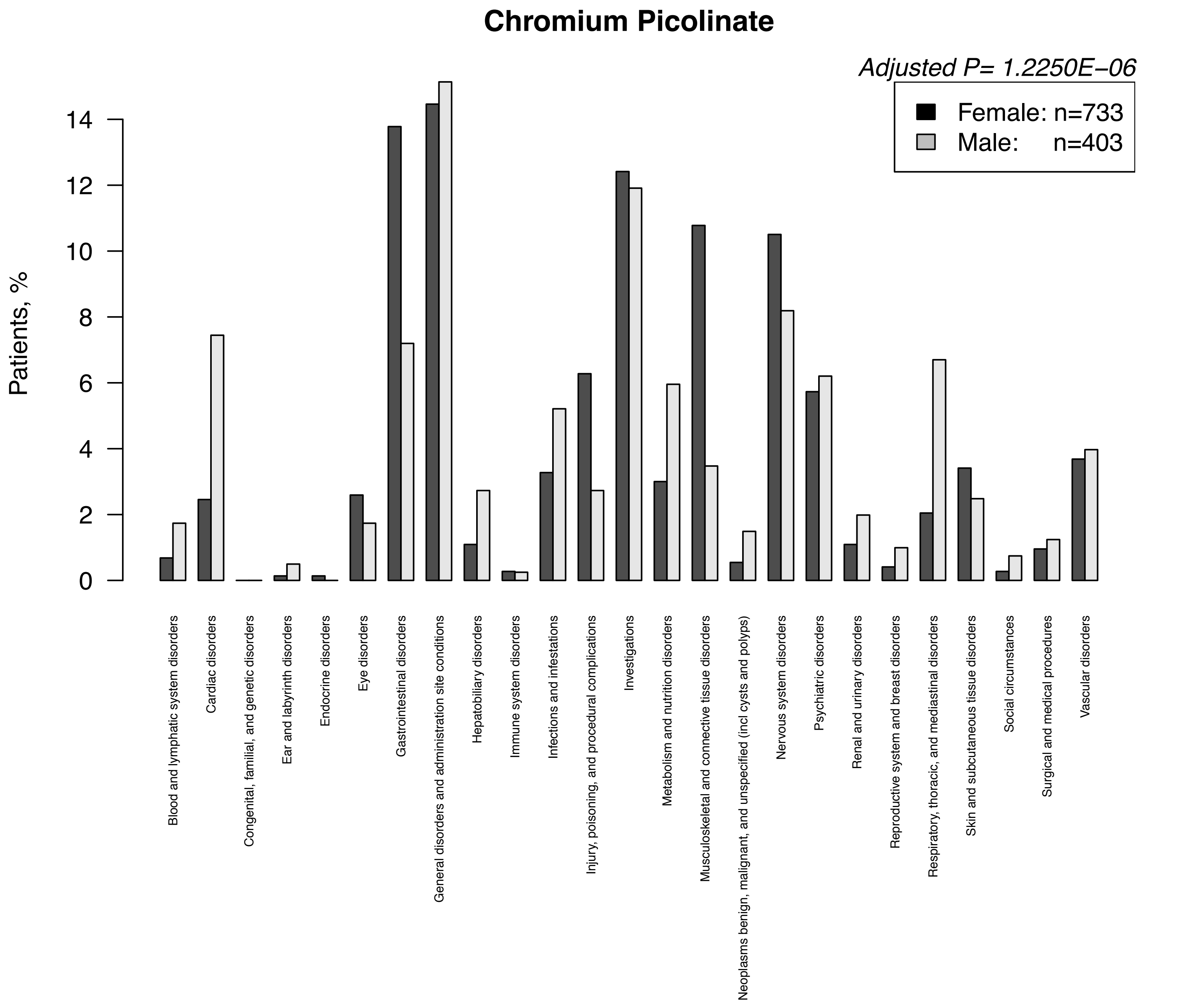
Chromium Picolinate
Adjusted P= 1.2250E−06
Female: n=733
Male: n=403
Patients, %
0
2
4
6
8
10
12
14
Blood and lymphatic system disorders
Cardiac disorders
Congenital, familial, and genetic disorders
Ear and labyrinth disorders
Endocrine disorders
Eye disorders
Gastrointestinal disorders
General disorders and administration site conditions
Hepatobiliary disorders
Immune system disorders
Infections and infestations
Injury, poisoning, and procedural complications
Investigations
Metabolism and nutrition disorders
Musculoskeletal and connective tissue disorders
Neoplasms benign, malignant, and unspecified (incl cysts and polyps)
Nervous system disorders
Psychiatric disorders
Renal and urinary disorders
Reproductive system and breast disorders
Respiratory, thoracic, and mediastinal disorders
Skin and subcutaneous tissue disorders
Social circumstances
Surgical and medical procedures
Vascular disorders

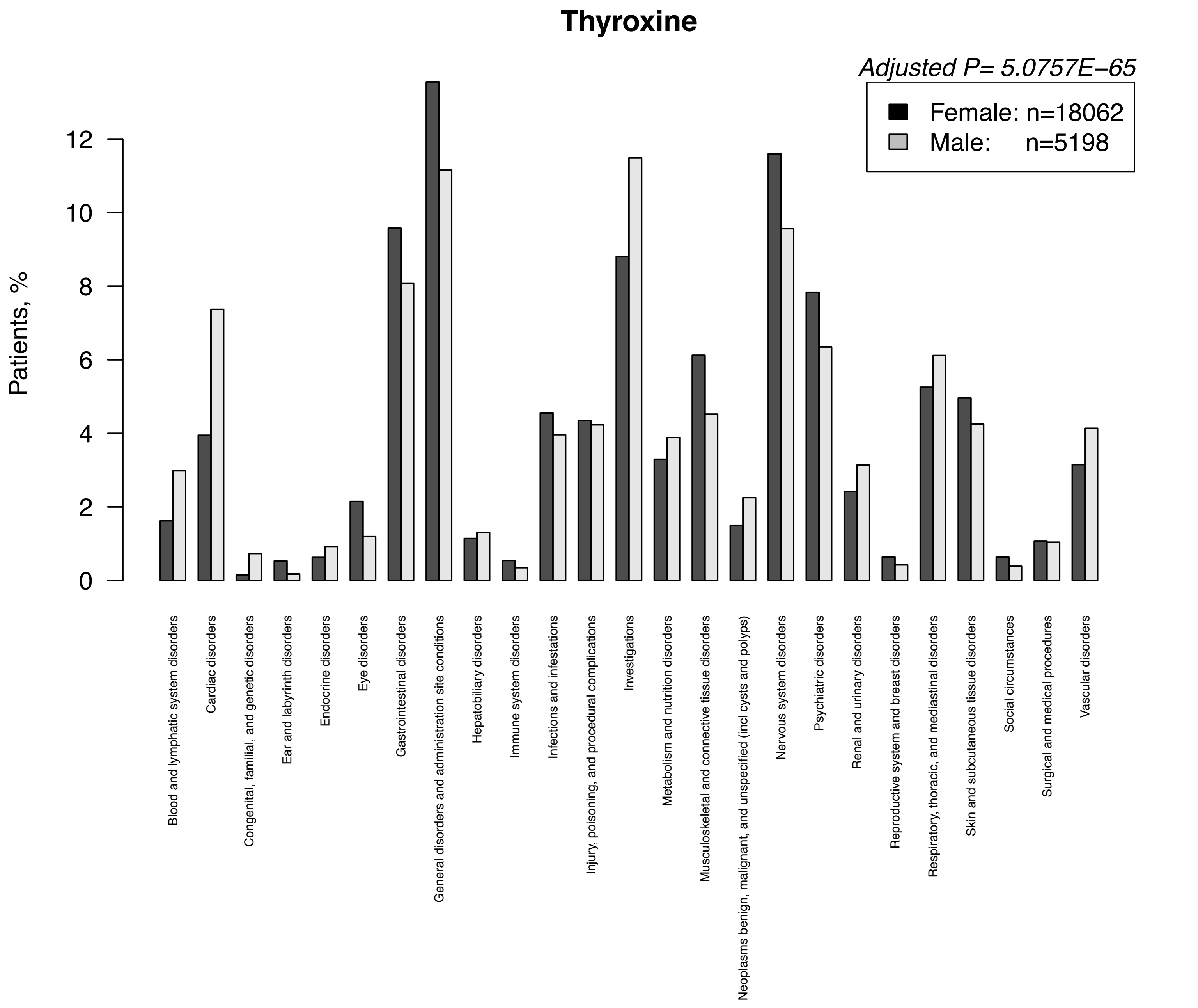
Thyroxine
Adjusted P= 5.0757E−65
Female: n=18062
Male: n=5198
Patients, %
0
2
4
6
8
10
12
Blood and lymphatic system disorders
Cardiac disorders
Congenital, familial, and genetic disorders
Ear and labyrinth disorders
Endocrine disorders
Eye disorders
Gastrointestinal disorders
General disorders and administration site conditions
Hepatobiliary disorders
Immune system disorders
Infections and infestations
Injury, poisoning, and procedural complications
Investigations
Metabolism and nutrition disorders
Musculoskeletal and connective tissue disorders
Neoplasms benign, malignant, and unspecified (incl cysts and polyps)
Nervous system disorders
Psychiatric disorders
Renal and urinary disorders
Reproductive system and breast disorders
Respiratory, thoracic, and mediastinal disorders
Skin and subcutaneous tissue disorders
Social circumstances
Surgical and medical procedures
Vascular disorders

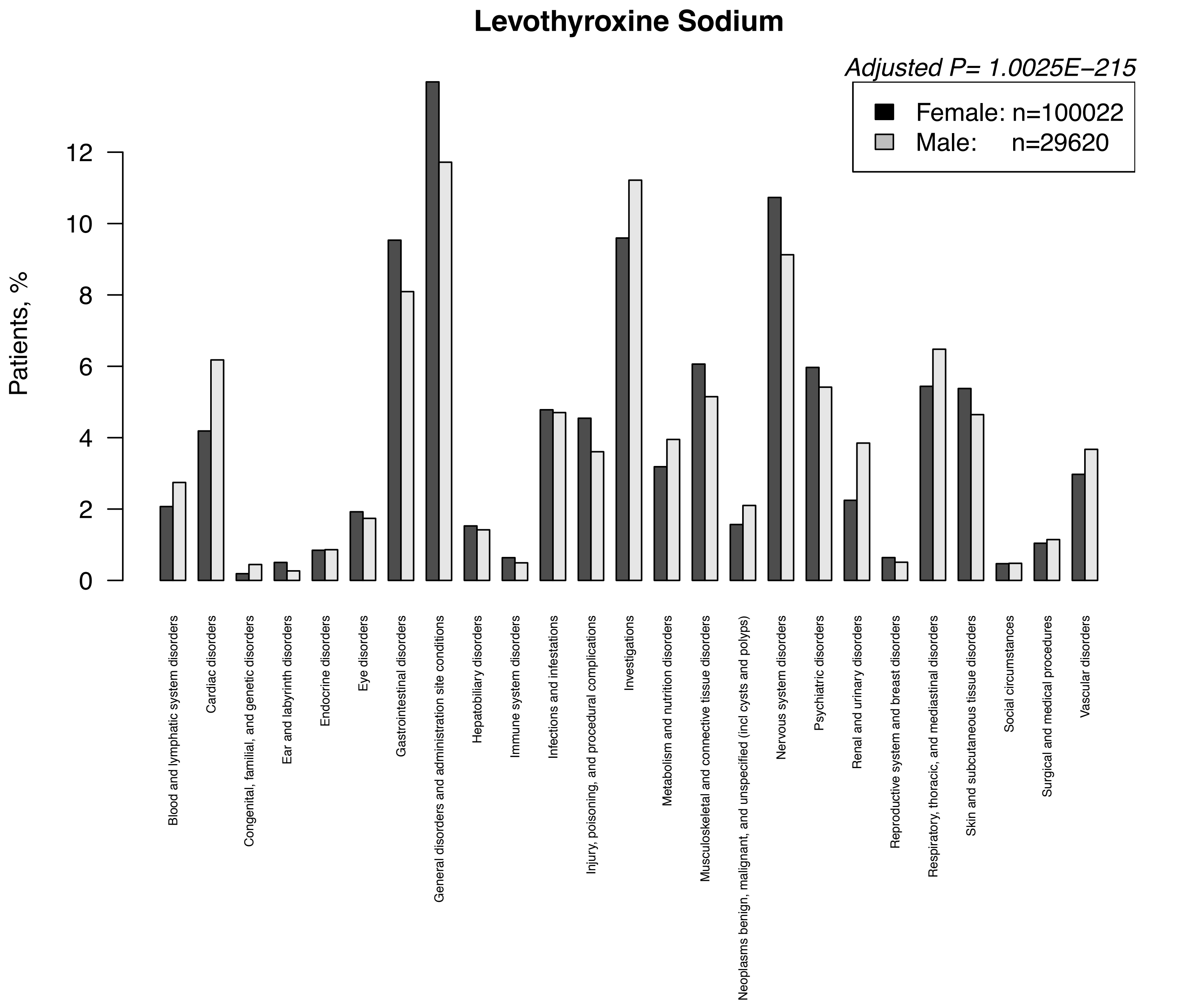
Levothyroxine Sodium
Adjusted P= 1.0025E−215
Female: n=100022
Male: n=29620
Patients, %
0
2
4
6
8
10
12
Blood and lymphatic system disorders
Cardiac disorders
Congenital, familial, and genetic disorders
Ear and labyrinth disorders
Endocrine disorders
Eye disorders
Gastrointestinal disorders
General disorders and administration site conditions
Hepatobiliary disorders
Immune system disorders
Infections and infestations
Injury, poisoning, and procedural complications
Investigations
Metabolism and nutrition disorders
Musculoskeletal and connective tissue disorders
Neoplasms benign, malignant, and unspecified (incl cysts and polyps)
Nervous system disorders
Psychiatric disorders
Renal and urinary disorders
Reproductive system and breast disorders
Respiratory, thoracic, and mediastinal disorders
Skin and subcutaneous tissue disorders
Social circumstances
Surgical and medical procedures
Vascular disorders

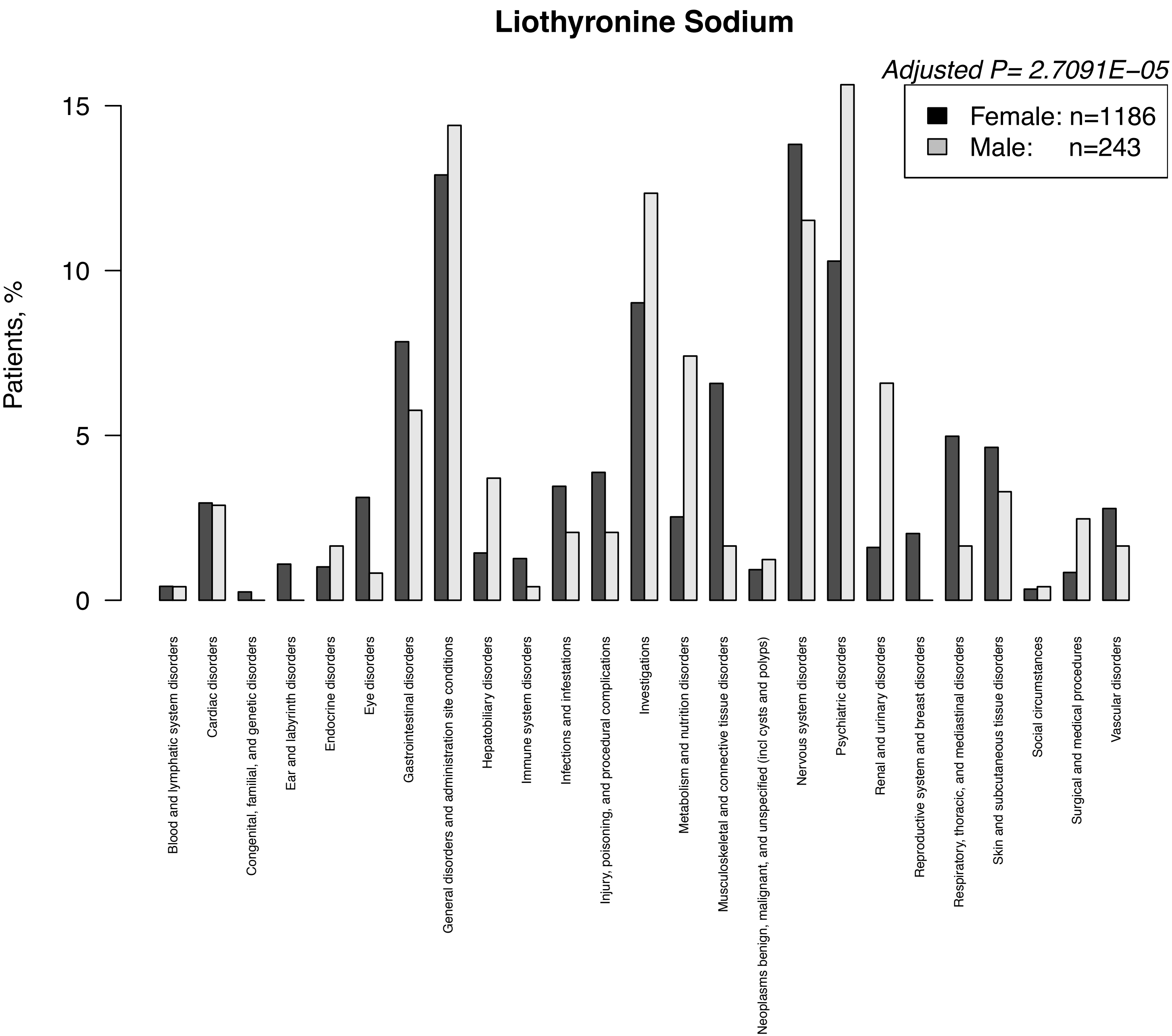
Liothyronine Sodium
Adjusted P= 2.7091E−05
Female: n=1186
Male: n=243
Patients, %
0
5
10
15
Blood and lymphatic system disorders
Cardiac disorders
Congenital, familial, and genetic disorders
Ear and labyrinth disorders
Endocrine disorders
Eye disorders
Gastrointestinal disorders
General disorders and administration site conditions
Hepatobiliary disorders
Immune system disorders
Infections and infestations
Injury, poisoning, and procedural complications
Investigations
Metabolism and nutrition disorders
Musculoskeletal and connective tissue disorders
Neoplasms benign, malignant, and unspecified (incl cysts and polyps)
Nervous system disorders
Psychiatric disorders
Renal and urinary disorders
Reproductive system and breast disorders
Respiratory, thoracic, and mediastinal disorders
Skin and subcutaneous tissue disorders
Social circumstances
Surgical and medical procedures
Vascular disorders

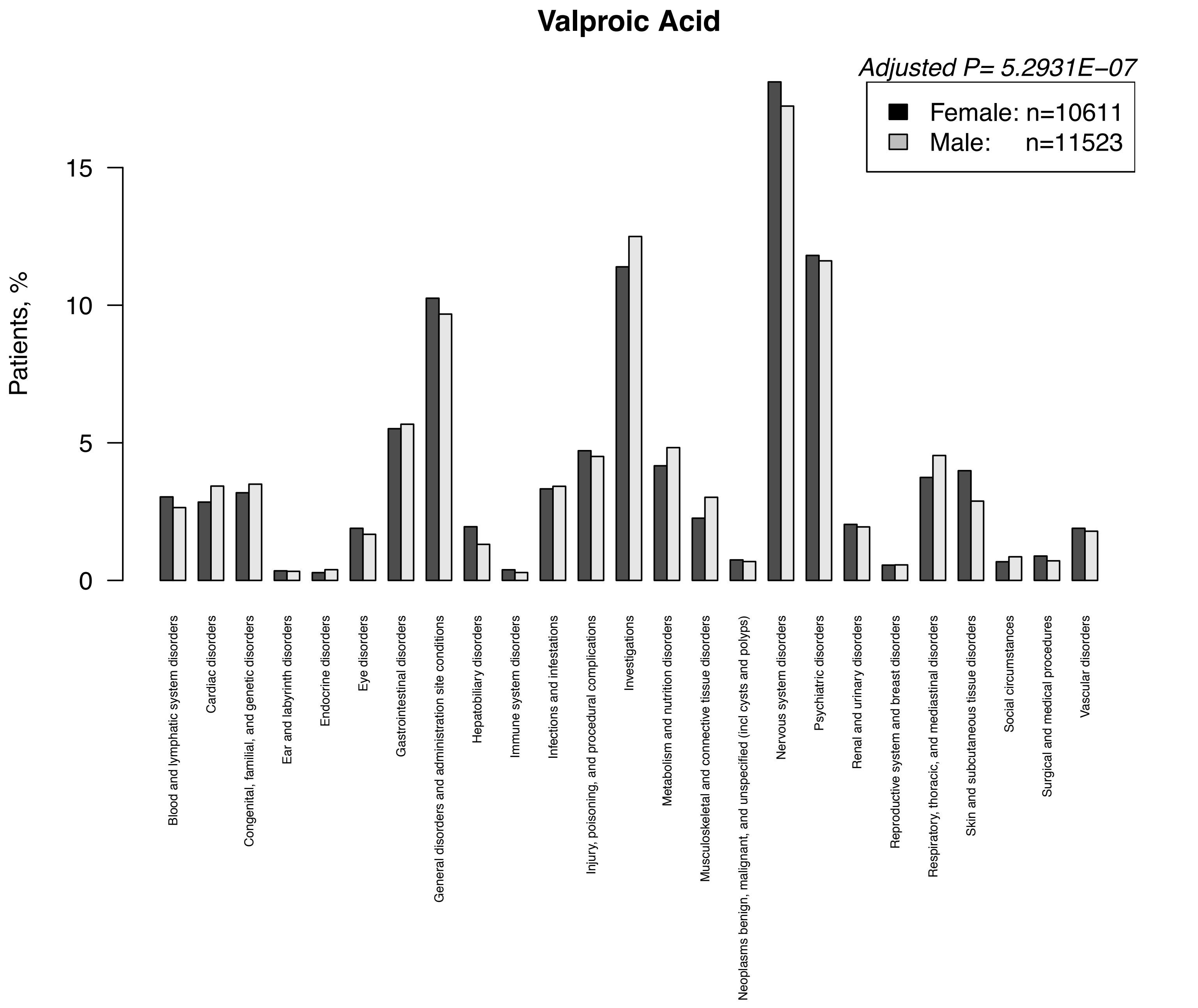
Valproic Acid
Adjusted P= 5.2931E−07
Female: n=10611
Male: n=11523
Patients, %
0
5
10
15
Blood and lymphatic system disorders
Cardiac disorders
Congenital, familial, and genetic disorders
Ear and labyrinth disorders
Endocrine disorders
Eye disorders
Gastrointestinal disorders
General disorders and administration site conditions
Hepatobiliary disorders
Immune system disorders
Infections and infestations
Injury, poisoning, and procedural complications
Investigations
Metabolism and nutrition disorders
Musculoskeletal and connective tissue disorders
Neoplasms benign, malignant, and unspecified (incl cysts and polyps)
Nervous system disorders
Psychiatric disorders
Renal and urinary disorders
Reproductive system and breast disorders
Respiratory, thoracic, and mediastinal disorders
Skin and subcutaneous tissue disorders
Social circumstances
Surgical and medical procedures
Vascular disorders

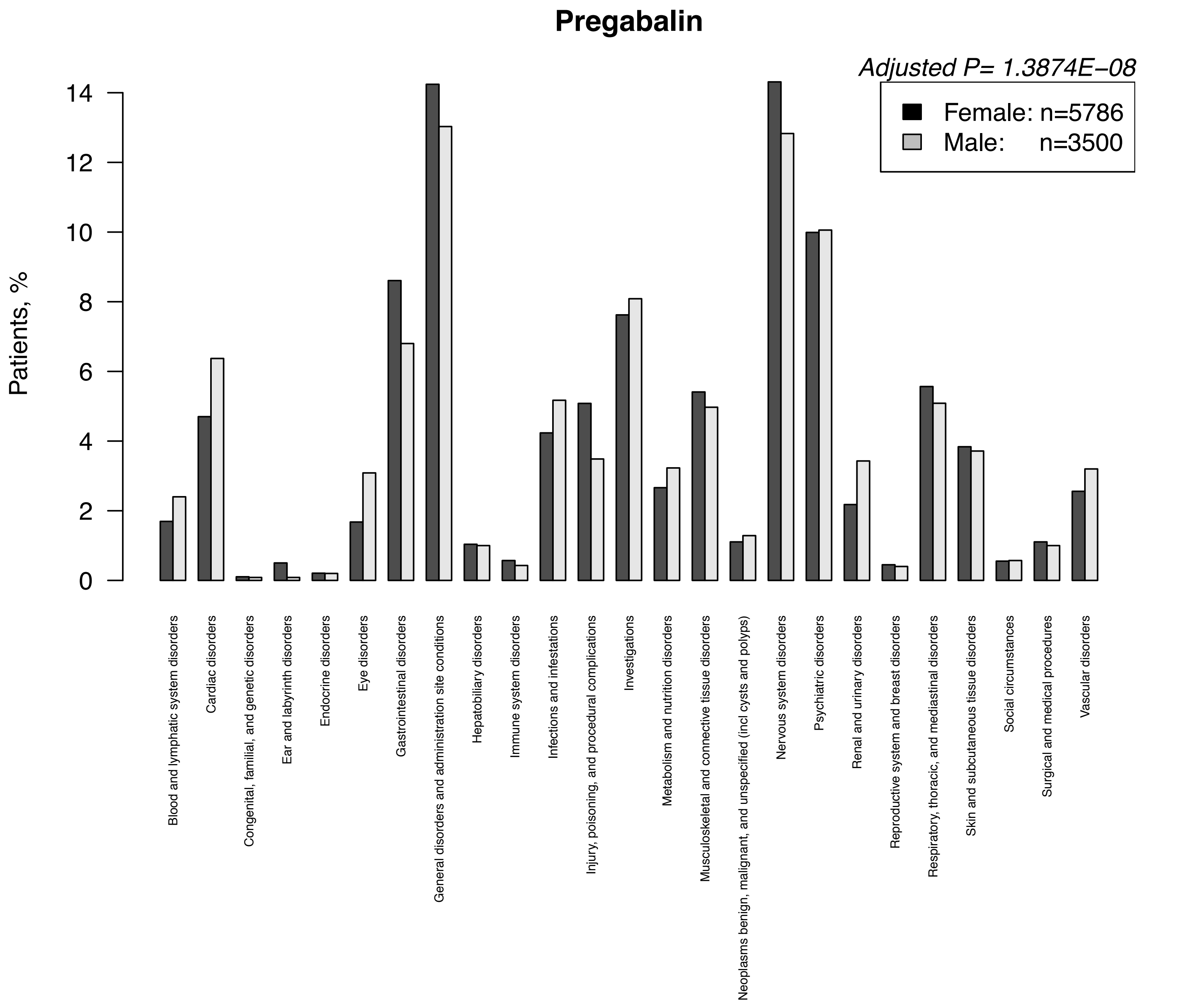

Pregabalin
Adjusted P= 1.3874E−08
Female: n=5786
Male: n=3500
Patients, %
0
2
4
6
8
10
12
14
Blood and lymphatic system disorders
Cardiac disorders
Congenital, familial, and genetic disorders
Ear and labyrinth disorders
Endocrine disorders
Eye disorders
Gastrointestinal disorders
General disorders and administration site conditions
Hepatobiliary disorders
Immune system disorders
Infections and infestations
Injury, poisoning, and procedural complications
Investigations
Metabolism and nutrition disorders
Musculoskeletal and connective tissue disorders
Neoplasms benign, malignant, and unspecified (incl cysts and polyps)
Nervous system disorders
Psychiatric disorders
Renal and urinary disorders
Reproductive system and breast disorders
Respiratory, thoracic, and mediastinal disorders
Skin and subcutaneous tissue disorders
Social circumstances
Surgical and medical procedures
Vascular disorders

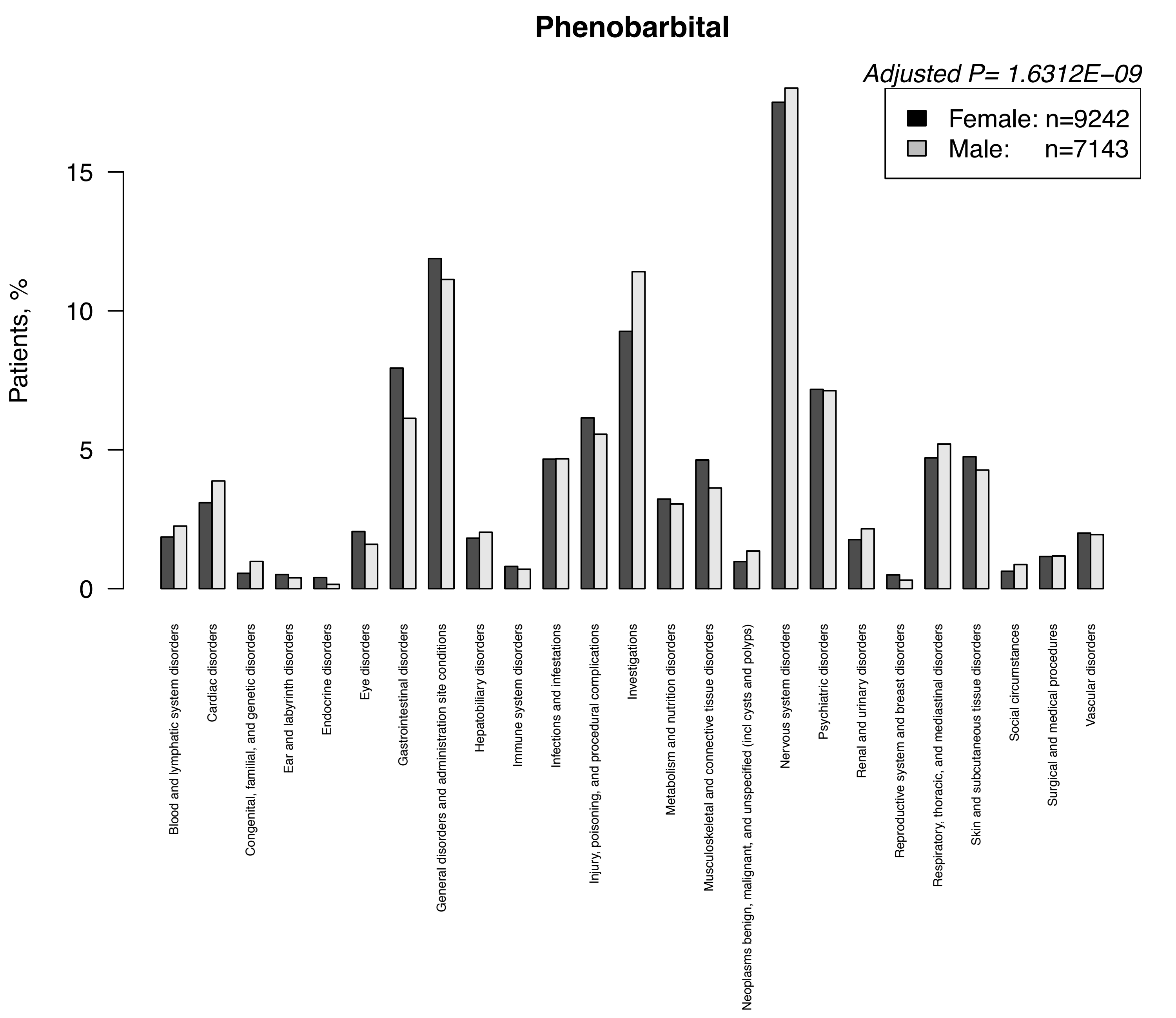
Phenobarbital
Adjusted P= 1.6312E−09
Female: n=9242
Male: n=7143
Patients, %
0
5
10
15
Blood and lymphatic system disorders
Cardiac disorders
Congenital, familial, and genetic disorders
Ear and labyrinth disorders
Endocrine disorders
Eye disorders
Gastrointestinal disorders
General disorders and administration site conditions
Hepatobiliary disorders
Immune system disorders
Infections and infestations
Injury, poisoning, and procedural complications
Investigations
Metabolism and nutrition disorders
Musculoskeletal and connective tissue disorders
Neoplasms benign, malignant, and unspecified (incl cysts and polyps)
Nervous system disorders
Psychiatric disorders
Renal and urinary disorders
Reproductive system and breast disorders
Respiratory, thoracic, and mediastinal disorders
Skin and subcutaneous tissue disorders
Social circumstances
Surgical and medical procedures
Vascular disorders

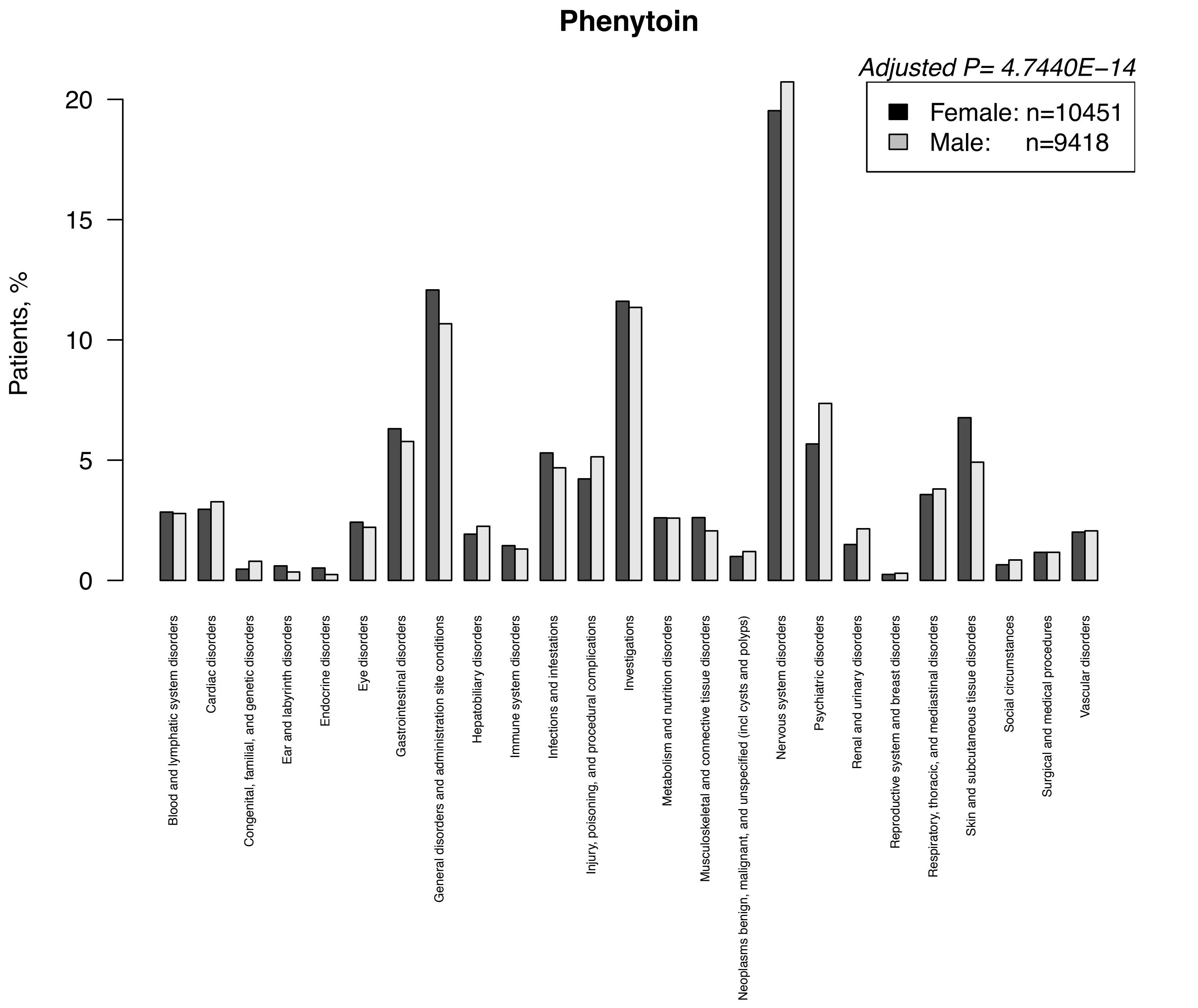

Phenytoin
Adjusted P= 4.7440E−14
Female: n=10451
Male: n=9418
Patients, %
0
5
10
15
20
Blood and lymphatic system disorders
Cardiac disorders
Congenital, familial, and genetic disorders
Ear and labyrinth disorders
Endocrine disorders
Eye disorders
Gastrointestinal disorders
General disorders and administration site conditions
Hepatobiliary disorders
Immune system disorders
Infections and infestations
Injury, poisoning, and procedural complications
Investigations
Metabolism and nutrition disorders
Musculoskeletal and connective tissue disorders
Neoplasms benign, malignant, and unspecified (incl cysts and polyps)
Nervous system disorders
Psychiatric disorders
Renal and urinary disorders
Reproductive system and breast disorders
Respiratory, thoracic, and mediastinal disorders
Skin and subcutaneous tissue disorders
Social circumstances
Surgical and medical procedures
Vascular disorders

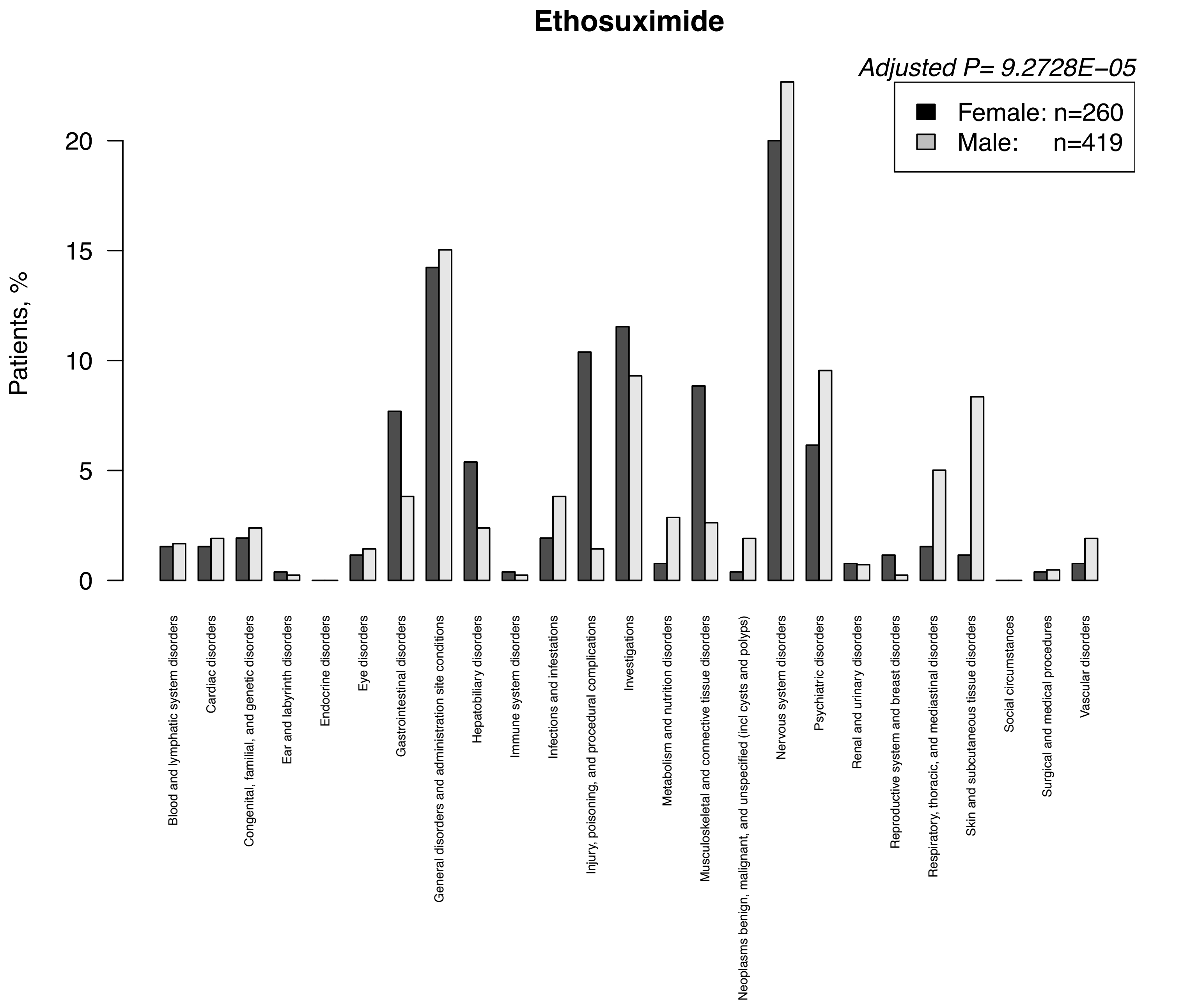
Ethosuximide
Adjusted P= 9.2728E−05
Female: n=260
Male: n=419
Patients, %
0
5
10
15
20
Blood and lymphatic system disorders
Cardiac disorders
Congenital, familial, and genetic disorders
Ear and labyrinth disorders
Endocrine disorders
Eye disorders
Gastrointestinal disorders
General disorders and administration site conditions
Hepatobiliary disorders
Immune system disorders
Infections and infestations
Injury, poisoning, and procedural complications
Investigations
Metabolism and nutrition disorders
Musculoskeletal and connective tissue disorders
Neoplasms benign, malignant, and unspecified (incl cysts and polyps)
Nervous system disorders
Psychiatric disorders
Renal and urinary disorders
Reproductive system and breast disorders
Respiratory, thoracic, and mediastinal disorders
Skin and subcutaneous tissue disorders
Social circumstances
Surgical and medical procedures
Vascular disorders

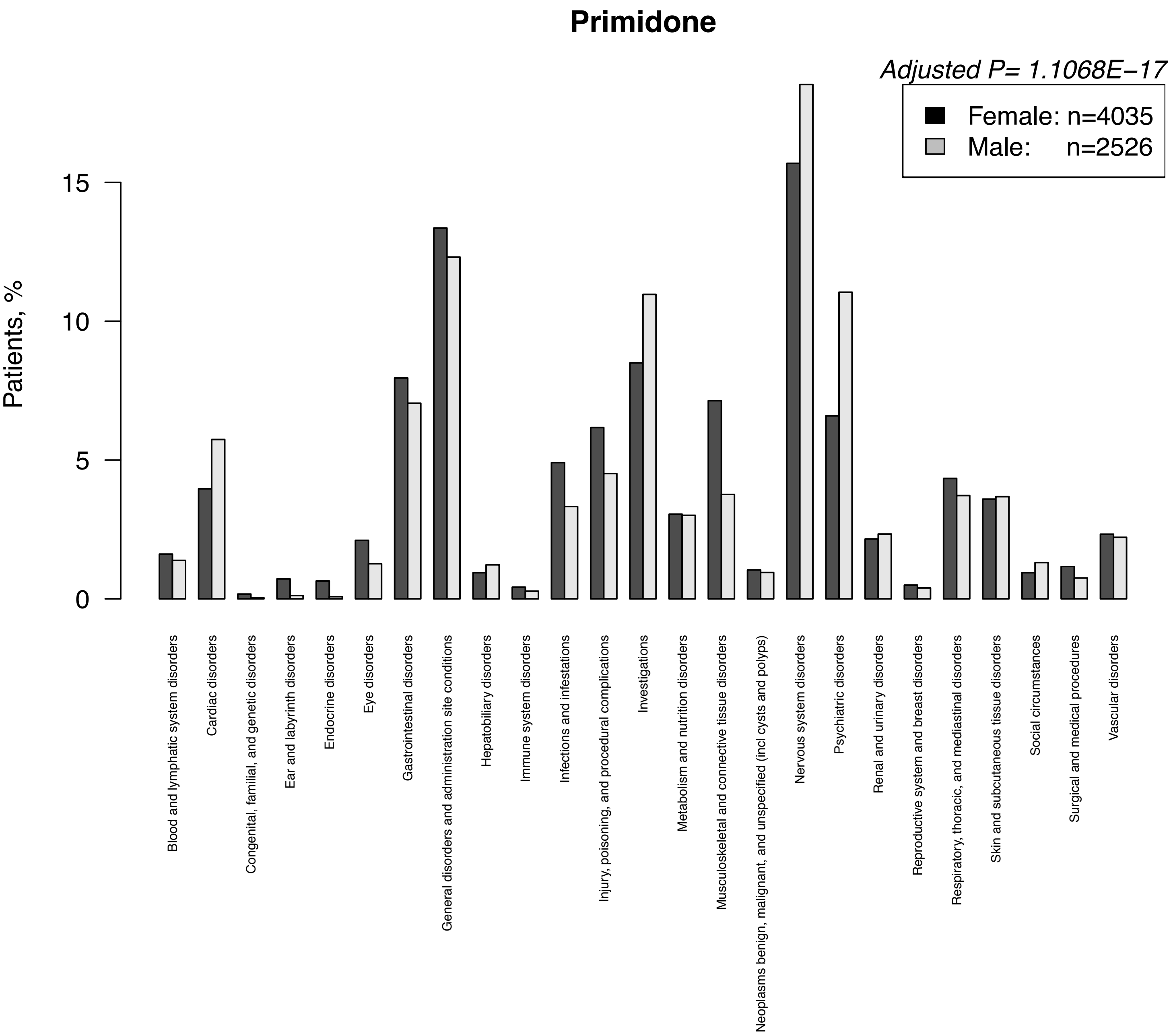

Primidone
Adjusted P= 1.1068E−17
Female: n=4035
Male: n=2526
Patients, %
0
5
10
15
Blood and lymphatic system disorders
Cardiac disorders
Congenital, familial, and genetic disorders
Ear and labyrinth disorders
Endocrine disorders
Eye disorders
Gastrointestinal disorders
General disorders and administration site conditions
Hepatobiliary disorders
Immune system disorders
Infections and infestations
Injury, poisoning, and procedural complications
Investigations
Metabolism and nutrition disorders
Musculoskeletal and connective tissue disorders
Neoplasms benign, malignant, and unspecified (incl cysts and polyps)
Nervous system disorders
Psychiatric disorders
Renal and urinary disorders
Reproductive system and breast disorders
Respiratory, thoracic, and mediastinal disorders
Skin and subcutaneous tissue disorders
Social circumstances
Surgical and medical procedures
Vascular disorders

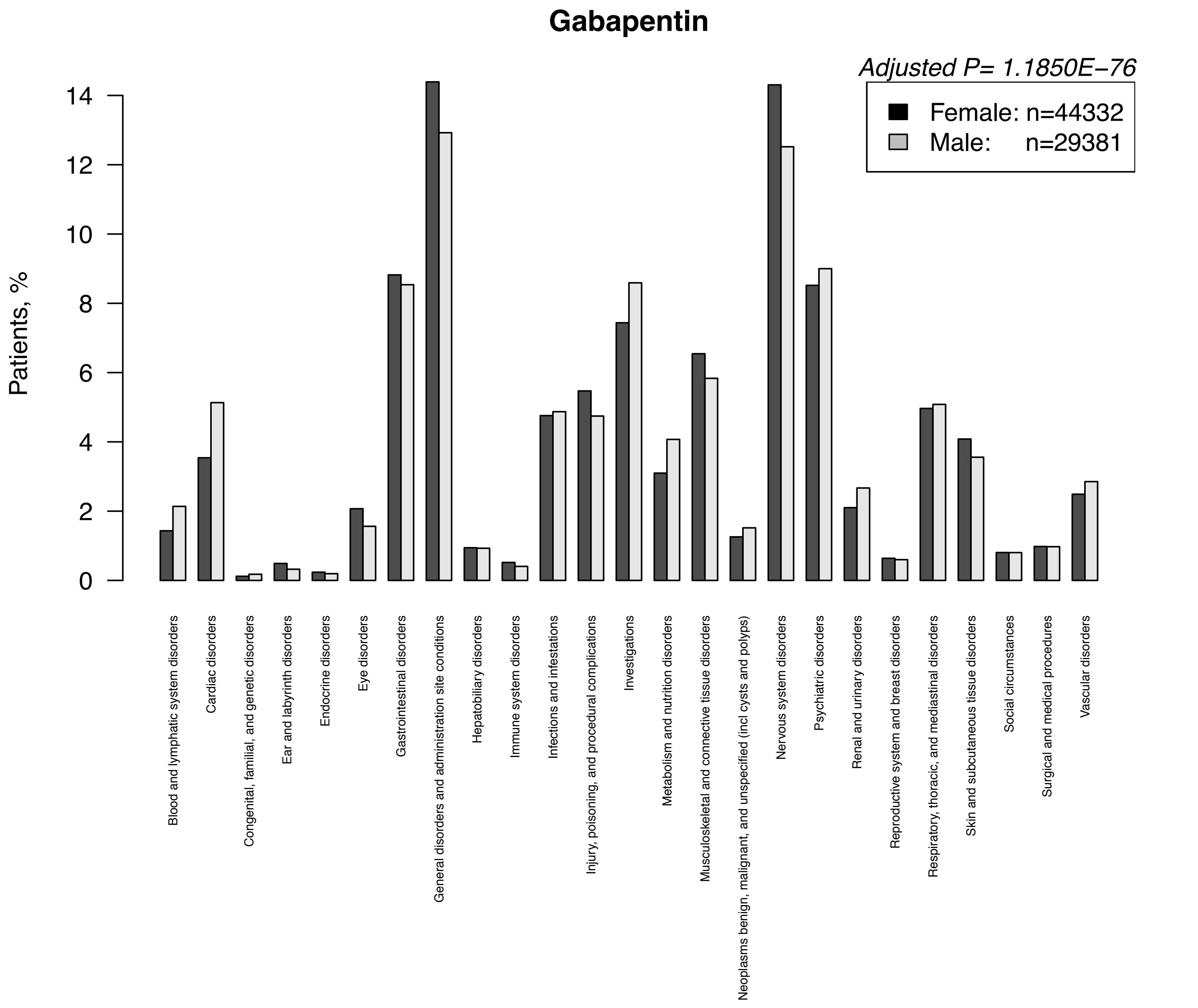

Gabapentin
Adjusted P= 1.1850E−76
Female: n=44332
Male: n=29381
Patients, %
0
2
4
6
8
10
12
14
Blood and lymphatic system disorders
Cardiac disorders
Congenital, familial, and genetic disorders
Ear and labyrinth disorders
Endocrine disorders
Eye disorders
Gastrointestinal disorders
General disorders and administration site conditions
Hepatobiliary disorders
Immune system disorders
Infections and infestations
Injury, poisoning, and procedural complications
Investigations
Metabolism and nutrition disorders
Musculoskeletal and connective tissue disorders
Neoplasms benign, malignant, and unspecified (incl cysts and polyps)
Nervous system disorders
Psychiatric disorders
Renal and urinary disorders
Reproductive system and breast disorders
Respiratory, thoracic, and mediastinal disorders
Skin and subcutaneous tissue disorders
Social circumstances
Surgical and medical procedures
Vascular disorders

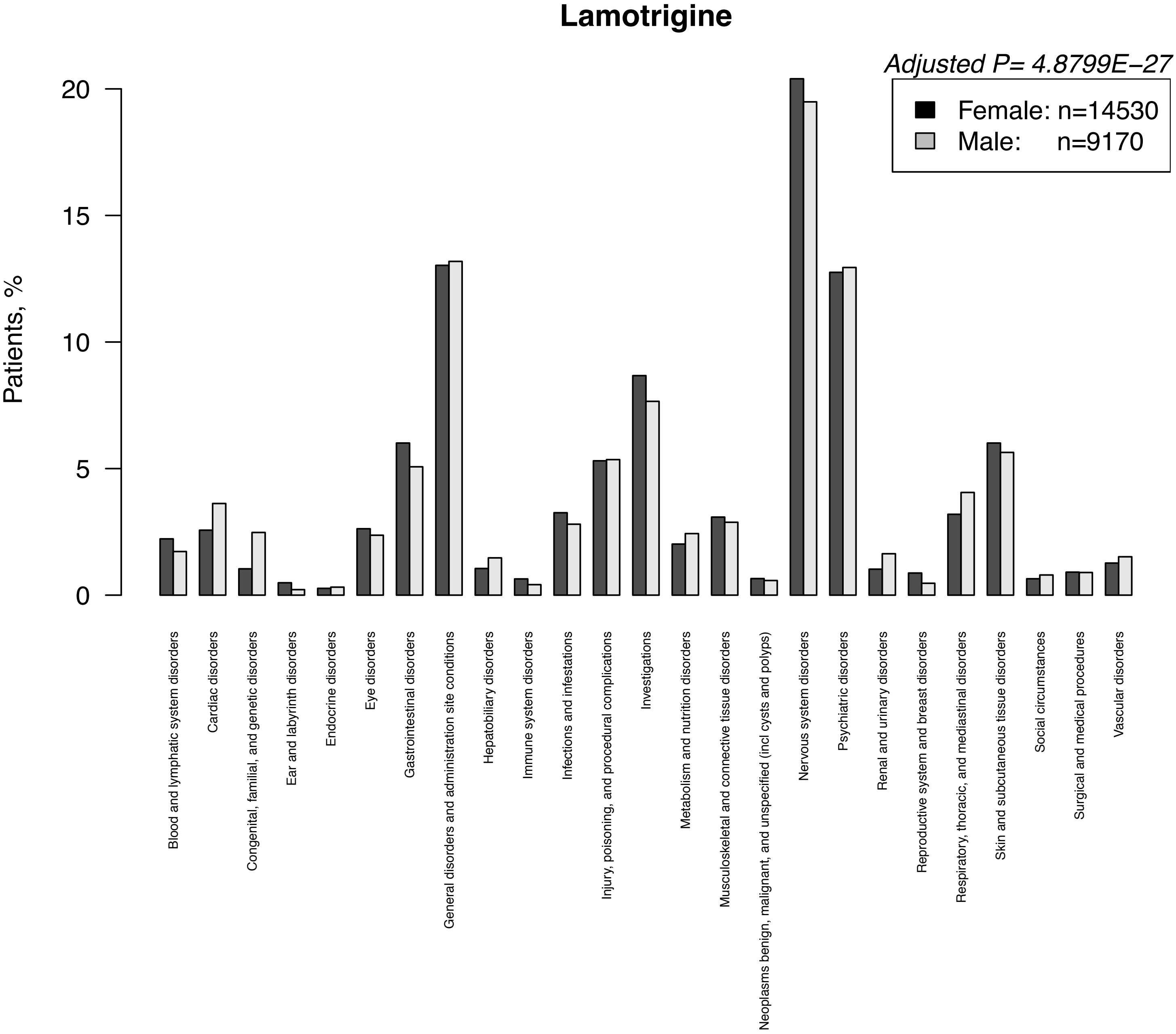
Lamotrigine
Adjusted P= 4.8799E−27
Female: n=14530
Male: n=9170
Patients, %
0
5
10
15
20
Blood and lymphatic system disorders
Cardiac disorders
Congenital, familial, and genetic disorders
Ear and labyrinth disorders
Endocrine disorders
Eye disorders
Gastrointestinal disorders
General disorders and administration site conditions
Hepatobiliary disorders
Immune system disorders
Infections and infestations
Injury, poisoning, and procedural complications
Investigations
Metabolism and nutrition disorders
Musculoskeletal and connective tissue disorders
Neoplasms benign, malignant, and unspecified (incl cysts and polyps)
Nervous system disorders
Psychiatric disorders
Renal and urinary disorders
Reproductive system and breast disorders
Respiratory, thoracic, and mediastinal disorders
Skin and subcutaneous tissue disorders
Social circumstances
Surgical and medical procedures
Vascular disorders

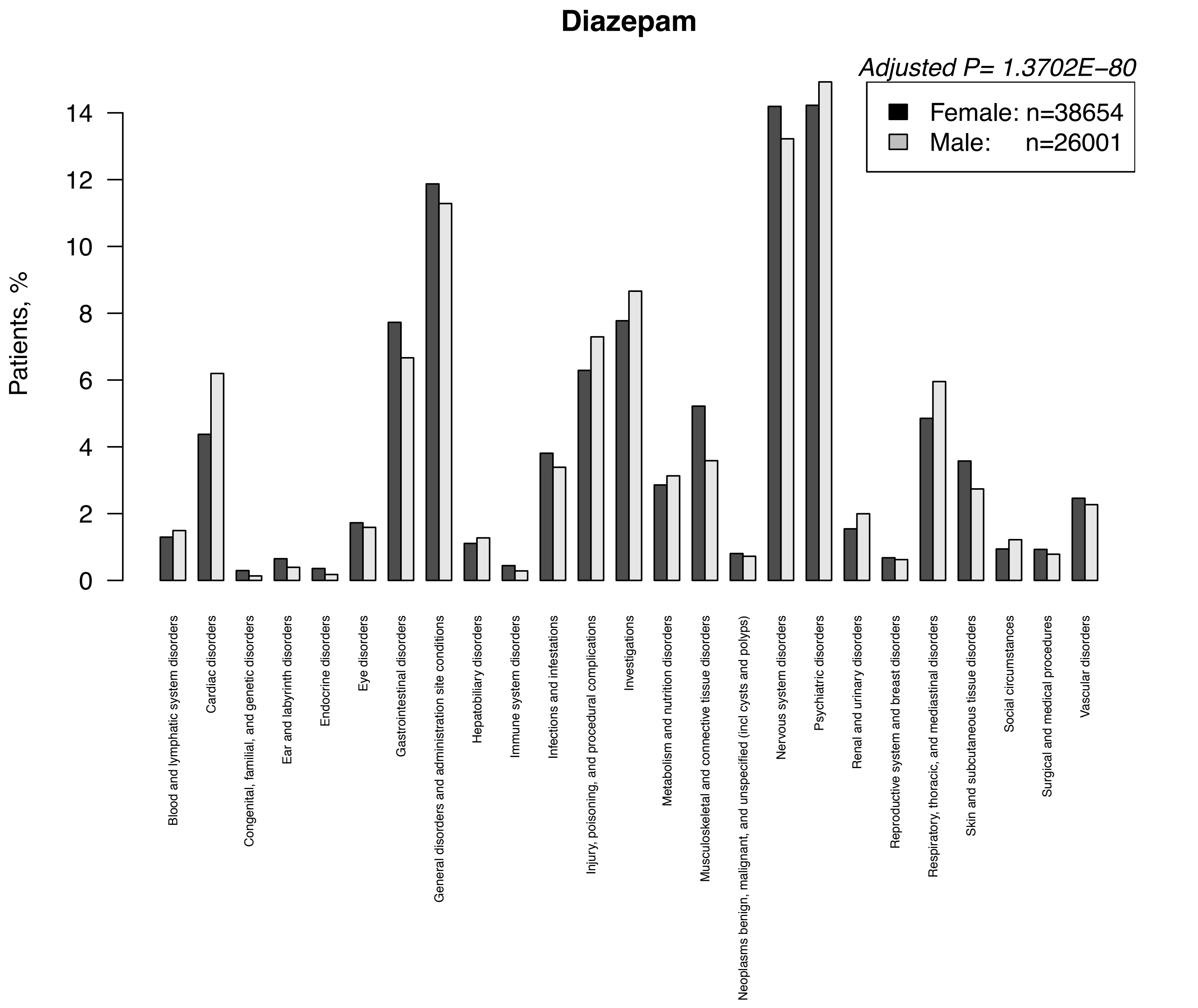
Diazepam
Adjusted P= 1.3702E−80
Female: n=38654
Male: n=26001
Patients, %
0
2
4
6
8
10
12
14
Blood and lymphatic system disorders
Cardiac disorders
Congenital, familial, and genetic disorders
Ear and labyrinth disorders
Endocrine disorders
Eye disorders
Gastrointestinal disorders
General disorders and administration site conditions
Hepatobiliary disorders
Immune system disorders
Infections and infestations
Injury, poisoning, and procedural complications
Investigations
Metabolism and nutrition disorders
Musculoskeletal and connective tissue disorders
Neoplasms benign, malignant, and unspecified (incl cysts and polyps)
Nervous system disorders
Psychiatric disorders
Renal and urinary disorders
Reproductive system and breast disorders
Respiratory, thoracic, and mediastinal disorders
Skin and subcutaneous tissue disorders
Social circumstances
Surgical and medical procedures
Vascular disorders

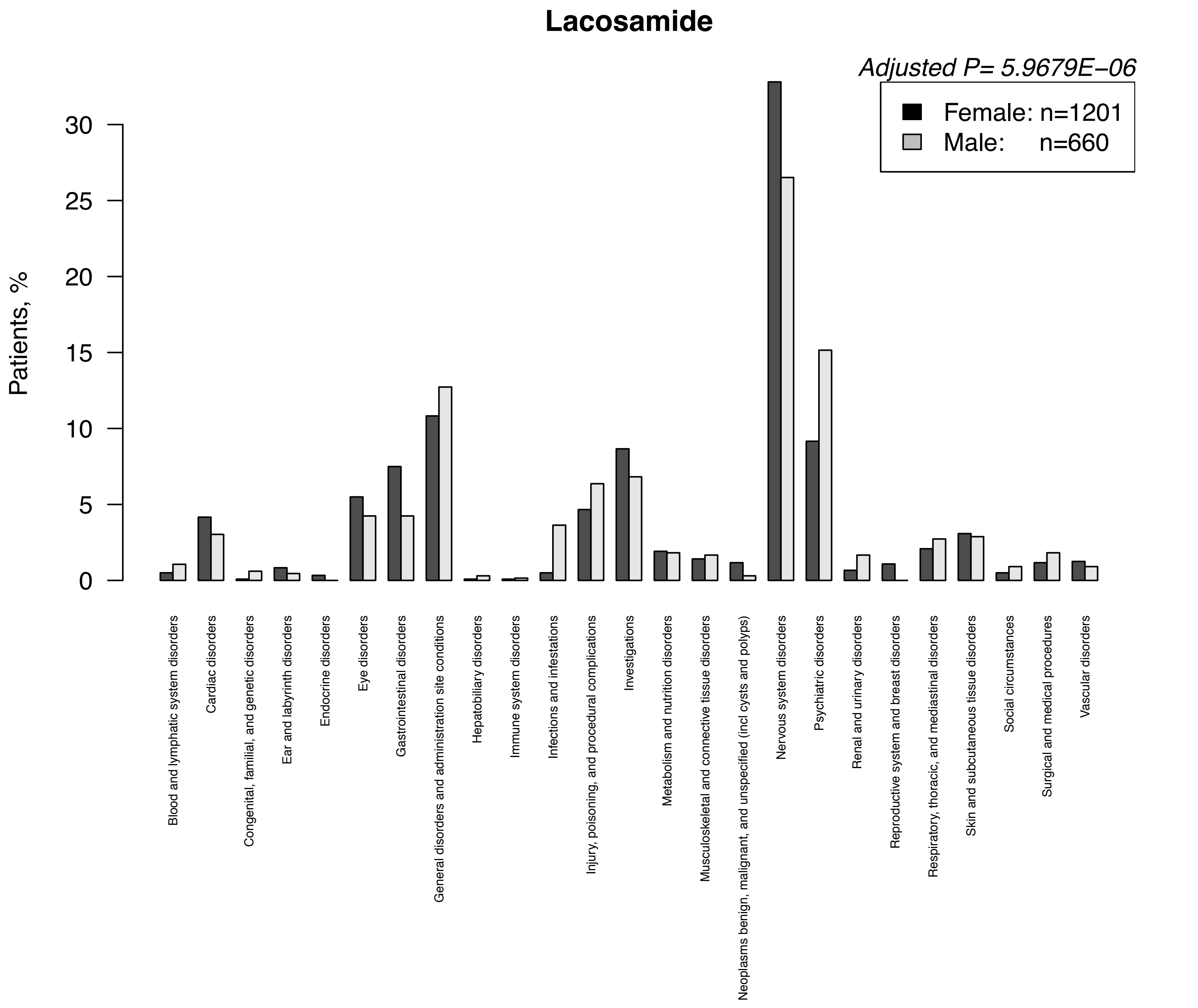

Lacosamide
Adjusted P= 5.9679E−06
Female: n=1201
Male: n=660
Patients, %
0
5
10
15
20
25
30
Blood and lymphatic system disorders
Cardiac disorders
Congenital, familial, and genetic disorders
Ear and labyrinth disorders
Endocrine disorders
Eye disorders
Gastrointestinal disorders
General disorders and administration site conditions
Hepatobiliary disorders
Immune system disorders
Infections and infestations
Injury, poisoning, and procedural complications
Investigations
Metabolism and nutrition disorders
Musculoskeletal and connective tissue disorders
Neoplasms benign, malignant, and unspecified (incl cysts and polyps)
Nervous system disorders
Psychiatric disorders
Renal and urinary disorders
Reproductive system and breast disorders
Respiratory, thoracic, and mediastinal disorders
Skin and subcutaneous tissue disorders
Social circumstances
Surgical and medical procedures
Vascular disorders

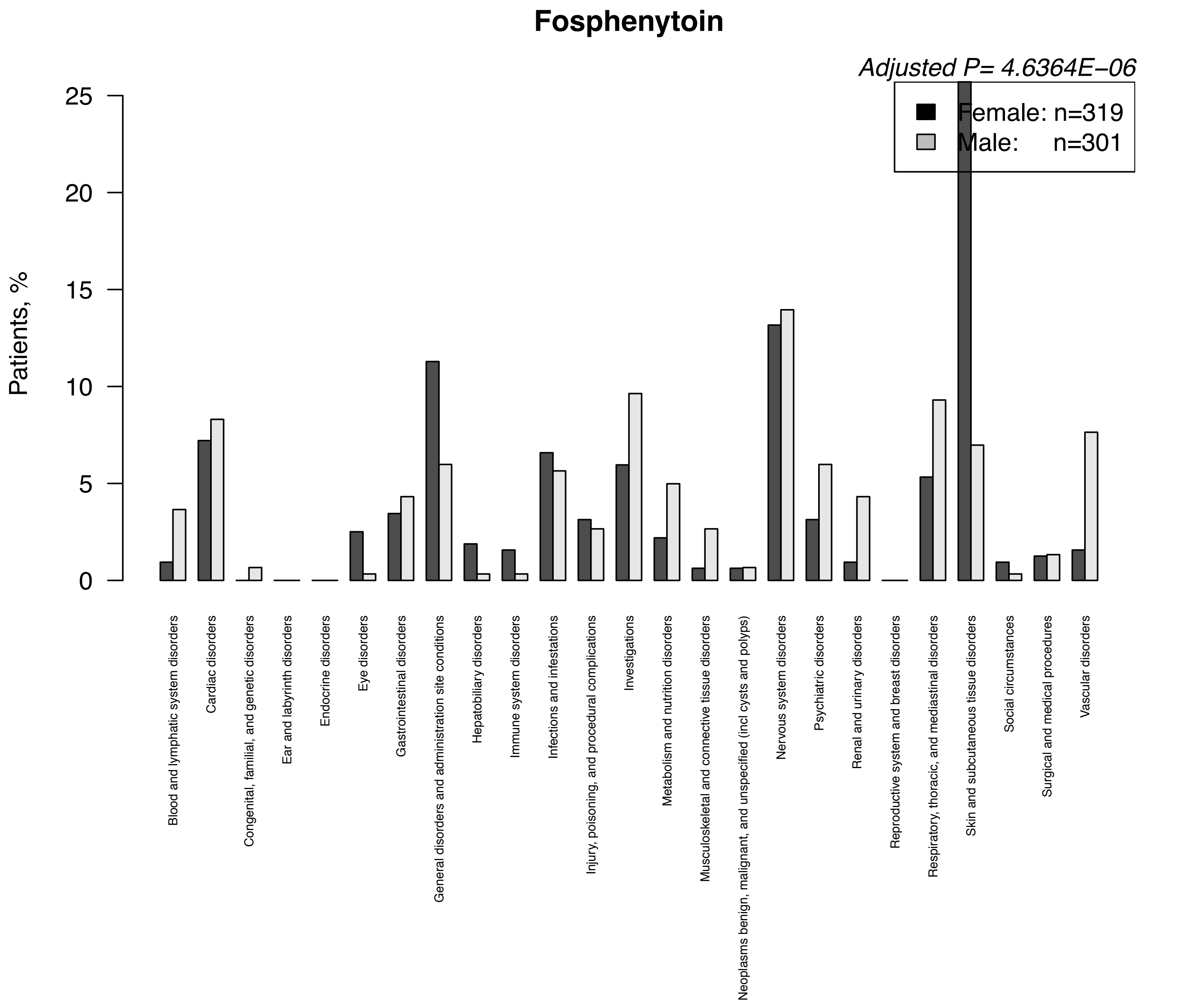

Fosphenytoin
Adjusted P= 4.6364E−06
Female: n=319
Male: n=301
Patients, %
25
20
15
10
5
0
Blood and lymphatic system disorders
Cardiac disorders
Congenital, familial, and genetic disorders
Ear and labyrinth disorders
Endocrine disorders
Eye disorders
Gastrointestinal disorders
General disorders and administration site conditions
Hepatobiliary disorders
Immune system disorders
Infections and infestations
Injury, poisoning, and procedural complications
Investigations
Metabolism and nutrition disorders
Musculoskeletal and connective tissue disorders
Neoplasms benign, malignant, and unspecified (incl cysts and polyps)
Nervous system disorders
Psychiatric disorders
Renal and urinary disorders
Reproductive system and breast disorders
Respiratory, thoracic, and mediastinal disorders
Skin and subcutaneous tissue disorders
Social circumstances
Surgical and medical procedures
Vascular disorders

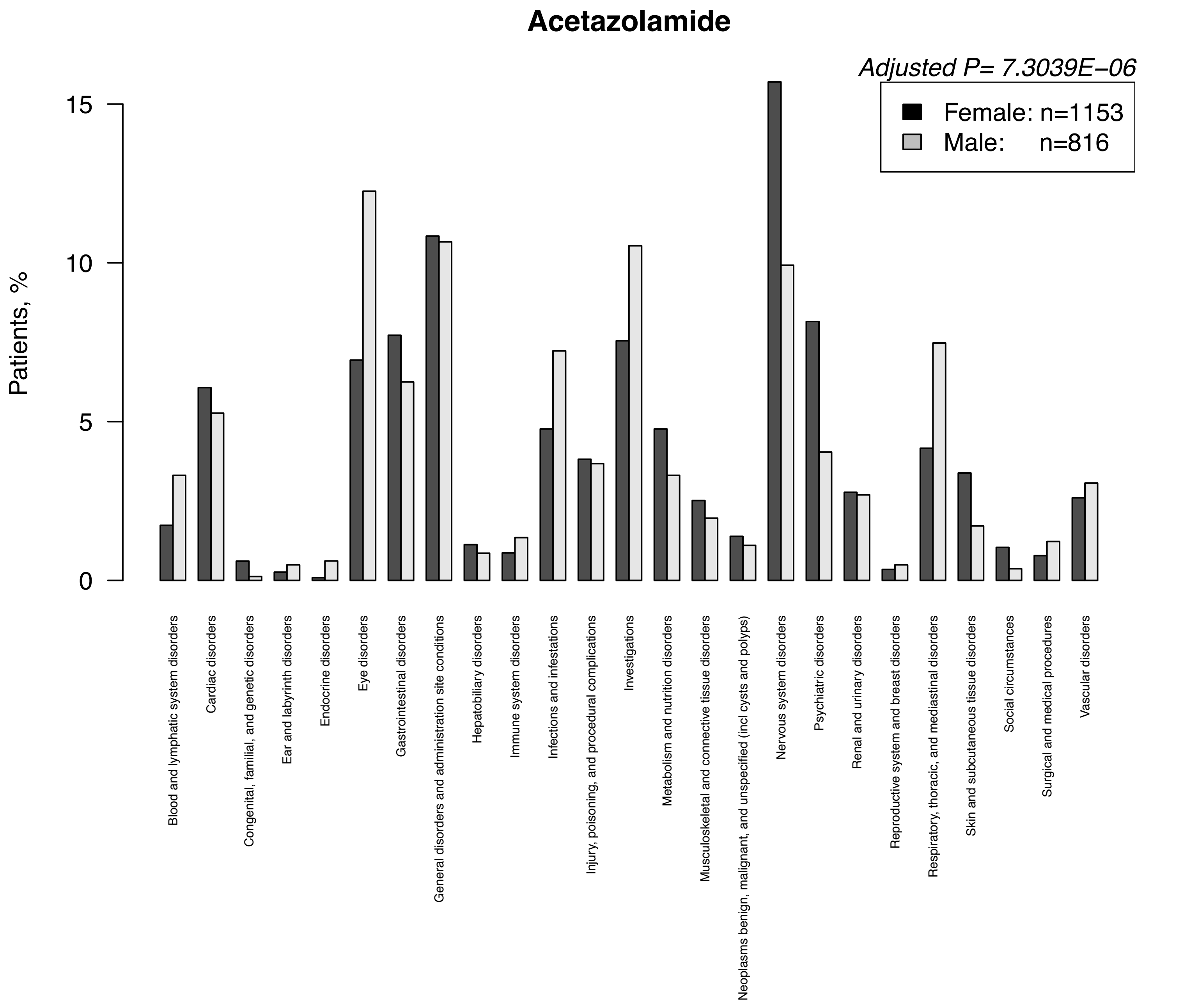
Acetazolamide
Adjusted P= 7.3039E−06
Female: n=1153
Male: n=816
Patients, %
15
10
5
0
Blood and lymphatic system disorders
Cardiac disorders
Congenital, familial, and genetic disorders
Ear and labyrinth disorders
Endocrine disorders
Eye disorders
Gastrointestinal disorders
General disorders and administration site conditions
Hepatobiliary disorders
Immune system disorders
Infections and infestations
Injury, poisoning, and procedural complications
Investigations
Metabolism and nutrition disorders
Musculoskeletal and connective tissue disorders
Neoplasms benign, malignant, and unspecified (incl cysts and polyps)
Nervous system disorders
Psychiatric disorders
Renal and urinary disorders
Reproductive system and breast disorders
Respiratory, thoracic, and mediastinal disorders
Skin and subcutaneous tissue disorders
Social circumstances
Surgical and medical procedures
Vascular disorders

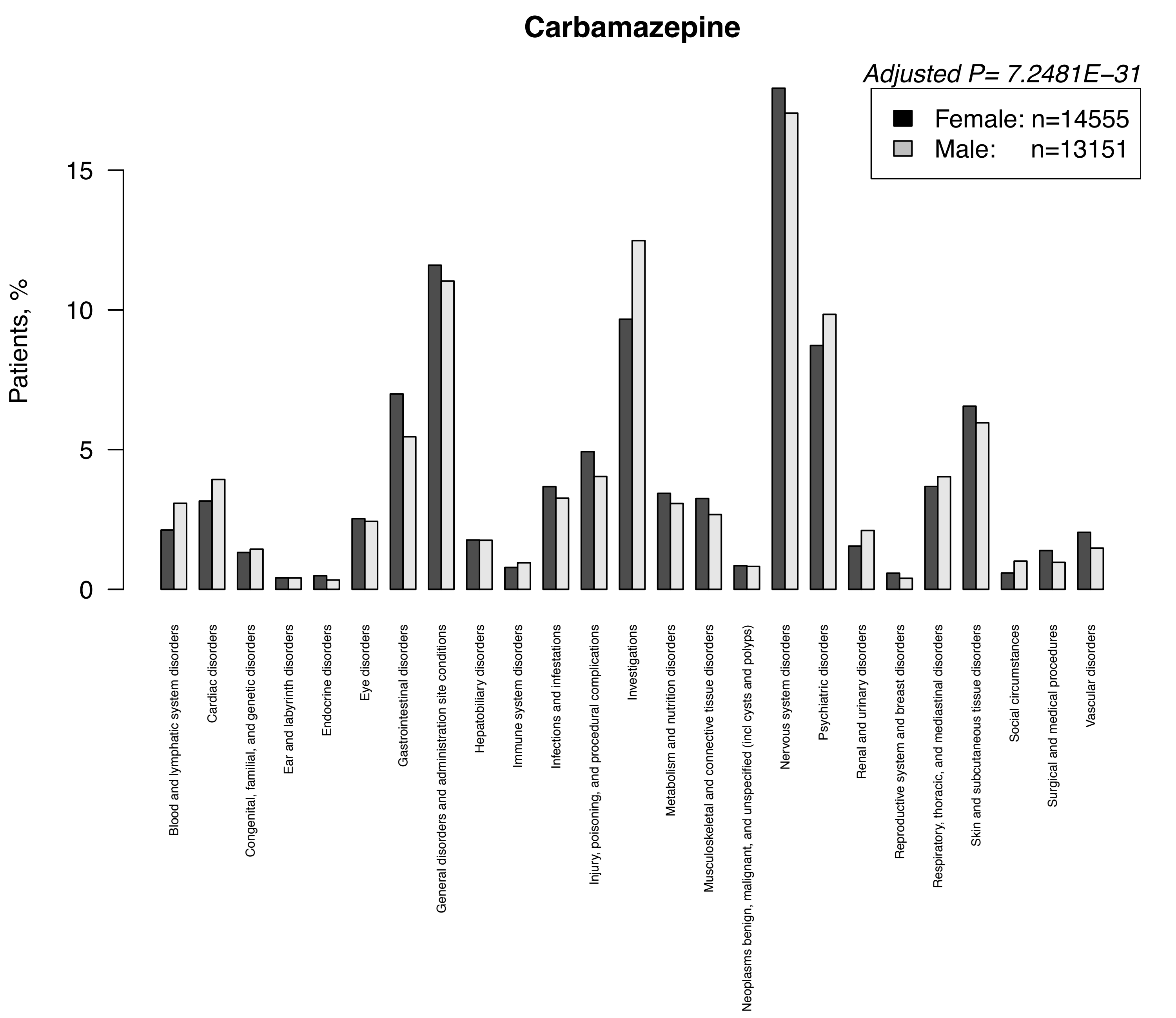
Carbamazepine
Adjusted P= 7.2481E−31
Female: n=14555
Male: n=13151
Patients, %
0
5
10
15
Blood and lymphatic system disorders
Cardiac disorders
Congenital, familial, and genetic disorders
Ear and labyrinth disorders
Endocrine disorders
Eye disorders
Gastrointestinal disorders
General disorders and administration site conditions
Hepatobiliary disorders
Immune system disorders
Infections and infestations
Injury, poisoning, and procedural complications
Investigations
Metabolism and nutrition disorders
Musculoskeletal and connective tissue disorders
Neoplasms benign, malignant, and unspecified (incl cysts and polyps)
Nervous system disorders
Psychiatric disorders
Renal and urinary disorders
Reproductive system and breast disorders
Respiratory, thoracic, and mediastinal disorders
Skin and subcutaneous tissue disorders
Social circumstances
Surgical and medical procedures
Vascular disorders

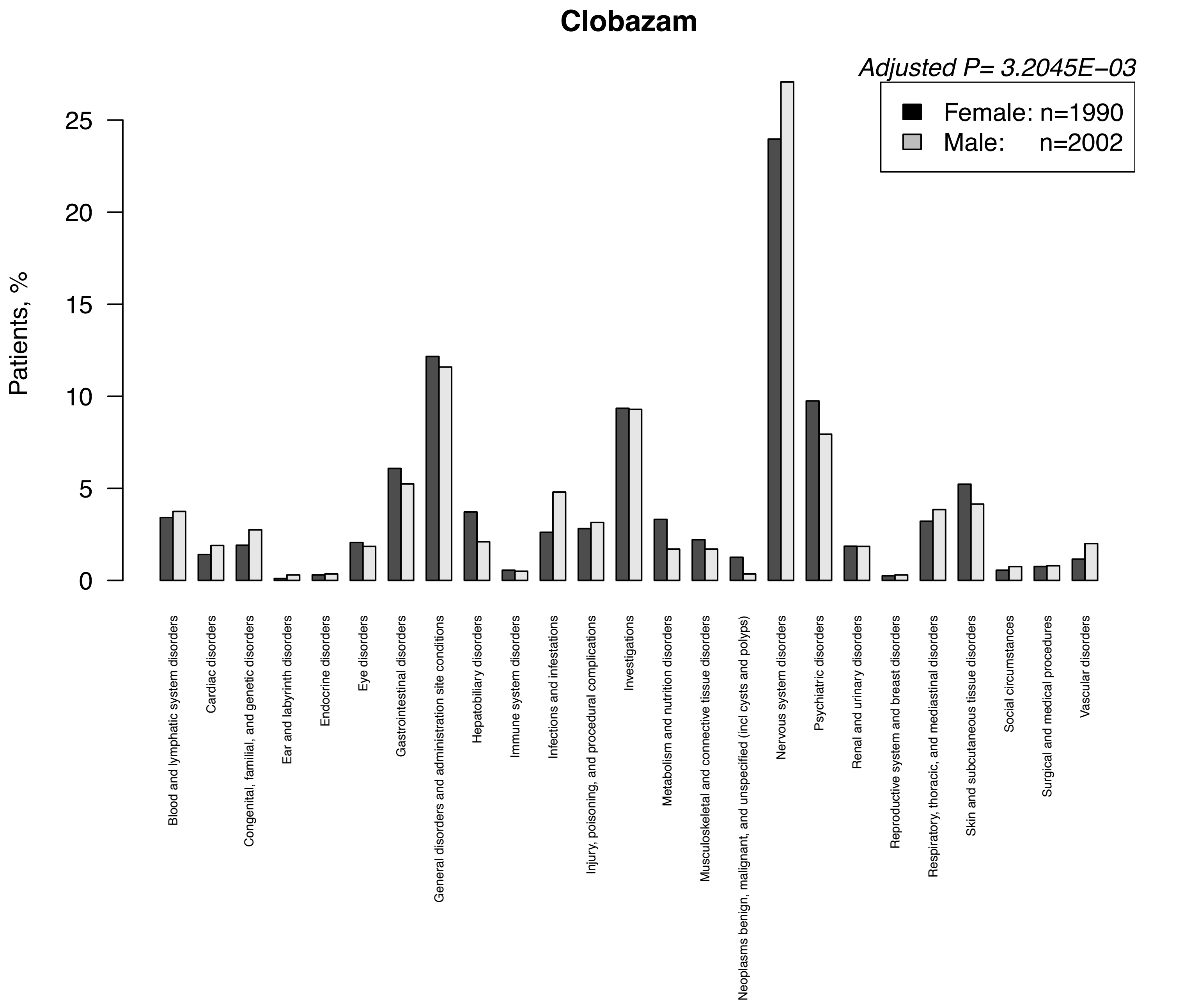
Clobazam
Adjusted P= 3.2045E−03
Female: n=1990
Male: n=2002
Patients, %
0
5
10
15
20
25
Blood and lymphatic system disorders
Cardiac disorders
Congenital, familial, and genetic disorders
Ear and labyrinth disorders
Endocrine disorders
Eye disorders
Gastrointestinal disorders
General disorders and administration site conditions
Hepatobiliary disorders
Immune system disorders
Infections and infestations
Injury, poisoning, and procedural complications
Investigations
Metabolism and nutrition disorders
Musculoskeletal and connective tissue disorders
Neoplasms benign, malignant, and unspecified (incl cysts and polyps)
Nervous system disorders
Psychiatric disorders
Renal and urinary disorders
Reproductive system and breast disorders
Respiratory, thoracic, and mediastinal disorders
Skin and subcutaneous tissue disorders
Social circumstances
Surgical and medical procedures
Vascular disorders

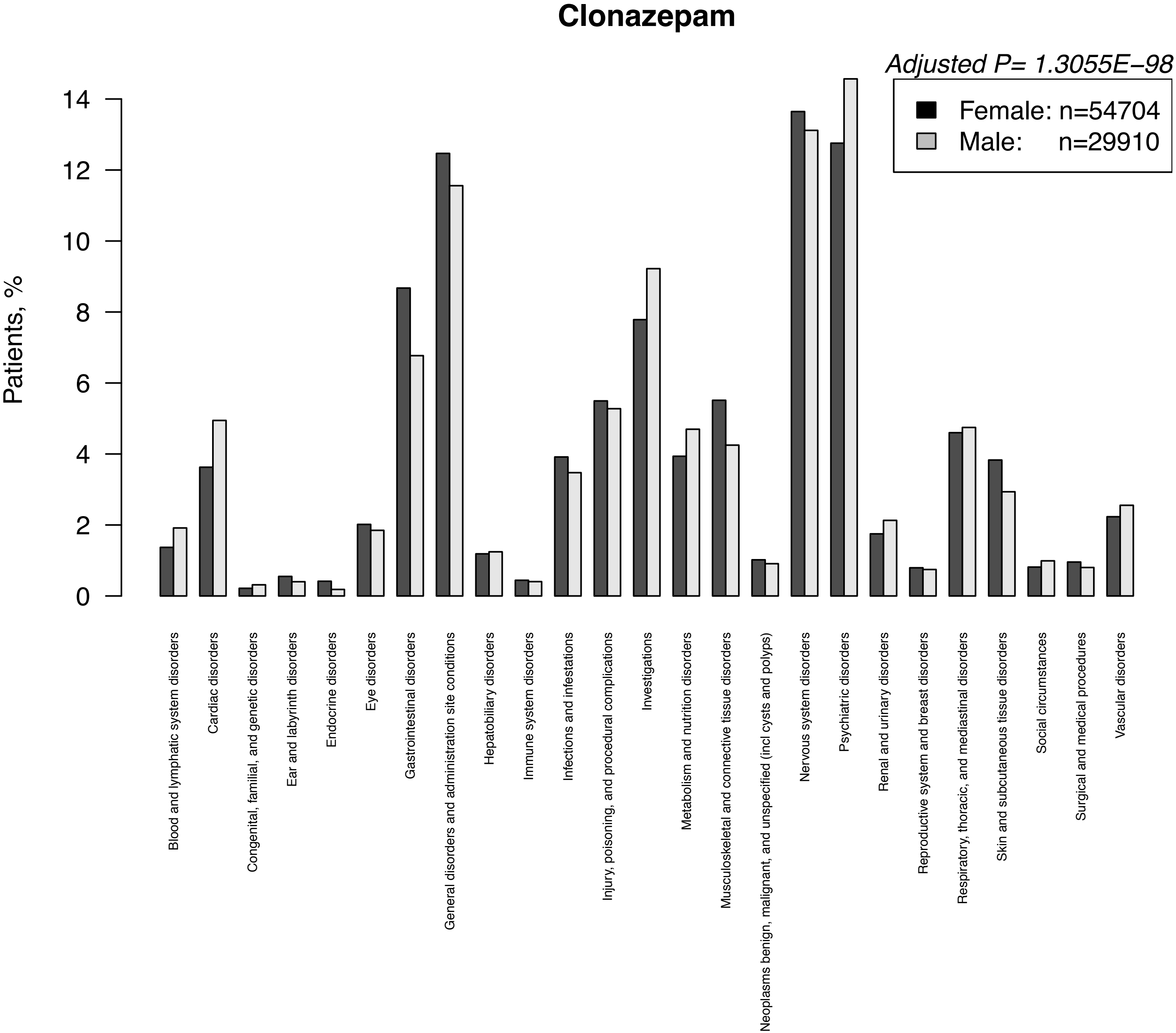

Clonazepam
Adjusted P= 1.3055E−98
Female: n=54704
Male: n=29910
Patients, %
0
2
4
6
8
10
12
14
Blood and lymphatic system disorders
Cardiac disorders
Congenital, familial, and genetic disorders
Ear and labyrinth disorders
Endocrine disorders
Eye disorders
Gastrointestinal disorders
General disorders and administration site conditions
Hepatobiliary disorders
Immune system disorders
Infections and infestations
Injury, poisoning, and procedural complications
Investigations
Metabolism and nutrition disorders
Musculoskeletal and connective tissue disorders
Neoplasms benign, malignant, and unspecified (incl cysts and polyps)
Nervous system disorders
Psychiatric disorders
Renal and urinary disorders
Reproductive system and breast disorders
Respiratory, thoracic, and mediastinal disorders
Skin and subcutaneous tissue disorders
Social circumstances
Surgical and medical procedures
Vascular disorders

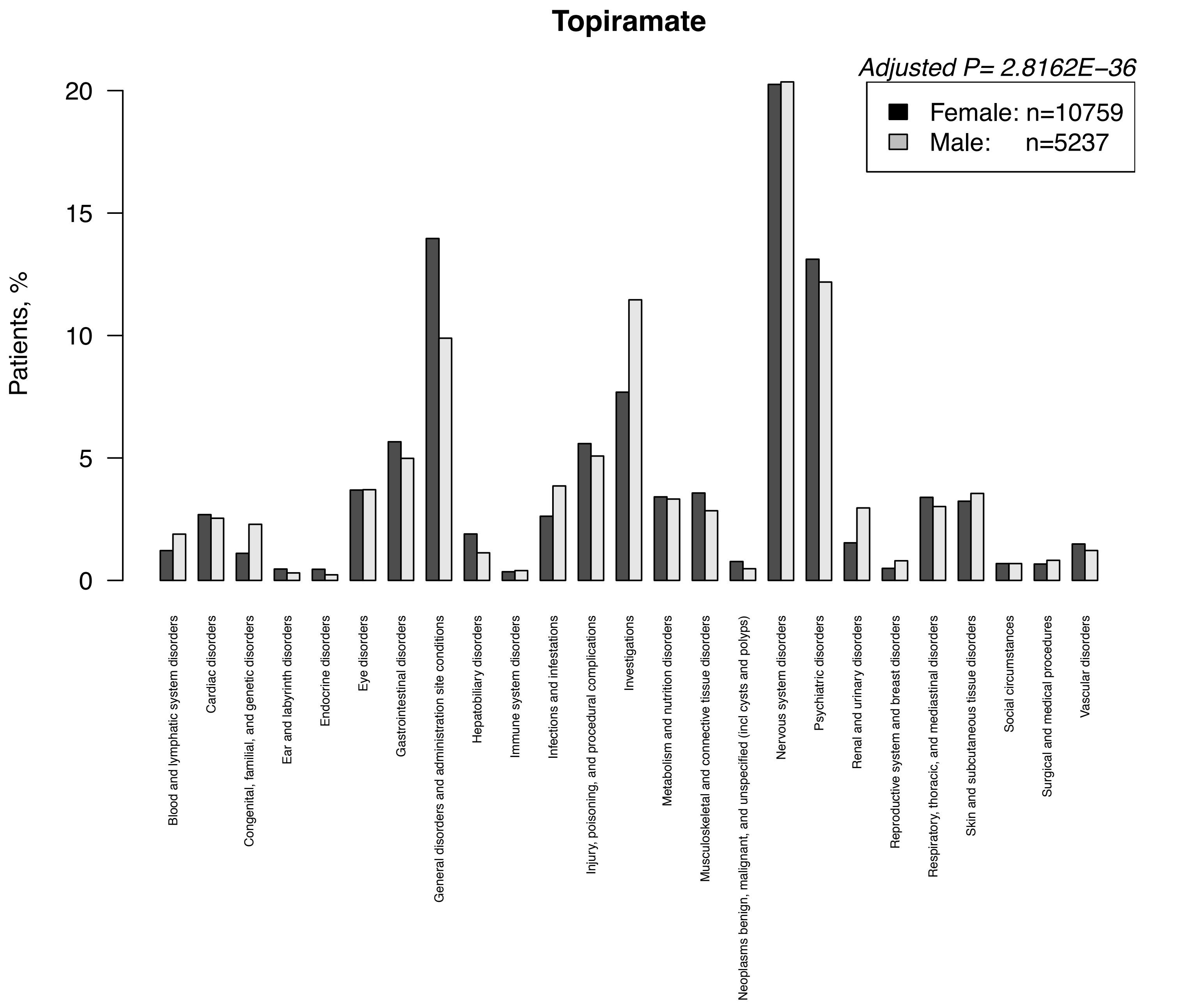
Topiramate
Adjusted P= 2.8162E−36
Female: n=10759
Male: n=5237
Patients, %
0
5
10
15
20
Blood and lymphatic system disorders
Cardiac disorders
Congenital, familial, and genetic disorders
Ear and labyrinth disorders
Endocrine disorders
Eye disorders
Gastrointestinal disorders
General disorders and administration site conditions
Hepatobiliary disorders
Immune system disorders
Infections and infestations
Injury, poisoning, and procedural complications
Investigations
Metabolism and nutrition disorders
Musculoskeletal and connective tissue disorders
Neoplasms benign, malignant, and unspecified (incl cysts and polyps)
Nervous system disorders
Psychiatric disorders
Renal and urinary disorders
Reproductive system and breast disorders
Respiratory, thoracic, and mediastinal disorders
Skin and subcutaneous tissue disorders
Social circumstances
Surgical and medical procedures
Vascular disorders

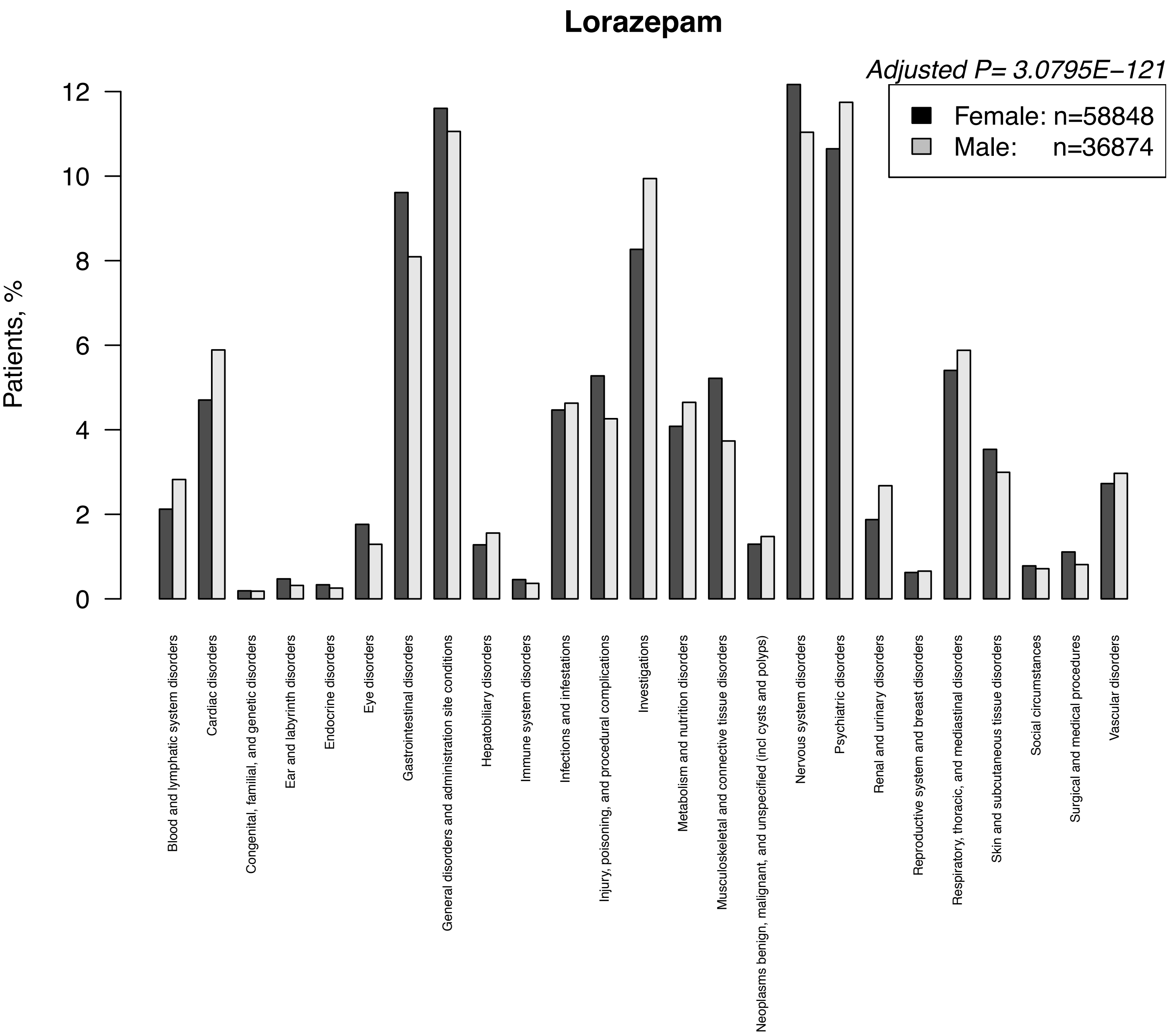
Lorazepam
Adjusted P= 3.0795E−121
Female: n=58848
Male: n=36874
Patients, %
0
2
4
6
8
10
12
Blood and lymphatic system disorders
Cardiac disorders
Congenital, familial, and genetic disorders
Ear and labyrinth disorders
Endocrine disorders
Eye disorders
Gastrointestinal disorders
General disorders and administration site conditions
Hepatobiliary disorders
Immune system disorders
Infections and infestations
Injury, poisoning, and procedural complications
Investigations
Metabolism and nutrition disorders
Musculoskeletal and connective tissue disorders
Neoplasms benign, malignant, and unspecified (incl cysts and polyps)
Nervous system disorders
Psychiatric disorders
Renal and urinary disorders
Reproductive system and breast disorders
Respiratory, thoracic, and mediastinal disorders
Skin and subcutaneous tissue disorders
Social circumstances
Surgical and medical procedures
Vascular disorders

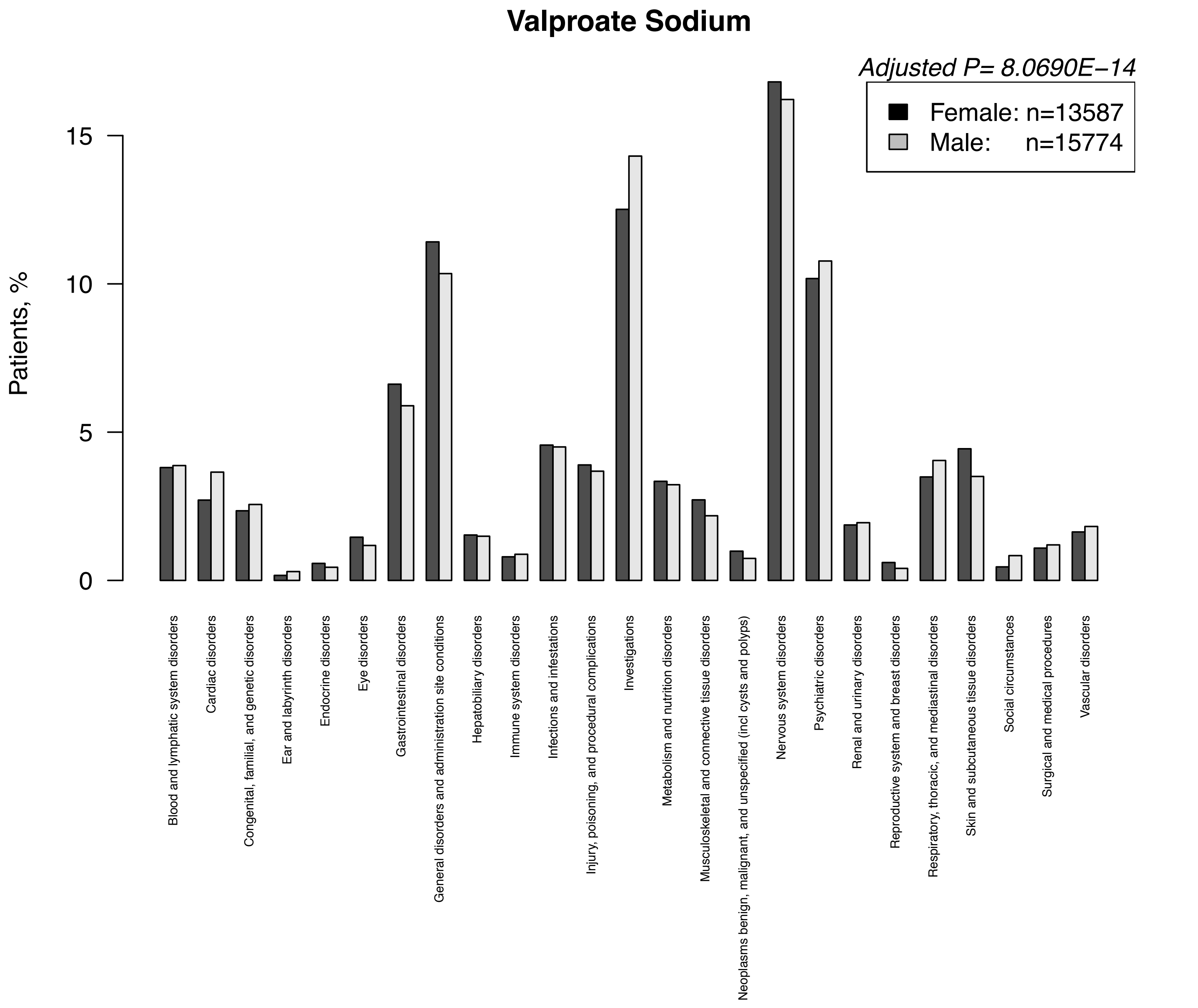
Valproate Sodium
Adjusted P= 8.0690E−14
Female: n=13587
Male: n=15774
Patients, %
0
5
10
15
Blood and lymphatic system disorders
Cardiac disorders
Congenital, familial, and genetic disorders
Ear and labyrinth disorders
Endocrine disorders
Eye disorders
Gastrointestinal disorders
General disorders and administration site conditions
Hepatobiliary disorders
Immune system disorders
Infections and infestations
Injury, poisoning, and procedural complications
Investigations
Metabolism and nutrition disorders
Musculoskeletal and connective tissue disorders
Neoplasms benign, malignant, and unspecified (incl cysts and polyps)
Nervous system disorders
Psychiatric disorders
Renal and urinary disorders
Reproductive system and breast disorders
Respiratory, thoracic, and mediastinal disorders
Skin and subcutaneous tissue disorders
Social circumstances
Surgical and medical procedures
Vascular disorders

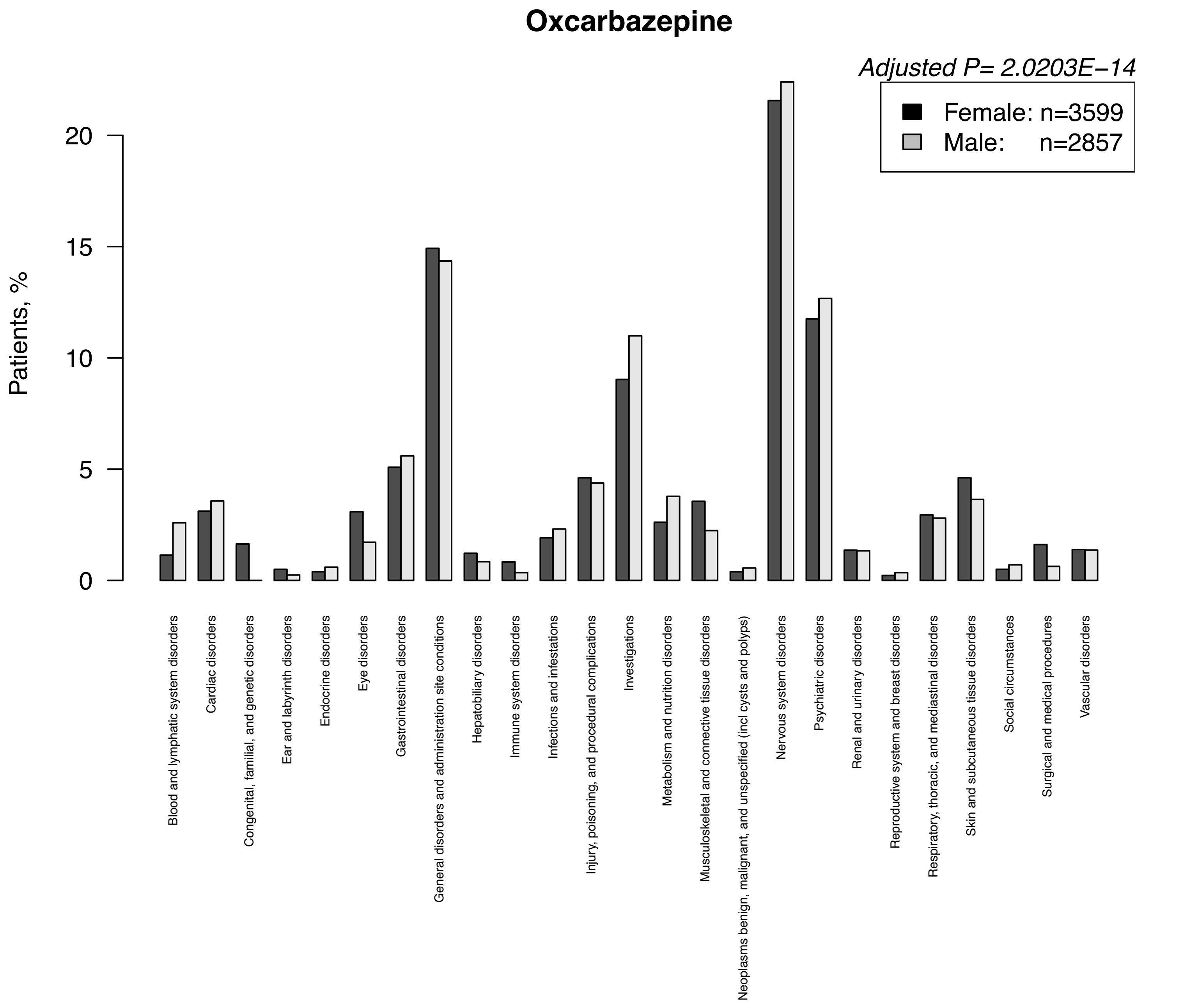

Oxcarbazepine
Adjusted P= 2.0203E−14
Female: n=3599
Male: n=2857
Patients, %
0
5
10
15
20
Blood and lymphatic system disorders
Cardiac disorders
Congenital, familial, and genetic disorders
Ear and labyrinth disorders
Endocrine disorders
Eye disorders
Gastrointestinal disorders
General disorders and administration site conditions
Hepatobiliary disorders
Immune system disorders
Infections and infestations
Injury, poisoning, and procedural complications
Investigations
Metabolism and nutrition disorders
Musculoskeletal and connective tissue disorders
Neoplasms benign, malignant, and unspecified (incl cysts and polyps)
Nervous system disorders
Psychiatric disorders
Renal and urinary disorders
Reproductive system and breast disorders
Respiratory, thoracic, and mediastinal disorders
Skin and subcutaneous tissue disorders
Social circumstances
Surgical and medical procedures
Vascular disorders

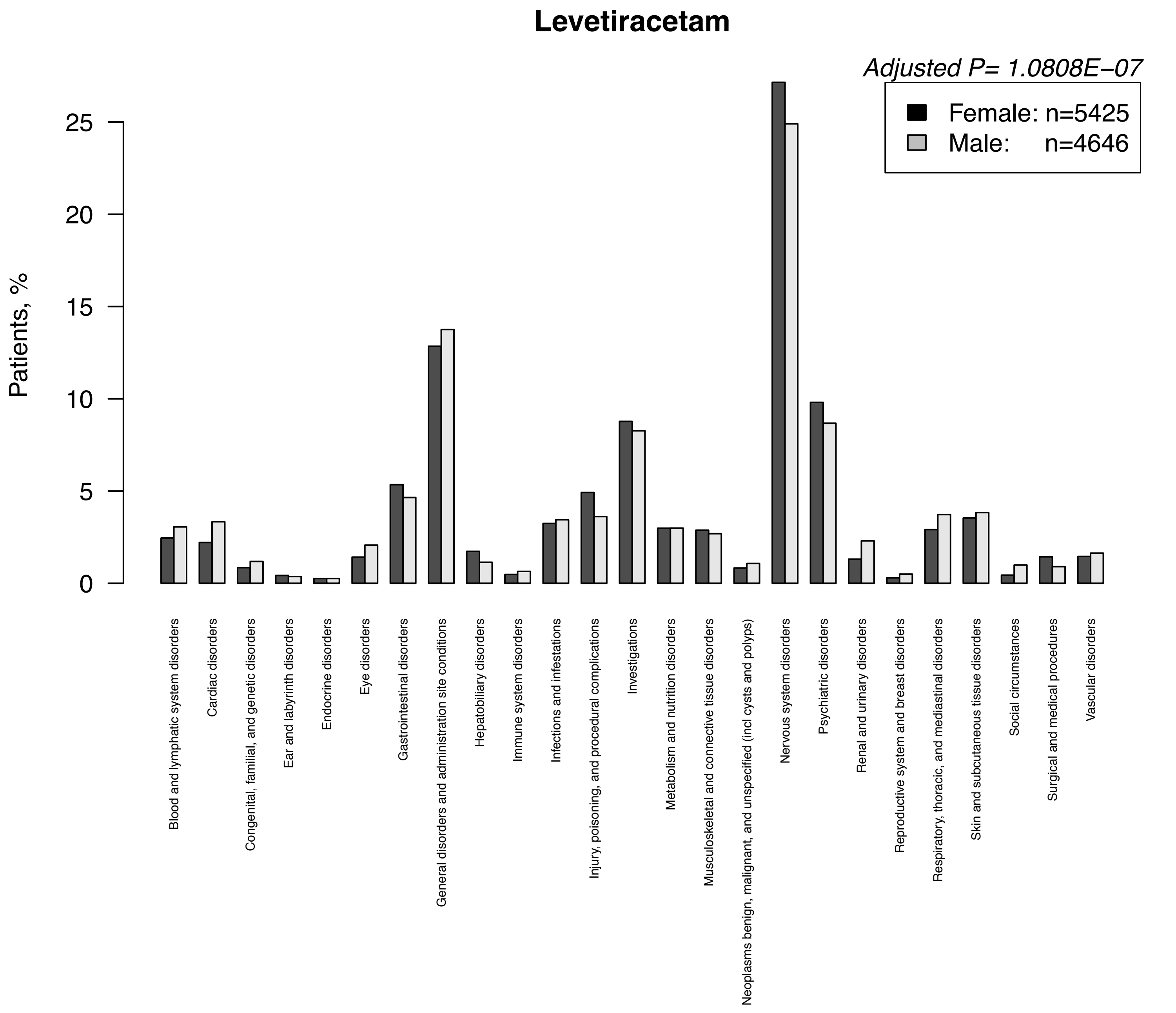
Levetiracetam
Adjusted P= 1.0808E−07
Female: n=5425
Male: n=4646
Patients, %
0
5
10
15
20
25
Blood and lymphatic system disorders
Cardiac disorders
Congenital, familial, and genetic disorders
Ear and labyrinth disorders
Endocrine disorders
Eye disorders
Gastrointestinal disorders
General disorders and administration site conditions
Hepatobiliary disorders
Immune system disorders
Infections and infestations
Injury, poisoning, and procedural complications
Investigations
Metabolism and nutrition disorders
Musculoskeletal and connective tissue disorders
Neoplasms benign, malignant, and unspecified (incl cysts and polyps)
Nervous system disorders
Psychiatric disorders
Renal and urinary disorders
Reproductive system and breast disorders
Respiratory, thoracic, and mediastinal disorders
Skin and subcutaneous tissue disorders
Social circumstances
Surgical and medical procedures
Vascular disorders

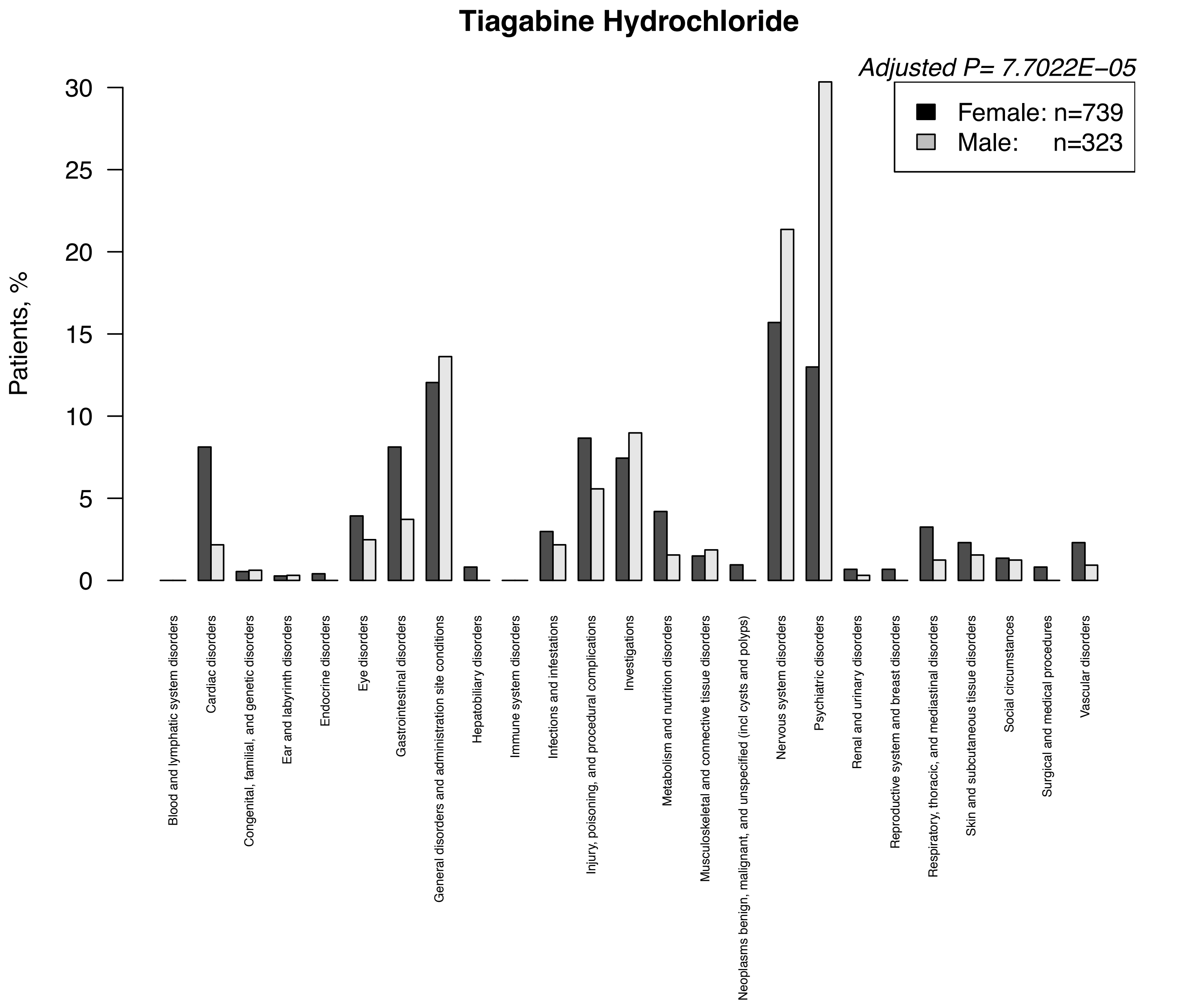
Tiagabine Hydrochloride
Adjusted P= 7.7022E−05
Female: n=739
Male: n=323
Patients, %
0
5
10
15
20
25
30
Blood and lymphatic system disorders
Cardiac disorders
Congenital, familial, and genetic disorders
Ear and labyrinth disorders
Endocrine disorders
Eye disorders
Gastrointestinal disorders
General disorders and administration site conditions
Hepatobiliary disorders
Immune system disorders
Infections and infestations
Injury, poisoning, and procedural complications
Investigations
Metabolism and nutrition disorders
Musculoskeletal and connective tissue disorders
Neoplasms benign, malignant, and unspecified (incl cysts and polyps)
Nervous system disorders
Psychiatric disorders
Renal and urinary disorders
Reproductive system and breast disorders
Respiratory, thoracic, and mediastinal disorders
Skin and subcutaneous tissue disorders
Social circumstances
Surgical and medical procedures
Vascular disorders

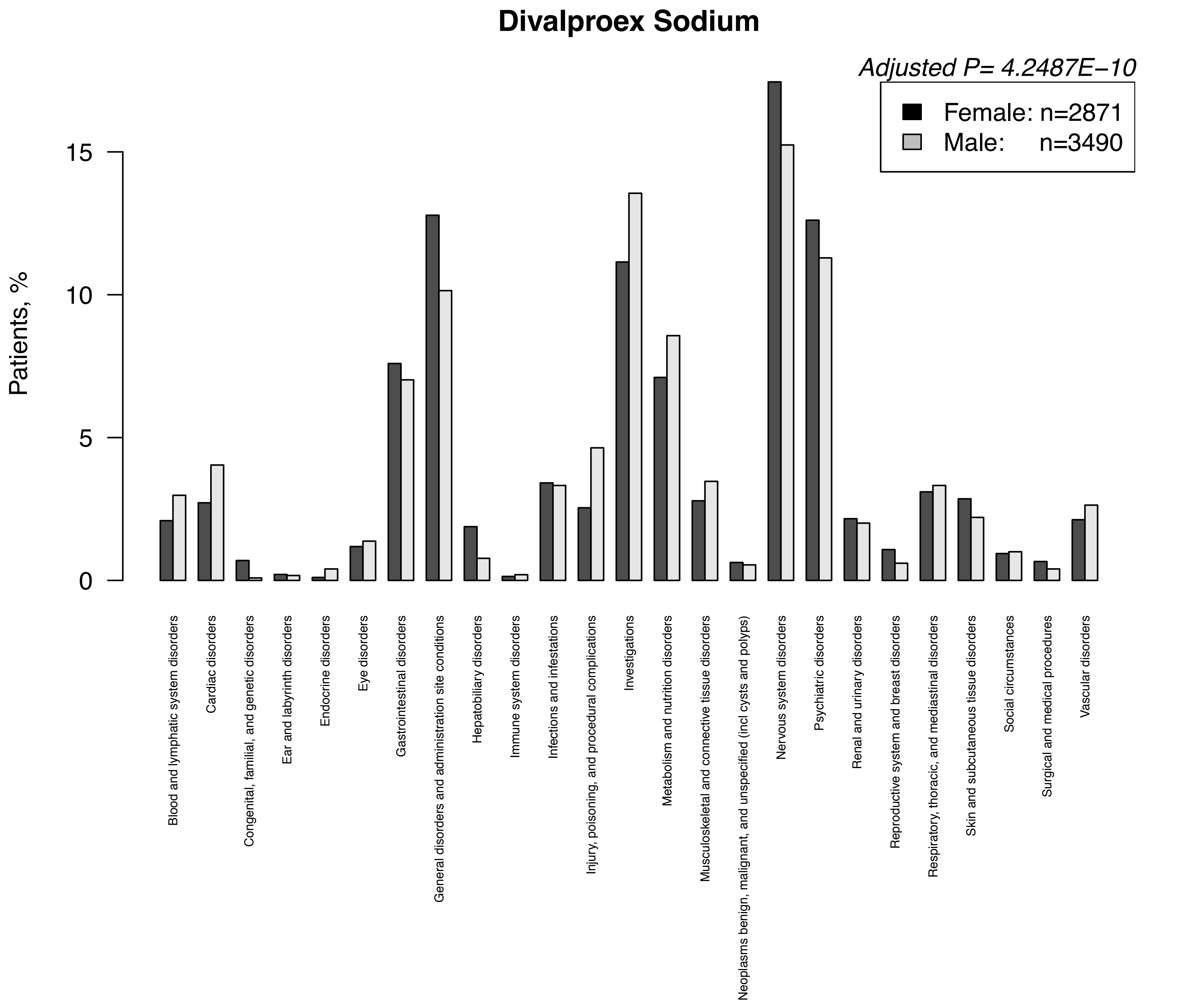

Divalproex Sodium
Adjusted P= 4.2487E−10
Female: n=2871
Male: n=3490
Patients, %
0
5
10
15
Blood and lymphatic system disorders
Cardiac disorders
Congenital, familial, and genetic disorders
Ear and labyrinth disorders
Endocrine disorders
Eye disorders
Gastrointestinal disorders
General disorders and administration site conditions
Hepatobiliary disorders
Immune system disorders
Infections and infestations
Injury, poisoning, and procedural complications
Investigations
Metabolism and nutrition disorders
Musculoskeletal and connective tissue disorders
Neoplasms benign, malignant, and unspecified (incl cysts and polyps)
Nervous system disorders
Psychiatric disorders
Renal and urinary disorders
Reproductive system and breast disorders
Respiratory, thoracic, and mediastinal disorders
Skin and subcutaneous tissue disorders
Social circumstances
Surgical and medical procedures
Vascular disorders

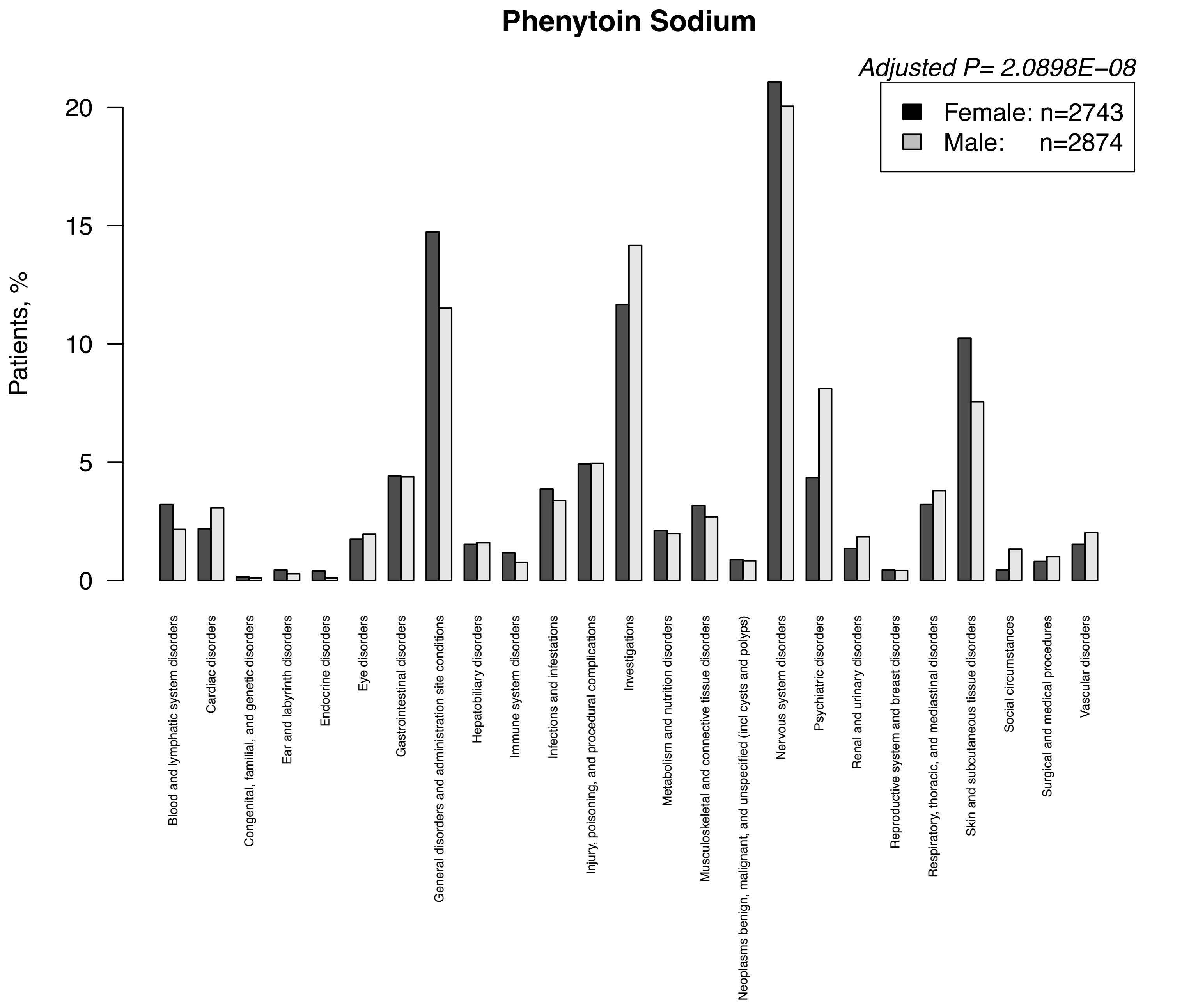

Phenytoin Sodium
Adjusted P= 2.0898E−08
Female: n=2743
Male: n=2874
Patients, %
0
5
10
15
20
Blood and lymphatic system disorders
Cardiac disorders
Congenital, familial, and genetic disorders
Ear and labyrinth disorders
Endocrine disorders
Eye disorders
Gastrointestinal disorders
General disorders and administration site conditions
Hepatobiliary disorders
Immune system disorders
Infections and infestations
Injury, poisoning, and procedural complications
Investigations
Metabolism and nutrition disorders
Musculoskeletal and connective tissue disorders
Neoplasms benign, malignant, and unspecified (incl cysts and polyps)
Nervous system disorders
Psychiatric disorders
Renal and urinary disorders
Reproductive system and breast disorders
Respiratory, thoracic, and mediastinal disorders
Skin and subcutaneous tissue disorders
Social circumstances
Surgical and medical procedures
Vascular disorders

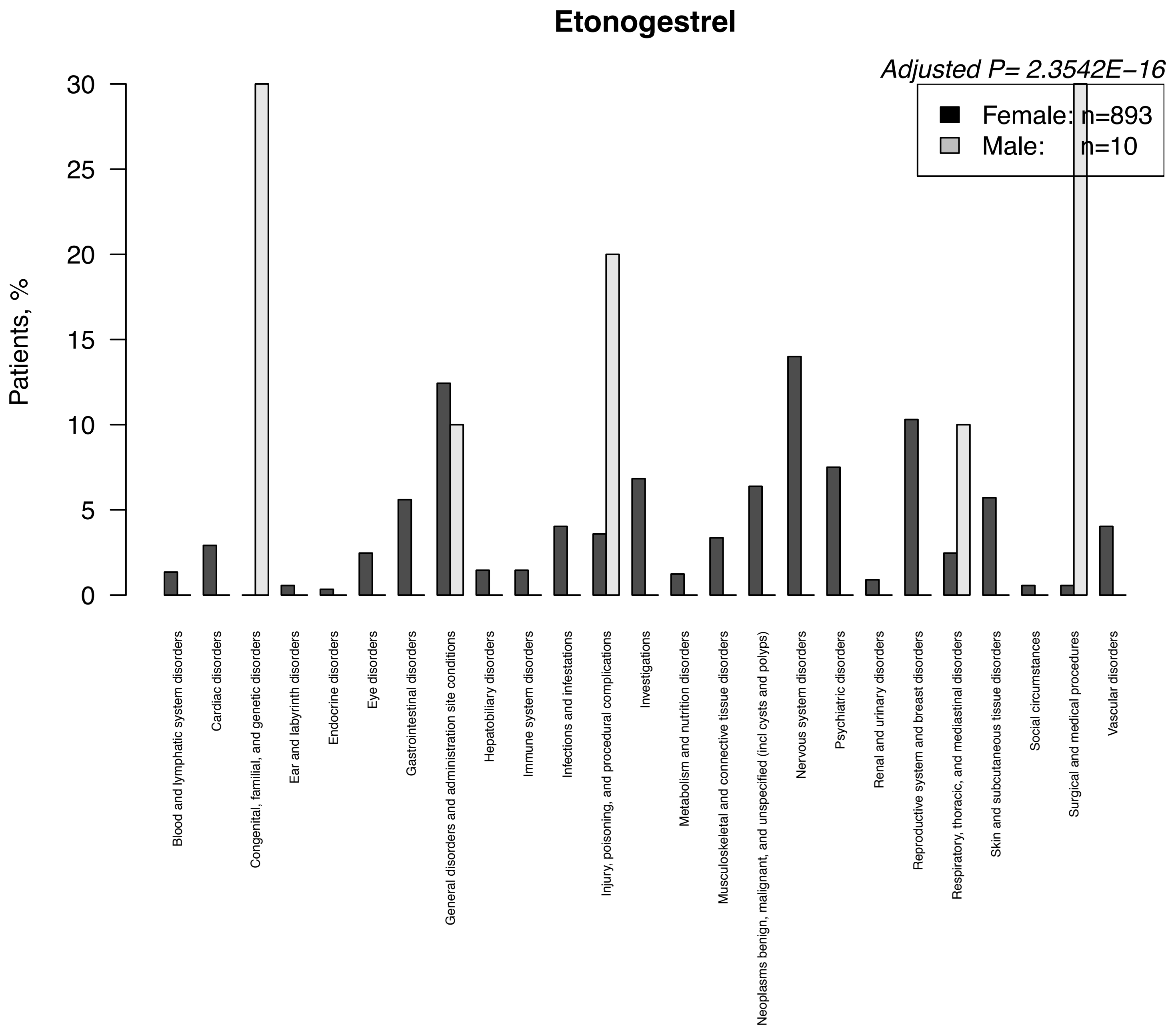

Etonogestrel
Adjusted P= 2.3542E−16
Female: n=893
Male: n=10
Patients, %
0
5
10
15
20
25
30
Blood and lymphatic system disorders
Cardiac disorders
Congenital, familial, and genetic disorders
Ear and labyrinth disorders
Endocrine disorders
Eye disorders
Gastrointestinal disorders
General disorders and administration site conditions
Hepatobiliary disorders
Immune system disorders
Infections and infestations
Injury, poisoning, and procedural complications
Investigations
Metabolism and nutrition disorders
Musculoskeletal and connective tissue disorders
Neoplasms benign, malignant, and unspecified (incl cysts and polyps)
Nervous system disorders
Psychiatric disorders
Renal and urinary disorders
Reproductive system and breast disorders
Respiratory, thoracic, and mediastinal disorders
Skin and subcutaneous tissue disorders
Social circumstances
Surgical and medical procedures
Vascular disorders

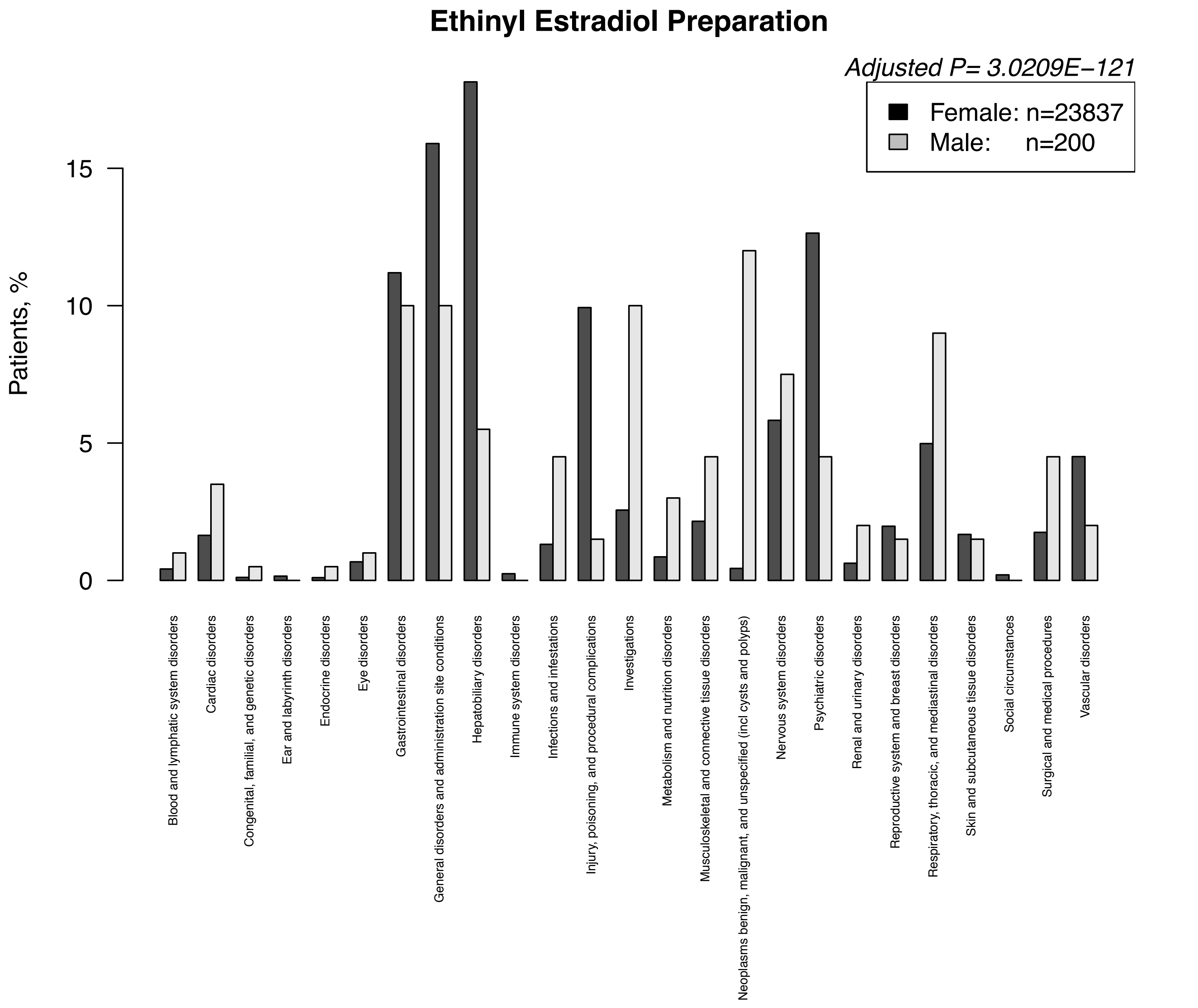
Ethinyl Estradiol Preparation
Adjusted P= 3.0209E−121
Female: n=23837
Male: n=200
Patients, %
0
5
10
15
Blood and lymphatic system disorders
Cardiac disorders
Congenital, familial, and genetic disorders
Ear and labyrinth disorders
Endocrine disorders
Eye disorders
Gastrointestinal disorders
General disorders and administration site conditions
Hepatobiliary disorders
Immune system disorders
Infections and infestations
Injury, poisoning, and procedural complications
Investigations
Metabolism and nutrition disorders
Musculoskeletal and connective tissue disorders
Neoplasms benign, malignant, and unspecified (incl cysts and polyps)
Nervous system disorders
Psychiatric disorders
Renal and urinary disorders
Reproductive system and breast disorders
Respiratory, thoracic, and mediastinal disorders
Skin and subcutaneous tissue disorders
Social circumstances
Surgical and medical procedures
Vascular disorders

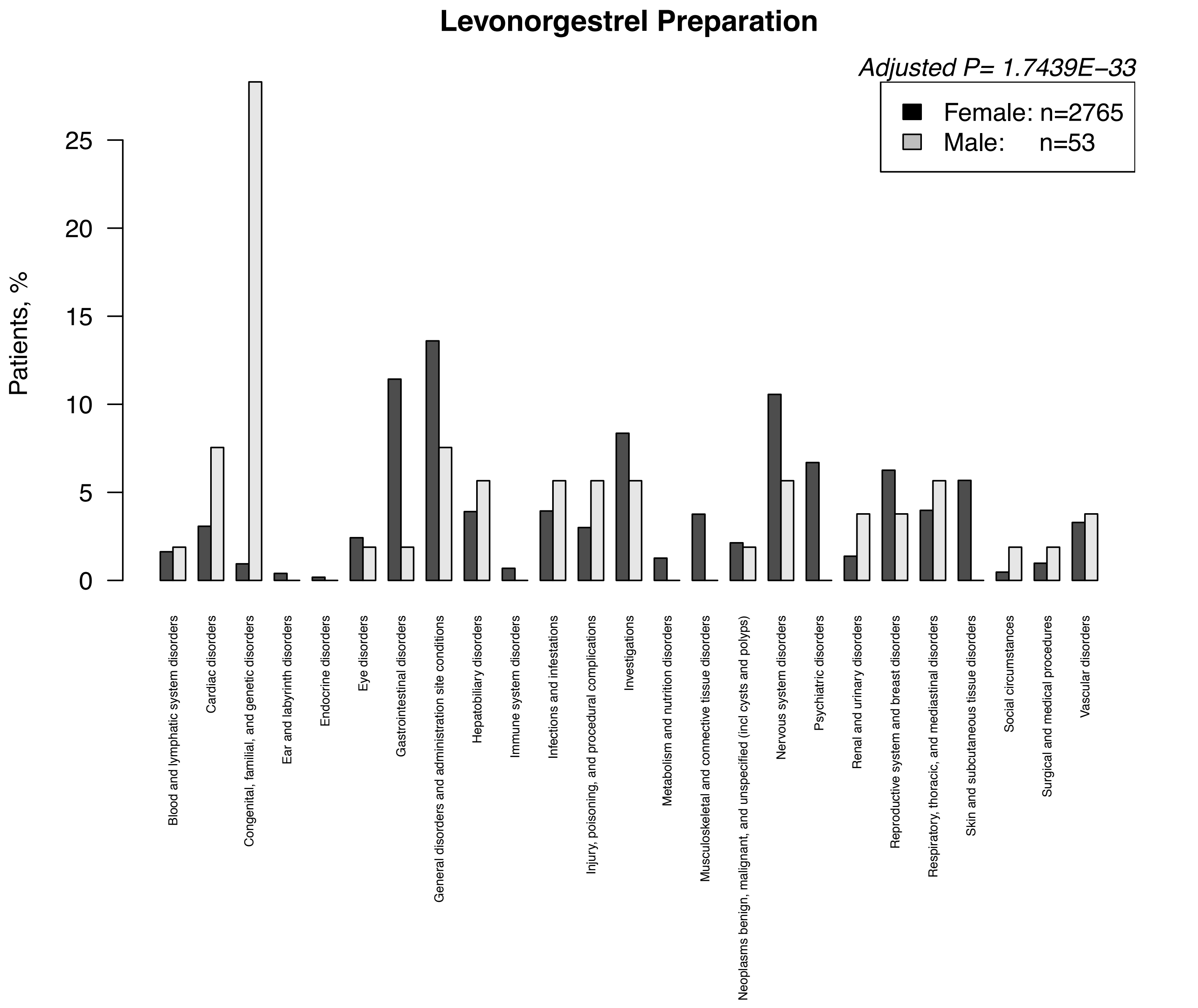
Levonorgestrel Preparation
Adjusted P= 1.7439E−33
Female: n=2765
Male: n=53
Patients, %
0
5
10
15
20
25
Blood and lymphatic system disorders
Cardiac disorders
Congenital, familial, and genetic disorders
Ear and labyrinth disorders
Endocrine disorders
Eye disorders
Gastrointestinal disorders
General disorders and administration site conditions
Hepatobiliary disorders
Immune system disorders
Infections and infestations
Injury, poisoning, and procedural complications
Investigations
Metabolism and nutrition disorders
Musculoskeletal and connective tissue disorders
Neoplasms benign, malignant, and unspecified (incl cysts and polyps)
Nervous system disorders
Psychiatric disorders
Renal and urinary disorders
Reproductive system and breast disorders
Respiratory, thoracic, and mediastinal disorders
Skin and subcutaneous tissue disorders
Social circumstances
Surgical and medical procedures
Vascular disorders

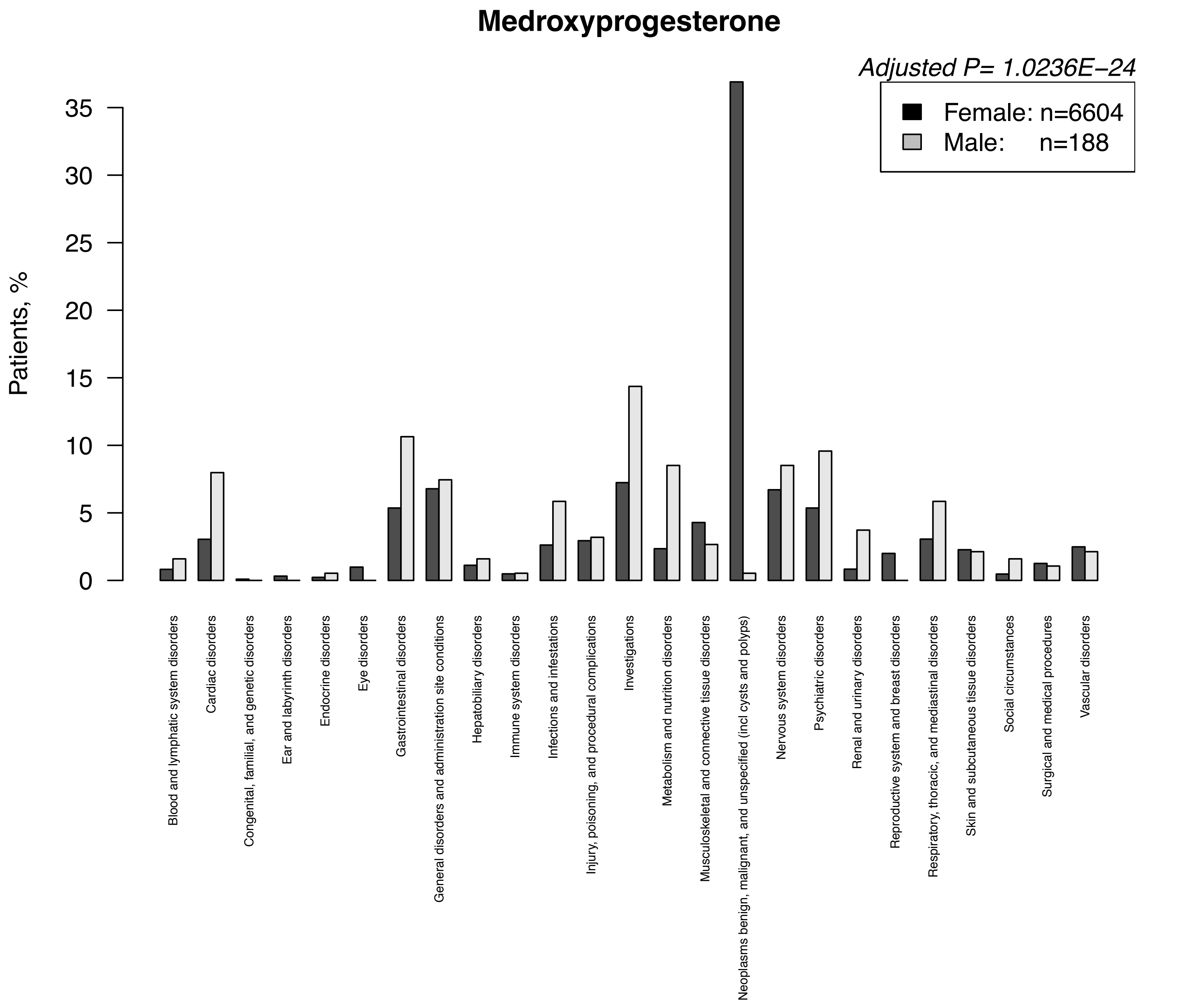
Medroxyprogesterone
Adjusted P= 1.0236E−24
Female: n=6604
Male: n=188
Patients, %
0
5
10
15
20
25
30
35
Blood and lymphatic system disorders
Cardiac disorders
Congenital, familial, and genetic disorders
Ear and labyrinth disorders
Endocrine disorders
Eye disorders
Gastrointestinal disorders
General disorders and administration site conditions
Hepatobiliary disorders
Immune system disorders
Infections and infestations
Injury, poisoning, and procedural complications
Investigations
Metabolism and nutrition disorders
Musculoskeletal and connective tissue disorders
Neoplasms benign, malignant, and unspecified (incl cysts and polyps)
Nervous system disorders
Psychiatric disorders
Renal and urinary disorders
Reproductive system and breast disorders
Respiratory, thoracic, and mediastinal disorders
Skin and subcutaneous tissue disorders
Social circumstances
Surgical and medical procedures
Vascular disorders

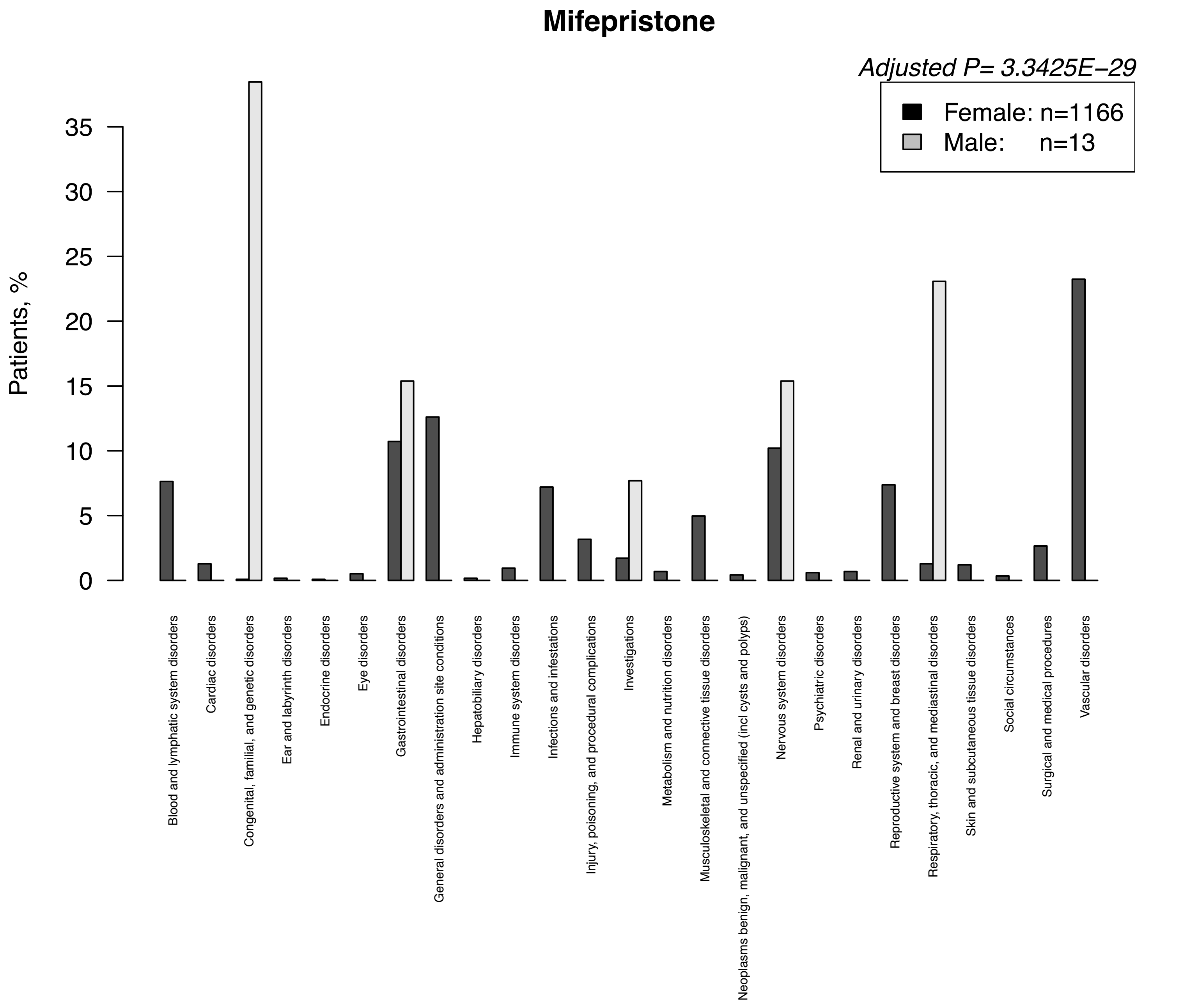

Mifepristone
Adjusted P= 3.3425E−29
Female: n=1166
Male: n=13
Patients, %
0
5
10
15
20
25
30
35
Blood and lymphatic system disorders
Cardiac disorders
Congenital, familial, and genetic disorders
Ear and labyrinth disorders
Endocrine disorders
Eye disorders
Gastrointestinal disorders
General disorders and administration site conditions
Hepatobiliary disorders
Immune system disorders
Infections and infestations
Injury, poisoning, and procedural complications
Investigations
Metabolism and nutrition disorders
Musculoskeletal and connective tissue disorders
Neoplasms benign, malignant, and unspecified (incl cysts and polyps)
Nervous system disorders
Psychiatric disorders
Renal and urinary disorders
Reproductive system and breast disorders
Respiratory, thoracic, and mediastinal disorders
Skin and subcutaneous tissue disorders
Social circumstances
Surgical and medical procedures
Vascular disorders

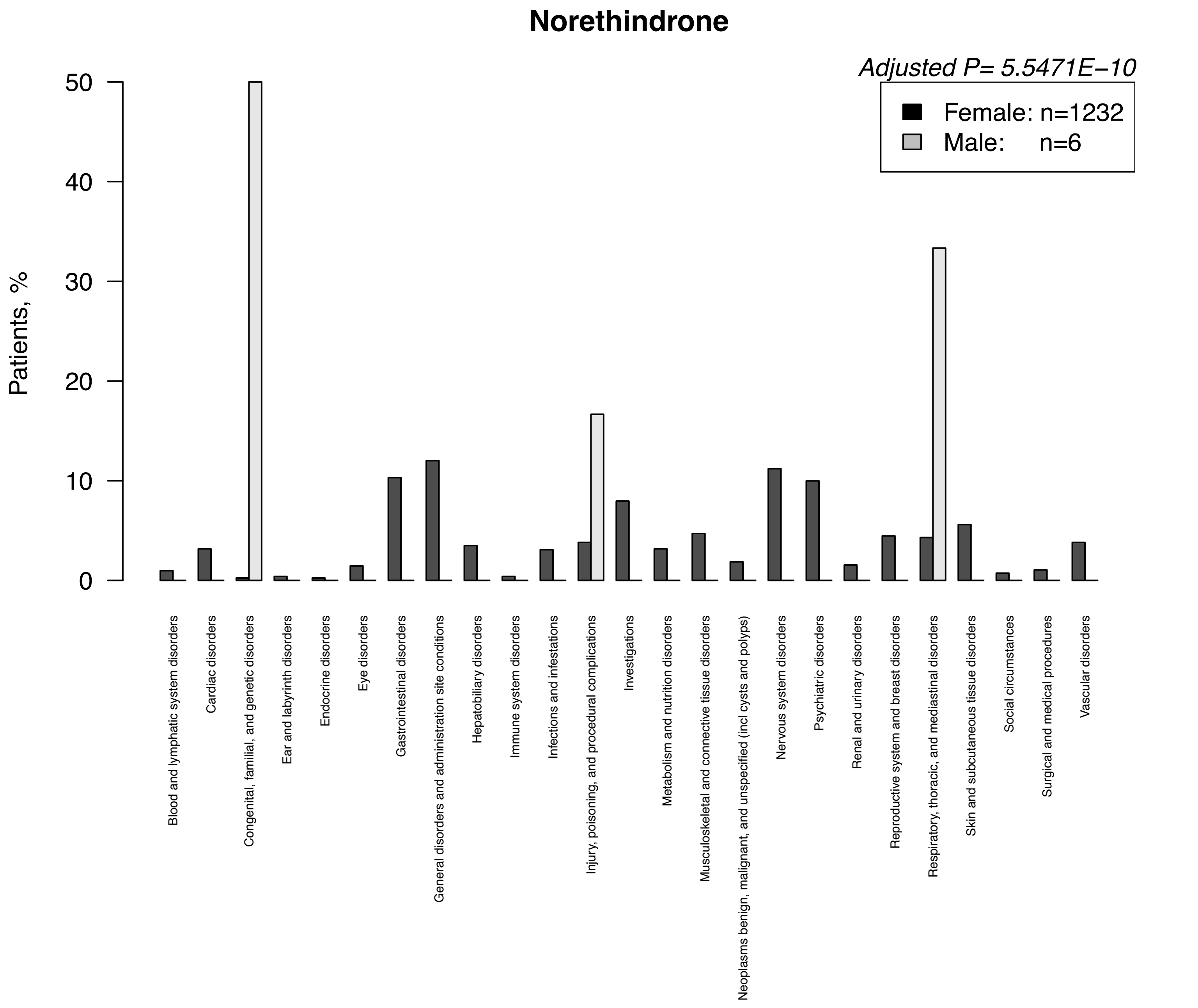
Norethindrone
Adjusted P= 5.5471E−10
Female: n=1232
Male: n=6
Patients, %
0
10
20
30
40
50
Blood and lymphatic system disorders
Cardiac disorders
Congenital, familial, and genetic disorders
Ear and labyrinth disorders
Endocrine disorders
Eye disorders
Gastrointestinal disorders
General disorders and administration site conditions
Hepatobiliary disorders
Immune system disorders
Infections and infestations
Injury, poisoning, and procedural complications
Investigations
Metabolism and nutrition disorders
Musculoskeletal and connective tissue disorders
Neoplasms benign, malignant, and unspecified (incl cysts and polyps)
Nervous system disorders
Psychiatric disorders
Renal and urinary disorders
Reproductive system and breast disorders
Respiratory, thoracic, and mediastinal disorders
Skin and subcutaneous tissue disorders
Social circumstances
Surgical and medical procedures
Vascular disorders

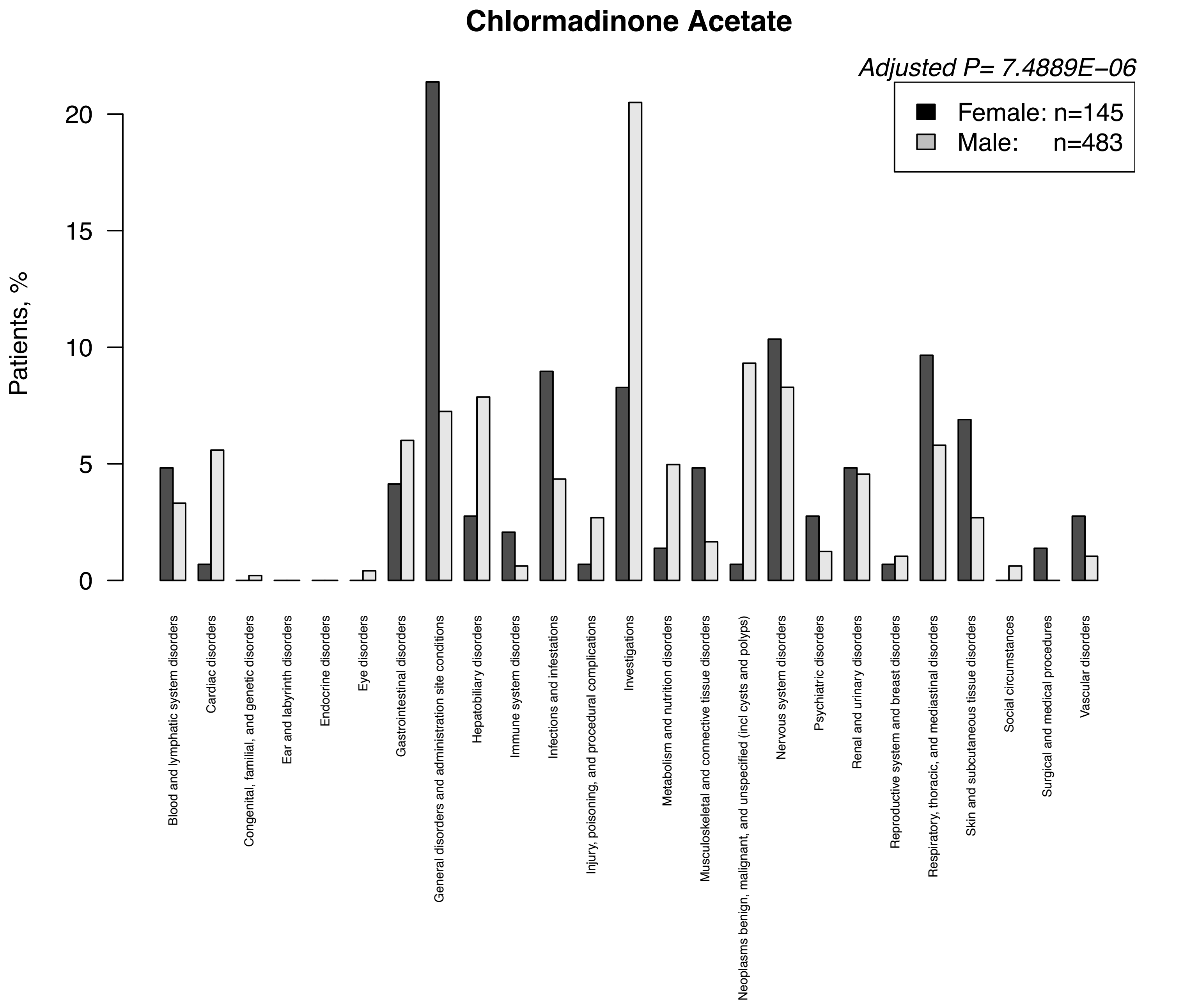

Chlormadinone Acetate
Adjusted P= 7.4889E−06
Female: n=145
Male: n=483
Patients, %
0
5
10
15
20
Blood and lymphatic system disorders
Cardiac disorders
Congenital, familial, and genetic disorders
Ear and labyrinth disorders
Endocrine disorders
Eye disorders
Gastrointestinal disorders
General disorders and administration site conditions
Hepatobiliary disorders
Immune system disorders
Infections and infestations
Injury, poisoning, and procedural complications
Investigations
Metabolism and nutrition disorders
Musculoskeletal and connective tissue disorders
Neoplasms benign, malignant, and unspecified (incl cysts and polyps)
Nervous system disorders
Psychiatric disorders
Renal and urinary disorders
Reproductive system and breast disorders
Respiratory, thoracic, and mediastinal disorders
Skin and subcutaneous tissue disorders
Social circumstances
Surgical and medical procedures
Vascular disorders

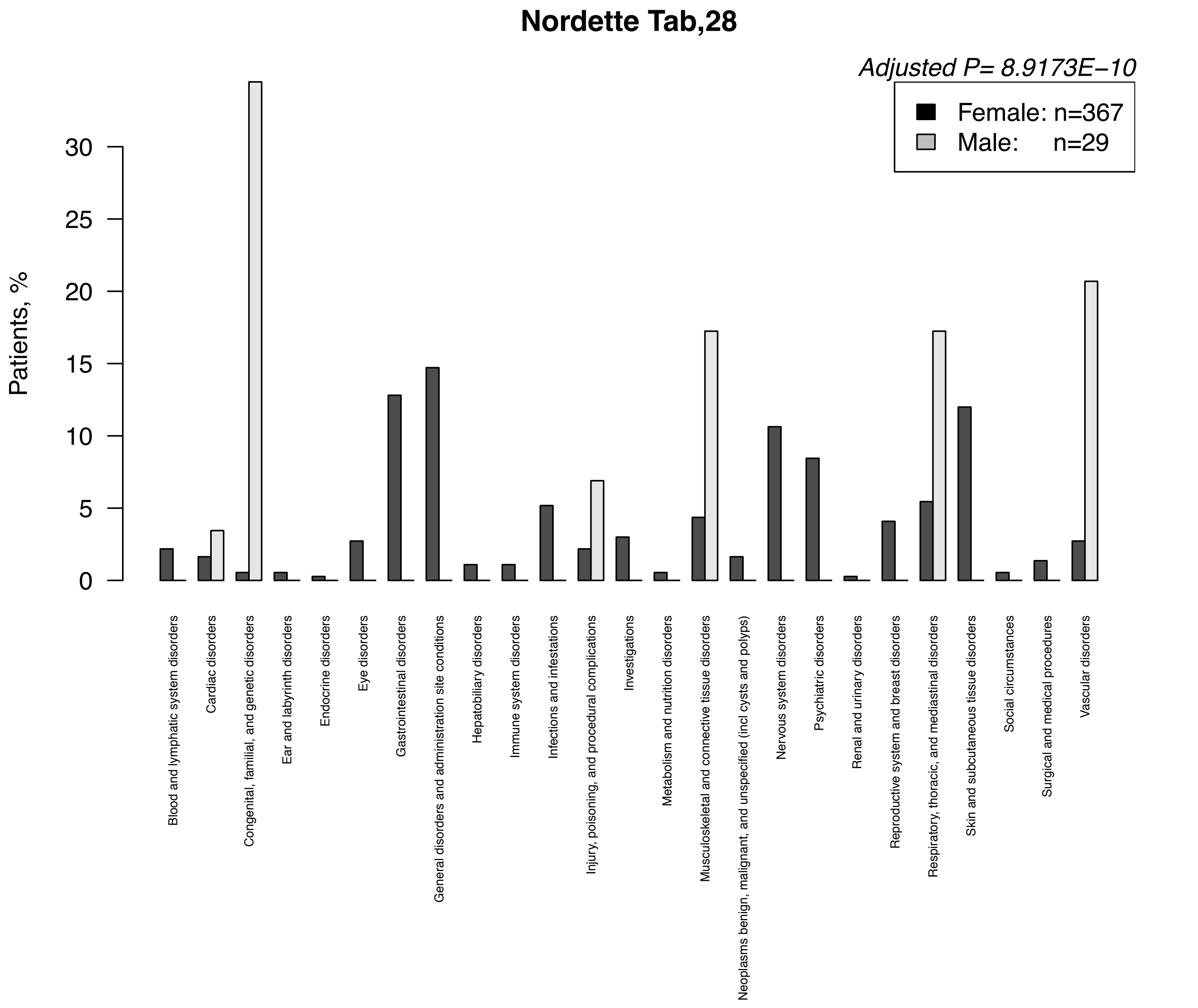
Nordette Tab,28
Adjusted P= 8.9173E−10
Female: n=367
Male: n=29
Patients, %
0
5
10
15
20
25
30
Blood and lymphatic system disorders
Cardiac disorders
Congenital, familial, and genetic disorders
Ear and labyrinth disorders
Endocrine disorders
Eye disorders
Gastrointestinal disorders
General disorders and administration site conditions
Hepatobiliary disorders
Immune system disorders
Infections and infestations
Injury, poisoning, and procedural complications
Investigations
Metabolism and nutrition disorders
Musculoskeletal and connective tissue disorders
Neoplasms benign, malignant, and unspecified (incl cysts and polyps)
Nervous system disorders
Psychiatric disorders
Renal and urinary disorders
Reproductive system and breast disorders
Respiratory, thoracic, and mediastinal disorders
Skin and subcutaneous tissue disorders
Social circumstances
Surgical and medical procedures
Vascular disorders

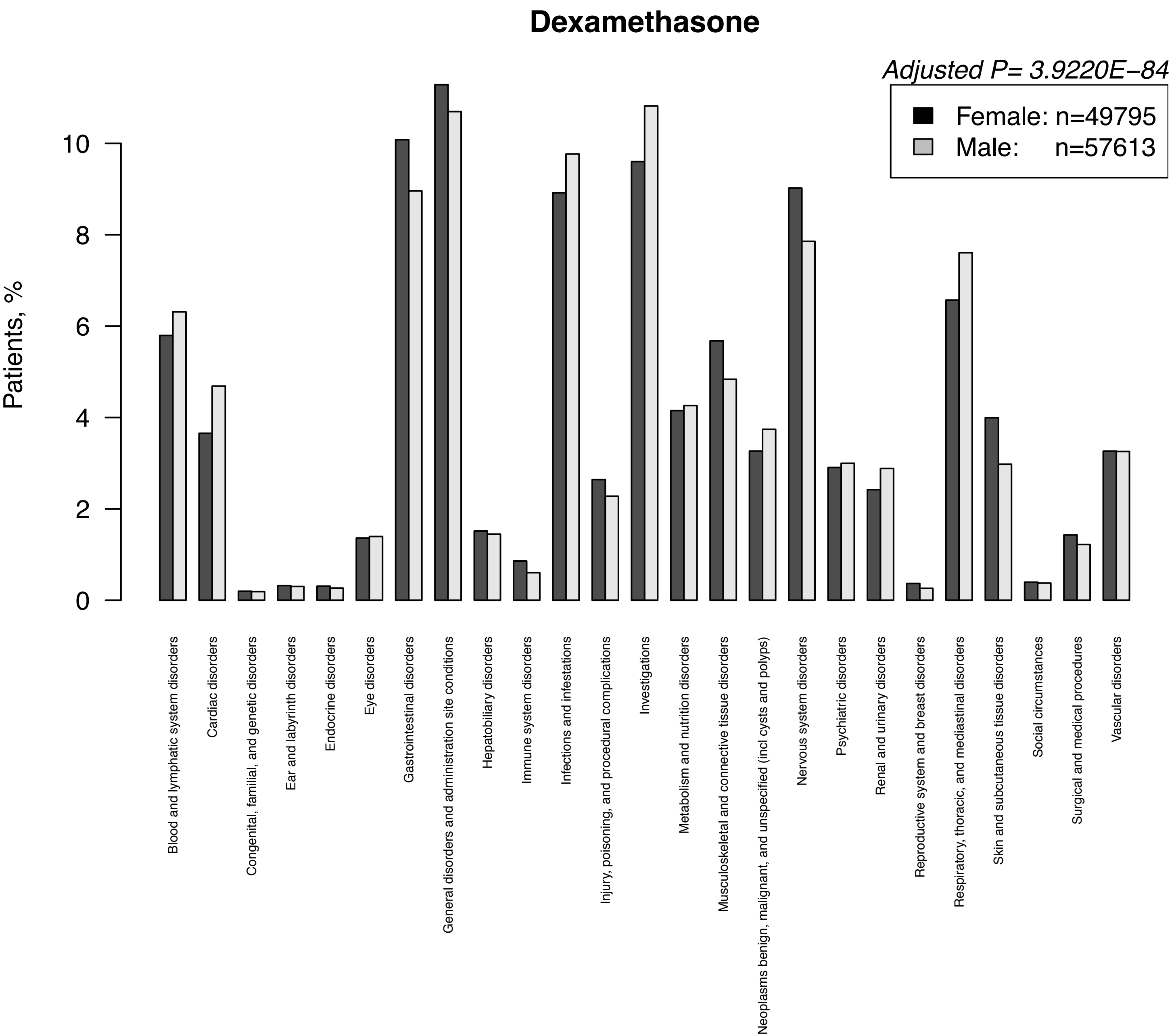

Dexamethasone
Adjusted P= 3.9220E−84
Female: n=49795
Male: n=57613
Patients, %
0
2
4
6
8
10
Blood and lymphatic system disorders
Cardiac disorders
Congenital, familial, and genetic disorders
Ear and labyrinth disorders
Endocrine disorders
Eye disorders
Gastrointestinal disorders
General disorders and administration site conditions
Hepatobiliary disorders
Immune system disorders
Infections and infestations
Injury, poisoning, and procedural complications
Investigations
Metabolism and nutrition disorders
Musculoskeletal and connective tissue disorders
Neoplasms benign, malignant, and unspecified (incl cysts and polyps)
Nervous system disorders
Psychiatric disorders
Renal and urinary disorders
Reproductive system and breast disorders
Respiratory, thoracic, and mediastinal disorders
Skin and subcutaneous tissue disorders
Social circumstances
Surgical and medical procedures
Vascular disorders

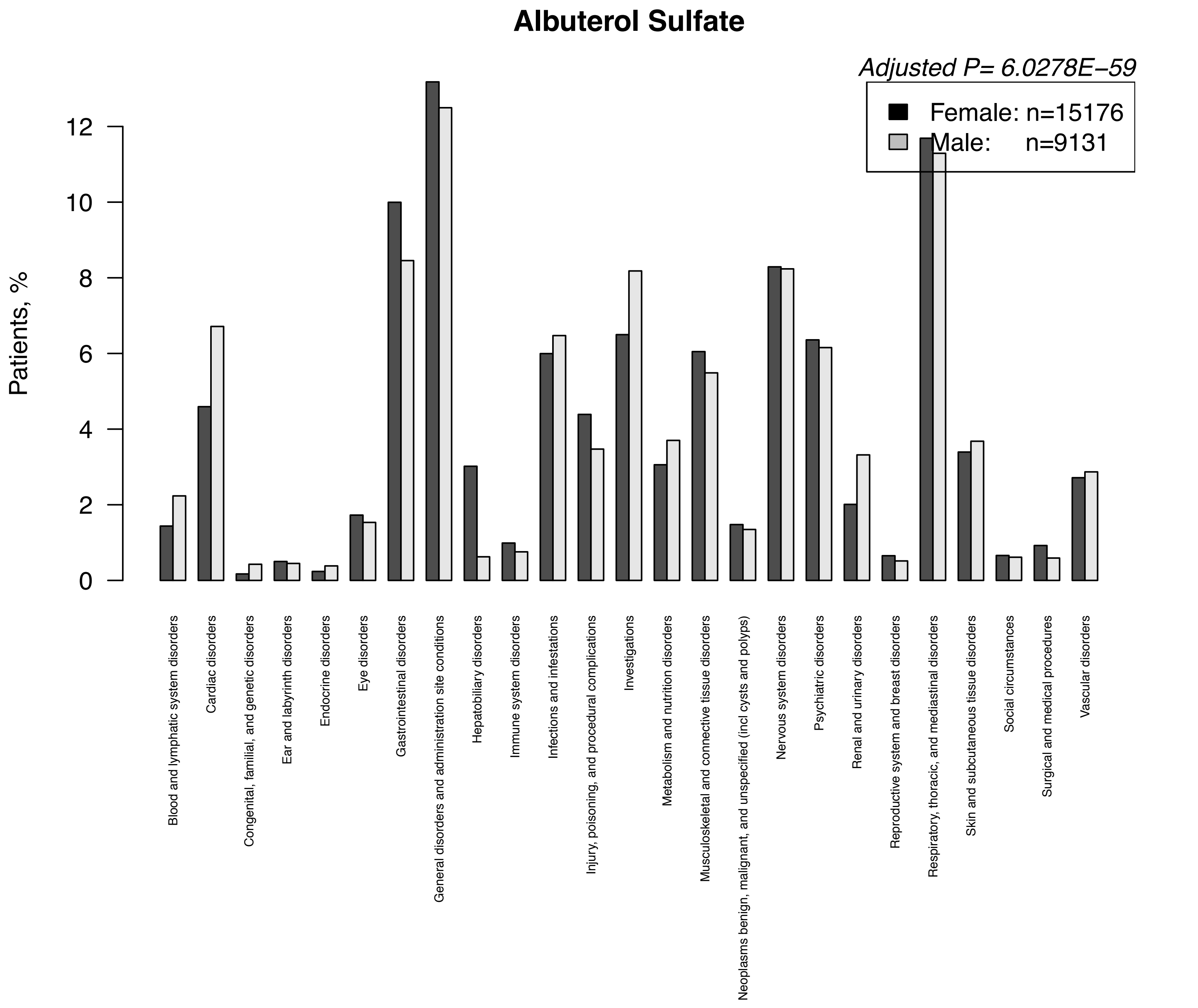

Albuterol Sulfate
Adjusted P= 6.0278E−59
Female: n=15176
Male: n=9131
Patients, %
0
2
4
6
8
10
12
Blood and lymphatic system disorders
Cardiac disorders
Congenital, familial, and genetic disorders
Ear and labyrinth disorders
Endocrine disorders
Eye disorders
Gastrointestinal disorders
General disorders and administration site conditions
Hepatobiliary disorders
Immune system disorders
Infections and infestations
Injury, poisoning, and procedural complications
Investigations
Metabolism and nutrition disorders
Musculoskeletal and connective tissue disorders
Neoplasms benign, malignant, and unspecified (incl cysts and polyps)
Nervous system disorders
Psychiatric disorders
Renal and urinary disorders
Reproductive system and breast disorders
Respiratory, thoracic, and mediastinal disorders
Skin and subcutaneous tissue disorders
Social circumstances
Surgical and medical procedures
Vascular disorders

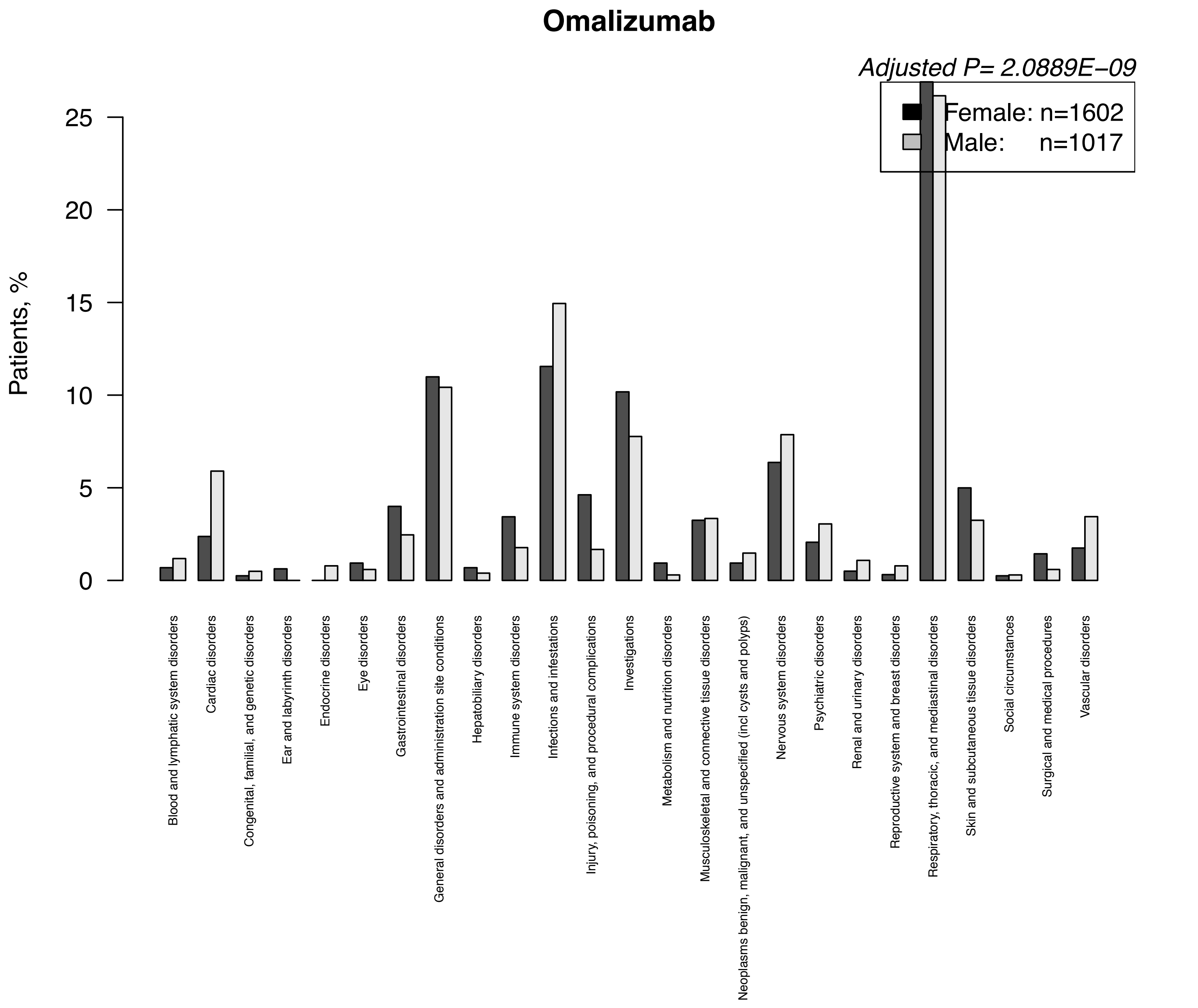

Omalizumab
Adjusted P= 2.0889E−09
Female: n=1602
Male: n=1017
Patients, %
0
5
10
15
20
25
Blood and lymphatic system disorders
Cardiac disorders
Congenital, familial, and genetic disorders
Ear and labyrinth disorders
Endocrine disorders
Eye disorders
Gastrointestinal disorders
General disorders and administration site conditions
Hepatobiliary disorders
Immune system disorders
Infections and infestations
Injury, poisoning, and procedural complications
Investigations
Metabolism and nutrition disorders
Musculoskeletal and connective tissue disorders
Neoplasms benign, malignant, and unspecified (incl cysts and polyps)
Nervous system disorders
Psychiatric disorders
Renal and urinary disorders
Reproductive system and breast disorders
Respiratory, thoracic, and mediastinal disorders
Skin and subcutaneous tissue disorders
Social circumstances
Surgical and medical procedures
Vascular disorders

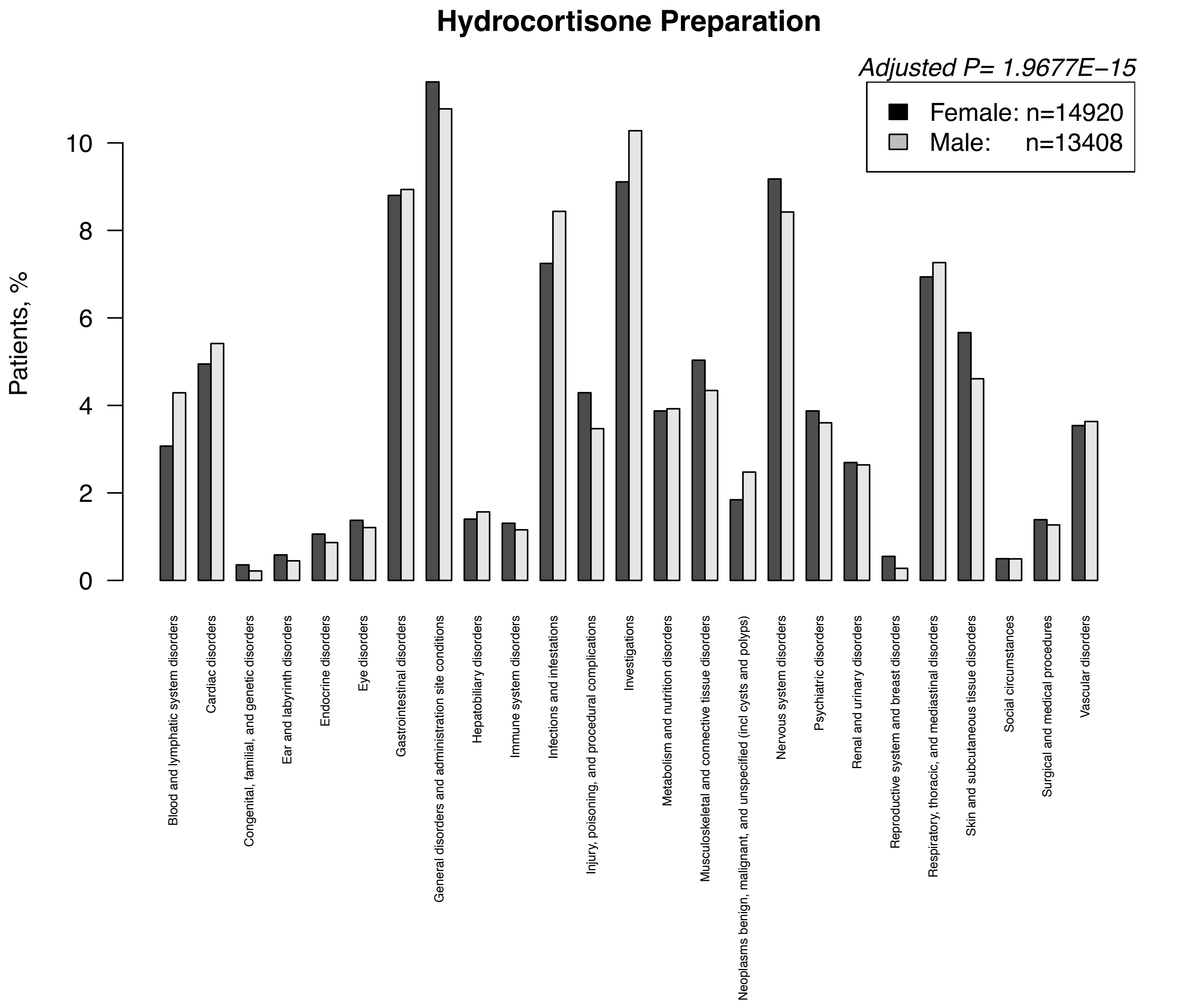
Hydrocortisone Preparation
Adjusted P= 1.9677E−15
Female: n=14920
Male: n=13408
Patients, %
0
2
4
6
8
10
Blood and lymphatic system disorders
Cardiac disorders
Congenital, familial, and genetic disorders
Ear and labyrinth disorders
Endocrine disorders
Eye disorders
Gastrointestinal disorders
General disorders and administration site conditions
Hepatobiliary disorders
Immune system disorders
Infections and infestations
Injury, poisoning, and procedural complications
Investigations
Metabolism and nutrition disorders
Musculoskeletal and connective tissue disorders
Neoplasms benign, malignant, and unspecified (incl cysts and polyps)
Nervous system disorders
Psychiatric disorders
Renal and urinary disorders
Reproductive system and breast disorders
Respiratory, thoracic, and mediastinal disorders
Skin and subcutaneous tissue disorders
Social circumstances
Surgical and medical procedures
Vascular disorders

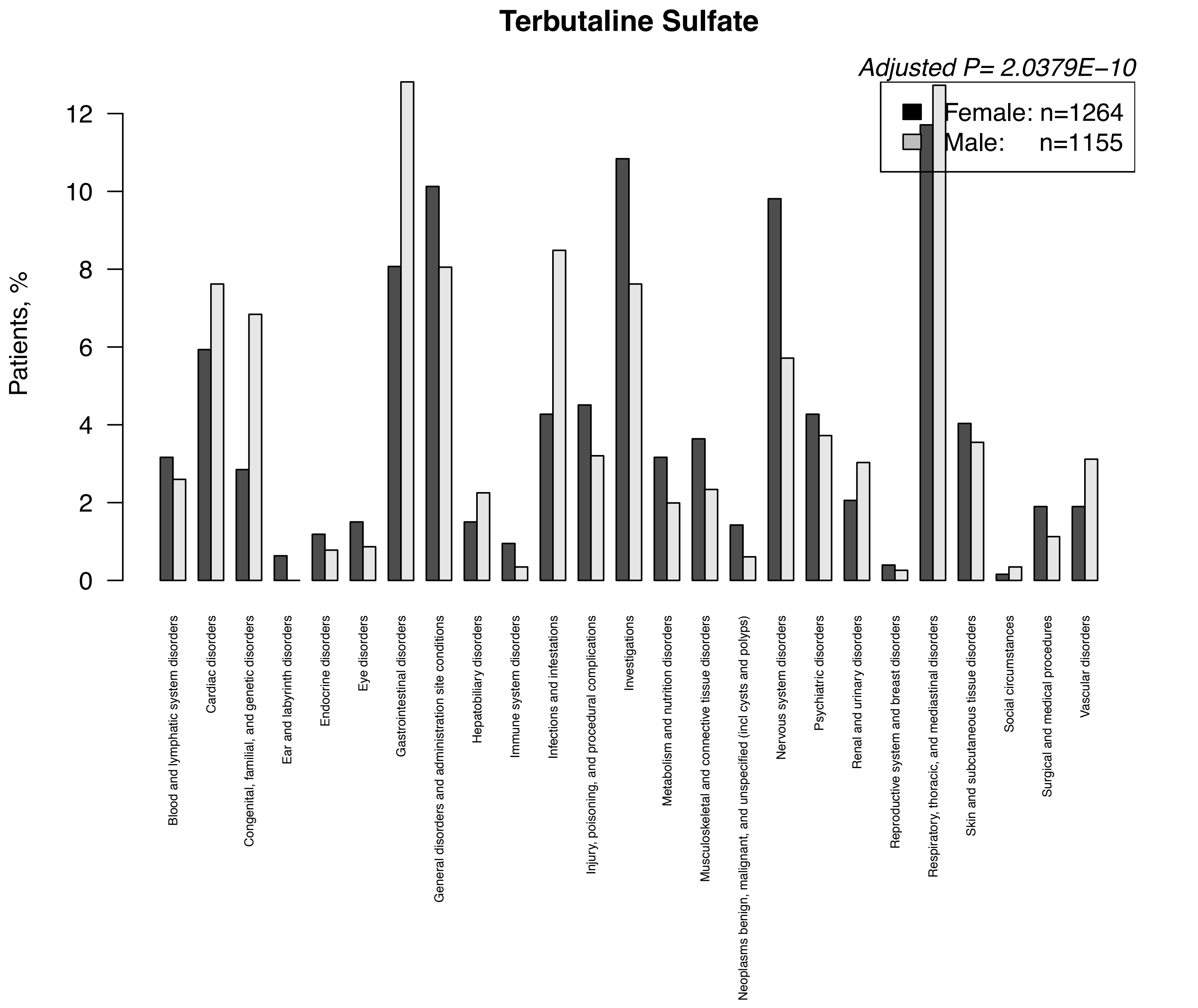
Terbutaline Sulfate
Adjusted P= 2.0379E−10
Female: n=1264
Male: n=1155
Patients, %
0
2
4
6
8
10
12
Blood and lymphatic system disorders
Cardiac disorders
Congenital, familial, and genetic disorders
Ear and labyrinth disorders
Endocrine disorders
Eye disorders
Gastrointestinal disorders
General disorders and administration site conditions
Hepatobiliary disorders
Immune system disorders
Infections and infestations
Injury, poisoning, and procedural complications
Investigations
Metabolism and nutrition disorders
Musculoskeletal and connective tissue disorders
Neoplasms benign, malignant, and unspecified (incl cysts and polyps)
Nervous system disorders
Psychiatric disorders
Renal and urinary disorders
Reproductive system and breast disorders
Respiratory, thoracic, and mediastinal disorders
Skin and subcutaneous tissue disorders
Social circumstances
Surgical and medical procedures
Vascular disorders

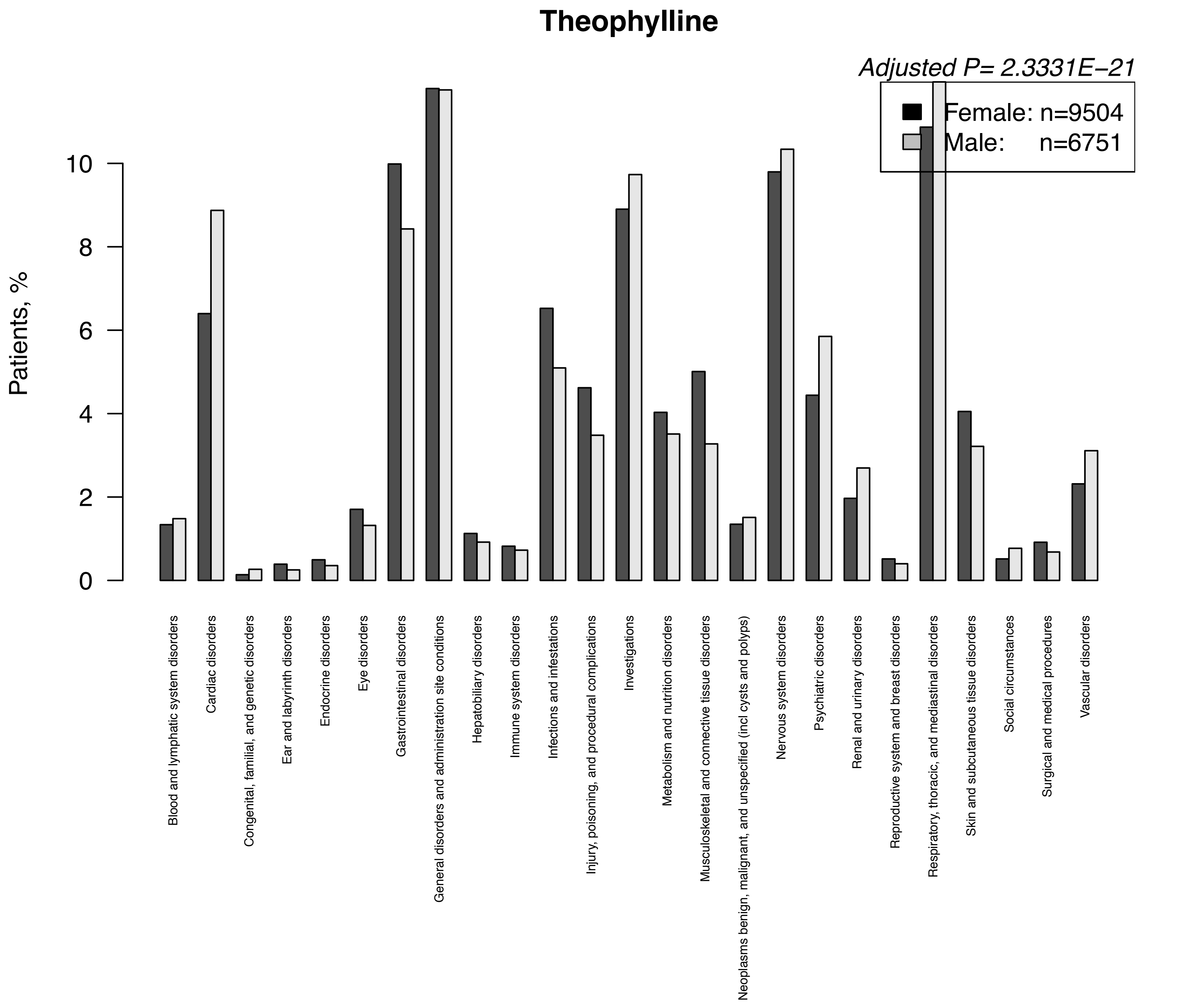

Theophylline
Adjusted P= 2.3331E−21
Female: n=9504
Male: n=6751
Patients, %
0
2
4
6
8
10
Blood and lymphatic system disorders
Cardiac disorders
Congenital, familial, and genetic disorders
Ear and labyrinth disorders
Endocrine disorders
Eye disorders
Gastrointestinal disorders
General disorders and administration site conditions
Hepatobiliary disorders
Immune system disorders
Infections and infestations
Injury, poisoning, and procedural complications
Investigations
Metabolism and nutrition disorders
Musculoskeletal and connective tissue disorders
Neoplasms benign, malignant, and unspecified (incl cysts and polyps)
Nervous system disorders
Psychiatric disorders
Renal and urinary disorders
Reproductive system and breast disorders
Respiratory, thoracic, and mediastinal disorders
Skin and subcutaneous tissue disorders
Social circumstances
Surgical and medical procedures
Vascular disorders

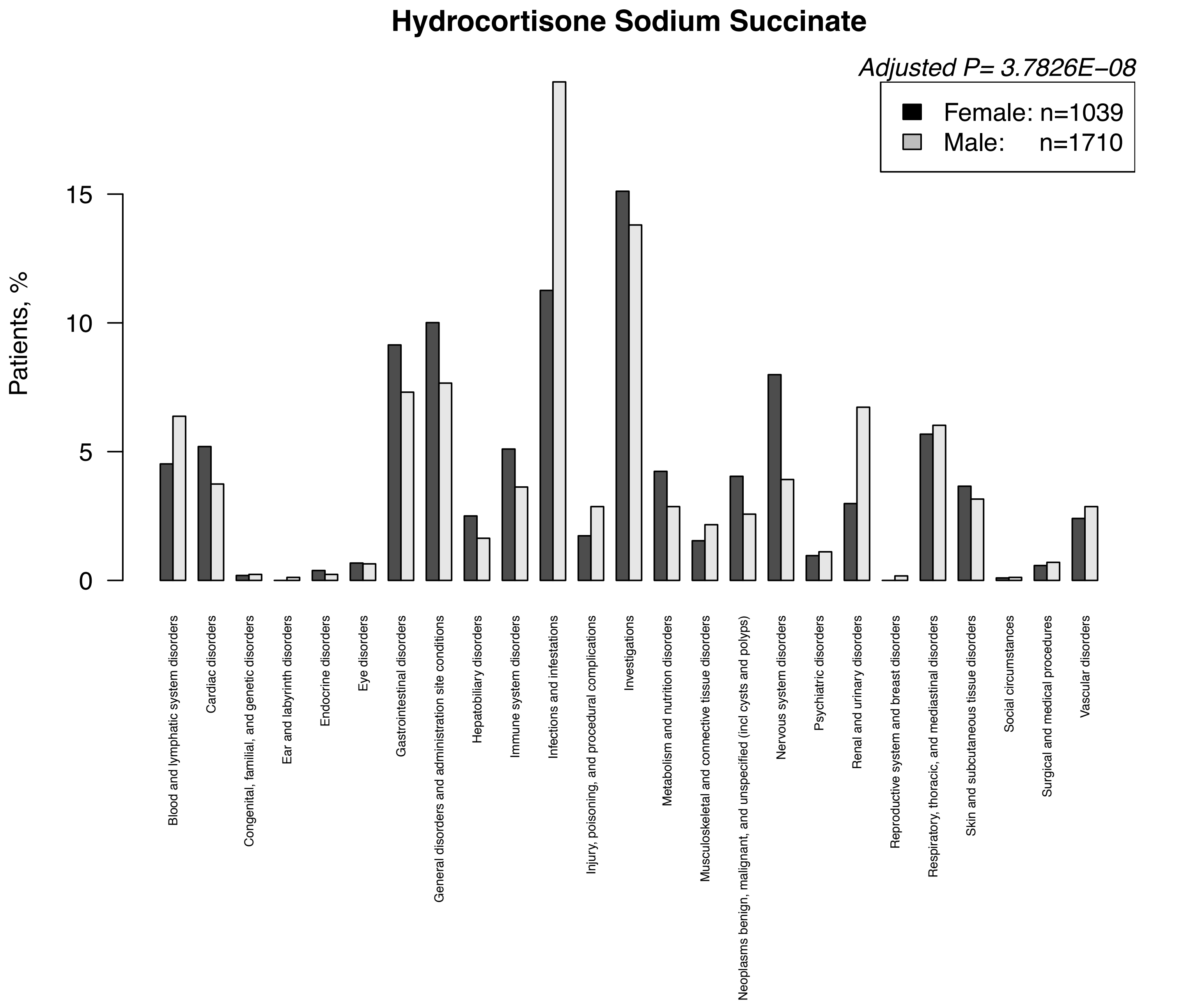
Hydrocortisone Sodium Succinate
Adjusted P= 3.7826E−08
Female: n=1039
Male: n=1710
Patients, %
0
5
10
15
Blood and lymphatic system disorders
Cardiac disorders
Congenital, familial, and genetic disorders
Ear and labyrinth disorders
Endocrine disorders
Eye disorders
Gastrointestinal disorders
General disorders and administration site conditions
Hepatobiliary disorders
Immune system disorders
Infections and infestations
Injury, poisoning, and procedural complications
Investigations
Metabolism and nutrition disorders
Musculoskeletal and connective tissue disorders
Neoplasms benign, malignant, and unspecified (incl cysts and polyps)
Nervous system disorders
Psychiatric disorders
Renal and urinary disorders
Reproductive system and breast disorders
Respiratory, thoracic, and mediastinal disorders
Skin and subcutaneous tissue disorders
Social circumstances
Surgical and medical procedures
Vascular disorders

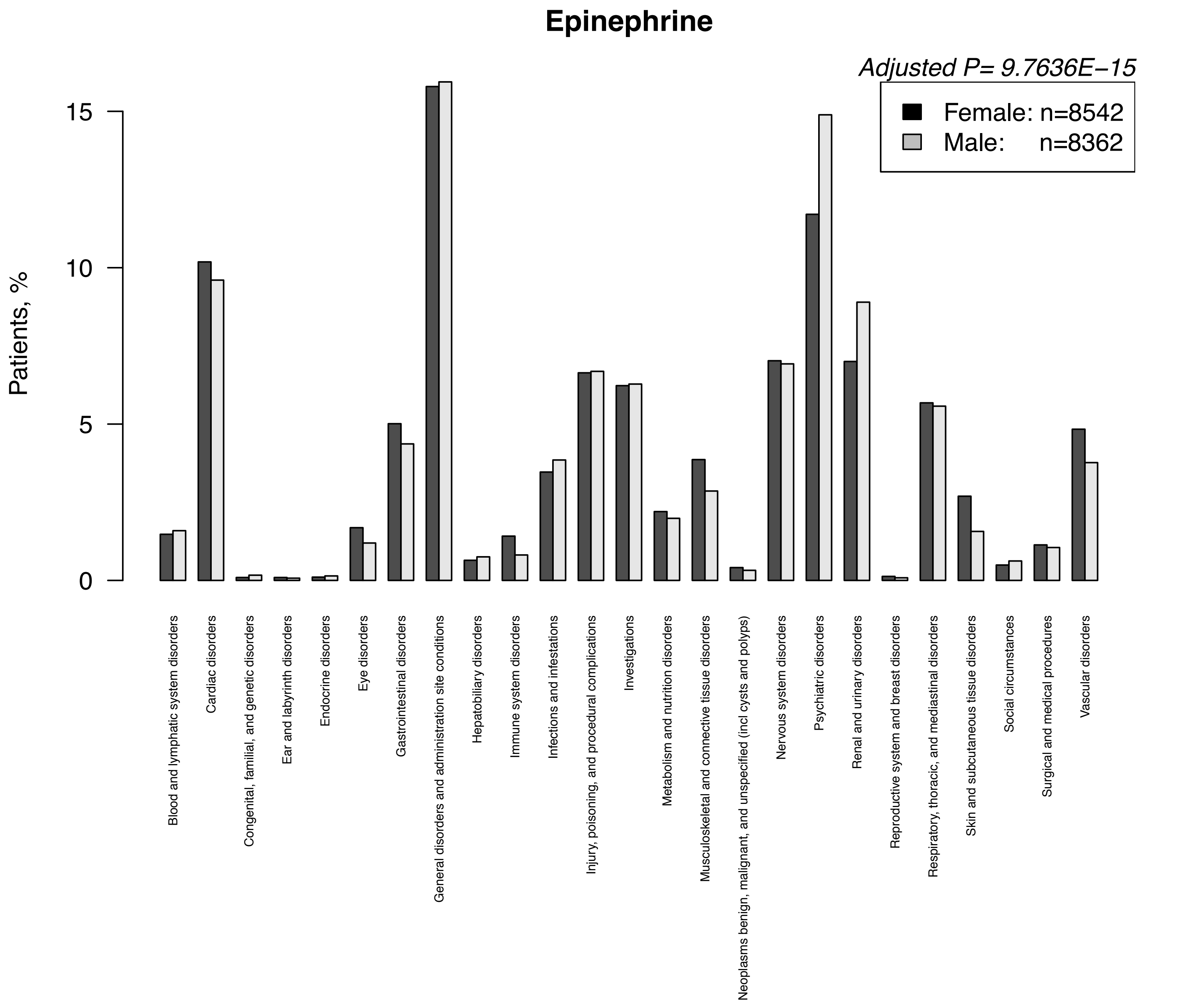
Epinephrine
Adjusted P= 9.7636E−15
Female: n=8542
Male: n=8362
Patients, %
0
5
10
15
Blood and lymphatic system disorders
Cardiac disorders
Congenital, familial, and genetic disorders
Ear and labyrinth disorders
Endocrine disorders
Eye disorders
Gastrointestinal disorders
General disorders and administration site conditions
Hepatobiliary disorders
Immune system disorders
Infections and infestations
Injury, poisoning, and procedural complications
Investigations
Metabolism and nutrition disorders
Musculoskeletal and connective tissue disorders
Neoplasms benign, malignant, and unspecified (incl cysts and polyps)
Nervous system disorders
Psychiatric disorders
Renal and urinary disorders
Reproductive system and breast disorders
Respiratory, thoracic, and mediastinal disorders
Skin and subcutaneous tissue disorders
Social circumstances
Surgical and medical procedures
Vascular disorders

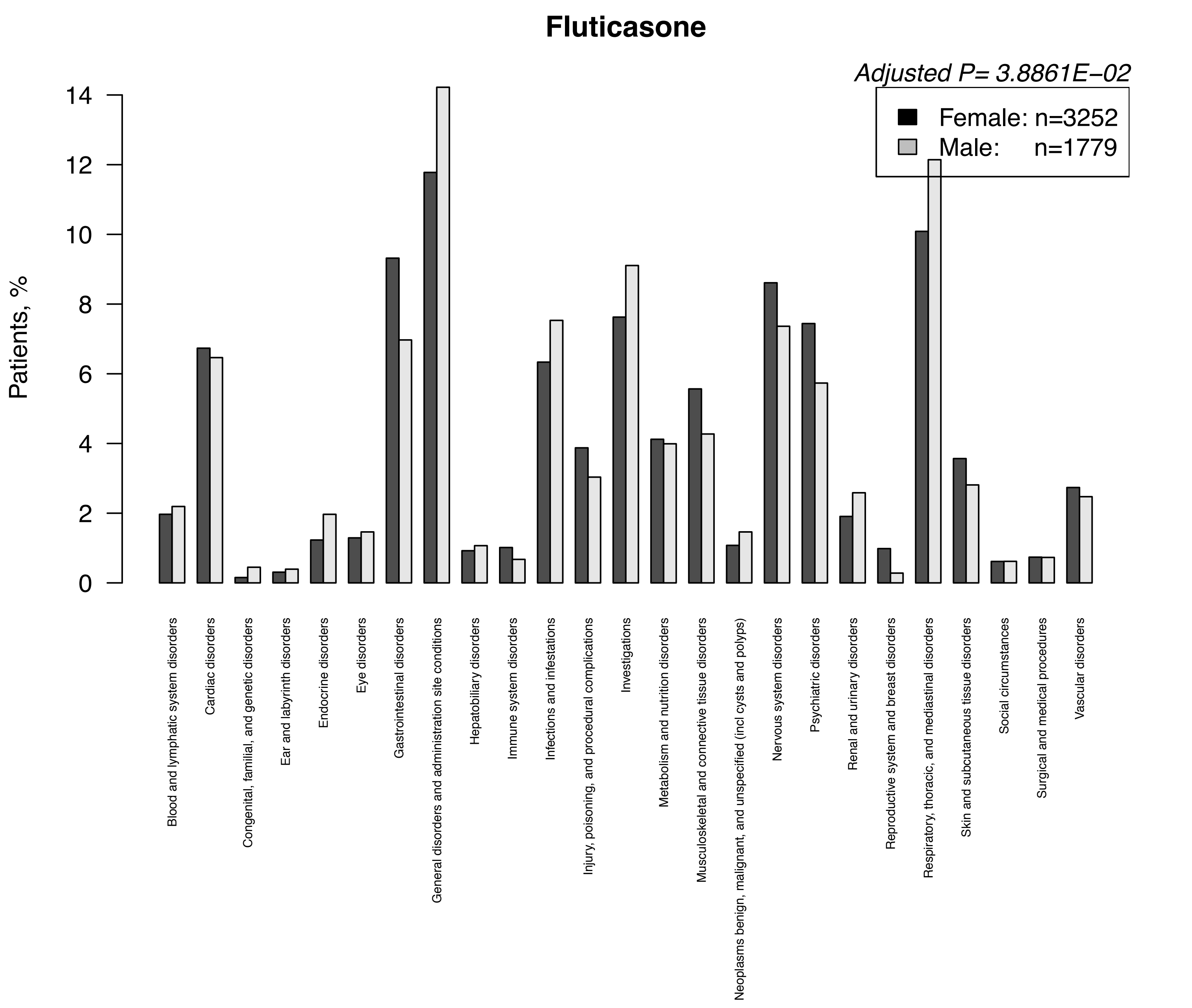
Fluticasone
Adjusted P= 3.8861E−02
Female: n=3252
Male: n=1779
Patients, %
0
2
4
6
8
10
12
14
Blood and lymphatic system disorders
Cardiac disorders
Congenital, familial, and genetic disorders
Ear and labyrinth disorders
Endocrine disorders
Eye disorders
Gastrointestinal disorders
General disorders and administration site conditions
Hepatobiliary disorders
Immune system disorders
Infections and infestations
Injury, poisoning, and procedural complications
Investigations
Metabolism and nutrition disorders
Musculoskeletal and connective tissue disorders
Neoplasms benign, malignant, and unspecified (incl cysts and polyps)
Nervous system disorders
Psychiatric disorders
Renal and urinary disorders
Reproductive system and breast disorders
Respiratory, thoracic, and mediastinal disorders
Skin and subcutaneous tissue disorders
Social circumstances
Surgical and medical procedures
Vascular disorders

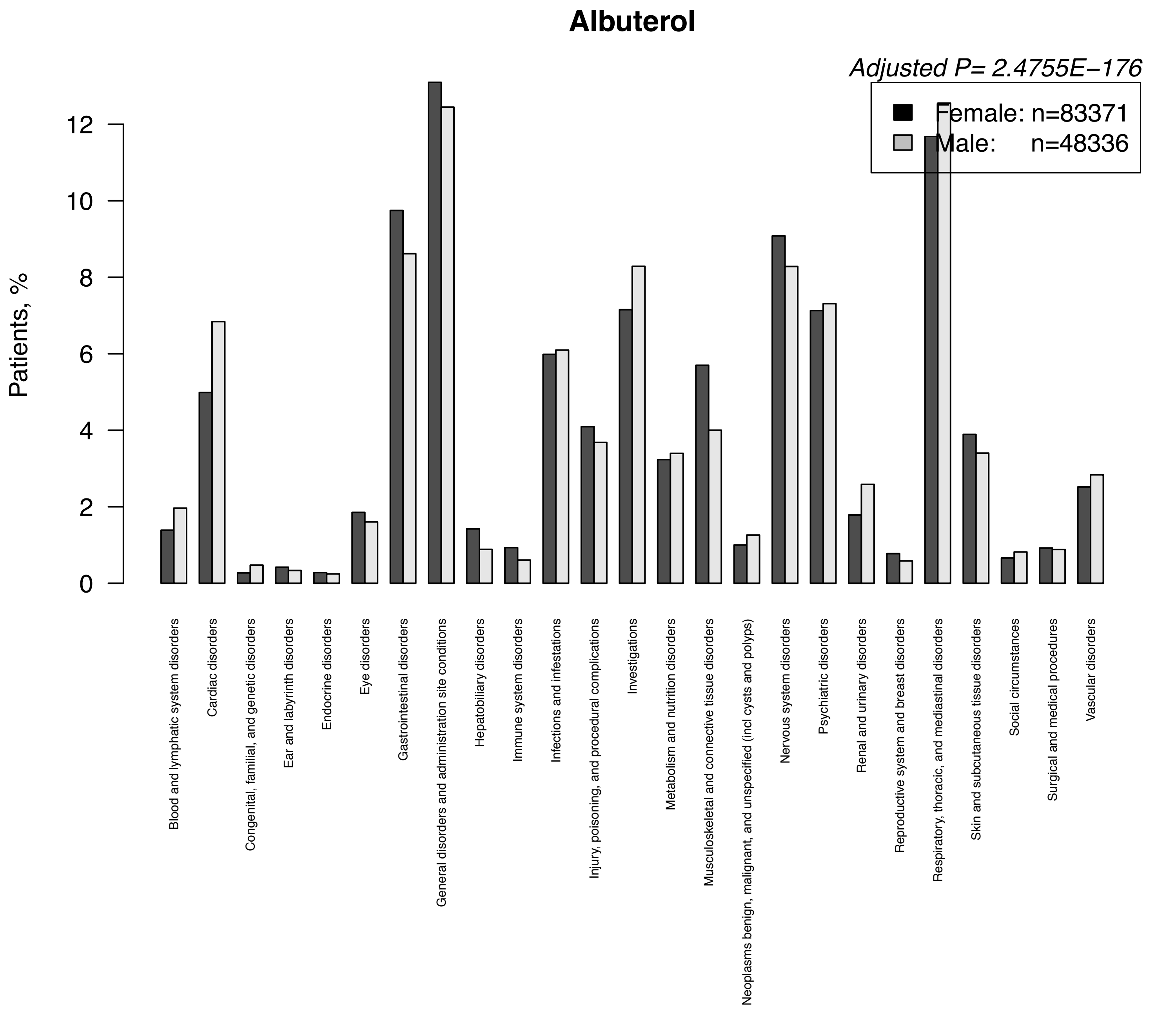

Albuterol
Adjusted P= 2.4755E−176
Female: n=83371
Male: n=48336
Patients, %
0
2
4
6
8
10
12
Blood and lymphatic system disorders
Cardiac disorders
Congenital, familial, and genetic disorders
Ear and labyrinth disorders
Endocrine disorders
Eye disorders
Gastrointestinal disorders
General disorders and administration site conditions
Hepatobiliary disorders
Immune system disorders
Infections and infestations
Injury, poisoning, and procedural complications
Investigations
Metabolism and nutrition disorders
Musculoskeletal and connective tissue disorders
Neoplasms benign, malignant, and unspecified (incl cysts and polyps)
Nervous system disorders
Psychiatric disorders
Renal and urinary disorders
Reproductive system and breast disorders
Respiratory, thoracic, and mediastinal disorders
Skin and subcutaneous tissue disorders
Social circumstances
Surgical and medical procedures
Vascular disorders

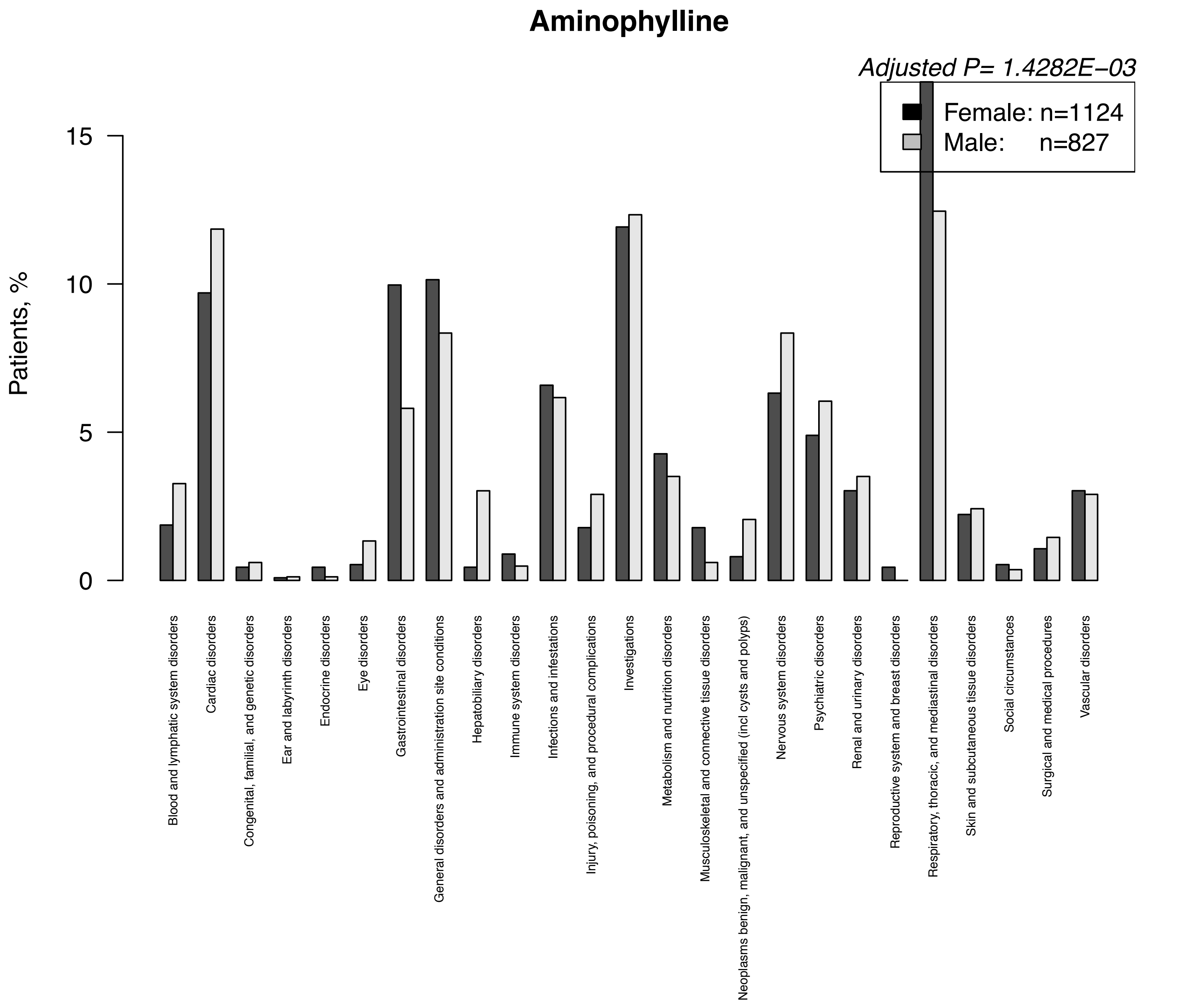
Aminophylline
Adjusted P= 1.4282E−03
Female: n=1124
Male: n=827
Patients, %
0
5
10
15
Blood and lymphatic system disorders
Cardiac disorders
Congenital, familial, and genetic disorders
Ear and labyrinth disorders
Endocrine disorders
Eye disorders
Gastrointestinal disorders
General disorders and administration site conditions
Hepatobiliary disorders
Immune system disorders
Infections and infestations
Injury, poisoning, and procedural complications
Investigations
Metabolism and nutrition disorders
Musculoskeletal and connective tissue disorders
Neoplasms benign, malignant, and unspecified (incl cysts and polyps)
Nervous system disorders
Psychiatric disorders
Renal and urinary disorders
Reproductive system and breast disorders
Respiratory, thoracic, and mediastinal disorders
Skin and subcutaneous tissue disorders
Social circumstances
Surgical and medical procedures
Vascular disorders

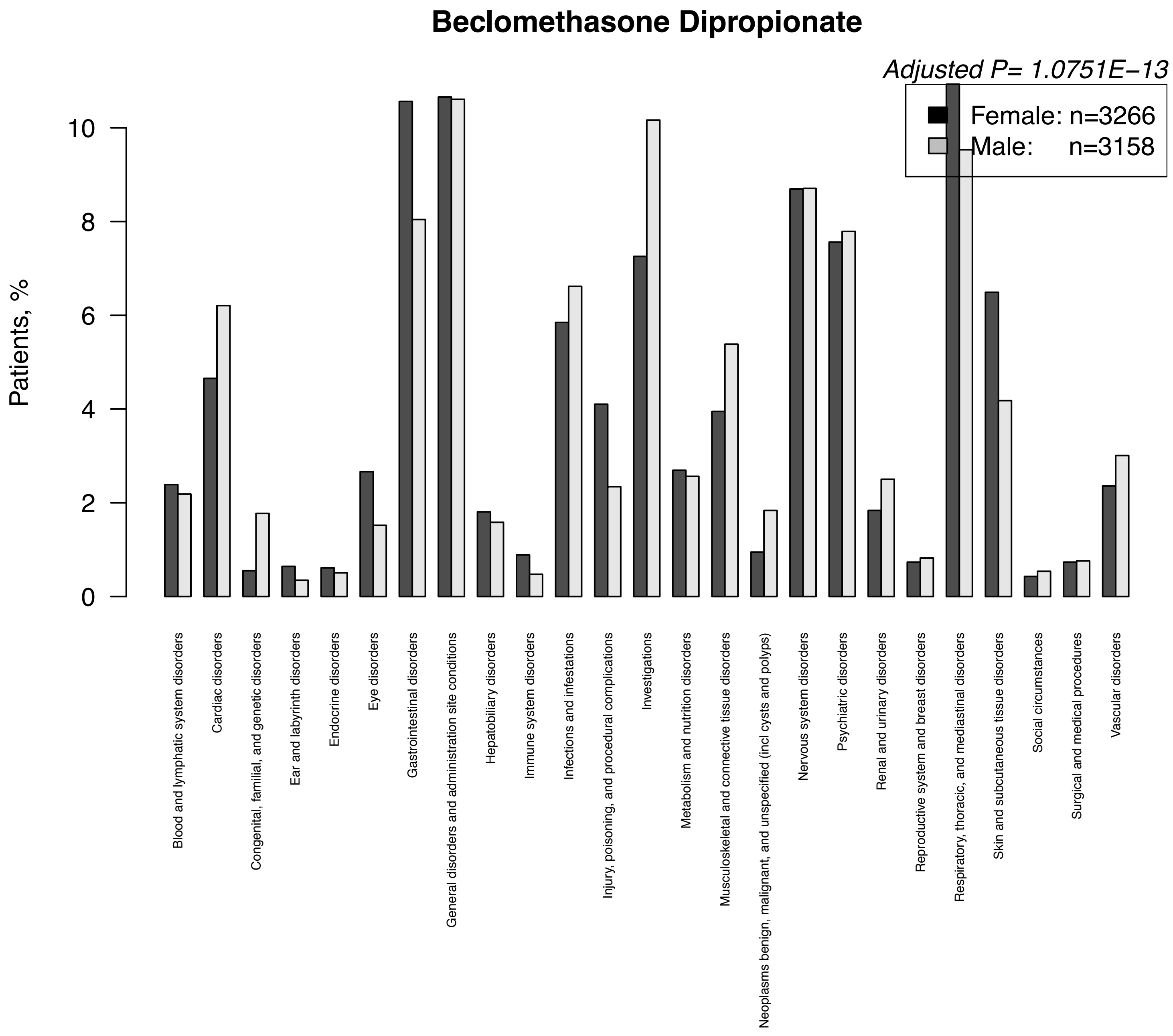

Beclomethasone Dipropionate
Adjusted P= 1.0751E−13
Female: n=3266
Male: n=3158
Patients, %
0
2
4
6
8
10
Blood and lymphatic system disorders
Cardiac disorders
Congenital, familial, and genetic disorders
Ear and labyrinth disorders
Endocrine disorders
Eye disorders
Gastrointestinal disorders
General disorders and administration site conditions
Hepatobiliary disorders
Immune system disorders
Infections and infestations
Injury, poisoning, and procedural complications
Investigations
Metabolism and nutrition disorders
Musculoskeletal and connective tissue disorders
Neoplasms benign, malignant, and unspecified (incl cysts and polyps)
Nervous system disorders
Psychiatric disorders
Renal and urinary disorders
Reproductive system and breast disorders
Respiratory, thoracic, and mediastinal disorders
Skin and subcutaneous tissue disorders
Social circumstances
Surgical and medical procedures
Vascular disorders

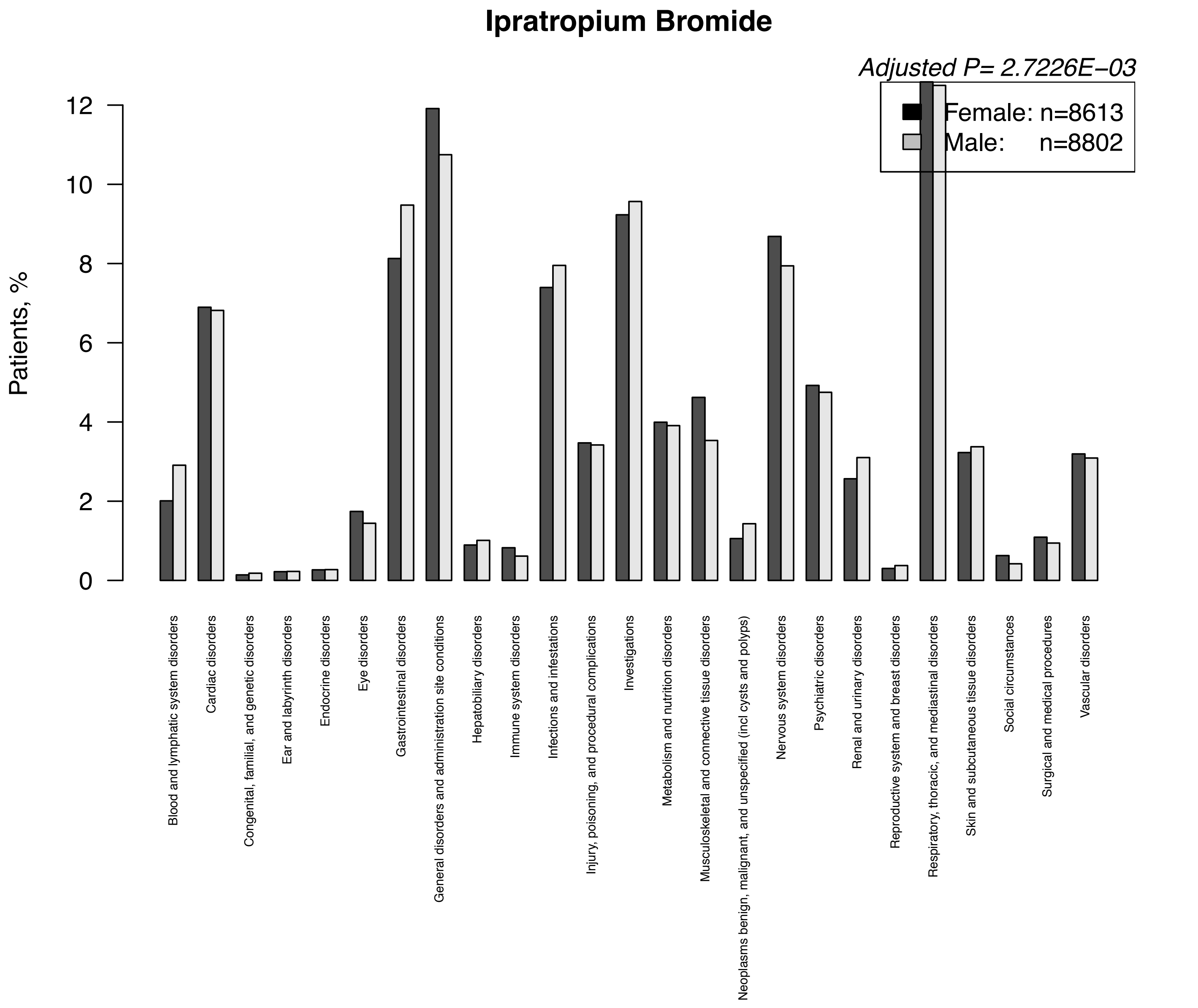

Ipratropium Bromide
Adjusted P= 2.7226E−03
Female: n=8613
Male: n=8802
Patients, %
0
2
4
6
8
10
12
Blood and lymphatic system disorders
Cardiac disorders
Congenital, familial, and genetic disorders
Ear and labyrinth disorders
Endocrine disorders
Eye disorders
Gastrointestinal disorders
General disorders and administration site conditions
Hepatobiliary disorders
Immune system disorders
Infections and infestations
Injury, poisoning, and procedural complications
Investigations
Metabolism and nutrition disorders
Musculoskeletal and connective tissue disorders
Neoplasms benign, malignant, and unspecified (incl cysts and polyps)
Nervous system disorders
Psychiatric disorders
Renal and urinary disorders
Reproductive system and breast disorders
Respiratory, thoracic, and mediastinal disorders
Skin and subcutaneous tissue disorders
Social circumstances
Surgical and medical procedures
Vascular disorders

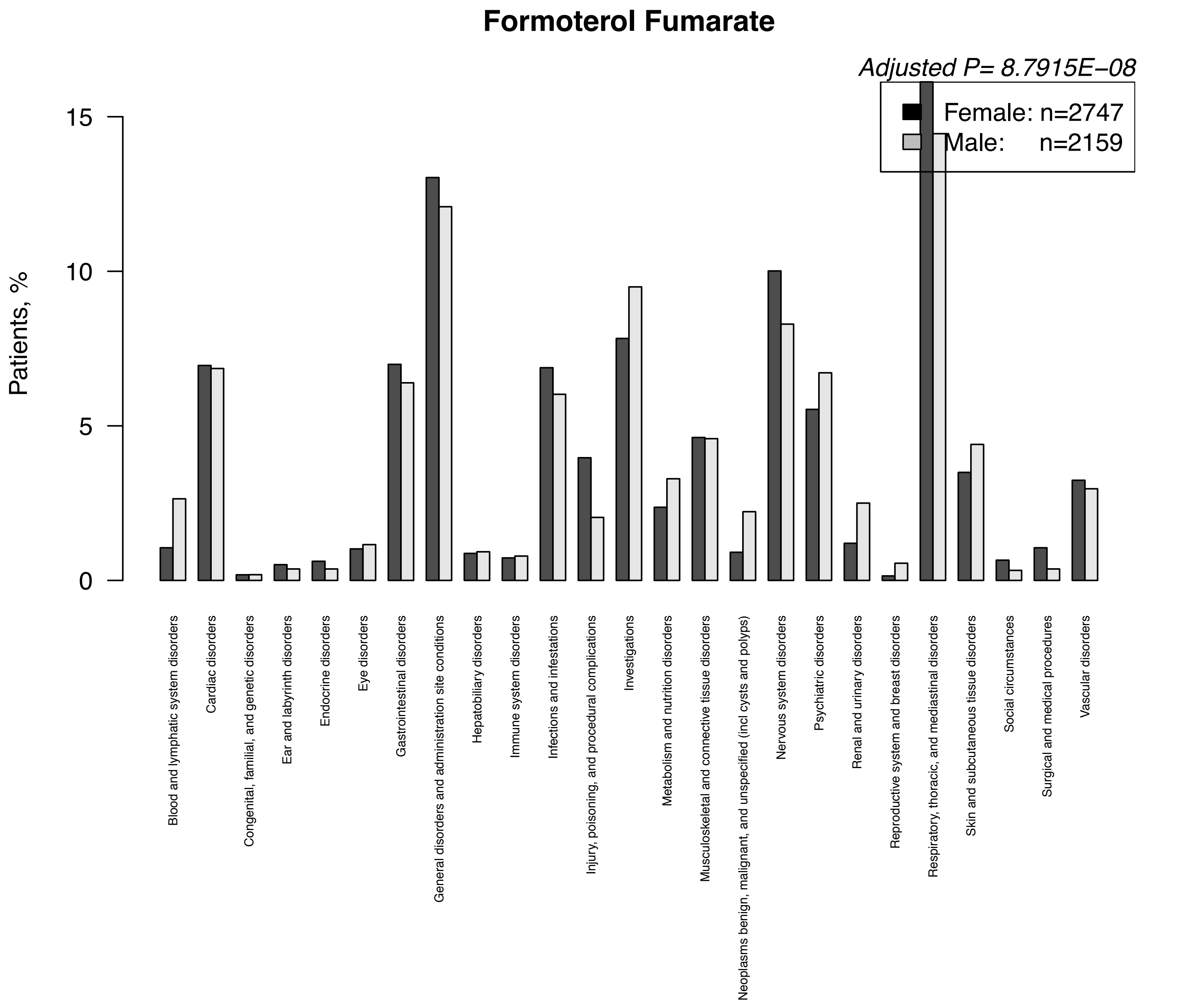

Formoterol Fumarate
Adjusted P= 8.7915E−08
Female: n=2747
Male: n=2159
Patients, %
15
10
5
0
Blood and lymphatic system disorders
Cardiac disorders
Congenital, familial, and genetic disorders
Ear and labyrinth disorders
Endocrine disorders
Eye disorders
Gastrointestinal disorders
General disorders and administration site conditions
Hepatobiliary disorders
Immune system disorders
Infections and infestations
Injury, poisoning, and procedural complications
Investigations
Metabolism and nutrition disorders
Musculoskeletal and connective tissue disorders
Neoplasms benign, malignant, and unspecified (incl cysts and polyps)
Nervous system disorders
Psychiatric disorders
Renal and urinary disorders
Reproductive system and breast disorders
Respiratory, thoracic, and mediastinal disorders
Skin and subcutaneous tissue disorders
Social circumstances
Surgical and medical procedures
Vascular disorders

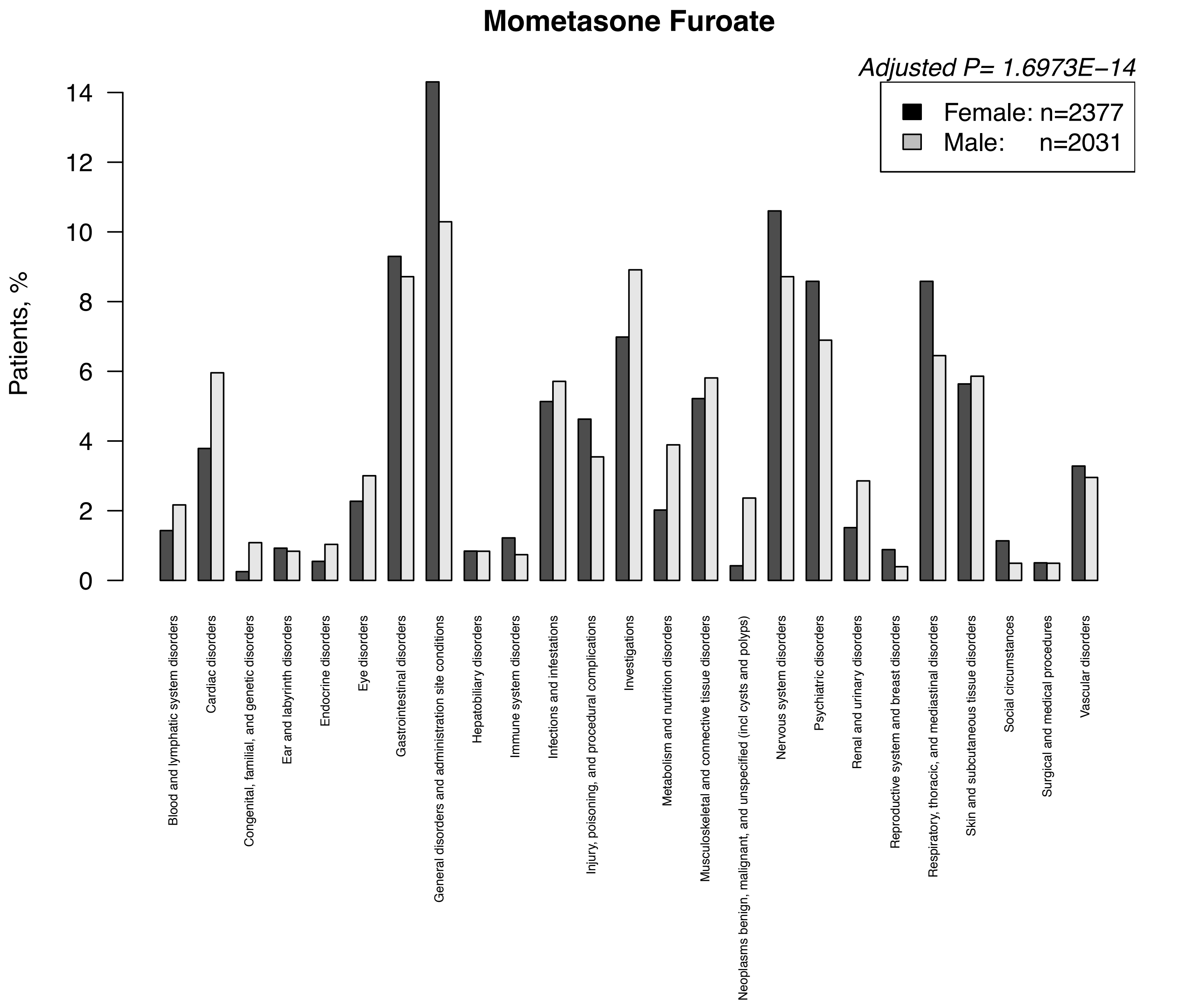
Mometasone Furoate
Adjusted P= 1.6973E−14
Female: n=2377
Male: n=2031
Patients, %
0
2
4
6
8
10
12
14
Blood and lymphatic system disorders
Cardiac disorders
Congenital, familial, and genetic disorders
Ear and labyrinth disorders
Endocrine disorders
Eye disorders
Gastrointestinal disorders
General disorders and administration site conditions
Hepatobiliary disorders
Immune system disorders
Infections and infestations
Injury, poisoning, and procedural complications
Investigations
Metabolism and nutrition disorders
Musculoskeletal and connective tissue disorders
Neoplasms benign, malignant, and unspecified (incl cysts and polyps)
Nervous system disorders
Psychiatric disorders
Renal and urinary disorders
Reproductive system and breast disorders
Respiratory, thoracic, and mediastinal disorders
Skin and subcutaneous tissue disorders
Social circumstances
Surgical and medical procedures
Vascular disorders

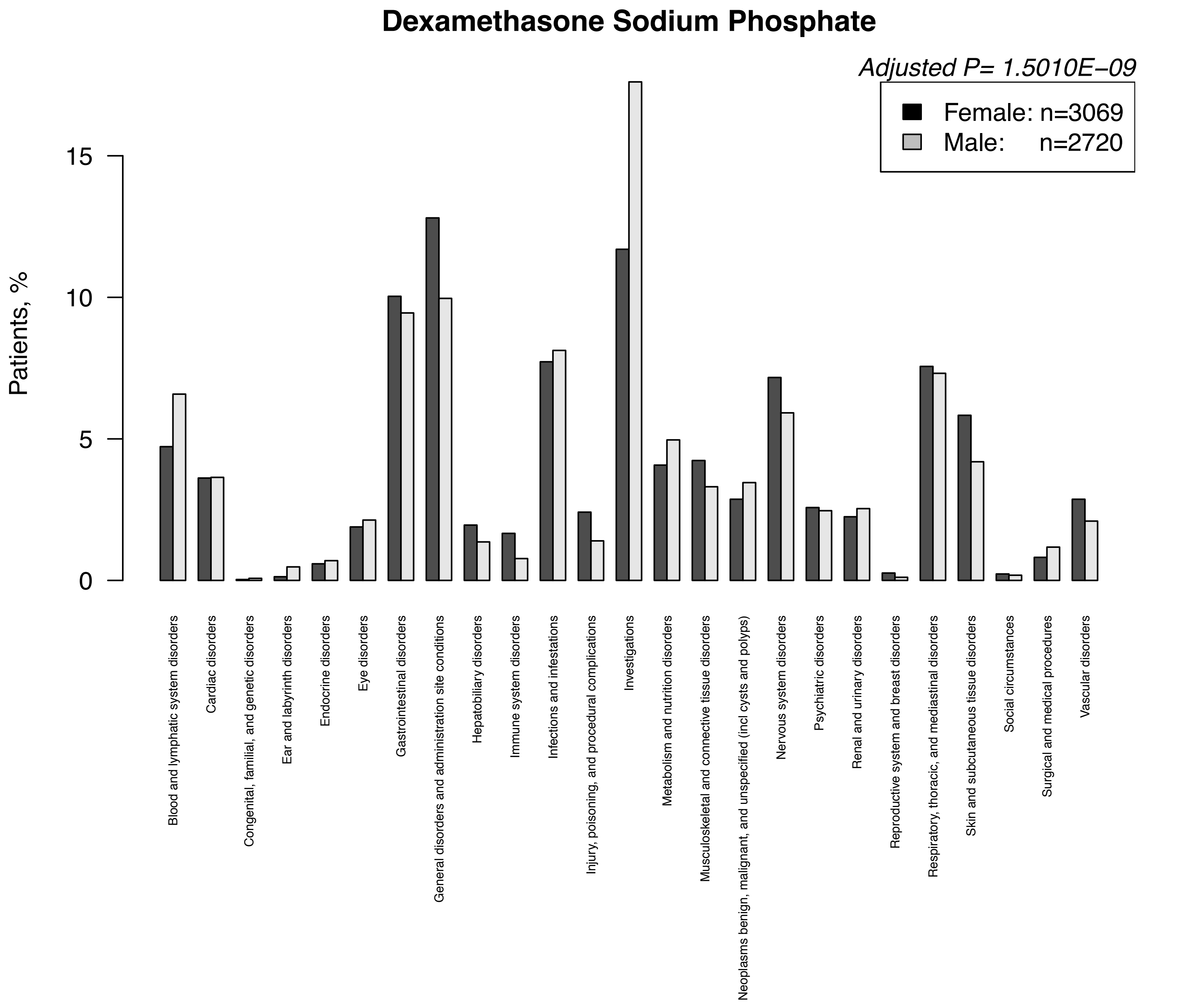
Dexamethasone Sodium Phosphate
Adjusted P= 1.5010E−09
Female: n=3069
Male: n=2720
Patients, %
0
5
10
15
Blood and lymphatic system disorders
Cardiac disorders
Congenital, familial, and genetic disorders
Ear and labyrinth disorders
Endocrine disorders
Eye disorders
Gastrointestinal disorders
General disorders and administration site conditions
Hepatobiliary disorders
Immune system disorders
Infections and infestations
Injury, poisoning, and procedural complications
Investigations
Metabolism and nutrition disorders
Musculoskeletal and connective tissue disorders
Neoplasms benign, malignant, and unspecified (incl cysts and polyps)
Nervous system disorders
Psychiatric disorders
Renal and urinary disorders
Reproductive system and breast disorders
Respiratory, thoracic, and mediastinal disorders
Skin and subcutaneous tissue disorders
Social circumstances
Surgical and medical procedures
Vascular disorders

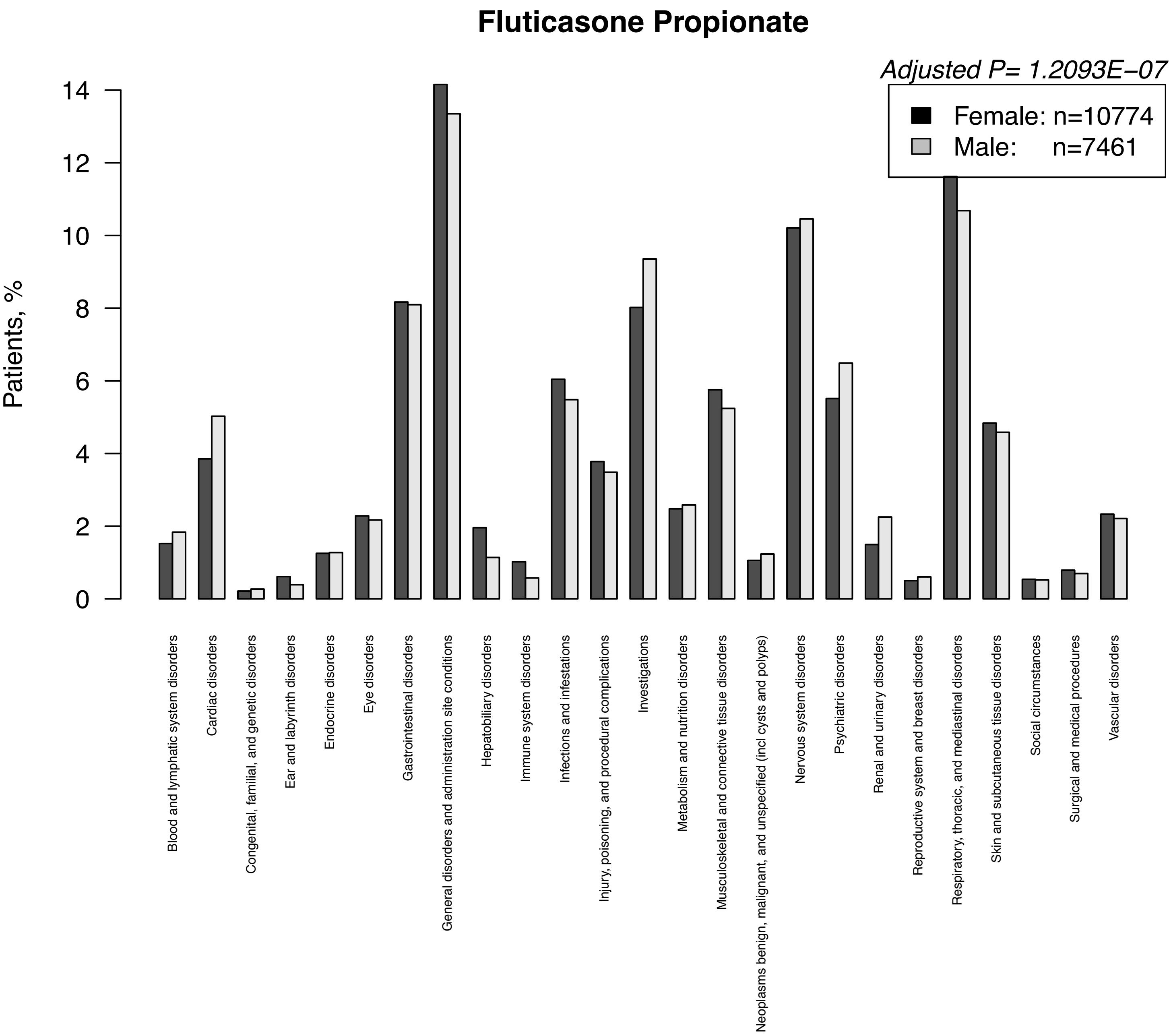
Fluticasone Propionate
Adjusted P= 1.2093E−07
Female: n=10774
Male: n=7461
Patients, %
0
2
4
6
8
10
12
14
Blood and lymphatic system disorders
Cardiac disorders
Congenital, familial, and genetic disorders
Ear and labyrinth disorders
Endocrine disorders
Eye disorders
Gastrointestinal disorders
General disorders and administration site conditions
Hepatobiliary disorders
Immune system disorders
Infections and infestations
Injury, poisoning, and procedural complications
Investigations
Metabolism and nutrition disorders
Musculoskeletal and connective tissue disorders
Neoplasms benign, malignant, and unspecified (incl cysts and polyps)
Nervous system disorders
Psychiatric disorders
Renal and urinary disorders
Reproductive system and breast disorders
Respiratory, thoracic, and mediastinal disorders
Skin and subcutaneous tissue disorders
Social circumstances
Surgical and medical procedures
Vascular disorders

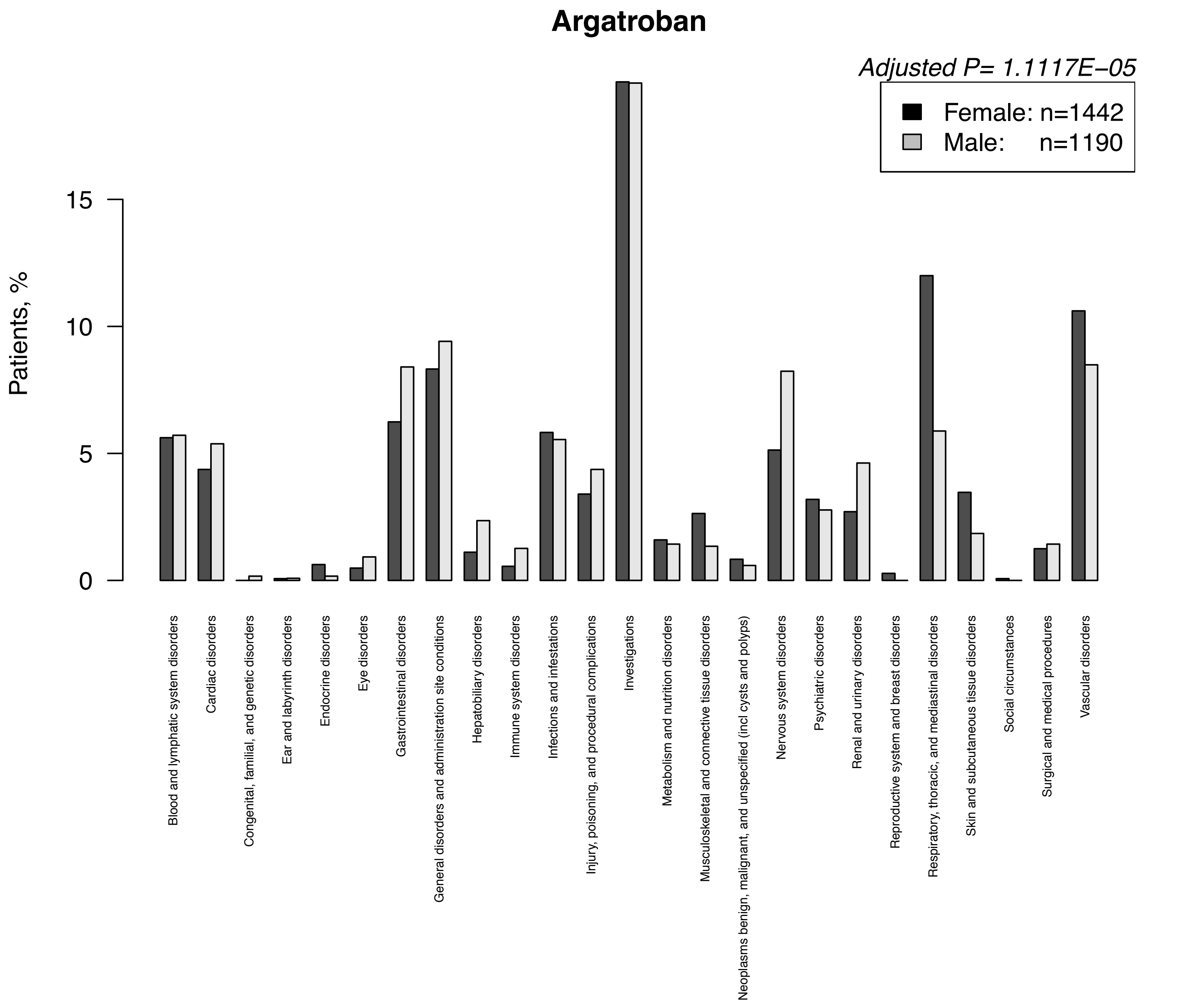
Argatroban
Adjusted P= 1.1117E−05
Female: n=1442
Male: n=1190
Patients, %
0
5
10
15
Blood and lymphatic system disorders
Cardiac disorders
Congenital, familial, and genetic disorders
Ear and labyrinth disorders
Endocrine disorders
Eye disorders
Gastrointestinal disorders
General disorders and administration site conditions
Hepatobiliary disorders
Immune system disorders
Infections and infestations
Injury, poisoning, and procedural complications
Investigations
Metabolism and nutrition disorders
Musculoskeletal and connective tissue disorders
Neoplasms benign, malignant, and unspecified (incl cysts and polyps)
Nervous system disorders
Psychiatric disorders
Renal and urinary disorders
Reproductive system and breast disorders
Respiratory, thoracic, and mediastinal disorders
Skin and subcutaneous tissue disorders
Social circumstances
Surgical and medical procedures
Vascular disorders

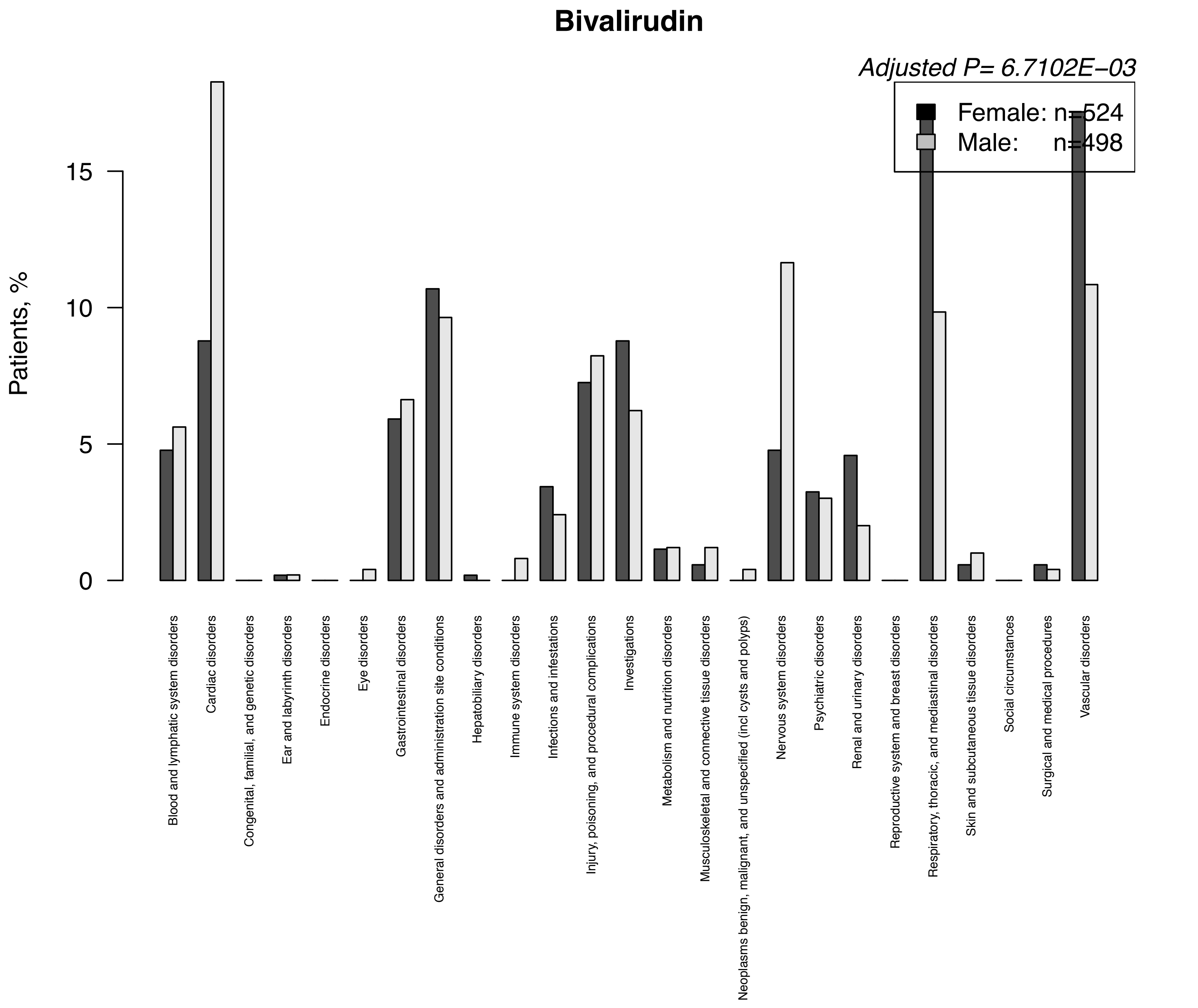
Bivalirudin
Adjusted P= 6.7102E−03
Female: n=524
Male: n=498
Patients, %
0
5
10
15
Blood and lymphatic system disorders
Cardiac disorders
Congenital, familial, and genetic disorders
Ear and labyrinth disorders
Endocrine disorders
Eye disorders
Gastrointestinal disorders
General disorders and administration site conditions
Hepatobiliary disorders
Immune system disorders
Infections and infestations
Injury, poisoning, and procedural complications
Investigations
Metabolism and nutrition disorders
Musculoskeletal and connective tissue disorders
Neoplasms benign, malignant, and unspecified (incl cysts and polyps)
Nervous system disorders
Psychiatric disorders
Renal and urinary disorders
Reproductive system and breast disorders
Respiratory, thoracic, and mediastinal disorders
Skin and subcutaneous tissue disorders
Social circumstances
Surgical and medical procedures
Vascular disorders

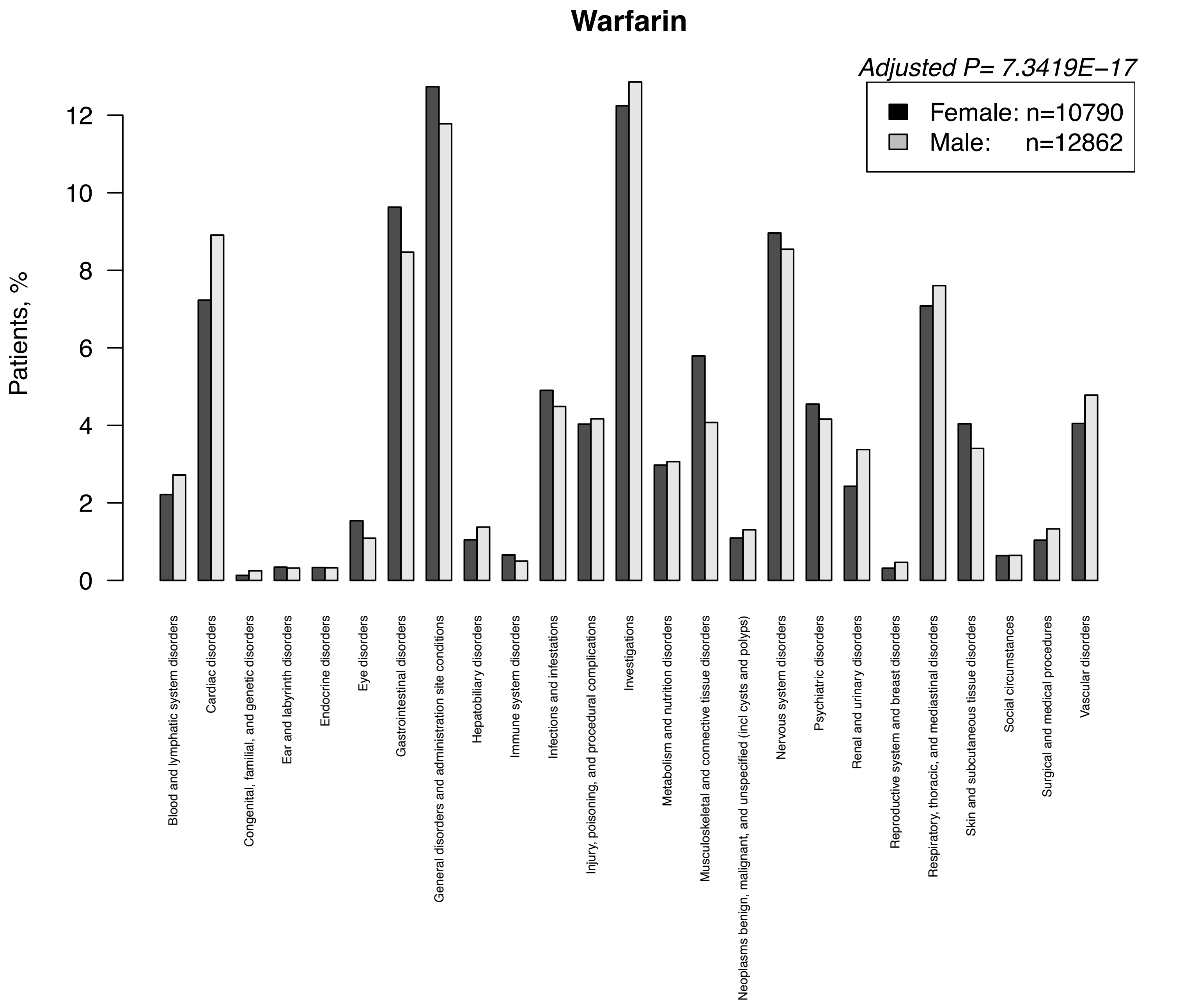
Warfarin
Adjusted P= 7.3419E−17
Female: n=10790
Male: n=12862
Patients, %
0
2
4
6
8
10
12
Blood and lymphatic system disorders
Cardiac disorders
Congenital, familial, and genetic disorders
Ear and labyrinth disorders
Endocrine disorders
Eye disorders
Gastrointestinal disorders
General disorders and administration site conditions
Hepatobiliary disorders
Immune system disorders
Infections and infestations
Injury, poisoning, and procedural complications
Investigations
Metabolism and nutrition disorders
Musculoskeletal and connective tissue disorders
Neoplasms benign, malignant, and unspecified (incl cysts and polyps)
Nervous system disorders
Psychiatric disorders
Renal and urinary disorders
Reproductive system and breast disorders
Respiratory, thoracic, and mediastinal disorders
Skin and subcutaneous tissue disorders
Social circumstances
Surgical and medical procedures
Vascular disorders

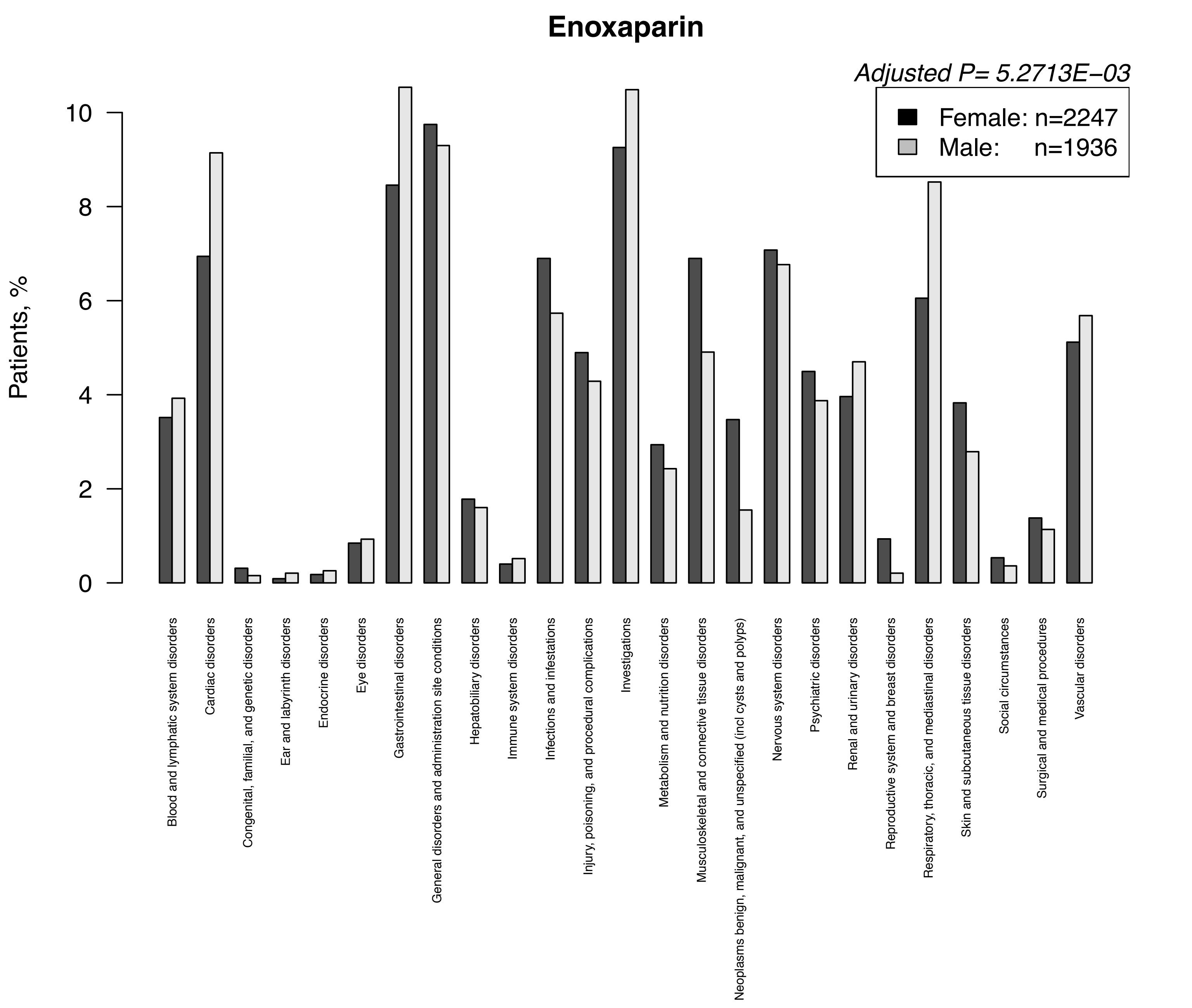

Enoxaparin
Adjusted P= 5.2713E−03
Female: n=2247
Male: n=1936
Patients, %
0
2
4
6
8
10
Blood and lymphatic system disorders
Cardiac disorders
Congenital, familial, and genetic disorders
Ear and labyrinth disorders
Endocrine disorders
Eye disorders
Gastrointestinal disorders
General disorders and administration site conditions
Hepatobiliary disorders
Immune system disorders
Infections and infestations
Injury, poisoning, and procedural complications
Investigations
Metabolism and nutrition disorders
Musculoskeletal and connective tissue disorders
Neoplasms benign, malignant, and unspecified (incl cysts and polyps)
Nervous system disorders
Psychiatric disorders
Renal and urinary disorders
Reproductive system and breast disorders
Respiratory, thoracic, and mediastinal disorders
Skin and subcutaneous tissue disorders
Social circumstances
Surgical and medical procedures
Vascular disorders

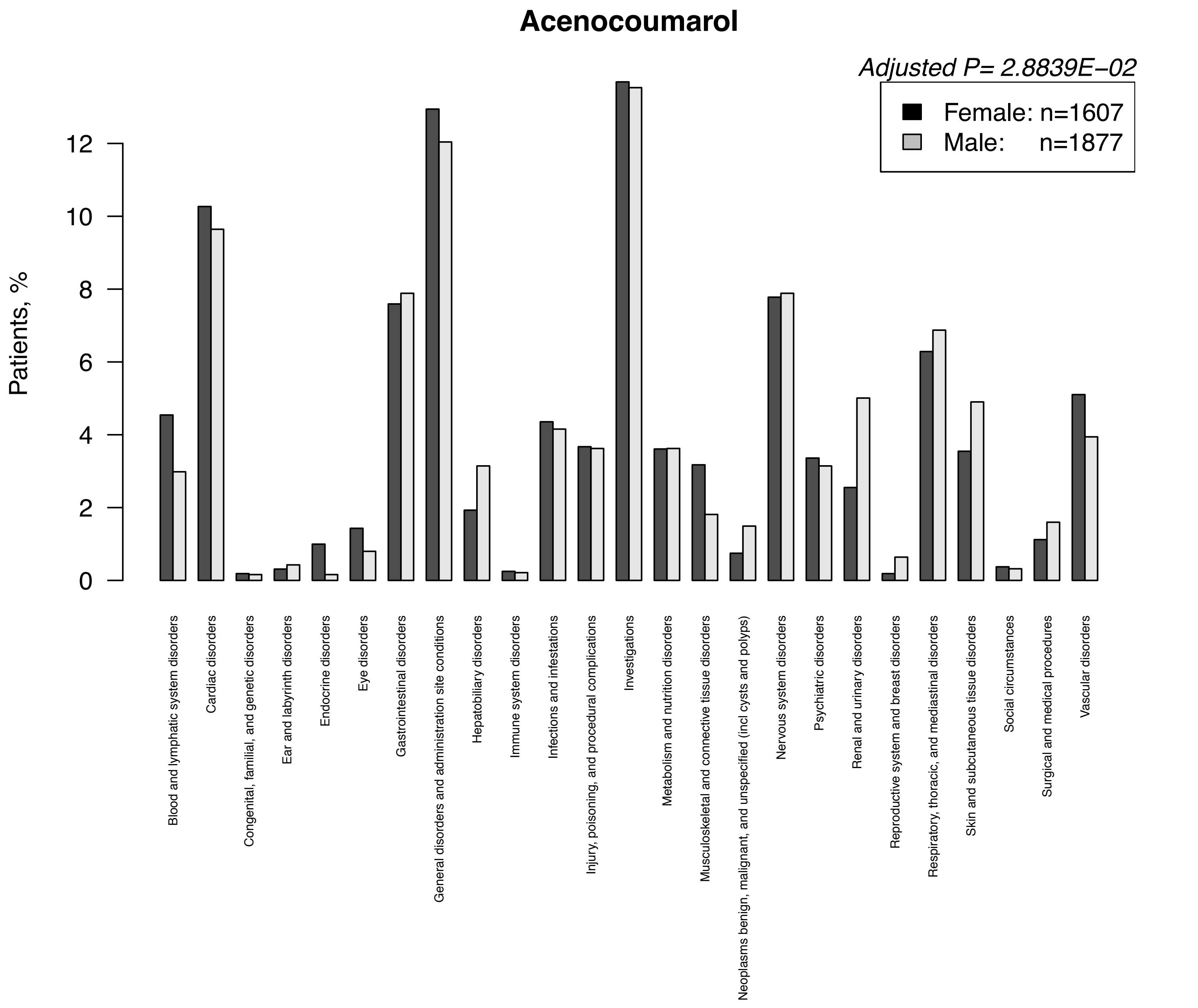
Acenocoumarol
Adjusted P= 2.8839E−02
Female: n=1607
Male: n=1877
Patients, %
0
2
4
6
8
10
12
Blood and lymphatic system disorders
Cardiac disorders
Congenital, familial, and genetic disorders
Ear and labyrinth disorders
Endocrine disorders
Eye disorders
Gastrointestinal disorders
General disorders and administration site conditions
Hepatobiliary disorders
Immune system disorders
Infections and infestations
Injury, poisoning, and procedural complications
Investigations
Metabolism and nutrition disorders
Musculoskeletal and connective tissue disorders
Neoplasms benign, malignant, and unspecified (incl cysts and polyps)
Nervous system disorders
Psychiatric disorders
Renal and urinary disorders
Reproductive system and breast disorders
Respiratory, thoracic, and mediastinal disorders
Skin and subcutaneous tissue disorders
Social circumstances
Surgical and medical procedures
Vascular disorders

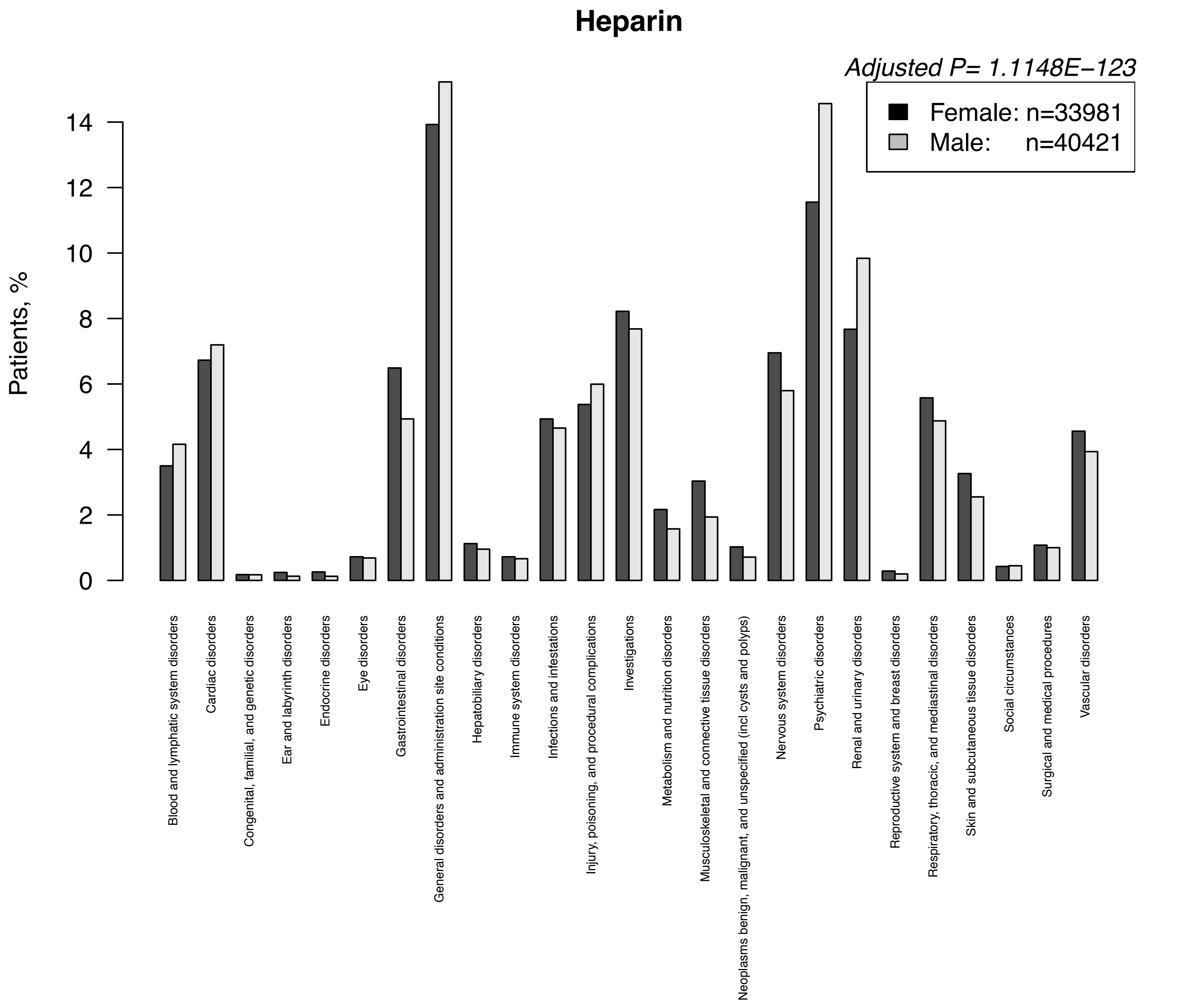

Heparin
Adjusted P= 1.1148E−123
Female: n=33981
Male: n=40421
Patients, %
0
2
4
6
8
10
12
14
Blood and lymphatic system disorders
Cardiac disorders
Congenital, familial, and genetic disorders
Ear and labyrinth disorders
Endocrine disorders
Eye disorders
Gastrointestinal disorders
General disorders and administration site conditions
Hepatobiliary disorders
Immune system disorders
Infections and infestations
Injury, poisoning, and procedural complications
Investigations
Metabolism and nutrition disorders
Musculoskeletal and connective tissue disorders
Neoplasms benign, malignant, and unspecified (incl cysts and polyps)
Nervous system disorders
Psychiatric disorders
Renal and urinary disorders
Reproductive system and breast disorders
Respiratory, thoracic, and mediastinal disorders
Skin and subcutaneous tissue disorders
Social circumstances
Surgical and medical procedures
Vascular disorders

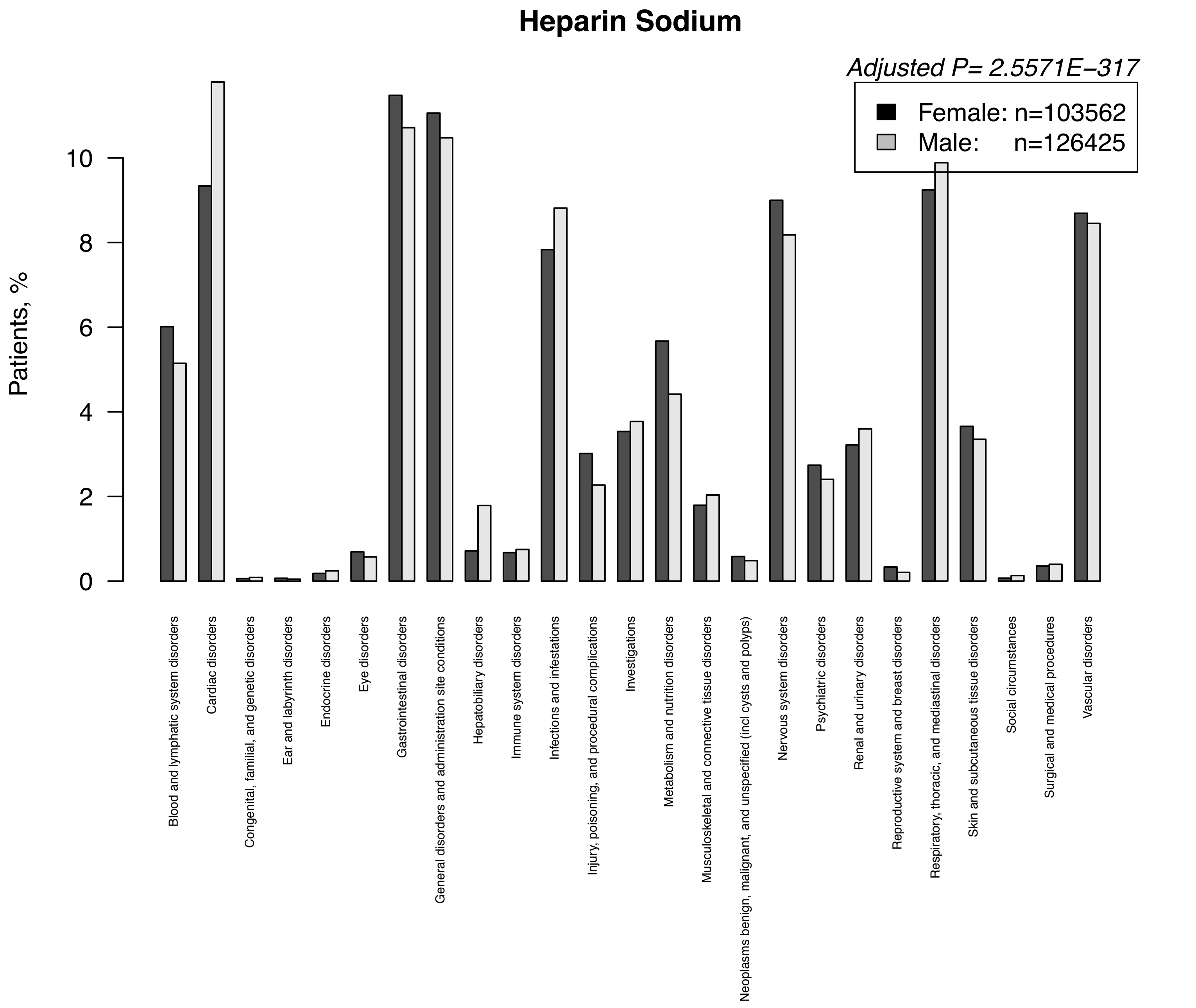
Heparin Sodium
Adjusted P= 2.5571E−317
Female: n=103562
Male: n=126425
Patients, %
0
2
4
6
8
10
Blood and lymphatic system disorders
Cardiac disorders
Congenital, familial, and genetic disorders
Ear and labyrinth disorders
Endocrine disorders
Eye disorders
Gastrointestinal disorders
General disorders and administration site conditions
Hepatobiliary disorders
Immune system disorders
Infections and infestations
Injury, poisoning, and procedural complications
Investigations
Metabolism and nutrition disorders
Musculoskeletal and connective tissue disorders
Neoplasms benign, malignant, and unspecified (incl cysts and polyps)
Nervous system disorders
Psychiatric disorders
Renal and urinary disorders
Reproductive system and breast disorders
Respiratory, thoracic, and mediastinal disorders
Skin and subcutaneous tissue disorders
Social circumstances
Surgical and medical procedures
Vascular disorders

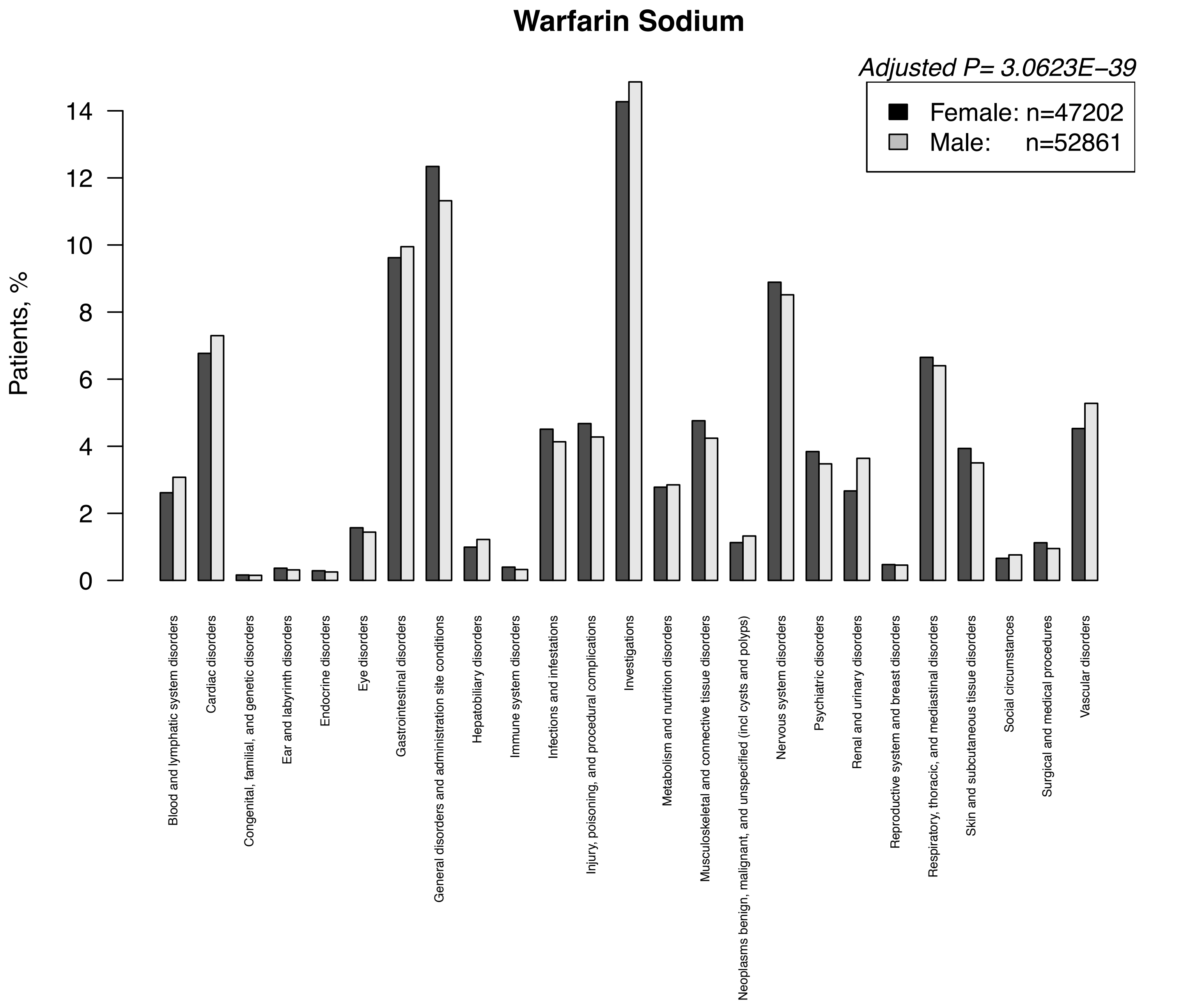

Warfarin Sodium
Adjusted P= 3.0623E−39
Female: n=47202
Male: n=52861
Patients, %
0
2
4
6
8
10
12
14
Blood and lymphatic system disorders
Cardiac disorders
Congenital, familial, and genetic disorders
Ear and labyrinth disorders
Endocrine disorders
Eye disorders
Gastrointestinal disorders
General disorders and administration site conditions
Hepatobiliary disorders
Immune system disorders
Infections and infestations
Injury, poisoning, and procedural complications
Investigations
Metabolism and nutrition disorders
Musculoskeletal and connective tissue disorders
Neoplasms benign, malignant, and unspecified (incl cysts and polyps)
Nervous system disorders
Psychiatric disorders
Renal and urinary disorders
Reproductive system and breast disorders
Respiratory, thoracic, and mediastinal disorders
Skin and subcutaneous tissue disorders
Social circumstances
Surgical and medical procedures
Vascular disorders

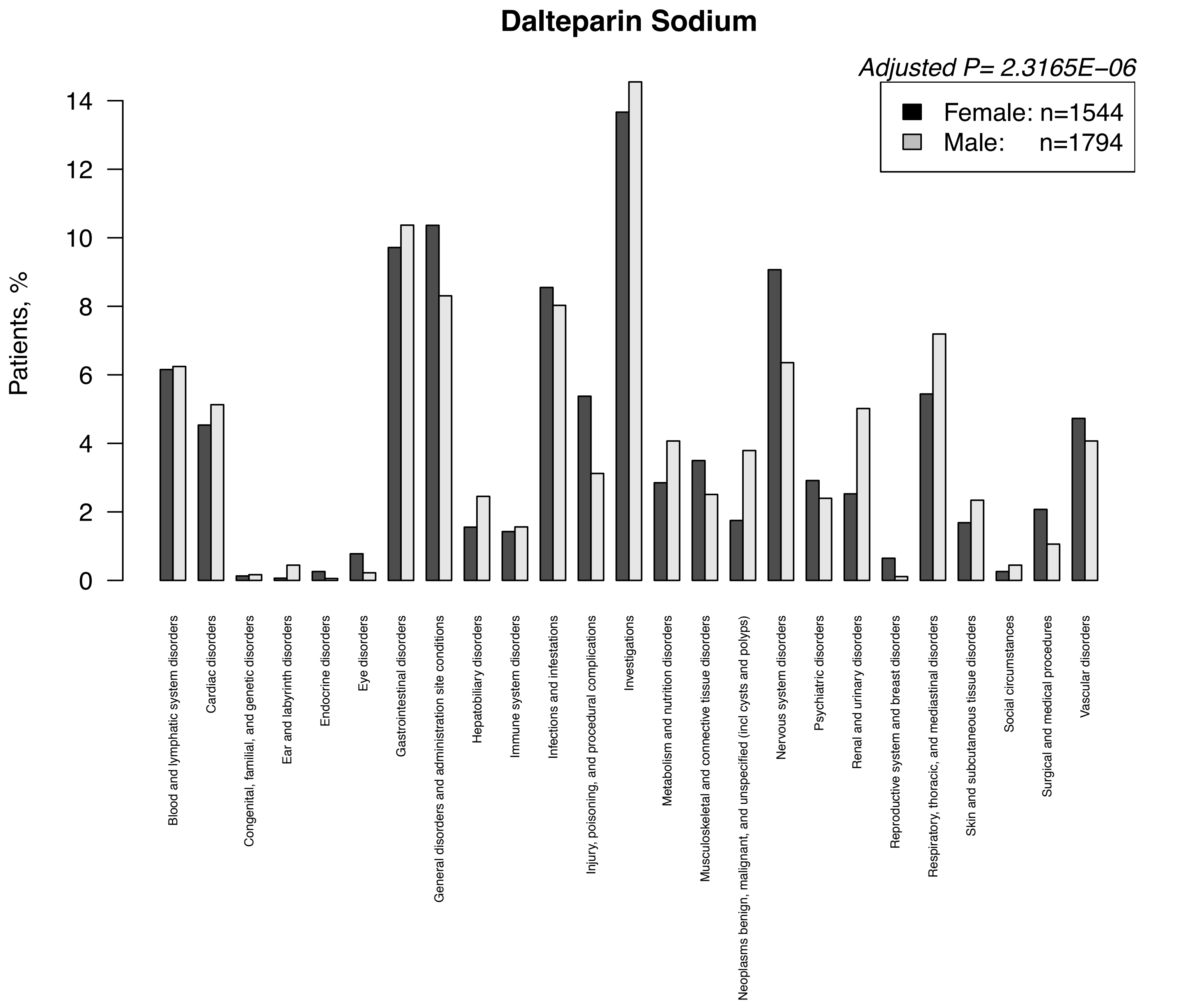

Dalteparin Sodium
Adjusted P= 2.3165E−06
Female: n=1544
Male: n=1794
Patients, %
0
2
4
6
8
10
12
14
Blood and lymphatic system disorders
Cardiac disorders
Congenital, familial, and genetic disorders
Ear and labyrinth disorders
Endocrine disorders
Eye disorders
Gastrointestinal disorders
General disorders and administration site conditions
Hepatobiliary disorders
Immune system disorders
Infections and infestations
Injury, poisoning, and procedural complications
Investigations
Metabolism and nutrition disorders
Musculoskeletal and connective tissue disorders
Neoplasms benign, malignant, and unspecified (incl cysts and polyps)
Nervous system disorders
Psychiatric disorders
Renal and urinary disorders
Reproductive system and breast disorders
Respiratory, thoracic, and mediastinal disorders
Skin and subcutaneous tissue disorders
Social circumstances
Surgical and medical procedures
Vascular disorders

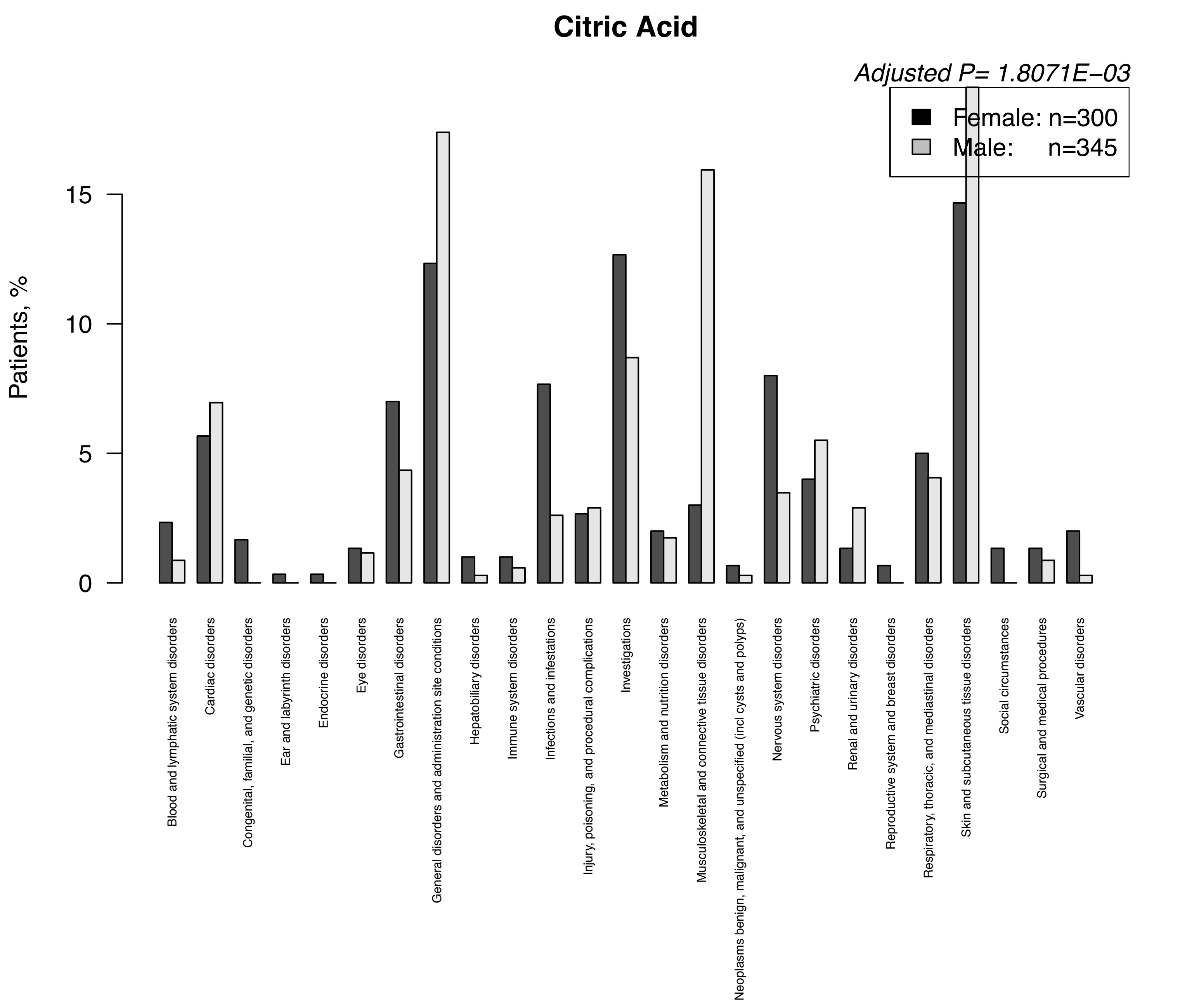

Citric Acid
Adjusted P= 1.8071E−03
Female: n=300
Male: n=345
Patients, %
0
5
10
15
Blood and lymphatic system disorders
Cardiac disorders
Congenital, familial, and genetic disorders
Ear and labyrinth disorders
Endocrine disorders
Eye disorders
Gastrointestinal disorders
General disorders and administration site conditions
Hepatobiliary disorders
Immune system disorders
Infections and infestations
Injury, poisoning, and procedural complications
Investigations
Metabolism and nutrition disorders
Musculoskeletal and connective tissue disorders
Neoplasms benign, malignant, and unspecified (incl cysts and polyps)
Nervous system disorders
Psychiatric disorders
Renal and urinary disorders
Reproductive system and breast disorders
Respiratory, thoracic, and mediastinal disorders
Skin and subcutaneous tissue disorders
Social circumstances
Surgical and medical procedures
Vascular disorders

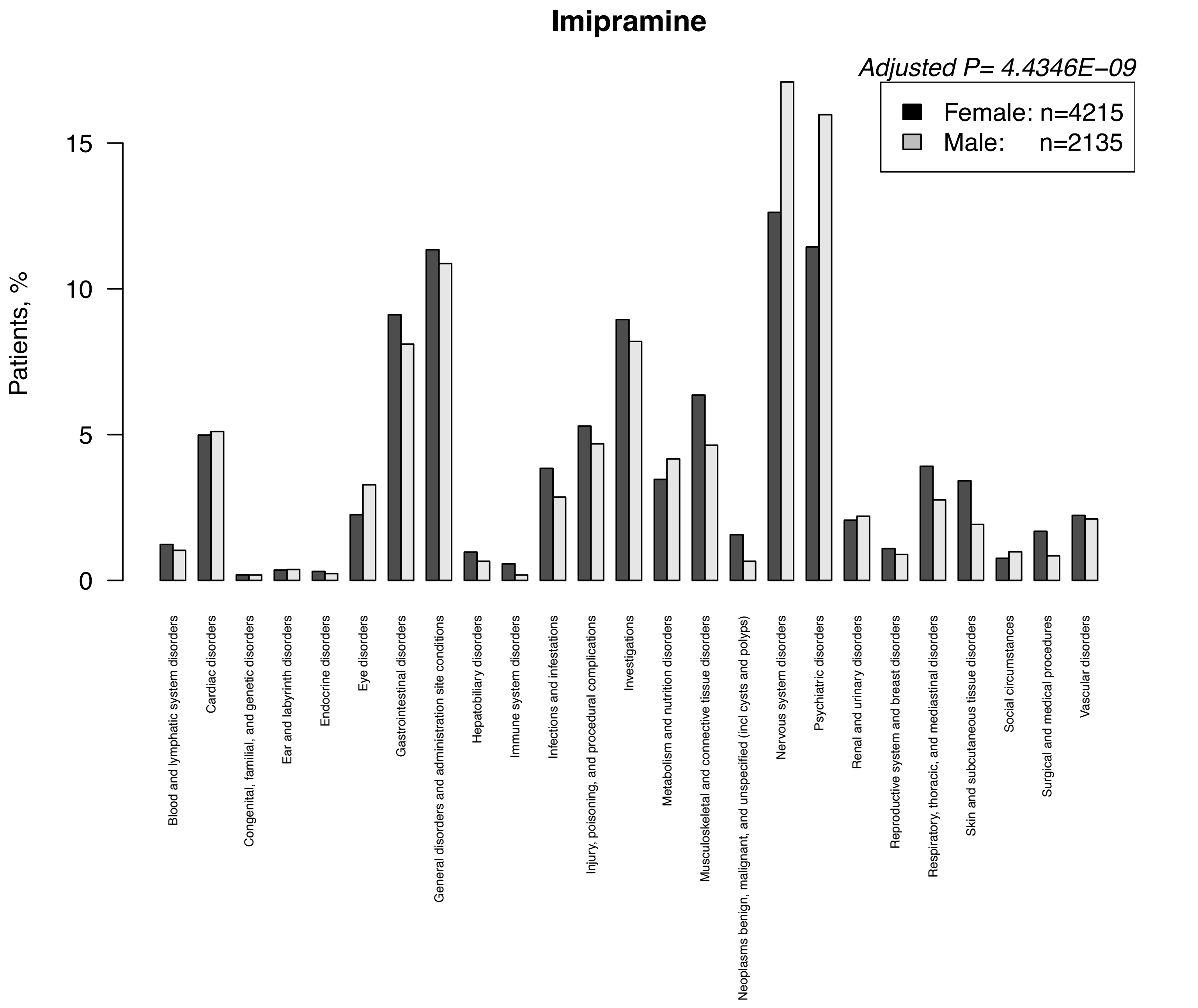
Imipramine
Adjusted P= 4.4346E−09
Female: n=4215
Male: n=2135
Patients, %
0
5
10
15
Blood and lymphatic system disorders
Cardiac disorders
Congenital, familial, and genetic disorders
Ear and labyrinth disorders
Endocrine disorders
Eye disorders
Gastrointestinal disorders
General disorders and administration site conditions
Hepatobiliary disorders
Immune system disorders
Infections and infestations
Injury, poisoning, and procedural complications
Investigations
Metabolism and nutrition disorders
Musculoskeletal and connective tissue disorders
Neoplasms benign, malignant, and unspecified (incl cysts and polyps)
Nervous system disorders
Psychiatric disorders
Renal and urinary disorders
Reproductive system and breast disorders
Respiratory, thoracic, and mediastinal disorders
Skin and subcutaneous tissue disorders
Social circumstances
Surgical and medical procedures
Vascular disorders

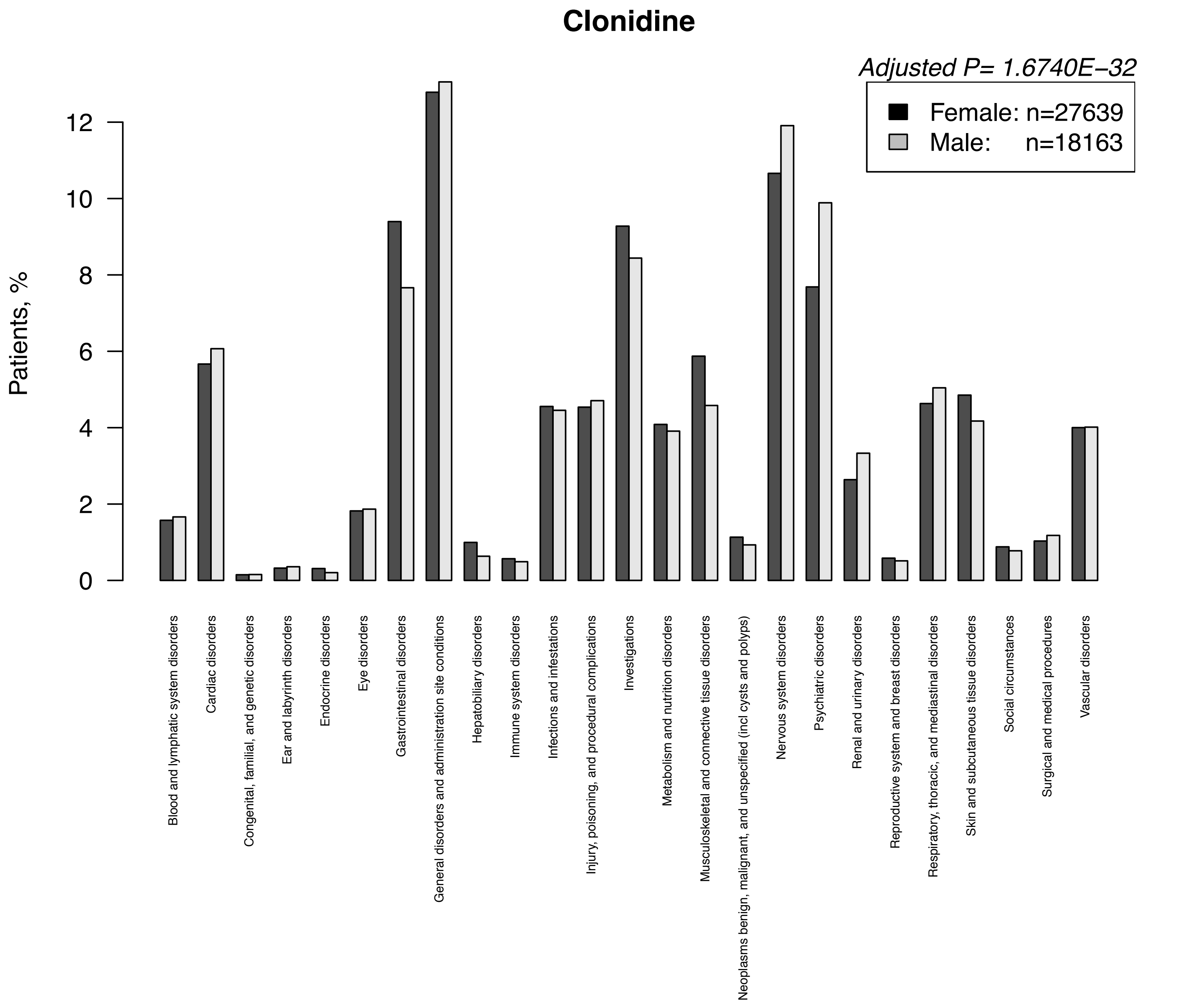
Clonidine
Adjusted P= 1.6740E−32
Female: n=27639
Male: n=18163
Patients, %
0
2
4
6
8
10
12
Blood and lymphatic system disorders
Cardiac disorders
Congenital, familial, and genetic disorders
Ear and labyrinth disorders
Endocrine disorders
Eye disorders
Gastrointestinal disorders
General disorders and administration site conditions
Hepatobiliary disorders
Immune system disorders
Infections and infestations
Injury, poisoning, and procedural complications
Investigations
Metabolism and nutrition disorders
Musculoskeletal and connective tissue disorders
Neoplasms benign, malignant, and unspecified (incl cysts and polyps)
Nervous system disorders
Psychiatric disorders
Renal and urinary disorders
Reproductive system and breast disorders
Respiratory, thoracic, and mediastinal disorders
Skin and subcutaneous tissue disorders
Social circumstances
Surgical and medical procedures
Vascular disorders

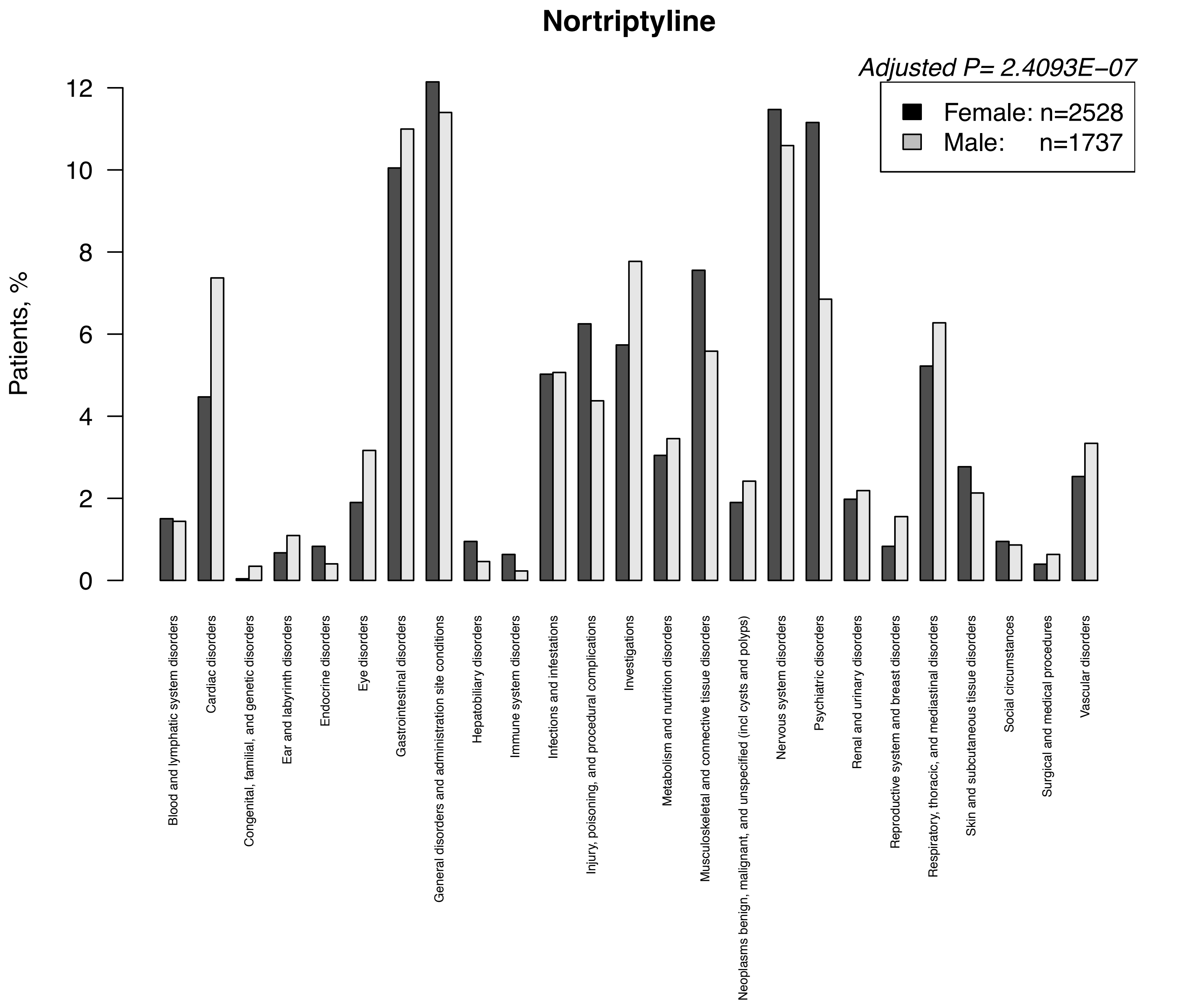

Nortriptyline
Adjusted P= 2.4093E−07
Female: n=2528
Male: n=1737
Patients, %
0
2
4
6
8
10
12
Blood and lymphatic system disorders
Cardiac disorders
Congenital, familial, and genetic disorders
Ear and labyrinth disorders
Endocrine disorders
Eye disorders
Gastrointestinal disorders
General disorders and administration site conditions
Hepatobiliary disorders
Immune system disorders
Infections and infestations
Injury, poisoning, and procedural complications
Investigations
Metabolism and nutrition disorders
Musculoskeletal and connective tissue disorders
Neoplasms benign, malignant, and unspecified (incl cysts and polyps)
Nervous system disorders
Psychiatric disorders
Renal and urinary disorders
Reproductive system and breast disorders
Respiratory, thoracic, and mediastinal disorders
Skin and subcutaneous tissue disorders
Social circumstances
Surgical and medical procedures
Vascular disorders

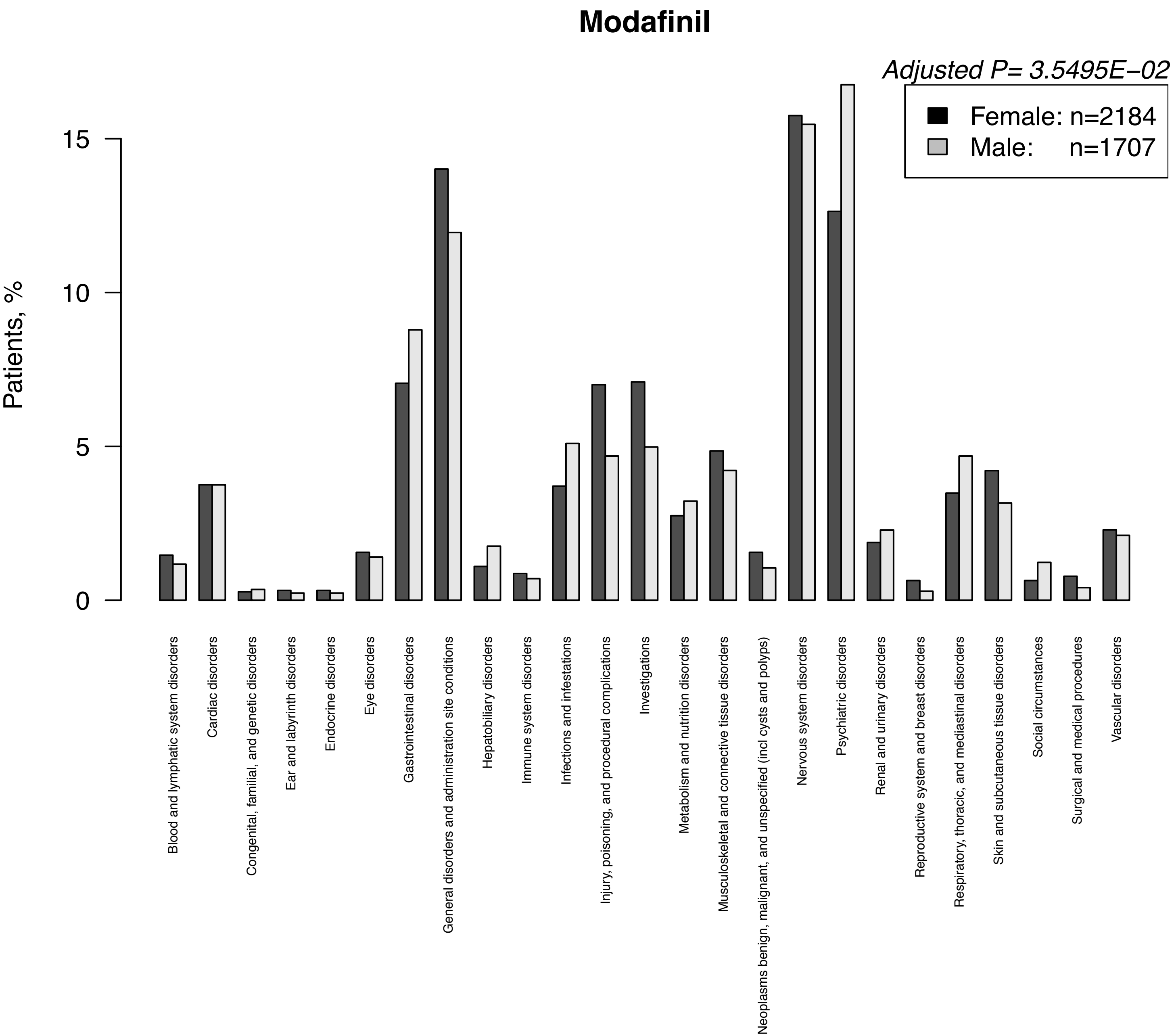
Modafinil
Adjusted P= 3.5495E−02
Female: n=2184
Male: n=1707
Patients, %
0
5
10
15
Blood and lymphatic system disorders
Cardiac disorders
Congenital, familial, and genetic disorders
Ear and labyrinth disorders
Endocrine disorders
Eye disorders
Gastrointestinal disorders
General disorders and administration site conditions
Hepatobiliary disorders
Immune system disorders
Infections and infestations
Injury, poisoning, and procedural complications
Investigations
Metabolism and nutrition disorders
Musculoskeletal and connective tissue disorders
Neoplasms benign, malignant, and unspecified (incl cysts and polyps)
Nervous system disorders
Psychiatric disorders
Renal and urinary disorders
Reproductive system and breast disorders
Respiratory, thoracic, and mediastinal disorders
Skin and subcutaneous tissue disorders
Social circumstances
Surgical and medical procedures
Vascular disorders

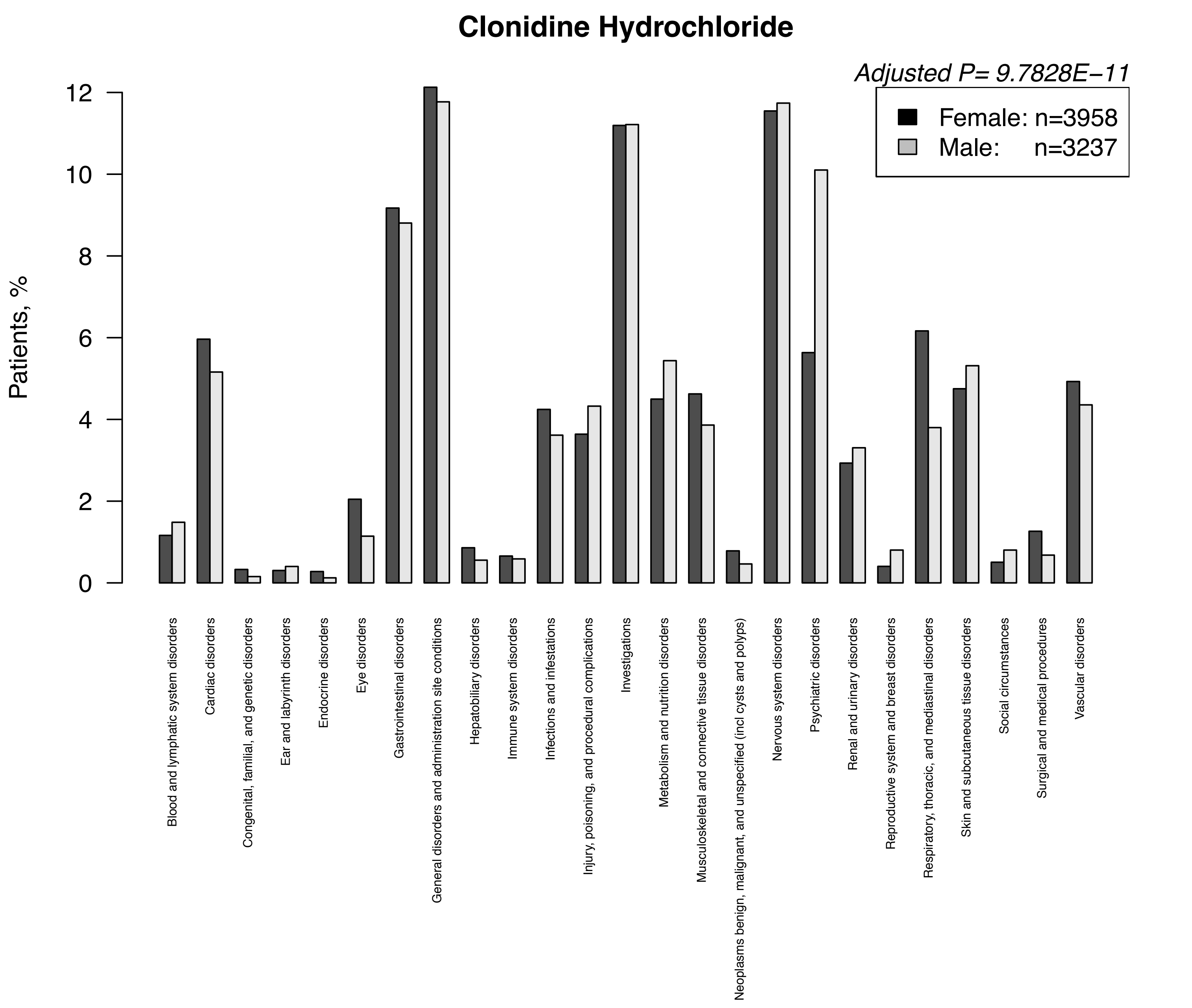

Clonidine Hydrochloride
Adjusted P= 9.7828E−11
Female: n=3958
Male: n=3237
Patients, %
0
2
4
6
8
10
12
Blood and lymphatic system disorders
Cardiac disorders
Congenital, familial, and genetic disorders
Ear and labyrinth disorders
Endocrine disorders
Eye disorders
Gastrointestinal disorders
General disorders and administration site conditions
Hepatobiliary disorders
Immune system disorders
Infections and infestations
Injury, poisoning, and procedural complications
Investigations
Metabolism and nutrition disorders
Musculoskeletal and connective tissue disorders
Neoplasms benign, malignant, and unspecified (incl cysts and polyps)
Nervous system disorders
Psychiatric disorders
Renal and urinary disorders
Reproductive system and breast disorders
Respiratory, thoracic, and mediastinal disorders
Skin and subcutaneous tissue disorders
Social circumstances
Surgical and medical procedures
Vascular disorders

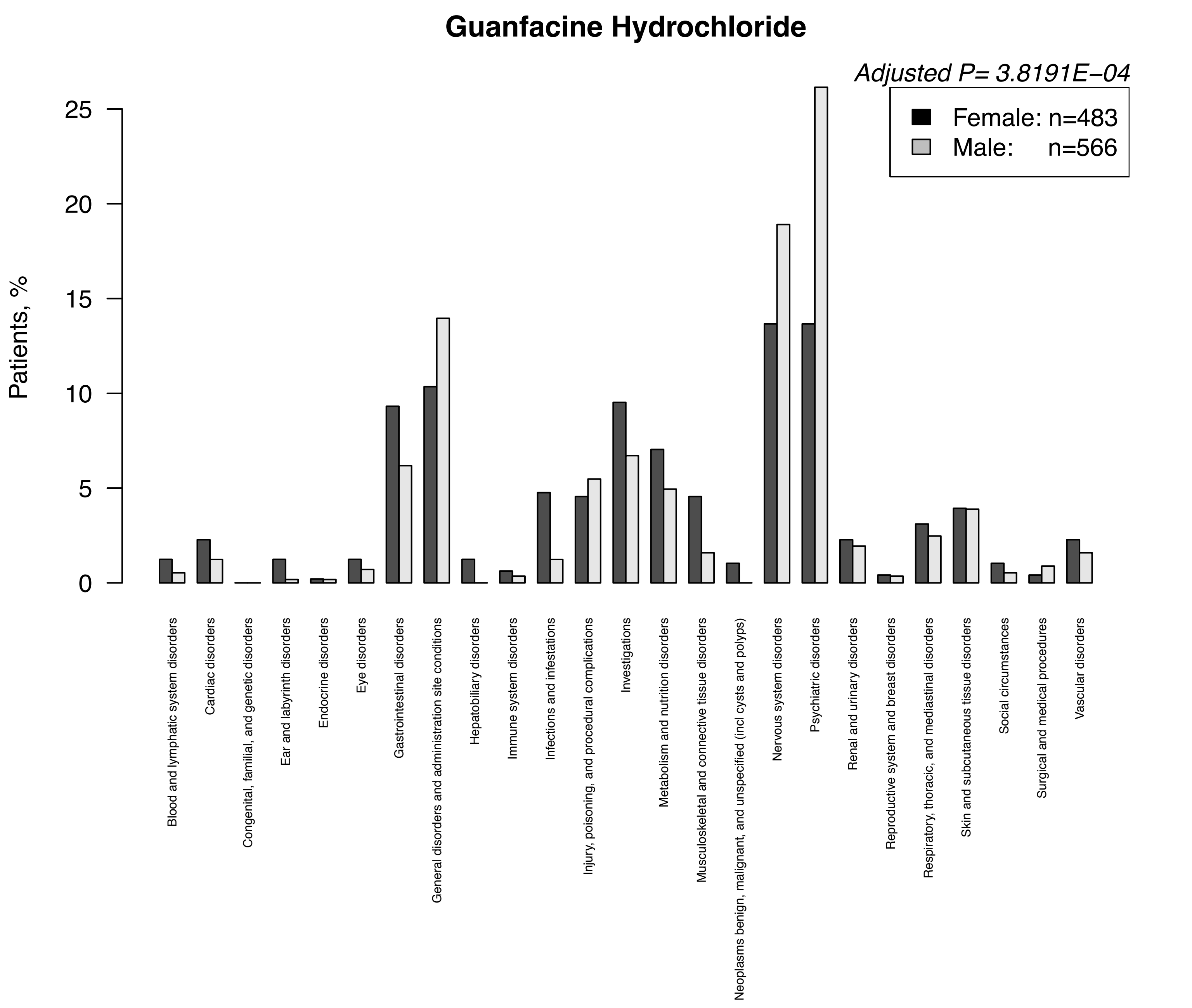

Guanfacine Hydrochloride
Adjusted P= 3.8191E−04
Female: n=483
Male: n=566
Patients, %
0
5
10
15
20
25
Blood and lymphatic system disorders
Cardiac disorders
Congenital, familial, and genetic disorders
Ear and labyrinth disorders
Endocrine disorders
Eye disorders
Gastrointestinal disorders
General disorders and administration site conditions
Hepatobiliary disorders
Immune system disorders
Infections and infestations
Injury, poisoning, and procedural complications
Investigations
Metabolism and nutrition disorders
Musculoskeletal and connective tissue disorders
Neoplasms benign, malignant, and unspecified (incl cysts and polyps)
Nervous system disorders
Psychiatric disorders
Renal and urinary disorders
Reproductive system and breast disorders
Respiratory, thoracic, and mediastinal disorders
Skin and subcutaneous tissue disorders
Social circumstances
Surgical and medical procedures
Vascular disorders

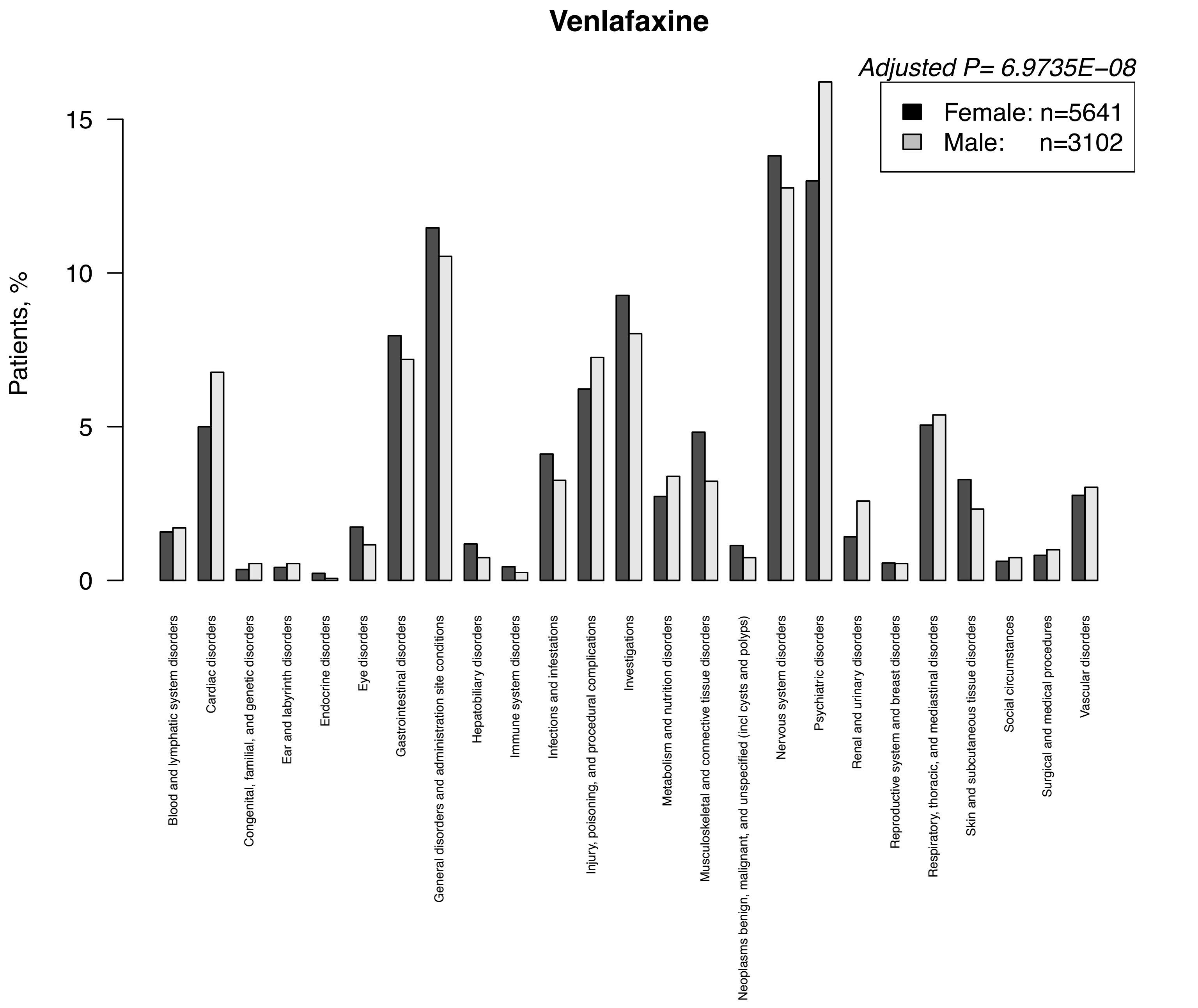

Venlafaxine
Adjusted P= 6.9735E−08
Female: n=5641
Male: n=3102
Patients, %
0
5
10
15
Blood and lymphatic system disorders
Cardiac disorders
Congenital, familial, and genetic disorders
Ear and labyrinth disorders
Endocrine disorders
Eye disorders
Gastrointestinal disorders
General disorders and administration site conditions
Hepatobiliary disorders
Immune system disorders
Infections and infestations
Injury, poisoning, and procedural complications
Investigations
Metabolism and nutrition disorders
Musculoskeletal and connective tissue disorders
Neoplasms benign, malignant, and unspecified (incl cysts and polyps)
Nervous system disorders
Psychiatric disorders
Renal and urinary disorders
Reproductive system and breast disorders
Respiratory, thoracic, and mediastinal disorders
Skin and subcutaneous tissue disorders
Social circumstances
Surgical and medical procedures
Vascular disorders

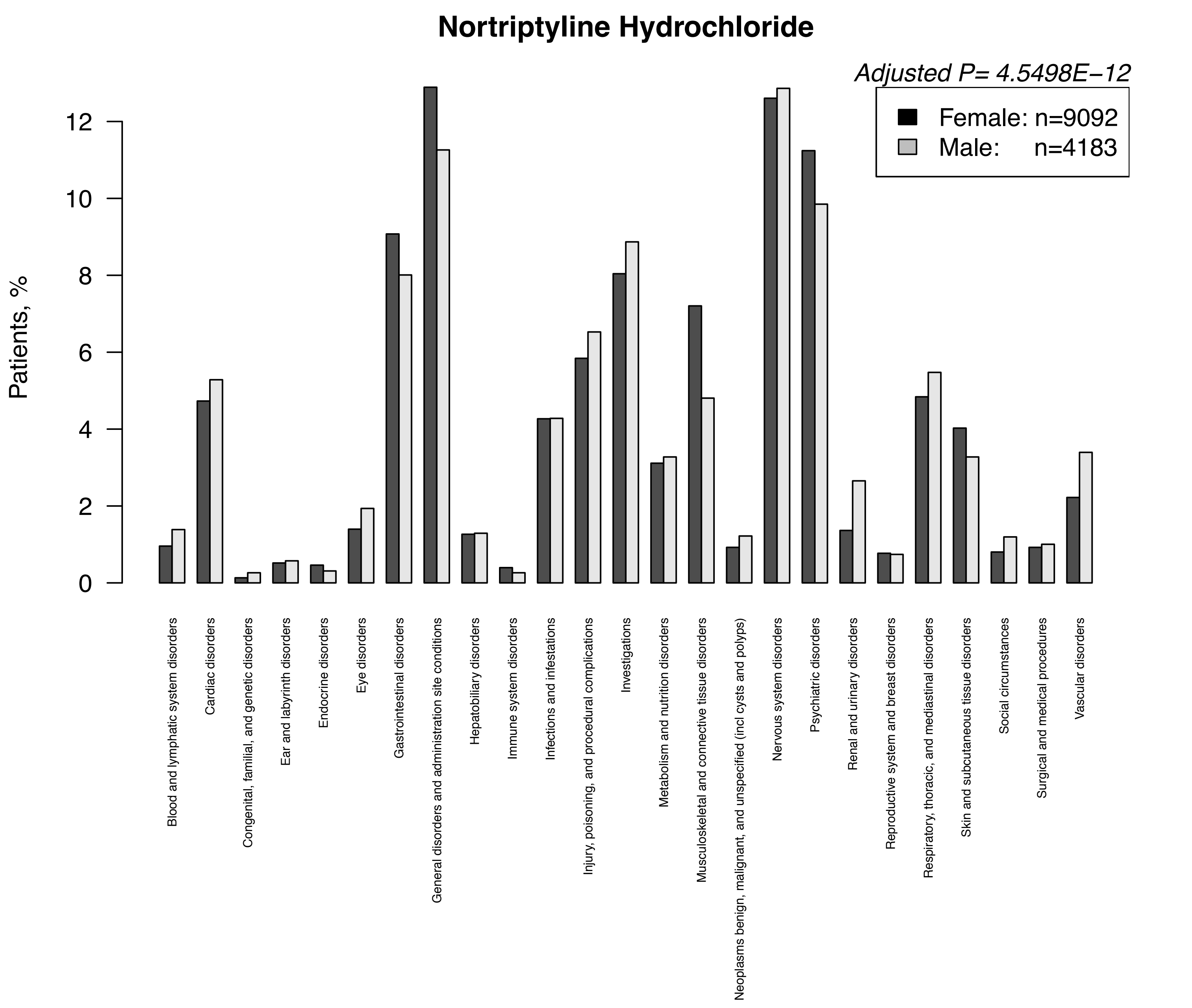
Nortriptyline Hydrochloride
Adjusted P= 4.5498E−12
Female: n=9092
Male: n=4183
Patients, %
0
2
4
6
8
10
12
Blood and lymphatic system disorders
Cardiac disorders
Congenital, familial, and genetic disorders
Ear and labyrinth disorders
Endocrine disorders
Eye disorders
Gastrointestinal disorders
General disorders and administration site conditions
Hepatobiliary disorders
Immune system disorders
Infections and infestations
Injury, poisoning, and procedural complications
Investigations
Metabolism and nutrition disorders
Musculoskeletal and connective tissue disorders
Neoplasms benign, malignant, and unspecified (incl cysts and polyps)
Nervous system disorders
Psychiatric disorders
Renal and urinary disorders
Reproductive system and breast disorders
Respiratory, thoracic, and mediastinal disorders
Skin and subcutaneous tissue disorders
Social circumstances
Surgical and medical procedures
Vascular disorders

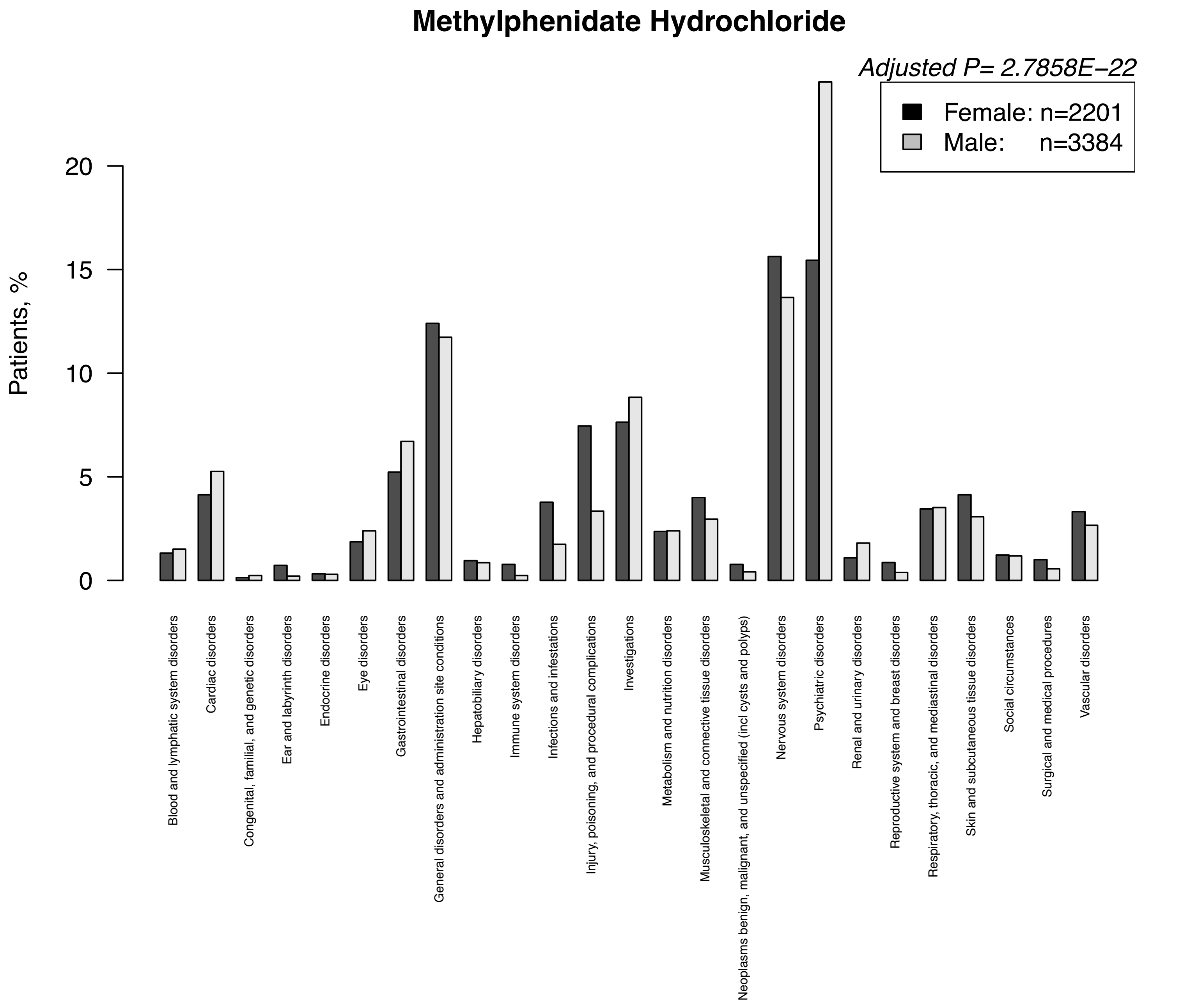

Methylphenidate Hydrochloride
Adjusted P= 2.7858E−22
Female: n=2201
Male: n=3384
Patients, %
0
5
10
15
20
Blood and lymphatic system disorders
Cardiac disorders
Congenital, familial, and genetic disorders
Ear and labyrinth disorders
Endocrine disorders
Eye disorders
Gastrointestinal disorders
General disorders and administration site conditions
Hepatobiliary disorders
Immune system disorders
Infections and infestations
Injury, poisoning, and procedural complications
Investigations
Metabolism and nutrition disorders
Musculoskeletal and connective tissue disorders
Neoplasms benign, malignant, and unspecified (incl cysts and polyps)
Nervous system disorders
Psychiatric disorders
Renal and urinary disorders
Reproductive system and breast disorders
Respiratory, thoracic, and mediastinal disorders
Skin and subcutaneous tissue disorders
Social circumstances
Surgical and medical procedures
Vascular disorders

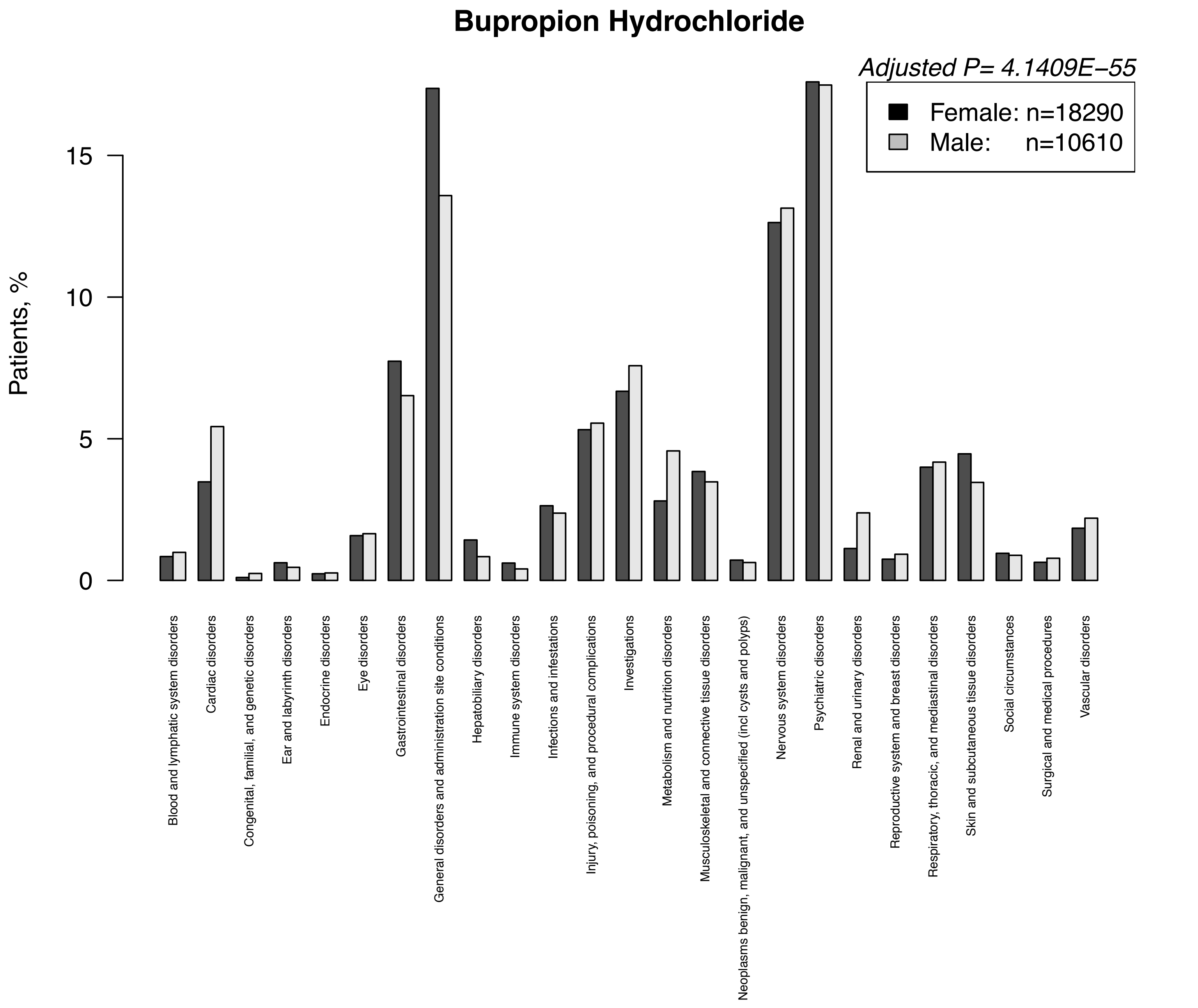

Bupropion Hydrochloride
Adjusted P= 4.1409E−55
Female: n=18290
Male: n=10610
Patients, %
0
5
10
15
Blood and lymphatic system disorders
Cardiac disorders
Congenital, familial, and genetic disorders
Ear and labyrinth disorders
Endocrine disorders
Eye disorders
Gastrointestinal disorders
General disorders and administration site conditions
Hepatobiliary disorders
Immune system disorders
Infections and infestations
Injury, poisoning, and procedural complications
Investigations
Metabolism and nutrition disorders
Musculoskeletal and connective tissue disorders
Neoplasms benign, malignant, and unspecified (incl cysts and polyps)
Nervous system disorders
Psychiatric disorders
Renal and urinary disorders
Reproductive system and breast disorders
Respiratory, thoracic, and mediastinal disorders
Skin and subcutaneous tissue disorders
Social circumstances
Surgical and medical procedures
Vascular disorders

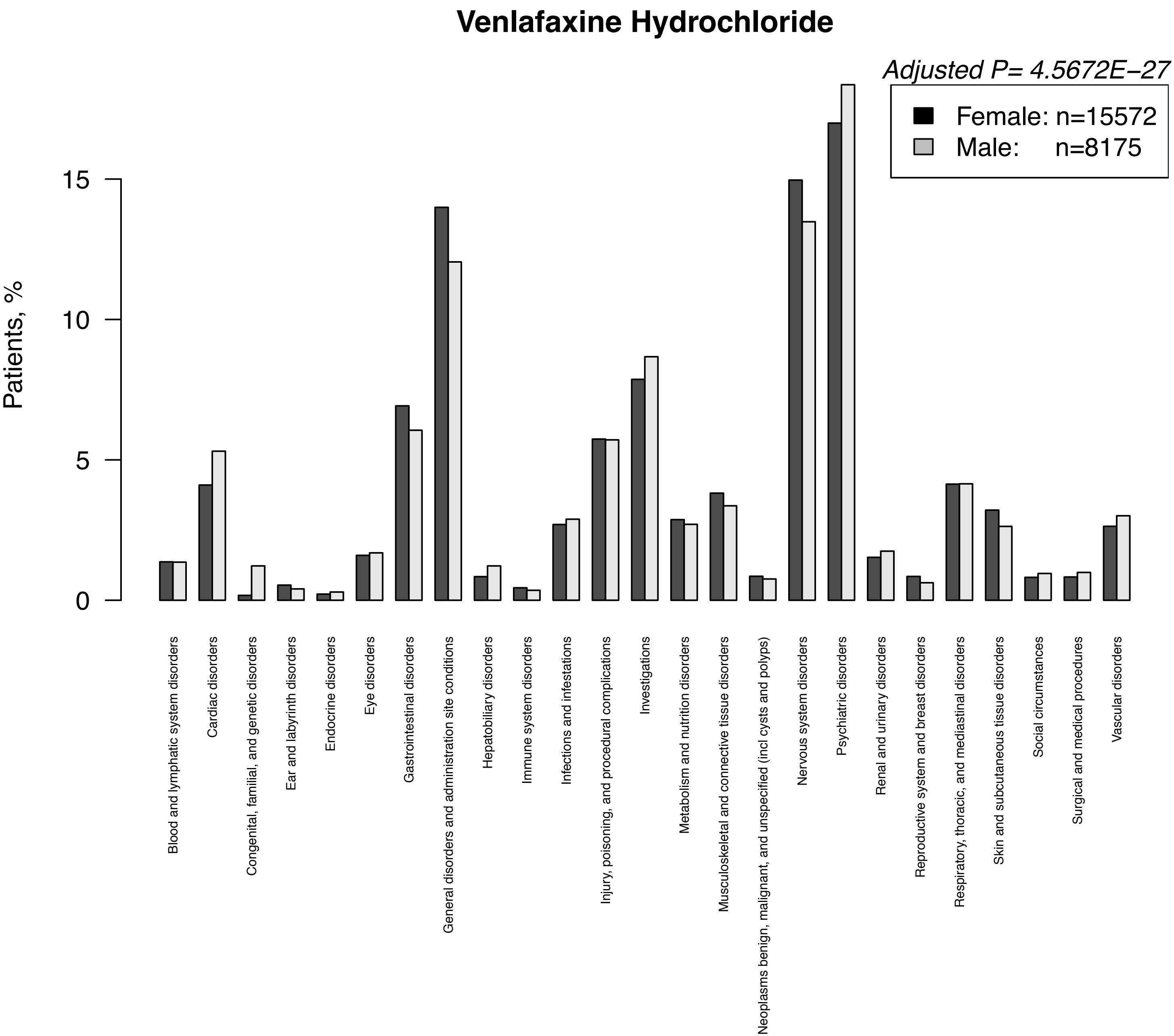
Venlafaxine Hydrochloride
Adjusted P= 4.5672E−27
Female: n=15572
Male: n=8175
Patients, %
15
10
5
0
Blood and lymphatic system disorders
Cardiac disorders
Congenital, familial, and genetic disorders
Ear and labyrinth disorders
Endocrine disorders
Eye disorders
Gastrointestinal disorders
General disorders and administration site conditions
Hepatobiliary disorders
Immune system disorders
Infections and infestations
Injury, poisoning, and procedural complications
Investigations
Metabolism and nutrition disorders
Musculoskeletal and connective tissue disorders
Neoplasms benign, malignant, and unspecified (incl cysts and polyps)
Nervous system disorders
Psychiatric disorders
Renal and urinary disorders
Reproductive system and breast disorders
Respiratory, thoracic, and mediastinal disorders
Skin and subcutaneous tissue disorders
Social circumstances
Surgical and medical procedures
Vascular disorders

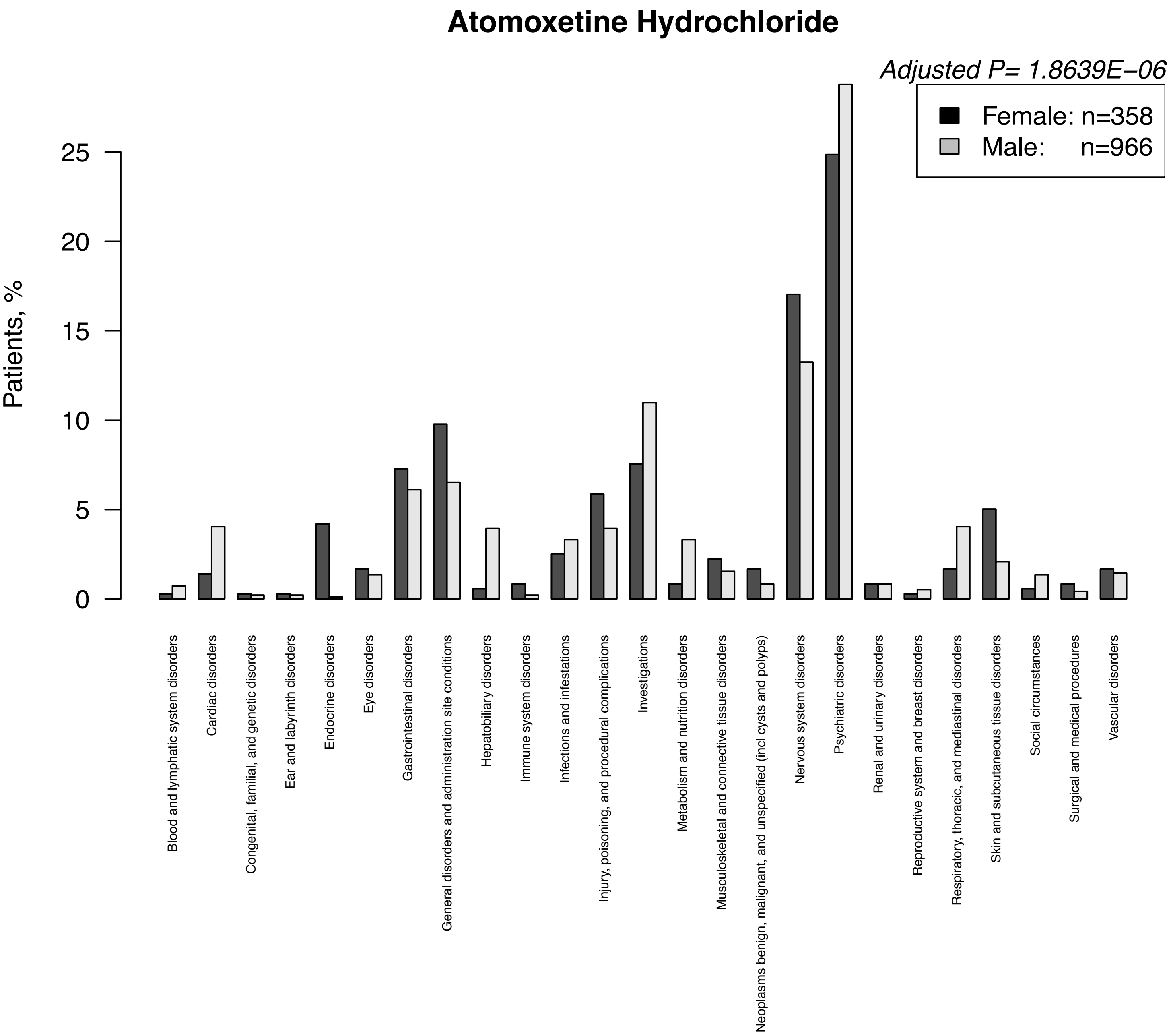
Atomoxetine Hydrochloride
Adjusted P= 1.8639E−06
Female: n=358
Male: n=966
Patients, %
0
5
10
15
20
25
Blood and lymphatic system disorders
Cardiac disorders
Congenital, familial, and genetic disorders
Ear and labyrinth disorders
Endocrine disorders
Eye disorders
Gastrointestinal disorders
General disorders and administration site conditions
Hepatobiliary disorders
Immune system disorders
Infections and infestations
Injury, poisoning, and procedural complications
Investigations
Metabolism and nutrition disorders
Musculoskeletal and connective tissue disorders
Neoplasms benign, malignant, and unspecified (incl cysts and polyps)
Nervous system disorders
Psychiatric disorders
Renal and urinary disorders
Reproductive system and breast disorders
Respiratory, thoracic, and mediastinal disorders
Skin and subcutaneous tissue disorders
Social circumstances
Surgical and medical procedures
Vascular disorders

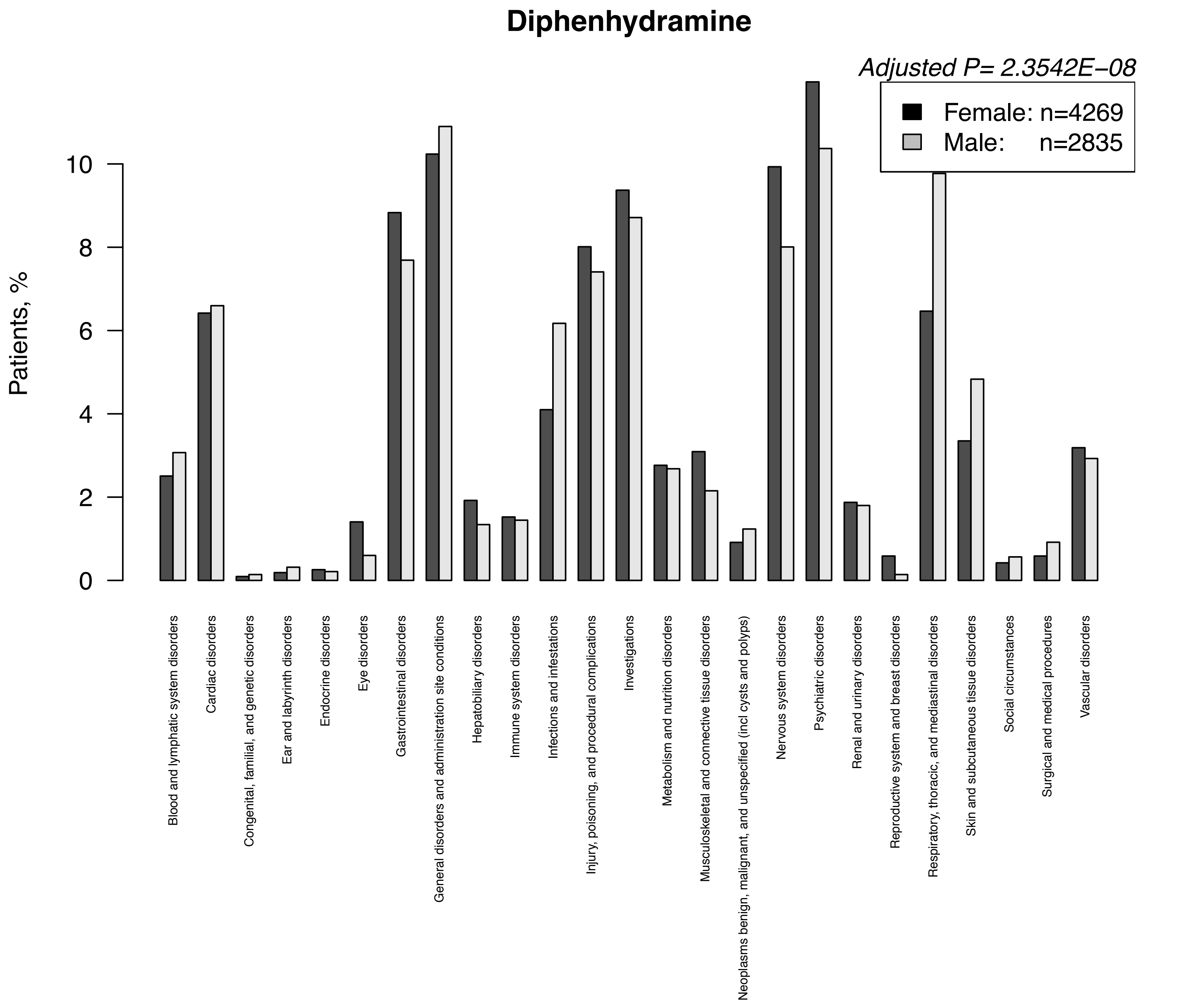
Diphenhydramine
Adjusted P= 2.3542E−08
Female: n=4269
Male: n=2835
Patients, %
0
2
4
6
8
10
Blood and lymphatic system disorders
Cardiac disorders
Congenital, familial, and genetic disorders
Ear and labyrinth disorders
Endocrine disorders
Eye disorders
Gastrointestinal disorders
General disorders and administration site conditions
Hepatobiliary disorders
Immune system disorders
Infections and infestations
Injury, poisoning, and procedural complications
Investigations
Metabolism and nutrition disorders
Musculoskeletal and connective tissue disorders
Neoplasms benign, malignant, and unspecified (incl cysts and polyps)
Nervous system disorders
Psychiatric disorders
Renal and urinary disorders
Reproductive system and breast disorders
Respiratory, thoracic, and mediastinal disorders
Skin and subcutaneous tissue disorders
Social circumstances
Surgical and medical procedures
Vascular disorders

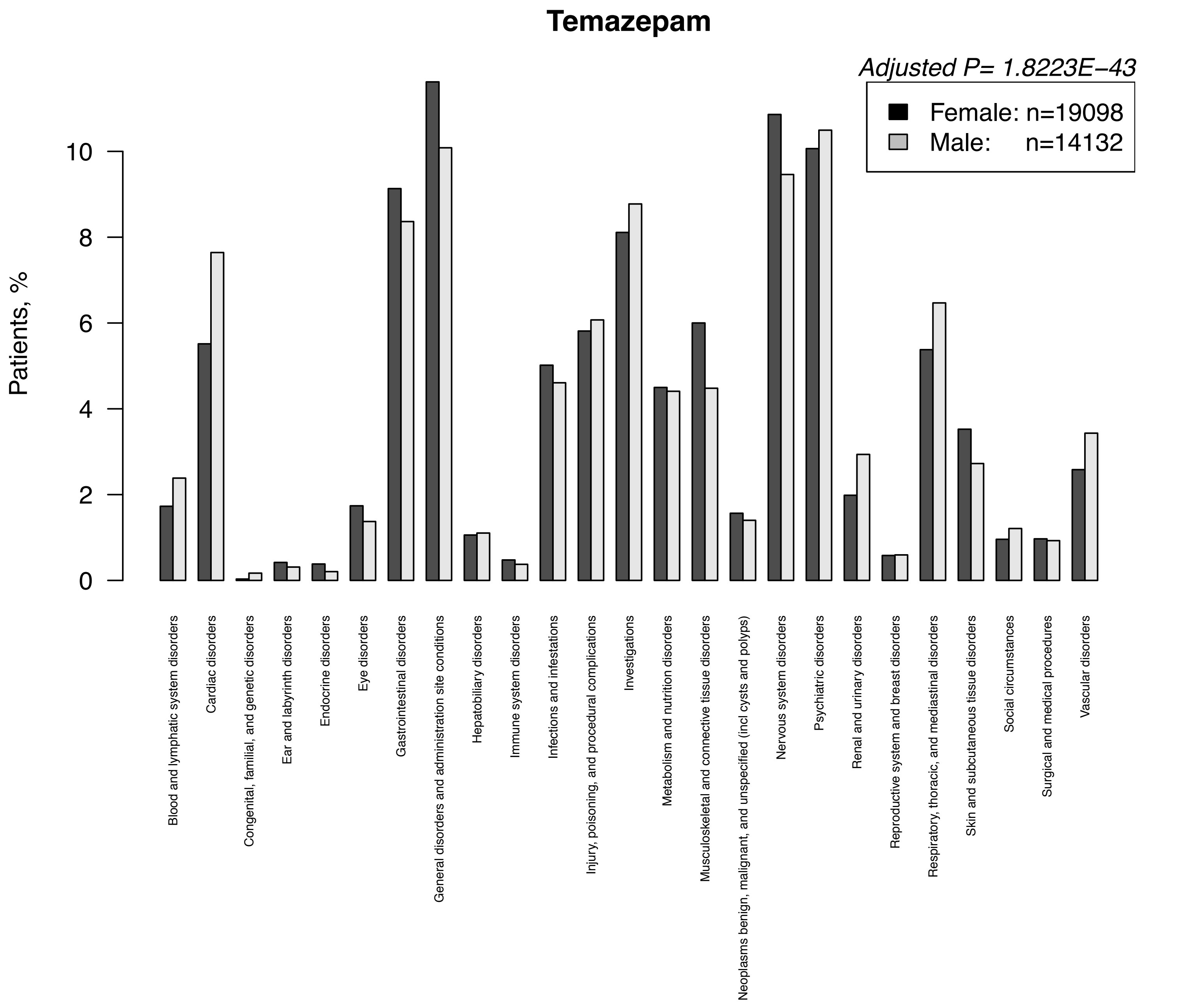
Temazepam
Adjusted P= 1.8223E−43
Female: n=19098
Male: n=14132
Patients, %
0
2
4
6
8
10
Blood and lymphatic system disorders
Cardiac disorders
Congenital, familial, and genetic disorders
Ear and labyrinth disorders
Endocrine disorders
Eye disorders
Gastrointestinal disorders
General disorders and administration site conditions
Hepatobiliary disorders
Immune system disorders
Infections and infestations
Injury, poisoning, and procedural complications
Investigations
Metabolism and nutrition disorders
Musculoskeletal and connective tissue disorders
Neoplasms benign, malignant, and unspecified (incl cysts and polyps)
Nervous system disorders
Psychiatric disorders
Renal and urinary disorders
Reproductive system and breast disorders
Respiratory, thoracic, and mediastinal disorders
Skin and subcutaneous tissue disorders
Social circumstances
Surgical and medical procedures
Vascular disorders

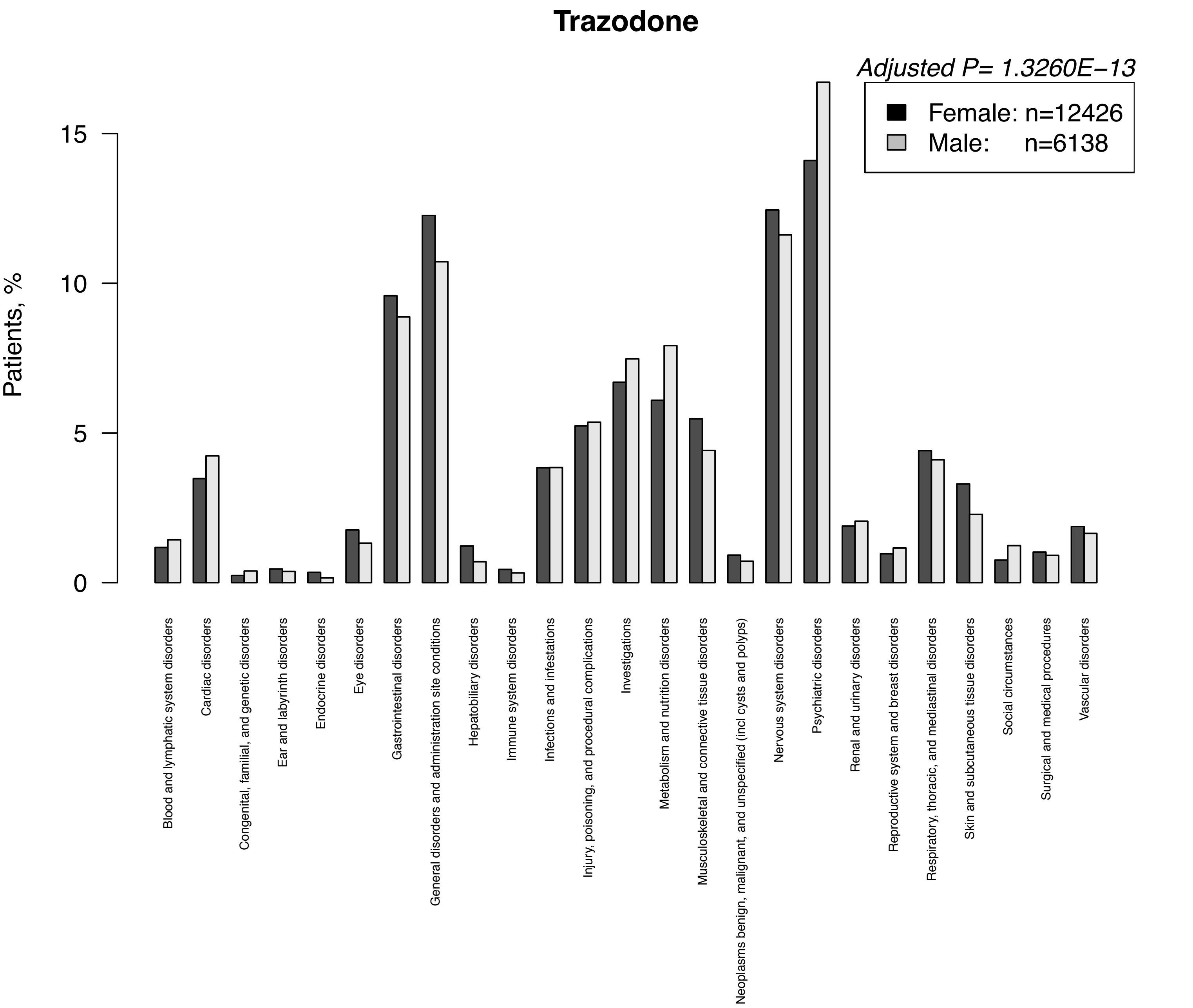
Trazodone
Adjusted P= 1.3260E−13
Female: n=12426
Male: n=6138
Patients, %
0
5
10
15
Blood and lymphatic system disorders
Cardiac disorders
Congenital, familial, and genetic disorders
Ear and labyrinth disorders
Endocrine disorders
Eye disorders
Gastrointestinal disorders
General disorders and administration site conditions
Hepatobiliary disorders
Immune system disorders
Infections and infestations
Injury, poisoning, and procedural complications
Investigations
Metabolism and nutrition disorders
Musculoskeletal and connective tissue disorders
Neoplasms benign, malignant, and unspecified (incl cysts and polyps)
Nervous system disorders
Psychiatric disorders
Renal and urinary disorders
Reproductive system and breast disorders
Respiratory, thoracic, and mediastinal disorders
Skin and subcutaneous tissue disorders
Social circumstances
Surgical and medical procedures
Vascular disorders

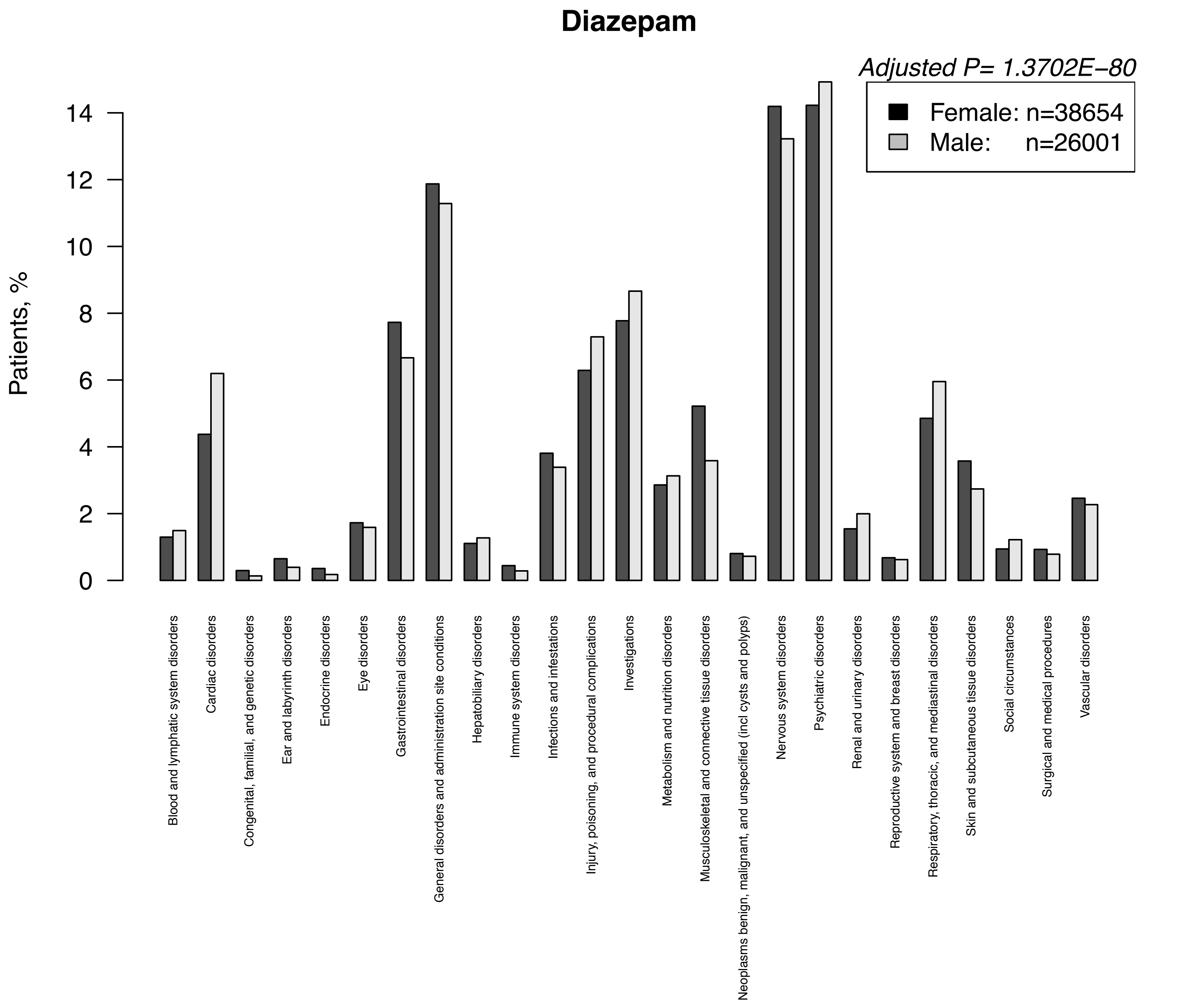

Diazepam
Adjusted P= 1.3702E−80
Female: n=38654
Male: n=26001
Patients, %
0
2
4
6
8
10
12
14
Blood and lymphatic system disorders
Cardiac disorders
Congenital, familial, and genetic disorders
Ear and labyrinth disorders
Endocrine disorders
Eye disorders
Gastrointestinal disorders
General disorders and administration site conditions
Hepatobiliary disorders
Immune system disorders
Infections and infestations
Injury, poisoning, and procedural complications
Investigations
Metabolism and nutrition disorders
Musculoskeletal and connective tissue disorders
Neoplasms benign, malignant, and unspecified (incl cysts and polyps)
Nervous system disorders
Psychiatric disorders
Renal and urinary disorders
Reproductive system and breast disorders
Respiratory, thoracic, and mediastinal disorders
Skin and subcutaneous tissue disorders
Social circumstances
Surgical and medical procedures
Vascular disorders

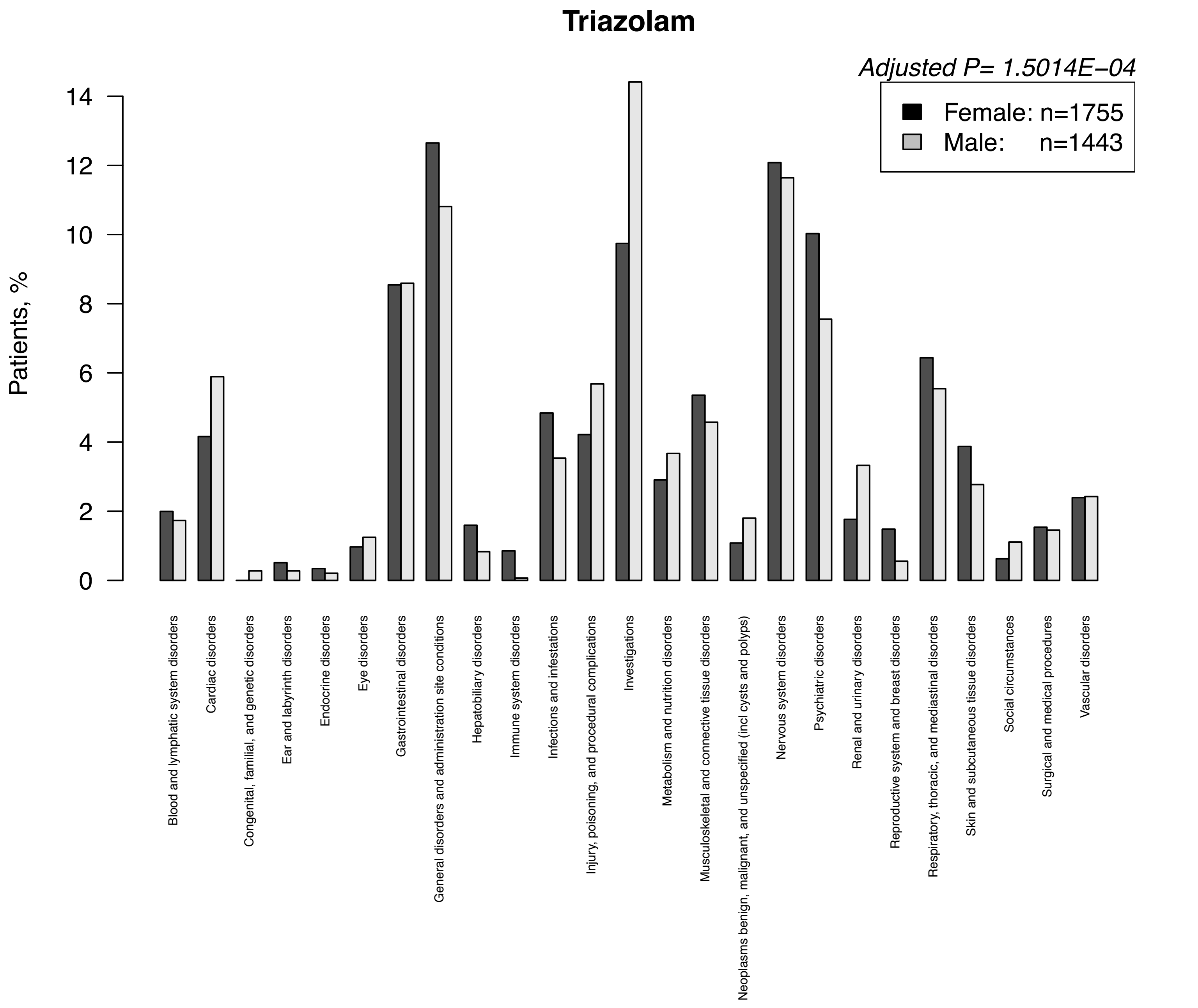

Triazolam
Adjusted P= 1.5014E−04
Female: n=1755
Male: n=1443
Patients, %
0
2
4
6
8
10
12
14
Blood and lymphatic system disorders
Cardiac disorders
Congenital, familial, and genetic disorders
Ear and labyrinth disorders
Endocrine disorders
Eye disorders
Gastrointestinal disorders
General disorders and administration site conditions
Hepatobiliary disorders
Immune system disorders
Infections and infestations
Injury, poisoning, and procedural complications
Investigations
Metabolism and nutrition disorders
Musculoskeletal and connective tissue disorders
Neoplasms benign, malignant, and unspecified (incl cysts and polyps)
Nervous system disorders
Psychiatric disorders
Renal and urinary disorders
Reproductive system and breast disorders
Respiratory, thoracic, and mediastinal disorders
Skin and subcutaneous tissue disorders
Social circumstances
Surgical and medical procedures
Vascular disorders

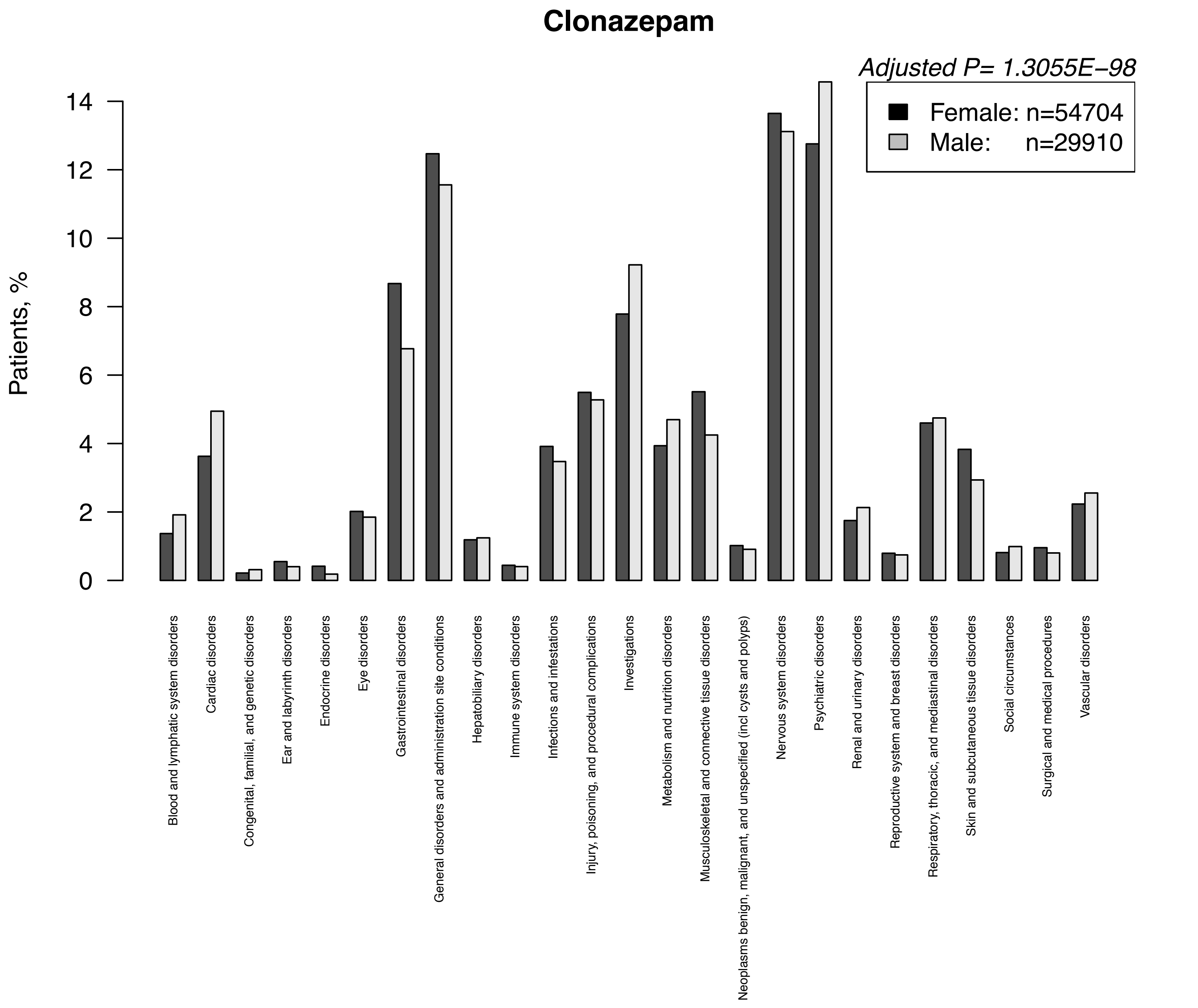
Clonazepam
Adjusted P= 1.3055E−98
Female: n=54704
Male: n=29910
Patients, %
0
2
4
6
8
10
12
14
Blood and lymphatic system disorders
Cardiac disorders
Congenital, familial, and genetic disorders
Ear and labyrinth disorders
Endocrine disorders
Eye disorders
Gastrointestinal disorders
General disorders and administration site conditions
Hepatobiliary disorders
Immune system disorders
Infections and infestations
Injury, poisoning, and procedural complications
Investigations
Metabolism and nutrition disorders
Musculoskeletal and connective tissue disorders
Neoplasms benign, malignant, and unspecified (incl cysts and polyps)
Nervous system disorders
Psychiatric disorders
Renal and urinary disorders
Reproductive system and breast disorders
Respiratory, thoracic, and mediastinal disorders
Skin and subcutaneous tissue disorders
Social circumstances
Surgical and medical procedures
Vascular disorders

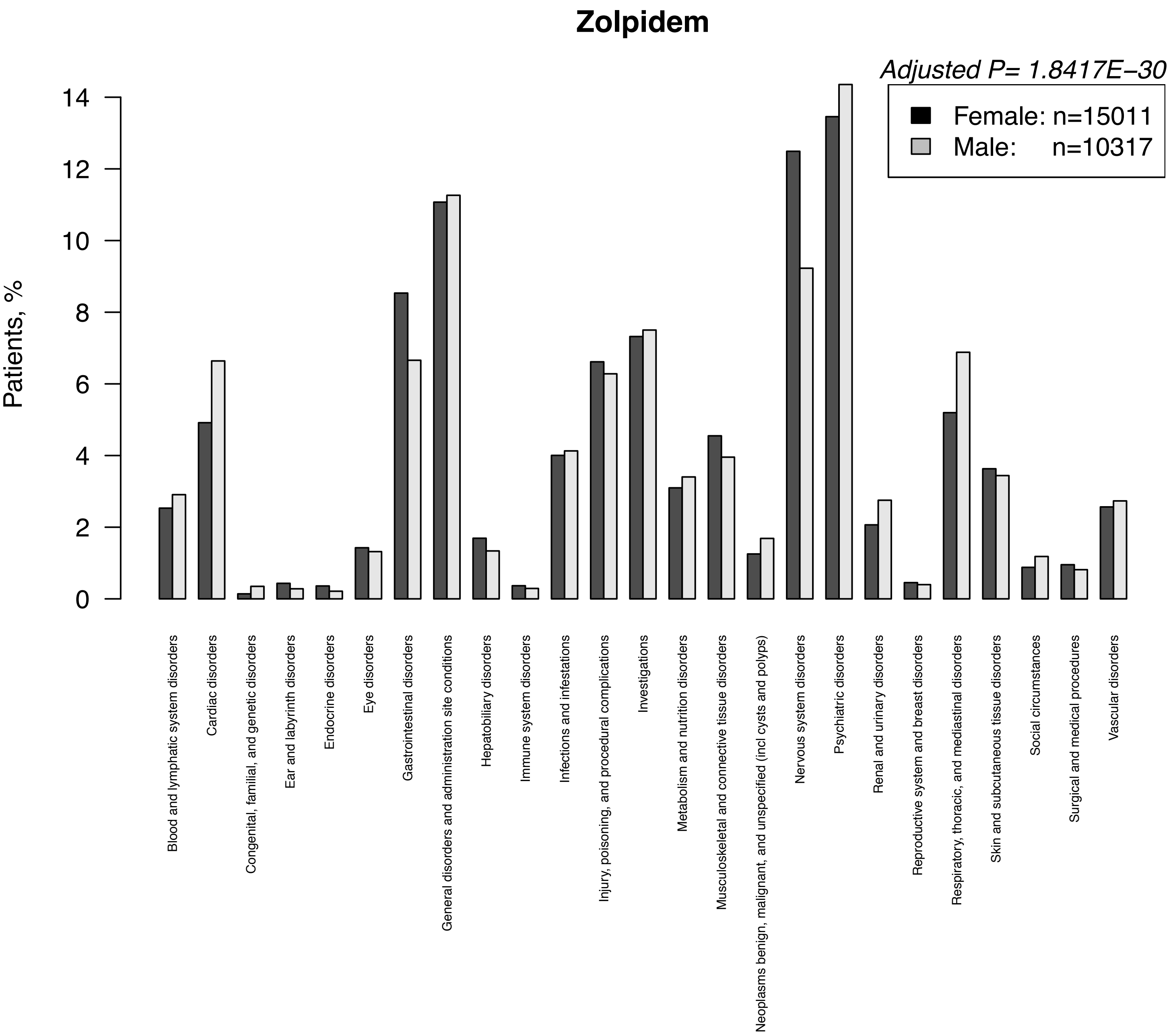

Zolpidem
Adjusted P= 1.8417E−30
Female: n=15011
Male: n=10317
Patients, %
0
2
4
6
8
10
12
14
Blood and lymphatic system disorders
Cardiac disorders
Congenital, familial, and genetic disorders
Ear and labyrinth disorders
Endocrine disorders
Eye disorders
Gastrointestinal disorders
General disorders and administration site conditions
Hepatobiliary disorders
Immune system disorders
Infections and infestations
Injury, poisoning, and procedural complications
Investigations
Metabolism and nutrition disorders
Musculoskeletal and connective tissue disorders
Neoplasms benign, malignant, and unspecified (incl cysts and polyps)
Nervous system disorders
Psychiatric disorders
Renal and urinary disorders
Reproductive system and breast disorders
Respiratory, thoracic, and mediastinal disorders
Skin and subcutaneous tissue disorders
Social circumstances
Surgical and medical procedures
Vascular disorders

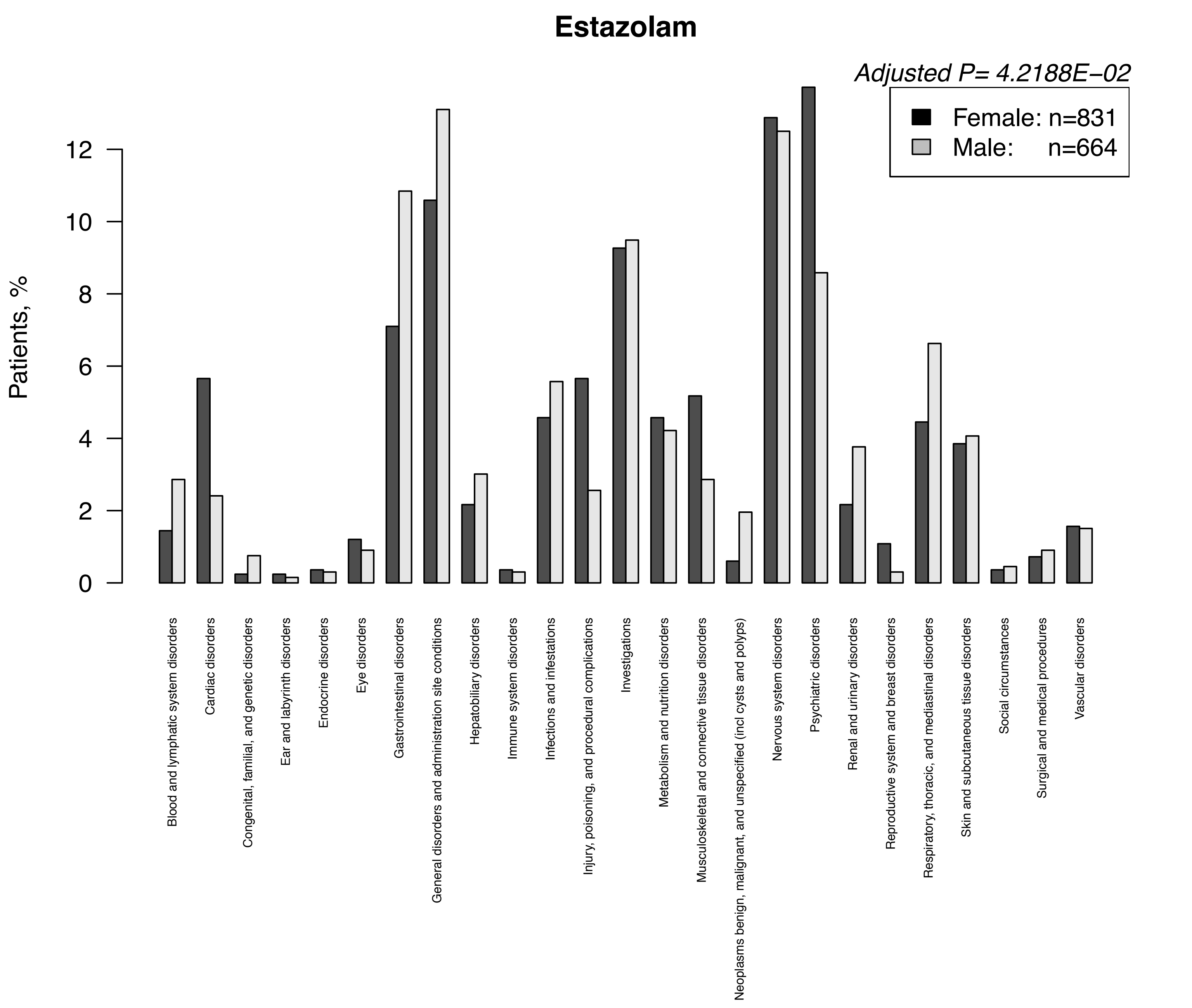
Estazolam
Adjusted P= 4.2188E−02
Female: n=831
Male: n=664
Patients, %
0
2
4
6
8
10
12
Blood and lymphatic system disorders
Cardiac disorders
Congenital, familial, and genetic disorders
Ear and labyrinth disorders
Endocrine disorders
Eye disorders
Gastrointestinal disorders
General disorders and administration site conditions
Hepatobiliary disorders
Immune system disorders
Infections and infestations
Injury, poisoning, and procedural complications
Investigations
Metabolism and nutrition disorders
Musculoskeletal and connective tissue disorders
Neoplasms benign, malignant, and unspecified (incl cysts and polyps)
Nervous system disorders
Psychiatric disorders
Renal and urinary disorders
Reproductive system and breast disorders
Respiratory, thoracic, and mediastinal disorders
Skin and subcutaneous tissue disorders
Social circumstances
Surgical and medical procedures
Vascular disorders

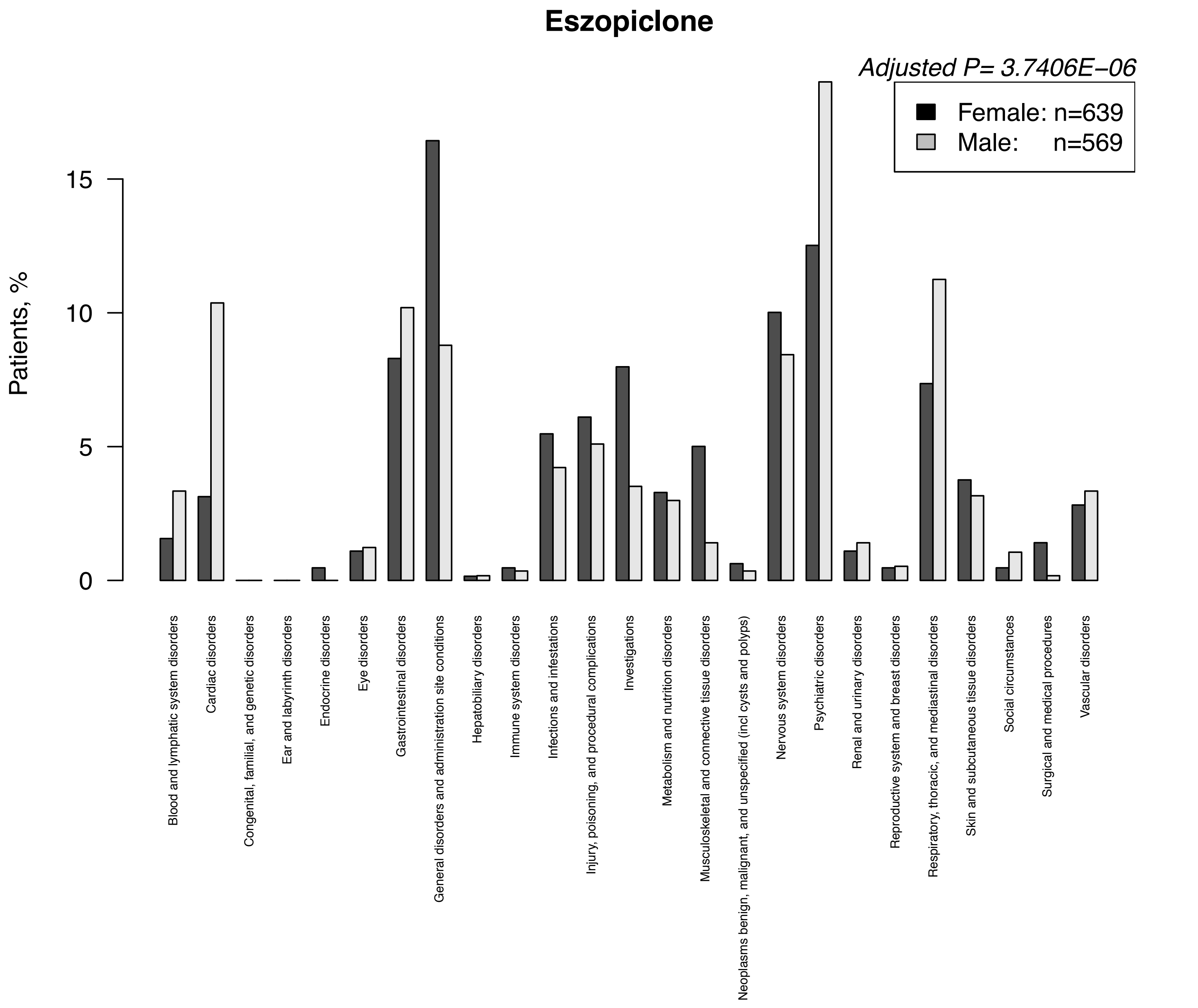
Eszopiclone
Adjusted P= 3.7406E−06
Female: n=639
Male: n=569
Patients, %
0
5
10
15
Blood and lymphatic system disorders
Cardiac disorders
Congenital, familial, and genetic disorders
Ear and labyrinth disorders
Endocrine disorders
Eye disorders
Gastrointestinal disorders
General disorders and administration site conditions
Hepatobiliary disorders
Immune system disorders
Infections and infestations
Injury, poisoning, and procedural complications
Investigations
Metabolism and nutrition disorders
Musculoskeletal and connective tissue disorders
Neoplasms benign, malignant, and unspecified (incl cysts and polyps)
Nervous system disorders
Psychiatric disorders
Renal and urinary disorders
Reproductive system and breast disorders
Respiratory, thoracic, and mediastinal disorders
Skin and subcutaneous tissue disorders
Social circumstances
Surgical and medical procedures
Vascular disorders

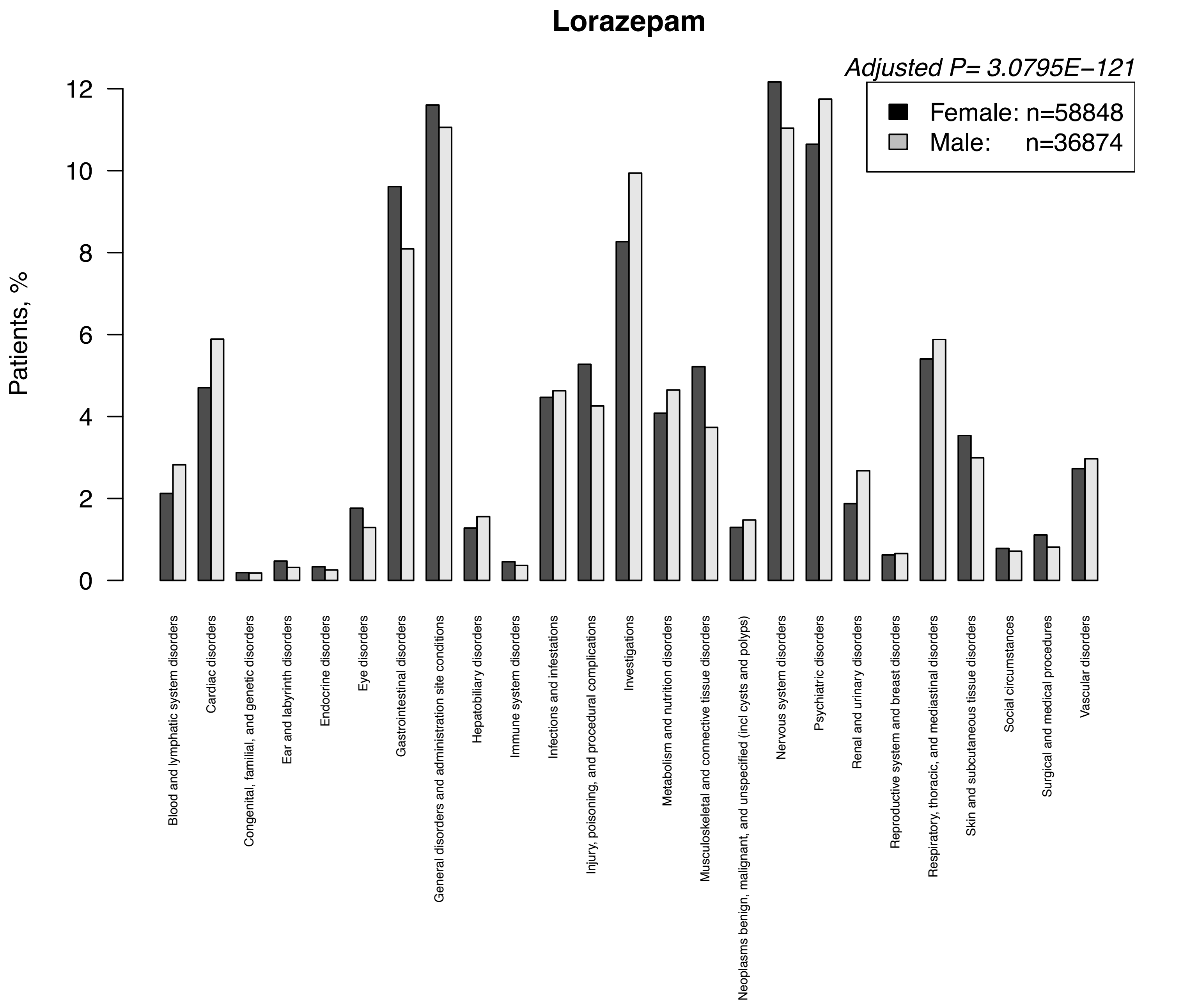

Lorazepam
Adjusted P= 3.0795E−121
Female: n=58848
Male: n=36874
Patients, %
0
2
4
6
8
10
12
Blood and lymphatic system disorders
Cardiac disorders
Congenital, familial, and genetic disorders
Ear and labyrinth disorders
Endocrine disorders
Eye disorders
Gastrointestinal disorders
General disorders and administration site conditions
Hepatobiliary disorders
Immune system disorders
Infections and infestations
Injury, poisoning, and procedural complications
Investigations
Metabolism and nutrition disorders
Musculoskeletal and connective tissue disorders
Neoplasms benign, malignant, and unspecified (incl cysts and polyps)
Nervous system disorders
Psychiatric disorders
Renal and urinary disorders
Reproductive system and breast disorders
Respiratory, thoracic, and mediastinal disorders
Skin and subcutaneous tissue disorders
Social circumstances
Surgical and medical procedures
Vascular disorders

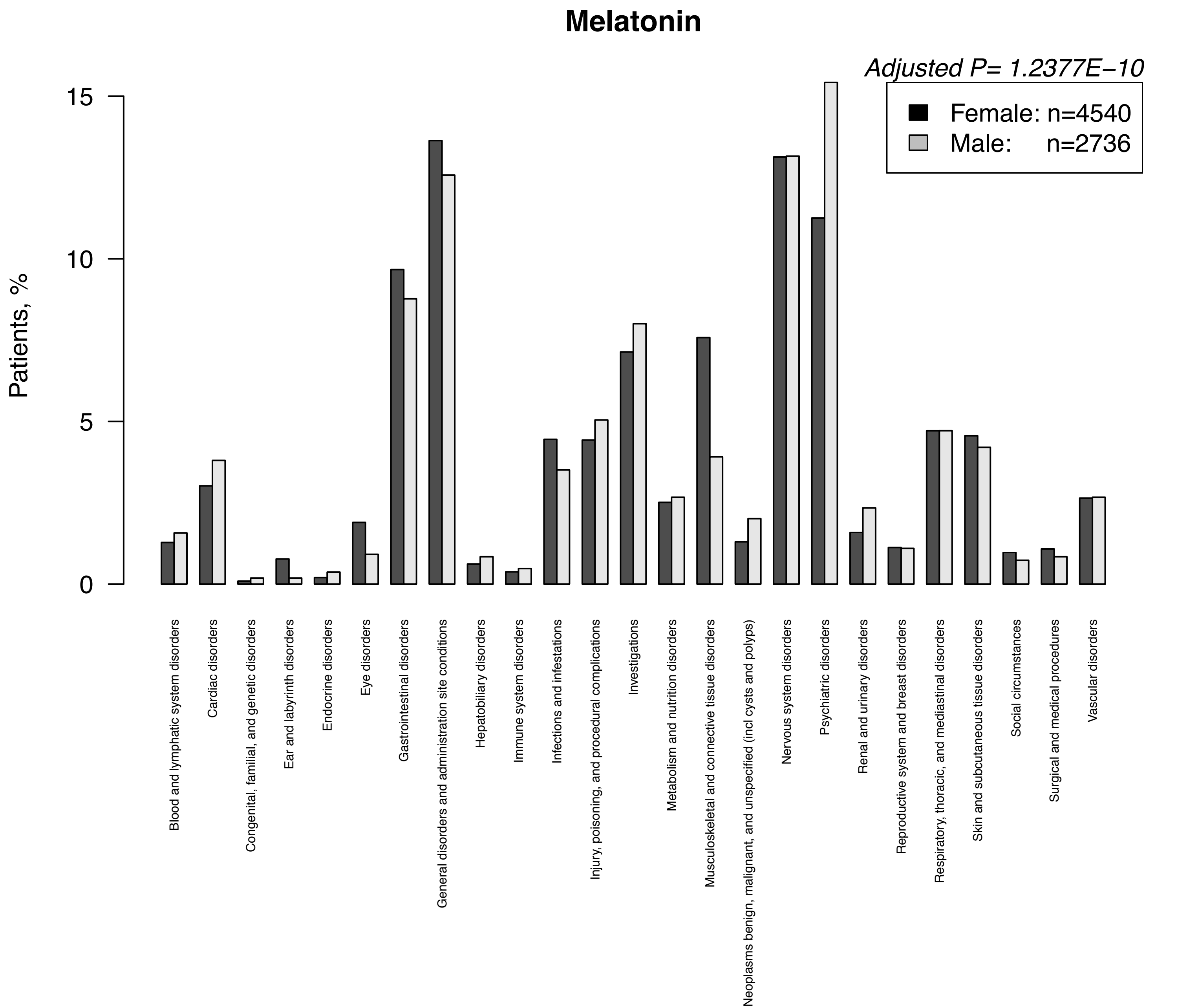
Melatonin
Adjusted P= 1.2377E−10
Female: n=4540
Male: n=2736
Patients, %
0
5
10
15
Blood and lymphatic system disorders
Cardiac disorders
Congenital, familial, and genetic disorders
Ear and labyrinth disorders
Endocrine disorders
Eye disorders
Gastrointestinal disorders
General disorders and administration site conditions
Hepatobiliary disorders
Immune system disorders
Infections and infestations
Injury, poisoning, and procedural complications
Investigations
Metabolism and nutrition disorders
Musculoskeletal and connective tissue disorders
Neoplasms benign, malignant, and unspecified (incl cysts and polyps)
Nervous system disorders
Psychiatric disorders
Renal and urinary disorders
Reproductive system and breast disorders
Respiratory, thoracic, and mediastinal disorders
Skin and subcutaneous tissue disorders
Social circumstances
Surgical and medical procedures
Vascular disorders

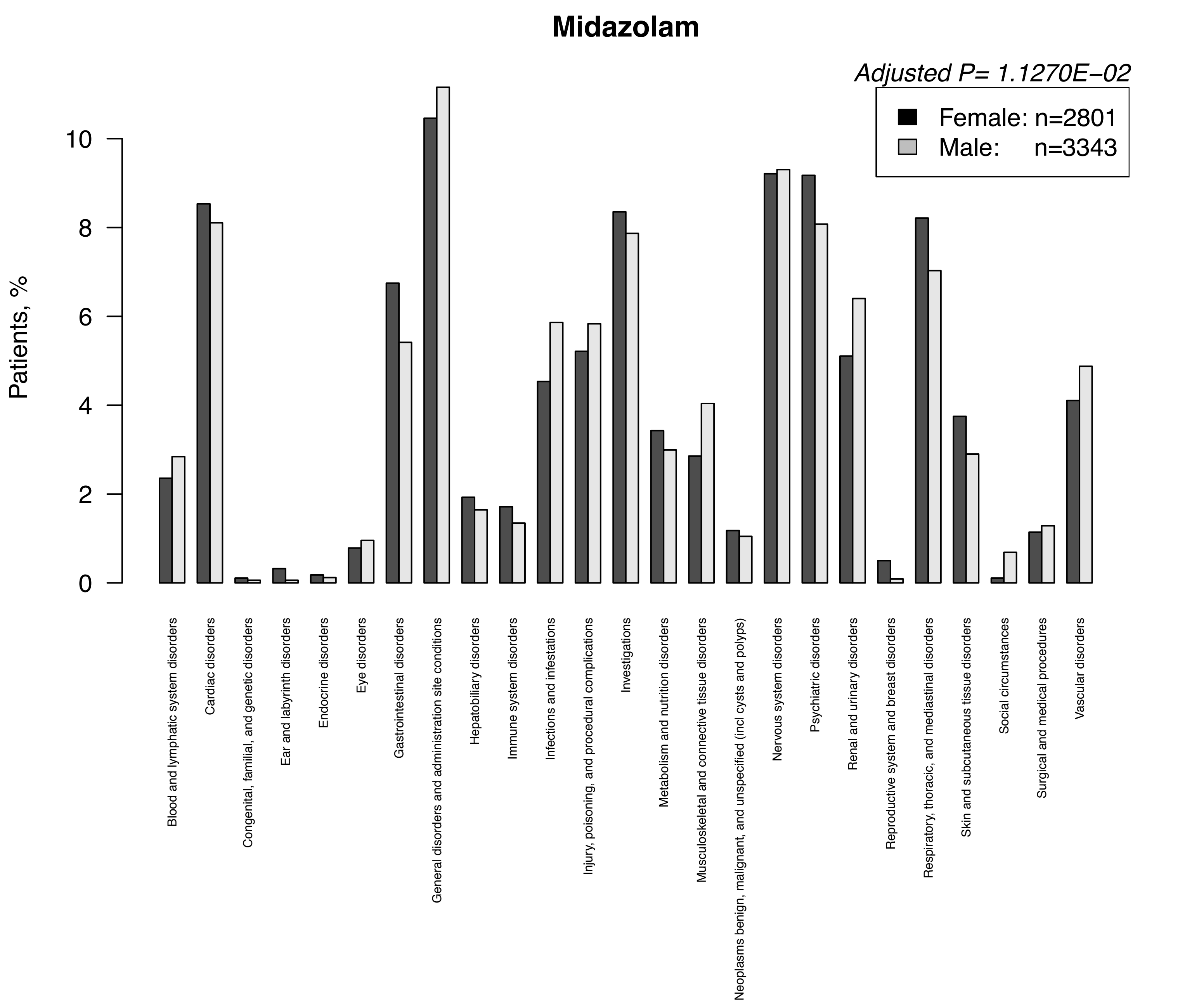
Midazolam
Adjusted P= 1.1270E−02
Female: n=2801
Male: n=3343
Patients, %
0
2
4
6
8
10
Blood and lymphatic system disorders
Cardiac disorders
Congenital, familial, and genetic disorders
Ear and labyrinth disorders
Endocrine disorders
Eye disorders
Gastrointestinal disorders
General disorders and administration site conditions
Hepatobiliary disorders
Immune system disorders
Infections and infestations
Injury, poisoning, and procedural complications
Investigations
Metabolism and nutrition disorders
Musculoskeletal and connective tissue disorders
Neoplasms benign, malignant, and unspecified (incl cysts and polyps)
Nervous system disorders
Psychiatric disorders
Renal and urinary disorders
Reproductive system and breast disorders
Respiratory, thoracic, and mediastinal disorders
Skin and subcutaneous tissue disorders
Social circumstances
Surgical and medical procedures
Vascular disorders

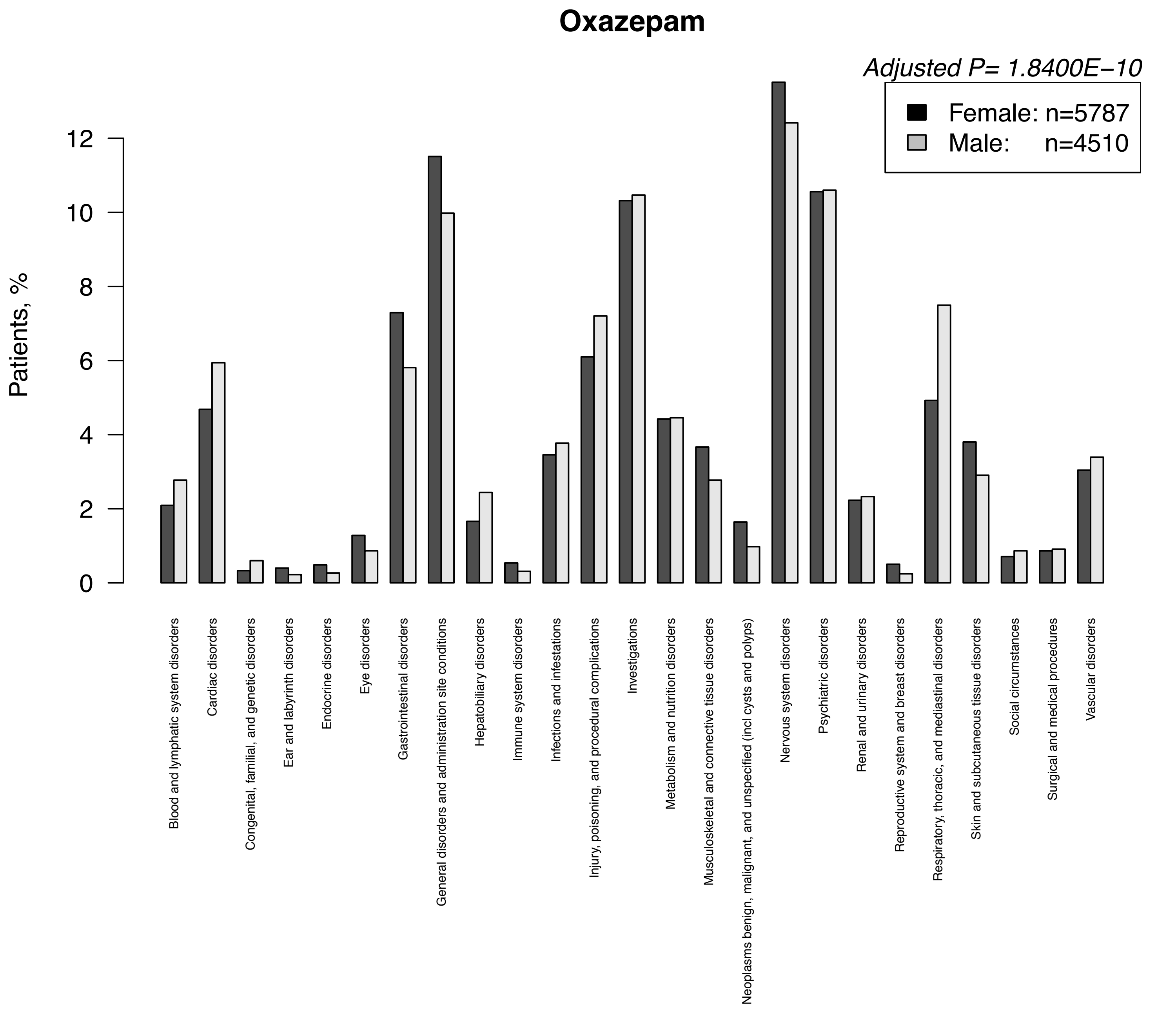

Oxazepam
Adjusted P= 1.8400E−10
Female: n=5787
Male: n=4510
Patients, %
0
2
4
6
8
10
12
Blood and lymphatic system disorders
Cardiac disorders
Congenital, familial, and genetic disorders
Ear and labyrinth disorders
Endocrine disorders
Eye disorders
Gastrointestinal disorders
General disorders and administration site conditions
Hepatobiliary disorders
Immune system disorders
Infections and infestations
Injury, poisoning, and procedural complications
Investigations
Metabolism and nutrition disorders
Musculoskeletal and connective tissue disorders
Neoplasms benign, malignant, and unspecified (incl cysts and polyps)
Nervous system disorders
Psychiatric disorders
Renal and urinary disorders
Reproductive system and breast disorders
Respiratory, thoracic, and mediastinal disorders
Skin and subcutaneous tissue disorders
Social circumstances
Surgical and medical procedures
Vascular disorders

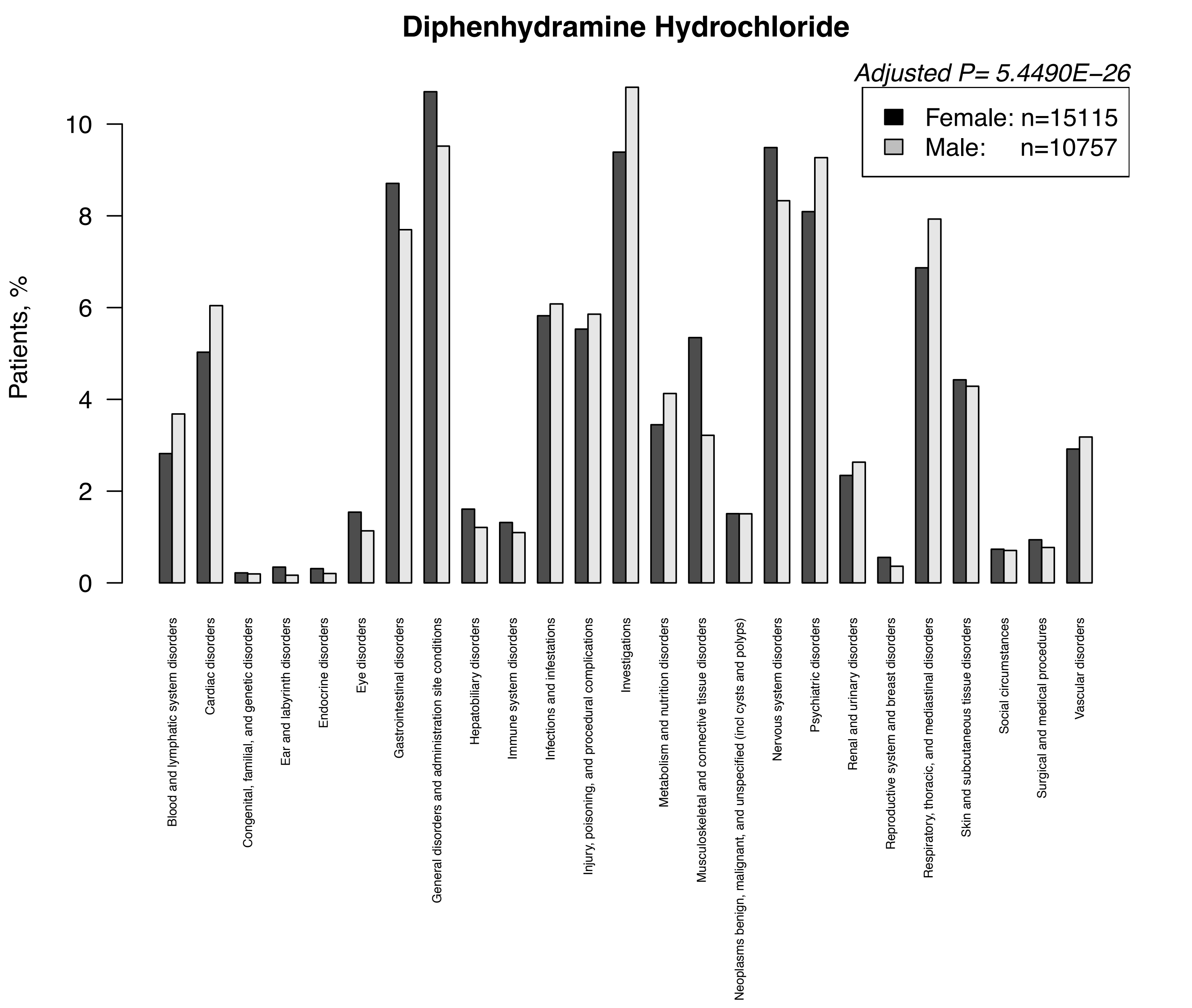

Diphenhydramine Hydrochloride
Adjusted P= 5.4490E−26
Female: n=15115
Male: n=10757
Patients, %
0
2
4
6
8
10
Blood and lymphatic system disorders
Cardiac disorders
Congenital, familial, and genetic disorders
Ear and labyrinth disorders
Endocrine disorders
Eye disorders
Gastrointestinal disorders
General disorders and administration site conditions
Hepatobiliary disorders
Immune system disorders
Infections and infestations
Injury, poisoning, and procedural complications
Investigations
Metabolism and nutrition disorders
Musculoskeletal and connective tissue disorders
Neoplasms benign, malignant, and unspecified (incl cysts and polyps)
Nervous system disorders
Psychiatric disorders
Renal and urinary disorders
Reproductive system and breast disorders
Respiratory, thoracic, and mediastinal disorders
Skin and subcutaneous tissue disorders
Social circumstances
Surgical and medical procedures
Vascular disorders

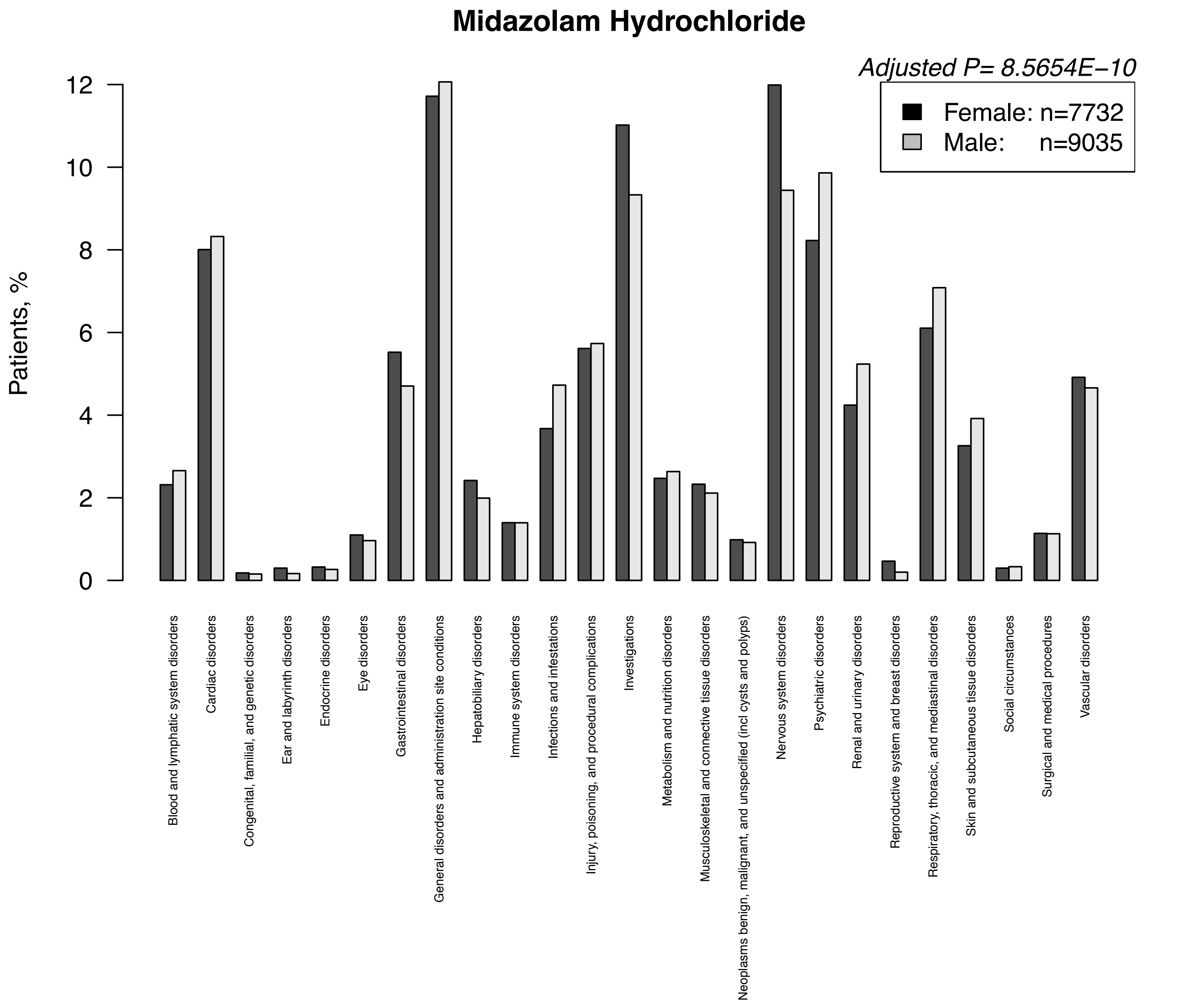

Midazolam Hydrochloride
Adjusted P= 8.5654E−10
Female: n=7732
Male: n=9035
Patients, %
0
2
4
6
8
10
12
Blood and lymphatic system disorders
Cardiac disorders
Congenital, familial, and genetic disorders
Ear and labyrinth disorders
Endocrine disorders
Eye disorders
Gastrointestinal disorders
General disorders and administration site conditions
Hepatobiliary disorders
Immune system disorders
Infections and infestations
Injury, poisoning, and procedural complications
Investigations
Metabolism and nutrition disorders
Musculoskeletal and connective tissue disorders
Neoplasms benign, malignant, and unspecified (incl cysts and polyps)
Nervous system disorders
Psychiatric disorders
Renal and urinary disorders
Reproductive system and breast disorders
Respiratory, thoracic, and mediastinal disorders
Skin and subcutaneous tissue disorders
Social circumstances
Surgical and medical procedures
Vascular disorders

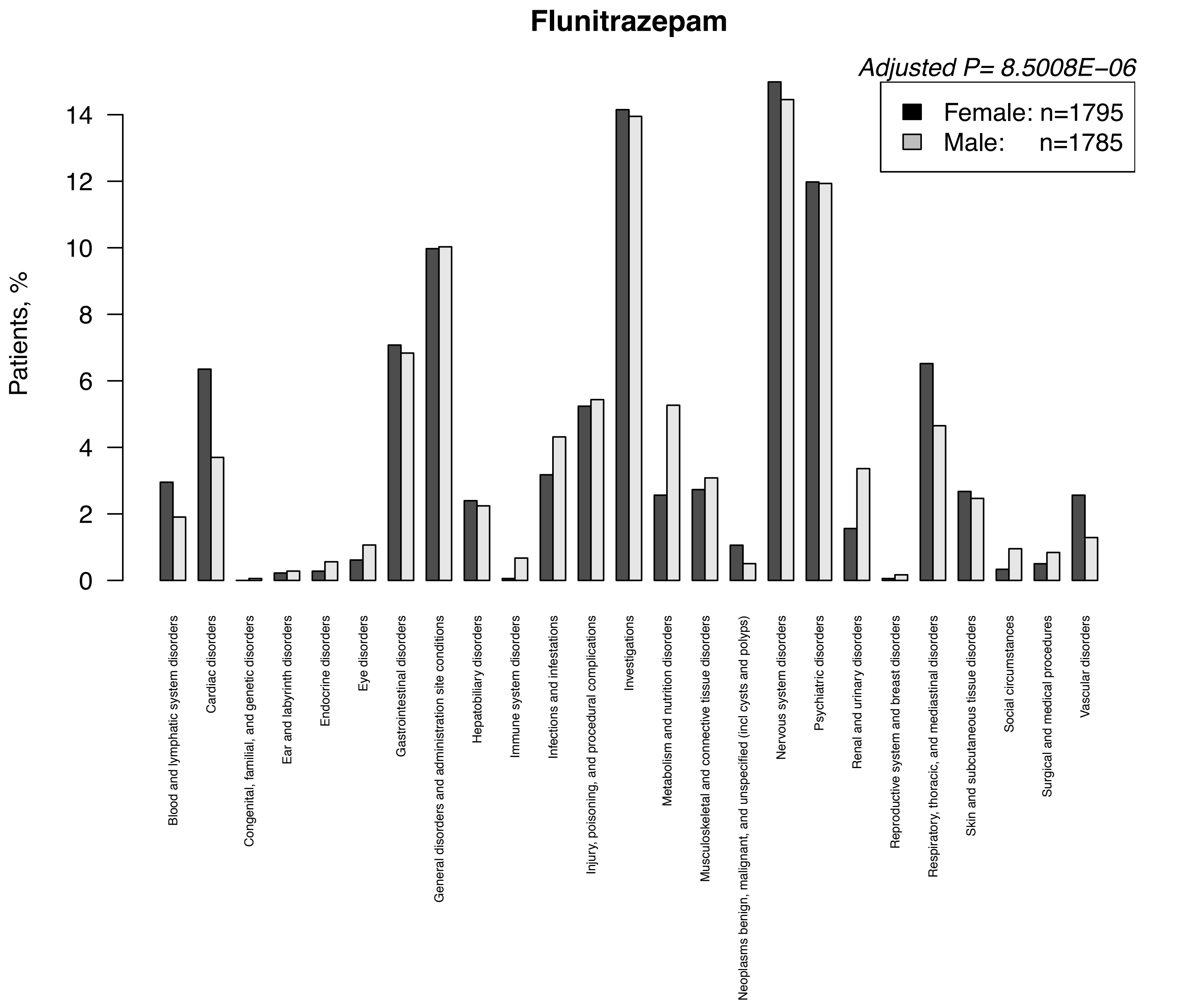
Flunitrazepam
Adjusted P= 8.5008E−06
Female: n=1795
Male: n=1785
Patients, %
0
2
4
6
8
10
12
14
Blood and lymphatic system disorders
Cardiac disorders
Congenital, familial, and genetic disorders
Ear and labyrinth disorders
Endocrine disorders
Eye disorders
Gastrointestinal disorders
General disorders and administration site conditions
Hepatobiliary disorders
Immune system disorders
Infections and infestations
Injury, poisoning, and procedural complications
Investigations
Metabolism and nutrition disorders
Musculoskeletal and connective tissue disorders
Neoplasms benign, malignant, and unspecified (incl cysts and polyps)
Nervous system disorders
Psychiatric disorders
Renal and urinary disorders
Reproductive system and breast disorders
Respiratory, thoracic, and mediastinal disorders
Skin and subcutaneous tissue disorders
Social circumstances
Surgical and medical procedures
Vascular disorders

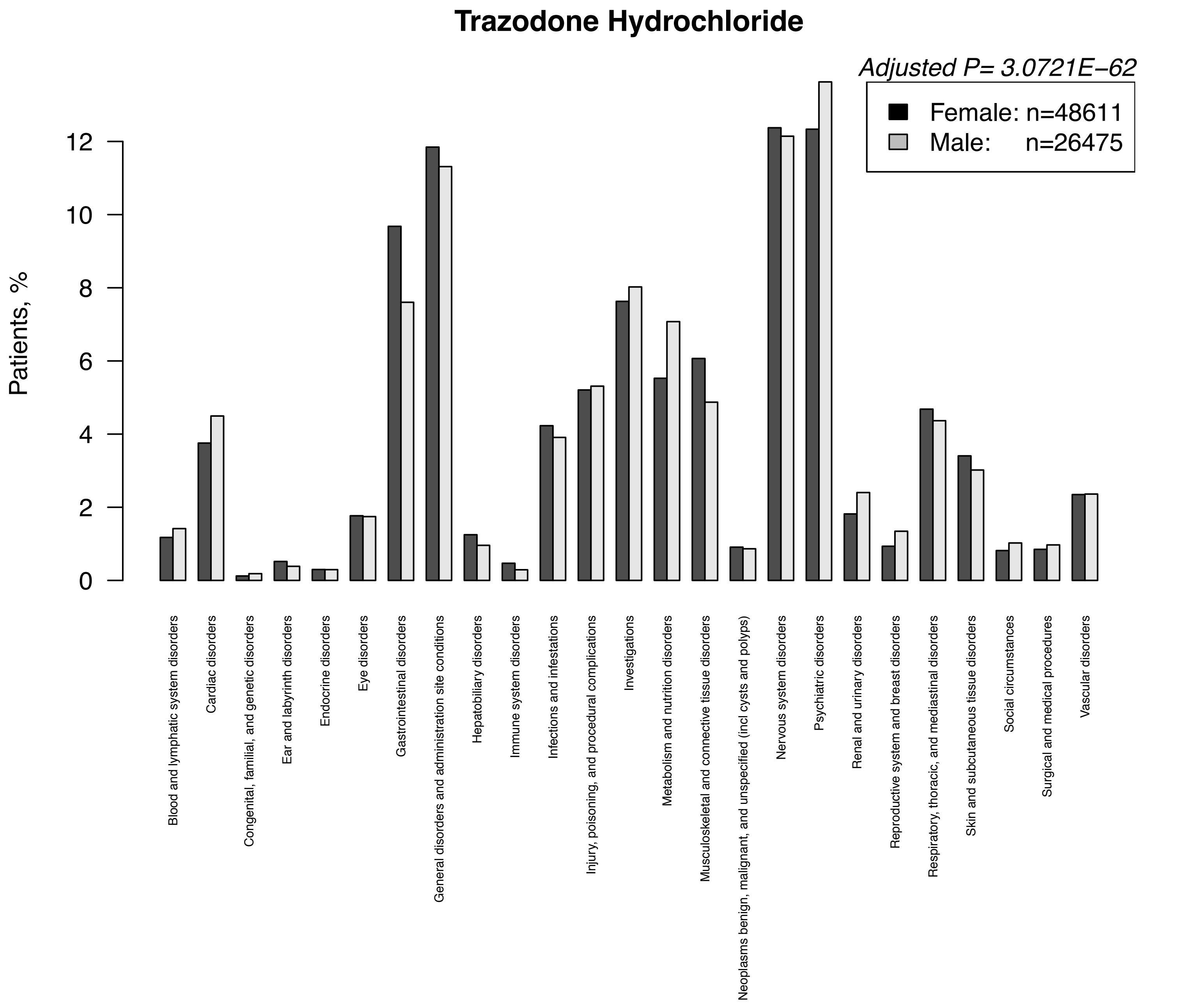
Trazodone Hydrochloride
Adjusted P= 3.0721E−62
Female: n=48611
Male: n=26475
Patients, %
0
2
4
6
8
10
12
Blood and lymphatic system disorders
Cardiac disorders
Congenital, familial, and genetic disorders
Ear and labyrinth disorders
Endocrine disorders
Eye disorders
Gastrointestinal disorders
General disorders and administration site conditions
Hepatobiliary disorders
Immune system disorders
Infections and infestations
Injury, poisoning, and procedural complications
Investigations
Metabolism and nutrition disorders
Musculoskeletal and connective tissue disorders
Neoplasms benign, malignant, and unspecified (incl cysts and polyps)
Nervous system disorders
Psychiatric disorders
Renal and urinary disorders
Reproductive system and breast disorders
Respiratory, thoracic, and mediastinal disorders
Skin and subcutaneous tissue disorders
Social circumstances
Surgical and medical procedures
Vascular disorders

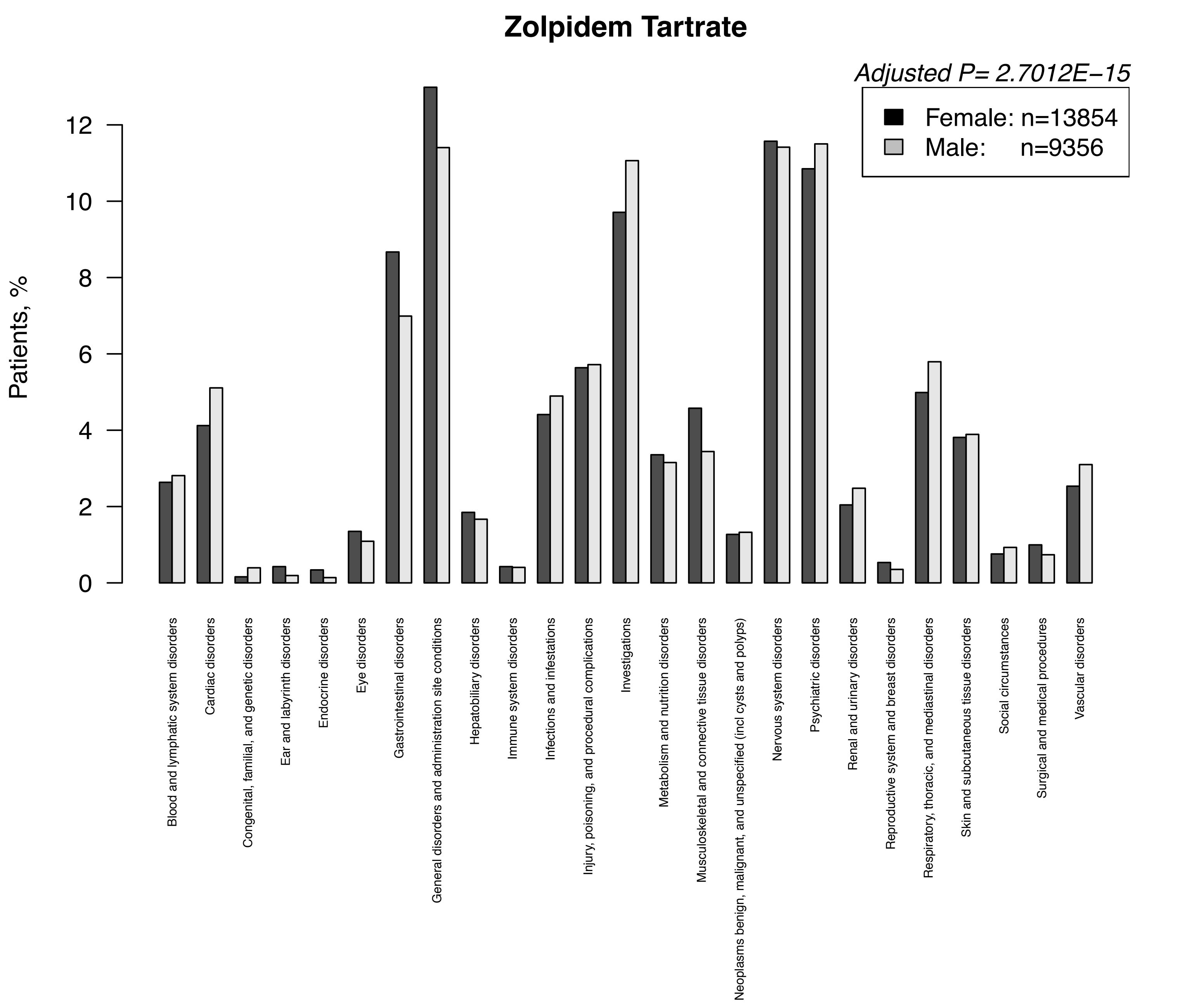

Zolpidem Tartrate
Adjusted P= 2.7012E−15
Female: n=13854
Male: n=9356
Patients, %
0
2
4
6
8
10
12
Blood and lymphatic system disorders
Cardiac disorders
Congenital, familial, and genetic disorders
Ear and labyrinth disorders
Endocrine disorders
Eye disorders
Gastrointestinal disorders
General disorders and administration site conditions
Hepatobiliary disorders
Immune system disorders
Infections and infestations
Injury, poisoning, and procedural complications
Investigations
Metabolism and nutrition disorders
Musculoskeletal and connective tissue disorders
Neoplasms benign, malignant, and unspecified (incl cysts and polyps)
Nervous system disorders
Psychiatric disorders
Renal and urinary disorders
Reproductive system and breast disorders
Respiratory, thoracic, and mediastinal disorders
Skin and subcutaneous tissue disorders
Social circumstances
Surgical and medical procedures
Vascular disorders

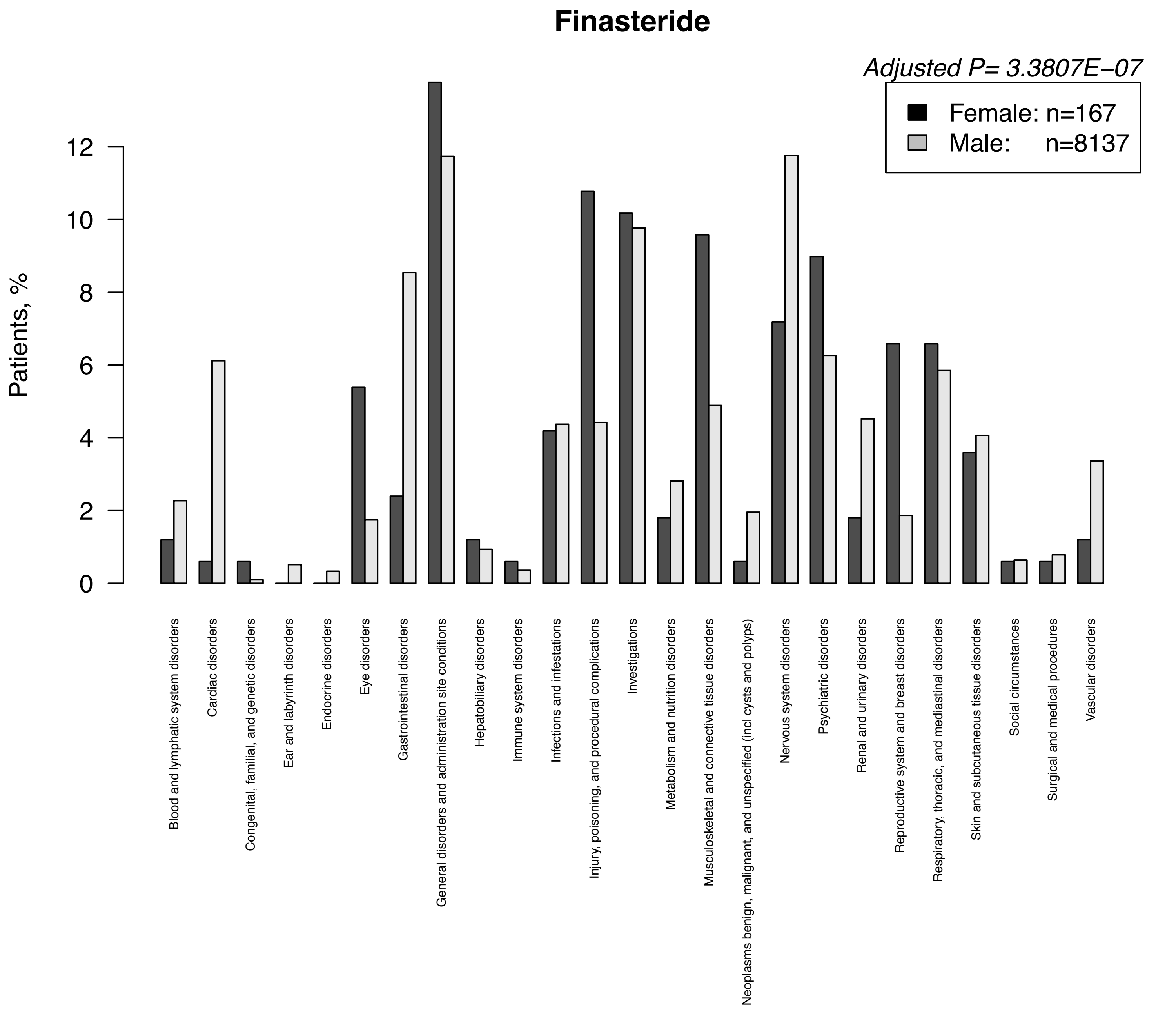

Finasteride
Adjusted P= 3.3807E−07
Female: n=167
Male: n=8137
Patients, %
0
2
4
6
8
10
12
Blood and lymphatic system disorders
Cardiac disorders
Congenital, familial, and genetic disorders
Ear and labyrinth disorders
Endocrine disorders
Eye disorders
Gastrointestinal disorders
General disorders and administration site conditions
Hepatobiliary disorders
Immune system disorders
Infections and infestations
Injury, poisoning, and procedural complications
Investigations
Metabolism and nutrition disorders
Musculoskeletal and connective tissue disorders
Neoplasms benign, malignant, and unspecified (incl cysts and polyps)
Nervous system disorders
Psychiatric disorders
Renal and urinary disorders
Reproductive system and breast disorders
Respiratory, thoracic, and mediastinal disorders
Skin and subcutaneous tissue disorders
Social circumstances
Surgical and medical procedures
Vascular disorders

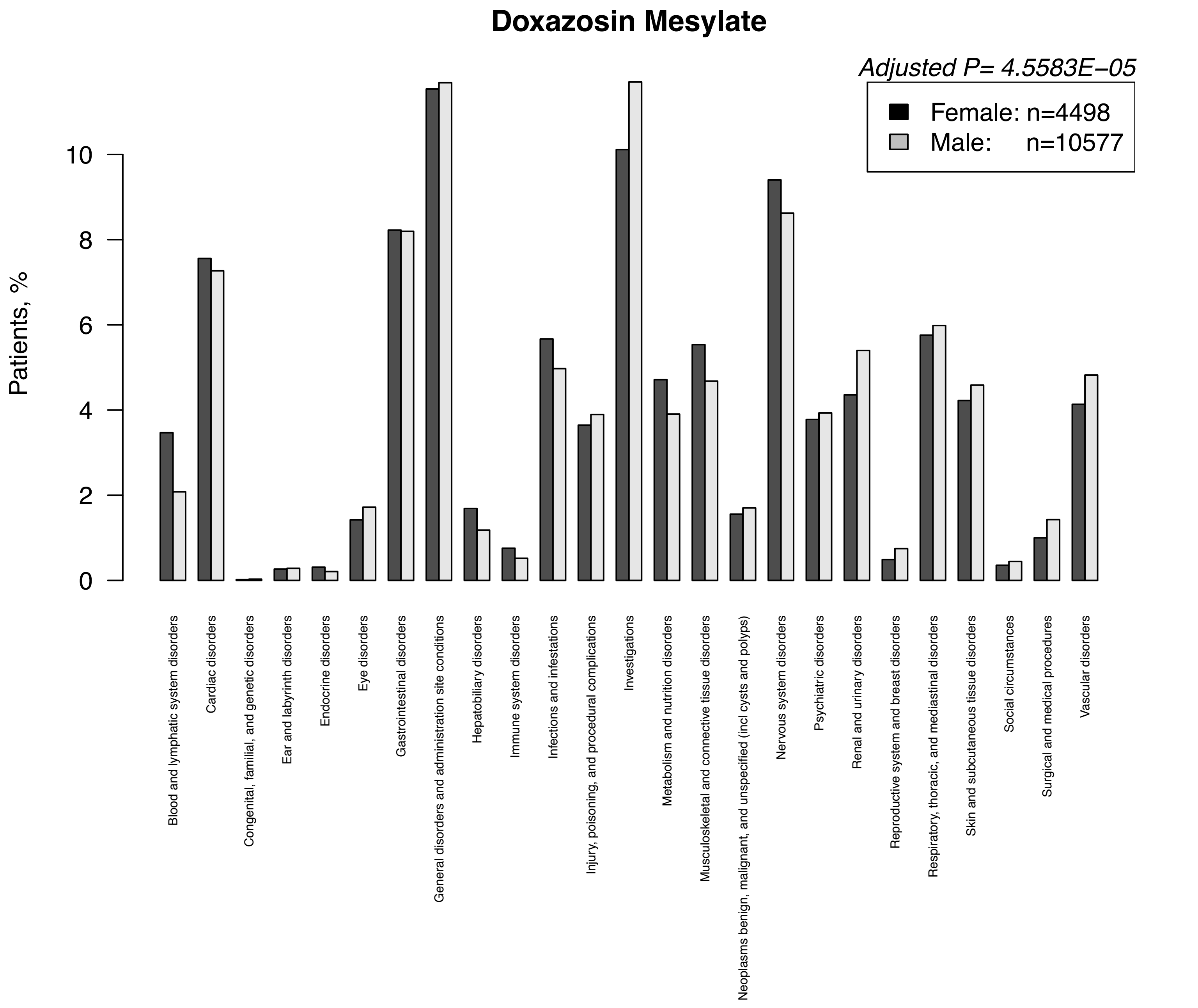
Doxazosin Mesylate
Adjusted P= 4.5583E−05
Female: n=4498
Male: n=10577
Patients, %
0
2
4
6
8
10
Blood and lymphatic system disorders
Cardiac disorders
Congenital, familial, and genetic disorders
Ear and labyrinth disorders
Endocrine disorders
Eye disorders
Gastrointestinal disorders
General disorders and administration site conditions
Hepatobiliary disorders
Immune system disorders
Infections and infestations
Injury, poisoning, and procedural complications
Investigations
Metabolism and nutrition disorders
Musculoskeletal and connective tissue disorders
Neoplasms benign, malignant, and unspecified (incl cysts and polyps)
Nervous system disorders
Psychiatric disorders
Renal and urinary disorders
Reproductive system and breast disorders
Respiratory, thoracic, and mediastinal disorders
Skin and subcutaneous tissue disorders
Social circumstances
Surgical and medical procedures
Vascular disorders

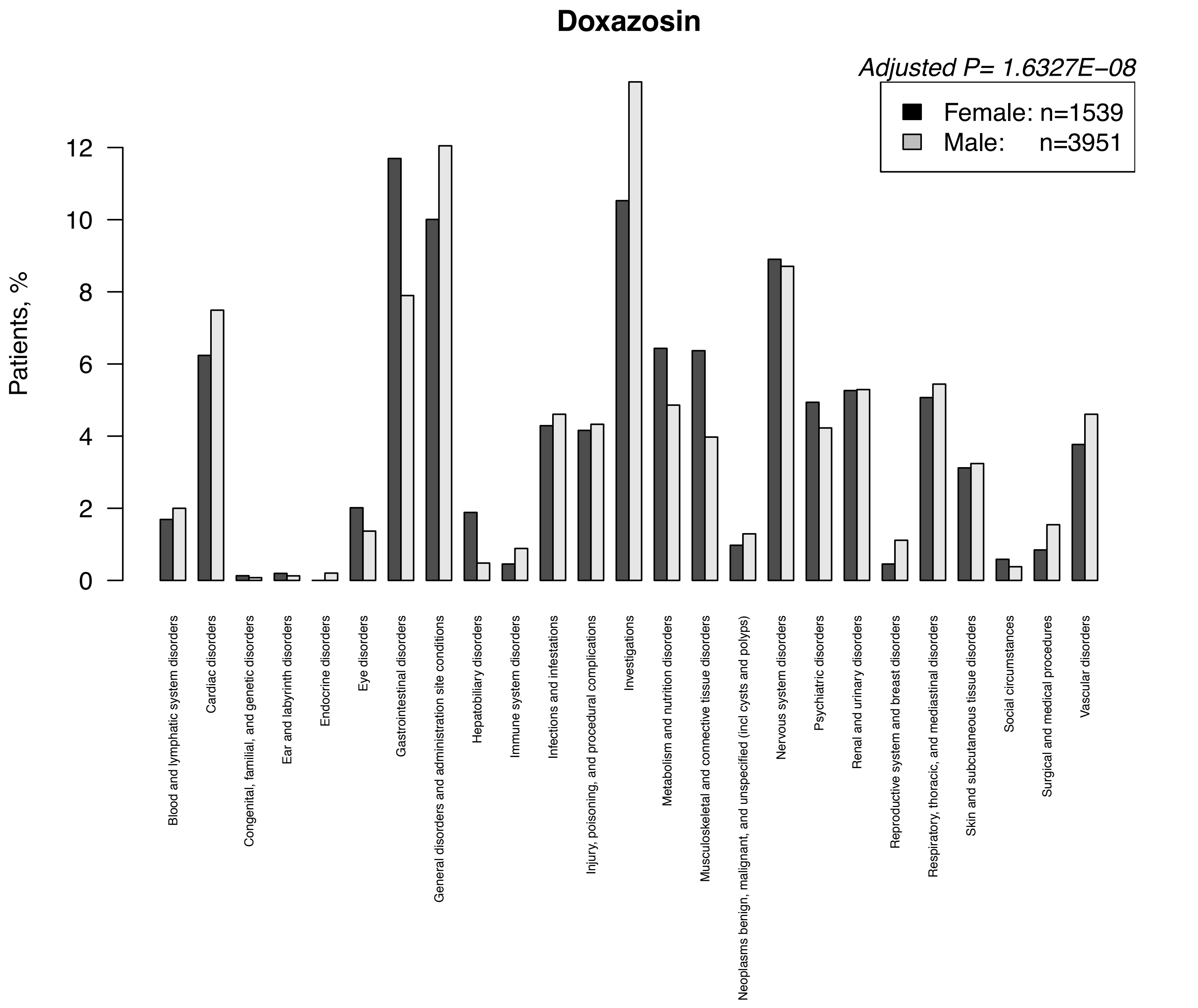
Doxazosin
Adjusted P= 1.6327E−08
Female: n=1539
Male: n=3951
Patients, %
0
2
4
6
8
10
12
Blood and lymphatic system disorders
Cardiac disorders
Congenital, familial, and genetic disorders
Ear and labyrinth disorders
Endocrine disorders
Eye disorders
Gastrointestinal disorders
General disorders and administration site conditions
Hepatobiliary disorders
Immune system disorders
Infections and infestations
Injury, poisoning, and procedural complications
Investigations
Metabolism and nutrition disorders
Musculoskeletal and connective tissue disorders
Neoplasms benign, malignant, and unspecified (incl cysts and polyps)
Nervous system disorders
Psychiatric disorders
Renal and urinary disorders
Reproductive system and breast disorders
Respiratory, thoracic, and mediastinal disorders
Skin and subcutaneous tissue disorders
Social circumstances
Surgical and medical procedures
Vascular disorders

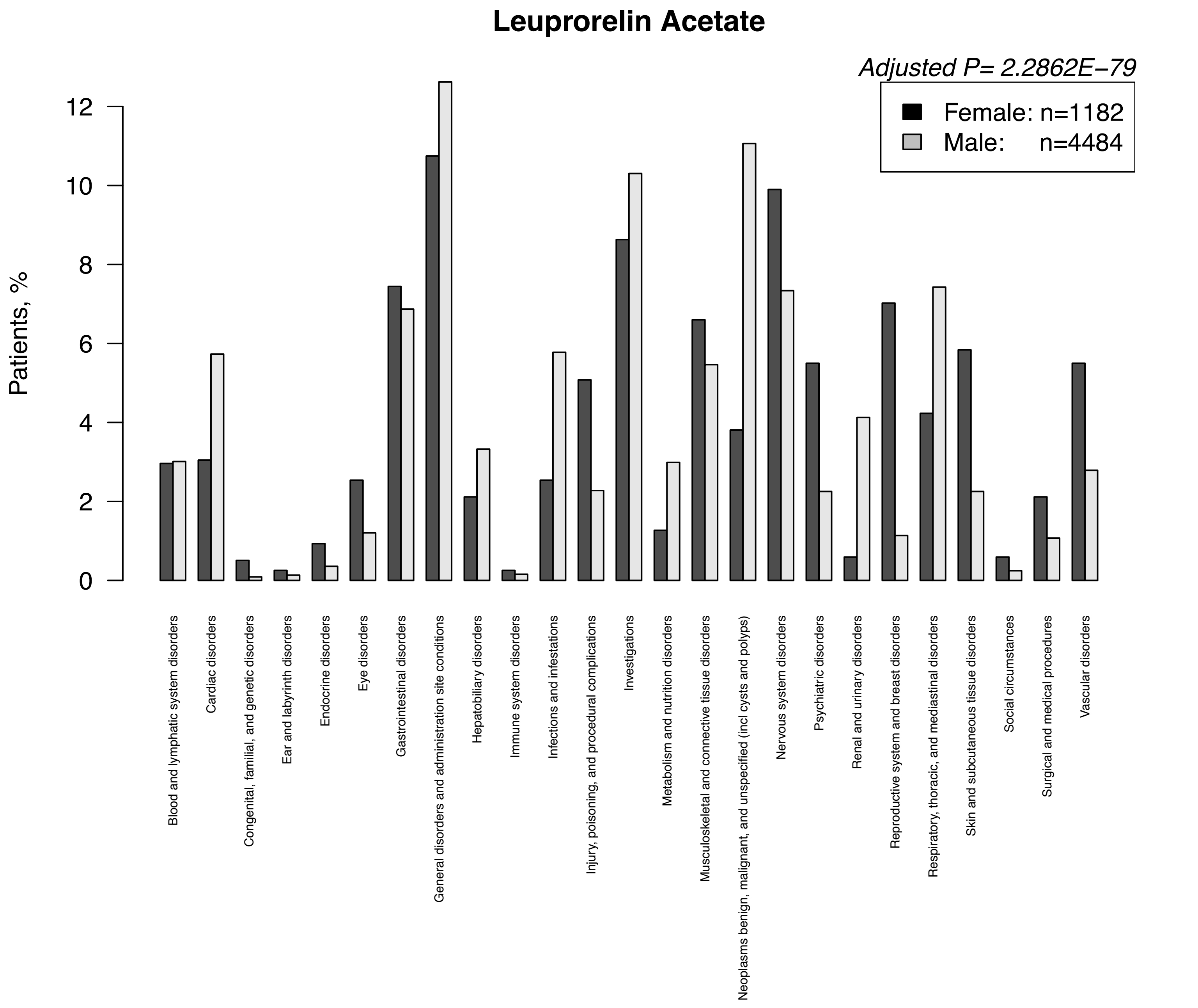

Leuprorelin Acetate
Adjusted P= 2.2862E−79
Female: n=1182
Male: n=4484
Patients, %
0
2
4
6
8
10
12
Blood and lymphatic system disorders
Cardiac disorders
Congenital, familial, and genetic disorders
Ear and labyrinth disorders
Endocrine disorders
Eye disorders
Gastrointestinal disorders
General disorders and administration site conditions
Hepatobiliary disorders
Immune system disorders
Infections and infestations
Injury, poisoning, and procedural complications
Investigations
Metabolism and nutrition disorders
Musculoskeletal and connective tissue disorders
Neoplasms benign, malignant, and unspecified (incl cysts and polyps)
Nervous system disorders
Psychiatric disorders
Renal and urinary disorders
Reproductive system and breast disorders
Respiratory, thoracic, and mediastinal disorders
Skin and subcutaneous tissue disorders
Social circumstances
Surgical and medical procedures
Vascular disorders

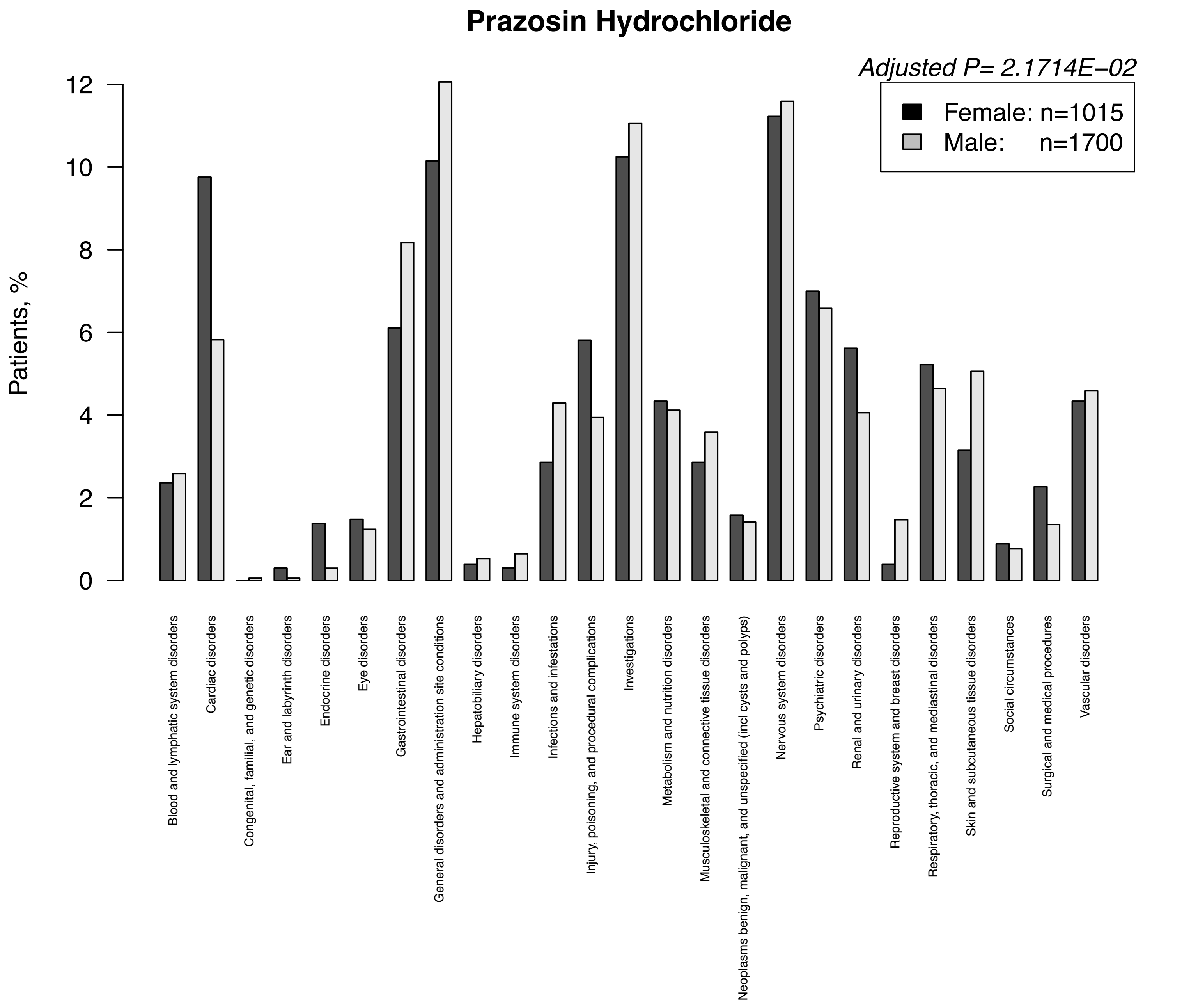
Prazosin Hydrochloride
Adjusted P= 2.1714E−02
Female: n=1015
Male: n=1700
Patients, %
0
2
4
6
8
10
12
Blood and lymphatic system disorders
Cardiac disorders
Congenital, familial, and genetic disorders
Ear and labyrinth disorders
Endocrine disorders
Eye disorders
Gastrointestinal disorders
General disorders and administration site conditions
Hepatobiliary disorders
Immune system disorders
Infections and infestations
Injury, poisoning, and procedural complications
Investigations
Metabolism and nutrition disorders
Musculoskeletal and connective tissue disorders
Neoplasms benign, malignant, and unspecified (incl cysts and polyps)
Nervous system disorders
Psychiatric disorders
Renal and urinary disorders
Reproductive system and breast disorders
Respiratory, thoracic, and mediastinal disorders
Skin and subcutaneous tissue disorders
Social circumstances
Surgical and medical procedures
Vascular disorders

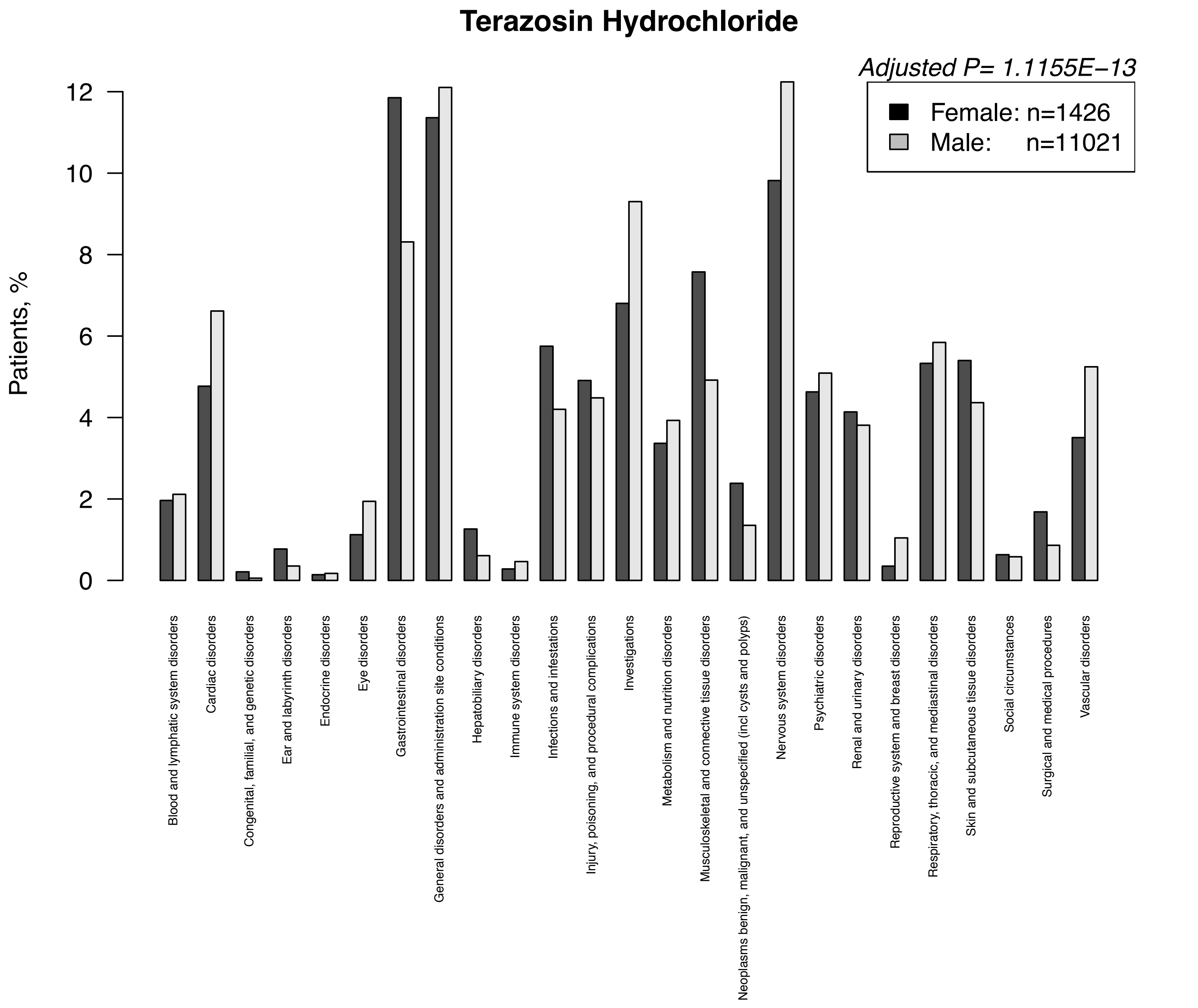
Terazosin Hydrochloride
Adjusted P= 1.1155E−13
Female: n=1426
Male: n=11021
Patients, %
0
2
4
6
8
10
12
Blood and lymphatic system disorders
Cardiac disorders
Congenital, familial, and genetic disorders
Ear and labyrinth disorders
Endocrine disorders
Eye disorders
Gastrointestinal disorders
General disorders and administration site conditions
Hepatobiliary disorders
Immune system disorders
Infections and infestations
Injury, poisoning, and procedural complications
Investigations
Metabolism and nutrition disorders
Musculoskeletal and connective tissue disorders
Neoplasms benign, malignant, and unspecified (incl cysts and polyps)
Nervous system disorders
Psychiatric disorders
Renal and urinary disorders
Reproductive system and breast disorders
Respiratory, thoracic, and mediastinal disorders
Skin and subcutaneous tissue disorders
Social circumstances
Surgical and medical procedures
Vascular disorders

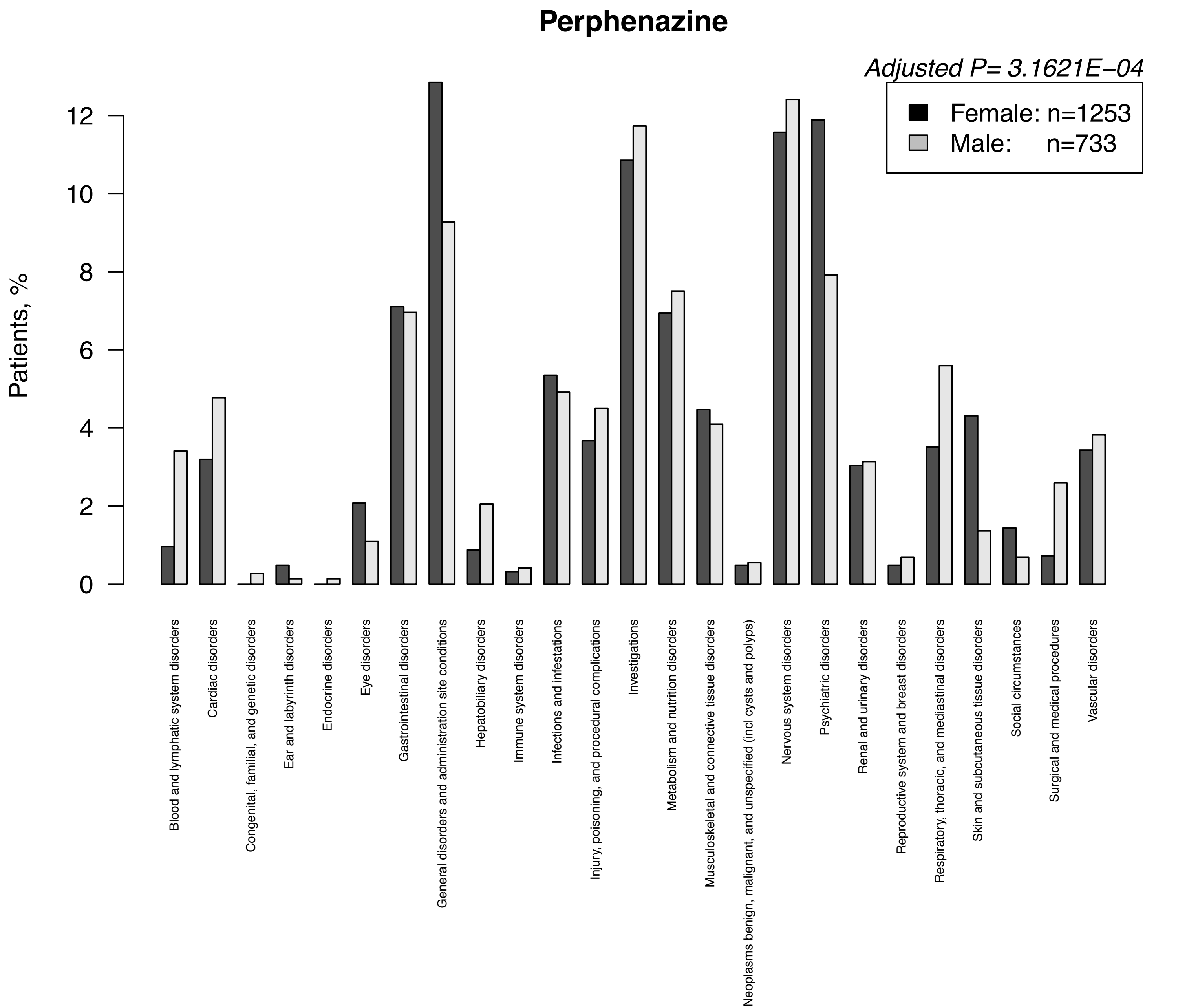
Perphenazine
Adjusted P= 3.1621E−04
Female: n=1253
Male: n=733
Patients, %
0
2
4
6
8
10
12
Blood and lymphatic system disorders
Cardiac disorders
Congenital, familial, and genetic disorders
Ear and labyrinth disorders
Endocrine disorders
Eye disorders
Gastrointestinal disorders
General disorders and administration site conditions
Hepatobiliary disorders
Immune system disorders
Infections and infestations
Injury, poisoning, and procedural complications
Investigations
Metabolism and nutrition disorders
Musculoskeletal and connective tissue disorders
Neoplasms benign, malignant, and unspecified (incl cysts and polyps)
Nervous system disorders
Psychiatric disorders
Renal and urinary disorders
Reproductive system and breast disorders
Respiratory, thoracic, and mediastinal disorders
Skin and subcutaneous tissue disorders
Social circumstances
Surgical and medical procedures
Vascular disorders

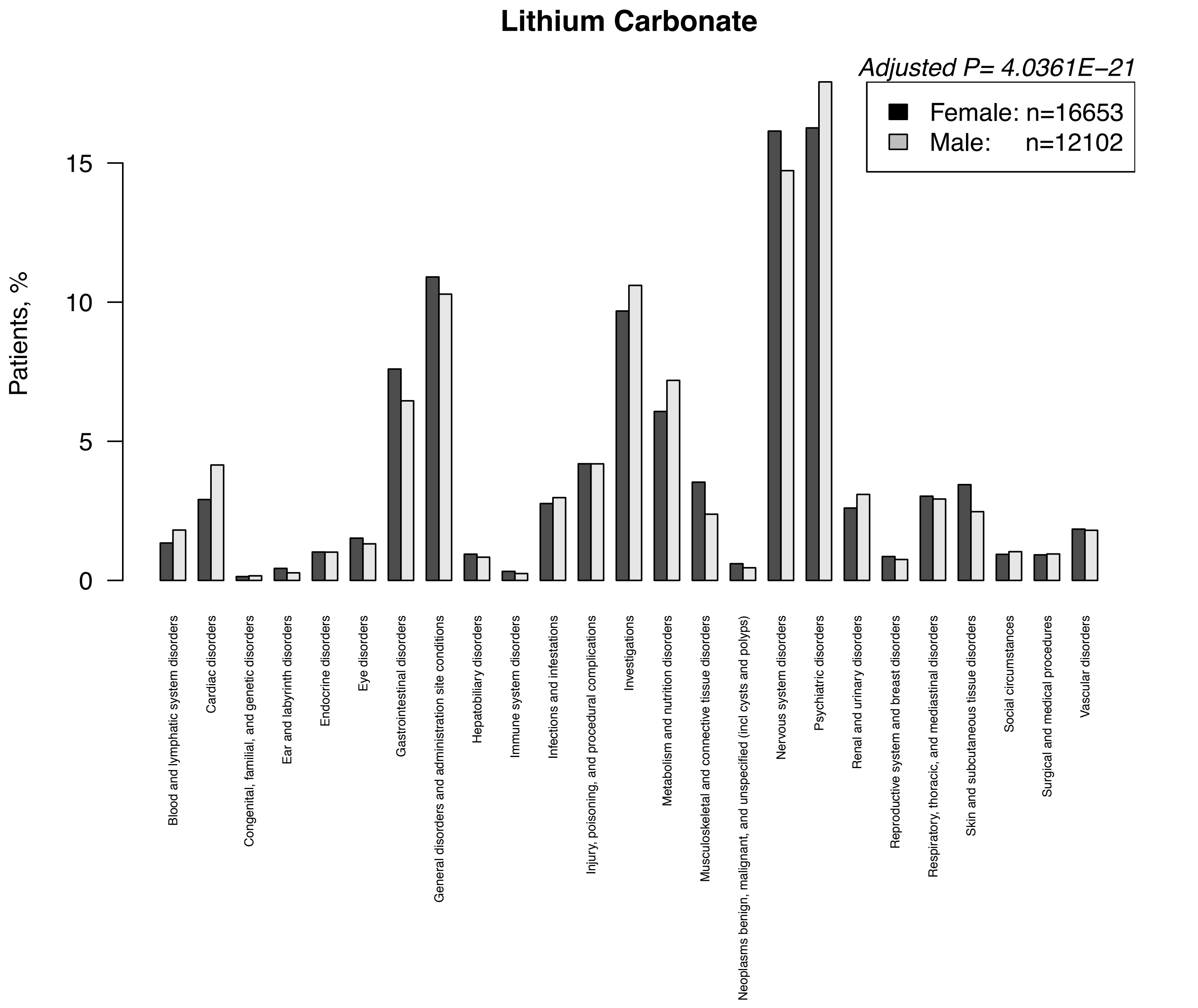
Lithium Carbonate
Adjusted P= 4.0361E−21
Female: n=16653
Male: n=12102
Patients, %
0
5
10
15
Blood and lymphatic system disorders
Cardiac disorders
Congenital, familial, and genetic disorders
Ear and labyrinth disorders
Endocrine disorders
Eye disorders
Gastrointestinal disorders
General disorders and administration site conditions
Hepatobiliary disorders
Immune system disorders
Infections and infestations
Injury, poisoning, and procedural complications
Investigations
Metabolism and nutrition disorders
Musculoskeletal and connective tissue disorders
Neoplasms benign, malignant, and unspecified (incl cysts and polyps)
Nervous system disorders
Psychiatric disorders
Renal and urinary disorders
Reproductive system and breast disorders
Respiratory, thoracic, and mediastinal disorders
Skin and subcutaneous tissue disorders
Social circumstances
Surgical and medical procedures
Vascular disorders

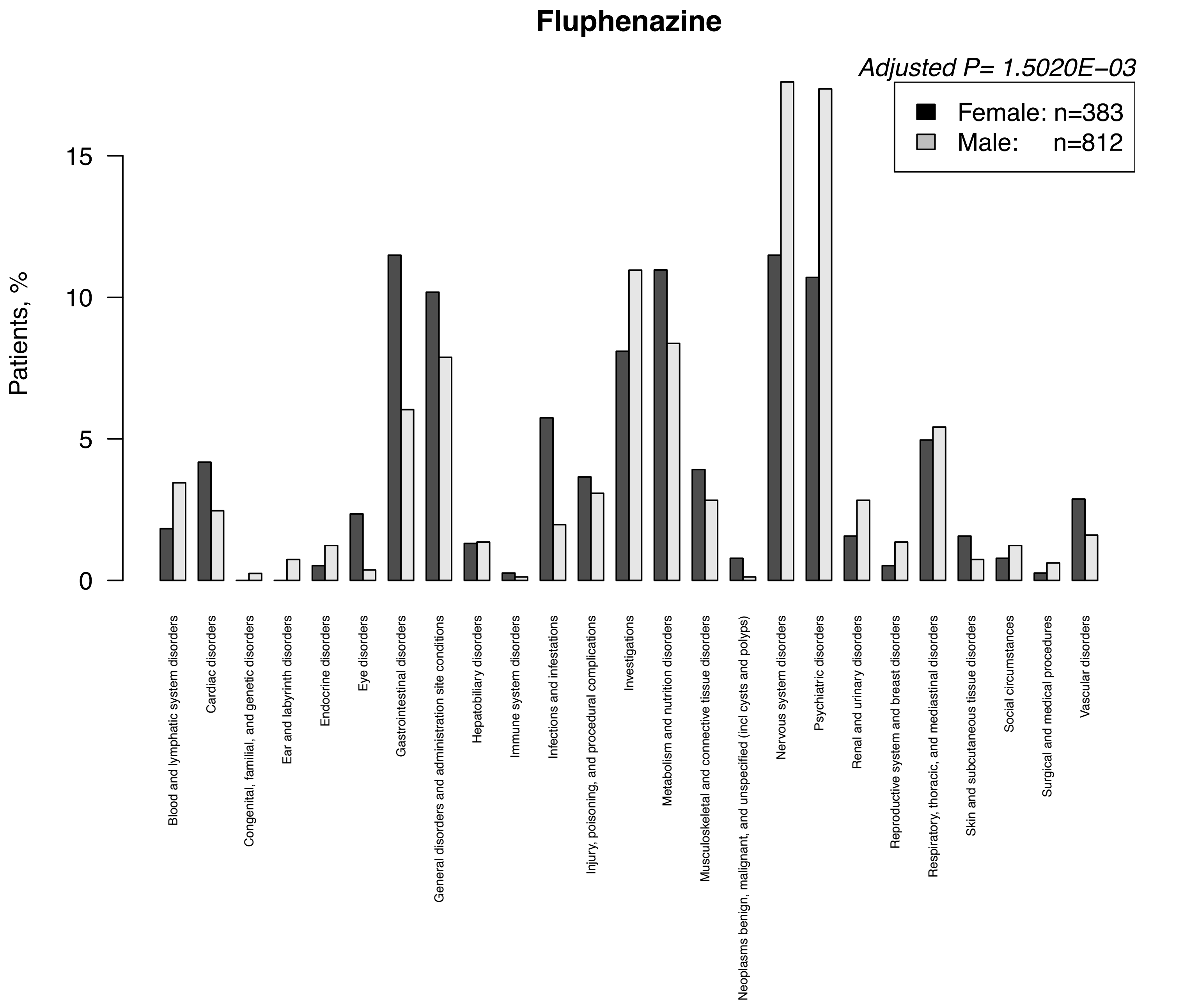
Fluphenazine
Adjusted P= 1.5020E−03
Female: n=383
Male: n=812
Patients, %
0
5
10
15
Blood and lymphatic system disorders
Cardiac disorders
Congenital, familial, and genetic disorders
Ear and labyrinth disorders
Endocrine disorders
Eye disorders
Gastrointestinal disorders
General disorders and administration site conditions
Hepatobiliary disorders
Immune system disorders
Infections and infestations
Injury, poisoning, and procedural complications
Investigations
Metabolism and nutrition disorders
Musculoskeletal and connective tissue disorders
Neoplasms benign, malignant, and unspecified (incl cysts and polyps)
Nervous system disorders
Psychiatric disorders
Renal and urinary disorders
Reproductive system and breast disorders
Respiratory, thoracic, and mediastinal disorders
Skin and subcutaneous tissue disorders
Social circumstances
Surgical and medical procedures
Vascular disorders

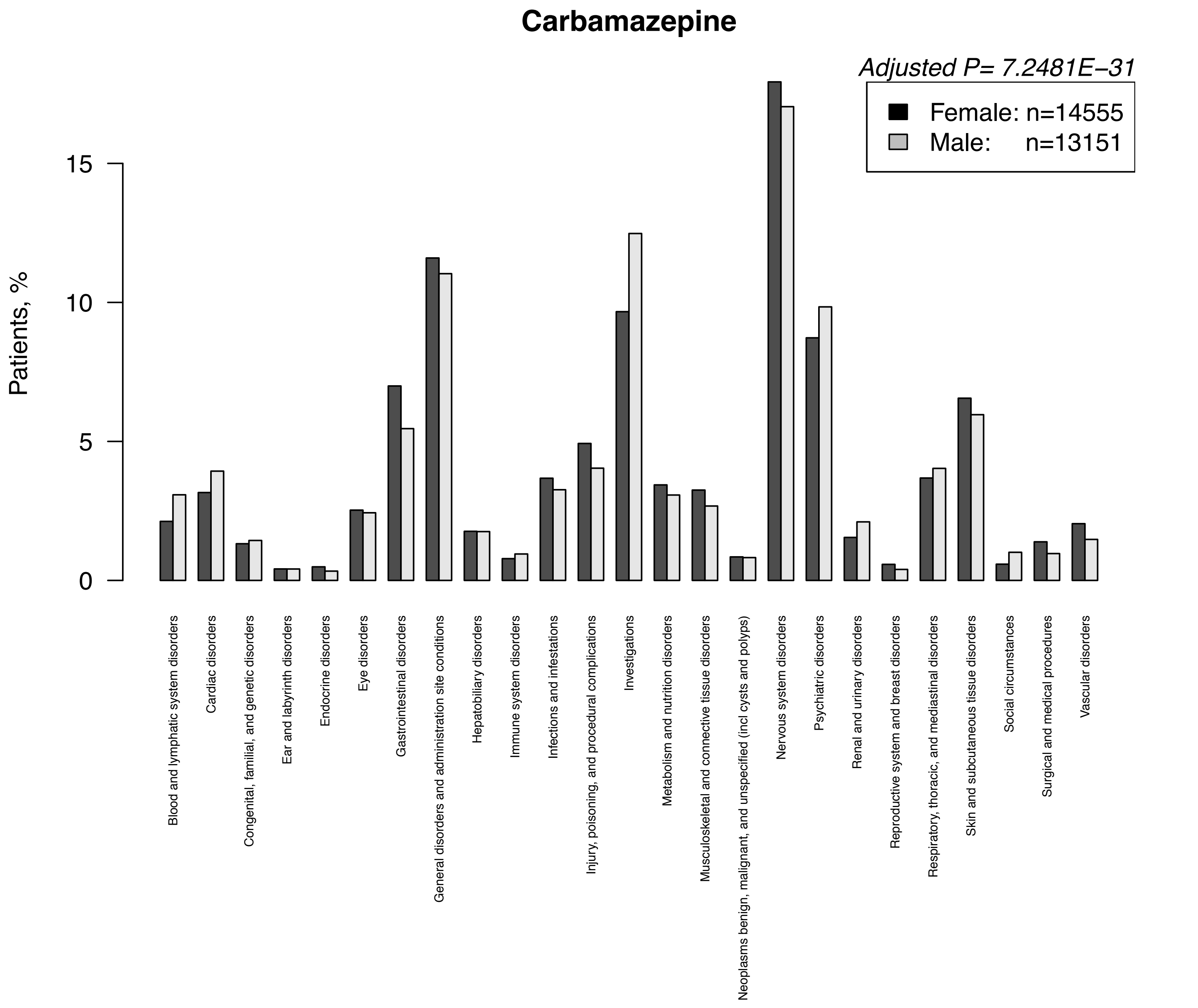

Carbamazepine
Adjusted P= 7.2481E−31
Female: n=14555
Male: n=13151
Patients, %
0
5
10
15
Blood and lymphatic system disorders
Cardiac disorders
Congenital, familial, and genetic disorders
Ear and labyrinth disorders
Endocrine disorders
Eye disorders
Gastrointestinal disorders
General disorders and administration site conditions
Hepatobiliary disorders
Immune system disorders
Infections and infestations
Injury, poisoning, and procedural complications
Investigations
Metabolism and nutrition disorders
Musculoskeletal and connective tissue disorders
Neoplasms benign, malignant, and unspecified (incl cysts and polyps)
Nervous system disorders
Psychiatric disorders
Renal and urinary disorders
Reproductive system and breast disorders
Respiratory, thoracic, and mediastinal disorders
Skin and subcutaneous tissue disorders
Social circumstances
Surgical and medical procedures
Vascular disorders

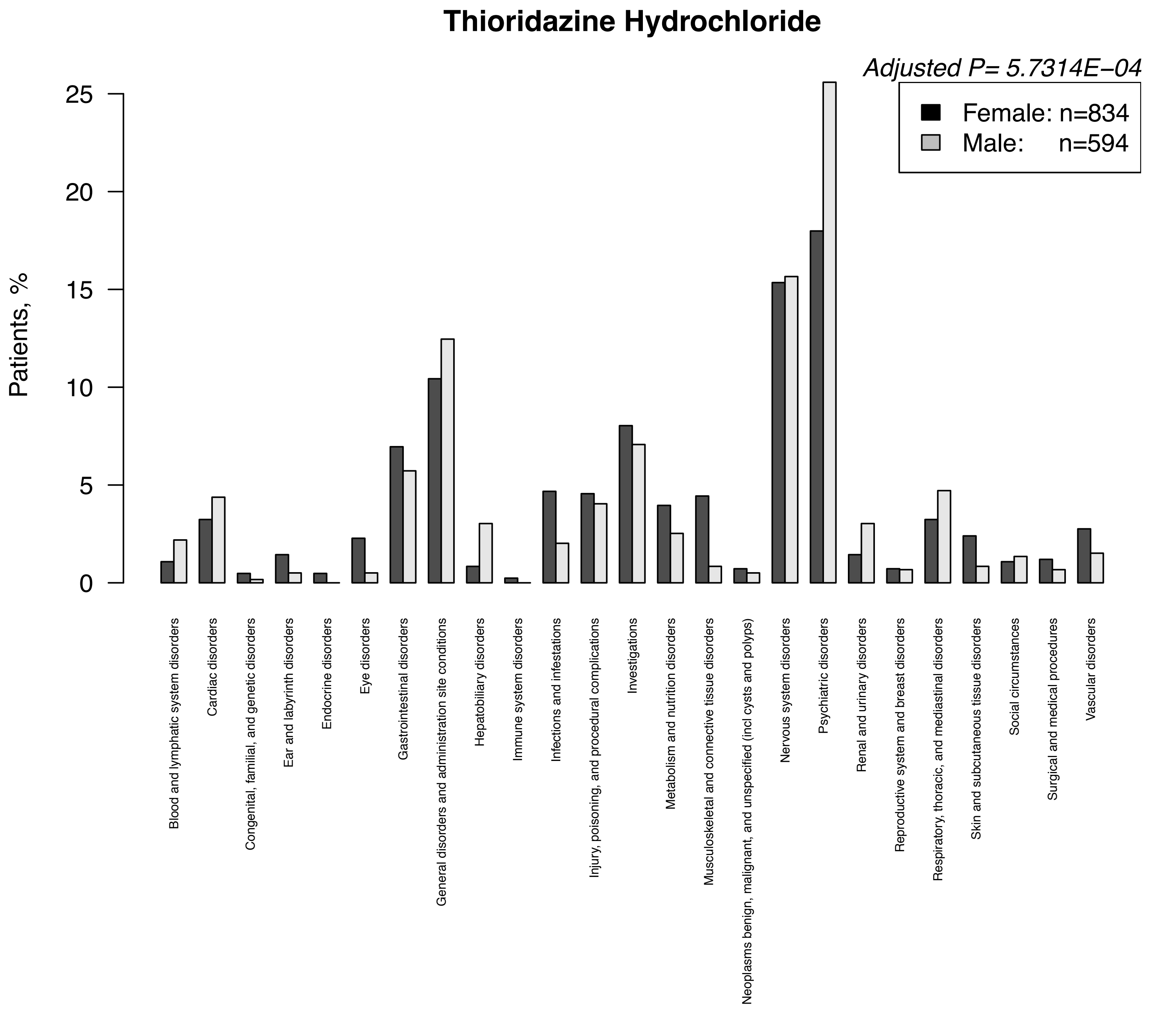

Thioridazine Hydrochloride
Adjusted P= 5.7314E−04
Female: n=834
Male: n=594
Patients, %
0
5
10
15
20
25
Blood and lymphatic system disorders
Cardiac disorders
Congenital, familial, and genetic disorders
Ear and labyrinth disorders
Endocrine disorders
Eye disorders
Gastrointestinal disorders
General disorders and administration site conditions
Hepatobiliary disorders
Immune system disorders
Infections and infestations
Injury, poisoning, and procedural complications
Investigations
Metabolism and nutrition disorders
Musculoskeletal and connective tissue disorders
Neoplasms benign, malignant, and unspecified (incl cysts and polyps)
Nervous system disorders
Psychiatric disorders
Renal and urinary disorders
Reproductive system and breast disorders
Respiratory, thoracic, and mediastinal disorders
Skin and subcutaneous tissue disorders
Social circumstances
Surgical and medical procedures
Vascular disorders

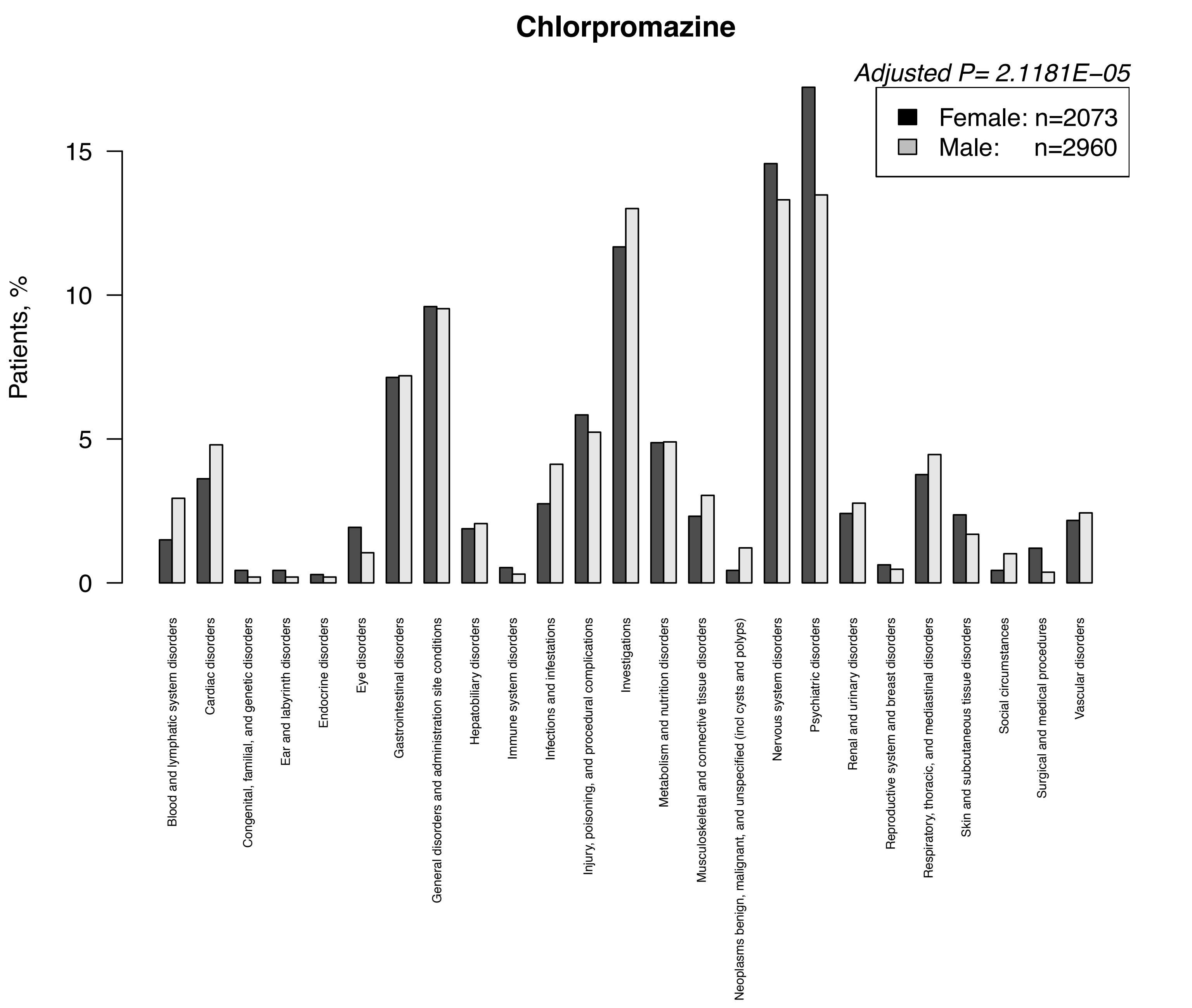

Chlorpromazine
Adjusted P= 2.1181E−05
Female: n=2073
Male: n=2960
Patients, %
0
5
10
15
Blood and lymphatic system disorders
Cardiac disorders
Congenital, familial, and genetic disorders
Ear and labyrinth disorders
Endocrine disorders
Eye disorders
Gastrointestinal disorders
General disorders and administration site conditions
Hepatobiliary disorders
Immune system disorders
Infections and infestations
Injury, poisoning, and procedural complications
Investigations
Metabolism and nutrition disorders
Musculoskeletal and connective tissue disorders
Neoplasms benign, malignant, and unspecified (incl cysts and polyps)
Nervous system disorders
Psychiatric disorders
Renal and urinary disorders
Reproductive system and breast disorders
Respiratory, thoracic, and mediastinal disorders
Skin and subcutaneous tissue disorders
Social circumstances
Surgical and medical procedures
Vascular disorders

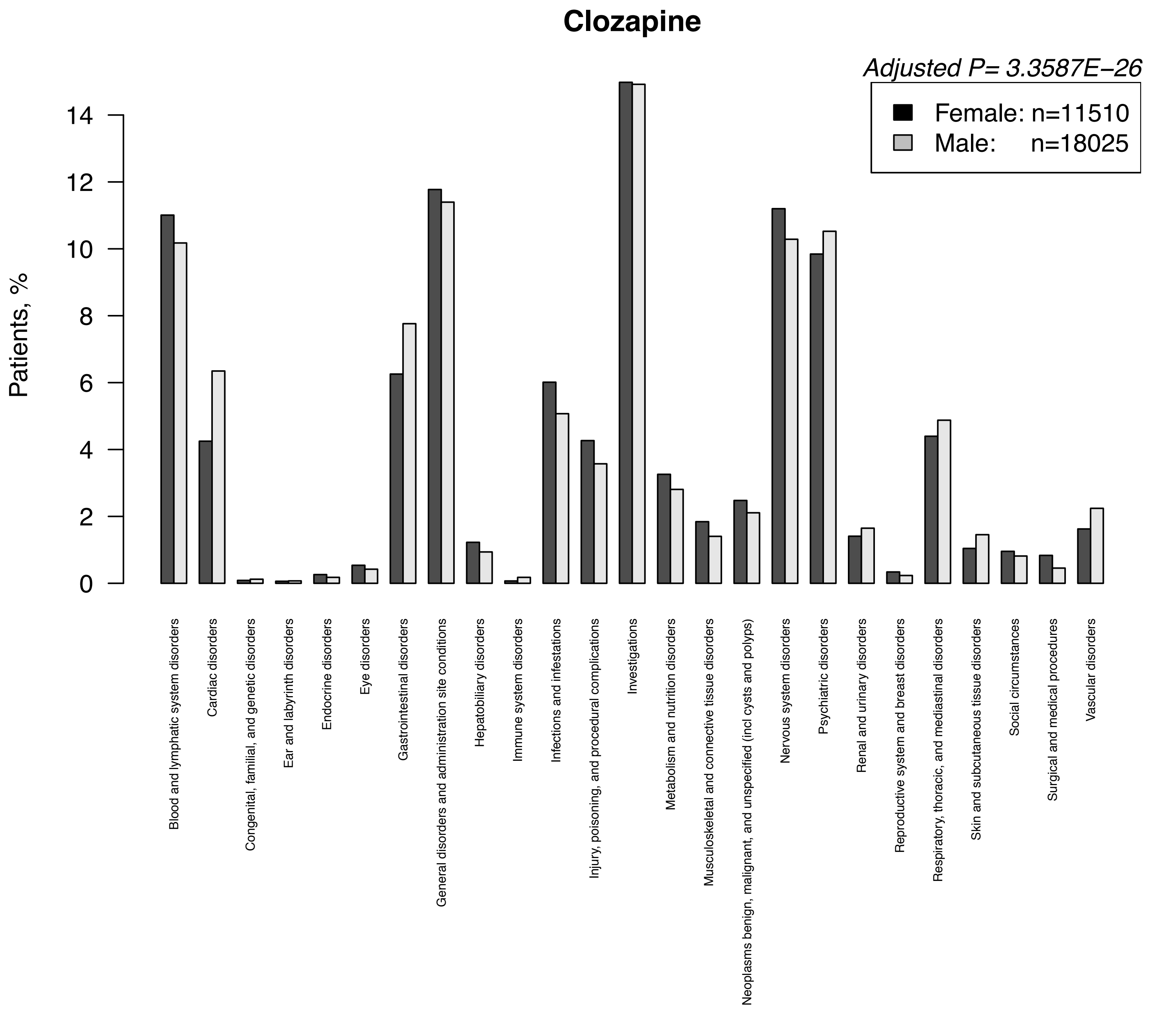
Clozapine
Adjusted P= 3.3587E−26
Female: n=11510
Male: n=18025
Patients, %
0
2
4
6
8
10
12
14
Blood and lymphatic system disorders
Cardiac disorders
Congenital, familial, and genetic disorders
Ear and labyrinth disorders
Endocrine disorders
Eye disorders
Gastrointestinal disorders
General disorders and administration site conditions
Hepatobiliary disorders
Immune system disorders
Infections and infestations
Injury, poisoning, and procedural complications
Investigations
Metabolism and nutrition disorders
Musculoskeletal and connective tissue disorders
Neoplasms benign, malignant, and unspecified (incl cysts and polyps)
Nervous system disorders
Psychiatric disorders
Renal and urinary disorders
Reproductive system and breast disorders
Respiratory, thoracic, and mediastinal disorders
Skin and subcutaneous tissue disorders
Social circumstances
Surgical and medical procedures
Vascular disorders

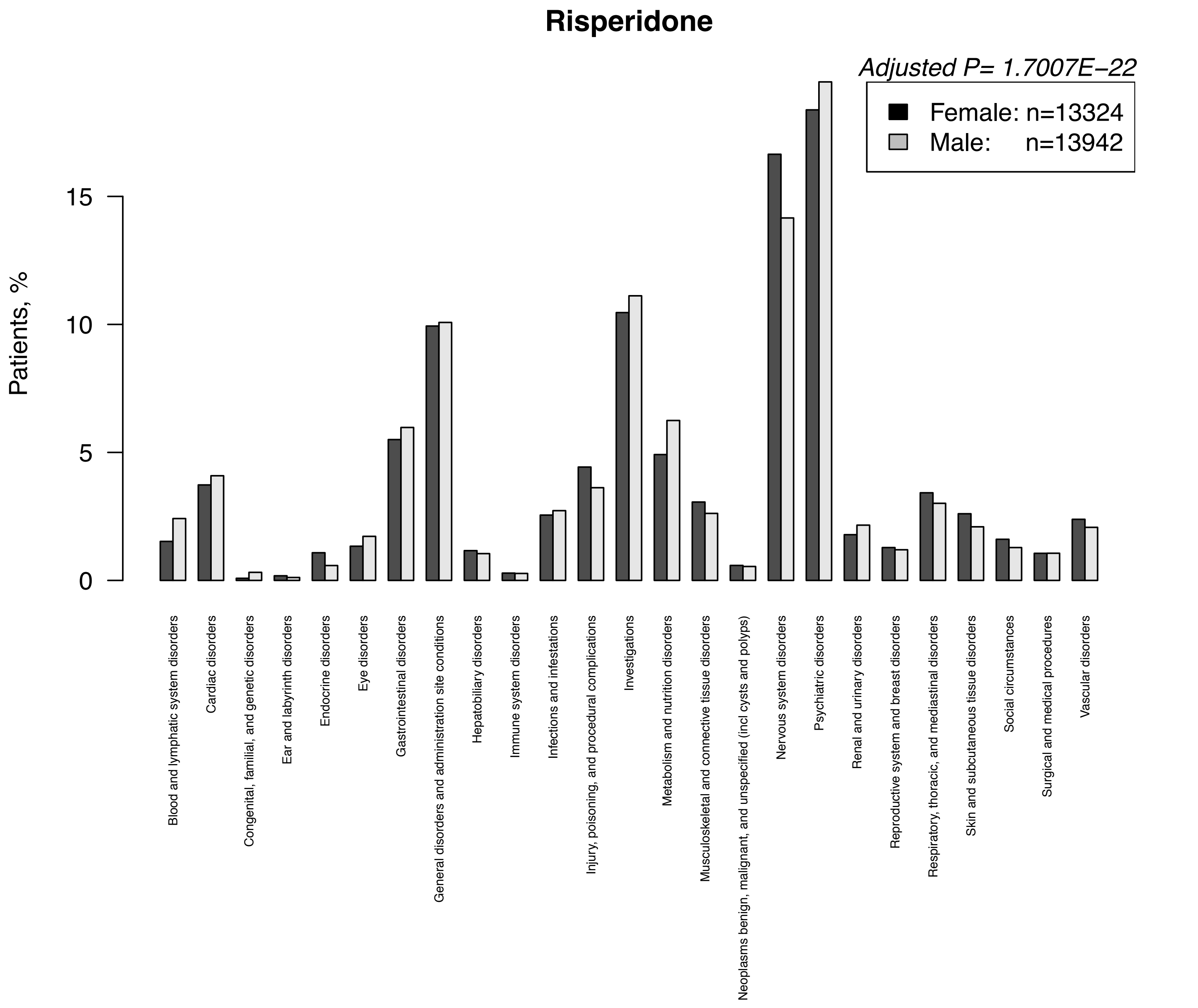
Risperidone
Adjusted P= 1.7007E−22
Female: n=13324
Male: n=13942
Patients, %
0
5
10
15
Blood and lymphatic system disorders
Cardiac disorders
Congenital, familial, and genetic disorders
Ear and labyrinth disorders
Endocrine disorders
Eye disorders
Gastrointestinal disorders
General disorders and administration site conditions
Hepatobiliary disorders
Immune system disorders
Infections and infestations
Injury, poisoning, and procedural complications
Investigations
Metabolism and nutrition disorders
Musculoskeletal and connective tissue disorders
Neoplasms benign, malignant, and unspecified (incl cysts and polyps)
Nervous system disorders
Psychiatric disorders
Renal and urinary disorders
Reproductive system and breast disorders
Respiratory, thoracic, and mediastinal disorders
Skin and subcutaneous tissue disorders
Social circumstances
Surgical and medical procedures
Vascular disorders

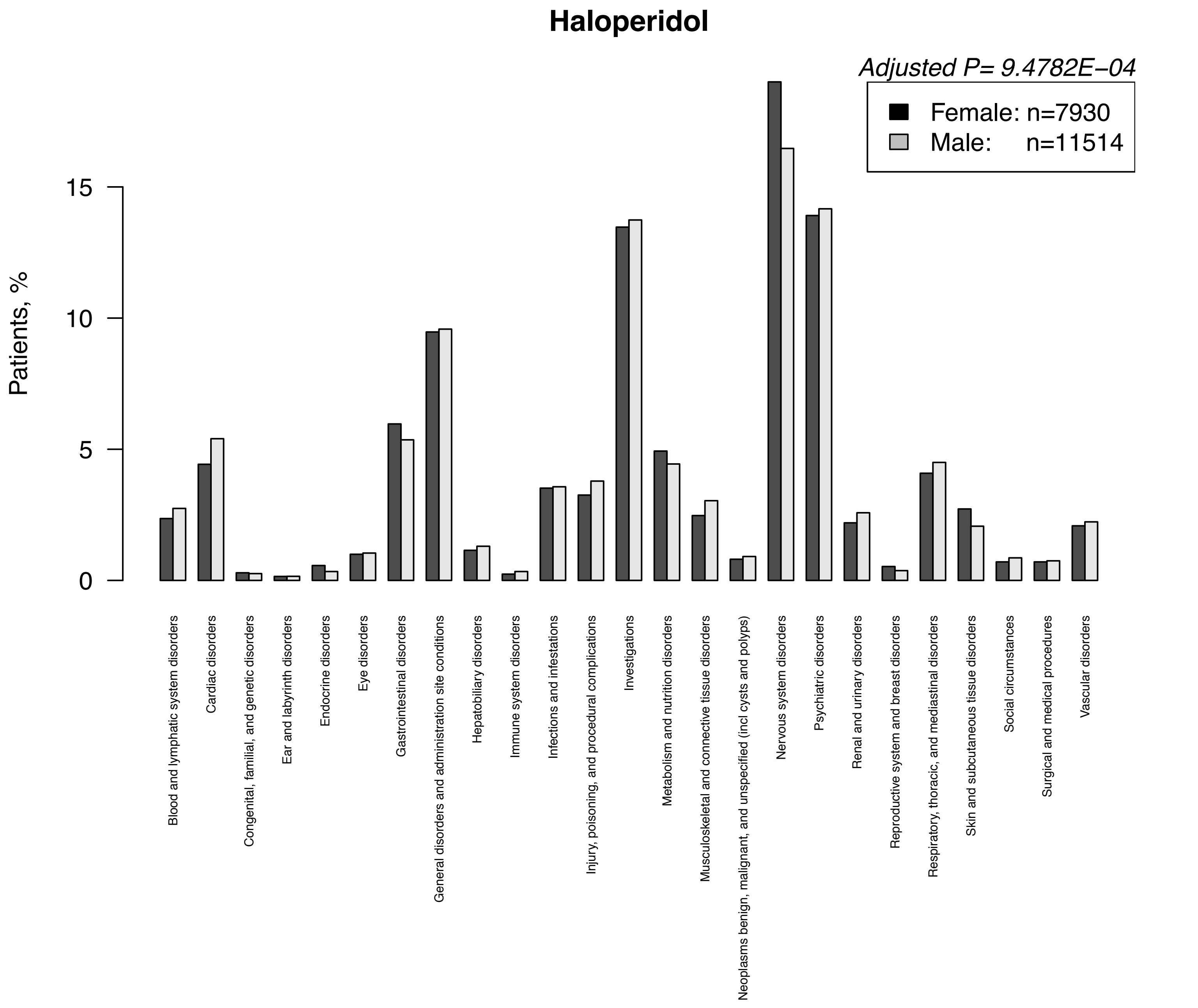

Haloperidol
Adjusted P= 9.4782E−04
Female: n=7930
Male: n=11514
Patients, %
0
5
10
15
Blood and lymphatic system disorders
Cardiac disorders
Congenital, familial, and genetic disorders
Ear and labyrinth disorders
Endocrine disorders
Eye disorders
Gastrointestinal disorders
General disorders and administration site conditions
Hepatobiliary disorders
Immune system disorders
Infections and infestations
Injury, poisoning, and procedural complications
Investigations
Metabolism and nutrition disorders
Musculoskeletal and connective tissue disorders
Neoplasms benign, malignant, and unspecified (incl cysts and polyps)
Nervous system disorders
Psychiatric disorders
Renal and urinary disorders
Reproductive system and breast disorders
Respiratory, thoracic, and mediastinal disorders
Skin and subcutaneous tissue disorders
Social circumstances
Surgical and medical procedures
Vascular disorders

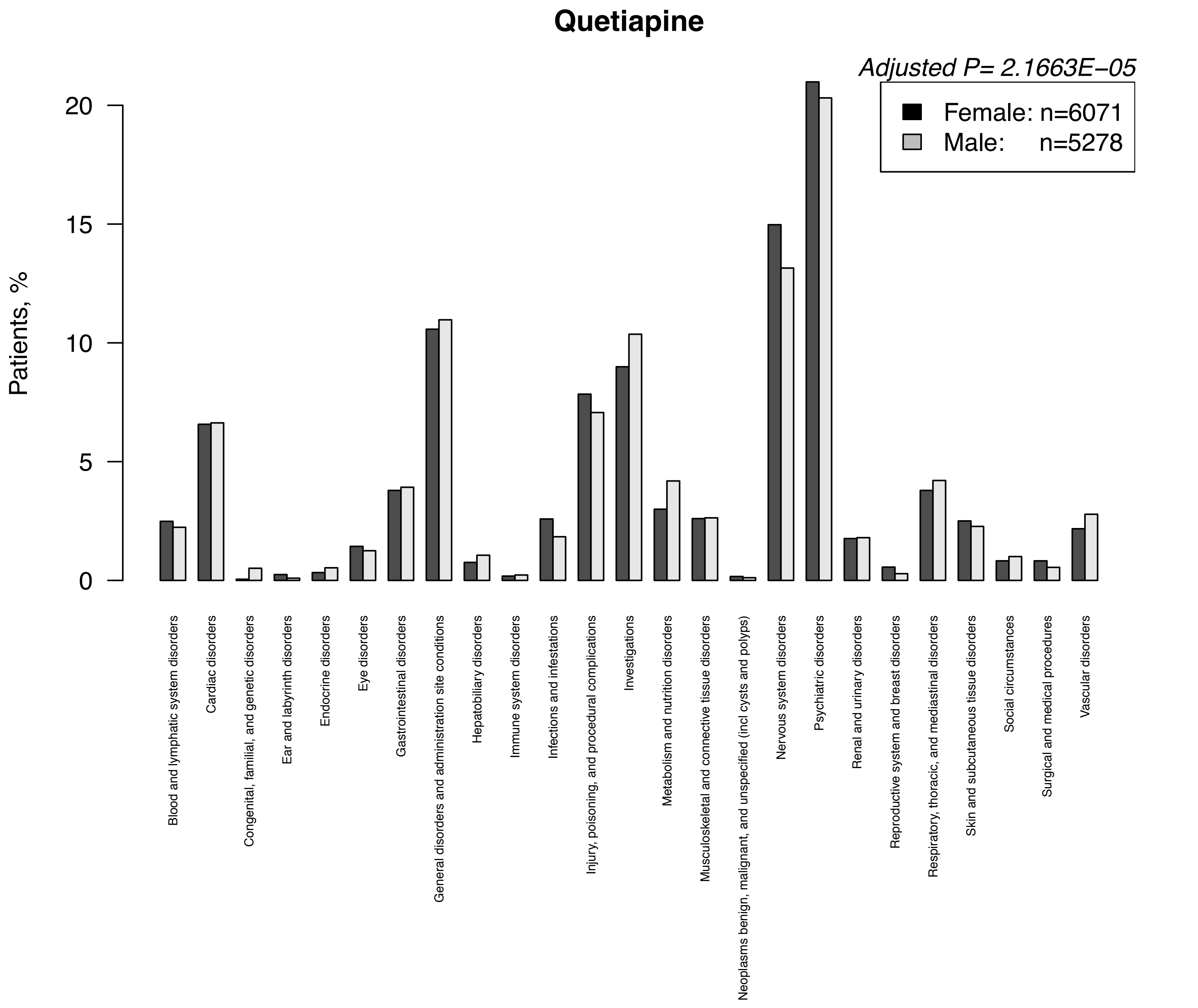
Quetiapine
Adjusted P= 2.1663E−05
Female: n=6071
Male: n=5278
Patients, %
0
5
10
15
20
Blood and lymphatic system disorders
Cardiac disorders
Congenital, familial, and genetic disorders
Ear and labyrinth disorders
Endocrine disorders
Eye disorders
Gastrointestinal disorders
General disorders and administration site conditions
Hepatobiliary disorders
Immune system disorders
Infections and infestations
Injury, poisoning, and procedural complications
Investigations
Metabolism and nutrition disorders
Musculoskeletal and connective tissue disorders
Neoplasms benign, malignant, and unspecified (incl cysts and polyps)
Nervous system disorders
Psychiatric disorders
Renal and urinary disorders
Reproductive system and breast disorders
Respiratory, thoracic, and mediastinal disorders
Skin and subcutaneous tissue disorders
Social circumstances
Surgical and medical procedures
Vascular disorders

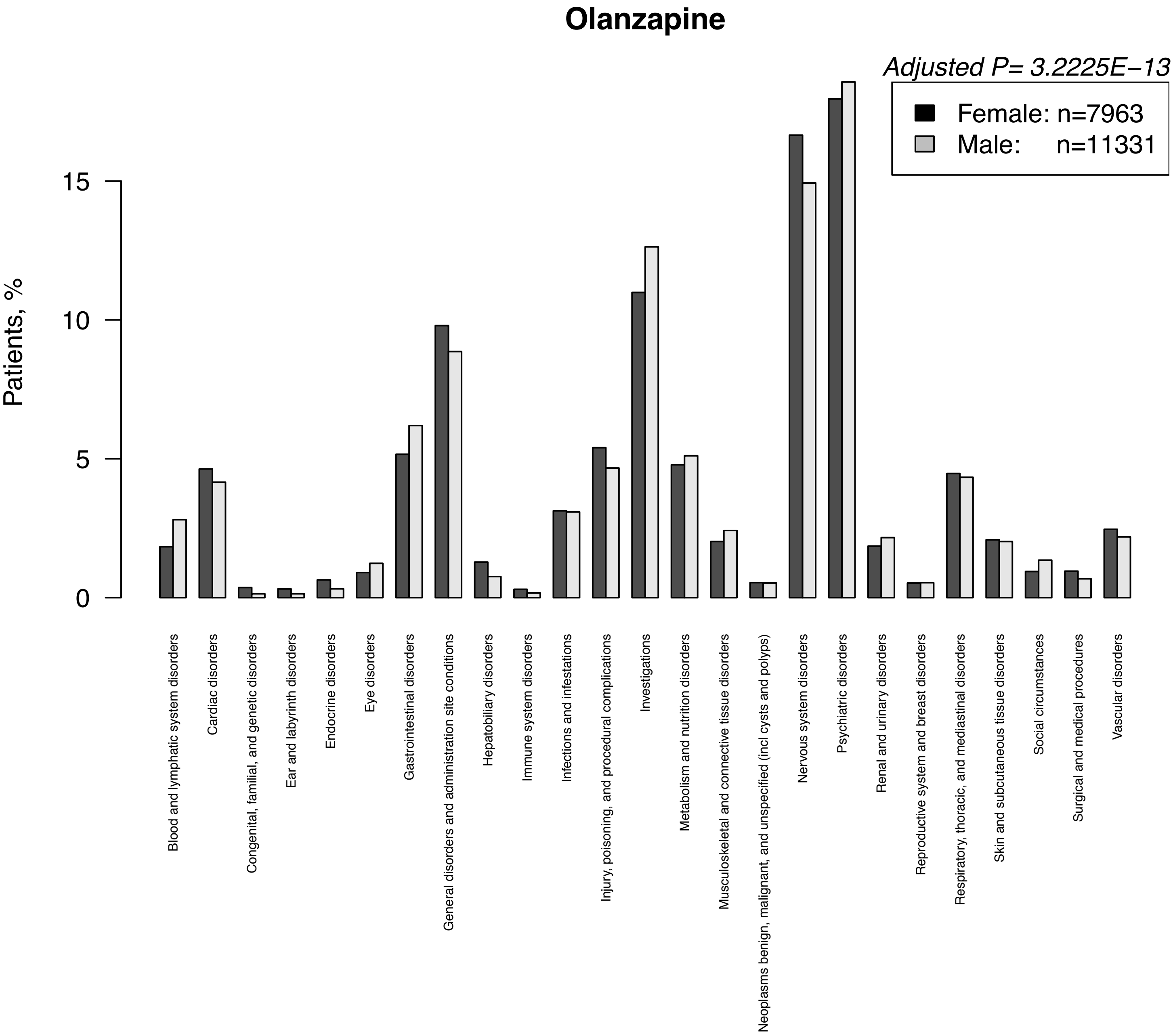
Olanzapine
Adjusted P= 3.2225E−13
Female: n=7963
Male: n=11331
Patients, %
0
5
10
15
Blood and lymphatic system disorders
Cardiac disorders
Congenital, familial, and genetic disorders
Ear and labyrinth disorders
Endocrine disorders
Eye disorders
Gastrointestinal disorders
General disorders and administration site conditions
Hepatobiliary disorders
Immune system disorders
Infections and infestations
Injury, poisoning, and procedural complications
Investigations
Metabolism and nutrition disorders
Musculoskeletal and connective tissue disorders
Neoplasms benign, malignant, and unspecified (incl cysts and polyps)
Nervous system disorders
Psychiatric disorders
Renal and urinary disorders
Reproductive system and breast disorders
Respiratory, thoracic, and mediastinal disorders
Skin and subcutaneous tissue disorders
Social circumstances
Surgical and medical procedures
Vascular disorders

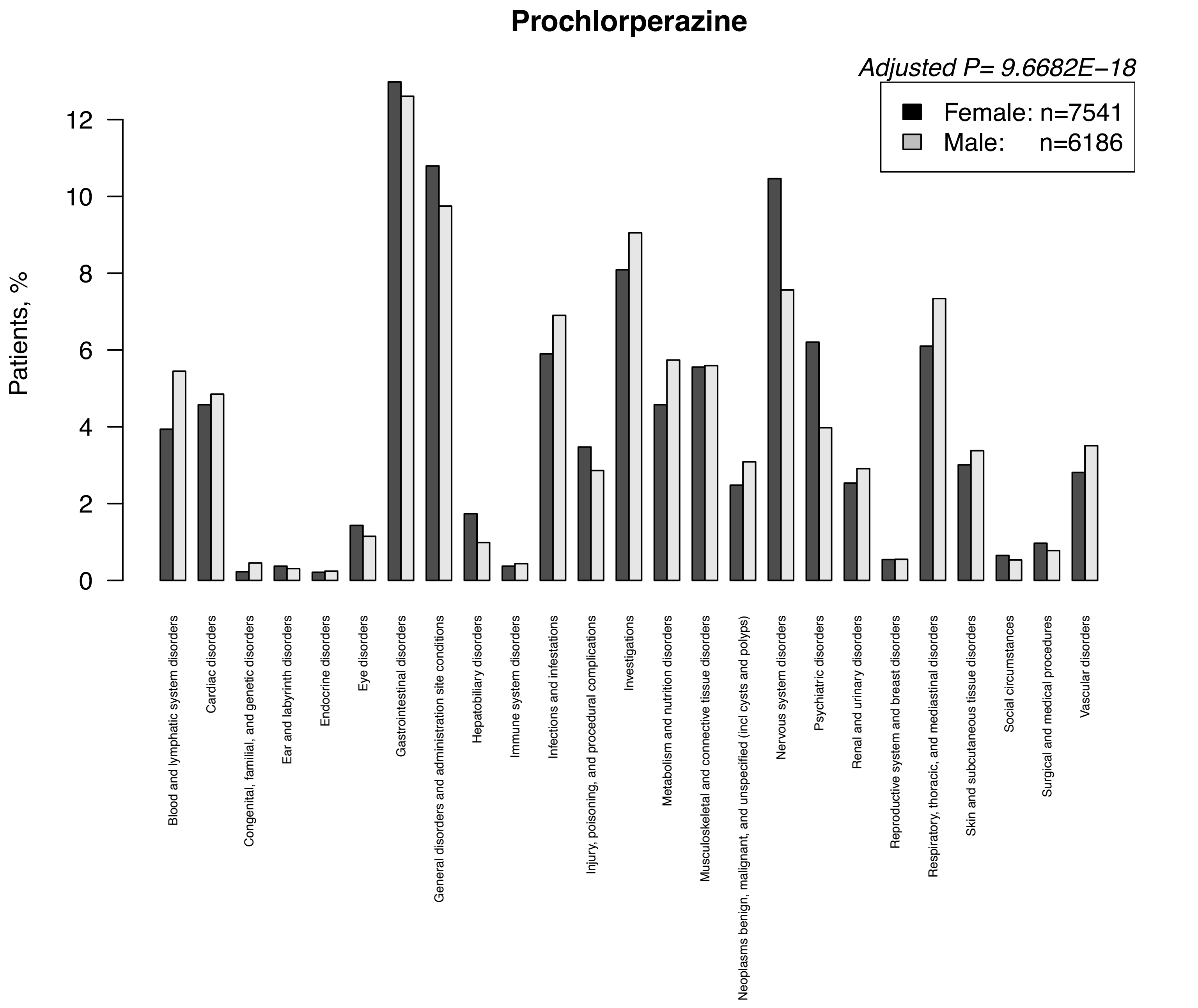
Prochlorperazine
Adjusted P= 9.6682E−18
Female: n=7541
Male: n=6186
Patients, %
0
2
4
6
8
10
12
Blood and lymphatic system disorders
Cardiac disorders
Congenital, familial, and genetic disorders
Ear and labyrinth disorders
Endocrine disorders
Eye disorders
Gastrointestinal disorders
General disorders and administration site conditions
Hepatobiliary disorders
Immune system disorders
Infections and infestations
Injury, poisoning, and procedural complications
Investigations
Metabolism and nutrition disorders
Musculoskeletal and connective tissue disorders
Neoplasms benign, malignant, and unspecified (incl cysts and polyps)
Nervous system disorders
Psychiatric disorders
Renal and urinary disorders
Reproductive system and breast disorders
Respiratory, thoracic, and mediastinal disorders
Skin and subcutaneous tissue disorders
Social circumstances
Surgical and medical procedures
Vascular disorders

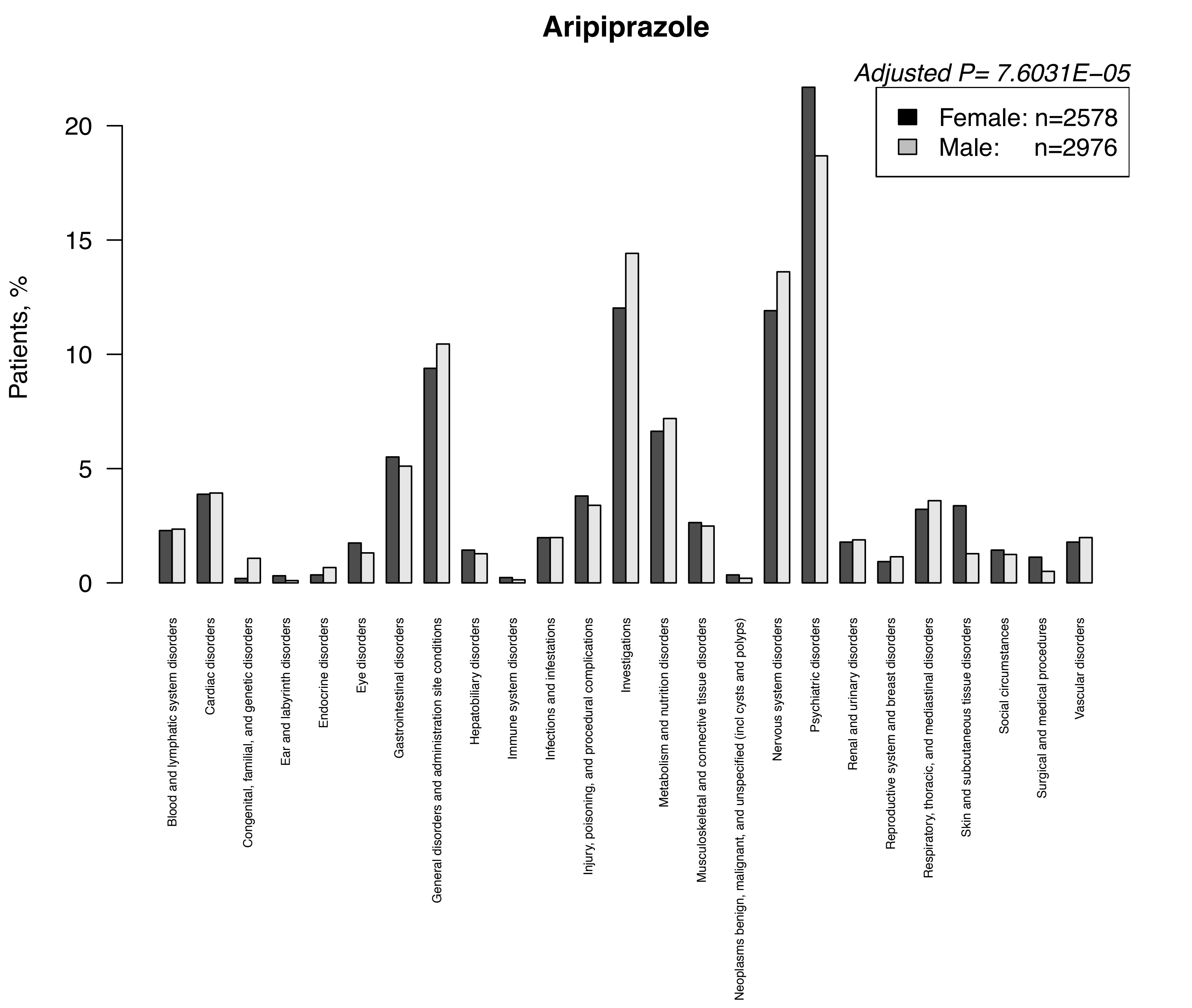

Aripiprazole
Adjusted P= 7.6031E−05
Female: n=2578
Male: n=2976
Patients, %
0
5
10
15
20
Blood and lymphatic system disorders
Cardiac disorders
Congenital, familial, and genetic disorders
Ear and labyrinth disorders
Endocrine disorders
Eye disorders
Gastrointestinal disorders
General disorders and administration site conditions
Hepatobiliary disorders
Immune system disorders
Infections and infestations
Injury, poisoning, and procedural complications
Investigations
Metabolism and nutrition disorders
Musculoskeletal and connective tissue disorders
Neoplasms benign, malignant, and unspecified (incl cysts and polyps)
Nervous system disorders
Psychiatric disorders
Renal and urinary disorders
Reproductive system and breast disorders
Respiratory, thoracic, and mediastinal disorders
Skin and subcutaneous tissue disorders
Social circumstances
Surgical and medical procedures
Vascular disorders

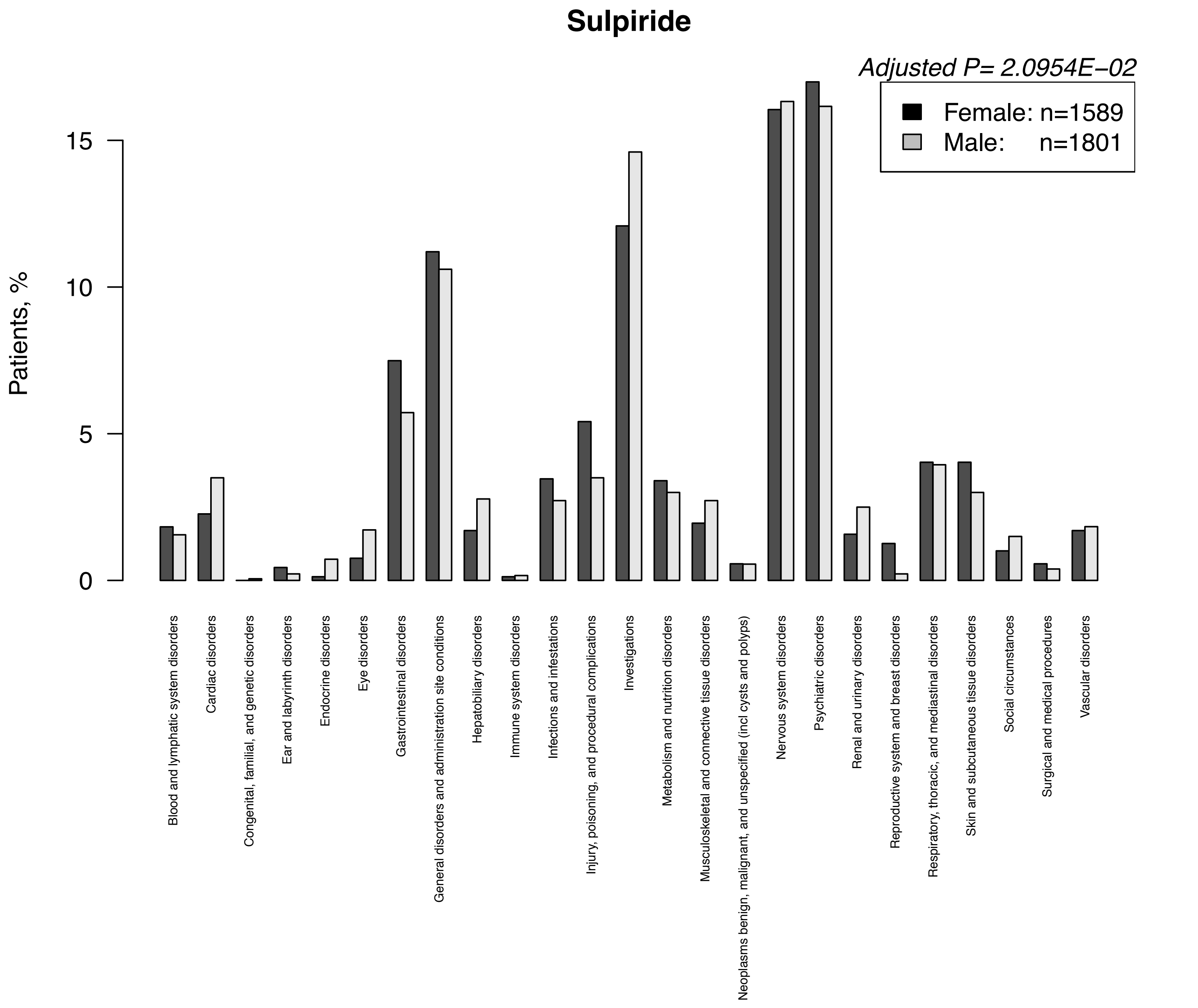

Sulpiride
Adjusted P= 2.0954E−02
Female: n=1589
Male: n=1801
Patients, %
0
5
10
15
Blood and lymphatic system disorders
Cardiac disorders
Congenital, familial, and genetic disorders
Ear and labyrinth disorders
Endocrine disorders
Eye disorders
Gastrointestinal disorders
General disorders and administration site conditions
Hepatobiliary disorders
Immune system disorders
Infections and infestations
Injury, poisoning, and procedural complications
Investigations
Metabolism and nutrition disorders
Musculoskeletal and connective tissue disorders
Neoplasms benign, malignant, and unspecified (incl cysts and polyps)
Nervous system disorders
Psychiatric disorders
Renal and urinary disorders
Reproductive system and breast disorders
Respiratory, thoracic, and mediastinal disorders
Skin and subcutaneous tissue disorders
Social circumstances
Surgical and medical procedures
Vascular disorders

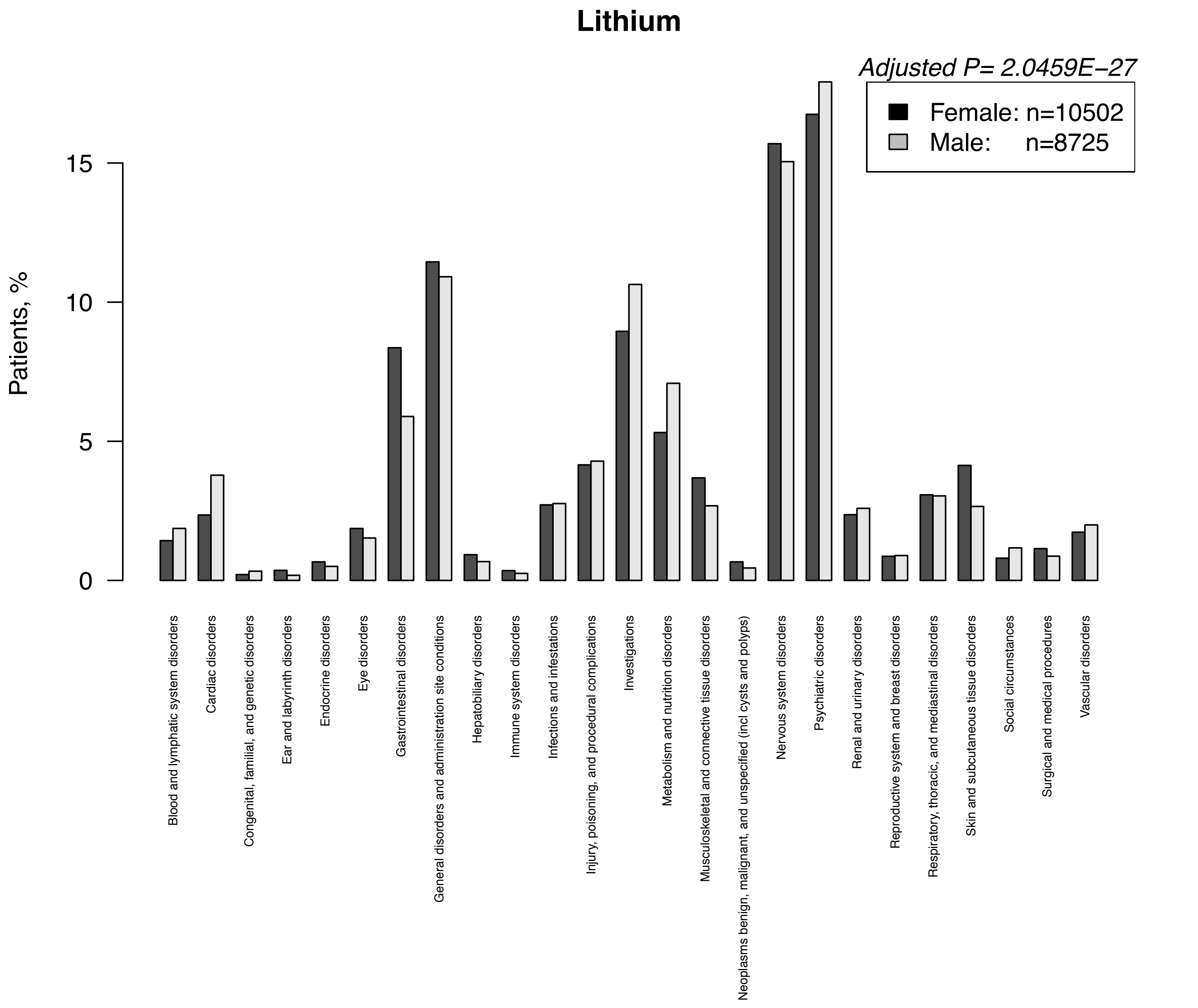

Lithium
Adjusted P= 2.0459E−27
Female: n=10502
Male: n=8725
Patients, %
0
5
10
15
Blood and lymphatic system disorders
Cardiac disorders
Congenital, familial, and genetic disorders
Ear and labyrinth disorders
Endocrine disorders
Eye disorders
Gastrointestinal disorders
General disorders and administration site conditions
Hepatobiliary disorders
Immune system disorders
Infections and infestations
Injury, poisoning, and procedural complications
Investigations
Metabolism and nutrition disorders
Musculoskeletal and connective tissue disorders
Neoplasms benign, malignant, and unspecified (incl cysts and polyps)
Nervous system disorders
Psychiatric disorders
Renal and urinary disorders
Reproductive system and breast disorders
Respiratory, thoracic, and mediastinal disorders
Skin and subcutaneous tissue disorders
Social circumstances
Surgical and medical procedures
Vascular disorders

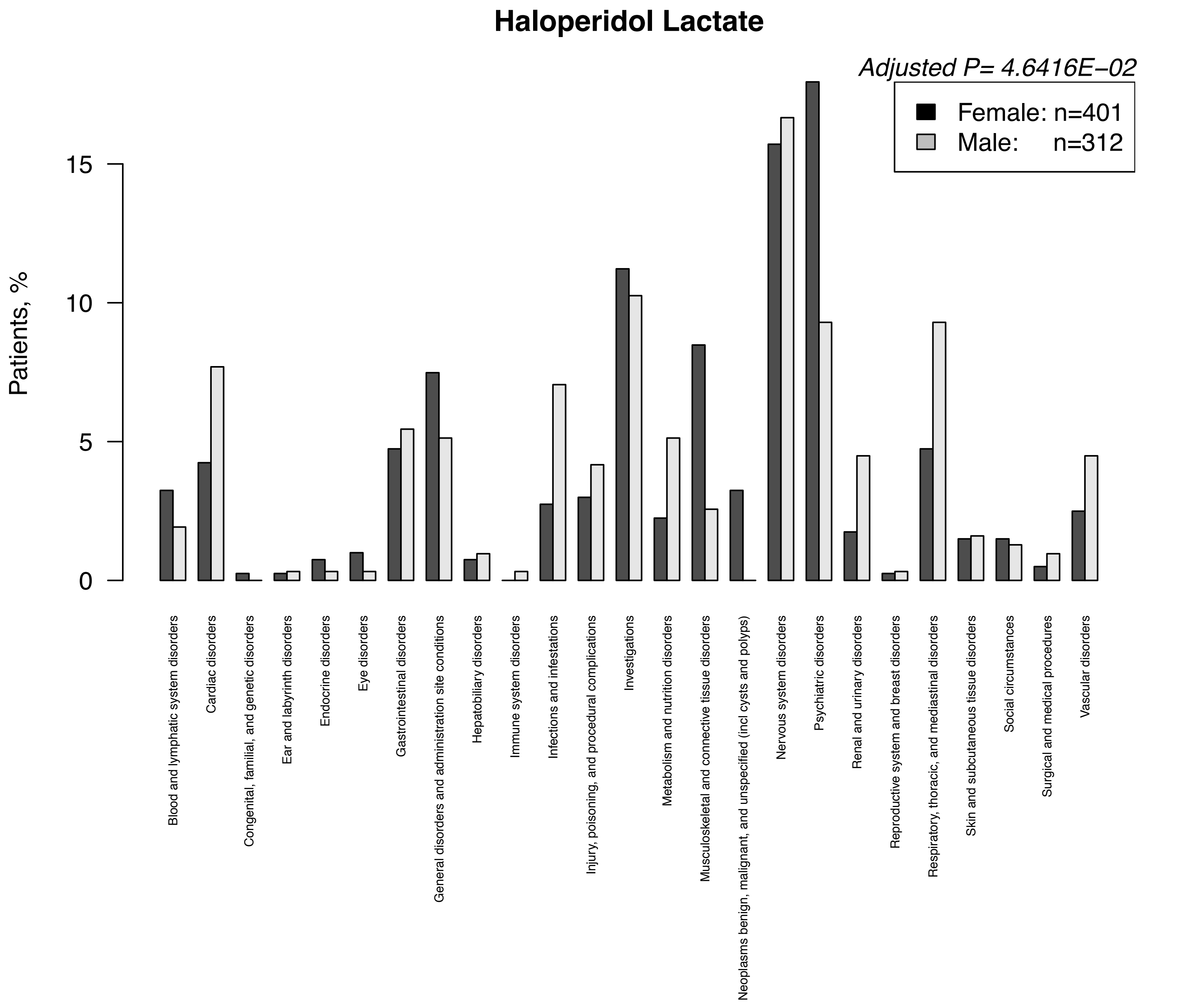
Haloperidol Lactate
Adjusted P= 4.6416E−02
Female: n=401
Male: n=312
Patients, %
0
5
10
15
Blood and lymphatic system disorders
Cardiac disorders
Congenital, familial, and genetic disorders
Ear and labyrinth disorders
Endocrine disorders
Eye disorders
Gastrointestinal disorders
General disorders and administration site conditions
Hepatobiliary disorders
Immune system disorders
Infections and infestations
Injury, poisoning, and procedural complications
Investigations
Metabolism and nutrition disorders
Musculoskeletal and connective tissue disorders
Neoplasms benign, malignant, and unspecified (incl cysts and polyps)
Nervous system disorders
Psychiatric disorders
Renal and urinary disorders
Reproductive system and breast disorders
Respiratory, thoracic, and mediastinal disorders
Skin and subcutaneous tissue disorders
Social circumstances
Surgical and medical procedures
Vascular disorders

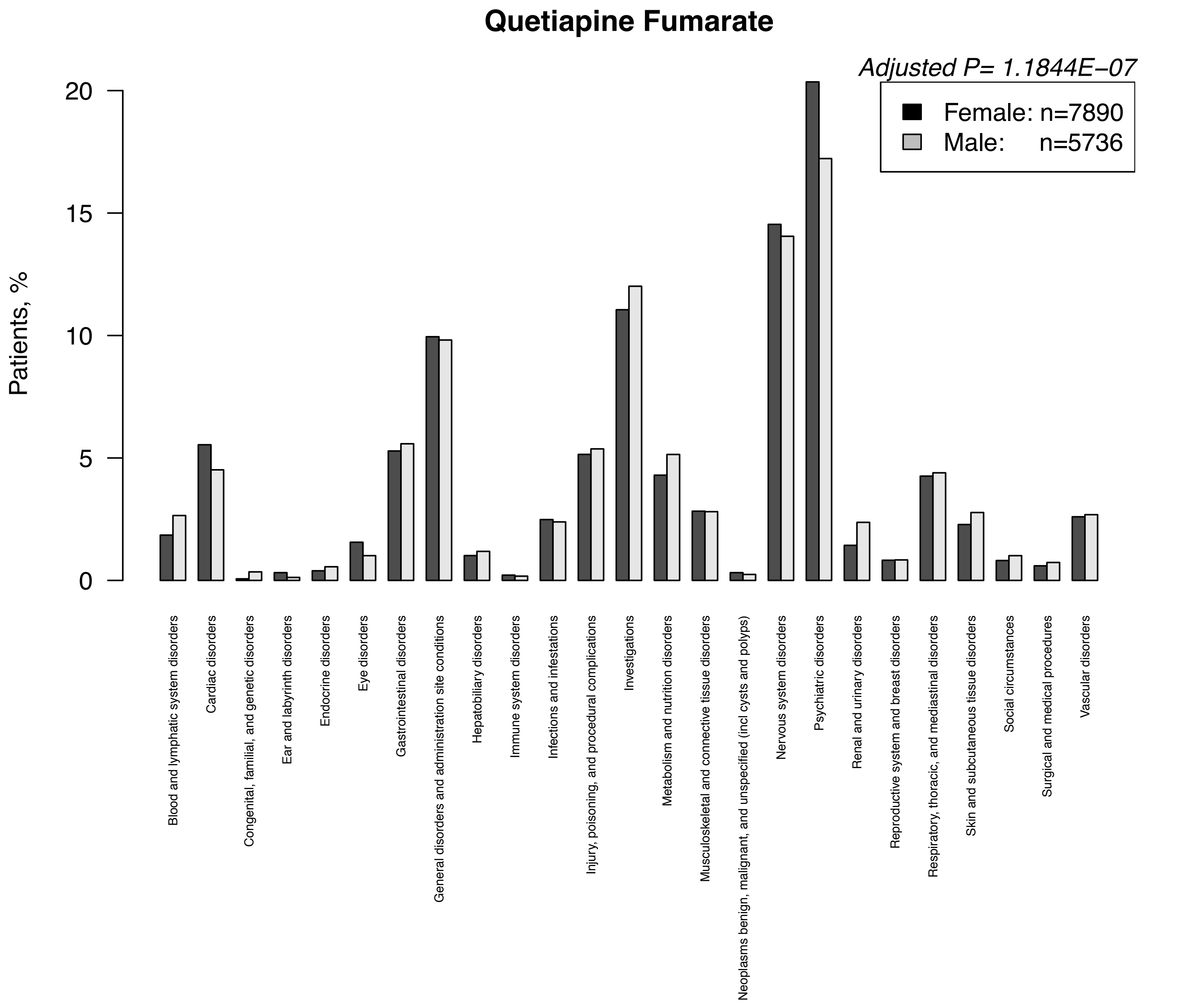
Quetiapine Fumarate
Adjusted P= 1.1844E−07
Female: n=7890
Male: n=5736
Patients, %
0
5
10
15
20
Blood and lymphatic system disorders
Cardiac disorders
Congenital, familial, and genetic disorders
Ear and labyrinth disorders
Endocrine disorders
Eye disorders
Gastrointestinal disorders
General disorders and administration site conditions
Hepatobiliary disorders
Immune system disorders
Infections and infestations
Injury, poisoning, and procedural complications
Investigations
Metabolism and nutrition disorders
Musculoskeletal and connective tissue disorders
Neoplasms benign, malignant, and unspecified (incl cysts and polyps)
Nervous system disorders
Psychiatric disorders
Renal and urinary disorders
Reproductive system and breast disorders
Respiratory, thoracic, and mediastinal disorders
Skin and subcutaneous tissue disorders
Social circumstances
Surgical and medical procedures
Vascular disorders

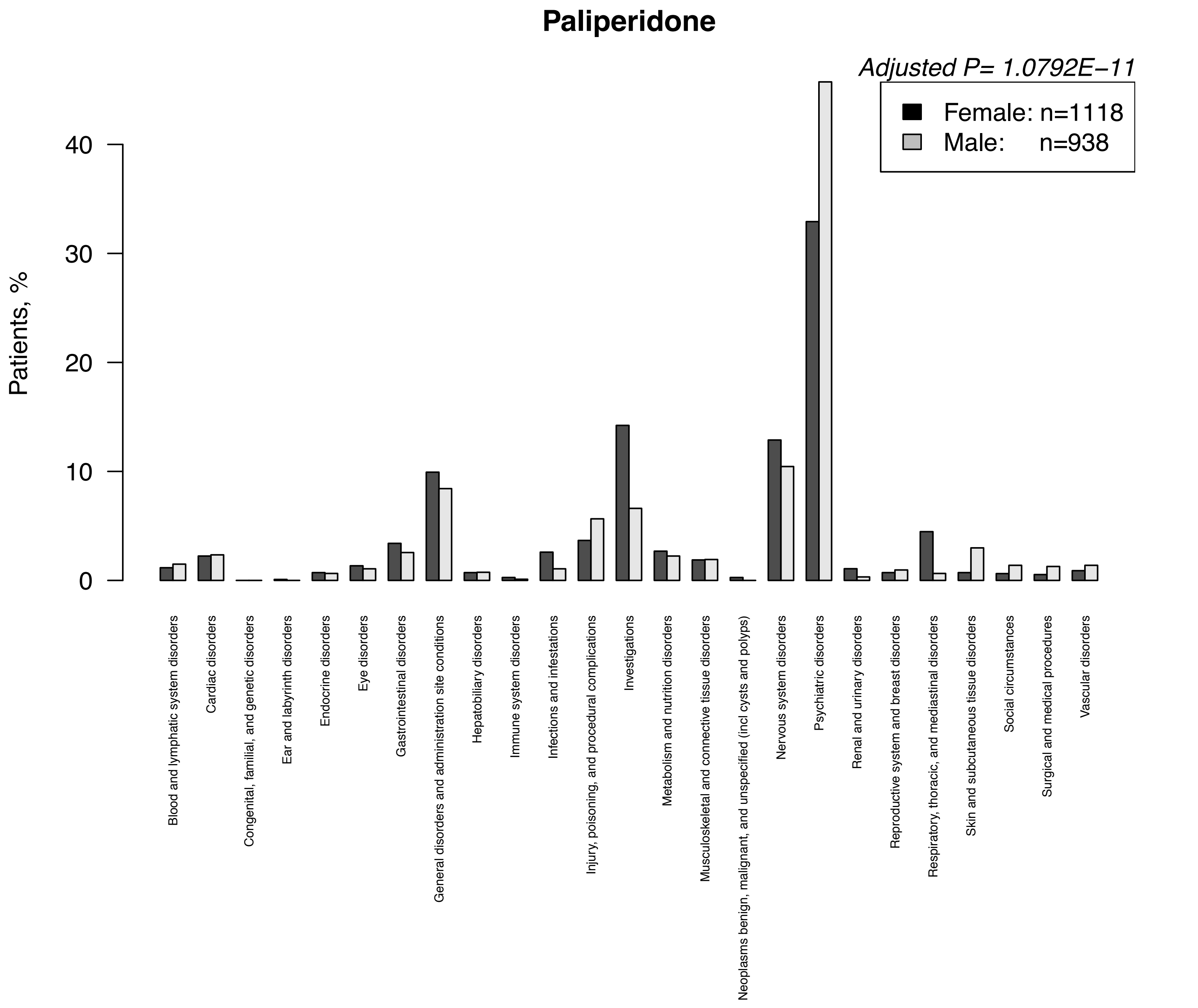
Paliperidone
Adjusted P= 1.0792E−11
Female: n=1118
Male: n=938
Patients, %
0
10
20
30
40
Blood and lymphatic system disorders
Cardiac disorders
Congenital, familial, and genetic disorders
Ear and labyrinth disorders
Endocrine disorders
Eye disorders
Gastrointestinal disorders
General disorders and administration site conditions
Hepatobiliary disorders
Immune system disorders
Infections and infestations
Injury, poisoning, and procedural complications
Investigations
Metabolism and nutrition disorders
Musculoskeletal and connective tissue disorders
Neoplasms benign, malignant, and unspecified (incl cysts and polyps)
Nervous system disorders
Psychiatric disorders
Renal and urinary disorders
Reproductive system and breast disorders
Respiratory, thoracic, and mediastinal disorders
Skin and subcutaneous tissue disorders
Social circumstances
Surgical and medical procedures
Vascular disorders

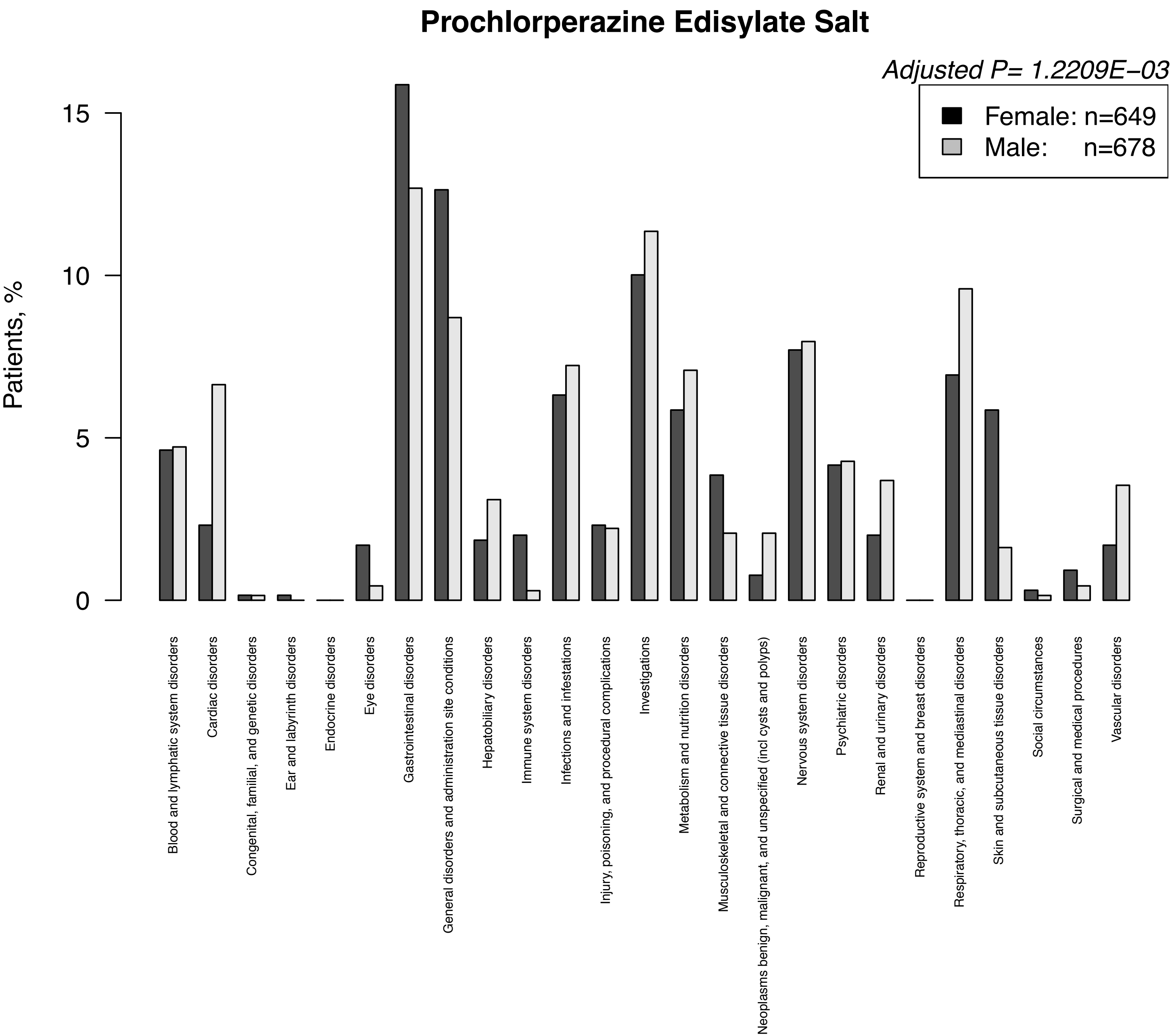

Prochlorperazine Edisylate Salt
Adjusted P= 1.2209E−03
Female: n=649
Male: n=678
Patients, %
0
5
10
15
Blood and lymphatic system disorders
Cardiac disorders
Congenital, familial, and genetic disorders
Ear and labyrinth disorders
Endocrine disorders
Eye disorders
Gastrointestinal disorders
General disorders and administration site conditions
Hepatobiliary disorders
Immune system disorders
Infections and infestations
Injury, poisoning, and procedural complications
Investigations
Metabolism and nutrition disorders
Musculoskeletal and connective tissue disorders
Neoplasms benign, malignant, and unspecified (incl cysts and polyps)
Nervous system disorders
Psychiatric disorders
Renal and urinary disorders
Reproductive system and breast disorders
Respiratory, thoracic, and mediastinal disorders
Skin and subcutaneous tissue disorders
Social circumstances
Surgical and medical procedures
Vascular disorders

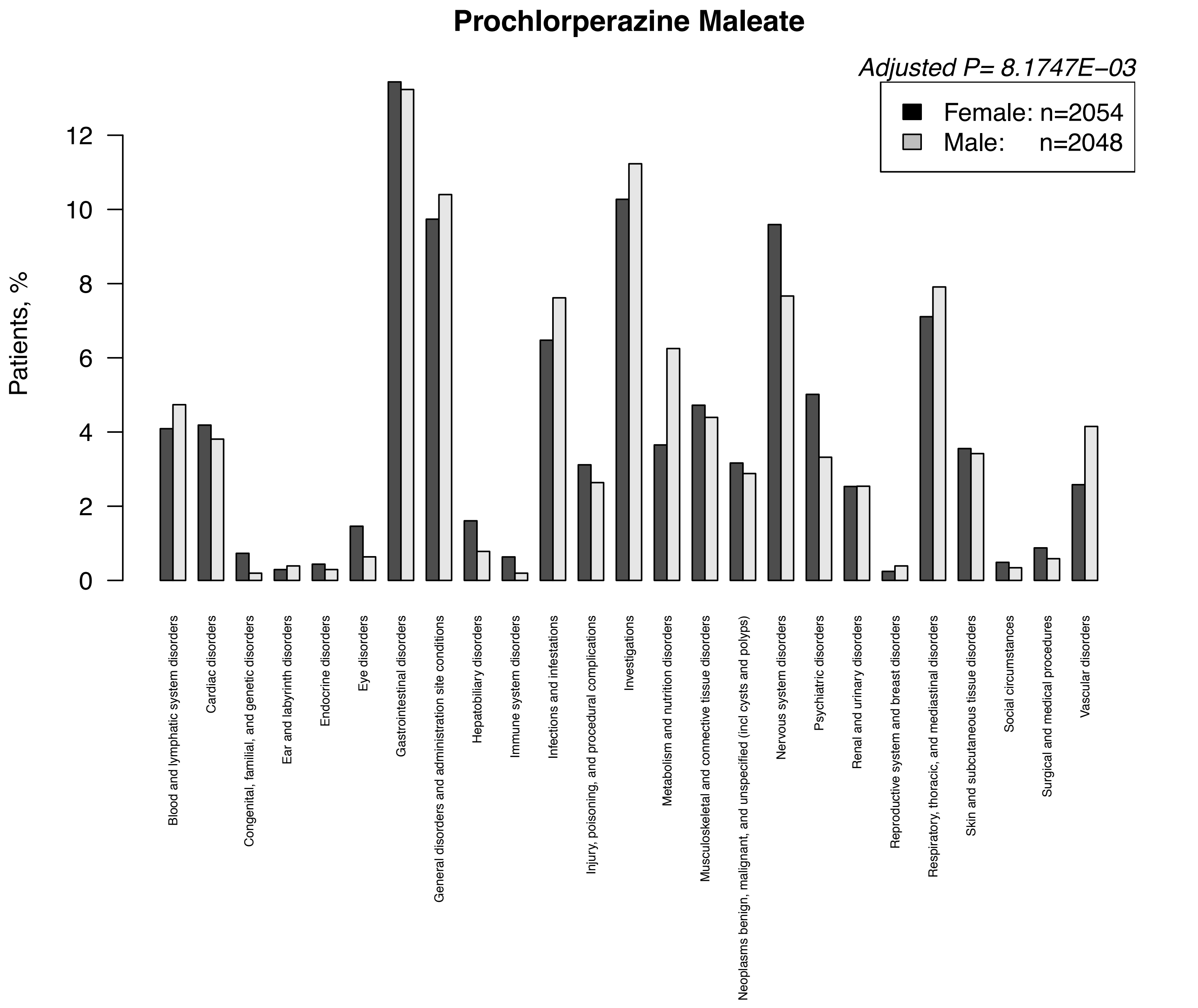
Prochlorperazine Maleate
Adjusted P= 8.1747E−03
Female: n=2054
Male: n=2048
Patients, %
0
2
4
6
8
10
12
Blood and lymphatic system disorders
Cardiac disorders
Congenital, familial, and genetic disorders
Ear and labyrinth disorders
Endocrine disorders
Eye disorders
Gastrointestinal disorders
General disorders and administration site conditions
Hepatobiliary disorders
Immune system disorders
Infections and infestations
Injury, poisoning, and procedural complications
Investigations
Metabolism and nutrition disorders
Musculoskeletal and connective tissue disorders
Neoplasms benign, malignant, and unspecified (incl cysts and polyps)
Nervous system disorders
Psychiatric disorders
Renal and urinary disorders
Reproductive system and breast disorders
Respiratory, thoracic, and mediastinal disorders
Skin and subcutaneous tissue disorders
Social circumstances
Surgical and medical procedures
Vascular disorders

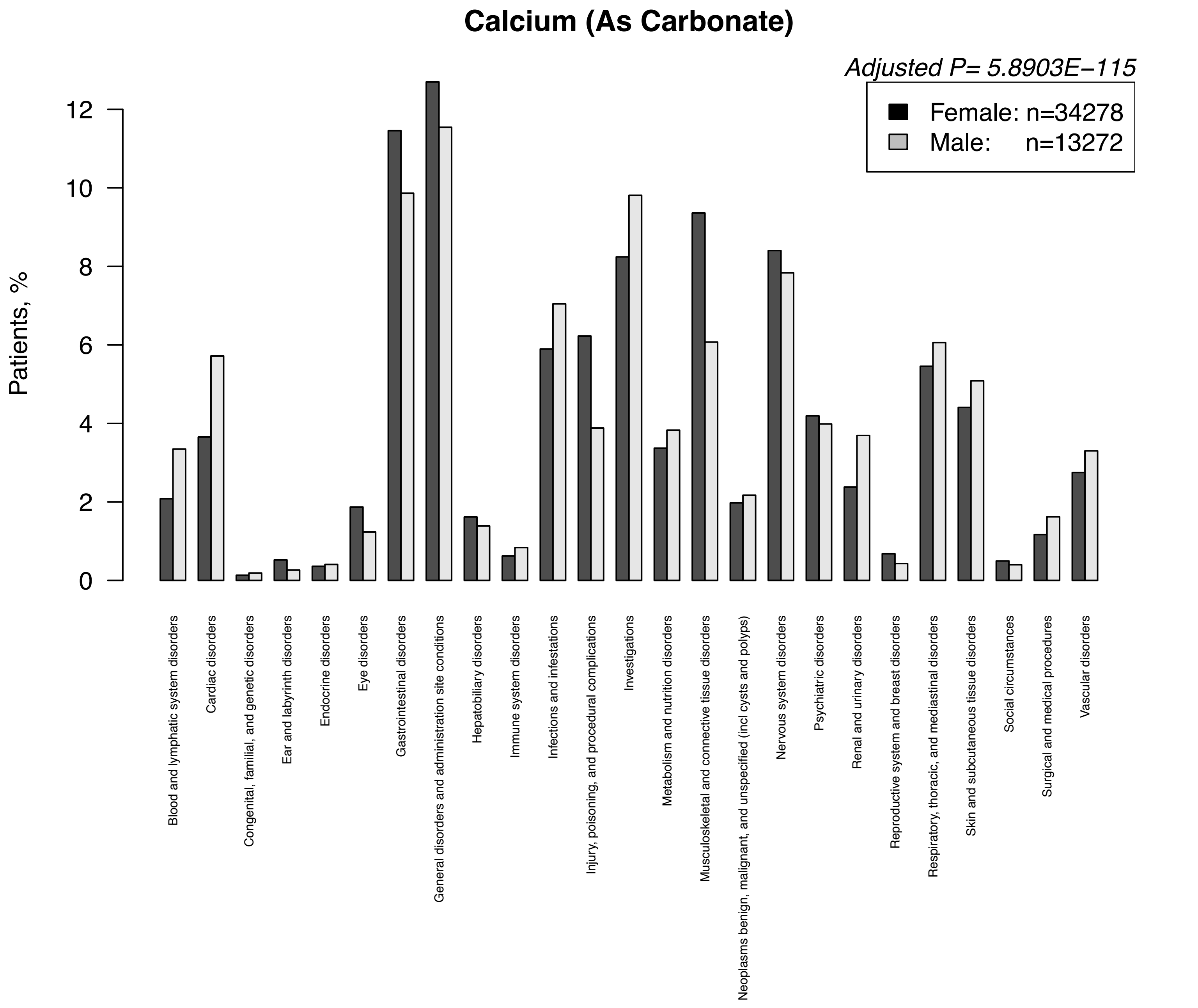

Calcium (As Carbonate)
Adjusted P= 5.8903E−115
Female: n=34278
Male: n=13272
Patients, %
0
2
4
6
8
10
12
Blood and lymphatic system disorders
Cardiac disorders
Congenital, familial, and genetic disorders
Ear and labyrinth disorders
Endocrine disorders
Eye disorders
Gastrointestinal disorders
General disorders and administration site conditions
Hepatobiliary disorders
Immune system disorders
Infections and infestations
Injury, poisoning, and procedural complications
Investigations
Metabolism and nutrition disorders
Musculoskeletal and connective tissue disorders
Neoplasms benign, malignant, and unspecified (incl cysts and polyps)
Nervous system disorders
Psychiatric disorders
Renal and urinary disorders
Reproductive system and breast disorders
Respiratory, thoracic, and mediastinal disorders
Skin and subcutaneous tissue disorders
Social circumstances
Surgical and medical procedures
Vascular disorders

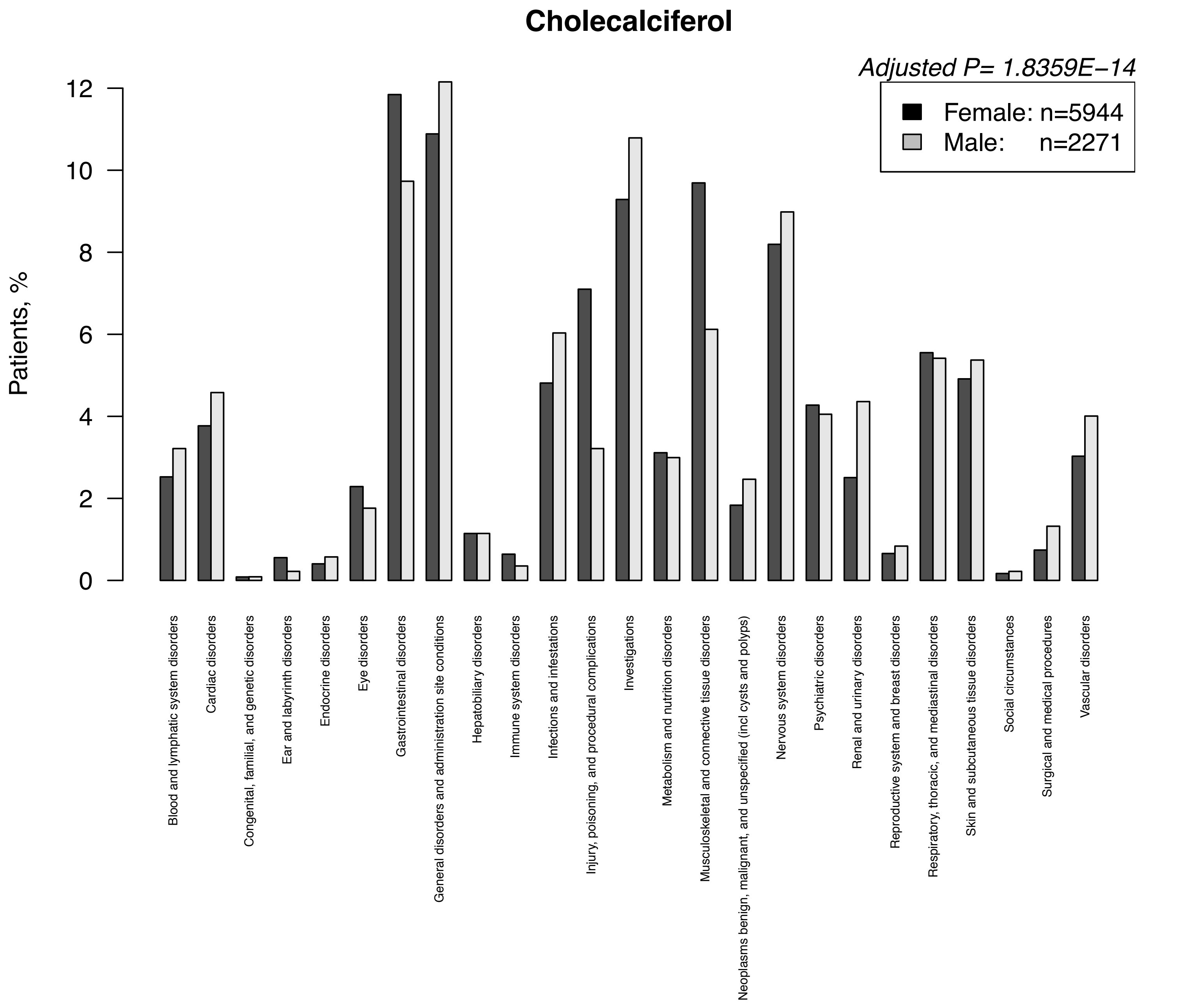

Cholecalciferol
Adjusted P= 1.8359E−14
Female: n=5944
Male: n=2271
Patients, %
0
2
4
6
8
10
12
Blood and lymphatic system disorders
Cardiac disorders
Congenital, familial, and genetic disorders
Ear and labyrinth disorders
Endocrine disorders
Eye disorders
Gastrointestinal disorders
General disorders and administration site conditions
Hepatobiliary disorders
Immune system disorders
Infections and infestations
Injury, poisoning, and procedural complications
Investigations
Metabolism and nutrition disorders
Musculoskeletal and connective tissue disorders
Neoplasms benign, malignant, and unspecified (incl cysts and polyps)
Nervous system disorders
Psychiatric disorders
Renal and urinary disorders
Reproductive system and breast disorders
Respiratory, thoracic, and mediastinal disorders
Skin and subcutaneous tissue disorders
Social circumstances
Surgical and medical procedures
Vascular disorders

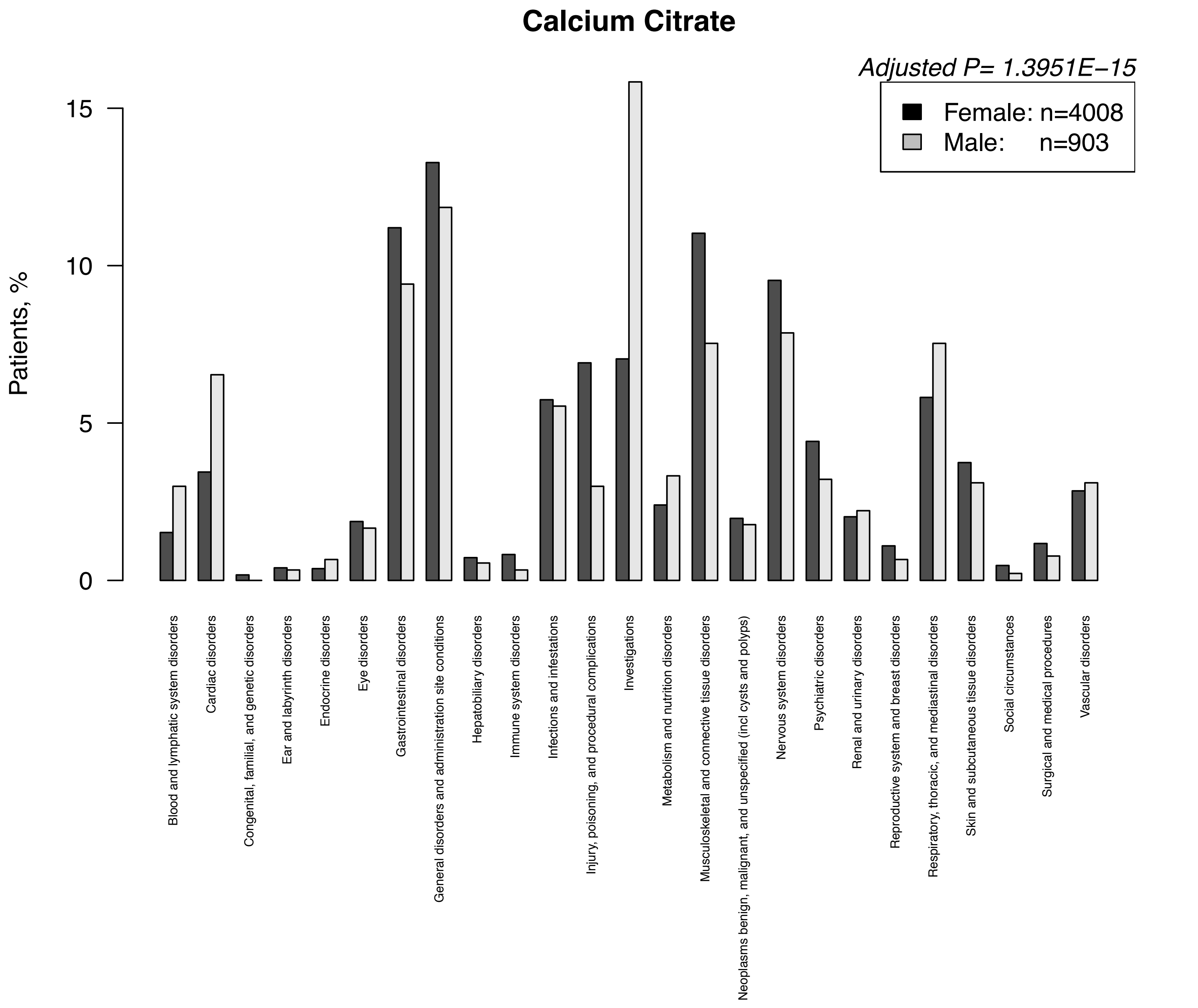
Calcium Citrate
Adjusted P= 1.3951E−15
Female: n=4008
Male: n=903
Patients, %
15
10
5
0
Blood and lymphatic system disorders
Cardiac disorders
Congenital, familial, and genetic disorders
Ear and labyrinth disorders
Endocrine disorders
Eye disorders
Gastrointestinal disorders
General disorders and administration site conditions
Hepatobiliary disorders
Immune system disorders
Infections and infestations
Injury, poisoning, and procedural complications
Investigations
Metabolism and nutrition disorders
Musculoskeletal and connective tissue disorders
Neoplasms benign, malignant, and unspecified (incl cysts and polyps)
Nervous system disorders
Psychiatric disorders
Renal and urinary disorders
Reproductive system and breast disorders
Respiratory, thoracic, and mediastinal disorders
Skin and subcutaneous tissue disorders
Social circumstances
Surgical and medical procedures
Vascular disorders

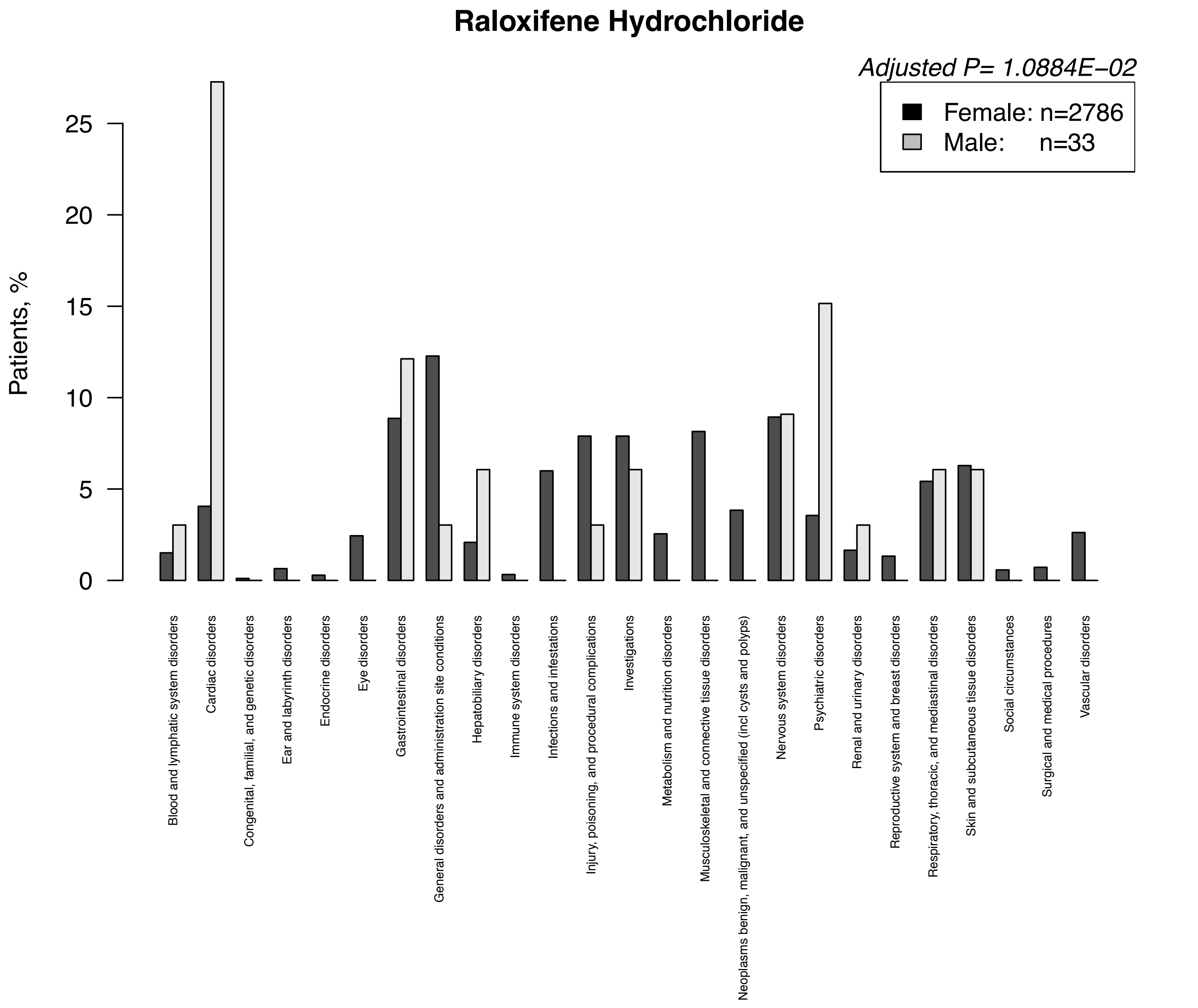

Raloxifene Hydrochloride
Adjusted P= 1.0884E−02
Female: n=2786
Male: n=33
Patients, %
0
5
10
15
20
25
Blood and lymphatic system disorders
Cardiac disorders
Congenital, familial, and genetic disorders
Ear and labyrinth disorders
Endocrine disorders
Eye disorders
Gastrointestinal disorders
General disorders and administration site conditions
Hepatobiliary disorders
Immune system disorders
Infections and infestations
Injury, poisoning, and procedural complications
Investigations
Metabolism and nutrition disorders
Musculoskeletal and connective tissue disorders
Neoplasms benign, malignant, and unspecified (incl cysts and polyps)
Nervous system disorders
Psychiatric disorders
Renal and urinary disorders
Reproductive system and breast disorders
Respiratory, thoracic, and mediastinal disorders
Skin and subcutaneous tissue disorders
Social circumstances
Surgical and medical procedures
Vascular disorders

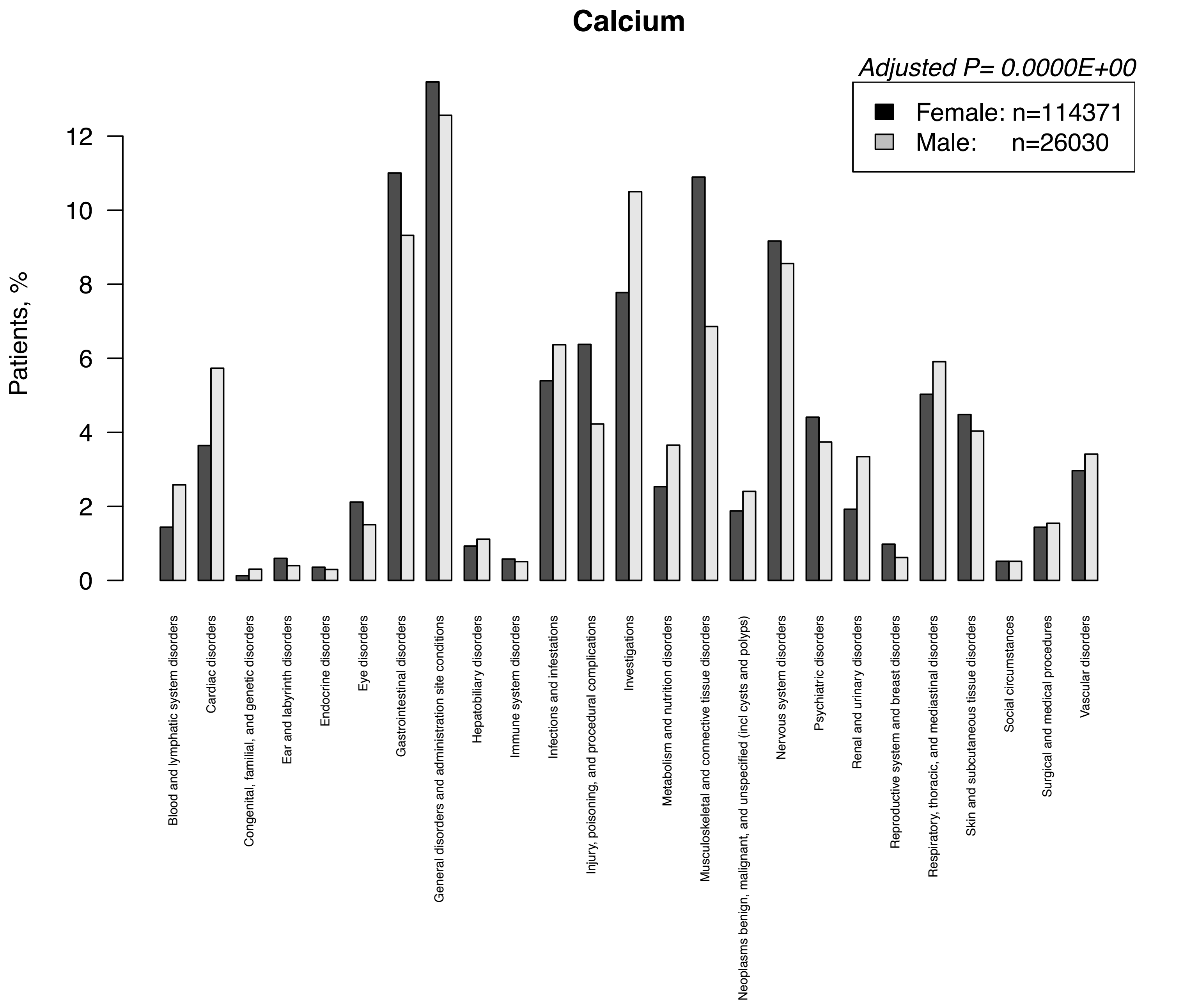

Calcium
Adjusted P= 0.0000E+00
Female: n=114371
Male: n=26030
Patients, %
0
2
4
6
8
10
12
Blood and lymphatic system disorders
Cardiac disorders
Congenital, familial, and genetic disorders
Ear and labyrinth disorders
Endocrine disorders
Eye disorders
Gastrointestinal disorders
General disorders and administration site conditions
Hepatobiliary disorders
Immune system disorders
Infections and infestations
Injury, poisoning, and procedural complications
Investigations
Metabolism and nutrition disorders
Musculoskeletal and connective tissue disorders
Neoplasms benign, malignant, and unspecified (incl cysts and polyps)
Nervous system disorders
Psychiatric disorders
Renal and urinary disorders
Reproductive system and breast disorders
Respiratory, thoracic, and mediastinal disorders
Skin and subcutaneous tissue disorders
Social circumstances
Surgical and medical procedures
Vascular disorders

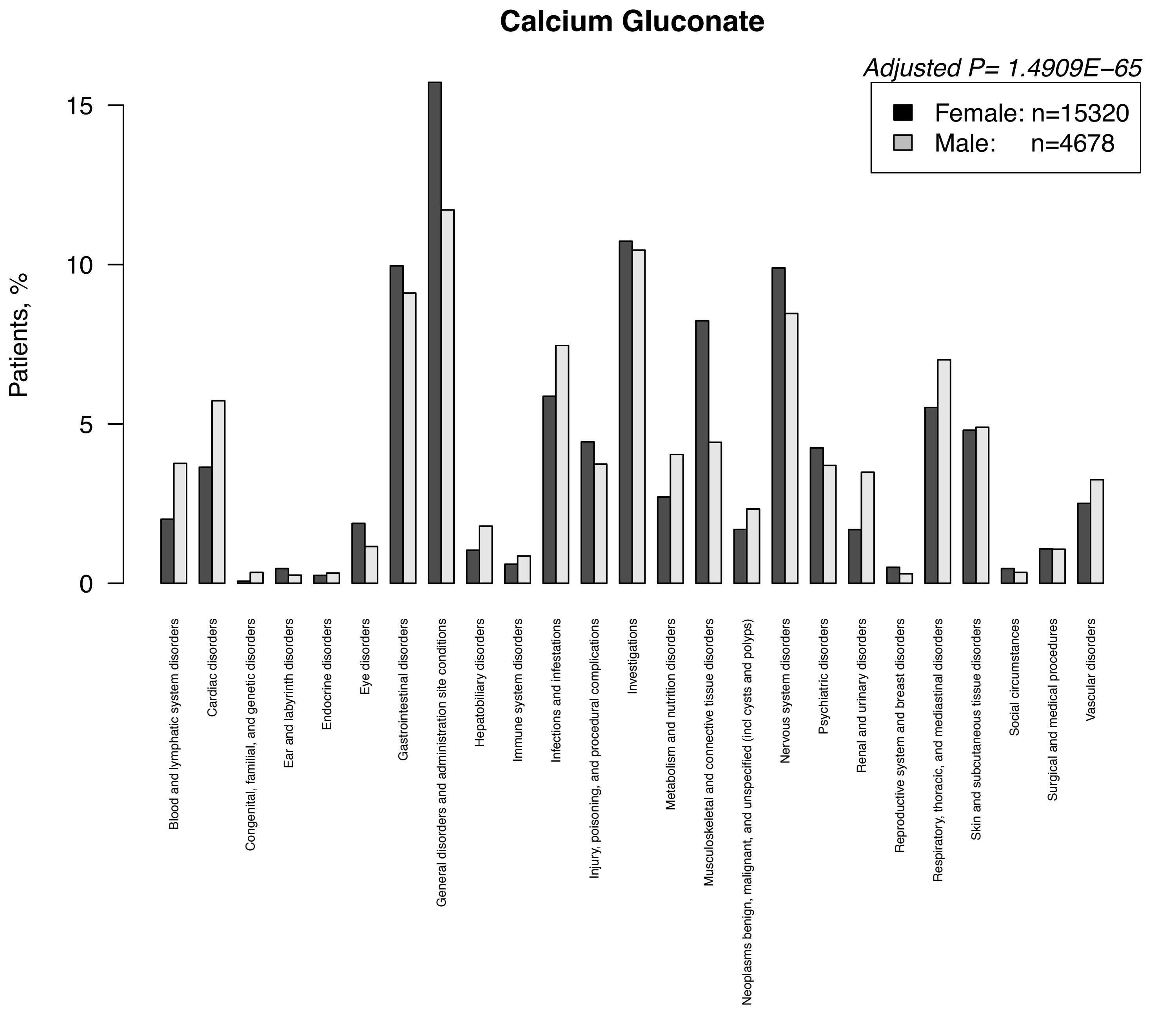

Calcium Gluconate
Adjusted P= 1.4909E−65
Female: n=15320
Male: n=4678
Patients, %
0
5
10
15
Blood and lymphatic system disorders
Cardiac disorders
Congenital, familial, and genetic disorders
Ear and labyrinth disorders
Endocrine disorders
Eye disorders
Gastrointestinal disorders
General disorders and administration site conditions
Hepatobiliary disorders
Immune system disorders
Infections and infestations
Injury, poisoning, and procedural complications
Investigations
Metabolism and nutrition disorders
Musculoskeletal and connective tissue disorders
Neoplasms benign, malignant, and unspecified (incl cysts and polyps)
Nervous system disorders
Psychiatric disorders
Renal and urinary disorders
Reproductive system and breast disorders
Respiratory, thoracic, and mediastinal disorders
Skin and subcutaneous tissue disorders
Social circumstances
Surgical and medical procedures
Vascular disorders

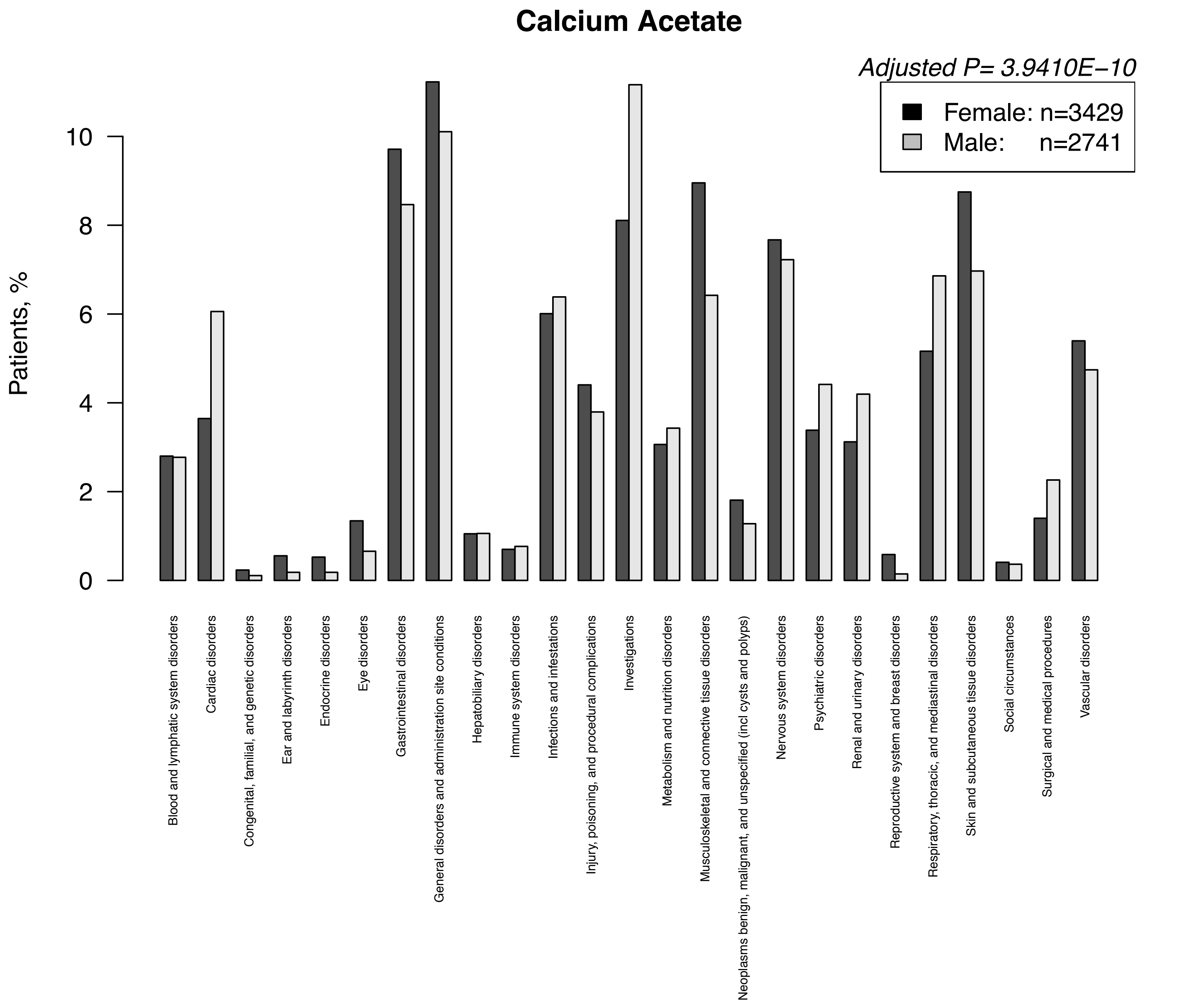

Calcium Acetate
Adjusted P= 3.9410E−10
Female: n=3429
Male: n=2741
Patients, %
0
2
4
6
8
10
Blood and lymphatic system disorders
Cardiac disorders
Congenital, familial, and genetic disorders
Ear and labyrinth disorders
Endocrine disorders
Eye disorders
Gastrointestinal disorders
General disorders and administration site conditions
Hepatobiliary disorders
Immune system disorders
Infections and infestations
Injury, poisoning, and procedural complications
Investigations
Metabolism and nutrition disorders
Musculoskeletal and connective tissue disorders
Neoplasms benign, malignant, and unspecified (incl cysts and polyps)
Nervous system disorders
Psychiatric disorders
Renal and urinary disorders
Reproductive system and breast disorders
Respiratory, thoracic, and mediastinal disorders
Skin and subcutaneous tissue disorders
Social circumstances
Surgical and medical procedures
Vascular disorders

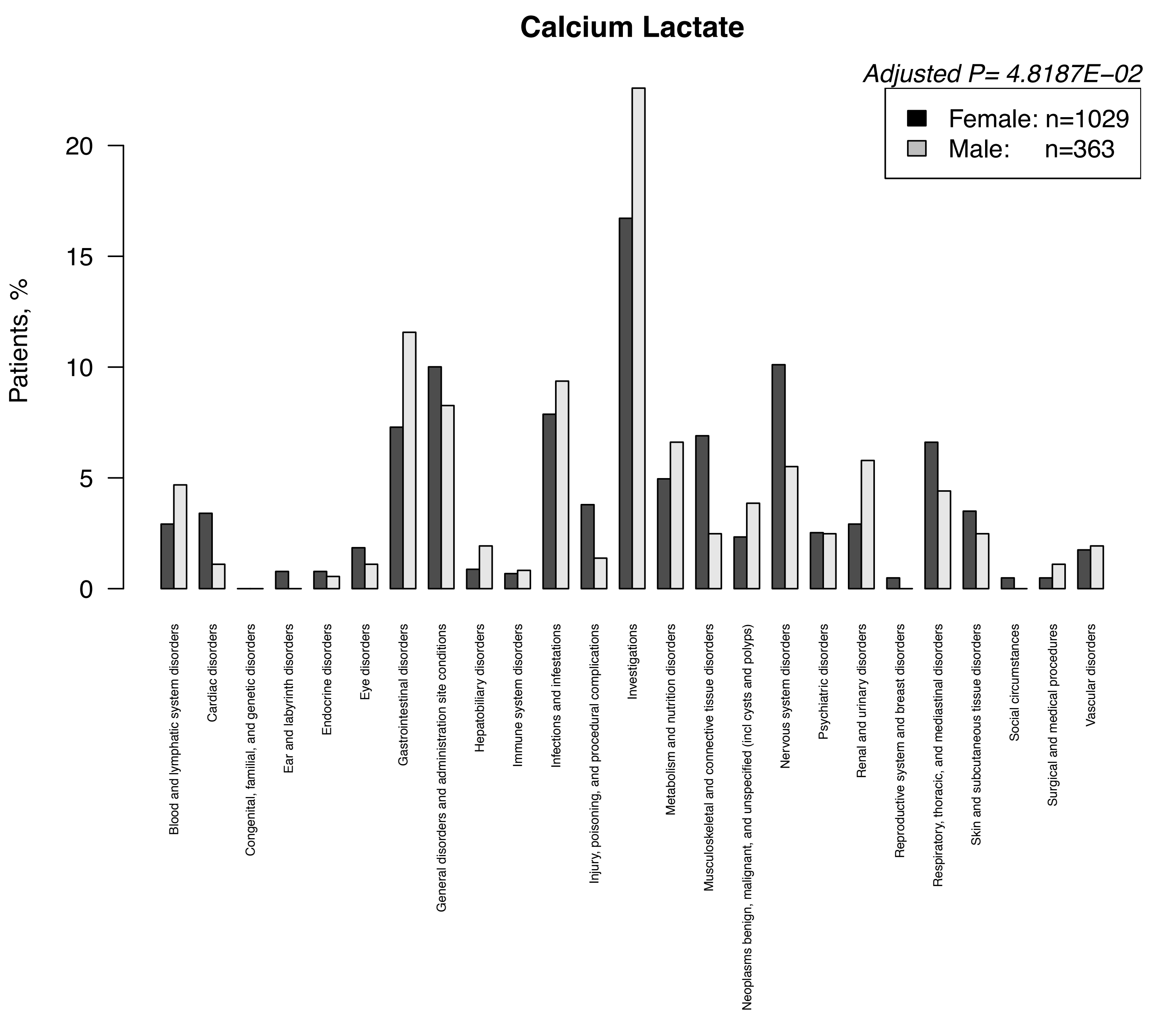
Calcium Lactate
Adjusted P= 4.8187E−02
Female: n=1029
Male: n=363
Patients, %
0
5
10
15
20
Blood and lymphatic system disorders
Cardiac disorders
Congenital, familial, and genetic disorders
Ear and labyrinth disorders
Endocrine disorders
Eye disorders
Gastrointestinal disorders
General disorders and administration site conditions
Hepatobiliary disorders
Immune system disorders
Infections and infestations
Injury, poisoning, and procedural complications
Investigations
Metabolism and nutrition disorders
Musculoskeletal and connective tissue disorders
Neoplasms benign, malignant, and unspecified (incl cysts and polyps)
Nervous system disorders
Psychiatric disorders
Renal and urinary disorders
Reproductive system and breast disorders
Respiratory, thoracic, and mediastinal disorders
Skin and subcutaneous tissue disorders
Social circumstances
Surgical and medical procedures
Vascular disorders

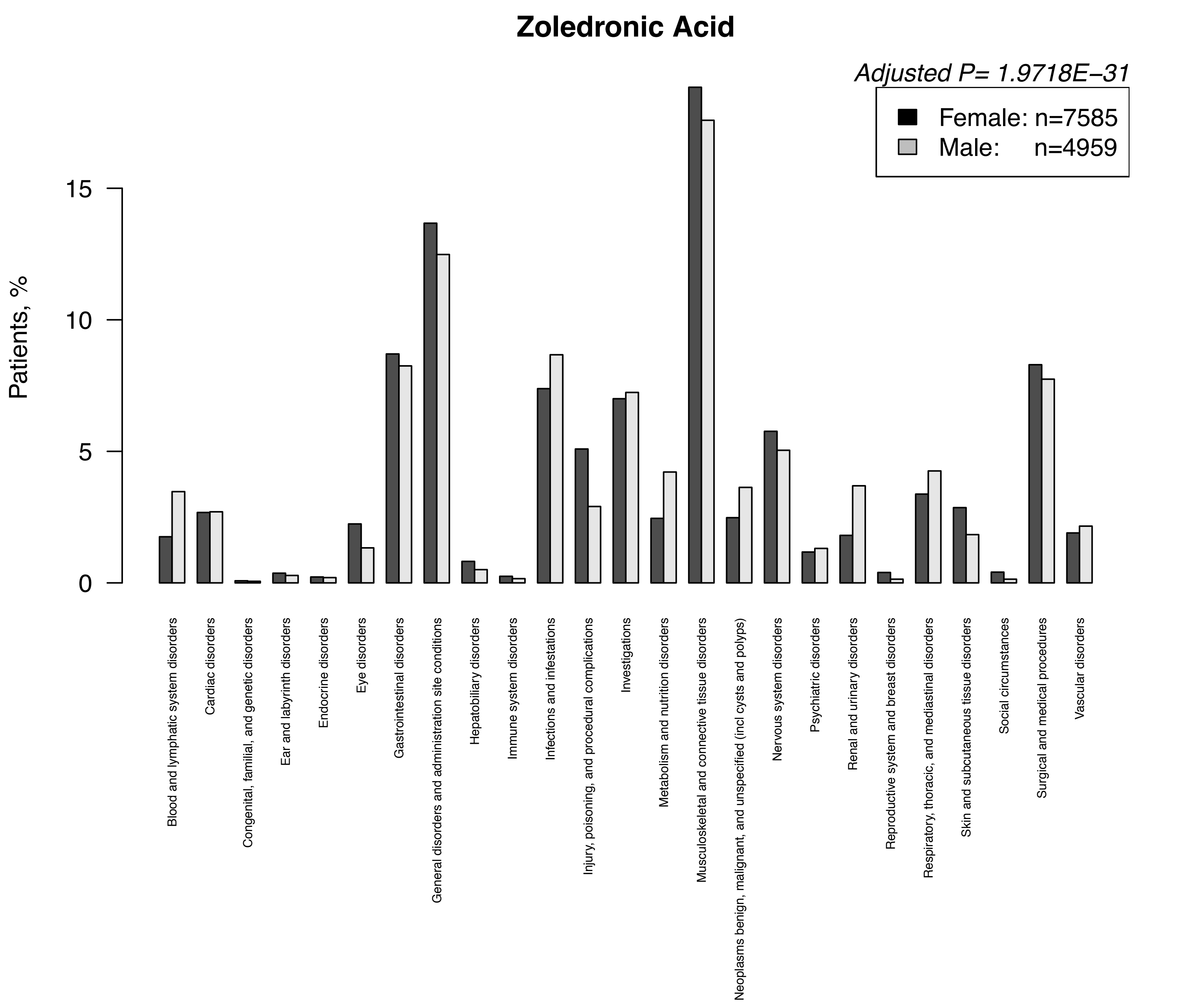
Zoledronic Acid
Adjusted P= 1.9718E−31
Female: n=7585
Male: n=4959
Patients, %
0
5
10
15
Blood and lymphatic system disorders
Cardiac disorders
Congenital, familial, and genetic disorders
Ear and labyrinth disorders
Endocrine disorders
Eye disorders
Gastrointestinal disorders
General disorders and administration site conditions
Hepatobiliary disorders
Immune system disorders
Infections and infestations
Injury, poisoning, and procedural complications
Investigations
Metabolism and nutrition disorders
Musculoskeletal and connective tissue disorders
Neoplasms benign, malignant, and unspecified (incl cysts and polyps)
Nervous system disorders
Psychiatric disorders
Renal and urinary disorders
Reproductive system and breast disorders
Respiratory, thoracic, and mediastinal disorders
Skin and subcutaneous tissue disorders
Social circumstances
Surgical and medical procedures
Vascular disorders

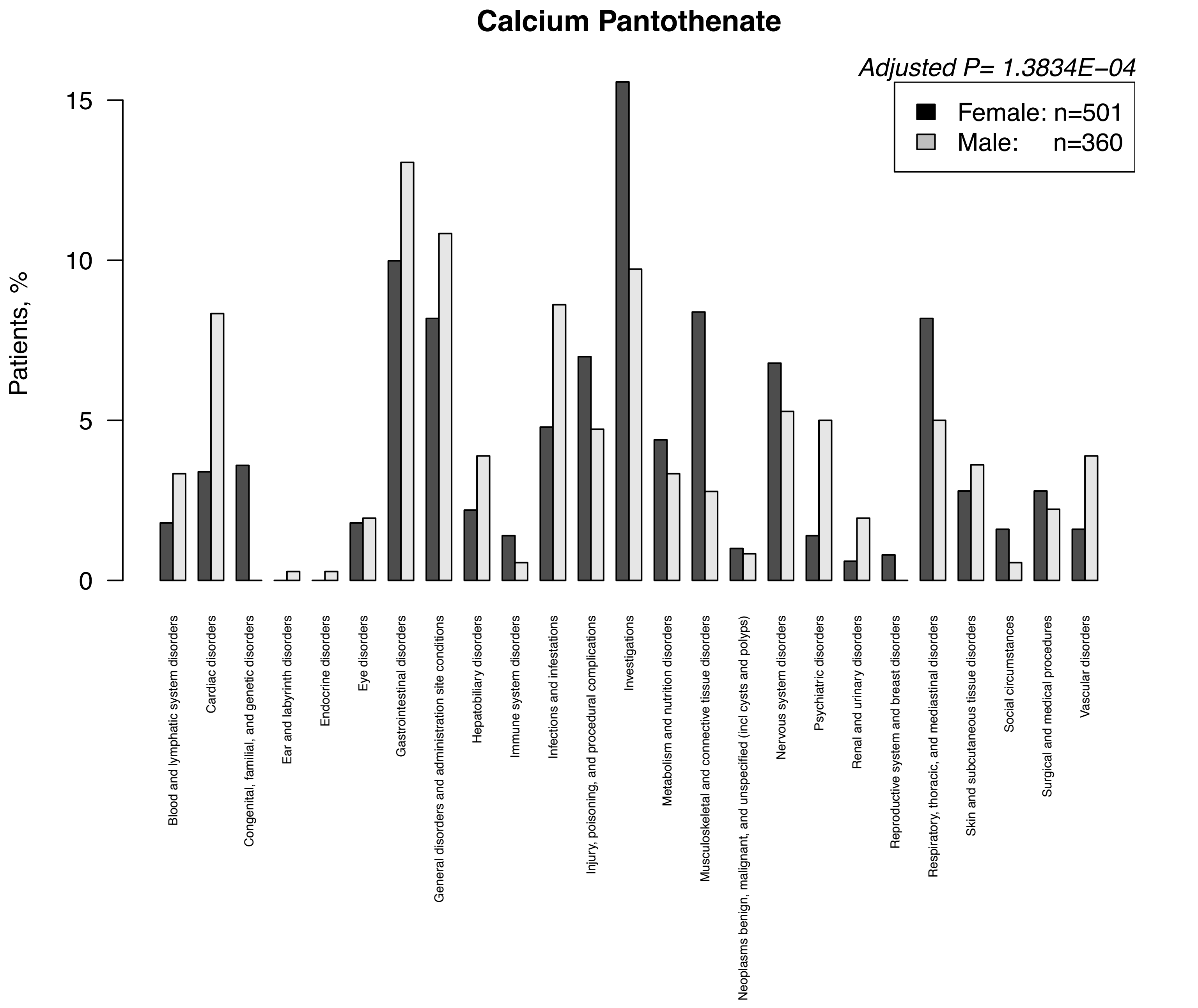
Calcium Pantothenate
Adjusted P= 1.3834E−04
Female: n=501
Male: n=360
Patients, %
0
5
10
15
Blood and lymphatic system disorders
Cardiac disorders
Congenital, familial, and genetic disorders
Ear and labyrinth disorders
Endocrine disorders
Eye disorders
Gastrointestinal disorders
General disorders and administration site conditions
Hepatobiliary disorders
Immune system disorders
Infections and infestations
Injury, poisoning, and procedural complications
Investigations
Metabolism and nutrition disorders
Musculoskeletal and connective tissue disorders
Neoplasms benign, malignant, and unspecified (incl cysts and polyps)
Nervous system disorders
Psychiatric disorders
Renal and urinary disorders
Reproductive system and breast disorders
Respiratory, thoracic, and mediastinal disorders
Skin and subcutaneous tissue disorders
Social circumstances
Surgical and medical procedures
Vascular disorders

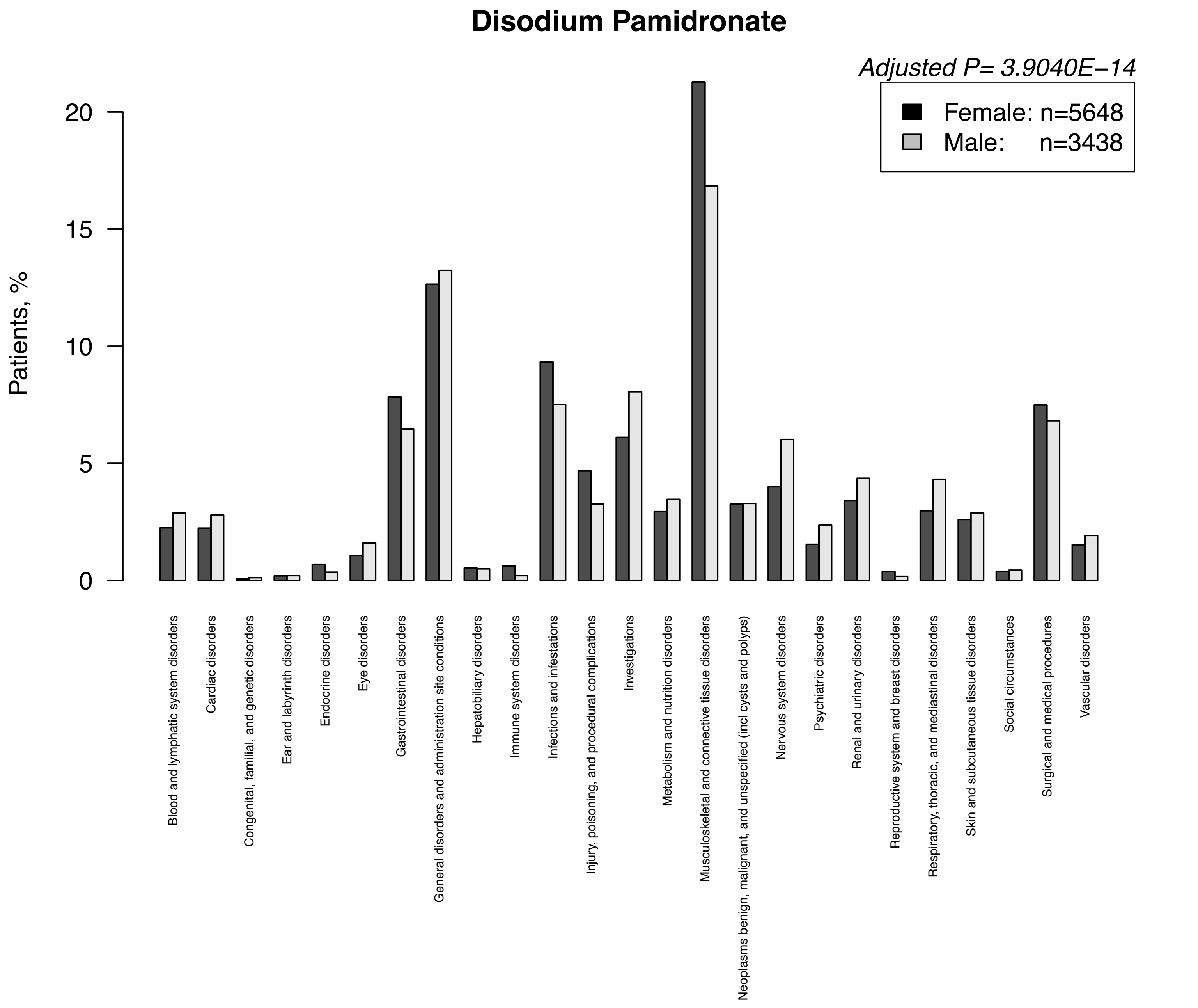
Disodium Pamidronate
Adjusted P= 3.9040E−14
Female: n=5648
Male: n=3438
Patients, %
0
5
10
15
20
Blood and lymphatic system disorders
Cardiac disorders
Congenital, familial, and genetic disorders
Ear and labyrinth disorders
Endocrine disorders
Eye disorders
Gastrointestinal disorders
General disorders and administration site conditions
Hepatobiliary disorders
Immune system disorders
Infections and infestations
Injury, poisoning, and procedural complications
Investigations
Metabolism and nutrition disorders
Musculoskeletal and connective tissue disorders
Neoplasms benign, malignant, and unspecified (incl cysts and polyps)
Nervous system disorders
Psychiatric disorders
Renal and urinary disorders
Reproductive system and breast disorders
Respiratory, thoracic, and mediastinal disorders
Skin and subcutaneous tissue disorders
Social circumstances
Surgical and medical procedures
Vascular disorders

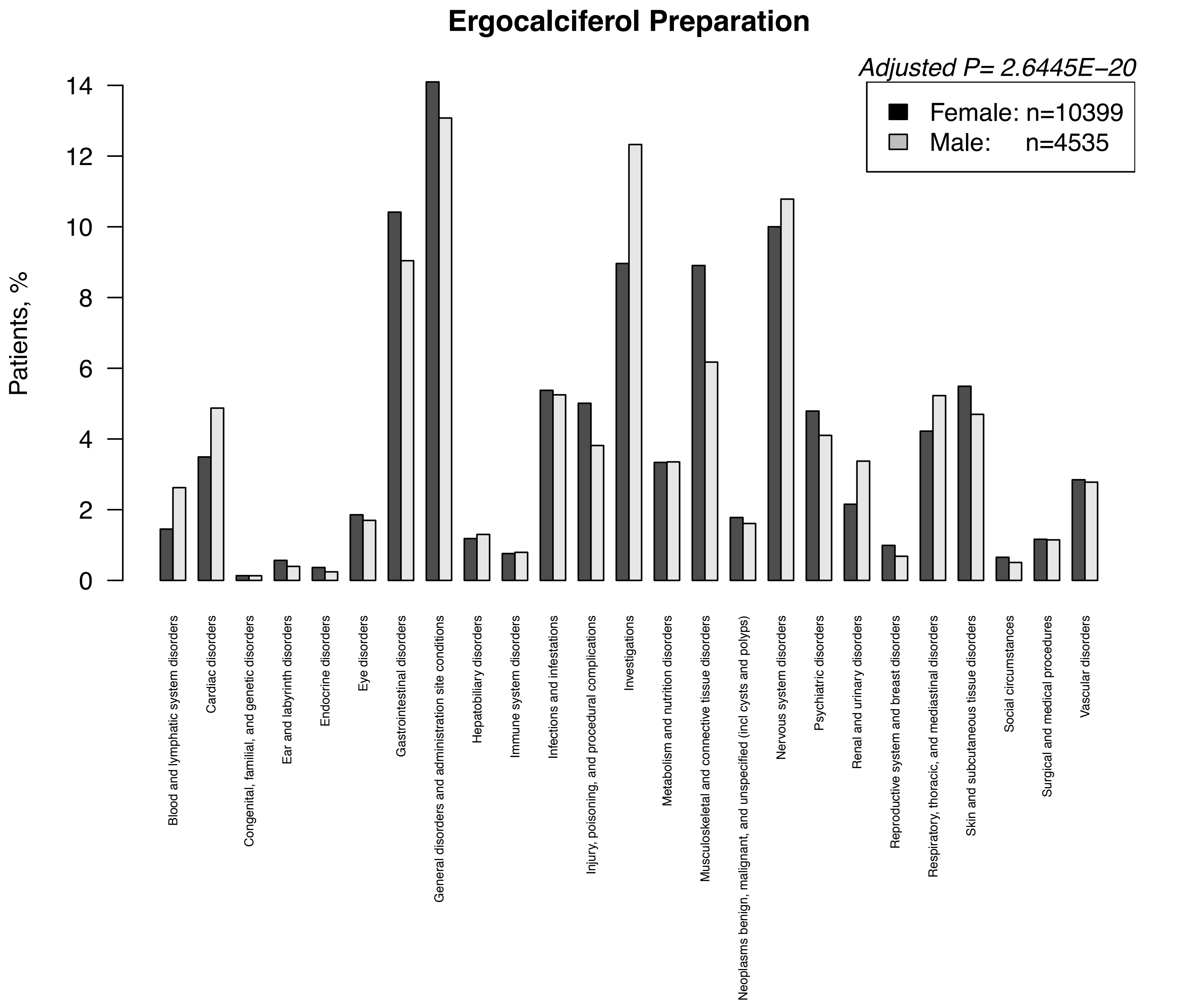

Ergocalciferol Preparation
Adjusted P= 2.6445E−20
Female: n=10399
Male: n=4535
Patients, %
0
2
4
6
8
10
12
14
Blood and lymphatic system disorders
Cardiac disorders
Congenital, familial, and genetic disorders
Ear and labyrinth disorders
Endocrine disorders
Eye disorders
Gastrointestinal disorders
General disorders and administration site conditions
Hepatobiliary disorders
Immune system disorders
Infections and infestations
Injury, poisoning, and procedural complications
Investigations
Metabolism and nutrition disorders
Musculoskeletal and connective tissue disorders
Neoplasms benign, malignant, and unspecified (incl cysts and polyps)
Nervous system disorders
Psychiatric disorders
Renal and urinary disorders
Reproductive system and breast disorders
Respiratory, thoracic, and mediastinal disorders
Skin and subcutaneous tissue disorders
Social circumstances
Surgical and medical procedures
Vascular disorders

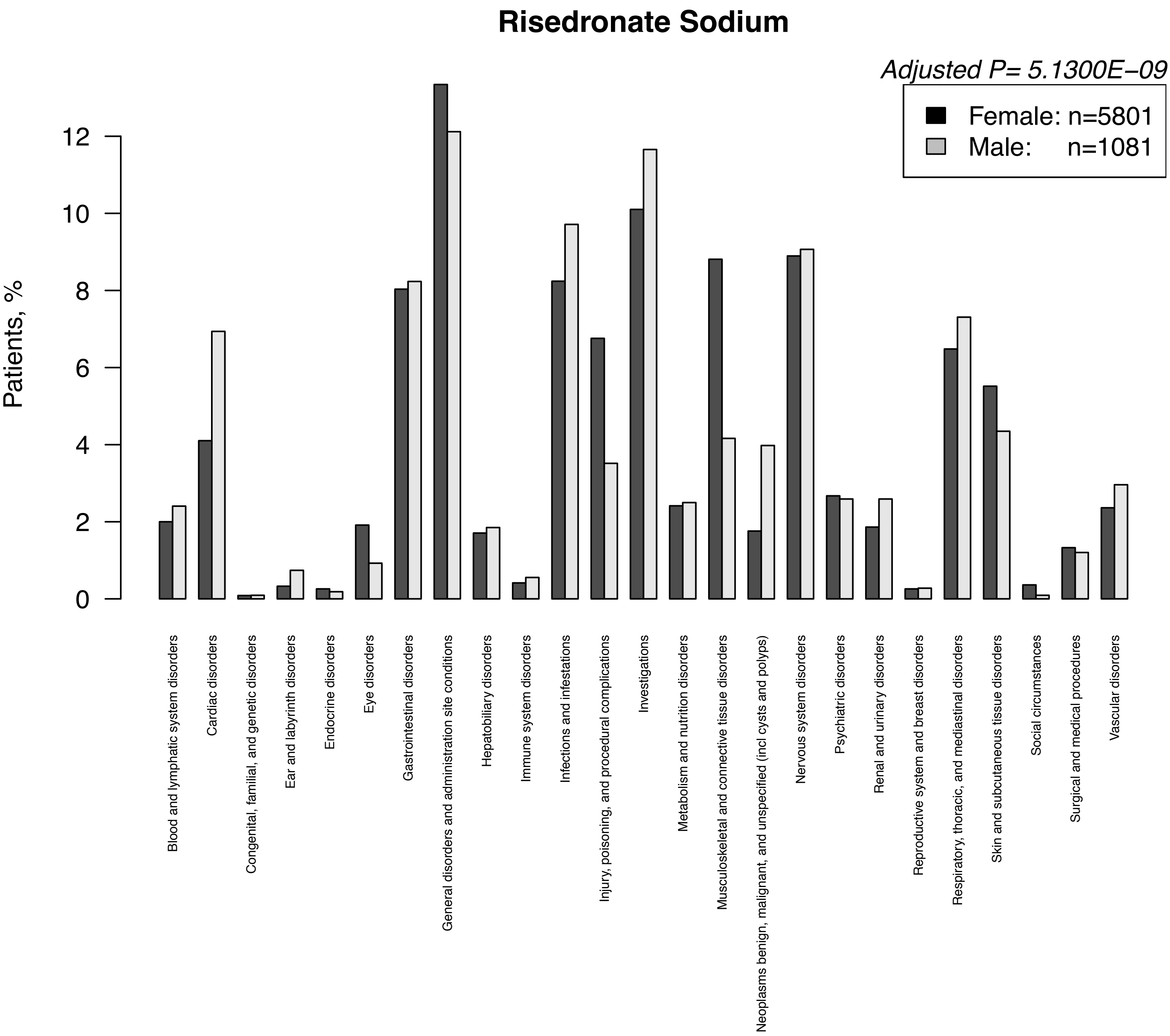

Risedronate Sodium
Adjusted P= 5.1300E−09
Female: n=5801
Male: n=1081
Patients, %
0
2
4
6
8
10
12
Blood and lymphatic system disorders
Cardiac disorders
Congenital, familial, and genetic disorders
Ear and labyrinth disorders
Endocrine disorders
Eye disorders
Gastrointestinal disorders
General disorders and administration site conditions
Hepatobiliary disorders
Immune system disorders
Infections and infestations
Injury, poisoning, and procedural complications
Investigations
Metabolism and nutrition disorders
Musculoskeletal and connective tissue disorders
Neoplasms benign, malignant, and unspecified (incl cysts and polyps)
Nervous system disorders
Psychiatric disorders
Renal and urinary disorders
Reproductive system and breast disorders
Respiratory, thoracic, and mediastinal disorders
Skin and subcutaneous tissue disorders
Social circumstances
Surgical and medical procedures
Vascular disorders

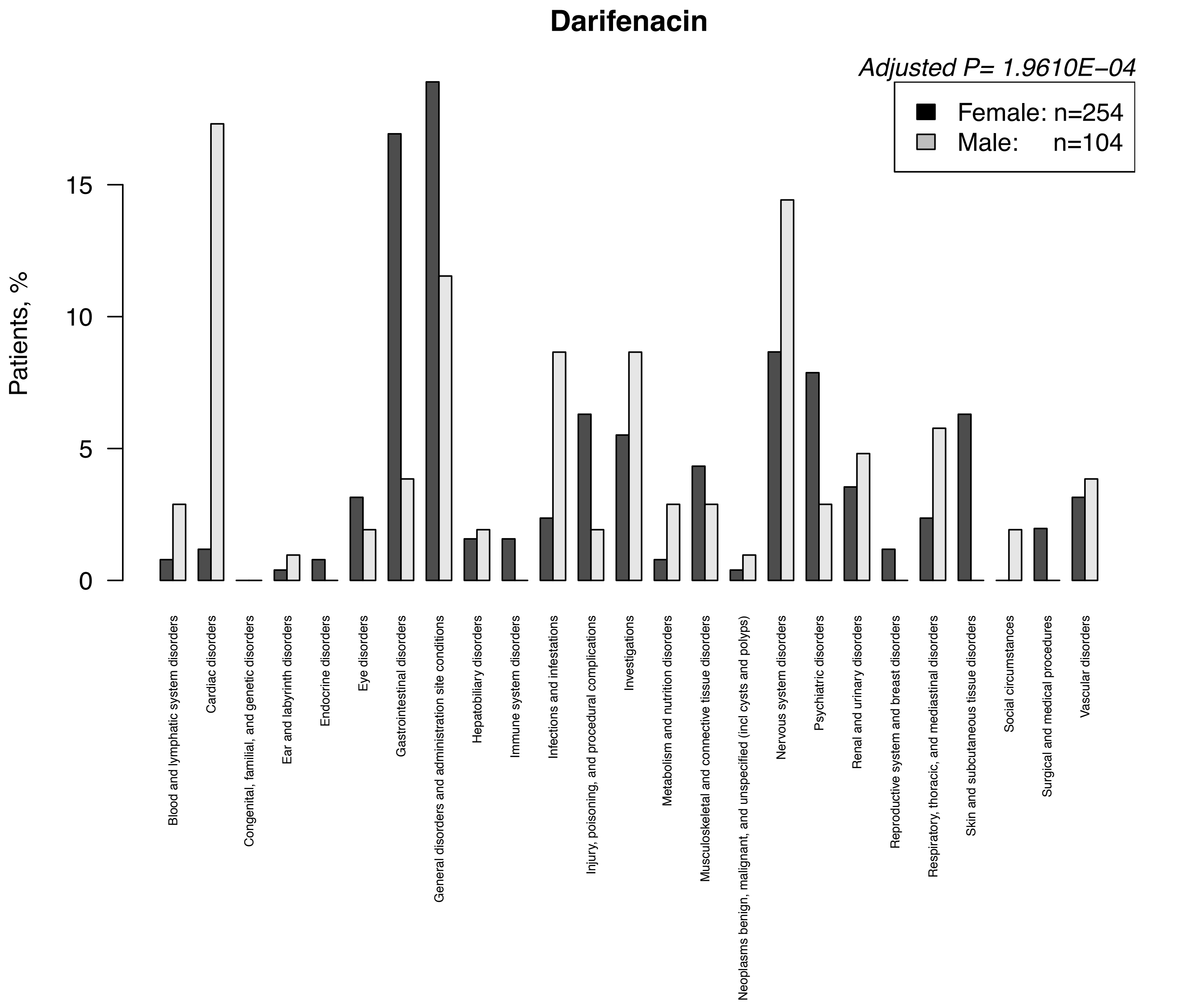
Darifenacin
Adjusted P= 1.9610E−04
Female: n=254
Male: n=104
Patients, %
0
5
10
15
Blood and lymphatic system disorders
Cardiac disorders
Congenital, familial, and genetic disorders
Ear and labyrinth disorders
Endocrine disorders
Eye disorders
Gastrointestinal disorders
General disorders and administration site conditions
Hepatobiliary disorders
Immune system disorders
Infections and infestations
Injury, poisoning, and procedural complications
Investigations
Metabolism and nutrition disorders
Musculoskeletal and connective tissue disorders
Neoplasms benign, malignant, and unspecified (incl cysts and polyps)
Nervous system disorders
Psychiatric disorders
Renal and urinary disorders
Reproductive system and breast disorders
Respiratory, thoracic, and mediastinal disorders
Skin and subcutaneous tissue disorders
Social circumstances
Surgical and medical procedures
Vascular disorders

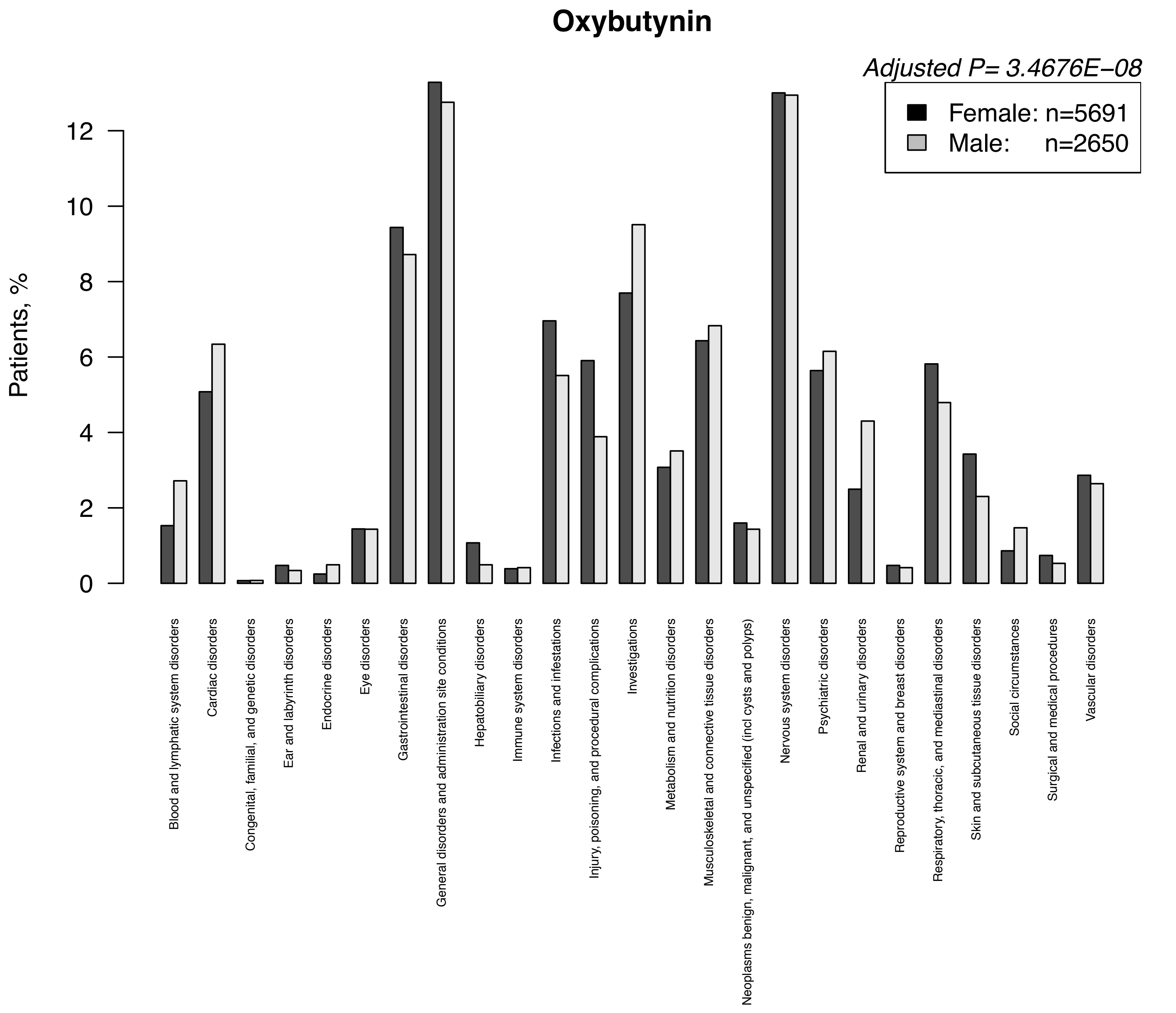

Oxybutynin
Adjusted P= 3.4676E−08
Female: n=5691
Male: n=2650
Patients, %
0
2
4
6
8
10
12
Blood and lymphatic system disorders
Cardiac disorders
Congenital, familial, and genetic disorders
Ear and labyrinth disorders
Endocrine disorders
Eye disorders
Gastrointestinal disorders
General disorders and administration site conditions
Hepatobiliary disorders
Immune system disorders
Infections and infestations
Injury, poisoning, and procedural complications
Investigations
Metabolism and nutrition disorders
Musculoskeletal and connective tissue disorders
Neoplasms benign, malignant, and unspecified (incl cysts and polyps)
Nervous system disorders
Psychiatric disorders
Renal and urinary disorders
Reproductive system and breast disorders
Respiratory, thoracic, and mediastinal disorders
Skin and subcutaneous tissue disorders
Social circumstances
Surgical and medical procedures
Vascular disorders

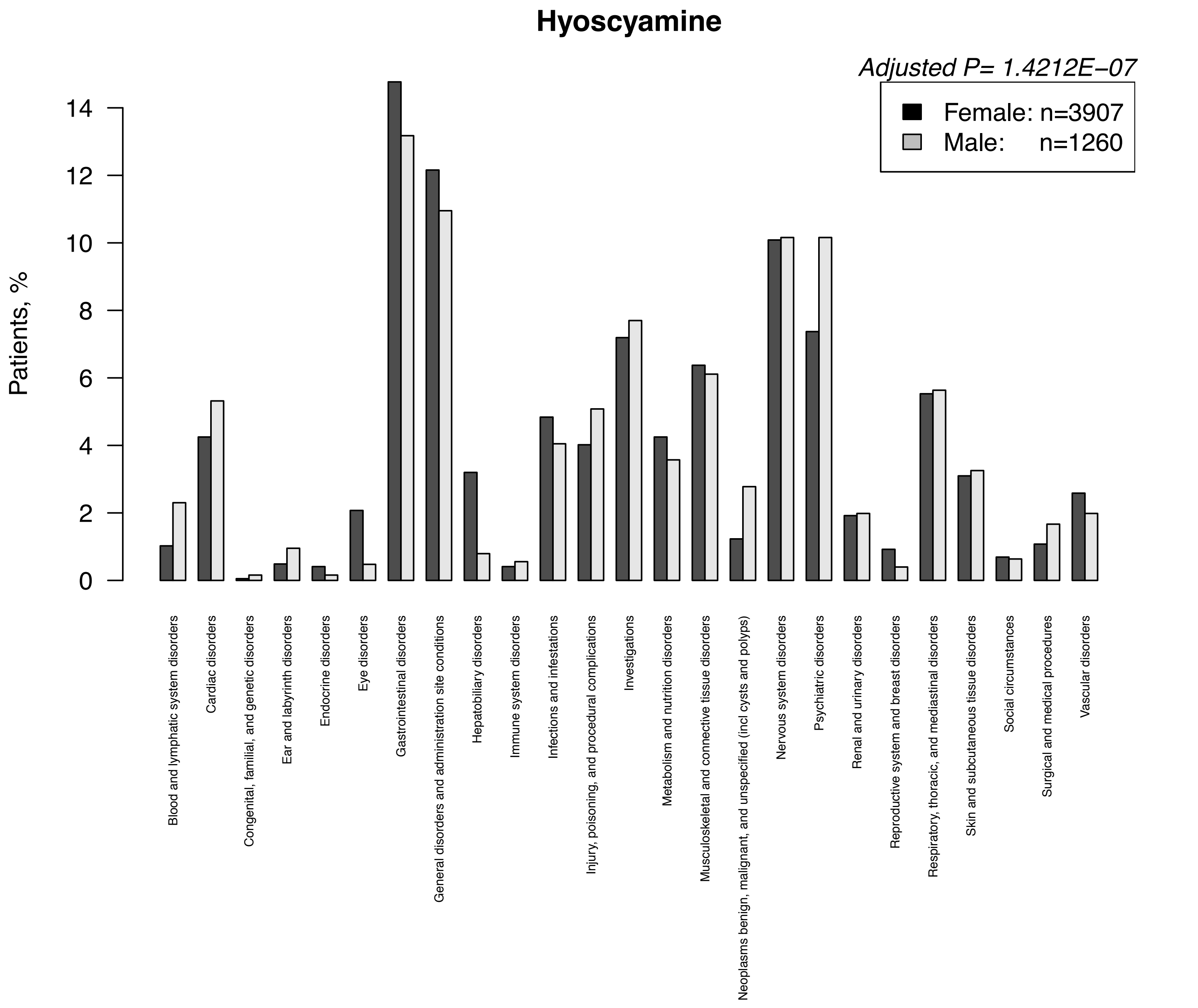

Hyoscyamine
Adjusted P= 1.4212E−07
Female: n=3907
Male: n=1260
Patients, %
0
2
4
6
8
10
12
14
Blood and lymphatic system disorders
Cardiac disorders
Congenital, familial, and genetic disorders
Ear and labyrinth disorders
Endocrine disorders
Eye disorders
Gastrointestinal disorders
General disorders and administration site conditions
Hepatobiliary disorders
Immune system disorders
Infections and infestations
Injury, poisoning, and procedural complications
Investigations
Metabolism and nutrition disorders
Musculoskeletal and connective tissue disorders
Neoplasms benign, malignant, and unspecified (incl cysts and polyps)
Nervous system disorders
Psychiatric disorders
Renal and urinary disorders
Reproductive system and breast disorders
Respiratory, thoracic, and mediastinal disorders
Skin and subcutaneous tissue disorders
Social circumstances
Surgical and medical procedures
Vascular disorders

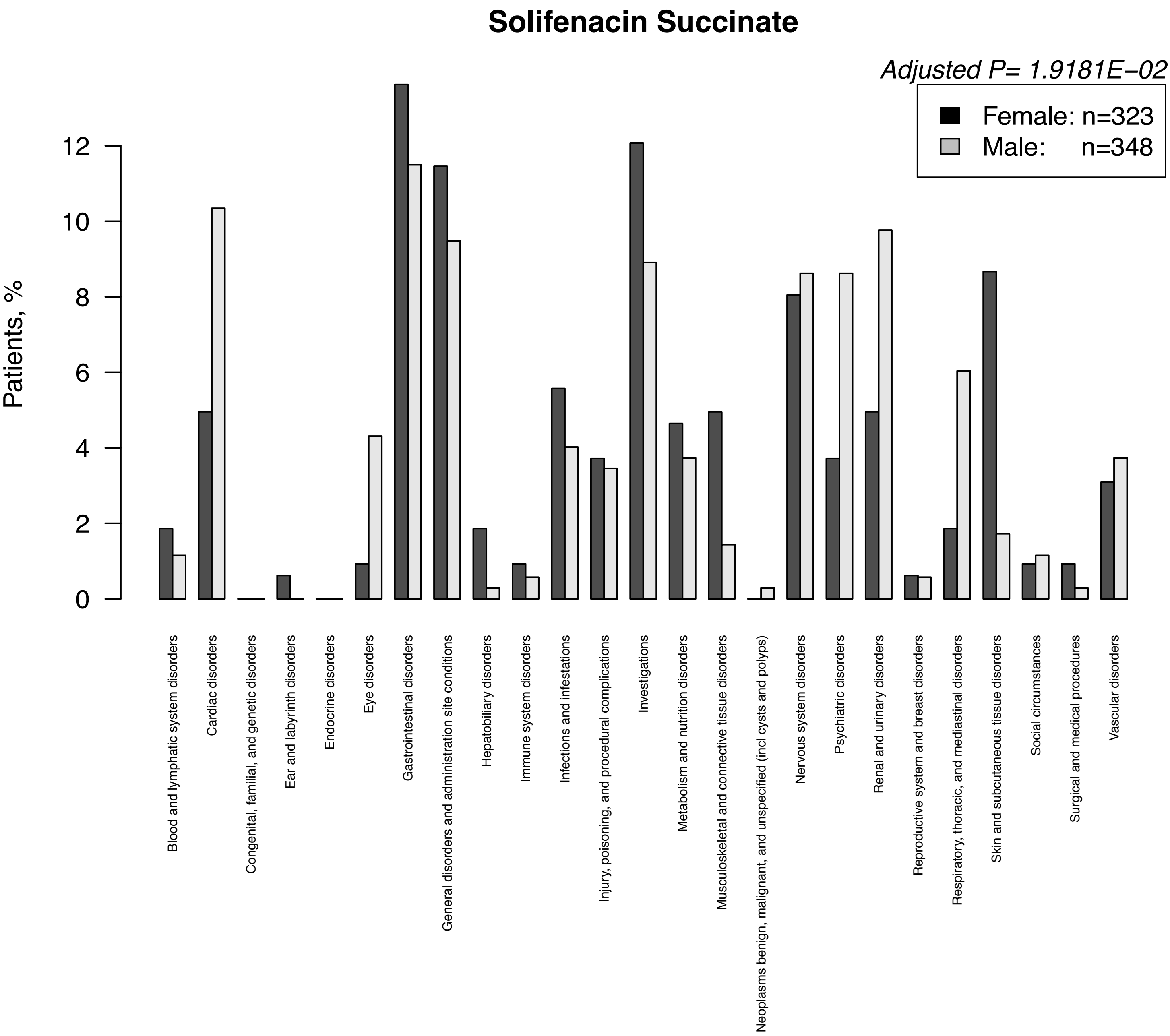
Solifenacin Succinate
Adjusted P= 1.9181E−02
Female: n=323
Male: n=348
Patients, %
0
2
4
6
8
10
12
Blood and lymphatic system disorders
Cardiac disorders
Congenital, familial, and genetic disorders
Ear and labyrinth disorders
Endocrine disorders
Eye disorders
Gastrointestinal disorders
General disorders and administration site conditions
Hepatobiliary disorders
Immune system disorders
Infections and infestations
Injury, poisoning, and procedural complications
Investigations
Metabolism and nutrition disorders
Musculoskeletal and connective tissue disorders
Neoplasms benign, malignant, and unspecified (incl cysts and polyps)
Nervous system disorders
Psychiatric disorders
Renal and urinary disorders
Reproductive system and breast disorders
Respiratory, thoracic, and mediastinal disorders
Skin and subcutaneous tissue disorders
Social circumstances
Surgical and medical procedures
Vascular disorders

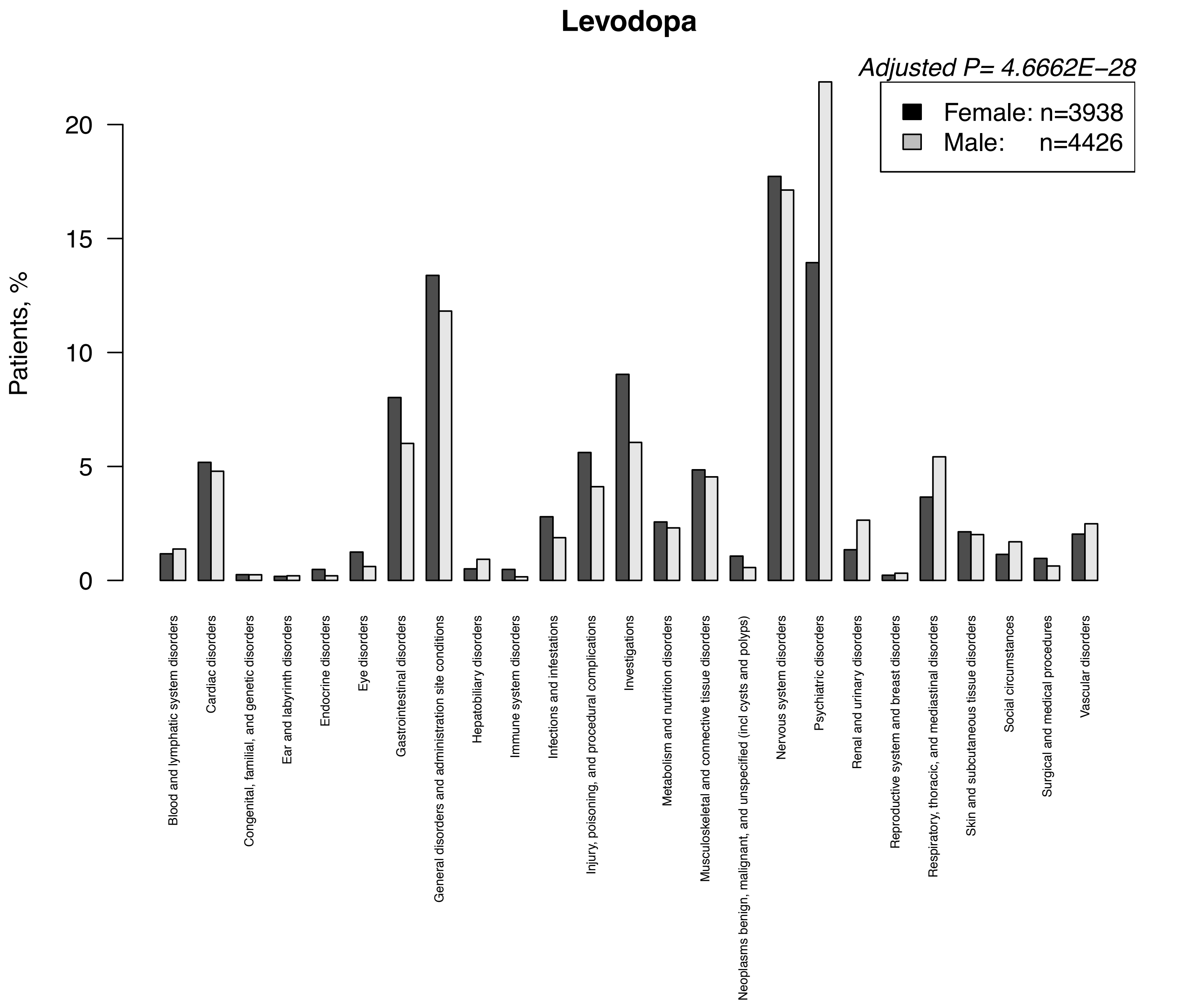

Levodopa
Adjusted P= 4.6662E−28
Female: n=3938
Male: n=4426
Patients, %
0
5
10
15
20
Blood and lymphatic system disorders
Cardiac disorders
Congenital, familial, and genetic disorders
Ear and labyrinth disorders
Endocrine disorders
Eye disorders
Gastrointestinal disorders
General disorders and administration site conditions
Hepatobiliary disorders
Immune system disorders
Infections and infestations
Injury, poisoning, and procedural complications
Investigations
Metabolism and nutrition disorders
Musculoskeletal and connective tissue disorders
Neoplasms benign, malignant, and unspecified (incl cysts and polyps)
Nervous system disorders
Psychiatric disorders
Renal and urinary disorders
Reproductive system and breast disorders
Respiratory, thoracic, and mediastinal disorders
Skin and subcutaneous tissue disorders
Social circumstances
Surgical and medical procedures
Vascular disorders

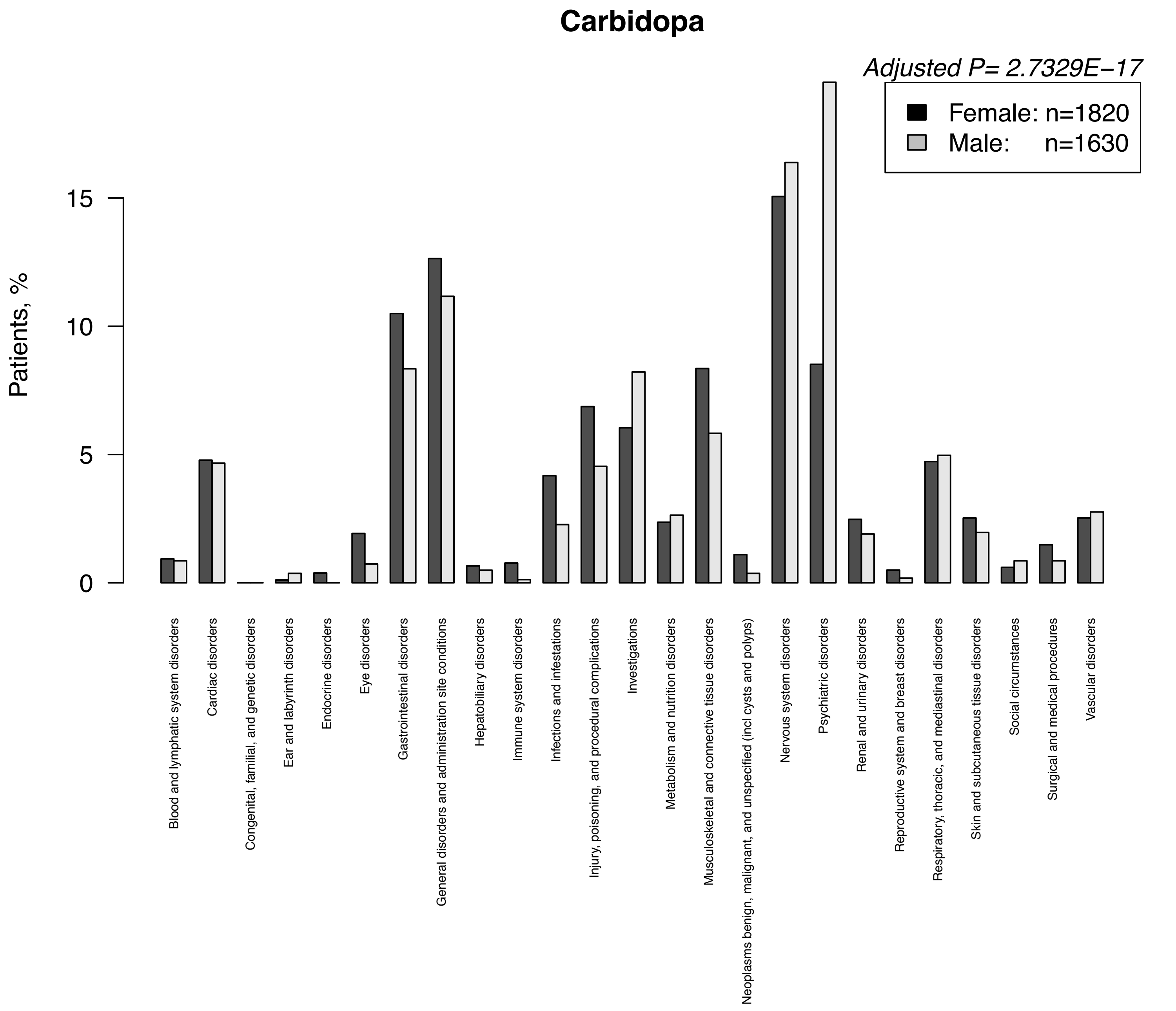
Carbidopa
Adjusted P= 2.7329E−17
Female: n=1820
Male: n=1630
Patients, %
0
5
10
15
Blood and lymphatic system disorders
Cardiac disorders
Congenital, familial, and genetic disorders
Ear and labyrinth disorders
Endocrine disorders
Eye disorders
Gastrointestinal disorders
General disorders and administration site conditions
Hepatobiliary disorders
Immune system disorders
Infections and infestations
Injury, poisoning, and procedural complications
Investigations
Metabolism and nutrition disorders
Musculoskeletal and connective tissue disorders
Neoplasms benign, malignant, and unspecified (incl cysts and polyps)
Nervous system disorders
Psychiatric disorders
Renal and urinary disorders
Reproductive system and breast disorders
Respiratory, thoracic, and mediastinal disorders
Skin and subcutaneous tissue disorders
Social circumstances
Surgical and medical procedures
Vascular disorders

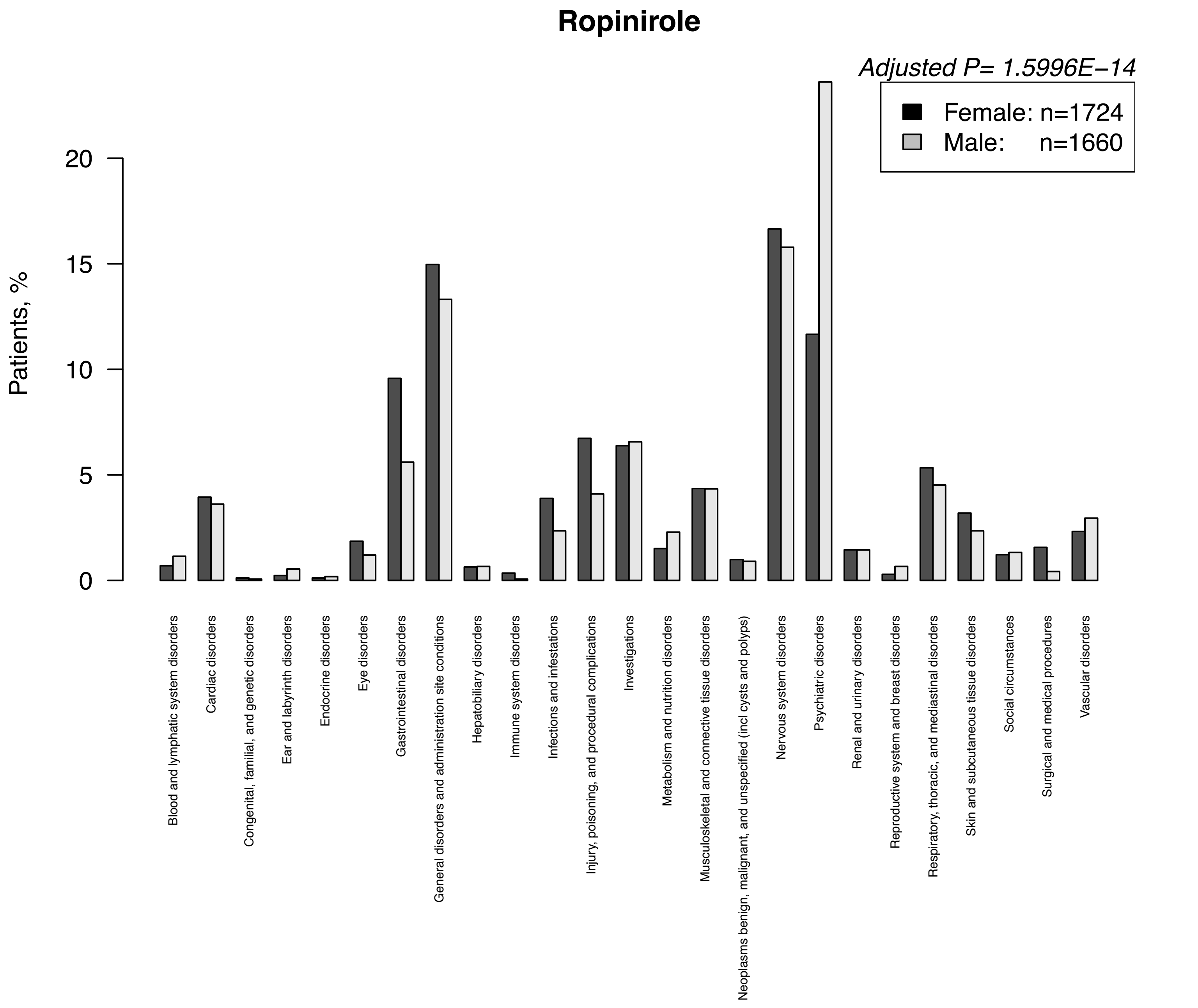

Ropinirole
Adjusted P= 1.5996E−14
Female: n=1724
Male: n=1660
Patients, %
0
5
10
15
20
Blood and lymphatic system disorders
Cardiac disorders
Congenital, familial, and genetic disorders
Ear and labyrinth disorders
Endocrine disorders
Eye disorders
Gastrointestinal disorders
General disorders and administration site conditions
Hepatobiliary disorders
Immune system disorders
Infections and infestations
Injury, poisoning, and procedural complications
Investigations
Metabolism and nutrition disorders
Musculoskeletal and connective tissue disorders
Neoplasms benign, malignant, and unspecified (incl cysts and polyps)
Nervous system disorders
Psychiatric disorders
Renal and urinary disorders
Reproductive system and breast disorders
Respiratory, thoracic, and mediastinal disorders
Skin and subcutaneous tissue disorders
Social circumstances
Surgical and medical procedures
Vascular disorders

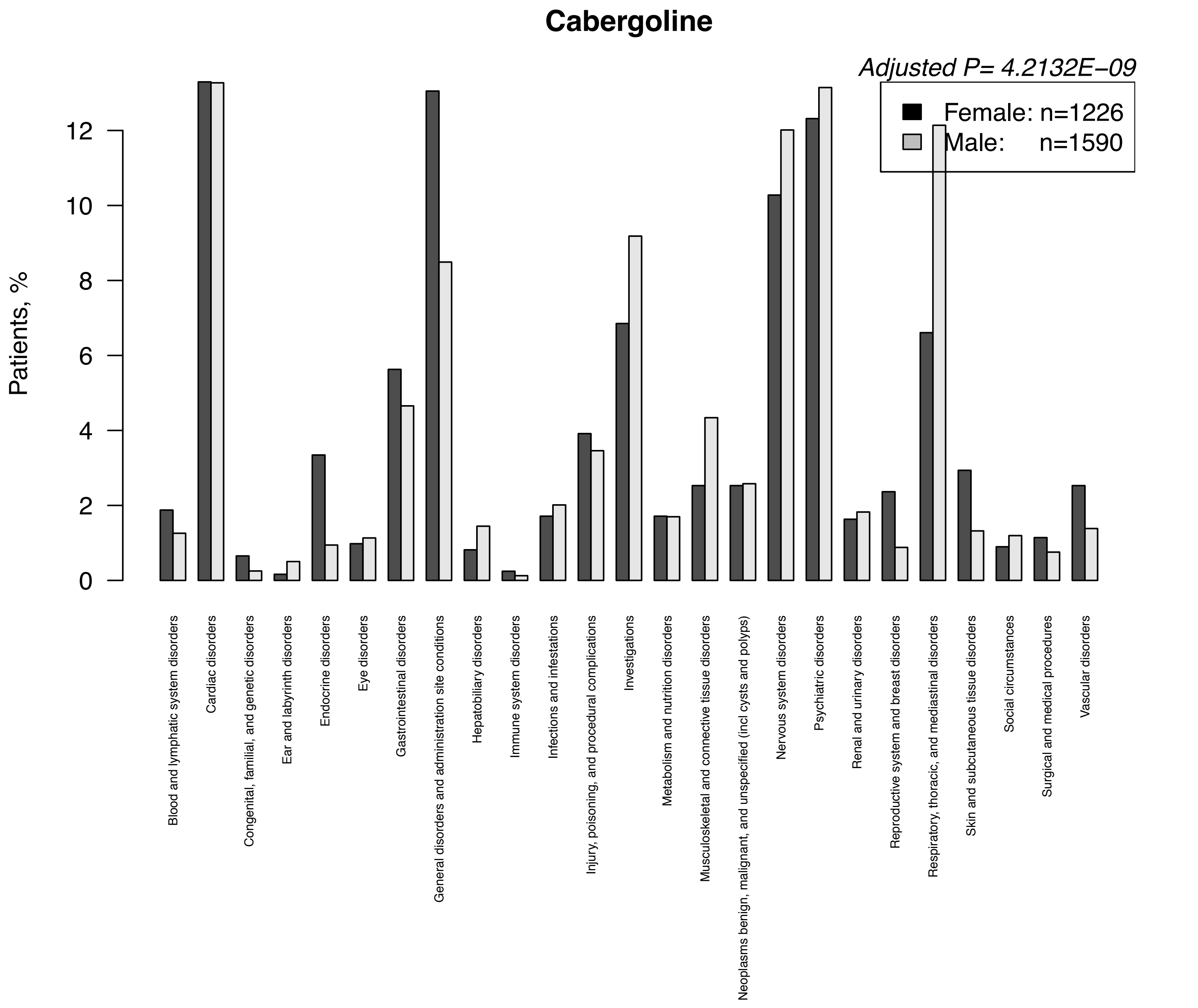
Cabergoline
Adjusted P= 4.2132E−09
Female: n=1226
Male: n=1590
Patients, %
0
2
4
6
8
10
12
Blood and lymphatic system disorders
Cardiac disorders
Congenital, familial, and genetic disorders
Ear and labyrinth disorders
Endocrine disorders
Eye disorders
Gastrointestinal disorders
General disorders and administration site conditions
Hepatobiliary disorders
Immune system disorders
Infections and infestations
Injury, poisoning, and procedural complications
Investigations
Metabolism and nutrition disorders
Musculoskeletal and connective tissue disorders
Neoplasms benign, malignant, and unspecified (incl cysts and polyps)
Nervous system disorders
Psychiatric disorders
Renal and urinary disorders
Reproductive system and breast disorders
Respiratory, thoracic, and mediastinal disorders
Skin and subcutaneous tissue disorders
Social circumstances
Surgical and medical procedures
Vascular disorders

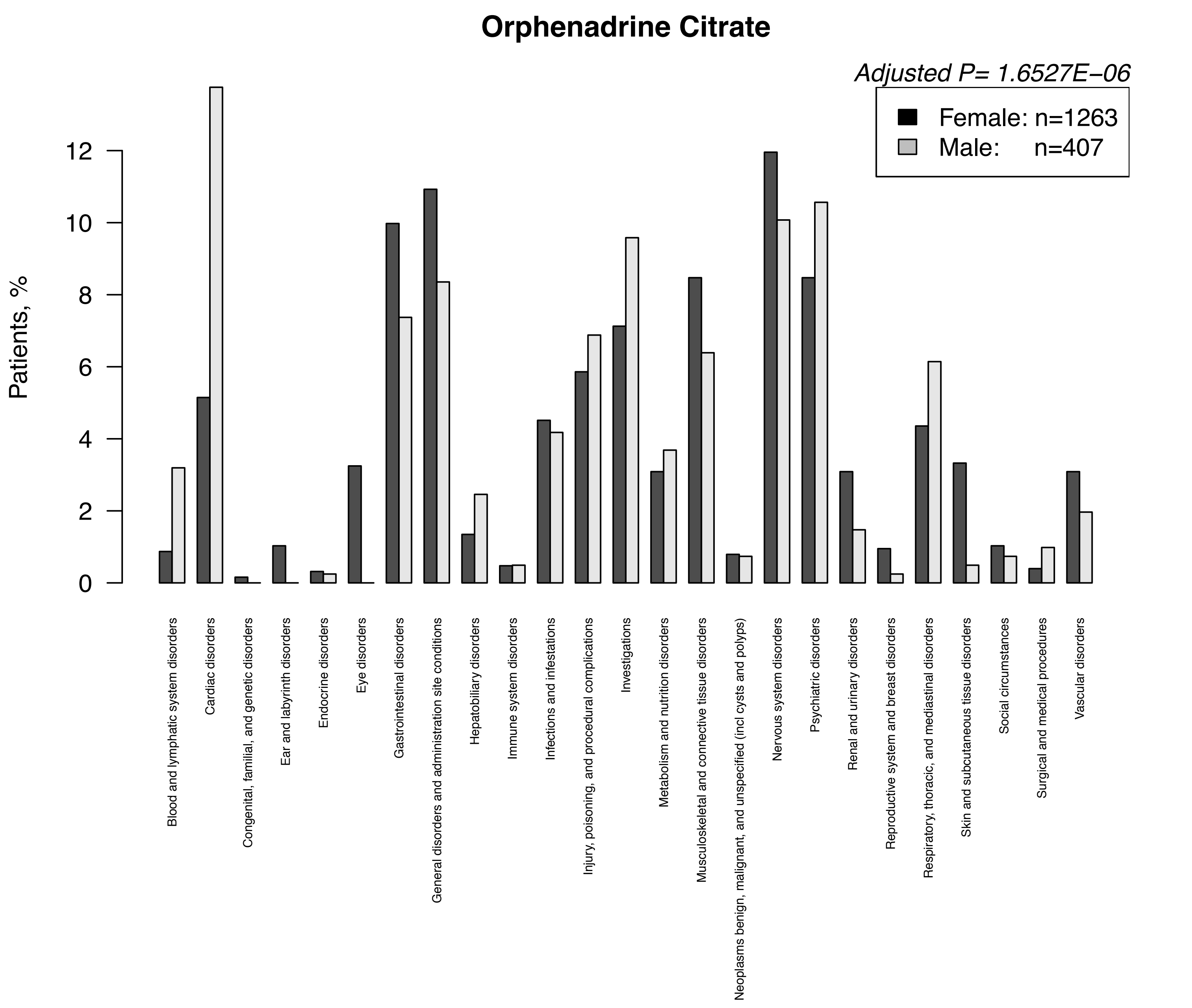
Orphenadrine Citrate
Adjusted P= 1.6527E−06
Female: n=1263
Male: n=407
Patients, %
0
2
4
6
8
10
12
Blood and lymphatic system disorders
Cardiac disorders
Congenital, familial, and genetic disorders
Ear and labyrinth disorders
Endocrine disorders
Eye disorders
Gastrointestinal disorders
General disorders and administration site conditions
Hepatobiliary disorders
Immune system disorders
Infections and infestations
Injury, poisoning, and procedural complications
Investigations
Metabolism and nutrition disorders
Musculoskeletal and connective tissue disorders
Neoplasms benign, malignant, and unspecified (incl cysts and polyps)
Nervous system disorders
Psychiatric disorders
Renal and urinary disorders
Reproductive system and breast disorders
Respiratory, thoracic, and mediastinal disorders
Skin and subcutaneous tissue disorders
Social circumstances
Surgical and medical procedures
Vascular disorders

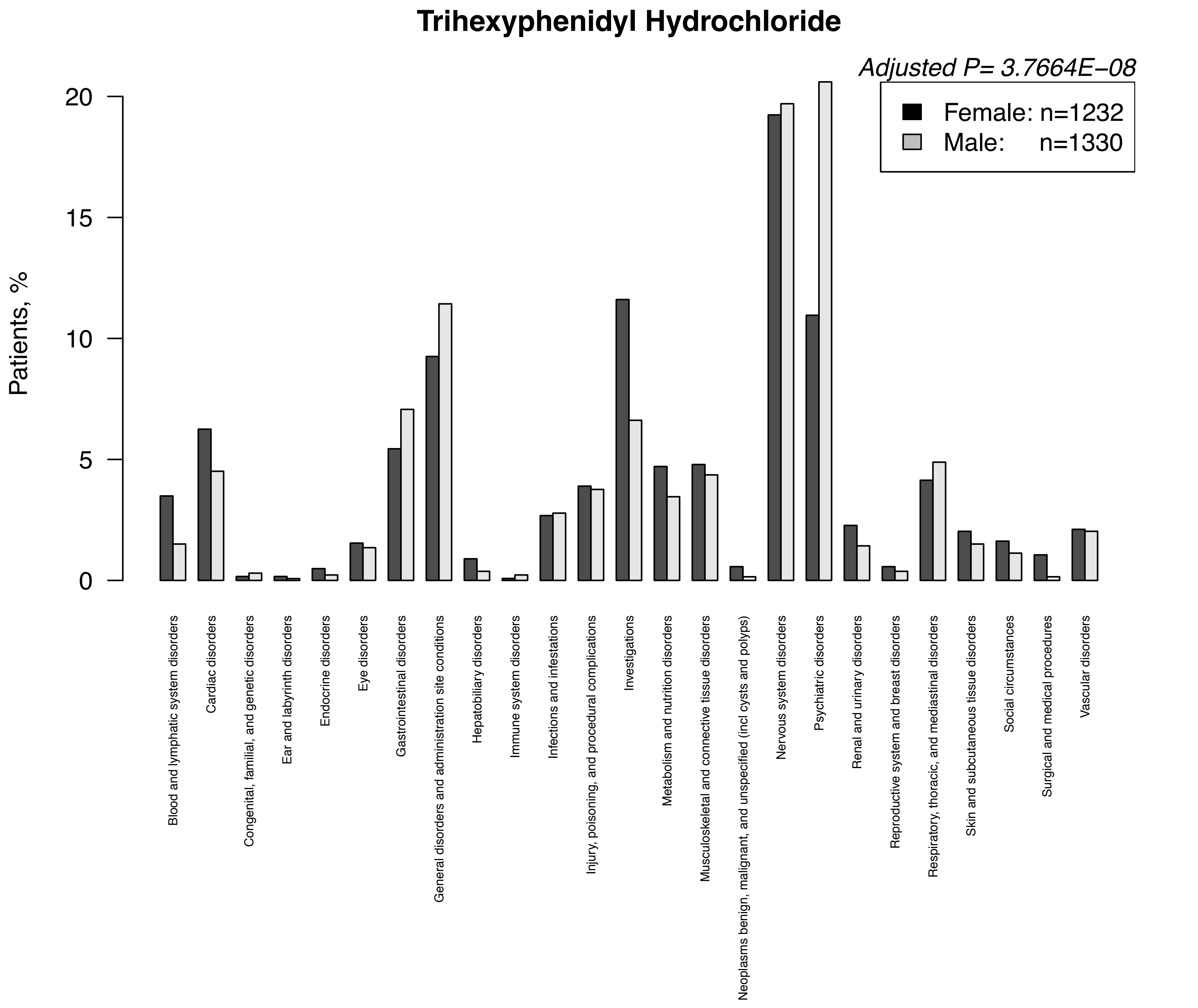
Trihexyphenidyl Hydrochloride
Adjusted P= 3.7664E−08
Female: n=1232
Male: n=1330
Patients, %
0
5
10
15
20
Blood and lymphatic system disorders
Cardiac disorders
Congenital, familial, and genetic disorders
Ear and labyrinth disorders
Endocrine disorders
Eye disorders
Gastrointestinal disorders
General disorders and administration site conditions
Hepatobiliary disorders
Immune system disorders
Infections and infestations
Injury, poisoning, and procedural complications
Investigations
Metabolism and nutrition disorders
Musculoskeletal and connective tissue disorders
Neoplasms benign, malignant, and unspecified (incl cysts and polyps)
Nervous system disorders
Psychiatric disorders
Renal and urinary disorders
Reproductive system and breast disorders
Respiratory, thoracic, and mediastinal disorders
Skin and subcutaneous tissue disorders
Social circumstances
Surgical and medical procedures
Vascular disorders

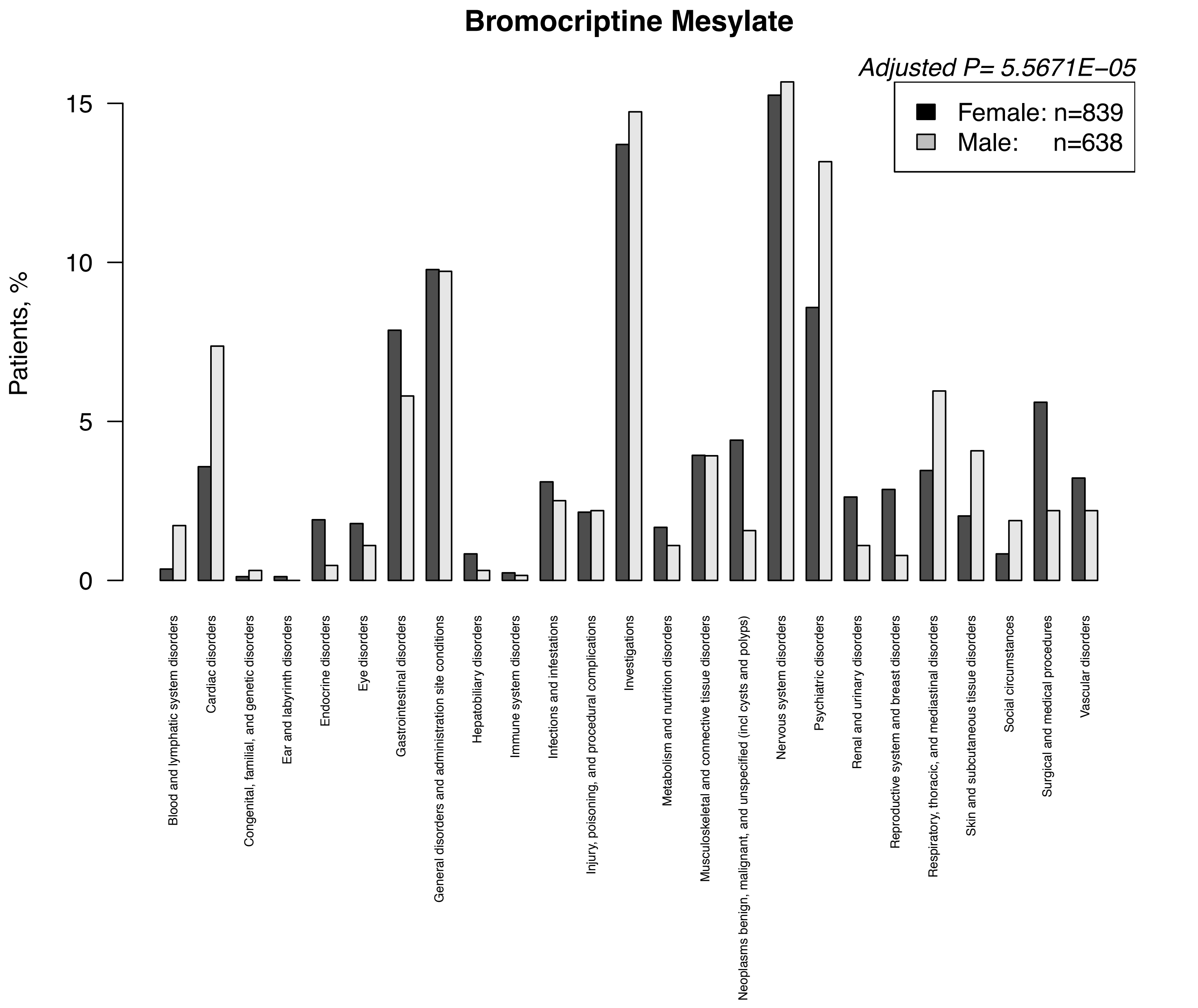

Bromocriptine Mesylate
Adjusted P= 5.5671E−05
Female: n=839
Male: n=638
Patients, %
0
5
10
15
Blood and lymphatic system disorders
Cardiac disorders
Congenital, familial, and genetic disorders
Ear and labyrinth disorders
Endocrine disorders
Eye disorders
Gastrointestinal disorders
General disorders and administration site conditions
Hepatobiliary disorders
Immune system disorders
Infections and infestations
Injury, poisoning, and procedural complications
Investigations
Metabolism and nutrition disorders
Musculoskeletal and connective tissue disorders
Neoplasms benign, malignant, and unspecified (incl cysts and polyps)
Nervous system disorders
Psychiatric disorders
Renal and urinary disorders
Reproductive system and breast disorders
Respiratory, thoracic, and mediastinal disorders
Skin and subcutaneous tissue disorders
Social circumstances
Surgical and medical procedures
Vascular disorders

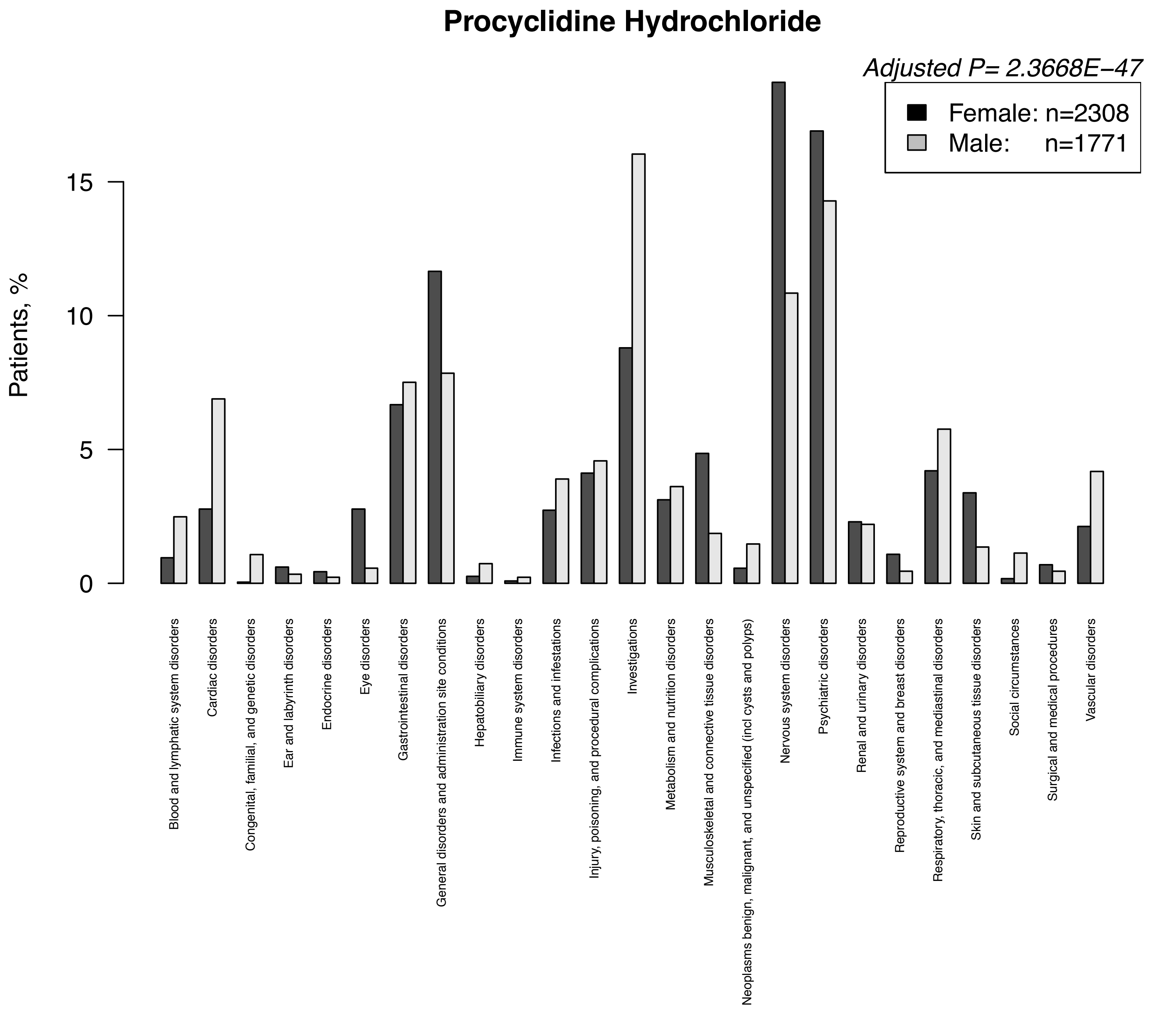
Procyclidine Hydrochloride
Adjusted P= 2.3668E−47
Female: n=2308
Male: n=1771
Patients, %
0
5
10
15
Blood and lymphatic system disorders
Cardiac disorders
Congenital, familial, and genetic disorders
Ear and labyrinth disorders
Endocrine disorders
Eye disorders
Gastrointestinal disorders
General disorders and administration site conditions
Hepatobiliary disorders
Immune system disorders
Infections and infestations
Injury, poisoning, and procedural complications
Investigations
Metabolism and nutrition disorders
Musculoskeletal and connective tissue disorders
Neoplasms benign, malignant, and unspecified (incl cysts and polyps)
Nervous system disorders
Psychiatric disorders
Renal and urinary disorders
Reproductive system and breast disorders
Respiratory, thoracic, and mediastinal disorders
Skin and subcutaneous tissue disorders
Social circumstances
Surgical and medical procedures
Vascular disorders

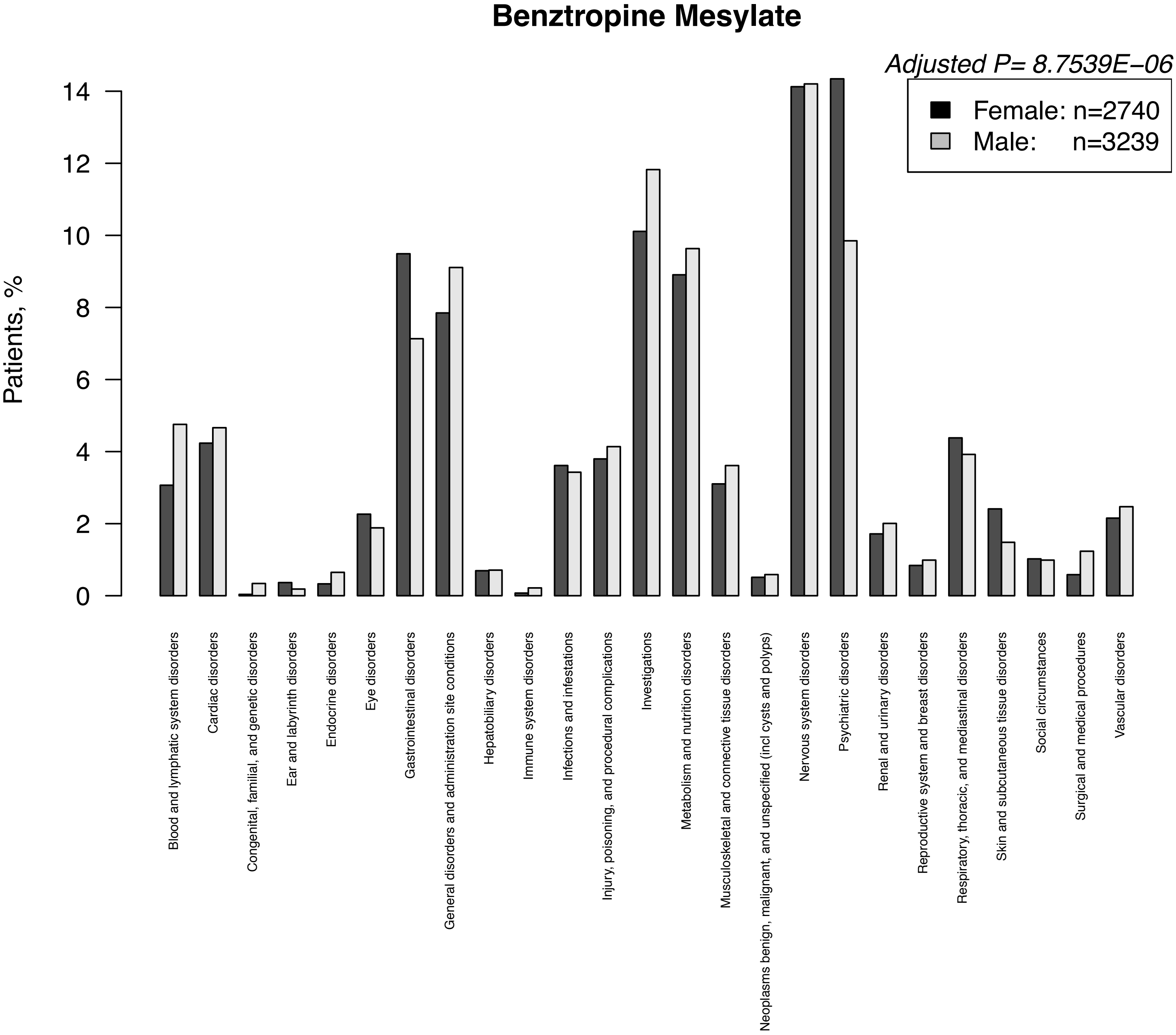
Benztropine Mesylate
Adjusted P= 8.7539E−06
Female: n=2740
Male: n=3239
Patients, %
0
2
4
6
8
10
12
14
Blood and lymphatic system disorders
Cardiac disorders
Congenital, familial, and genetic disorders
Ear and labyrinth disorders
Endocrine disorders
Eye disorders
Gastrointestinal disorders
General disorders and administration site conditions
Hepatobiliary disorders
Immune system disorders
Infections and infestations
Injury, poisoning, and procedural complications
Investigations
Metabolism and nutrition disorders
Musculoskeletal and connective tissue disorders
Neoplasms benign, malignant, and unspecified (incl cysts and polyps)
Nervous system disorders
Psychiatric disorders
Renal and urinary disorders
Reproductive system and breast disorders
Respiratory, thoracic, and mediastinal disorders
Skin and subcutaneous tissue disorders
Social circumstances
Surgical and medical procedures
Vascular disorders

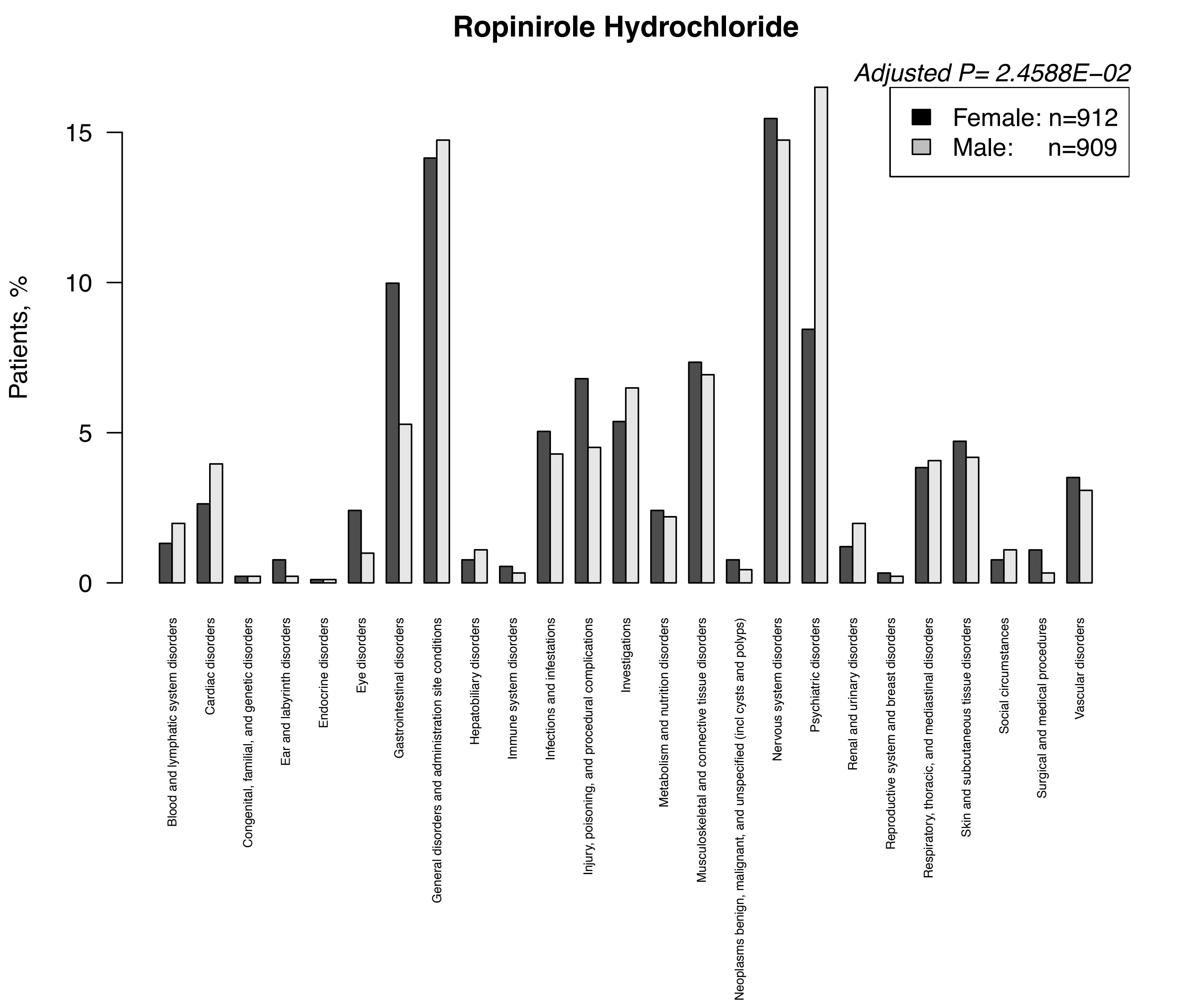

Ropinirole Hydrochloride
Adjusted P= 2.4588E−02
Female: n=912
Male: n=909
Patients, %
0
5
10
15
Blood and lymphatic system disorders
Cardiac disorders
Congenital, familial, and genetic disorders
Ear and labyrinth disorders
Endocrine disorders
Eye disorders
Gastrointestinal disorders
General disorders and administration site conditions
Hepatobiliary disorders
Immune system disorders
Infections and infestations
Injury, poisoning, and procedural complications
Investigations
Metabolism and nutrition disorders
Musculoskeletal and connective tissue disorders
Neoplasms benign, malignant, and unspecified (incl cysts and polyps)
Nervous system disorders
Psychiatric disorders
Renal and urinary disorders
Reproductive system and breast disorders
Respiratory, thoracic, and mediastinal disorders
Skin and subcutaneous tissue disorders
Social circumstances
Surgical and medical procedures
Vascular disorders

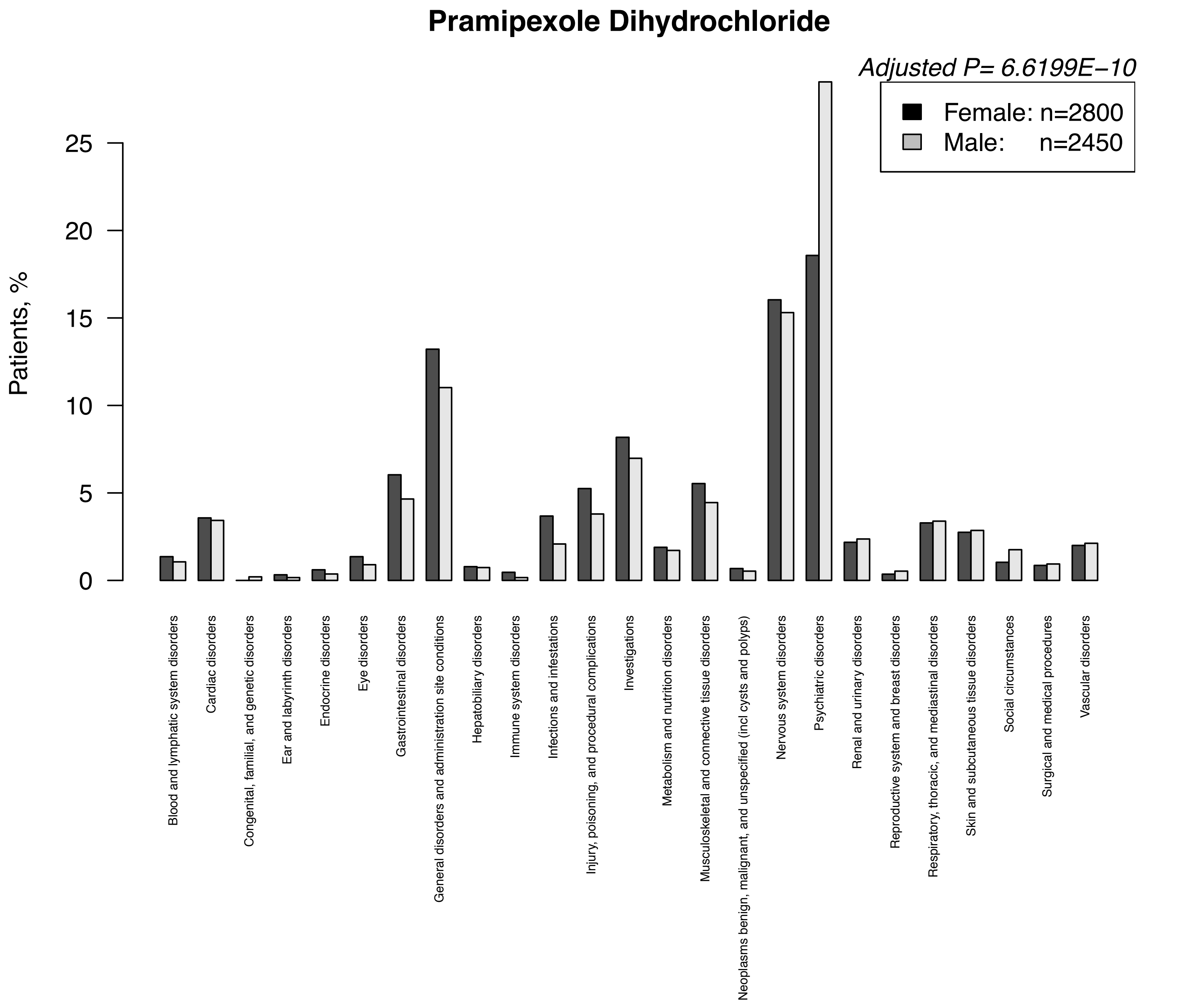
Pramipexole Dihydrochloride
Adjusted P= 6.6199E−10
Female: n=2800
Male: n=2450
Patients, %
0
5
10
15
20
25
Blood and lymphatic system disorders
Cardiac disorders
Congenital, familial, and genetic disorders
Ear and labyrinth disorders
Endocrine disorders
Eye disorders
Gastrointestinal disorders
General disorders and administration site conditions
Hepatobiliary disorders
Immune system disorders
Infections and infestations
Injury, poisoning, and procedural complications
Investigations
Metabolism and nutrition disorders
Musculoskeletal and connective tissue disorders
Neoplasms benign, malignant, and unspecified (incl cysts and polyps)
Nervous system disorders
Psychiatric disorders
Renal and urinary disorders
Reproductive system and breast disorders
Respiratory, thoracic, and mediastinal disorders
Skin and subcutaneous tissue disorders
Social circumstances
Surgical and medical procedures
Vascular disorders

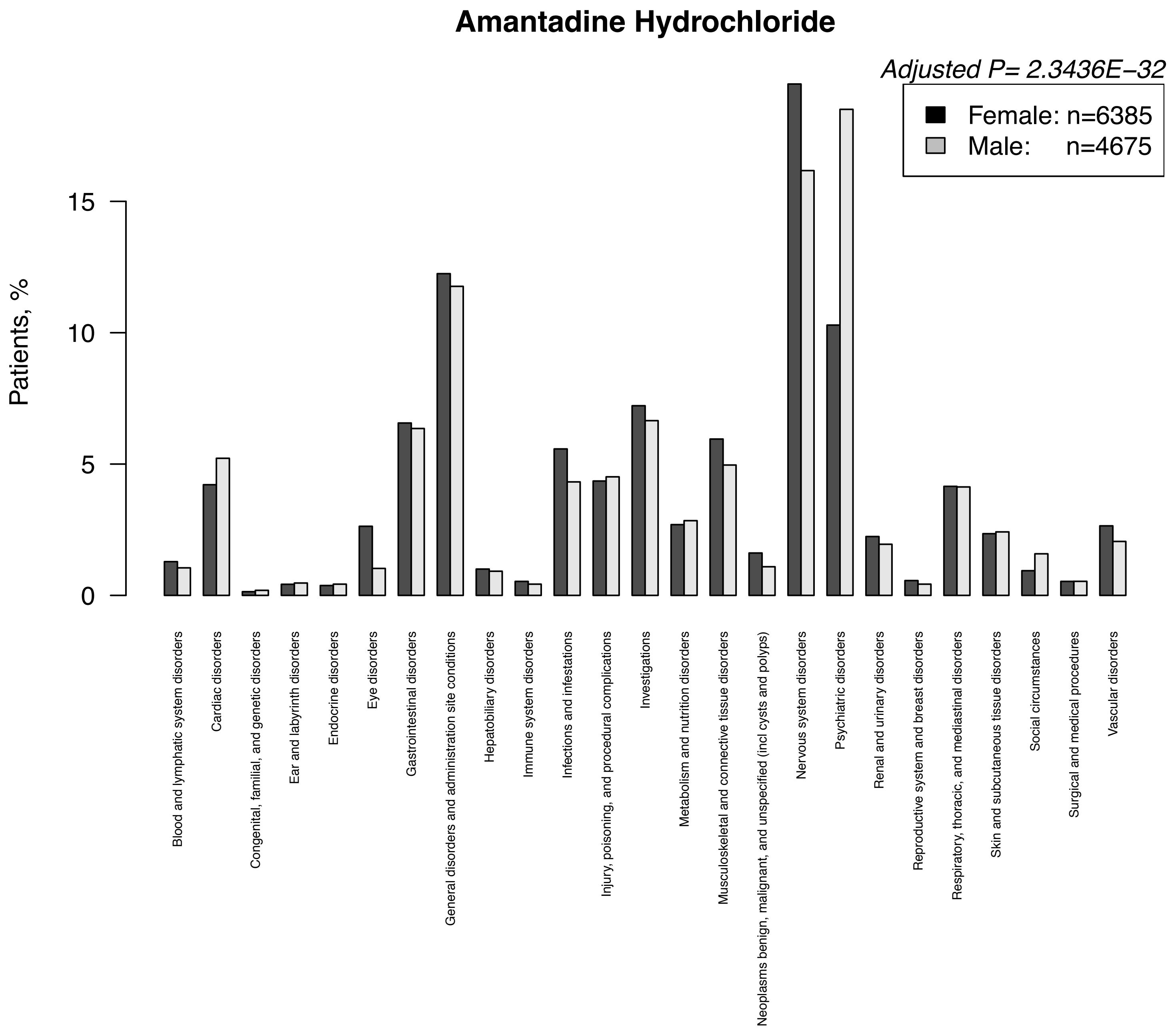
Amantadine Hydrochloride
Adjusted P= 2.3436E−32
Female: n=6385
Male: n=4675
Patients, %
0
5
10
15
Blood and lymphatic system disorders
Cardiac disorders
Congenital, familial, and genetic disorders
Ear and labyrinth disorders
Endocrine disorders
Eye disorders
Gastrointestinal disorders
General disorders and administration site conditions
Hepatobiliary disorders
Immune system disorders
Infections and infestations
Injury, poisoning, and procedural complications
Investigations
Metabolism and nutrition disorders
Musculoskeletal and connective tissue disorders
Neoplasms benign, malignant, and unspecified (incl cysts and polyps)
Nervous system disorders
Psychiatric disorders
Renal and urinary disorders
Reproductive system and breast disorders
Respiratory, thoracic, and mediastinal disorders
Skin and subcutaneous tissue disorders
Social circumstances
Surgical and medical procedures
Vascular disorders

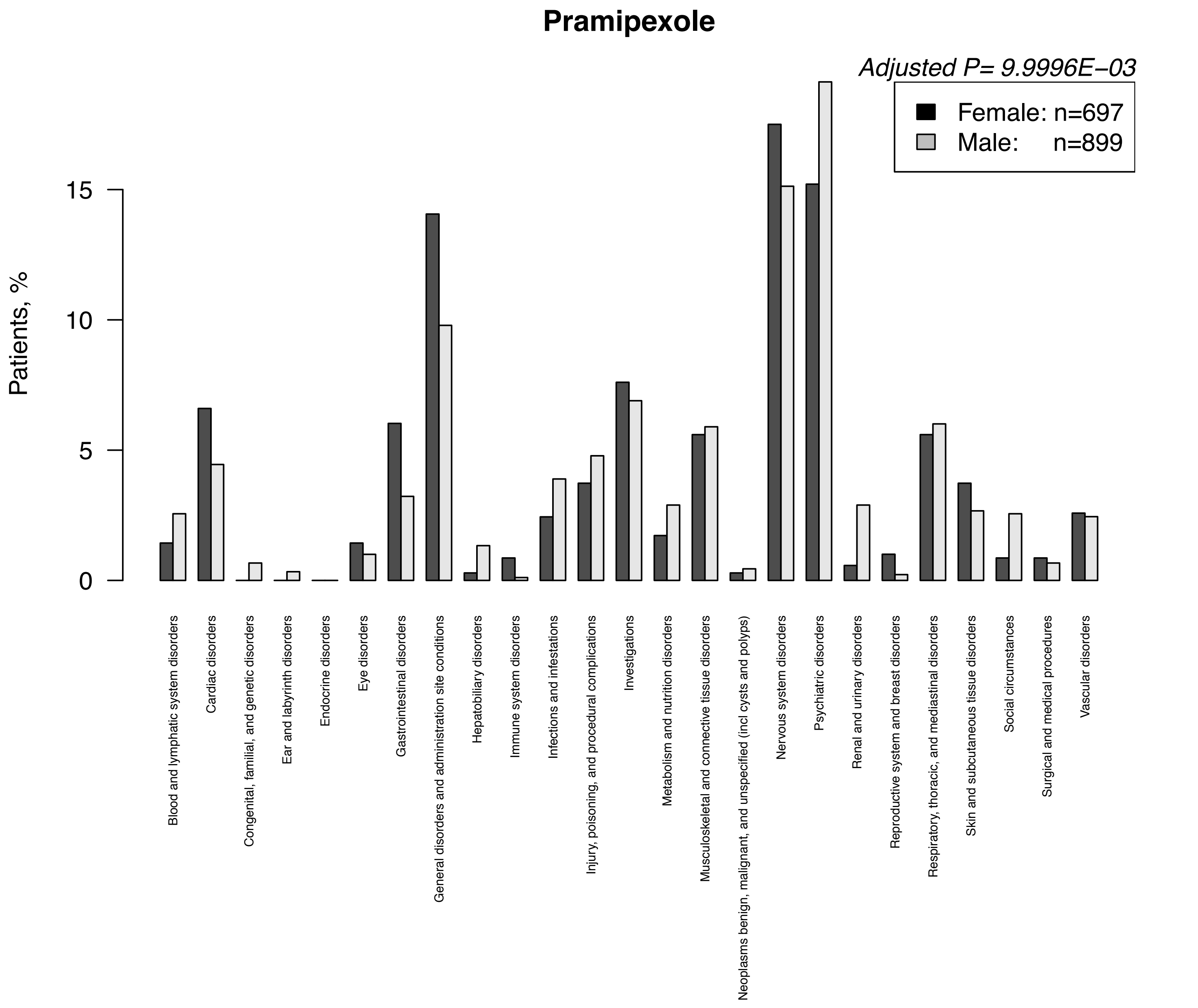
Pramipexole
Adjusted P= 9.9996E−03
Female: n=697
Male: n=899
Patients, %
0
5
10
15
Blood and lymphatic system disorders
Cardiac disorders
Congenital, familial, and genetic disorders
Ear and labyrinth disorders
Endocrine disorders
Eye disorders
Gastrointestinal disorders
General disorders and administration site conditions
Hepatobiliary disorders
Immune system disorders
Infections and infestations
Injury, poisoning, and procedural complications
Investigations
Metabolism and nutrition disorders
Musculoskeletal and connective tissue disorders
Neoplasms benign, malignant, and unspecified (incl cysts and polyps)
Nervous system disorders
Psychiatric disorders
Renal and urinary disorders
Reproductive system and breast disorders
Respiratory, thoracic, and mediastinal disorders
Skin and subcutaneous tissue disorders
Social circumstances
Surgical and medical procedures
Vascular disorders

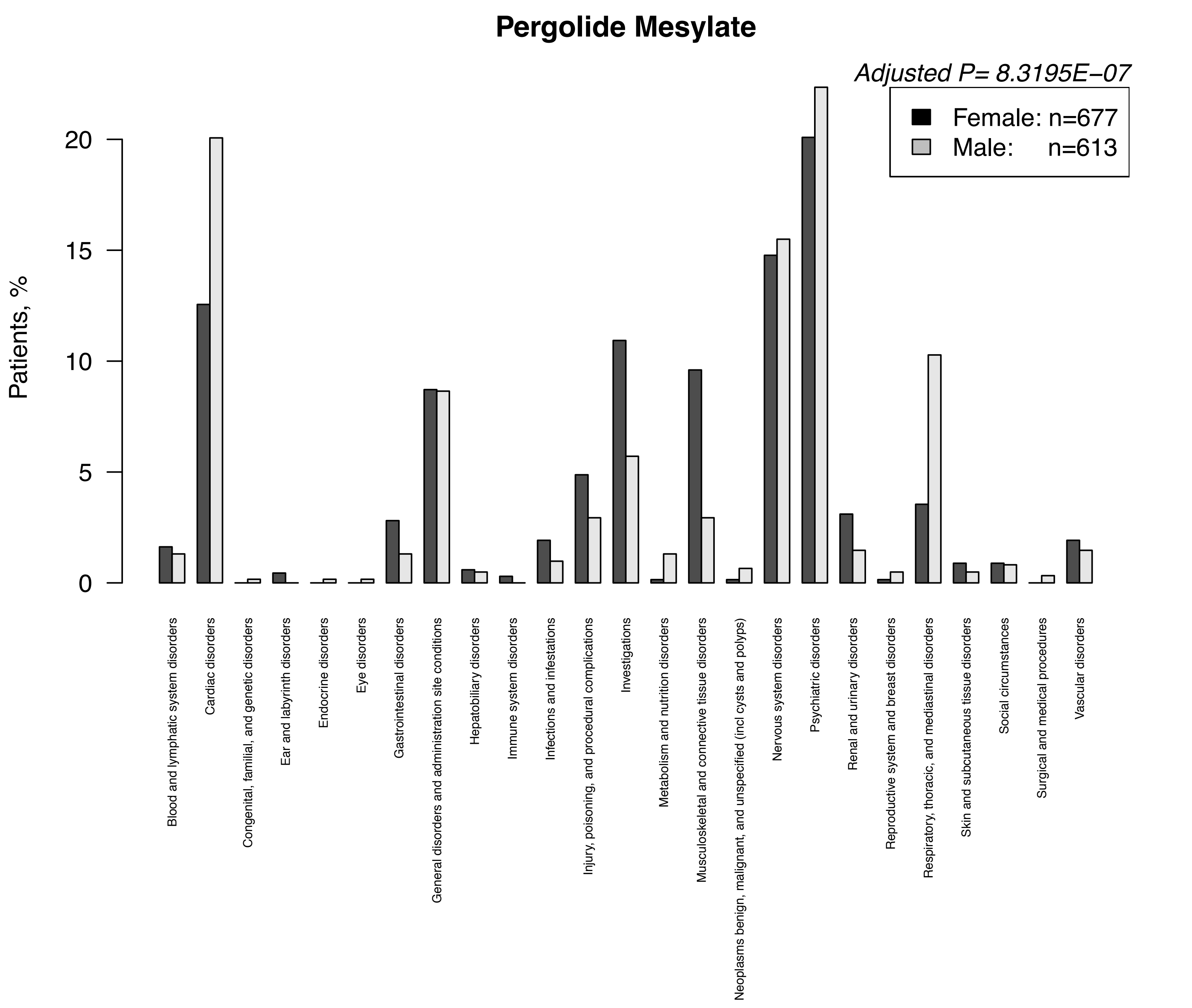

Pergolide Mesylate
Adjusted P= 8.3195E−07
Female: n=677
Male: n=613
Patients, %
0
5
10
15
20
Blood and lymphatic system disorders
Cardiac disorders
Congenital, familial, and genetic disorders
Ear and labyrinth disorders
Endocrine disorders
Eye disorders
Gastrointestinal disorders
General disorders and administration site conditions
Hepatobiliary disorders
Immune system disorders
Infections and infestations
Injury, poisoning, and procedural complications
Investigations
Metabolism and nutrition disorders
Musculoskeletal and connective tissue disorders
Neoplasms benign, malignant, and unspecified (incl cysts and polyps)
Nervous system disorders
Psychiatric disorders
Renal and urinary disorders
Reproductive system and breast disorders
Respiratory, thoracic, and mediastinal disorders
Skin and subcutaneous tissue disorders
Social circumstances
Surgical and medical procedures
Vascular disorders

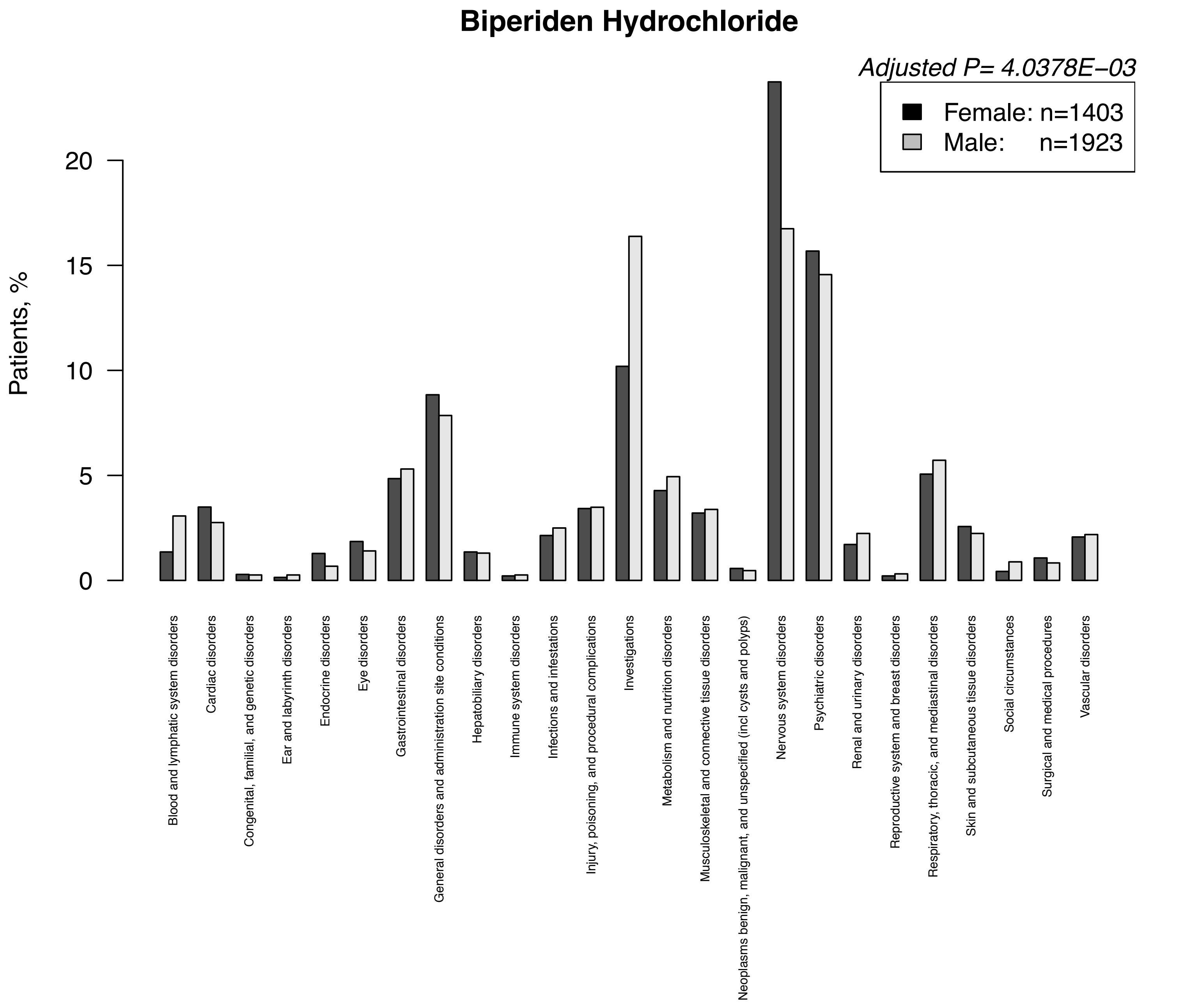

Biperiden Hydrochloride
Adjusted P= 4.0378E−03
Female: n=1403
Male: n=1923
Patients, %
0
5
10
15
20
Blood and lymphatic system disorders
Cardiac disorders
Congenital, familial, and genetic disorders
Ear and labyrinth disorders
Endocrine disorders
Eye disorders
Gastrointestinal disorders
General disorders and administration site conditions
Hepatobiliary disorders
Immune system disorders
Infections and infestations
Injury, poisoning, and procedural complications
Investigations
Metabolism and nutrition disorders
Musculoskeletal and connective tissue disorders
Neoplasms benign, malignant, and unspecified (incl cysts and polyps)
Nervous system disorders
Psychiatric disorders
Renal and urinary disorders
Reproductive system and breast disorders
Respiratory, thoracic, and mediastinal disorders
Skin and subcutaneous tissue disorders
Social circumstances
Surgical and medical procedures
Vascular disorders

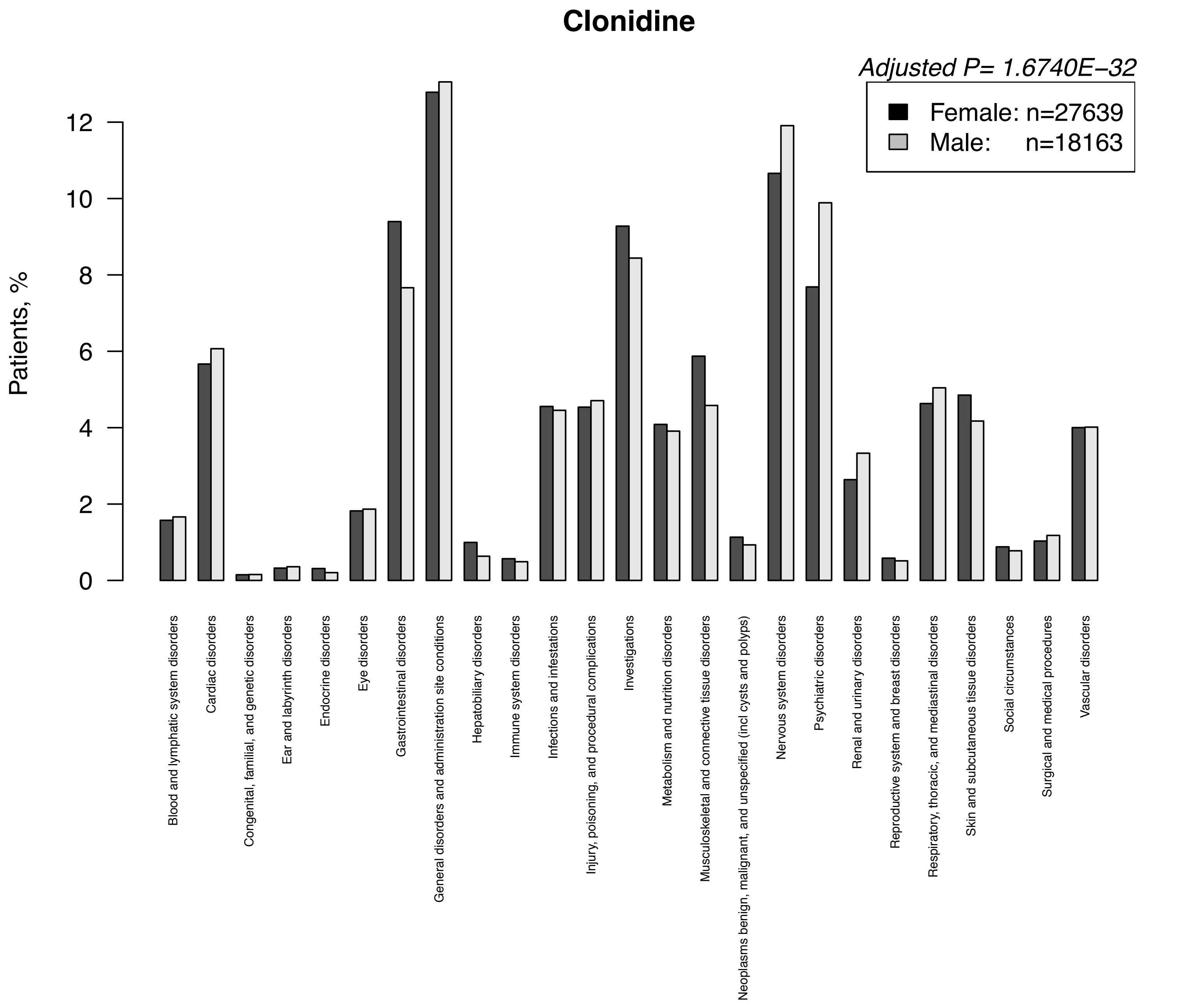
Clonidine
Adjusted P= 1.6740E−32
Female: n=27639
Male: n=18163
Patients, %
0
2
4
6
8
10
12
Blood and lymphatic system disorders
Cardiac disorders
Congenital, familial, and genetic disorders
Ear and labyrinth disorders
Endocrine disorders
Eye disorders
Gastrointestinal disorders
General disorders and administration site conditions
Hepatobiliary disorders
Immune system disorders
Infections and infestations
Injury, poisoning, and procedural complications
Investigations
Metabolism and nutrition disorders
Musculoskeletal and connective tissue disorders
Neoplasms benign, malignant, and unspecified (incl cysts and polyps)
Nervous system disorders
Psychiatric disorders
Renal and urinary disorders
Reproductive system and breast disorders
Respiratory, thoracic, and mediastinal disorders
Skin and subcutaneous tissue disorders
Social circumstances
Surgical and medical procedures
Vascular disorders

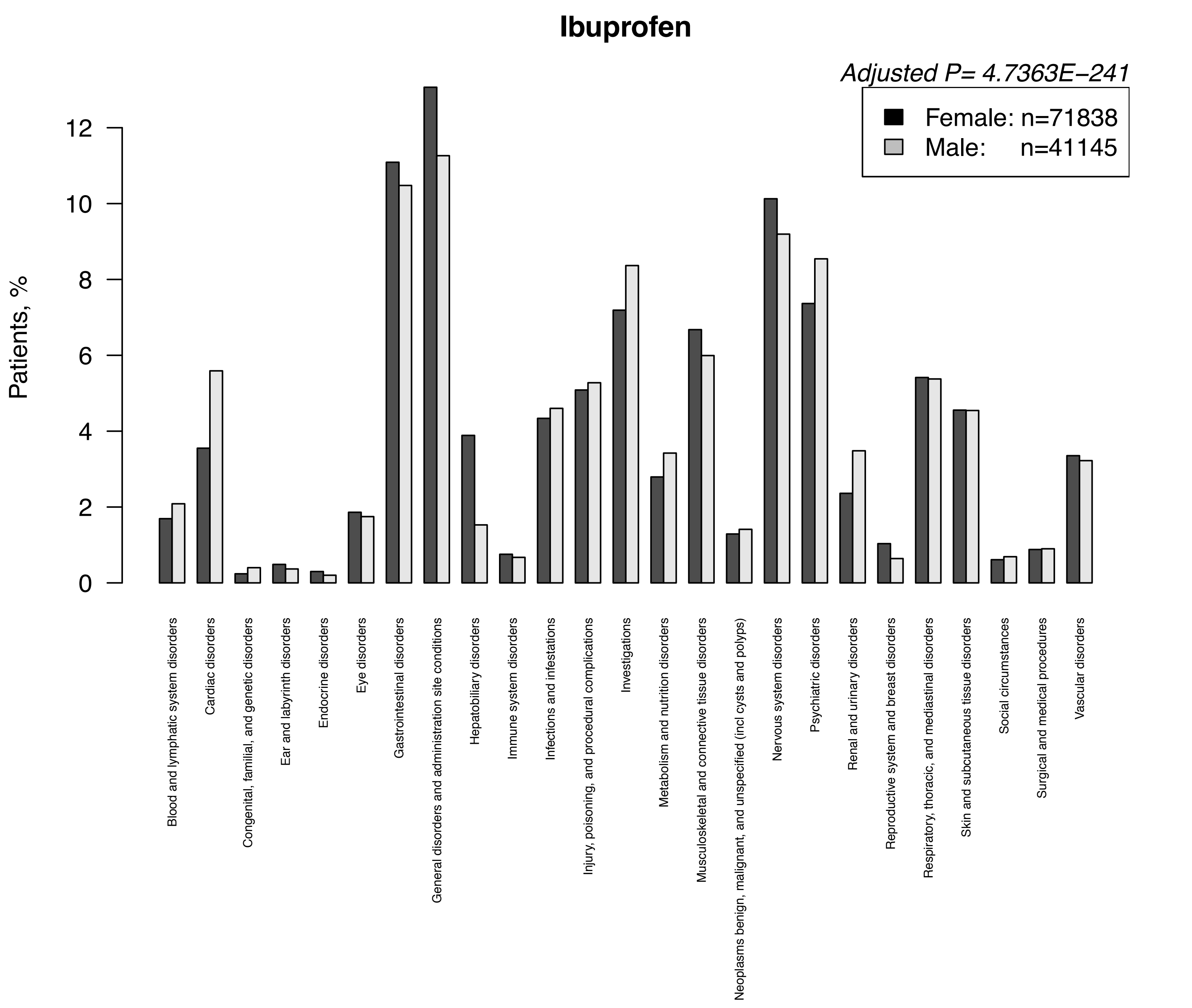
Ibuprofen
Adjusted P= 4.7363E−241
Female: n=71838
Male: n=41145
Patients, %
0
2
4
6
8
10
12
Blood and lymphatic system disorders
Cardiac disorders
Congenital, familial, and genetic disorders
Ear and labyrinth disorders
Endocrine disorders
Eye disorders
Gastrointestinal disorders
General disorders and administration site conditions
Hepatobiliary disorders
Immune system disorders
Infections and infestations
Injury, poisoning, and procedural complications
Investigations
Metabolism and nutrition disorders
Musculoskeletal and connective tissue disorders
Neoplasms benign, malignant, and unspecified (incl cysts and polyps)
Nervous system disorders
Psychiatric disorders
Renal and urinary disorders
Reproductive system and breast disorders
Respiratory, thoracic, and mediastinal disorders
Skin and subcutaneous tissue disorders
Social circumstances
Surgical and medical procedures
Vascular disorders

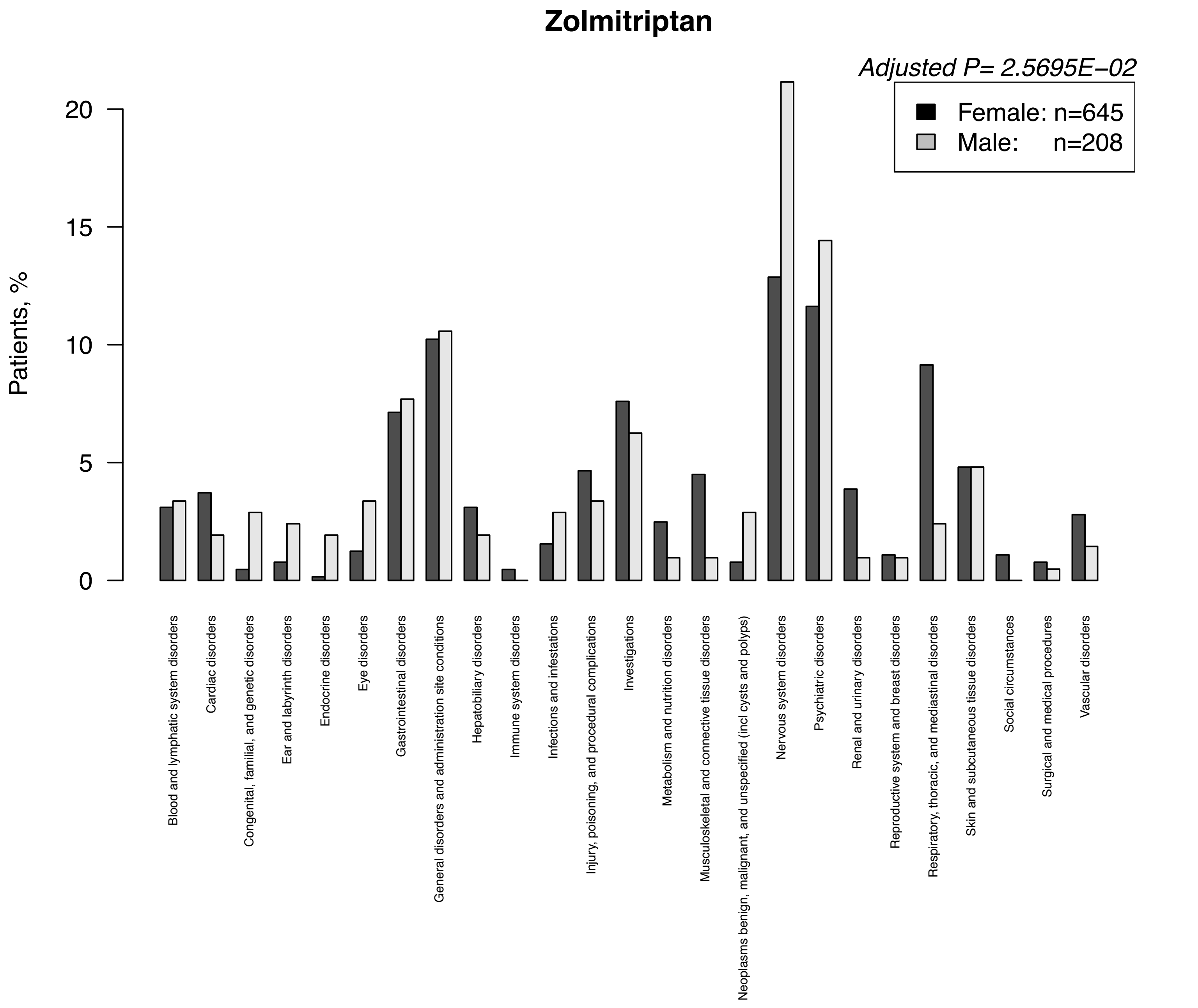
Zolmitriptan
Adjusted P= 2.5695E−02
Female: n=645
Male: n=208
Patients, %
0
5
10
15
20
Blood and lymphatic system disorders
Cardiac disorders
Congenital, familial, and genetic disorders
Ear and labyrinth disorders
Endocrine disorders
Eye disorders
Gastrointestinal disorders
General disorders and administration site conditions
Hepatobiliary disorders
Immune system disorders
Infections and infestations
Injury, poisoning, and procedural complications
Investigations
Metabolism and nutrition disorders
Musculoskeletal and connective tissue disorders
Neoplasms benign, malignant, and unspecified (incl cysts and polyps)
Nervous system disorders
Psychiatric disorders
Renal and urinary disorders
Reproductive system and breast disorders
Respiratory, thoracic, and mediastinal disorders
Skin and subcutaneous tissue disorders
Social circumstances
Surgical and medical procedures
Vascular disorders

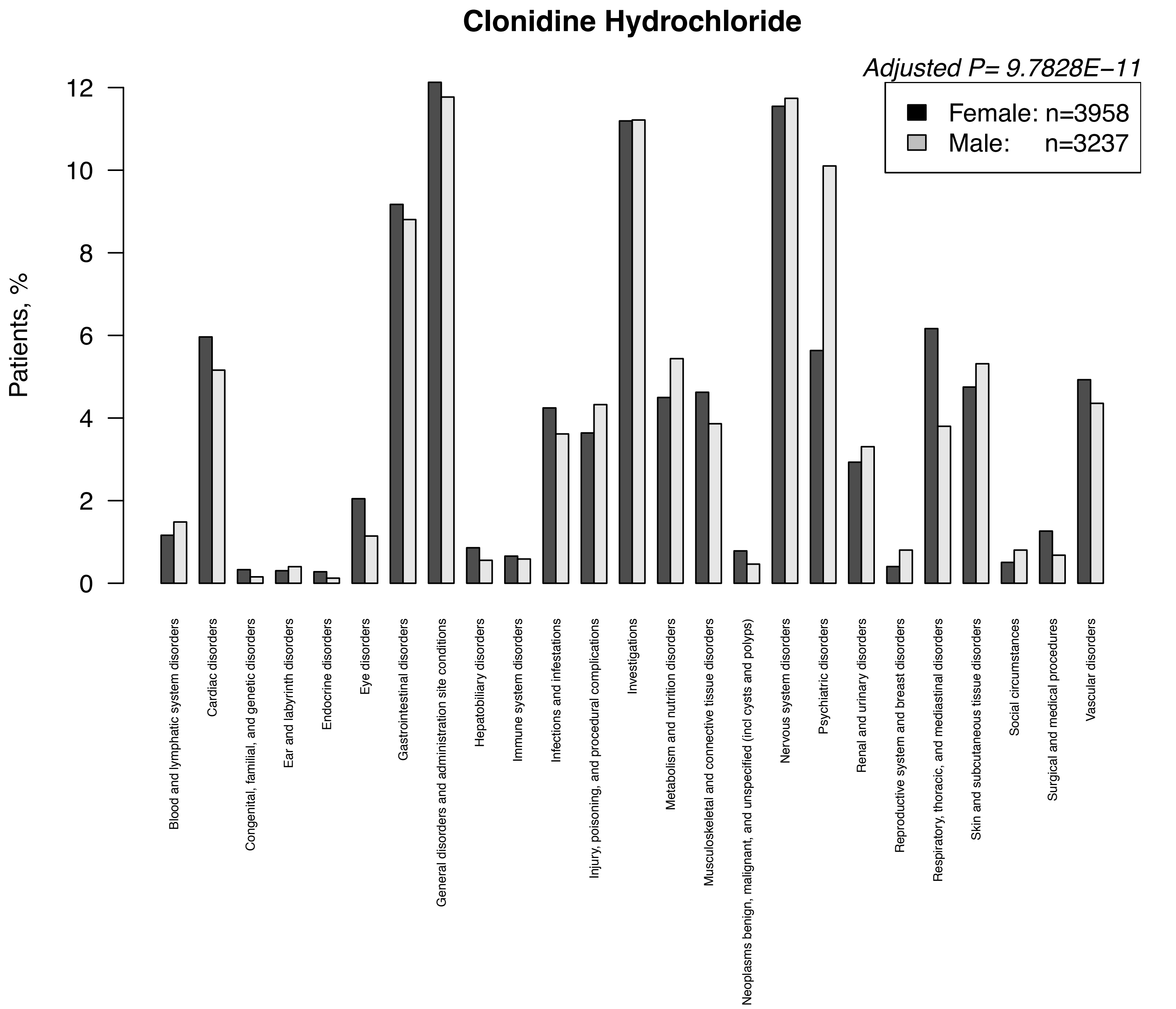
Clonidine Hydrochloride
Adjusted P= 9.7828E−11
Female: n=3958
Male: n=3237
Patients, %
0
2
4
6
8
10
12
Blood and lymphatic system disorders
Cardiac disorders
Congenital, familial, and genetic disorders
Ear and labyrinth disorders
Endocrine disorders
Eye disorders
Gastrointestinal disorders
General disorders and administration site conditions
Hepatobiliary disorders
Immune system disorders
Infections and infestations
Injury, poisoning, and procedural complications
Investigations
Metabolism and nutrition disorders
Musculoskeletal and connective tissue disorders
Neoplasms benign, malignant, and unspecified (incl cysts and polyps)
Nervous system disorders
Psychiatric disorders
Renal and urinary disorders
Reproductive system and breast disorders
Respiratory, thoracic, and mediastinal disorders
Skin and subcutaneous tissue disorders
Social circumstances
Surgical and medical procedures
Vascular disorders

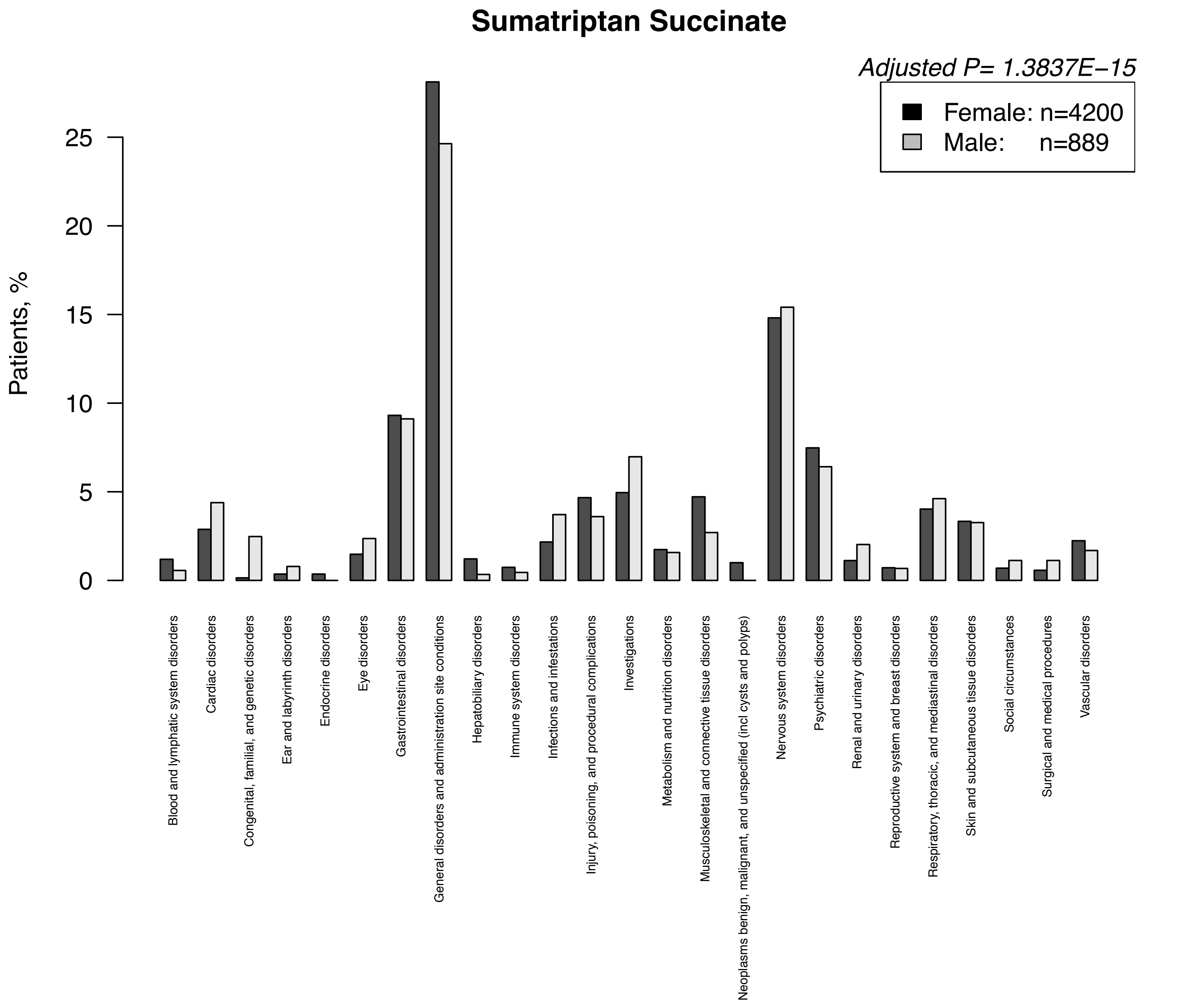

Sumatriptan Succinate
Adjusted P= 1.3837E−15
Female: n=4200
Male: n=889
Patients, %
0
5
10
15
20
25
Blood and lymphatic system disorders
Cardiac disorders
Congenital, familial, and genetic disorders
Ear and labyrinth disorders
Endocrine disorders
Eye disorders
Gastrointestinal disorders
General disorders and administration site conditions
Hepatobiliary disorders
Immune system disorders
Infections and infestations
Injury, poisoning, and procedural complications
Investigations
Metabolism and nutrition disorders
Musculoskeletal and connective tissue disorders
Neoplasms benign, malignant, and unspecified (incl cysts and polyps)
Nervous system disorders
Psychiatric disorders
Renal and urinary disorders
Reproductive system and breast disorders
Respiratory, thoracic, and mediastinal disorders
Skin and subcutaneous tissue disorders
Social circumstances
Surgical and medical procedures
Vascular disorders

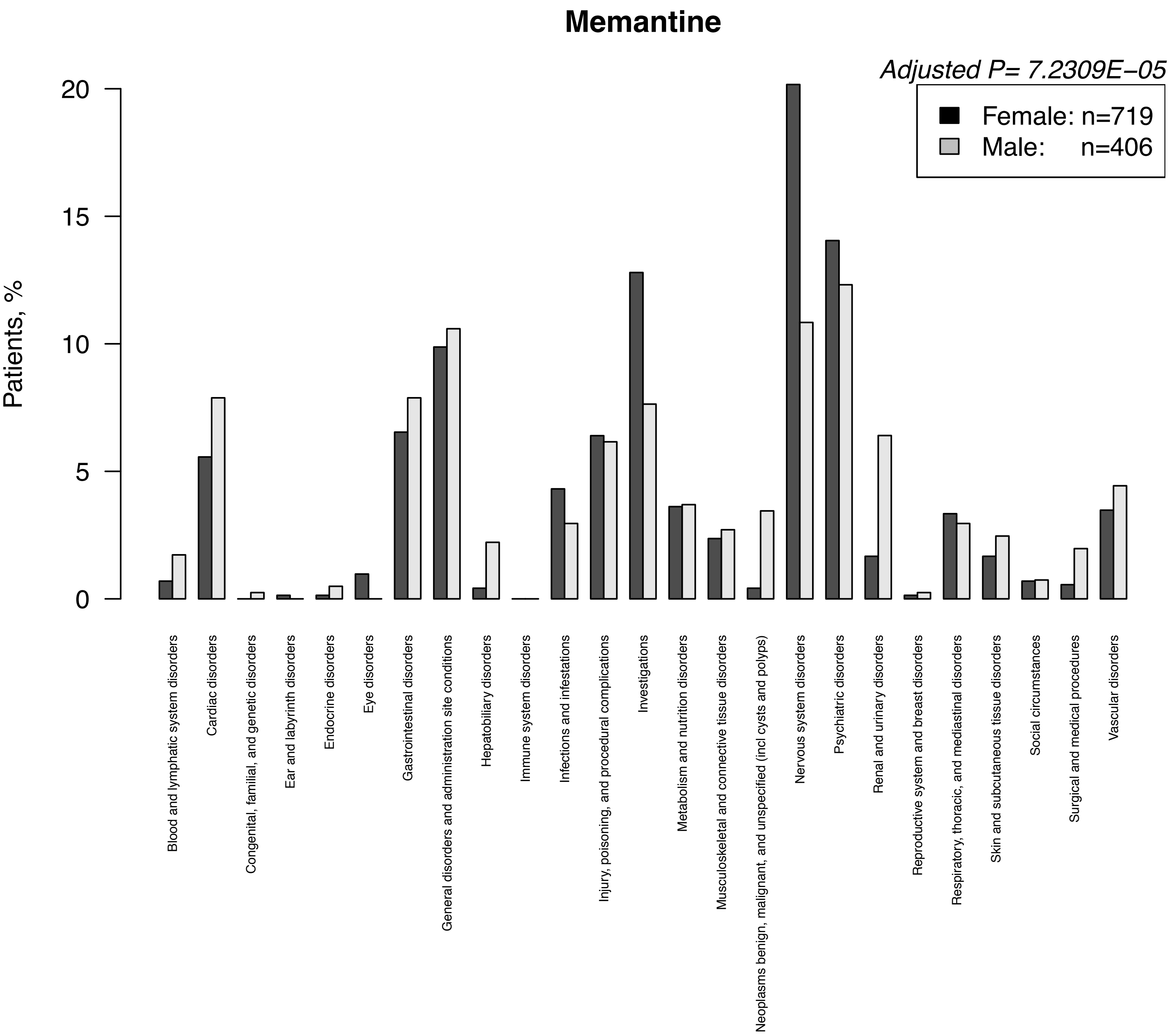

Memantine
Adjusted P= 7.2309E−05
Female: n=719
Male: n=406
Patients, %
0
5
10
15
20
Blood and lymphatic system disorders
Cardiac disorders
Congenital, familial, and genetic disorders
Ear and labyrinth disorders
Endocrine disorders
Eye disorders
Gastrointestinal disorders
General disorders and administration site conditions
Hepatobiliary disorders
Immune system disorders
Infections and infestations
Injury, poisoning, and procedural complications
Investigations
Metabolism and nutrition disorders
Musculoskeletal and connective tissue disorders
Neoplasms benign, malignant, and unspecified (incl cysts and polyps)
Nervous system disorders
Psychiatric disorders
Renal and urinary disorders
Reproductive system and breast disorders
Respiratory, thoracic, and mediastinal disorders
Skin and subcutaneous tissue disorders
Social circumstances
Surgical and medical procedures
Vascular disorders

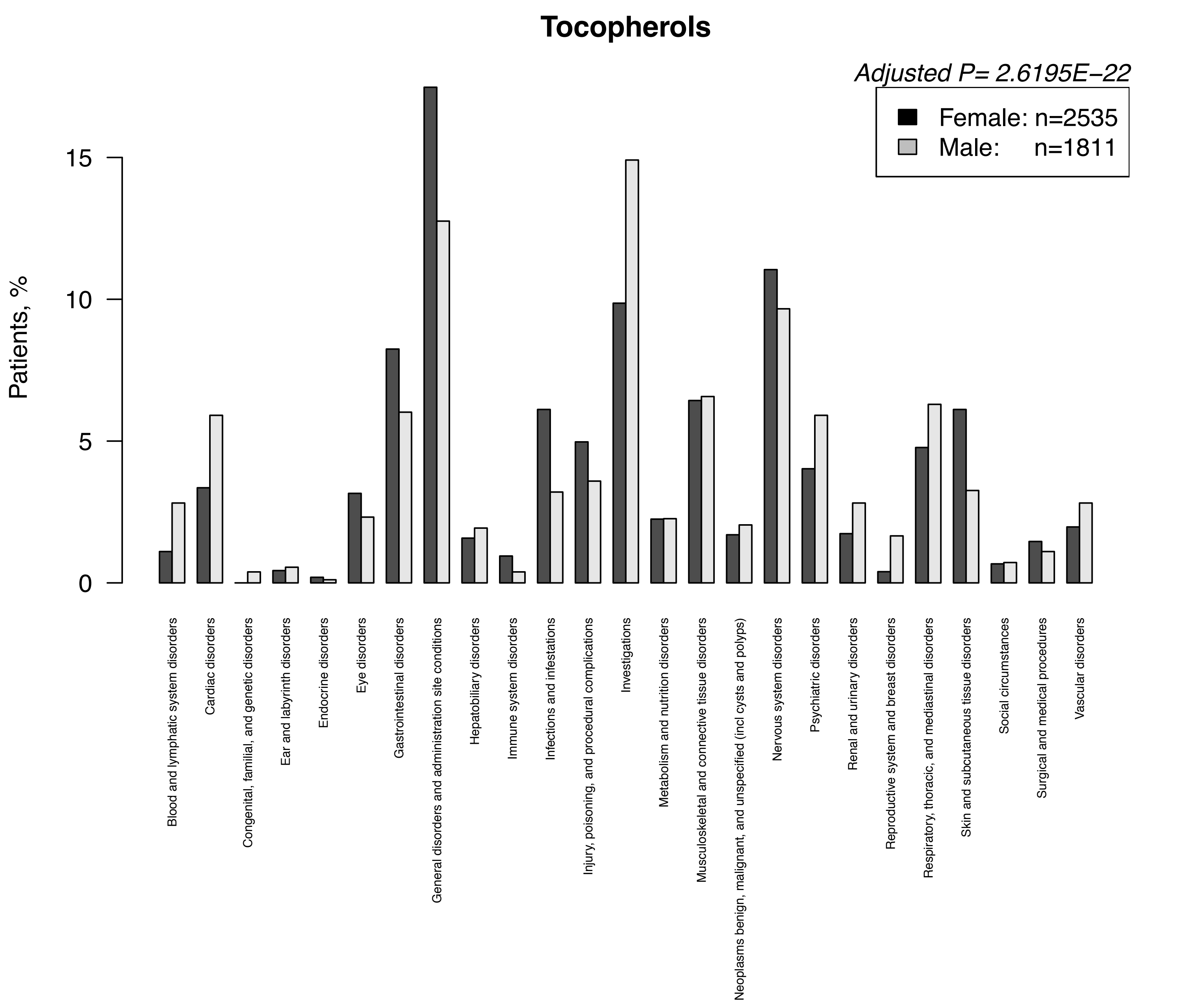

Tocopherols
Adjusted P= 2.6195E−22
Female: n=2535
Male: n=1811
Patients, %
15
10
5
0
Blood and lymphatic system disorders
Cardiac disorders
Congenital, familial, and genetic disorders
Ear and labyrinth disorders
Endocrine disorders
Eye disorders
Gastrointestinal disorders
General disorders and administration site conditions
Hepatobiliary disorders
Immune system disorders
Infections and infestations
Injury, poisoning, and procedural complications
Investigations
Metabolism and nutrition disorders
Musculoskeletal and connective tissue disorders
Neoplasms benign, malignant, and unspecified (incl cysts and polyps)
Nervous system disorders
Psychiatric disorders
Renal and urinary disorders
Reproductive system and breast disorders
Respiratory, thoracic, and mediastinal disorders
Skin and subcutaneous tissue disorders
Social circumstances
Surgical and medical procedures
Vascular disorders

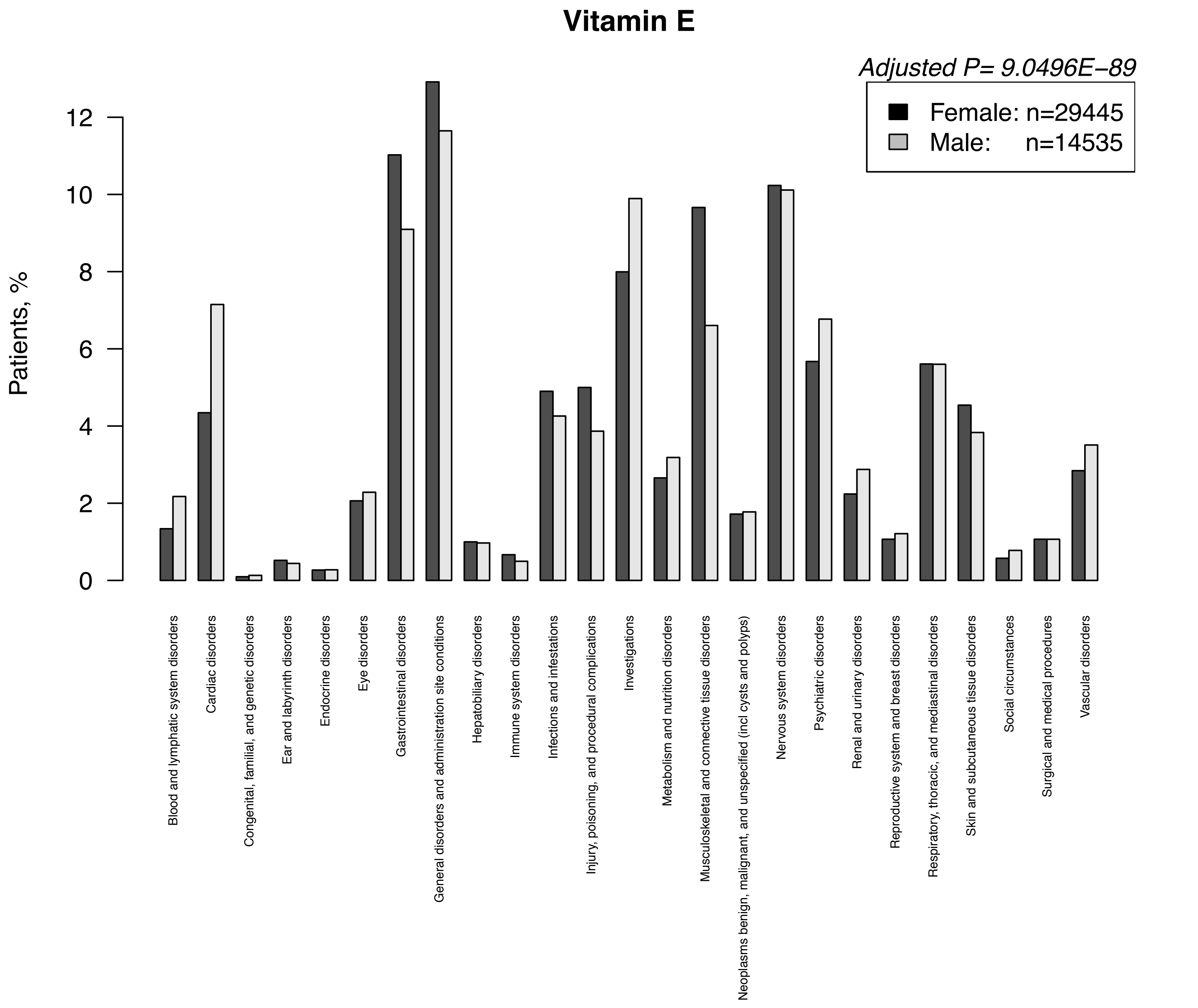
Vitamin E
Adjusted P= 9.0496E−89
Female: n=29445
Male: n=14535
Patients, %
0
2
4
6
8
10
12
Blood and lymphatic system disorders
Cardiac disorders
Congenital, familial, and genetic disorders
Ear and labyrinth disorders
Endocrine disorders
Eye disorders
Gastrointestinal disorders
General disorders and administration site conditions
Hepatobiliary disorders
Immune system disorders
Infections and infestations
Injury, poisoning, and procedural complications
Investigations
Metabolism and nutrition disorders
Musculoskeletal and connective tissue disorders
Neoplasms benign, malignant, and unspecified (incl cysts and polyps)
Nervous system disorders
Psychiatric disorders
Renal and urinary disorders
Reproductive system and breast disorders
Respiratory, thoracic, and mediastinal disorders
Skin and subcutaneous tissue disorders
Social circumstances
Surgical and medical procedures
Vascular disorders
